# Extension of Mitogenome Enrichment Based on Single Long-Range PCR: mtDNAs and Putative Mitochondrial-Derived Peptides of Five Rodent Hibernators

**DOI:** 10.3389/fgene.2021.685806

**Published:** 2021-12-13

**Authors:** Sarah V. Emser, Helmut Schaschl, Eva Millesi, Ralf Steinborn

**Affiliations:** ^1^ Genomics Core Facility, VetCore, University of Veterinary Medicine, Vienna, Austria; ^2^ Department of Behavioral and Cognitive Biology, University of Vienna, Vienna, Austria; ^3^ Department of Evolutionary Anthropology, University of Vienna, Vienna, Austria

**Keywords:** mitogenome, long-range PCR, back-to-back amplification primers, next-generation sequencing, mitochondrial-derived peptides

## Abstract

Enriching mitochondrial DNA (mtDNA) for sequencing entire mitochondrial genomes (mitogenomes) can be achieved by single long-range PCR. This avoids interference from the omnipresent nuclear mtDNA sequences (NUMTs). The approach is currently restricted to the use of samples collected from humans and ray-finned fishes. Here, we extended the use of single long-range PCR by introducing back-to-back oligonucleotides that target a sequence of extraordinary homology across vertebrates. The assay was applied to five hibernating rodents, namely alpine marmot, Arctic and European ground squirrels, and common and garden dormice, four of which have not been fully sequenced before. Analysis of the novel mitogenomes focussed on the prediction of mitochondrial-derived peptides (MDPs) providing another level of information encoded by mtDNA. The comparison of MOTS-c, SHLP4 and SHLP6 sequences across vertebrate species identified segments of high homology that argue for future experimentation. In addition, we evaluated four candidate polymorphisms replacing an amino acid in mitochondrially encoded subunits of the oxidative phosphorylation (OXPHOS) system that were reported in relation to cold-adaptation. No obvious pattern was found for the diverse sets of mammalian species that either apply daily or multiday torpor or otherwise cope with cold. In summary, our single long-range PCR assay applying a pair of back-to-back primers that target a consensus sequence motif of Vertebrata has potential to amplify (intact) mitochondrial rings present in templates from a taxonomically diverse range of vertebrates. It could be promising for studying novel mitogenomes, mitotypes of a population and mitochondrial heteroplasmy in a sensitive, straightforward and flexible manner.

## Introduction

Vertebrate mitochondria harbour a double-stranded circular mitogenome with a general size between 14 and 20 kb (Kolesnikov and Gerasimov, 2012). It is maternally inherited and exists in a highly polyploid state. The cellular copy number of mitochondrial DNA (mtDNA) ranges from ∼400 in peripheral blood mononuclear cells (O’Hara et al., 2019) over 6,000 or 7,000 copies in cells with high ATP requirement such as hepatocytes or cardiomyocytes (Miller et al., 2003; Yin et al., 2004), respectively, up to >200,000 in mammalian oocytes (Cree et al., 2008). Typically, the vertebrate mitogenome has a highly conserved organization with only 37 canonical genes that encode 13 of 83 protein subunits involved in electron transport and oxidative phosphorylation, 22 transfer RNAs (tRNAs), two ribosomal RNAs (rRNAs), a minor noncoding region (NCR) containing the origin of DNA replication for the light (guanine-poor) strand (*O*_*L*_) and one longer NCR also referred to as the control region. The main NCR harbours the promoters for polycistronic transcription, one for each mtDNA strand, the replication origin of the heavy (guanine-rich) strand (*O*_*H*_), a triple-stranded displacement-loop (D-loop) structure incorporating the mitochondrial single-stranded DNA molecule *7S-DNA* and other prominent elements such as the conserved sequence boxes *CSB1-3* or the termination-associated sequence (*TAS* (Gammage and Frezza, 2019)). Occasionally, variable numbers of tandem repeats (VNTRs; *alias*: minisatellites) are inserted within the main NCR (Lunt et al., 1998) or in closed proximity between two tRNA genes (Gorički and Trontelj, 2006; Shu et al., 2018). Short tandem repeats (STRs; *alias*: microsatellites), rarer cases of mtDNA length polymorphism, have also been reported for the main NCR (Sbisà et al., 1997; Feeroz et al., 2008).

Complexity is added to the genome by ‘dwarf’ short or small open reading frames (‘dwarf’ sORFs or smORFs) of ∼20 amino acids (Aspden et al., 2014). These “Russian doll genes” located in the rRNA genes *MT-RNR1* and *MT-RNR2* as well as in the protein-coding gene *MT-CO1* encode a group of dwarf peptides termed MDPs. They comprise MOTS-c (mitochondrial ORF of the 12S rRNA type-c; (Lee et al., 2015), humanin (Hashimoto et al., 2001), the small humanin-like peptides 1 to 6 (SHLP1 to 6 (Cobb et al., 2016)), and gau microprotein (gene antisense ubiquitous in mtDNAs (Faure et al., 2011)). A unique feature of MDPs represents the combination of intra-mitochondrial transcription with cytoplasmic translation according to the nuclear genetic code (Cobb et al., 2016; Lee et al., 2016). In case of human MOTS-c, the “gold-standard” technology of liquid chromatography-mass spectrometry successfully detected the synthetic peptide used as positive assay control. However, the test failed to detect the circulating cell-free form of MOTS-c in human plasma samples irrespectively of the success in ELISA-based measurement (Knoop et al., 2019). MDPs regulate apoptosis and metabolic homeostasis, insulin sensitivity and obesity, reduce oxidative stress and inflammation, extend life expectancy, signal retrograde communication of mitochondria with the nucleus, protect against cancer and Alzheimer´s disease, show decreasing expression with age (Cobb et al., 2016; Kim et al., 2017; Lu et al., 2019) and bear considerable potential to be misused as a performance-enhancing drug in elite sports (Thevis and Schänzer, 2016). Considering their key functionalities in the human organism, the existence of orthologous MDPs in other vertebrate species is implicated.

Whole-genome sequencing (WGS) represents a straightforward approach for assembling the mitochondrial genome (Feldmeyer et al., 2010). Low-pass, shallow sequencing of a genome, termed ‘genome skimming’, can be used to assemble the entire mitogenome from <1% of the reads (Liu et al., 2016). Concomitantly, this shotgun sequencing approach delivers valuable genetic information about the high-copy fraction of the nuclear genome such as rRNA sequences, transposons and high-copy-number genes (Trevisan et al., 2019; Bohmann et al., 2020; Malukiewicz et al., 2021; Margaryan et al., 2021). Reliable mitogenome assembly can be obtained from genome skimming data (∼1 Gb) at an estimated cost of sequencing of 25 € per specimen (∼100 samples per lane, 200 × mtDNA depth; pricing of 2019 (Margaryan et al., 2021)). This would be equivalent with less than 17.3 € (20 $) per 1 Gb on Illumina’s NovaSeq 6,000 Sequencing System according to the current pricing.

However, this calculation does not consider the recent recommendation of the Vertebrate Genomes Project (www.vertebrategenomesproject.org) to assemble a mitogenome by combining WGS long reads for structural accuracy and WGS short reads for base calling accuracy (Formenti et al., 2021). The so-called ‘hybrid assembly’ helps to cope with the widespread presence of sequence duplications and repeats occurring in vertebrates mitogenomes, namely the (prevalent) short repeats of the NCR, but especially the (rarer) relatively large repetitive regions (Gibb et al., 2007; Satoh et al., 2016; Rhie et al., 2021). Third-generation approaches for long-read sequencing such as Oxford Nanopore technology or PacBio sequencing are options of choice to overcome this challenge (Kingan et al., 2019; Dhorne-Pollet et al., 2020). Moreover, the homology between NUMTs and mtDNA can confound the outcome of next generation sequencing (NGS) pipelines that align short reads of DNA to the reference genome based on sequence similarity (Maude et al., 2019). NUMTs represent a general evolutionary trend across vertebrate species (Puertas and González-Sánchez, 2020). They can occur at up to ∼1,000 integration sites (Calabrese et al., 2012; Dayama et al., 2014), vary in size from smaller fragments to one or several full-length mitochondrial integrations like in case of a bat species (Shi et al., 2017) or healthy humans (Lutz-Bonengel et al., 2021), share varying degrees of homology up to complete identity with parental mtDNA depending on the evolutionary time of the insertion event (Dayama et al., 2014; Wei and Chinnery, 2020).

**FIGURE 1:**
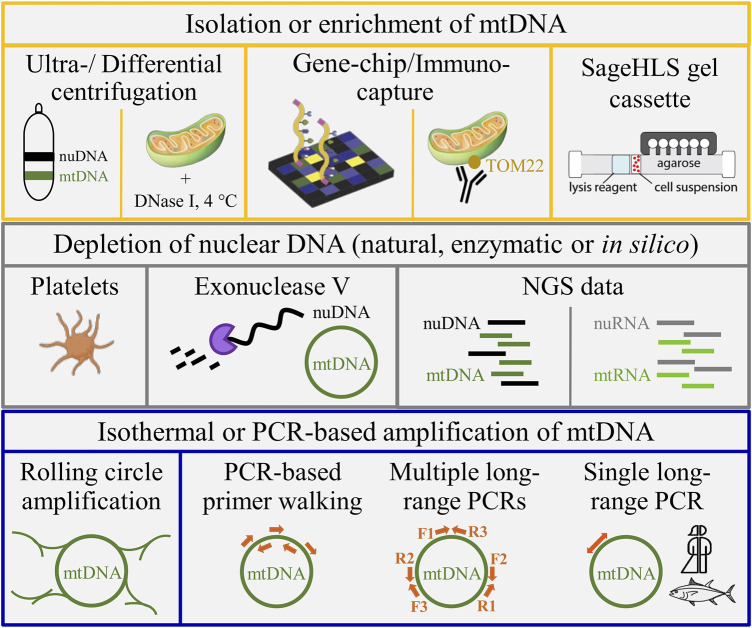
Alternative approaches for mtDNA enrichment. Upper panel from left to right: CsCl gradient ultracentrifugation, isolation of native mitochondria with nucleolytic removal of surrounding nuclear DNA (nuDNA) contamination, mtDNA capture using a gene chip, TOM22-based immunocapture, and automated extraction of mtDNA in a proprietary agarose gel cassette (SageHLS™ system; Sage Science, Beverly, MA, United States). Central panel from left to right: natural depletion of nuDNA (platelet icon from www.biorender.com), enzymatic (exonuclease) depletion of nuDNA, and joint sequencing of nuDNA and mtDNA or nuclear RNA (nuRNA) and mitochondrial RNA (mtRNA) by NGS analysis (DNA or RNA sequencing, respectively). Bottom panel from left to right: rolling-circle amplification, primer walking, several long-range PCRs to cover the mitogenome (F & R: amplification primers), and single-amplicon long-range PCR with back-to-back primers that was extended within this study beyond application in humans (symbolized by the gender-neutral person icon) and ray-finned fishes (class *Actinopterygii*).

There are several ways for reducing the amount of undesired nuclear DNA (nuDNA) reads, thus solving the redundancy challenge in sequencing, mapping, and genotyping caused by NUMTs (Figure 1). Classically, contaminating nuDNA was depleted from a total extract of cellular DNA based on its different buoyant density using caesium chloride density gradient ultra-centrifugation (Carr and Griffith, 1987). While this approach is effective, there are several drawbacks such as the use of dangerous and toxic chemicals (CsCl and nucleic acid stain), the patience and dexterity to collect the stained mtDNA band, the force generated during the ultracentrifugation step and the expenditure of time. Time effectiveness and sample throughput can be increased by removing contaminating nuDNA by deoxyribonuclease I (DNase I)-treatment of native mitochondria obtained by differential and gradient centrifugations ((Chase and Pring, 1986; Zimmerman et al., 1988; Skubatz and Bendich, 1990); Figure 1). Nowadays, simpler and more straightforward extraction and enrichment of mtDNA without the need of (ultra)centrifugation can be achieved by immunocapture of mitochondria from a cell lysate (*e.g*., based on TOM22 (Franko et al., 2013)) or by electrophoretic separation of a cellular extract in a proprietary agarose gel cassette (Boles and Houde, 2019). Enzymatic linearization of the mitochondrial ring in combination with electrophoretic separation can be used to extract mtDNA from a mixture of total cellular DNA (Wiesner et al., 1991). However, lack of *a priori* knowledge on the (species-specific) restriction endonuclease that cuts only once in the respective circular mtDNA, *e.g*., restrictases *AleI*, *BbvCI* or *PacI*, argues against universal application of the method for sequencing novel mitogenomes. The size and conformation (supercoiling) similarities between conventional bacterial plasmids and mammalian mtDNA inspired introduction of rolling circle amplification (Tang and Hyman, 2005; Simison et al., 2006). The approach produced a ∼ 2,000-fold enrichment of mtDNA before NGS analysis (Quispe-Tintaya et al., 2013; Weissig and Edeas, 2015). Alternatively, interfering NUMTs can be excluded from the very start by targeting material naturally depleted of nuDNA such as thrombocytes (cellular mtDNA content of platelets: ∼1.6 copies (Urata et al., 2008; Wang et al., 2018)) or plasma that was recently reported to contain free-floating mitochondria (Al Amir Dache et al., 2020) and circular mtDNA derived from the haematopoietic system (Ma et al., 2019). The use of plasma DNA as natural source of nuclear depletion is hampered by the fact that direct NGS analysis would produce unnecessary nuDNA reads since extrachromosomal circular DNA was found to be released from tissues into the circulation (Kumar et al., 2017). This phenomenon impairs sequence analysis of circular mtDNA enriched by random-hexamer primed isothermal rolling circle amplification (Simison et al., 2006) and the enrichment of circular mtDNA by exonuclease V-based depletion of nuDNA (Gould et al., 2015; Zhu et al., 2017; Dhorne-Pollet et al., 2020). The latter likely explains the admixture of 70 to 41% of nuDNA reads following exonuclease depletion of linear DNA and phi29 polymerase-mediated isothermal amplification (Dhorne-Pollet et al., 2020). Another PCR-free pipeline for mtDNA enrichment and reduction of sequencing volume used a gene-capture chip that produced an up to 100-fold increase of the mtDNA fraction (Liu et al., 2016). The advent of RNA sequencing introduced another option for mitogenome assembly with less bias from NUMTs. Notably the Illumina platform has led to an explosion in the number of samples that can be studied at transcriptomic level at relatively low cost. Mitochondrial protein-coding and rRNA genes can be obtained from almost any transcriptomic sequencing experiment. When sequencing is performed in sufficient depth, even the complete mitogenome can be recovered (Moreira et al., 2015; Plese et al., 2019).

To avoid NUMT confounding biases, this study established an assay to sequence the entire mtDNA from a single amplicon produced by long-range PCR, a method that was used before for this purpose. Most long-range PCR approaches covered the entire mitogenome with one to three overlapping amplicons (Zhang et al., 2012; Burgstaller et al., 2014; Kloss-Brandstätter et al., 2015; Seneca et al., 2015; Paijmans et al., 2016; Deiner et al., 2017; Taylor et al., 2020). Nowadays, NGS-based determination of the entire mitogenome from a single long-range PCR product has become routine practice in human medicine, *e.g.,* for diagnosing patients with clinically suspected mtDNA disease (Zhang et al., 2012; Luo et al., 2018). The approach was also successfully applied to address environmental DNA (eDNA)-related issues in ray-finned fishes (class *Actinopterygii* (Deiner et al., 2017)).

Here we extended the approach to various kinds of vertebrates including mammals, birds, reptiles, amphibians, and bony fishes. Templates from five hibernator rodents were used for testing the set of universal back-to-back primers capable of amplifying a whole mitogenome. Hibernation is characterized by multiday torpor as a highly efficient energy-saving strategy to survive predictable periods of low nutrient availability and cold ambient temperature. It is found in many mammals such as rodents, bears, hedgehogs, bats, marsupials (Heldmaier et al., 2004; Geiser et al., 2014) and even primates (Dausmann et al., 2004; Blanco et al., 2021). Other strategies used by endothermic mammals for coping with low temperature include increased heat production via shivering and non-shivering thermogenesis, reduction of heat loss from the body surface with the help of fur (Hart, 1956) and/or subcutaneous adipose tissue (Tattersall et al., 2012), or modifications of the blood system such as sequence changes of haemoglobin genes (Campbell et al., 2010), counter-current heat exchange (Ninomiya et al., 2011) or the heart’s pumping ability mediated by a missense mutation in the *NRAP* gene (Buggiotti et al., 2021). Mitogenome adaptations include polymorphisms in mitochondrial genes encoding subunits of the respiratory chain (Balloux et al., 2009; Awadi et al., 2021) and stem-loop formation(s) in the major NCR (Melo-Ferreira et al., 2014). Amino-acid changes associated with cold adaptation can lead to heat dissipation due to uncoupling between OXPHOS and ATP synthesis (Wijers et al., 2008). Here, we analysed the novel mitogenomes with special emphasis on these putative footprints of cold-adaptive evolution. Intraspecies mtDNA variants of individuals supposed to be not cold-adapted were selected for comparison. In addition, we addressed the evolutionary conservation of the MDPs predicted *in silico*.

## Materials and Methods

### Biological Material and Ethical Aspects

Phylogenetic categories in this article were derived from the current version of the NCBI Taxonomy database, a curated set of names and classifications that is regarded to be the phylogenetic taxonomy standard for GenBank's source organisms (DOI: 10.1093/nar/gkr1178; http://www.ncbi.nlm.nih.gov/taxonomy). Five rodent hibernators, *Eliomys quercinus* (garden dormouse), *Marmota marmota* (European marmot), *Muscardinus avellanarius* (hazel dormouse), *Spermophilus citellus* (European souslik) and *Urocitellus parryii* (Arctic ground squirrel) served as model species for this Genomic Assay Technology study (Table 1).

**TABLE 1:** Target species of the long-range PCR assay amplifying vertebrate mtDNA with a pair of back-to-back primers.

Latin (common) name	Geographic origin	Tissue	Storage temperature	Isolation kit^a^	Source
***Eliomys quercinus* (Garden dormouse)**	Niederfischbach (Rhineland-Palatinate, Germany; 0°51′20″N, 7°52′40″E)	liver	−20°C	2	University of Veterinary Medicine (Vienna, Austria)
***Marmota marmota* (European marmot)**	Schröcken (Vorarlberg, Austria; 47°15′00″N, 10°05′00″E)	liver	−20 °C	2	University of Veterinary Medicine (Vienna, Austria)
***Muscardinus avellanarius* (Hazel dormouse)**	Mechelen (Flemish Region, Belgium; 51°01′40″N, 4°28′50″E)	tail	4°C (in alcohol)	2	Non-governmental organization Natuurpunt (Mechelen, Belgium)
***Spermophilus citellus* (European suslik)**	Bisamberg (Lower Austria, Austria; 48°19′15″N, 16°21′47″E)	heart	−20°C	2	University of Vienna (Austria)
***Urocitellus parryii* (Arctic ground squirrel)**	Atigun River (Northern Alaska, United States; 68°27′N, 149°21′W)	liver	−20°C	1	Institute of Arctic Biology, University of Alaska Fairbanks (United States)

a Kit 1: QIAamp Fast DNA Tissue Kit (Qiagen, Hilden, Germany), kit 2: Tissue & Cell Genomic DNA Purification Kit (GeneMark, Taichung, Taiwan).

In addition, tissue of an individual of *Cricetus cricetus* (black-bellied hamster) deceased in the Vienna area (Austria) was used to increase resolution of phylogenetic reconstruction.

*E. quercinus* and *S. citellus* originated from breeding facilities that were approved by the Austrian government and the respective local authorities (BMBWF-68.205/0137-WF/V/3b/2014, MA22-1854/2012). Tissue of *M. marmota* was obtained from a local Austrian hunter in the course of regulated population-management activity. An individual of *M. avellanarius* was stored after its natural death by Natuurpunt Mechelen (Belgium), a non-governmental organisation. Animal trapping, housing, care and sampling related to *U. parryii* was carried out in accordance with approved IACUC protocols (#1081763 and #340270) through the University of Alaska Fairbanks and with state (ADF&G #17–100) and federal (BLM #F-94817) permits.

The procedures related to *C. cricetus* were performed in accordance with guidelines of the European Union (EU) for the protection of animals used for scientific purposes (Directive 2010/63/EU) and were approved by the ethics committee of the Faculty of Life Sciences, University of Vienna (2015-010), the Austrian Federal Ministry of Education, Science and Research (GZ: BMWFW-66.006/0013-WF/V/3b/2015), and the City of Vienna (MA22-2484/10, MA22-310/11).

The study was reviewed and approved by the board on animal ethics and experimentation of the Faculty of Life Science of the University of Vienna (#2021-009).

### Deoxyribonucleic Acid Extraction and Single Long-Range Polymerase Chain Reaction With Pan-Vertebrate Back-To-Back Primers

**FIGURE 2:**
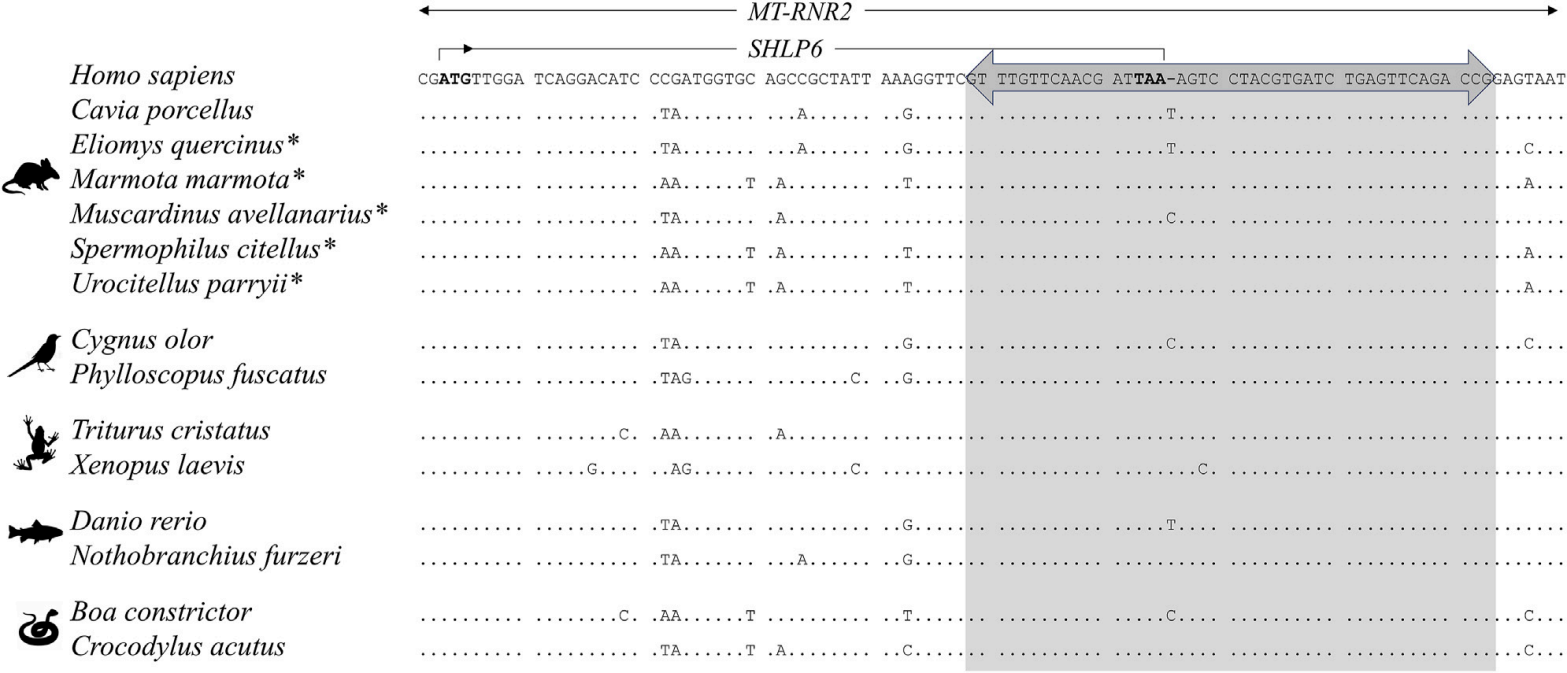
Target sites of the universal back-to-back primers are located in the conserved region of *MT-RNR2* (grey box) and include part of the *SHLP6*-encoding sORFs. Representatively, two species were selected for different classes of the subphylum Vertebrata. Asterisk: target species of this study; dot: identity with human sequence; hyphen: nucleotide gap; specific base: single-nucleotide polymorphism (SNP). Note that the back-to-back primers are completely homologous to the main mtDNA haplotypes of *Homo sapiens* (data not shown).

DNA of the five hibernator rodents was isolated using silica-column-based procedures (Table 1). Universal back-to-back primers for single long-range PCR were derived from an alignment of representative mitogenomes of the five groups of the vertebrate phylum, *i.e*., bony fishes, amphibians, reptiles, birds and mammals (primers: 5′-TAC GTG ATC TGA GTT CAG ACC G and 5′-GTA GGA CTT TAA TCG TTG AAC AAA C; Figure 2). The 20-μL reaction for mitogenome amplification consisted of 1 × Phusion HF Buffer containing 1.5 mM MgCl_2_ (Thermo Fisher Scientific, Waltham, MA, United States), 0.2 mM of each dNTP (Solis Biodyne, Tartu, Estonia), 0.5 mM of additional MgCl_2_, 1 × Solution S (Solis Biodyne), 0.5 μM of each primer, 0.6 units Phusion Hot Start II DNA polymerase (Thermo Fisher Scientific) and ∼10 ng genomic DNA. The cycling conditions consisted of 98°C for 2 min, followed by 30 cycles of 95°C for 10 s, 66°C for 25 s and of 72°C for 500 s and final extension at 72°C for 5 min. Specificity of amplification was assessed by electrophoresis on a 0.8% agarose gel stained with GelGreen™ Nucleic Acid Stain (Biotium, Hayward, CA, United States) and run at 10 V/cm. The band of the expected size was purified using Hi Yield^®^ Gel/PCR DNA Fragment Extraction Kit (SLG, Gauting, Germany).

### Next-Generation Sequencing, Bioinformatic Analysis and Annotation

**FIGURE 3:**
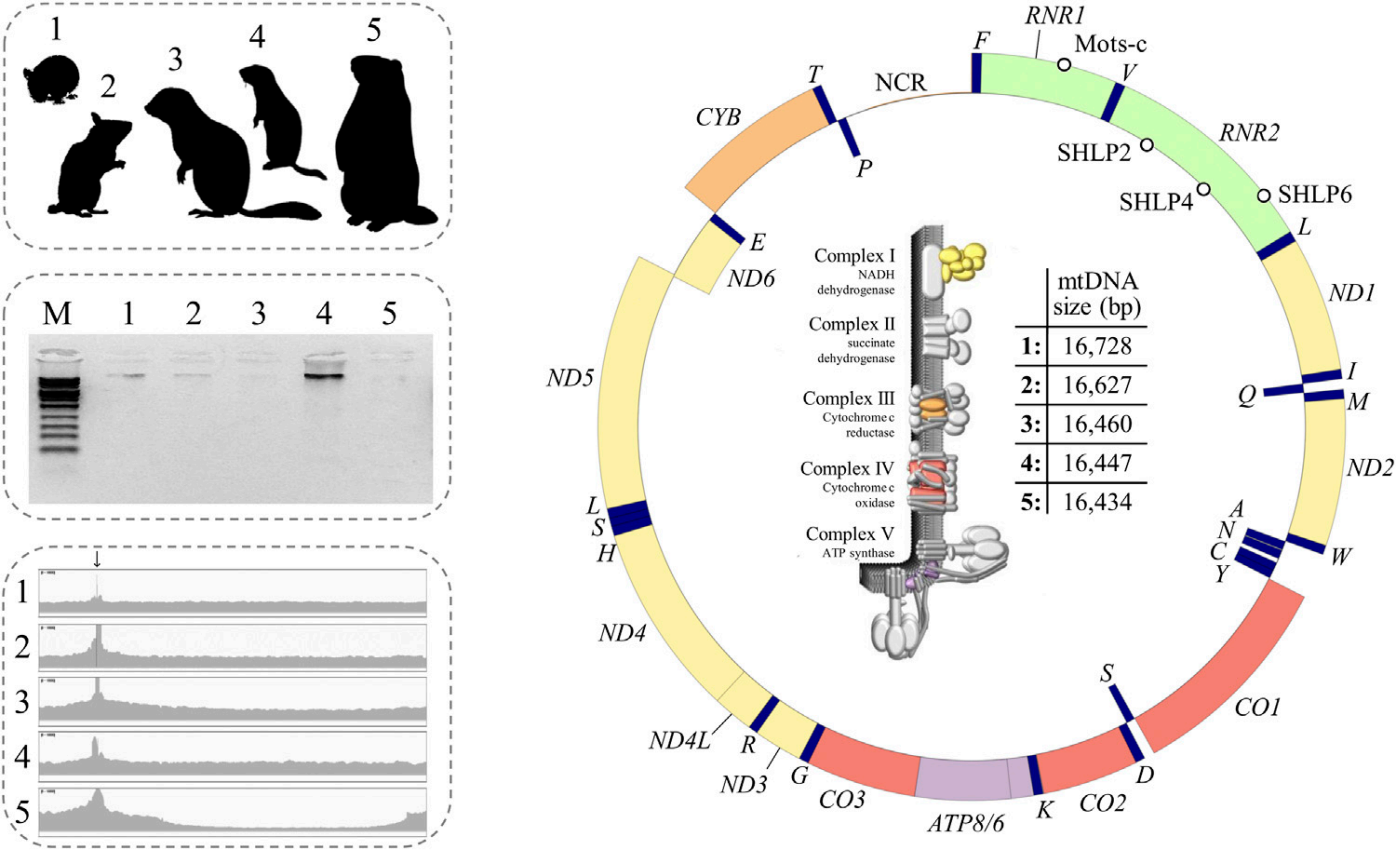
Summary of this study including hibernator model species, electrophoretic separation of amplification products, sequence uniformity coverage plot, mitochondrial gene architecture, and a tabular view summarizing the slightly different mitogenome sizes (from left to right). The functional map of mtDNA depicts the similarity of gene architectures (identical gene order). The scheme of the circular mitogenome was constructed with the GenomeVx Web Browser ((Conant and Wolfe, 2008); http://wolfe.ucd.ie/GenomeVx/). The outside and inside of the ring indicate that the gene is encoded by the heavy and light chains of the mtDNA ring, respectively. Colour depicts assignation of a protein subunit to a particular complex of the electron transport chain (illustration kindly provided by Scott Lujan (Lujan et al., 2020)). Note that complex II is exclusively composed of nuclear subunits. Empty circles: MDPs. The tRNAs were designated by single-letter amino acid abbreviation. Origins of heavy and light strand replications and *7S-DNA* are not shown.

100 ng of the amplified long-range PCR product was used for library preparation with the NEBNext^®^ Ultra II FS DNA Library Prep Kit for Illumina^®^ and the NEBNext^®^ Multiplex Oligos for Illumina^®^ (Index Primers Set 1; New England Biolabs, Ipswich, United States) according to manufacturer’s instruction. Briefly, fragmentation was performed at 37°C for 6 min, adapters were tenfold diluted, and four cycles were run for PCR amplification before PCR clean-up and quality control. Libraries were quantified using the Quantus™ Fluorometer (Promega, Mannheim, Germany) and run on the DNA fragment analysis system BioAnalyzer 2100 (Agilent). Final libraries were pooled and sequenced on a MiSeq benchtop sequencer (Illumina, Eindhoven, Netherlands) with MiSeq Nano Reagent Kit v2 500 cycles chemistry in 2 × 250 mode at 10 pM with 5% PhiX control. FastQ raw data were used for data analysis. Sequencing reads were assembled using the genome assembly algorithm SPAdes (version 3.13.1; (Nurk et al., 2013); http://cab.spbu.ru/software/spades/). The coverage plots were visualized with the IGV desktop application (version 2.8.6; Figure 3 (Robinson et al., 2011)).

Novel mitogenomes were annotated using the MITOS web server ((Bernt et al., 2013); http://mitos.bioinf.uni-leipzig.de/index.py).

### Discovery of sORF-Encoding Mitochondrial-Derived Peptides

The sORFs of all known MDPs were determined using the Translate tool of the bioinformatics resource portal ExPASy ((Artimo et al., 2012); Swiss Institute of Bioinformatics, https://web.expasy.org/translate/). The prediction of MDPs was restricted to the rule that intra-mitochondrial transcription is combined with cytoplasmic translation according to the standard (nuclear) genetic code (Cobb et al., 2016; Lee et al., 2016). Considering the size range of the putative mitochondrial sORFome (9–40 amino acids (Miller et al., 2020)), an arbitrary cut-off of eight amino acids was applied as minimal sORF length.

### Validation of Back-To-Back Primer’s Binding Sites by Bidirectional Sanger Amplicon Sequencing

Consensus primers adjacent to the back-to-back primers for mitogenome amplification were designed based on the mtDNA sequence assembled *de novo* (5′-GTT TAC GAC CTC GAT GTT G and 5′-TAG ATA GAA ACC GAC CTG G). Sequencing of the resulting PCR products was outsourced to a commercial service provider (LGC Genomics GmbH, Berlin, Germany). Electrochromatograms were assembled and aligned with CodonCode Aligner (version 7.1.2; CodonCode Corporation, Centerville, MA, United States).

### Determination of the *C. Cricetus* Mitogenome Using Genome Skimming

To deeper illustrate the genetic relationship of our hibernator models, the mtDNA sequence of *C. cricetus* was determined *de novo* using genome skimming. Next-generation sequencing, bioinformatic analysis and annotation were performed as described (see above).

### Phylogenetic Relationship Using Maximum Likelihood Analysis and Bayesian Inference

Nucleotide sequences of the entire mtDNA were retrieved from NCBI or added from own data, aligned using the CLC Sequence Viewer 8.0 software (Qiagen, Hilden, Germany). We excluded the major NCR following the recommendation that the control region of mtDNA should be avoided as a tool for constructing phylogenies or in dating evolutionary events (Slate and Phua, 2003). Since the pervasiveness of mtDNA recombination in animals is considered to be a very rare phenomenon (White et al., 2008; Duchêne et al., 2011; Yamamoto et al., 2020), linkage of mitochondrial regions was assumed meaning that they are expected to share the same phylogeny. Therefore, phylogenetic reconstruction was based on the three sets of coding sequences—protein-, rRNA- and tRNA-coding genes (Chan et al., 2010) without partitioning (Castañeda-Rico et al., 2020) using the maximum-likelihood (ML) approach performed in IQ-TREE (release 1.6.12 of August 15, 2019; (Nguyen et al., 2015; Kalyaanamoorthy et al., 2017; Hoang et al., 2018); http://iqtree.cibiv.univie.ac.at/). The best-fitting substitution model, the TIM2+F+I+G4 model, was selected according to the Bayesian Information Criterion using ModelFinder (Lanfear et al., 2012; Chernomor et al., 2016; Kalyaanamoorthy et al., 2017). Nodal support values were assessed with 1,000 bootstrap replicates. In parallel, we compared the bootstrap support values for the mtDNA sequence dataset partitioned into protein-coding, rRNA and tRNA genes using the best-fitting substitution models TIM2+F+I+G4, TIM2+F+I+G4 and TIM2+F+G4, respectively.

Alternatively, phylogenetic reconstruction of the dataset was assessed by Bayesian inference analysis using MrBayes version 3.2.7a ((Ronquist et al., 2012); http://nbisweden.github.io/MrBayes/) and the GTR + I + Γ model. Two simultaneous analyses were run. The Markov chain Monte Carlo analysis was performed with Markov chains for 10 million generations with trees sampled every 1,000 generations. Consensus topology and posterior probabilities value were produced after discarding (burn-in of 25%) the early resulting, typically poorer quality trees from the simulation.

The ML and Bayesian consensus trees were viewed and edited using the Interactive Tree of Life (iToL) version 6.3 ((Letunic and Bork, 2021); https://itol.embl.de/).

## Results

### Vertebrate Universal Back-To-Back Primers for Long-Range Polymerase Chain Reaction of the Mitogenome

This study extended the use of long-range PCR allowing to amplify the mitogenome in a single amplicon. A sequence segment that partly overlapped with the sORF of *SHLP6* hosted by *MT-RNR2* was found to be universally conserved across vertebrate mitogenomes and used as the binding site for the back-to-back primers (Figure 2). The approach was demonstrated for *de novo* determination of mitogenomes of the five hibernator rodents *Eliomys quercinus, Marmota marmota*, *Muscardinus avellanarius, Spermophilus citellus* and *Urocitellus parryii* (for common names see Table 1).

### Next Generation Sequencing Analysis of the Amplicon Covering the Mitogenome

Single long-range PCR successfully produced the expected amplicon across the five target species (Figure 3) including *E. quercinus* and *M. avellanarius* that exhibited a single-base insertion at the binding site of one of the amplification primers (Figure 2). Their sequencing on a single MiSeq lane resulted in 408,245 ± 41,396 reads with a proportion of 95.7 ± 2.3% mitochondrial reads. Except for the obligatory peak at the primer binding site, coverage depth per base was similar across the genome (Figure 3). Analysis by Sanger sequencing confirmed the target sequence of the pan-vertebrate back-to-back primers.

### Genome Skimming for *C. Cricetus*

Muscle DNA of the *C. cricetus* individual showed a proportion of 2.2% reads that aligned to the mitogenome (average coverage of mtDNA sequence: 51.9-fold). The mitogenome variant was 98.7% homologous to a Russian (RUS) haplotype (GenBank: NC_037,888.1 or MF034880 (Ding et al., 2020)). Further details are provided in Supplementary Table S1.

### Phylogenetic Analysis

**FIGURE 4:**
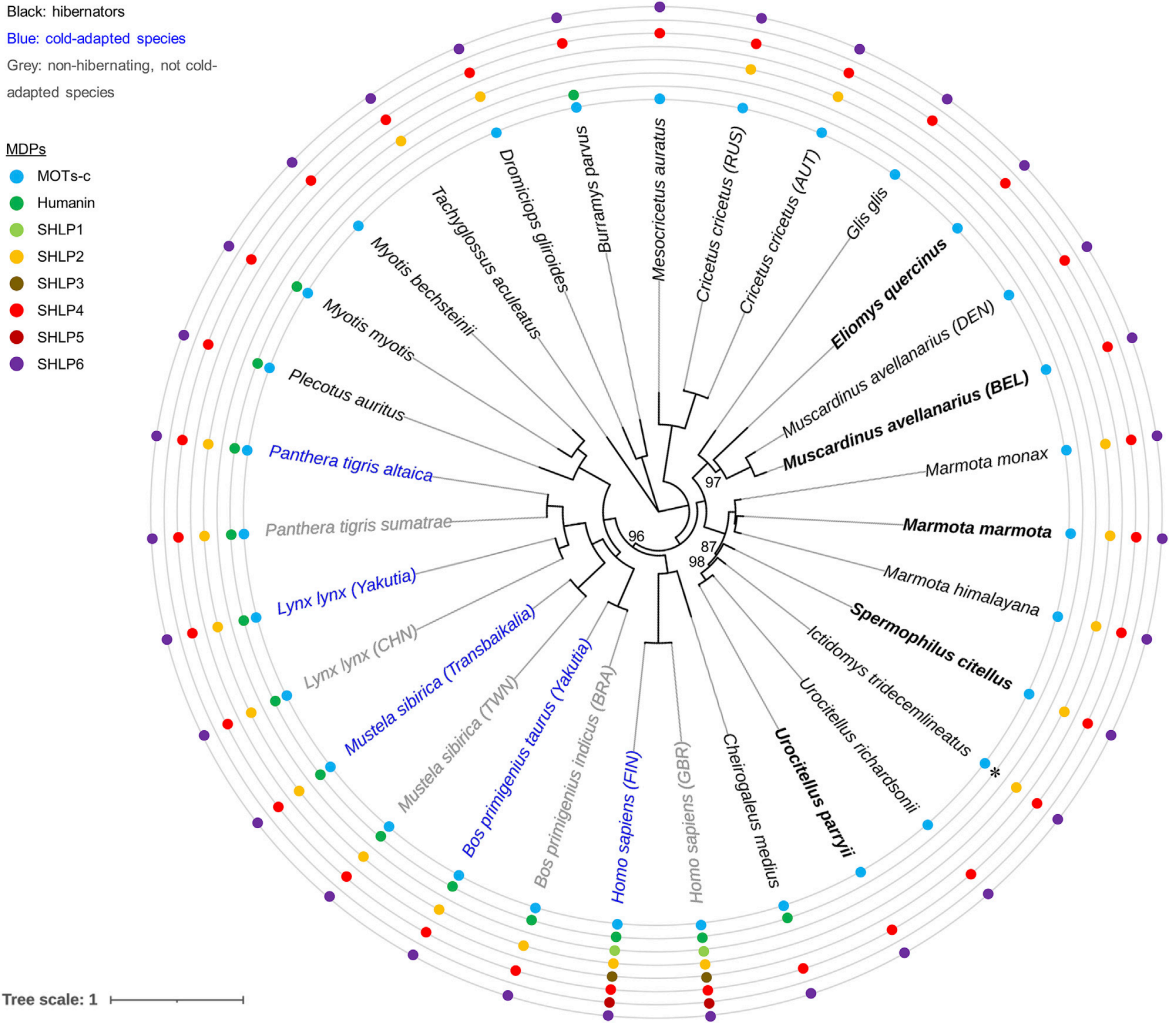
Inference of evolutionary and mtDNA-based relationships among our five hibernator rodent models. Summary on predicted MDPs and ML phylogenetic reconstruction using NCR-depleted mitogenomes without partitioning. Rodent hibernators used for extending mitogenome enrichment based on single long-range PCR are highlighted in bold. *Cricetus cricetus*, a facultative hibernator (Weinhold, 2015), is represented by a Russian (RUS) haplotype (GenBank: NC_037,888.1 or MF034880 (Ding et al., 2020)) originating from Nalchik (republic Kabardino-Balkariya, southwestern Russia), and by an Austrian (AUT) haplotype determined by genome skimming within this project (GenBank: MF405145.2; sequence homology: 98.7%). Note that a second mitogenome was also available for the hazel dormouse (Danish (DEN) haplotype, mtDNA divergence: 9.1%). Bootstrap support measures were calculated from 1,000 tree replicates; only values below 100% are depicted. GenBank accession numbers of the presented mitogenomes are provided in Supplementary Table S4. Pairs of subspecies, intraspecies cohorts or haplogroups were selected to illustrate the different degrees of adaptation to cold temperatures (cattle, Eurasian lynx, human*,* Siberian weasel and tiger). Prediction of MDPs (specified by coloured circles) was restricted to the rule that intra-mitochondrial transcription is combined with cytoplasmic translation according to the standard (nuclear) genetic code (Cobb et al., 2016; Lee et al., 2016). Eight amino acids were applied as an arbitrary minimum for sORF size. The asterisk depicts the only deviation from this general rule—the experimental proof of humanin translation according to the vertebrate mitochondrial code in case of the thirteen-lined ground squirrel (Szereszewski and Storey, 2019). We note that this illustration neither specifies length and sequence differences of MDPs nor issues expression of MDPs from nuDNA that cannot be ruled out in principle. Names of countries are presented as three-letter country codes of the International Organization for Standardization: Brazil (BRA), China (CHN), Finland (FIN), Great Britain (GBR), Taiwan (TWN). The nucleotide alignment used for phylogenetic analysis is available as Supplementary File 1. Similar bootstrap support was obtained by partitioning the NCR-depleted mitogenome sequences into three sets of coding sequence (mRNA, rRNA and tRNA genes; values of 85–99 (*n* = 5) and 100).

Phylogenetic reconstruction was performed to depict the relationship between the five rodent hibernators targeted in this study in relation to other rodents and representatives of hibernation or cold-adaptation phenotypes (Figure 4). Considering the highly conserved nature of mitogenomic architecture and sequence, we applied a single “grand alignment” strategy for the reconstruction of the NCR-depleted mitogenomes without partitioning. Congruent topologies were inferred by the ML and Bayesian frameworks supported by nodes with high or maximal posterior probability values (≥87%; Figure 4, Supplementary Figure S1). The common partitioning of the mitogenome into the three mtDNA gene sets, mRNA-, rRNA- and tRNA-coding genes, produced similar nodal support (for details see legend to Figure 4).

Optimal bootstrap support was also obtained for two genetic geographies of *M. avellanarius* represented by the mtDNA of a Belgian individual (this study) and a Danish mitogenome that became available during the course of this work (GenBank accessions MT410887.1 or NC_050,264.1). The latter individual of *M. avellanarius* originated from Svendborg, a town in south-central Denmark (coordinates: 55°3′34″N, 10°36′30″E (Margaryan et al., 2021)). Its mitogenome differed slightly in nucleotide frequency and in the length of several genes (three protein-coding genes and five tRNAs) and of the mitogenome overall (Supplementary Table S2). However, the two individuals were considerably different in the nucleotide sequences of the mitochondrial *MT-CYB* gene and of the entire mitogenome (10.3 and 9.9%, respectively). A similar degree of dissimilarity was reported earlier for *MT-CYB* of two genetically distinct lineages of the hazel dormouse (∼11% (Mouton et al., 2017)). This was indicative of species recognition according to the Genetic Species Concept (threshold of *MT-CYB* divergence: > 11% (Bradley and Baker, 2001; Baker and Bradley, 2006)). The two sampling spots, Belgium and Denmark, match the two distinct genetic geographies of *M. avellanarius* located in Western Europe or Central-Eastern Europe and Anatolia, respectively, regarded to be cryptic species (Mouton et al., 2017).

Summarising, the complex phylogeographic structure with at least two lineages assumed for the cryptic *M. avellanarius* species is in line with our phylogenetic reconstruction.

### Candidate Polymorphisms of mtDNA Genes

We focussed our analysis of candidate polymorphisms in mitochondrial OXPHOS genes to amino acid substitutions formerly associated with cold adaptation and living at high altitude (*n* = 4 and 1, respectively). To study their distribution across mammalian species, we composed three sets of species that either cope with cold by using a torpor-free strategy, by entering daily torpor or hibernation, or that occupy habitats with moderate or hot climates where cold adaptation is not regarded a key issue (each: *n* = 29; Supplementary Table S3). A unique pattern was not obvious for the candidate polymorphisms linked to minimum habitat temperature (*MT-ATP6*: 8,701G/A and *MT-ND3*: 10,398G/A of *Homo sapiens,* (Balloux et al., 2009); *MT-ND4*: codon 29 in hare species of the genus *Lepus* (Awadi et al., 2021)).

In addition to these signatures of cold adaptation, we analysed the occurrence of the high altitude-associated amino acid change Y30C/H located in *MT-ND1*, a polymorphism that affected a highly conserved amino acid of the gene. It was reported for three lineages of high-altitude Tibetans of the macrohaplogroup M (Y30H), but not high-altitude Tibetans of haplogroup N (Ji et al., 2012), and the gelada, the high-altitude Ethiopian monkey (Y30C; Wallace, 2015). Surprisingly, two sloth species (genus *Bradypus*), slowly moving mammals inhabiting moderate climatic zones, also harboured this change (Y30H; Supplementary Table S3). It is unclear whether this points to a partial functional overlap between the phenotypes of high-altitude adaptation and slowness of movement caused by the respiratory chain.

### Mitochondrial-Derived Peptides and Their Potentially Translatable sORFs

During the annotation of the novel mitogenomes determined for our five hibernator models, special focus was attributed to the sequences encoding putative MDPs (Figure 4; Supplementary Figure S3; Supplementary Table S4).

In case of MOTS-c, the prediction indicated an evolutionarily conserved core of four amino acids, differences of peptide length and/or the occurrence of a downstream AUG start codon. No reasonable sORF size was predicted for the homologous nucleotide sequences sporadically encoding humanin, SHLP1, 3 and 5. In contrast, the predicted amino acid sequences and sizes of SHLP4 and 6 peptides were highly similar (Supplementary Figure S3). In case of SHLP4, the first twelve amino acids were identical among the target species. For *M. marmota*, *M. avellanarius, S. citellus* and *U. parryii* the prediction indicated a very short peptide of twelve amino acids. A longer one similar to the human SHLP4 was derived for *E. quercinus*. Both size variants were frequently seen across vertebrate phylogeny with slight variation of the longer sORF (Supplementary Figure S4). For SHLP6, two sORF size variants were observed, one with an oligopeptide of nine amino acids and the other one exhibiting the length of the human homologue (20 amino acids). In general, the pattern of the two SHLP6 size variants was consistently found across different vertebrate species (Supplementary Figure S4).

Evolutionary conservation of the gau protein was evaluated a decade ago (Faure et al., 2011). For all species that have been sequenced in this study, translation according to the nuclear genetic code returned no functional start codon (ATA instead of ATG). When the repertoire of mitochondrial start codon variants was extended, the translation yielded a dwarf sORF of 14 amino acids for the sequenced species as well as for human mtDNA (Supplementary Figure S5). Interestingly, except for the terminal amino acid the predicted dwarf peptide covered the synthetic peptide sequence used for raising the monoclonal antibody that was key to prove the colocalization of the gau protein with mitochondria (Faure et al., 2011). In this regard we note that the exact size of the gau protein remains an experimental issue to be solved.

The comparison of intraspecies haplotype pairs differing in the degree of adaptation to a cold climate (cattle, Eurasian lynx, human*,* Siberian weasel and tiger) yielded identical signatures of putative MDPs (Figure 4). Future research should increase cohort sizes for the phenotypes of hibernation, no hibernation and cold adaptation to assess the marker issue more comprehensively.

## Discussion

Out of the various approaches for enriching mtDNA before mitogenome sequencing, this study favoured the use of back-to-back amplification primers considering its simplicity, throughput, universality, as well as flexibility and amount of template source. The assay for mitogenome amplification applicable for fresh or well-preserved blood and tissue samples can be helpful to assess issues of mitogenomics such as point and length heteroplasmy of clinical and forensic samples (Cagnin et al., 2009), evolutionary mechanisms of heteroplasmy (Tikochinski et al., 2020) or intraspecific phylogeography (Avise et al., 1987). Our assay holds promise for tissue samples preserved in ethylenediaminetetraacetic acid (EDTA) (Sharpe et al., 2020), or choline-based ionic liquids (Oosting et al., 2020) as well as for certain applications based on aquatic eDNA (Deiner et al., 2017; Ding et al., 2020). The proof that eDNA derived from macro-organisms inhabiting a water body remains intact at least at the size of vertebrate mitogenomes (∼16 kb) contradicts the former common view that eDNA from communities of living fishes in water is highly degraded (Bohmann et al., 2014). Currently, the assay’s full potential for eDNA that is shed by animals into water, moist soil or air *via* cells, hair, pieces of skin, mucus, gametes, urine, faeces, or free-floating, naked DNA etc., cannot be comprehensively assessed based on the available literature (Cagnin et al., 2009; Bylemans et al., 2018; Clare et al., 2021). Other types of templates derived from formalin-fixed and paraffin-embedded (FFPE) tissue (Guyard et al., 2017), museum samples, ancient and highly degraded material (*e.g*., ice cores, permafrost, fossil bones, faeces, *etc*.), or samples preserved for several years in ethanol will likely not be suited due to extensive DNA degradation (Merheb et al., 2019).

Our enrichment assay facilitates amplification with highest accuracy. Similar replication error rates have been reported for the current top-standard enzyme for long-range PCR, Q5^®^ High-Fidelity DNA polymerase, and the phi29 (Φ29) DNA polymerase used in isothermal multiple-displacement amplification (error rates of 5.3 × 10^−7^ (Potapov and Ong, 2017) and 1 × 10^−6^ to 10^−7^ nucleotides (www.qiagen.com), respectively). Our assay is resistant against partial or single-copy NUMTs dispersed in the nuclear genome since they generate no double-stranded amplification product (Balciuniene and Balciunas, 2019). Notably, in case of nuclear mtDNA concatemers (Mega-NUMTs) amplification seems to be likely (Balciuniene and Balciunas, 2019). However, the rareness of their occurrence renders this phenomenon rather negligible.

For a mitoepigenetic perspective, single long-range PCR can identify restriction enzymes cutting the novel mitogenome of interest at a single site. Linearization of the circular mitogenome would facilitate mtDNA enrichment from total cellular DNA based on the different electrophoretic mobilities of covalently closed circular mtDNA *versus* endonuclease-digested nuDNA (Wiesner et al., 1991), thus, providing a mtDNA template for measuring epigenetic marks such as 5-methylcytosine, 5-hydroxymethylcystosine, 5-formylcytosine and 5-carboxylcytosine (Devall et al., 2016).

Accumulation of genetic changes in response to coping with cold is a historical and current topic of scientific interest. Enrichment of mtDNA by single long-range PCR seems promising to decipher mtDNA variants related to cold adaptation. For example, after early humans had migrated to colder climates, their chance of survival increased when their mtDNA was mutated leading to mitochondrial uncoupling of oxidative phosphorylation and ATP synthesis and heat dissipation (Wijers et al., 2008; Wallace, 2015). For example, amino acid replacements in mitochondrial subunits of OXPHOS complexes I or V were related to cold adaptation in man (Wijers et al., 2008) or monkey (Wallace, 2015).

There are various options to further extend the range of vertebrates for which an experimental proof of applicability for our mtDNA-enrichment assay is demonstrated concomitantly focussing on mtDNA markers for cold adaptation and/or the alteration of the time point when animals enter or leave hibernation. In cape hares (*Lepus capensis*) collected along a steep ecological gradient, polymorphisms in mitochondrially encoded OXPHOS subunits are significantly associated with mean annual temperature (Slimen et al., 2017). Likewise, climatic variation and/or introgression were suggested to be at the origin of such adaptation among eight Chinese hare species occupying different habitats ranging from the cold north of the interior mainland to the tropic island of Hainan (Awadi et al., 2021). Analysing these cohorts of hares with our approach would allow targeting the entire mitogenome instead of just several candidate genes. A similar field of possible application for the approach are the many shrew species distributed from cold to warmer habitats of the United States. The ability of the cold-adapted, but not hibernating shrews to survive in cold climates is evidenced by Vermont having eight kinds of shrews and Alaska ten, compared to Alabama with six and Florida with only three (Gibbons, 2019). Furthermore, it could also be addressed if there are changes in the mtDNA of thirteen-lined ground squirrels (*Ictidomys tridecemlineatus*) that are related to the different latitudes and climatic zones of their Northern American habitats and/or that contribute to the genetic variation driving the seasonal onset of its hibernation (Grabek et al., 2019). Currently, we discuss two other putative applications of our mtDNA-enrichment assay. First, we asked whether polymorphisms in mtDNA-encoded OXPHOS subunits might contribute to different adaptability to climate change-induced duration of hibernation in bats and, second, we would like to issue the hypothesis whether specific mtDNA mutations are accumulated in Japanese macaques (*Macaca fuscata*) to adapt to cold climates (Kawamoto et al., 2007). Japanese macaques can be stratified into two main mitogroups that show strong correspondence with geographic distribution (Kawamoto et al., 2007). The two subspecies of this non-human primate inhabit geographically separated and climatically different regions with cold to moderate up to subtropical climates (Aimi and Fooden, 2005). Whereas Northern and central habitats are covered by *M. fuscata fuscata,* the macaque of the Japanese mainland, Yakushima Island—the southern limit of Japanese macaque’s distribution, hosts the indigenous subspecies *M. fuscata yakui*.

The prediction of MDPs represents another issue when annotating novel mitogenomes. It is complicated by the facts that mammalian mitochondria actively import tRNAs (Rubio et al., 2008; Duchêne et al., 2009; Schneider, 2011), and that mitochondrial mRNAs can be exported into the cytoplasm and translated according to the standard genetic code of eukaryotic nuclear genomes (Sreekumar et al., 2016; Kim et al., 2018). These options and their combination could have implication for predicting the amino acid sequences of gau and other MDPs – an issue that might be addressed in future experiments. Likewise, the highly conserved predictions of SHLP4 and 6 as well as of a four amino acid-core in MOTS-c ((Lee et al., 2015); this study), a highly regulative peptide encoded by *MT-RNR1* (Fuku et al., 2015; Lee et al., 2015, 2016; Kim et al., 2018; Benayoun and Lee, 2019; Lu et al., 2019), argue for research beyond the classical model species of human and mice. For humanin our extended range of target species confirmed the mosaic of absent and putatively functional sORFs reported earlier (Logan, 2017). Analogously, pseudogenization caused by a missing translational start, a premature stop codon and/or a frameshifting insertion/deletion mutation (Logan, 2017), might also explain why our five hibernating target species do not exhibit sORFs encoding SHLP1, 3 and 5.

The MDP’s role in bioenergetics and metabolism of mitochondria would implicate a connection to cold adaptation (Lu et al., 2019) and hibernation requiring cytoprotecting and oxidative stress reduction. Recently, the first experimental link between hibernation and MDPs was demonstrated for the Alzheimer’s survival peptide humanin (Szereszewski and Storey, 2019). Likewise, the functional similarities of other MDPs such as SHLP4 and 6 that play roles in proliferation and apoptosis (Cobb et al., 2016) might participate in reversing hibernation-associated mass reduction of tissues such as the gastrointestinal tract and the mucosa (Carey, 1990, 1992; Hume et al., 2002). Future research should also address whether mtDNA plays a role in the up to ∼20% skull shrinkage in the non-hibernating mole-like mammal *Sorex araneus*, the most common shrew species, that occurs before the hardships of winter and, come spring, returns to roughly normal size (Lázaro et al., 2017). Concluding, the relationship of some of the annotated MDPs to hibernation physiology could inspire and direct future studies.

## Conclusion

This study provided experimental evidence for the option of mitogenome enrichment by single long-range PCR amplification extended from humans and ray-finned fishes to five rodent hibernator species. Our assay used a set of universal back-to-back oligonucleotides designed against a consensus motif of the vertebrate subphylum (bony fishes, amphibians, reptiles, birds, and mammals), and the size range reported for mitogenomes of vertebrate species (14–20 kb (Kolesnikov and Gerasimov, 2012)) that is well compatible with single long-range PCR, argue for the assay’s potential for application across an extended set of vertebrates beyond the hibernating rodent species analysed in this work.

We report the degree of nucleotide-sequence dissimilarity at the level of the complete mitogenome for two *M. avellanarius* individuals derived from Belgium and Denmark (9.9%). These locations match the genetically distinct lineages of *M. avellanarius* regarded to be a cryptic species (at least two species hidden under a single name). Future research on social communication, reproduction mechanisms or morphometrical differentiation, *etc.* may help gaining insight into possible adaptive differentiation across Europe and to delimit the species of the cryptic *M. avellanarius* species complex (Mouton et al., 2017) that is in line with our data.

The “mitochondrial Russian doll genes”–sORFs located in mitochondrial rRNA or protein-coding genes, represent promising targets of future *in-silico* analyses and experimental studies given the functional importance of the encoded micropeptides in retrograde signalling (Kim et al., 2018), cellular metabolism (Merry et al., 2020; Miller et al., 2020; Breton, 2021), but also hibernation (Szereszewski and Storey, 2019). A special focus on cold adaption and the various torpor-related phenotypes might be of interest.

## Data Availability

The datasets presented in this study can be found in online repositories. The names of the repositories and accession number(s) can be found below: NCBI’s Nucleotide Database (https://www.ncbi.nlm.nih.gov/nucleotide/), accession numbers MN935776 to MN935780 and MF405145.2; NCBI’s Sequence Read Archive (https://www.ncbi.nlm.nih.gov/sra), accession number PRJNA748678.
