# Corrigendum: Extension of mitogenome enrichment based on single long-range PCR: mtDNAs and putative mitochondrial-derived peptides of five rodent hibernators

**DOI:** 10.3389/fgene.2024.1386502

**Published:** 2024-03-12

**Authors:** Sarah V. Emser, Helmut Schaschl, Eva Millesi, Ralf Steinborn

**Affiliations:** ^1^ Genomics Core Facility, VetCore, University of Veterinary Medicine, Vienna, Austria; ^2^ Department of Behavioral and Cognitive Biology, University of Vienna, Vienna, Austria; ^3^ Department of Evolutionary Anthropology, University of Vienna, Vienna, Austria

**Keywords:** mitogenome, long-range PCR, back-to-back amplification primers, next generation sequencing (NGS), mitochondrial-derived peptides (MDPs)

In the published article, there was an error. We incorrectly documented the experimental detection of MOTS-c through mass spectrometry and furnished a reference to substantiate this assertion. Nonetheless, despite the endeavors outlined in the cited study, endogenous MOTS-c still remains undetected by this “gold-standard” technology. The second reference is incorrect and should be removed.

A correction has been made to **Introduction**, paragraph 2. The sentence previously stated:

“In case of circulating cell-free MOTS-c, mass spectrophotometry delivered gold-standard proof for MDP expression (**Knoop et al., 2019; Reynolds et al., 2021**).”

The corrected sentence appears below:

“In case of human MOTS-c, the “gold-standard” technology of liquid chromatography-mass spectrometry successfully detected the synthetic peptide used as positive assay control. However, the test failed to detect the circulating cell-free form of MOTS-c in human plasma samples irrespectively of the success in ELISA-based measurement (**Knoop et al., 2019**).”

The authors apologies for this error and state that this does not change the scientific conclusions of the article in any way. The original article has been updated.

