# Supplementary material for: Extension of Mitogenome Enrichment Based on Single Long-Range PCR: mtDNAs and Putative Mitochondrial-Derived Peptides of Five Rodent Hibernators
Source: Front Genet. 2021 Dec 13;12:685806. doi: 10.3389/fgene.2021.685806 (PMC8749263; doi:10.3389/fgene.2021.685806)
Supplement: Supplementary file 1 [file DataSheet1.zip › Figure S1.docx]

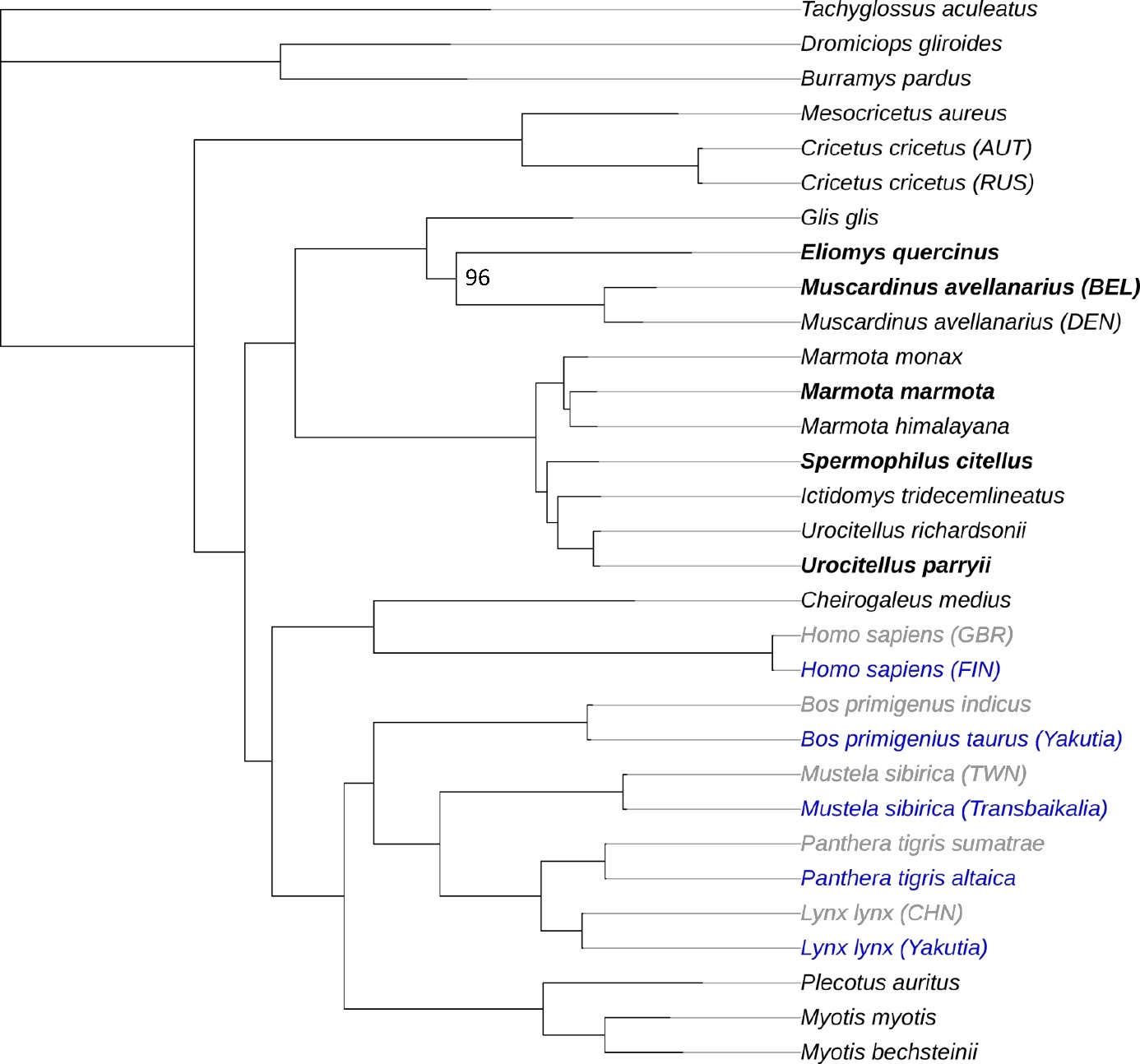


**Supplementary Figure S1.** Analysis of Bayesian phylogenetic inference using MrBayes version 3.2.7a ((Ronquist et al., 2012); <http://nbisweden.github.io/MrBayes/)>. Two simultaneous runs of 10 million generations were conducted for the datasets, and trees were sampled every 1,000 generations, with the first 25 % discarded as burn-in. The only Bayesian support value below 100 % was depicted. Black: hibernators, blue: cold-adapted individual; grey: not cold-adapted and not hibernating phenotype.

References

Ronquist *et al*. (2012) *Syst Biol* 61: 539-542
