# Supplementary material for: Extension of Mitogenome Enrichment Based on Single Long-Range PCR: mtDNAs and Putative Mitochondrial-Derived Peptides of Five Rodent Hibernators
Source: Front Genet. 2021 Dec 13;12:685806. doi: 10.3389/fgene.2021.685806 (PMC8749263; doi:10.3389/fgene.2021.685806)
Supplement: Supplementary file 1 [file DataSheet1.zip › Figure S2.docx]

|  | MOTs-c | SHLP2 |
| --- | --- | --- |
| *Homo sapiens* | MRWQEMGYIF YPRKLR* | MGVKFFTLST RFFPSVQRAV PLWTNS* |
| *Marmota marmota* | ..... .LPRIDPTIA FMK..A* | .E.GS...* |
| *Spermophilus citellus* | ..... ..PRTNSYDS FYETQSIRRI * | .E.NRV..* |
| *Urocitellus parryii* | ..... .FSRINIHDS FHET* | .E.NRV..* |
| *Eliomys quercinus* | .NRE.....Y FIS.YANSRE IREQKADLVV S* | - |
| *Muscardinus avellanarius* | .K.E.....Y FTKYIHVNSY EI* | - |
| *Vulpes lagopus* | ...E...... CFKNTLRKFL * | .V.LV...* |
| *Ursus maritimus* | .E.E...... .S.TTYESFY ETKN* | .I* |
| *Ovis nivicula* | .E.E...... ..K.NLIRKP L* | .VFLDL..* |
| *Balaenoptera acutorostrata* | .S.K...... .TKNISYTHT KVFMKFKNQR RI* | .VYV* |
| *Mammuthus primigenius* | ..... .Y.IDKRIPL * | .KLMVNL.AN QGCILG.KSC TPFEYL* |
|  |  |  |
|  | SHLP4 | SHLP6 |
| *Homo sapiens* | MLEVMFLVNR RGKICRVPFT FFNLSL* | MLDQDIPMVQ PLLKVRLFND * |
| *Marmota marmota* | .......... ..* | ......Q..* |
| *Spermophilus citellus* | .......... ..* | ......L..* |
| *Urocitellus parryii* | .......... ..* | ......Q..* |
| *Eliomys quercinus* | .......... ..LYLPSS.Y ..LSF.IALL CWVNN* | ......L... Q..R...... * |
| *Muscardinus avellanarius* | .......... ..* | ......L... .......... * |
| *Vulpes lagopus* | .......... ..LCLPSS.Y ..* | ......L... Q..R...... * |
| *Ursus maritimus* | .......... ..LCLPSS.Y ..* | ......L... Q..R...... * |
| *Ovis nivicula* | .......... ..LCLPSS.Y ..* | .....T.... ..S....... * |
| *Balaenoptera acutorostrata* | .......... ..LCLPSS.Y ..* | ......L... ...R...... * |
| *Mammuthus primigenius* | .......... ..LCLPSS.S ..* | ......L... ...R...... * |
|  |  |  |
|  | Humanin |  |
| *Homo sapiens* | MAPRGFSCLL LLTSEIDLPV KRRA* |  |
| *Marmota marmota* | ..* |  |
| *Spermophilus citellus* | ..* |  |
| *Urocitellus parryii* | ..* |  |
| *Eliomys quercinus* | ..* |  |
| *Muscardinus avellanarius* | ..* |  |
| *Vulpes lagopus* | ..T...N... .PIR...... ...EYHNKTR RPYGALIN* |  |
| *Ursus maritimus* | - |  |
| *Ovis nivicula* | ..T...Y... .PI....... ...E* |  |
| *Balaenoptera acutorostrata* | ..T...Y... ..I....... ...G* |  |
| *Mammuthus primigenius* | - |  |

**Supplementary Figure S2**. A comparison of MDP-containing sORFs between *Homo sapiens* and the five rodent hibernator model species as well as the five cold-adapted species Arctic fox, snow sheep, Mammoth, Polar bear, and Minke whale (in blue). For SHLP1, 3 and 5 no sORF was found across these target species. Asterisk: end of sORF; dot: identity with human sequence; hyphen: failure to annotate a sORF. In brown: species subjected to the extended single long-range PCR assay developed within this study. In blue: cold-adapted species/populations.
