# Supplementary material for: Extension of Mitogenome Enrichment Based on Single Long-Range PCR: mtDNAs and Putative Mitochondrial-Derived Peptides of Five Rodent Hibernators
Source: Front Genet. 2021 Dec 13;12:685806. doi: 10.3389/fgene.2021.685806 (PMC8749263; doi:10.3389/fgene.2021.685806)
Supplement: Supplementary file 1 [file DataSheet1.zip › Figure S3.docx]

|  | SHLP4 | SHLP6 |
| --- | --- | --- |
| *Homo sapiens* | MLEVMFLVNR RGKICRVPFT FFNLSL* | MLDQDIPMVQ PLLKVRLFND * |
| *Cavia porcellus* | MLEVMFLVNR RDLCLPSSFC FVLSFLSSTP VSG | ......L... Q..R...... * |
|  |  |  |
| *Cygnus olor* | .....S .ALWFAEFLS QISIVLMGAP ESG* | ......L.. ...R...... * |
| *Phylloscopus fuscatus* | .....S .ALT.LPSSF YRF* | ......LV.. ...R...... * |
|  |  |  |
| *Triturus cristatus* | ...... .NSWLPSS.L IF* | .....TQ... .......... * |
| *Xenopus laevis* | ...... ..S* | ....G..V.. .......... * |
|  |  |  |
| *Danio rerio* | ...... ..LCLPSS.P ..* | ......L... ...R...... * |
| *Nothobranchius furzeri* | MGQAGLLILR IQ.A...... ..F.VYL.NS ..FFLSFL* | ......L... Q..R...... * |
|  |  |  |
| *Boa constrictor* | .....S WSHWLPSS.Y NFLSFFLALL CRVYSFLISC ELGCFIPLMV C* | .....TQ..* |
| *Crocodylus acutus* | - | ......L..* |
|  |  |  |

**Supplementary Figure S3.** The conservation of SHLP4 and 6 across vertebrate species. Asterisk: end of sORF; dot: identity with human sequence; hyphen: failure to annotate a sORF.
