# Supplementary material for: Extension of Mitogenome Enrichment Based on Single Long-Range PCR: mtDNAs and Putative Mitochondrial-Derived Peptides of Five Rodent Hibernators
Source: Front Genet. 2021 Dec 13;12:685806. doi: 10.3389/fgene.2021.685806 (PMC8749263; doi:10.3389/fgene.2021.685806)
Supplement: Supplementary file 1 [file DataSheet1.zip › Figure S4.docx]

|  | Vertebrate mitochondrial code | | | |  |  |  |  |  |  |
| --- | --- | --- | --- | --- | --- | --- | --- | --- | --- | --- |
| *Homo sapiens* | MGSPPPAGSK | KVVL* |  |  |  |  |  |  |  |  |
| *Eliomys quercinus* | .......... | ..... |  |  |  |  |  |  |  |  |
| *Muscardinus avellanarius* | .......... | ...F. |  |  |  |  |  |  |  |  |
| *Marmota marmota* | .......... | N.... |  |  |  |  |  |  |  |  |
| *Spermophilus citellus* | .......... | N.... |  |  |  |  |  |  |  |  |
| *Spermophilus parryii* | .......... | N.... |  |  |  |  |  |  |  |  |
|  |  |  |  |  |  |  |  |  |  |  |
|  | Nuclear genetic code | | | |  |  |  |  |  |  |
| *Homo sapiens* | MGSPPPAGSK | KVVLRLRSVS | SIVMPAARTG | RDRRSRTAVI | RTDQTKRGVW | YWVMAGGFIL | IIVVMKLMAP | KIEETPARCK | EKMVRSTEAP | GWE* |
| *Eliomys quercinus* | I......... | ...F...... | .......N.. | SE.S.K.... | .....NK..* | .CDI....M. | ....I..I.. | ..D....K*S | ..I....... | ACAKL..N.. |
| *Muscardinus avellanarius* | I......... | .....F...R | .......N.. | ..NN.K.... | ...H.N...* | .CD....... | M...I..I.. | R.DD....*S | ..I....... | ACAK...N.. |
| *Marmota marmota* | I......... | N......... | ...I...... | ...SNS.... | N..H.NK..* | .*DI....M. | ....I..I.. | R.D....K*R | .......... | A.AR...N.. |
| *Spermophilus citellus* | I......... | N....F.... | ...I...... | ...SN..... | N..H.N...* | .*D.....M. | ....I..I.. | R.DD...K*R | .......... | A.AR...R.. |
| *Spermophilus parryii* | I......... | N........R | ...I...... | ...SNS...T | S..H.N...* | .*D.....M. | ....I..I.. | R.DD...K*R | ..I....... | A.AR...S.. |

**Supplementary Figure S4.** Predicted amino acid sequences for the gau peptide. In all analysed species, translation with the vertebrate mitochondrial code predicted a 14 amino-acid sORF encoding the gau peptide. In contrast, translation with the standard (nuclear) genetic code did no yield a start codon at the position that corresponded to the translational start of the human gau peptide. Note that the possibility of a later or earlier start/end cannot be ruled out according to Faure et al., 2011. Asterisk: end of sORF; dot: identity with human sequence.

Reference

Faure E, *et al.* (2011) *Biol Direct* 6: 56
