# Supplementary material for: Extension of Mitogenome Enrichment Based on Single Long-Range PCR: mtDNAs and Putative Mitochondrial-Derived Peptides of Five Rodent Hibernators
Source: Front Genet. 2021 Dec 13;12:685806. doi: 10.3389/fgene.2021.685806 (PMC8749263; doi:10.3389/fgene.2021.685806)
Supplement: Supplementary file 1 [file DataSheet1.zip › Supplementary File1.docx]

>Tachyglossus_aculeatus Tachyglossus aculeatus mitochondrion, complete genome.

G-----GTAGTTTA------TAATGCTAAAACAAAACACTGAAAATGTTT

AGATGATTTTTAACTAAATCC--TAGTGCACAAAGGTTTGGTCCTAGCCT

TATTGTTAGATTTGACTAAATTTATACATGCAAGTATCCGCAGACCAGTG

AGAATACCCTAAAAACTTTAAGCA-----AGTTAAAAGGAGTTGATATCA

GGCACACT-----AACGTAGCCCACAACATCTTGCCTTAGCCACACCCCC

ACGGGACACAGCAGTAATAAAAATT-GGTCAATAAACGTAAGTTTGAACA

AGTTATAGTC---AACAAGAGTCGGTAAATTTCGTGCCAGCCACCGCGGT

TATACGATTGACTCAACCTAACAAATA-AC-GGTGTAAAGCGTGTTTAAA

A--------CTTTAATCAATAAGATTCAAGCAGGACTAATCCGTGACAAG

TCCTAGTCCATG-CTAAAATCACCCACGAAAGTGATCTTAT---AATTCT

TGAATACACGATAGCTAAGACACAAACTGGGATTAGATACCCCACTATGC

TTAGCCCTAAACTTAAGTCGTTAAAC--AACAAAACCACTCACCAGAGAA

CTACTAGCAACAGCTTAAAACTCAAAGGACTTGGCGGTGCTT--CACCCC

TCTAGAGGAGCCTGTTCTATAATCGATAAACCCCGATACACCCCACCATC

TTTTGCCACTA-CTGTCTATATACCGCCATCGTCAGCCAACCCTAA-AAA

GGAATAACAGTAGGCACAATTATTCTT--CATAAAAACGTTA-GGTCAAG

GTGTAGCCTATAAGATGG--AAGAAATGGGCTACATTTTCTAACCTAGAA

TA---------------TACGAAAAACCCTATGAAACTAGA---GTCCCA

AGGAGGATTTAGCAGTAAGTT-GAGAATAGAGAGCTTAACTGAACCG-GG

CAATGAAGCACGCACACACCGCCCGTCACCCTCCTCAACCAGT-------

-AACACCCCAATCCCTAATACAAAATACTAAACA----------AGAGGA

GATAAGTCGTAACAAGGTAAGCATACCGGAAGGTGTGCTTGGAATA-TCA

AAATGTAGCTTA----ACCCAAAGCACTCAGCTTACACCTGAAAGATATT

--TATCACAAAATCATTTTGAGCCTTAACTCTTAGCCCAAATC-------

AACACAATCAACCTATGTATTTCAACAAACTAAAACATTTTAACATCAAC

CCCTA--GTATTTGAGACAGAAA----GGAATAACCGGAGCTTTAGATAA

AGTACTGTGAAGGAACAAATGAAAGATT--TCCCCAAGCACGAAAAAGCC

AAGTTTAAATCTTGTACCTTTTGCATAAATGGTTTAGCTAGAAAAT-CTA

TACACAAAG-ATTTGTAATATAAAACCCCGAAACTAAATGAGCTACTATA

GGACAATTT--ATCAGAATGCACCCGTCTATGTCGCAAAATAGTGGGACG

ATTTTATAGTAGAGGTGAAAAATCAACCGGATTTAGTGATAGCTGGTTAA

CCAAGAAATGAATTTAAGTTCAACAGTAAGTTTATTTCCCTAGCACCCT-

-ATATGACTCATAAACTTACTAGCTACTTATAAGAGGGTCAGCCCT-TAT

AAGTAAGGAAACAACCTCCAATAGAGGGAAA-------CTCTTACCACGT

ACATAGTAGGCTTAAAAGCAGACACCTATTAAGAAAGCGTTAAAGCTCAA

AC-----------CCAACCCTTTTCTCTAATTCCACAATATCTCAACTAC

CCCTAAATCAATATTGGTTTATTCTATATCTTTAT-AGAAGAAATAATGC

TAAAATAAGTAACCAGAA---TTTATTCTCCTTGCACTAGCTTAAGTTAG

AACGGAACAA-CCACTAACAATTAACAGTTAAATAAT----TAAAACTAT

AAACAAGAGCCATTATTTTTCTTGACTGTTAACCCGACACAGGCGTGCA-

--CTAAAGGAAAGATAAAAAAGAGTAAAAGGAACTCGGCAAATTAGGATT

TCGCCTGTTTACCAAAAACATCGCCTCTAGCATAACAAGTATTAGAGGTC

CTGCCTGCCCAGTGATATTA------TTAAACGGCCGCGGTATCCTGACC

GTGCAAAGGTAGCATAATCACTTGTCTCCTAATTAGAGACTAGCATGAAC

GGCTAAACGAAAATCCAACTGTCTCTTACTCTCAATCAGTGAAATTGCCC

TCCCCGTGCAGAGACGGGGATAAGAACATAAGACGAGAAGACCCTGTGGA

GCTTTAATTAAAGAG---TAACTTCTTCGAACATTCAACTTAAGGGTGTA

GCATCTAAAAATTTTACTCAACTAG--------TTTCGGTTGGGGTGACC

TCGGAGAACAGTTAAACCTCCGAATGA-----ACAGTAAAGACCTACAAG

TCTAAAC-GCAATACTGCCAGTAATAGACCCATA----TTATTGATCAAA

GGACCAAGTTACCCCAGGGATAACAGCGCAATCCCATTCTAGAGTTCATA

TCGACAAT-GGGGTTTACGACCTCGATGTTGGATCAGGACATCCAAATGG

TGCAGCAGCTATTAATG-GTTCGTTTGTTCAACGATTAA-AGTCCTACGT

GATCTGAGTTCAGACCGGAGCAATCCAGGTCGGTTTCTATCTAT--GAGT

TATTTCTCCCAGTACGAAAGGACCAGAGAAATCAGGCCAATCTCACAAAG

AA-GCCTTCTATCTAACAAATGATATCA-TCTTAATTTGTTCACACCACT

CTCTA-----CAATCCTAGA-CAAGGAGCCCGATTAAGGTGACAGAGACC

GGCAA-TTGTGTAAAACTTAAGCTTTTATAATCAGAGGTTCAAATCCTCT

CCTTAATACCACTCCCGCCCACAGGGCTAAACTTATCAACGGTTAACAAT

AGATCACTCACTCTGAACTAAGTATAGCAACTTTCCTTAATAATATTTTT

AATTAATCTATTACTACTTATTGTCCCCGTTCTATTAGCAGTAGCCTTTC

TAACCTTAATTGAACGCAAGATCTTAGGTTACATACAATTTCGGAAAGGA

CCAAACATCGTCGGACCCCATGGACTCCTCCAACCCATTGCAGACGCAGT

TAAACTATTTATCAAAGAACCCCTACGCCCATTAACATCTTCAATCTACA

TGTTCATCCTCGCCCCAATCTTAGCCCTATCCCTAGCCCTAACTATTTGA

GTACCACTTCCCATACCCCTCCCCCTTATTGACCTAAACCTAGGCCTCTT

ATTCATTCTCTCAGTATCAGGGCTATCTGTCTACTCCATCCTCTGATCAG

GGTGAGCCTCAAATTCCAAATACGCACTAACTGGGGCCCTACGGGCCGTA

GCCCAAACCATTTCCTACGAAGTAACCTTCGCGATCATCCTCCTCTCGAT

CATATTAATCAATGGTTCCTTTACCCTAACCACCCTAAATCTAACCCAGG

AGTTCATATGATTAGTTGTACCAACTTGACCCCTAATACTAATATGATTT

ATTTCAACCCTAGCTGAAACCAATCGCGCACCATTTGATTTAACAGAAGG

GGAATCTGAATTGGTATCCGGCTTTAACGTAGAATACGCAGCAGGTCCTT

TCGCCATATTTTTCTTAGCCGAATATGCTAACATTATCATTATAAATGCC

CTCACAGTAATCCTATTTTTTGGCACCTACCACCTCATCTTTTTACCCGA

GCTATCCACTACCAACTTTATAGTCAAAACCATACTACTAACCTCCCTAT

TTTTATGAGTCCGAGCATCCTACCCACGTTTTCGCTACGACCAGTTGATA

CACCTACTATGAAAAAACTTTCTACCCATTACACTTGTTACATGCCTCTG

ATATATTATATTTCCTACTATGCTGTCAGGAACCCCTCCACAAATATAAA

GAAATATGTCTGATAAAAGAGTTACATTGATAGCGTAAATAATAGAGGTT

-AAAACCCTCTTATTTCTAGAATAATAGGACTTAAACCTACATCTAAGGC

TTCAAAAACCTCCGTGCTCTCAAT--TACACCATACTCTA----------

GTAAGGTCAGCTAAAT-AAGCTATCGGGCCCATACCCCGAAAATGTTGGT

TTATACCCTTCCCATACTAATTACACCTATAACTAACTTAATCATAATAT

CTAGCCTACTCATAGGAACAATAATTACCCTAACAAGCTCCCACTGATTA

CTAATATGAATAGGTCTCGAAATTAACACCCTGGCTATCATTCCTCTTCT

TACTAGTAAGAAACACCCTCGATCCACTGAATCAGCAATTAAGTACTTTT

TAACGCAAGCAACAGCCTCCATACTTCTTATATTCGCTGCATCCCTCAAT

ACCTGACTAACAGGACACTGAACCTTAATACAAATCAACAATATAGTACC

CTCCATAATTATGACATTCGCATTAGCAATGAAACTAGGCCTGGCACCCT

TCCACTACTGAGTACCTGAGGTCCTCCAAGGATCACCCCTGTTATCAGGG

ATAATCCTACTAACCTGACAAAAACTTGCACCAATTTCAATTATCTACCA

AATTTCCCCAACCCTTAACATAAACACCCTATTAATCTTAGCAATCTCAT

CTATCCTATTAGGAGGATGAAACGGCCTGAACCAAACCCAATTACGCAAA

ATCATAGCCTACTCATCAATTGCCCACATAGGATGAATAATCGTAATTAT

CATTTATTTCCCCCTACTCACCATCCTTAATCTAGTACTCTATATTATAT

CAACAGTAGCCTTATTTATAGTATTTTATCACATTAATATTACGAAAACC

AAACCCCTCTCCCTCATATGAAATAAATCACCAACCCTCATGTTAACAAC

CATCCTCGTCTTGCTATCCCTCGGAGGTCTCCCCCCACTCACTGGATTTG

CTCCCAAATGACTAGTCGTACAAGAACTAATTATACACAATAATATTATA

ATAGCAACTACCCTGGCAATCATAGCCCTCCTAAACTTATTTTTCTACAT

ACGAATTATTTACTCATCAACACTAACAACATTCCCAACGACTAATAACA

ACAAATACCACTGATATAAGCAACCCATAAAAACCCCCTTATCCCTCCCC

CCTCTAACCATTCTCTCCACCGCATTACTACCTCTTACACCCATGTTTAT

TACATT---GGGCT----AAAGGTTTAGGTTAAT----TAGACCAAGAGC

CTTCAAAGCTCTAAGTAGGTATCTC--TCATGCCTAACCTTTGC------

-------TTCGAAAACTGGAAGTCTCTCTTCCAT------CTTTTGACTG

CAAATCAACTACTTT---CCTTAAGCTAAATTCTC------------CTA

GACTAGTA---GGCCTTGATCCTAC-AAAATTTTAGTTAACAGCTAAACA

CTTTAACCAGCAAGTCTTAATCTACT--TTTCCCGCCTTAAGAAAGGGA-

-----GGCGGGAAAAGCCCCGGCA-----CTCTGAGTTGCTTTTCC----

---------------------------GAATTTGCAATTCGACG-----T

GATTCCACTGCAGGGCTT------GGTAACAGGGGA-------TTGCTCC

CCGTCTTTAGATTTACAGTCTAATGCCTA--CTCAGCCATATTACC----

-------TATGTTCATTAATCGCTGACTATTTTCAACTAACCATAAAGAT

ATTGGTACCCTCTATCTTCTATTCGGTGCATGAGCTGGCATAGCCGGCAC

AGCCCTCAGTATTCTCATTCGATCCGAATTAGGCCAACCAGGCTCCCTCT

TAGGTGATGATCAAATTTATAACGTTATCGTCACAGCCCATGCATTTGTT

ATGATTTTTTTCATAGTTATGCCAATCATAATCGGAGGTTTTGGTAACTG

ATTGGTCCCCCTAATGATTGGGGCTCCAGATATAGCATTCCCACGAATAA

ACAATATGAGTTTCTGGCTTTTACCCCCTTCATTTCTCCTACTCCTAGTT

TCCTCCACAGTAGAAGCAGGCGCAGGAACTGGCTGAACCGTCTATCCACC

CCTAGCAGGCAACCTAGCCCATGCTGGAGCCTCAGTAGACCTGGCTATTT

TTTCCCTTCACCTAGCTGGAGTTTCCTCTATCCTAGGGGCTATTAACTTT

ATTACCACAATCATTAACATGAAACCTCCTGCAATATCCCAATATCAAAC

ACCCCTGTTCGTCTGATCAGTACTAGTTACAGCTGTCCTTCTCCTTTTAT

CACTCCCCGTCCTTGCGGCAGGCATTACCATACTTCTCACTGACCGAAAT

CTTAATACAACTTTCTTTGACCCAGCAGGGGGTGGAGATCCTATTTTATA

TCAACACCTGTTCTGATTTTTTGGACACCCTGAAGTCTATATCTTAATCT

TACCAGGCTTTGGAATTATCTCTCATATTGTTACTTACTACTCAGGAAAA

AAAGAACCATTCGGGTATATAGGAATAGTTTGAGCTATGATATCCATCGG

ATTTTTAGGTTTCATCGTATGGGCTCACCACATATTTACAGTTGGCATAG

ACGTAGATACGCGAGCCTACTTCACATCCGCTACAATAATTATTGCTATT

CCCACTGGCGTTAAAGTTTTTAGCTGGCTTGCCACACTTCACGGTGGTGA

TATCAAGTGAACTCCCCCTATACTATGAGCTCTCGGCTTTATTTTCCTTT

TTACCGTAGGAGGCCTAACGGGTATTGTTTTAGCAAACTCATCATTAGAT

ATTATTCTTCACGATACATACTACGTAGTAGCCCACTTTCATTACGTCTT

ATCCATGGGAGCTGTATTTGCTATCATAGGAGGCTTTGTCCACTGATTCC

CTCTTCTATCAGGCTTTACACTCCATACAACATGGGCCAAAGTCCACTTT

ACCCTGATATTTGTCGGAGTTAATTTAACCTTTTTCCCACAACATTTTCT

AGGTTTAGCAGGTATACCACGTCGTTACTCAGATTACCCAGACGCCTACA

CCCTATGAAACGCTATCTCATCTCTTGGATCTTTTATTTCACTAACAGCT

GTCATAGTAATAATTTTTATGGTTTGAGAGGCCTTTGCATCCAAACGTGA

AGTCCTAACTGTAGAACTAACTTCAACCAACATTGAGTGACTCCACGGAT

GTCCACCGCCTTACCACACCTTTGAAGAACCGGTATACATTAAAATTTAA

-----------------TCAAGAAAGGAAGGAATTGAACCTCCTAATATT

GGTTTCAAGCCAATCTCACTACCATTATGATTCTTTC------TTTATGA

AGTGTTAGTTAA-CAGATAACATAGCCCTGTCATAGCTAAGTCACAAATC

-------TAATTTTGTACACTTTA-ATGGCCTACCCCCTCCAACTAGGAT

TTCAAGATGCAACCTCACCCATTATAGAGGAACTCTTACACTTCCATGAC

CACACCCTAATAATTGTCTTCCTTATCAGCTCCCTAGTTCTTTATGTCAT

CTCAACAATGCTCACTACAAAACTAACTCACACAAACACTATAGACGCTC

AAGAGGTGGAAACTATCTGGACTATTCTACCAGCCATCATCTTGATCCTC

ATTGCCTTACCCTCATTACGTATTCTATACATAATGGATGAAATTAATAA

CCCAAATTTAACCATCAAAACAATGGGCCACCAATGATACTGAAGCTATG

AATACAGTGATTATGAGGACCTATCATTTGACTCCTACATAATTCCTACC

CAAGACCTTACTACAGGGCAAATACGTTTATTAGAAGTAGACAATCATTT

AGTAATACCCATTGAGCTACCAATCCGCATGCTAATTTCATCTGAAGATG

TTTTACACTCATGAGCCCTTCCATCCATGGGCTTAAAGACAGACGCCATC

CCAGGCCGACTCAATCAAGCCACCATTACATCAACACGTCCAGGTTTATT

TTATGGTCAGTGCTCAGAAATTTGTGGTTCAAACCATAGTTTTATACCTA

TTGTACTCGAAATGGTCCCATTAAAACACTTCGAGAACTGATCCTCTTCA

ATGATGTCA--------------------------------ACCTCATAA

AGAAGCTA--TAGTAGCAGTAACCTTTTAAGTTAAAGACCGAGA----TA

TCCT-TTCTCCTTTATGATATGCCCCAATTAGACACATCTACCTGATTTA

TAGCAATTCTTCTTA-TAGTTTTCACCATCTATGGCATCTTCCAACTAAA

AGTAGTTAAATACCTCCCCACGGTCCTACCTACACCTTCTACCCAGGCAA

TGCCTGTCACGAATCTAACCCCTTGAAACTCCAAATGAACGAAAATCTAT

TTGCCTCATTCATCACTCCTACAATCCTAGGTATTTCAATTCTCCCACTT

ATCATAATCTTCCCATGTCTTTTATTTTCAGCCCCCAACCGTTGGATACC

TAATCGTCTAGTTGCCCTTCAACTCTGATTAGTTCGCATAGTTACT-AAG

CAAATGATGTCAATACATAATAAACAAGGTCGAATATGAACACTAATACT

AATCACCCTAATCATATTTATTGCTTCAACAAACCTTCTTGGATTACTAC

CCTACACATTCACCCCTACAACCCAACTATCAATAAACATGGGAATGGCA

GTCCCCCTATGACTAGGCACAGTCCTTATGGGATTTCGCAATAAAC-CTA

AATCCTCACTAGCCCATTTCTTACCCCAAGGAACCCCTACCCCCCTGATT

CCTATATTAATTATTATCGAAACTATCAGTTTATTTATTCAACCAGTAGC

ACTCGCAGTACGGCTTACTGCTAACATTACAGCAGGACATCTCTTAATCC

ACCTCATCGGATCAGCTACACTAGCCCTATCATCCATCAGTCTTACAGTA

TCAACAATCACGTTCACCATTCTTTTCCTCCTCACAATTCTAGAAATCGC

TGTAGCTCTAATCCAGGCGTATGTTTTCACACTACTAGTTAGTCTTTATC

TACATGATAACACCTAATGACCCACCAAACCCACGCCTATCACATAGTTA

ACCCCAGTCCATGGCCCTTAACAGGAGCTTTATCTGCCCTATTATTAACA

TCTGGCCTAATGATGTGATTTCACTTTAATAACCCCACCCTTCTTGTATT

AGGCCTACTTACCAATTTAATCTCATCCTATCAGTGATGACGAGATATTG

TCCGAGAGGGTACCTACCAAGGCCATCACACCAAAGTTGTCCAAAAAGGT

CTGCGCTACGGAATGGTCTTATTCATTATCTCGGAAGTTTTCTTTTTTTT

AGGCTTTTTCTGAGCCTTTTACCACTCCAGCCTAGCTCCAACTCCAGAAC

TCGGGGGGTGTTGACCCCCTACGGGCATTTCACCCTTAAACCCACTCGAA

GTTCCTTTATTAAACACCTCGATCCTTCTGGCCTCAGGGGTATCCATCAC

ATGATCTCATCATAGCCTAATGGAAGGCAACCGTAAACAAATGATTCAAG

CTCTAATAATTACTATTGCCCTAGGCTTATATTTTACTGCCCTACAAGCC

ATAGAGTACTACGAATCTTCCTTCACCATCTCAGACGGAGTATACGGCTC

TACTTTCTTTGTAGCAACAGGCTTTCATGGCCTCCATGTTATCATTGGTA

CCACTTTCCTAATTACCTGTCTTCTACGCCAACTTCTTTACCACTTTACA

TCAAACCACCATTTCGGCTTTGAAGCCGCAGCCTGATACTGACACTTTGT

AGATGTTGTATGACTATTCCTGTATGTCTCAATTTATTGATGAGGCTCAT

ATTTT-TCTAGTATTAA-TTAGTACAAGTGACTTCCAATCACTAAGTTTT

GGT-CTAATCCAAAGAAAAGTAATTAACCTATTAATTTCACTTCTAATCA

ACACTTGTCTAGCCACCATTTTAGTAGTAGTTGCATTCTGACTCCCCCAA

CTCTACACCTACTTAGAAAAGTCAAGTCCCTACGAATGTGGCTTTGACCC

TCTTGGATCGGCTCGACTTCCTTTCTCTCTAAAATTTTTCTTAGTAGCTA

TCACATTTCTTCTCTTCGACCTAGAAATTGCAATTCTCCTCCCACTCCCA

TGAGCATCTCAAACATCCTCTCCTTACTTACTCCTTGGCTTATCAGGAGT

CTTATTAACTTTACTAACACTAGGGTTAGCATACGAGTGACTCCAAAAAG

GCTTGGAGTGGACAGAA--------AGGTGATTAGTCTAA--TGAAGACC

ATTGATTTCGGCTCAATTAACCCTGGTATTAACCCATGATCACCTA---A

TAACAACAATATTTTTTAATTTACTATTGGCGTTTATAGTAGCTTTAATA

GGGGTCTACATTTACCGAGAACACCTTATATCCACCCTACTATGTTTAGA

AGGCATAATACTCTCCATCTTCATTATAGTATCACTCATCCTTCTTCACC

ACCACCTAAACTCAACTATAATATTCCCCCTTATTTTACTAGTTTTTTCT

GCATGTGAAGCAGGGGTGGGACTCGCACTCTTAGTTAAAACCTCCAACTC

ATATGGAACAGACTACATTGACAACCTTAACCTGCTTCAATGTTAAAAAT

CTTACTACCAACTGTAATACTCCTACCCCTTATTTCCTTCTCGAAAAAAG

AATGAATGTGAATCAACTCCTCAGCCTATAGTATTCTGATTAGCTCACTT

AGCCTCCTTACCCTTAACCAACACATAGACTTAGGCCTCAATTTCAACTT

AAACTTTTTCACAGATCCTCTATCCTCCCCCCTATTGGTACTCTCATGCT

GACTACTTCCACTCATGATTCTCGCAAGTCAATTCCACCTAATGAACGAA

TCAACCACCCATAAACGAATATACCTAATTTTACTAGTCTCTCTCCAAGT

TGCTCTCCTTATAGCTTTTAGCGCAGTAGAGTTTATAATGTATTATATCC

TATTCGAAACCACTCTAATCCCCACCCTAATTATTATCGCACGATGAGGG

AATCAAACGGAACGCTTAAATGCAGGTTTATACTTCCTATTTTATACCCT

ATTAGGCTCCCTACCCCTTCTAGTAGCCCTGATCTTTACGCAAGCACAAA

TAGGCTCACTTCATATCCTCCTATTAACGCTTACACCCAACCCCCTACTA

AACTCT----------TGATCCAATGACATTTTATGATTGGCCTGCATAA

TGGCTTTCCTAGTTAAAATACCACTATATGGTTTTCACTTATGACTCCCC

AAAGCCCATGTTGAAGCCCCTATTGCCGGATCAATAGTCCTAGCAGCTAT

TTTGTTAAAACTCGGAGGATATGGAATTCTACGCATTATTATCATCCTAG

AACCCATCTCCAAACTCATAGCCTACCCCTTCATTATTTTAGCAACCTGG

GGTATGATTATAACTAGCTCTATTTGCCTACGACAAACGGACCTCAAGTC

ACTAATCGCCTACTCATCAGTCAGTCACATAGGCTTAGTAGTAGCTGCCT

CCTTGATCCAAACACCCTGAGGTTTCATAGGAGCTACAGCTATAATAATT

GCTCACGGACTCACATCCACAATACTATTTCGCCTAGCCAATACTAACTA

CGAACGAGCCCACAGTCGGACAATAGTCCTAATCCGAGGCCTCCAAATAG

TCCTACCTTTAATAAGCTCATGATGACTACTGGCCAGCCTAGCCAACCTA

GGACTACCACCAACTATTAACCTAATCAGTGAATTAATAATCATTGTCTC

TGCATTCTCATGATCAAATCTTACTCTAATTTTACTAGGGTTAAACACTG

TTATTACAGCTATCTATTCCTTATATATATTAACTTCCGTTCAACGAGGC

AAAATAACCACCCACTCCTTATCTATTAACCCAACCTTCACCCGAGAGCA

CATAATCATAGCCCTCCACTTACTTCCCCTTATTCTCCTAACACTTAACC

CAAAATTAATTCTGGGGGTAGCTTACTGTAAATATAGTTCAACAATAACA

TTAGATTGTGAATCTAAAATTAGAAGTT--TAATTCTTCTTATTTACCGA

GAGAGAA----ATAAGAACTGCTAATCCTTAA-TCTCATGTCTAACCACA

TGACTCT-------------------------------------------

-------------------------ACTTTTAAAGGATAAAAGTC-TTCC

ATTGACCTTAGGAGTCAAAA---TTTGGTGCAATTCCAAATAAAAGTAAT

TAACCTGATATTTACCTCCACCCTTTTAATATCCCTAATTATCTTACTTA

TCCCCCTCTTAACATCATATACACCCTTTTACAAACTCACAACATACCCC

CACCACGTAAAAAATATGACAATATGATCTTTCATTATCAGCCTTTTCCC

CCTCCTACTATTCCTTAACCAAGGTTTTGAGTCAACCGTTACCAACTGAC

ACTGATTTACTTCCCAAACACTTGGACTAACAATAAACTTTAAAGTAGAC

CTCTATAGCATCATCTTCCTACCTATTGCTCTTTTAGTCACCTGATCAAT

CATAGAATTCTCCATTTGATATATAAGCTCCGACCCAAAAATCAACCAAT

TTATAAAATACCTATTAATCTTTTTAATCACCATATTAACTCTAATCTCC

GCCAGCAACCTTTTTCAACTATTTGTCGGTTGAGAAGGAGTAGGAGTAAT

ATCCTTTATGCTCATTGGCTGATGACACGCTCGGACCGACGCAAATACGG

CAGCCATACAAGCTATCCTCTACAATCGAATCGGAGACATTGGGTTTATT

CTAGCTATAGCCTGATTTATTATAAACTCAAACTCATGAGAGCTTAATCA

GATTTTCCTACTCCACATAGACCTA------CTCCCCCTGTTAGGTCTAA

TCCTTGCAGCAACAGGCAAATCAGCTCAATTTGGTCTACACCCATGACTT

CCATCCGCCATGGAAGGGCCAACCCCTGTCTCAGCCCTACTACACTCAAG

CACAATAGTCGTAGCTGGGATCTTCTTACTAATCCGATTTTCCCCTATGT

TT-GAACGAAATTATGTCGCCCTAACAGTCGCCTTATGTTTAGGGGCCAT

CACTACTTTATTCACAGCTGCATGCGCCCTAACACAAAATGACATACAAA

AAATCGTTACTTTTTCCACCTCAAGCCAACTAGGGTTGATAATAGTAACT

GTTGGGCTCAACCAACCATTTCTAGCCTTCCTTCACATTTGCACCCACGC

CTTCTTCAAAGCTATACTATTCCTATGCTCAGGATCTATTATCCACAATC

TTAATGACGAACAAGATATTCGAAAAATGGGAGGCCTGGTTAACACCCTA

CCAATTACATCATCAGCCCTAATCATTGGAAGCCTAGCTTTAACAGGGAT

ACCATTTTTAGCCGGCTTCTACTCCAAAGACTCAATTATTGAGTCTCTTA

ACATATCTAACGCAAACGCCTGAGCCCTGTGCCTAACACTCGTAGCCACC

ACATTCACCGCTGTATACAGCACCCGATTAGTTTTCCTTGCTCTACTCAA

CCAACCTCGATTCTCCCCAATAAGCATGATCAATGAAAACAACCCATTAC

TAATCAACCCTATTAAGCGACTTGCTTGCGGAAGTGTTATCGCTGGTTTC

CTCCTAACTACCTTCATCAACCCAACCACCCTAAACCCCACAACCATACC

TCTCTACATCAAAATAGCTGCCATTACTGTTACACTACTTGGCTTCCTTC

TAGCCTTAGAATTATACATAGCAACCAACAATCTAACCCATAAACCCCAC

TCCCAAAT-CTACTCCTTTTCCAACTTACTTGGTTATTTCCCACTAATCA

TCCATCGTAAACCCACCACTCAAAACTTTTCCCTAAGTCAAAGTATAGCA

ACCATATTAATCGACCTAACGTGGTTTGAAAAATCGGGCCCTAAGGGAAT

CTCCGCACAACAAATAACCTTTTCTTCATCTATCACAGAAACACAAAAGG

GGTTAATAAAAATCTACTTCCTATCATTTCTAATTACACCCCTTATCATC

ATCATATTCTTTCTT--------------------ACCTAATGCTCACCA

CGAGTCACCTCAAGTACCACAAAAATAGTTAAAAATAAAATCCACCCTGA

AAAGACCAACTCCCAACCCCCACAAGCATATAATAAGGACACACCATTAA

AGTCCCCACCCAACACCTCCATCCCCCCTAAATCCAACAAGTCATAACCC

ACCCCTAAGTCAA---------------------------CCTCCTCAAA

AAACTCATACCAAACTACCCCTAATATTCCTACCATAATAGCTAA-----

----ATTTAATACCACAGTATAATCTACCCAAGTCTCAGGATACTCCTCA

GTCGCTATAGCAGCTGTATACCCAAAAACAACAAGTATACCACCAAGATA

CACCAAAAAAACAATTAACCCTAAAAAGGACCCCCCTAAACTCACAATAA

TACCACACCCAACACCCCCACTTAAAACCAAACTTAACCCTCCATAAATA

GGTGAAGGGTTGGAAGCAAAAGCCATAAAACTCAACACCAATATAACACT

TAATAAGTAAATAAAATACATCATTAT----TCTCGCCTGGA-TTCAAAC

CAAGACCTATGATATGAAAAACCATCGTTGT-TATTCAACTACAAAAAC-

------TAATGCACAACCCACGAAAAACCCATCCTCTAATTAAAATTGTC

AACCACACATTCATCGACCTACCCACCCCATCCAACATCTCATCTTGATG

AAACTTTGGCTCCCTACTAGGCATATGCCTTATCGTCCAAATCCTCACAG

GCCTATTTCTCGCCATACATTACACCGCAGACACTACAACGGCATTTTCA

TCCGTTGCCCACATTTGCCGAGATGTAAATTACGGATGACTCATTCGCTA

CCTACACGCTAACGGAGCTTCTCTATTTTTTATTTGCATCTTCCTCCACG

TAGGCCGGGGACTTTACTATGGATCCTACATAAACACAGAAACATGAAAC

ATTGGAGTAATCCTACTATTCACCGTCATAGCAACTGCTTTTGTCGGCTA

CGTTCTTCCCTGAGGCCAGATATCTTTCTGAGGCGCTACAGTCATTACTA

ACCTTTTATCAGCCATCCCTTATATTGGAACTACTTTGGTAGAGTGAATC

TGAGGGGGATTCTCGGTAGACAAAGCAACACTAACCCGCTTTTTTGCGTT

CCATTTCATTCTTCCGTTCGTAGTTGCTGCCCTAACAATCATCCATCTTT

TATTCCTACATGAAACAGGGTCCAACAACCCATCTGGATTAAACTCAGAC

TCAGACAAAATCCCTTTTCACCCCTACTACTCAATCAAGGACCTCTTAGG

ATTTTTTATCGCCACCCTCGCCCTAATACTACTTGTCCTATTCACCCCAG

ATCTCCTAGGAGATCCAGACAACTACACCCCTGCCAACCCACTAAGTACC

CCACCCCATATTAAACCAGAATGATACTTCCTATTTGCCTACGCAATTCT

ACGCTCAATCCCCAACAAACTAGGGGGTGTTTTAGCCCTTGTAGCTTCAA

TCATAATCCTAGCCTTAATCCCTATACTCCACACATCTCGCCAACGGGGT

TTAACTTTTCGTCCACTAACCCAAACCCTCTTTTGAATTCTAGTCACAAA

CCTGTTAACGCTAACCTGAATTGGAGGTCAACCAGTCGAACAACCGTTTA

TCCTCATTGGCCAGCTCGCTTCAATTCTTTACTTTCTTCTAATTACAGTC

CTTATCCCATTTGCAGGGTCCCTGGAAAATAATCTACTAAAGTGATA---

--GCCTAAGTAATTTAAT---CAAAATCTTGGTCTTGTAAGCCAAACATG

AGGATGAAACTTCCTCCTAAGGCCTC-------AGAAGGAGGGCTTAAAC

CCTACCATCAGCTCCCAAAGCTGAAATTCTAAA--TAAACTACCTTCTG-

--------------------------------------------------

--------------------------------------------------

--------------------------------------------------

--------------------------------------------------

--------------------------------------------------

--------------------------------------------------

--------------------------------------------------

--------------------------------------------------

--------------------------------------------------

--------------------------------------------------

--------------------------------------------------

--------------------------------------------------

--------------------------------------------------

--------------------------------------------------

--------------------------------------------------

--------------------------------------------------

--------------------------------------------------

--------------------------------------------------

--------------------------------------------------

--------------------------------------------------

--------------------------------------------------

--------------------------------------------------

--------------------------------------------------

------

>Burramys_parvus Burramys parvus voucher ABTC76089 mitochondrion, partial genome.

---------------------------------------------T---T

AGATGGACCCTAACAAGTCCCATGAACATAAA--GGTTTGGTCCTAGCCT

TACTGTTAATTATAATTAAAACTACACATGCAAGTTTCCGCTGCCCAGTG

AGAATGCCCTTAAAA-TTCCATAA-----AATCAAAAGGAGCCGGCATCA

GGCACACCC----TAGGTAGCCCATTACGCCTTGC-TTAACCACACCCCC

ACGGGAAACAGCAGTGACTAACATTAAGAC-ATAAACGAAAGTTTGACTA

AATTATAATTATA---TAGGGTTGGTAAATTTCGTGCCAGCCACCGCGGT

CATACGATTAACCCAAATTAACAGAAA-GACGGCGTAAAGTGTGTTTAAG

CTAAA-----CAAGTAAAATAAAGCTAAGATTAGACTAGGCTGTAATACG

CCAGAGTTGACA-TCAAAATAAACAACCAAAGTGGCTTTAA-C--TCCGC

TGAGTACACGAAAGCTAAGACCCAAACTGGGATTAGATACCCCACTATGC

TTAGCCCTAAACCTAGATAGTT-ATTATAACAAAACTATTCGCCAGAGAA

CTACTAGCCAGCGCTTAAAACTCAAAGGACTTGGCGGTGCCCTAAACCCA

CCTAGAGGAGCCTGTTCTATAATCGATAAACCCCGATAGACCTCACCTCT

TCTTGCTAATA-CAGTCTATATACCGCCATCGTCAGCTTACCCCAAC-AG

GGAGGAAAAGTAAGCAAGATAAT--TAACCATAAAAACGTTA-GGTCAAG

GTGTAACATATGAAGAGGA-AAGTAATGGGCTACATTTTCTATTTTAGAA

CA--------------CAACGGATCATCTTATGAAACCTAA--GATATGA

AGGAGGATTTAGCAGTAAATC-AAGAATAGAGAGCTTGGTTGAA-ACAGG

CAATAGGGCGCGCACACACCGCCCGTCACCCTCCTCAATATTA-AACCTA

CATTTACCTAATAAATTAGATAATCAA--A--------------AGAGGA

GAAAAGTCGTAACATGGTAAGTGTACTGGAAAGTGCACTTGGAGTA-CCA

AAATGTAGCTTA-----TATAAAGCATTTAGCTTACACCTAAAAGATTTC

AGCTAACCCTGACCATTTTGAGCTAT-TACAA-AGCCCTACTACCA----

ACCCAAATCAACTATCAATT-TTTATTAACCAAAACATTAATC-TAA---

--CCTTAGTATAGGTGATAGAA---CAGTTAATCCAGGCGCAATAACGTT

AGTACCGTAAGGGAAT-AATGAAAGATCTACTT-ACAGCATAACAAAGCA

AAGATTAAACCTTCTACCTTTTGCATAA-TGGTTTAGCCAGTCTAC-CCG

GACAAAAAGAA--TTATGCCCGTCTCCCCGAAATCAAGTGAGCTACTATA

AAACAGTTT-A-CCAGAACCAACTCATCTATGTAGCAAAATAGTGAGAAG

ATTTTATAGTAGAGGTGAAAGGCCTACCGAACTTGAGGATAGCTGGTTGT

CCAAAACACGAATTTAAGTTCGACTTTAAATT-TAACTAAAGTACCAATA

AA--CACAATTTAAATTTAAAAGCTATTCAAAAGGGGGACAACCCTTTTG

A-T-ATGTGAACAAACTTTTTTAGAGGGTAAT-----GACTAGTATTGAT

TCATTGTGGGCCTAAAAGCAGCCACCAATTATGAAAGCGTTAAAGCTCAA

ACT------TACAAATAACTTAATACCCATAATT----CCACCAAAAC--

CCCTAATTCATTATTGGACGATTCTATAAATTCAT-AGAAGACATAATGC

TAAAATTAGTAACAAGAAATAC---TTCTCCCTGCACAAGCCTGCATTAG

CAACGGAACATCCACTAATAATTAACAAGCAAATAAAA--ACAACCTTCC

CACTAGCACT--ATTATTAACTTACTTGTCAACCCGACACAGGTGTGCAT

CTTATAAGGAAAGATTAAAAAGAATAAAAGGAACTCAGCAAACACAAACC

CCGCCTGTTTACCAAAAACATCACCTCTAGCATATCAAATATTAGAGGCA

CCGCCTGCCCAGTGAGCTTAAACCTCTTTAACGGCCGCGGTATCCTGACC

GTGCAAAGGTAGCATAATCATTTGTCTCTTAATTAGGGACTTGTATGAAT

GGCTTCACGAGGGTTTAACTGTCTCTTATCCTCAATCAGTGAAATTGACC

TTCCCGTGCAGAGGCGGGGATGTACATACAAGACGAGAAGACCCTGTGGA

GCTTAAAATTCATAACCTACTTAATTAACAACTTCACCC-------TAAG

GGACTAACACTTAAATTACGTATAGGTTATAATTTTTGGTTGGGGTGACC

TCGGAGTAAAAATCAACCTCCGAATGAC---TAAACCTAGAT-CAACAAA

TCTAAGT-GTAACAAAACCAGTAATTGACCCAT-----ATTTTGATCAAC

GGAACAAGTTACCCCAGGGATAACAGCGCAATCCTATTTAAGAGCCCATA

TCGACAATTAGGGTTTACGACCTCGATGTTGGATCAGGACATCCCAATGG

TGCAGCCGCTATTAAAG-GTTCGTTTGTTCAACGATTAA-AGTCCTACGT

GATCTGAGTTCAGACCGGAGAAATCCAGGTCGGTTTCTATCTGTATAT-T

TATTTCTCCCAGTACGAAAGGACCAGAGAAATAAGGCCAACATAAATTCA

TGCGCCTTAAAGACAAGATATGAAT------TCATCTAAATATCTTAACT

TATCCTCTCCAAACTCAAGAAATAGAG---CAATTAAGGTGGCAGAG-T-

GGTAA-TTGCATAAAACTTAAGCCTTTATTACCAGAGGTTCAATTCCTCT

CCTTAATA----C-------------------------------------

------------------------------------------ATGTTTAT

CATTAATTTGCTTCTCTATATTGTTCCTATTCTATTAGCTATTGCCTTCC

TTACACTAGTTGAACGTAAAGTCTTAGGTTACATACAATTTCGCAAAGGA

CCTAATGTTGTAGGGCCTTATGGCCTTCTACAACCCGTTGCTGACGGAGT

AAAACTATTCACAAAAGAACCACTTCGTCCCCTTACATCCTCCATTTCCA

TATTCATTATTGCCCCTGTACTAGCTTTAACCCTAGCCCTCACTATCTGA

ACACCCTTACCTATACCCCATTCTTTAATTGACTTAAATCTAGGGTTACT

TTTCATTCTCTCACTTTCAGGACTATCAGTCTATTCCATTCTTTGATCGG

GTTGAGCCTCCAATTCCAAATATGCCTTAATTGGAGCCCTACGAGCCGTA

GCCCAAACTATTTCATATGAAGTATCATTAGCCATTATTCTTTTATCCAT

CATACTATTCAACGGCTCCTTTACCTTAAAAAACCTAAGTGTTACACAAG

AAAATATGTGATTAATCGTAGCTACATGACCACTGACCATGATATGATAT

ATCTCAACACTAGCAGAAACCAATCGAGCTCCCTTTGATCTAACTGAAGG

AGAATCAGAGCTTGTATCTGGCTTTAATGTAGAGTATGCAGCAGGACCAT

TCGCCATATTCTTTTTAGCCGAATATGCTAACATTATGGCTATAAATGCT

ATAACCGCTATCCTATTCTTAGGCTCGTCAGTTACCTCCAATTATAGTCA

TCTCGATACCTTATCCTTCGTAATAAAAACCACTCTACTAACAGTCTTAT

TCCTATGAGTACGAGCCTCTTACCCTCGATTTCGATATGATCAGCTTATA

TATTTATTATGAAAAAATTTCCTACCTATAACACTAGCCCTATGCCTATG

ATATATTTCTATTCCAGTTGCCCTGTCATGTATCCCCCCACAAATCTAA-

GAAATATGTCTGACAAAAGAGTTATCTTGATAGGATAAATCATAGGGGCG

CA-AACCCCCTTATTTCTAGAAAGATAGGAATTGAACCTACATCAAAGAA

TTCAAAATTCTACATGTTTCC---ATTACACTACATTCTA----------

GTAAGGTCAGCTAAAT-AAGCTATCGGGCCCATACCCCGAAAATGTTGGT

TTACACCCTTCCCATACTAATGTCACCATATGTAATAATCGTAATCTTTA

CCAGCCTTCTTTTAGGAACCTCATTAACCCTATTTAGTGGCCACTGAATC

ACAGCCTGAATAGGACTAGAAATTAATACACTAGCAATTATTCCAATAAT

AACATACCCCAACCACCCCCGAGCCACAGAATCCGCCATCAAGTATTTCC

TCACCCAAGCAACTGCCTCAATAATAATTATATTTGCTATCATCTACAAC

GCCTGAACAACTAACCAATGATCTCTATTTCAAATTCCTGACCACCTAGC

CTCTACCCTTATAACAATTGCTCTTGCCACAAAACTAGGCCTGGCACCAT

TCCACTTTTGAGTCCCCGAAGTTACACAAGGAATTCCACTTACATCGGGC

ATAATTCTCCTAACCTGACAAAAAATCGCCCCAACATCGCTAATATATCA

GATCTCCCCTTCGCTCAACATAAAACTACTAATTACCCTAGCAATTTTAT

CAACTTTACTAGGAGGTTGAGGAGGTCTTAATCAAACTCACCTACGAAAA

GTCCTAGCATACTCATCCATTGCCCACATAGGATGAATAACTATTATTAT

TCTTATTAATCCAACTATAACCATTCTCAATTTAGTCATCTACATTATAG

CTACCCTCACCCTTTTTCTTACCCTTAACTTCGCATCCATTACCAAAATC

AAATCACTAGCAAATTTATGAAACAAATCAGCTCCAATAACCATTATCAT

CCTATTAACACTACTATCTTTAGGAGGCCTTCCCCCACTAACTGGCTTTA

TACCAAAGTGACTAATTCTCCAAGAACTTGTAGCCAATAATAATATTATT

ATAGCTACTCTCATAGCCCTATCAGCACTGCTAAACCTATTCTTTTACAT

ACGAATTATTTATGCATCAACACTTACCATGTTTCCATCAACCAACAACG

CTAAGCTTCAATGACCCAACTCACAAACAAAGTCAATTCGCATAATCCCC

ACATTAGCAATCTTATCCTCGATACTCCTCCCATTAACCCCAATATTTAT

TAATTTATCATATT----AAGAATT------ACAAGCCTTTATCTTGCAT

CATTCGAACGCAAATCGAACACTTTAACTTAAGCTAAATCCTTCAA---T

ATCAAGCCCCGGCAATTATTCAATTGCTTCTCTGAATTTGCAATTCAACG

TAA--TAAATACTTCAAGGCTTAAACAAAGGCT---------------TA

GGTTAAACATAGACCCAAGGCCTTC-AAAGCCTTAAGCAGGTGTTTAAAC

CACCTAGCCTTTGTCCACAATACACAAGCCTCACGCCTTGTAAGAAAAAA

A---AGGCGTGAG--GCCCCGGGGGTCTCAAAATAG-CACTGCCTCTAAA

TTGGTGGGTATTTATCCCACTAGATCTTAGTTAACAGCTAAGTACCTAAA

CATTTGGCTTCAATTTATT-----GGTAAAAAGAGATG----TTTAGTCC

CTGTCTTTGGATTTACAGTCCAATGCTTAC-CTCAGCCATTTTACC----

-------TATGTTCATCAACCGCTGACTATTTTCAACCAATCACAAAGAT

ATTGGTACCCTTTACTTACTATTCGGCGCCTGAGCAGGAATAGTAGGAAC

AGCCCTAAGCCTTTTAATTCGAGCAGAACTTGGCCAACCTGGCACCTTGA

TTGGTGACGACCAAATTTACAACGTAATTGTTACCGCACACGCTTTTGTA

ATAATCTTCTTTATAGTCATACCTATTATAATCGGAGGCTTTGGCAACTG

ACTAGTCCCTCTAATAATTGGCGCTCCCGATATAGCATTTCCTCGTATAA

ACAATATAAGCTTCTGACTTTTACCACCCTCATTCTTACTATTACTTGCT

TCATCCACAGTTGAGGCAGGAGCTGGAACTGGATGAACCGTTTACCCCCC

TCTAGCCGGTAATTTAGCGCATGCAGGAGCCTCTGTGGACTTGGCAATTT

TCTCCCTGCACTTAGCGGGAGTATCCTCTATTCTAGGGGCTATCAACTTC

ATTACTACAATTATTAATATAAAACCACCAGCCCTATCTCAGTACCAAAC

TCCGTTATTTGTATGATCCGTAATAATTACAGCCGTCCTACTACTTTTAT

CCCTTCCAGTACTAGCAGCAGGCATTACTATACTACTCACAGATCGAAAC

CTAAATACTACTTTCTTTGACCCCGCTGGAGGGGGCGACCCTATTTTATA

TCAACATCTGTTCTGATTCTTTGGCCACCCTGAAGTATATATTCTAATTC

TTCCTGGTTTTGGTATTATCTCTCATATCGTAACGTACTACTCCGGCAAA

AAAGAACCATTTGGCTATATAGGAATAGTATGAGCTATAATATCAATTGG

TTTTCTAGGATTCATTGTATGGGCCCATCATATATTTACCGTGGGTCTAG

ACGTTGACACCCGAGCATACTTTACCTCTGCTACCATAATTATTGCTATT

CCAACGGGAGTAAAAGTATTCAGCTGACTCGCCACCCTTCATGGAGGAAA

CATCAAGTGATCTCCAGCCATATTATGGGCCTTAGGATTCATCTTTCTTT

TTACAATTGGAGGTCTCACAGGAATCGTTCTAGCTAATTCATCCCTAGAT

ATTGTCCTACACGATACTTACTATGTAGTAGCCCATTTCCATTACGTACT

TTCCATAGGGGCCGTCTTTGCAATCATGGGCGGCTTCGTTCACTGATTCC

CTTTATTTACAGGTTATATGTTAAATGATATATGGGCAAAAATTCACTTC

TCCATCATATTTGTTGGAGTTAACTTAACATTCTTCCCTCAGCACTTCCT

TGGATTATCTGGAATACCACGACGTTATTCAGATTACCCAGATGCTTACA

CAACATGAAATGTAGTTTCATCAATCGGCTCTTTTATCTCACTAACTGCC

GTCATTTTAATAGTATTTATTATTTGAGAGGCCTTCGCTTCTAAACGTGA

AGTCTCTACAGTAGAACTCACTACAACCAATATTGAATGACTCTATGGTT

GTCCCCCACCATATCATACATTTGAGCAACCAGTCTTTGTAAAATCCAA-

----------------CTCAAGAAAGGAAGGAATCGAACCCCCAAAAATT

GATTTCAAGTCAACTCCATAACCTCTATGACTTTCTC---------ACAA

GATATTAGTAATAATCATTACATAACTTTGCCATAGTTAAATTATAGGTT

------TAAATCCTATATATCTTATATGCCTTATCCAATACAACTAGGCT

TCCAAGACGCCACATCCCCAATTATAGAAGAACTTATATACTTTCACGAC

CATACCCTTATAATTGTTTTTCTGATTAGCTCTCTAGTTCTTTATATTAT

TATTCTCATACTAACAACAAAACTTACACACACAAGCACAATGGATGCTC

AAGAAGTAGAAACTATTTGAACAATCTTGCCTGCTGTAATCTTAGTTCTT

ATTGCCTTACCATCCTTACGCATTCTTTACATGATAGACGAAATTTACAA

CCCCTACCTCACAGTAAAAGCTATGGGCCATCAATGATACTGAAGCTACG

AATATACTGACTATGAAGACCTCACATTTGACTCATATATAATTCCCACC

CAAGATCTAACTCCTGGCCAATTCCGATTATTAGAAGTAGATAATCGAGT

TGTCCTACCTATAGAGCTACCCATTCGTATACTCATTTCATCAGAAGATG

TGATCCATGCCTGAGCCATTCCATCCTTAGGTTTGAAAGCTGATGCCATT

CCCGGTCGACTAAACCAAGCCACACTAACATCAACTCGTCCTGGGGTCTA

TTATGGACAATGCTCAGAAATCTGTGGTTCTAATCATAGTTTTATACCTA

TTGTATTAGAAATAACTACATTGAAATACTTTGAAAATTGGTCCTCTATG

ATGCAATCA-----------------------------------TTTTTG

AGAAACTAGCTAATACTGAACGTATTTAAGG-------------AGA--A

ATTC-ACCCTCAAAAT-T-ATGCCACAATTAGACACCTCCACATGATTCC

TTGTAATTACCCTCA-TAACTATTTCACTATTCTGCGTATATCAACTAAA

AATAATTAACCAAACCATAATCTCCATTACTCCCCAGGACCAAAAAGACA

TTAATACAAAACAACAATTACCTTGAGAAAAATCATGAACGAAAATTTAT

TTGCCCCCTTCATCACCCCTACAATCATAGGTGTTTCTACACTACCAATT

ATCATTCTATTTCCCTGCTTAATTCTCAGCTCCCCAAAACGCTGACTACC

CAATCGCATTCAAACCCTCCAAATCTGATTAATTCGCCTAATTACT-AAA

CAAATAATAACAATACATAATAAACAAGGTCGAACATGAGCCCTAATACT

TATCTCCCTAATCCTATTTATTGCATCCACCAATCTCCTAGGCTTACTTC

CCTATTCCTTTACCCCTACAACACAACTTTCCATAAATATCGGGATAGCC

ATCCCCTTATGAATAGGAACTGTGGCAATAGGCTTCCGCAATAAAC-CAA

AAGCCTCCTTAGCCCACTTTCTTCCACAAGGCACTCCAACACCATTAATT

CCCATACTCATTATTATCGAGACAATTAGCCTATTCATCCAACCATTGGC

CCTAGCAGTTCGATTAACAGCGAATATTACCGCTGGCCACCTGCTCATTC

ATCTCATTGGCTCCGCTACGTTGGCTCTGTCTTCAATCAGCATAACAGTC

TCAGCCATTACCTTCACCATTCTATTCCTACTCACTATCTTAGAACTAGC

TGTAGCCATAATTCAAGCCTATGTCTTTACGCTTCTAGTAAGCCTGTACC

TCTATGACAACGCCTAATGACTCACCAAACGCACGCATTTCACATAGTTA

ATCCAAGCCCATGACCACTCACAGGAGCTCTTTCAGCTCTTCTTCTCACA

TCTGGCCTCACCATATGATTTCACTTCAATACTCCCCTCCTACTAATCAT

TGGCATCACATGCCTACTATTAACAATATATCAATGATGACGCGATATCG

TACGAGAAGGAACATTCCAAGGACATCACACACCCGTAGTACAAAAAGGC

CTACGATATGGAATAATTTTATTCATCCTATCAGAAGTATTCTTTTTCTT

TGGATTCTTCTGAGCTTTTTATCATTCAAGTTTAGCACCAACTCACGAAC

TAGGTGGCTGCTGACCACCTACTGGTATTCACCCCCTTAATCCACTTGAA

GTTCCATTGCTAAATACAGCCATTCTGCTGGCTTCCGGAGTTTCAATCAC

ATGAGCCCATCACAGCCTTATAGAAGGGAATCGTAAACAAATAATTCAAG

CATTAATCATTACAATTCTTCTTGGCTTGTACTTCACAATTCTCCAAGCA

CTAGAATACTATGAAGCTCCCTTTACAATCTCAGACGGTGTTTACGGTTC

AACCTTCTTCGTGGCAACAGGCTTTCACGGCCTTCATGTTATTATTGGAA

CAACCTTTTTAATCGTATGCCTCTTCCGCCAATTCAACTTCCACTTCACT

TCCACCCACCACTTTGGATTTGAAGCAGCCGCCTGATATTGACACTTCGT

AGATGTAGTATGACTTTTCCTCTATATTTCAATTTATTGATGAGGCTCTT

ATTTT-TCTAGTAT--AATTAGTACTACTGATTTCCAATCATTAAGTTCT

GGGTA-AAACCAGAGAAAAATAATGAACCTTATTATTACACTCATCATCA

ACACTACCCTATCAACAATTATTGTCCTGATCGCTTTTTGACTTCCCCAA

TTATATCTTTACTTAGAAAAATCTAGCCCCTACGAGTGCGGGTTCGACCC

ACTAGGCTCAGCCCGCCTACCCTTTTCAATAAAATTTTTTTTGGTTGCTA

TTACATTTCTTCTATTTGATCTAGAAATTGCTCTACTTCTACCTCTTCCA

TGAGCCATTCAACTGTCTTCCCCAAAAATAACACTACTTCTGGCCTACAG

TCTTATTCTCCTTCTATCAGCTGGCTTAGCCTACGAATGAAAACAAAAAG

GCTTAGAATGAACTGAGTA-------GGTCTTTAATCTAA--TTAAGATG

CTTGATTTCGACTCAATTTATCATGGTCTCAATCCATGAAGACC---TTA

TGATATCCATCAACTCAAACCTAATCATAGCATTCTTTCTTGCTCTAGCA

GGCGTATTAATTTATCGCTCTCATCTCATATCAACTCTTATATGCTTAGA

GGGGATAATACTATCCTTATTTATCCTAATAGCCCTTCTAATCTCCCACT

TCCACATATTTTCCCTATCCATAGCCCCCCTAATCCTCCTAGTATTCTCA

GCCTGCGAAGCAGGCGTAGGCTTAGCCTTATTAGTTAAAACCTCCAATGA

TTACGGCAATGACTACGTCCAAAATCTTAATCTCCTCCAATGCTAAAAAT

CCTTATTCCTACTCTCATGCTAATTCCACTTACCTGATATTCAAAAAAGC

CATGAATATGAATCAACTCCACATCCCATAGCTTATTAATTAGTGTAATT

AGCCTATCTCTCCTATACCACAACGACGACCTAGGGTATAATTTCAACAG

CTCTTTCTGCATAGACTCACTCTCAAGTCCCCTCCTAGTTTTATCCTGCT

GACTTCTACCCCTAATAATAATTGCCAGTCAAAACCATCTAATAAAAGAA

TCTACTAATCGAAAAAAAACATATTTAACGATATTAATTATTCTACAATT

ATTCCTAATTATAGCATTCTCCTCATCAGAACTAATAATATTTTATATTT

TATTCGAAGCTACCCTGATTCCCACATTAATTATTATCACTCGATGAGGT

AATCAAAATGAACGACTCAACGCTGGCATTTATTTTTTATTTTACACCCT

CGTAGGATCCTTACCCCTTCTAATTGCTCTACTTTTACTATATAATAATC

TAGGAACCCTGCATATTTTAACTGTTTCCATACTCTCTCACTCAATAAAA

ACCTCT----------CTACCTAATTCTCTCCTATGGTTCGCATGCATAA

CAGCATTCATAGTAAAAATACCTCTATACGGTCTTCACCTGTGGCTACCA

AAAGCACATGTAGAAGCTCCTATCGCCGGCTCTATAGTTTTAGCCGCTAT

TCTACTAAAACTAGGAGGCTATGGGATTATACGAATAACTGTCTTCACTG

AACCAATCACCTCCCATCTATACTACCCCTTTATTATTCTATCCCTATGA

GGCATAATTATAACTAGCTCCATCTGCCTCCGCCAAACAGATCTAAAATC

ACTTATTGCTTACTCATCAGTTAGCCATATAGCTTTAGTAATCGTAGCAG

CCCTAATACAAACCCCTCTAAGCTTCATAGGTGCTACAACCCTAATAATT

GCACATGGCCTAACTTCTTCCATATTATTCTGTCTAGCTAATACAAACTA

CGAACGAATCCACAGCCGAACTATAGTCTTAGCGCGTGGCCTGCAAACTA

TTCTCCCCCTAATATGCGTATGATGACTACTTGCAAGCTTAGCTAATCTA

GCTCTACCTCCCACAATCAACCTTCTAGGTGAACTGACAGTTATTATCTC

TTCTTTCTCCTGATCCCAATTCTCCATCATCCTTTTAGGCATTAACACCG

CAATCACAGCGCTGTATTCACTATATATACTTATTACTTCTCAACGAGGC

AAATTCACATATCACCTATACCCTACCAACCCCTCATTTACACGAGAACA

CTTACTAATAGTTCTTCACCTCTTCCCTTTATTAATCCTGTCACTAAGCC

CCAAATTCATCCTAGGACCGACATACTGCATATATAGTTTAACAAAAACA

TTAGATTGTGAATCTAAACACAGAAGTT--TAAATCTTCTTATACGCCGA

GAAAGTTC----CAAGAACTGCTAATTCTTGATACCGTAAATAACACTTA

CGGCTT--------------------------------------------

-------------------CCTCA--CTTTTAAAGGATAGAAGTA-ATCC

ACTGGTCTTAGGAACCAACAACT-TTGGTGCAAATCCAAATAAAAGTAAT

TAACTACTTACTTAACACCTCAATTCTCCTCTCAATTATTACACTTACCC

TACCCCTTATTCACAACCTTATTCTCCCTAACAAAATCAATCAATTCCCT

CTATACTGCAAAAACACTATCAAAATGGCATTTCTCATCAGCCTCGCACC

ACTATTGCTGTTTATTAATCTAGGTTATGAATCAACCATTACCAATTGAC

AATGATTCTCAATTAACTCATTTAATCTAACTATAAGCTTTAAACTAGAC

TACTTCTCAATTATCTTTATTCCAATCGCGTTATATGTCACCTGGGCTAT

CCTAGAATTCTCACTATGATATATACACTCAGACCCATATATTCATCGAT

TCTTCAAATACTTAATTACATTTCTACTTACTATAATTATTCTAGTTTCC

GCTAACAACCTATTTCAATTATTCATCGGCTGAGAAGGCGTTGGCATCAT

ATCTTTTATACTAATTGGGTGATGATTTGGTCGTACAGACGCCAACACAG

CAGCCCTCCAAGCCATCCTCTACAACCGAATTGGAGATATTGGCTTTATA

TTAACAATAGCCTGACTAATAATTAATAATAACTCATGAGATCTCCAACA

CATCTTTATAACTAATATAAACACT------TTAGCACTTTTAGGTCTTA

TCATCGCCGCAACCGGTAAATCAGCTCAATTCGGACTCCATCCATGACTA

CCCTCCGCAATAGAAGGCCCAACTCCAGTATCAGCACTTCTTCACTCTAG

TACAATAGTAGTTGCCGGAATCTTCCTACTAATCCGATTCCACCCCATAC

TC-GAAGATAACCAGCATATCCTCACCATTGCTCTTTGTTTAGGAGCAAT

TACCACCCTTTTTACCGCTATCTGTGCTATTACCCAAAACGATATTAAAA

AAATTGTAGCATTCTCAACATCAAGCCAGCTAGGCTTAATAATAGTAACA

ATCGGCCTTAACCAACCTCATTTAGCATTCCTTCACATCTGTACCCATGC

ATTCTTCAAAGCCATATTATTTCTCTGTTCAGGGTCAATCATTCACAGTC

TCAATGACGAACAAGACATCCGAAAAATAGGGGGTCTACTAACAACAATG

CCCATTACCTCTTCAGCCCTAATAACTGGCAGCTTAGCACTAATAGGCAC

GCCCTTTCTAGCTGGGTTTTACTCCAAAGATTCAATCATTGAAGCTATAA

ACACCTCCTACACCAACACATGAGCCCTAACTATCACTATAATTGCTACC

TCACTAACAGCCATCTACAGCATACGAATTGTCTACTTCGCCCTACTAAA

CCAACCACGCTTTCTCCCCATATCACCCATCAACGAAAATAATCCCAACC

TTATTAACCCTATTACCCGCCTTGCATTAGGCAGTATTTTTGCAGGCTTC

TTACTAACAATAAATGTTCCTCCAACAACACTAGTTCCAATGACTATACC

TTCAATAATAAAATTATCAGCCCTTATTGTAACAATTATTGGGCTCACAA

TCGCTATAGAACTAAATTCATTAACCAATAAGTCCCCAATAGCACTTTCA

ATCCATAC-GCACAACTTCTCTAATATGCTAGGATACTTCACCCACATCT

TCCACCGACTATACCCCCTAGCAAATTTACAAATAGGCCAACATATTGCT

ACCATACTAATTGATTTAAACTGATACGAAAAAACCGGGCCTAAAGGTCA

AGCTAACCTCCACAGTTCCATATCTTCATCTATTTCTTCTGCCCAAAAAG

GCTTTATTAAAATTTACTTTATATCTTTCATTATCTCTATTCTAACAATT

ATTATTATTATTACTTAAT-T----------------------GCGACCT

CGAACAACTTCCAAAACAATAAAAATAGTAATAAATAAAATTCAACCTAA

TAAGACTAAAGCCCATCCTCCACAACTATATAACAAGGACACACCACTAT

AGTCCTGACCTACACAATAATTACCAATAGTATCAAATAAC---------

---------TCAACAGCAGTAATCACTTCAA------------CTTCATC

TGACATAAAATACCAAACCA------CTTCAACTAATATAGTAAATAGCA

GTATACTTAATGCCACCGCATTACCAACTCAACTTTCAGGGTACTCTTCA

GTCGCCATAGCAGCAGTATATCCAAAAACTACTAATATGCCTCCAAGATA

AACTAAAAAAACAACTAAACCTAAAAAAGTATCTTCAAGACTCACAACAA

TTGCACAACCTAAACCTCCACTCACTACAAGGCTTAATCCCCCATATACA

GGAGAAGGCTTAGAGGCAAAAGCAACAAATCCAAAAATTAACAGAAGTGA

AAATAAAAAAATCACCATTATTTTCATCA-TTTTAGTATGGACTTTA-AC

CATAACCTATGGCATGAAAAACCATTGTTGTCT-TTCAACTACAAAAAC-

------TAATGATCAACCTACGTAAAACCCACCCATTACTAAAAATCATT

AACCACTCTTTTATTGATTTACCTGCACCTTCCAATATTTCCGCCTGATG

AAACTTCGGGTCTCTTCTAGGAATCTGCTTAGTTATCCAAATTCTTACAG

GCCTATTCCTAGCTATACATTATACATCAGACACCTTAACCGCCTTCTCG

TCTGTAGCCCATATCTGTCGAGACGTAAATTACGGTTGACTCATCCGTAA

TCTTCATGCCAACGGGGCATCCATATTCTTTATATGCCTTTTCCTCCATG

TTGGACGAGGCATCTATTATGGCTCCTATCTTTACAAAGAAACATGAAAC

ATTGGAGTATTCTTACTATTAACCGTCATAGCCACAGCATTCGTAGGGTA

TGTGCTTCCATGGGGTCAAATATCCTTCTGAGGCGCAACTGTCATTACCA

ACCTCCTATCAGCCATCCCATACATTGGTACCACCTTAGTTGAATGAATC

TGAGGTGGGTTCTCCGTAGATAAAGCTACTCTCACTCGATTCTTCGCTTT

CCACTTTATTCTCCCATTCATTATTACAGCCATAGTAATCGTCCACCTAC

TATTCCTCCACGAAACAGGCTCTAATAACCCCTCAGGTATTAATCCTGAT

TCCGATAAAATTCCCTTCCATCCATACTACACCATTAAAGACACATTAGG

CCTAGCTCTCATACTCCTTATCTTACTACTTCTAGCCTTATTCTCCCCAG

ACATACTTGGAGACCCAGACAACTTCTCTCCAGCTAACCCCCTAAACACA

CCCCCTCACATCAAACCAGAGTGATACTTTCTATTTGCATATGCAATTCT

CCGATCCATTCCCAATAAATTAGGCGGCGTTCTAGCCTTGCTAGCATCCA

TTCTAGTTCTATTGATCATTCCCTTCCTCCACACATCAAACCAACGCAGC

CTAATATTCCGCCCAATCTCCCAGACACTCTTCTGAATTCTAACCGCTAA

CCTATTTACCCTTACATGAATTGGGGGCCAACCTGTAGAACAACCATACA

TCATTATTGGACAAATTGCATCCATTATATACTTCCTTCTAATTATTGTT

CTCATACCAGCCGCAGGCCTATTCGAAAATTATATACTCAAACCTAAATG

AAGA-GTCCCAGTAATTTAACCAAAATACTGGTCTTGTAAGCCAGCAATG

AAG-----------------------------------------------

--------------------------------------------------

--------------------------------------------------

--------------------------------------------------

--------------------------------------------------

--------------------------------------------------

--------------------------------------------------

--------------------------------------------------

--------------------------------------------------

--------------------------------------------------

--------------------------------------------------

--------------------------------------------------

--------------------------------------------------

--------------------------------------------------

--------------------------------------------------

--------------------------------------------------

--------------------------------------------------

--------------------------------------------------

--------------------------------------------------

--------------------------------------------------

--------------------------------------------------

--------------------------------------------------

--------------------------------------------------

--------------------------------------------------

--------------------------------------------------

------

>Dromiciops_gliroides Dromiciops gliroides mitochondrion, complete genome.

---------------------------------------------TGCTT

AGACGGGCTTTCACAAGCCCCATAAACACAAA--GGTTTGGTCCTAGCCT

TACTGTTAATTCTAATTAGACCTACACATGCAAGTTTCCGCTAACCGGTG

AGAATGCCCTTAAAAACTATATAA-----AGTTAAAAGGAGCTGGTATCA

GGCACACCC--CCTGGGTAGCCCATTACACCTTGC-TTAACCACACCCCC

ACGGGATACAGCAGTGACTAACATTAAGCT-ATAAACGAAAGTTTGACTG

AGTCATAATTTAA---TAGGGTTGGTAAATTTCGTGCCAGCCACCGCGGT

CATACGATTAACCCAAATTAACAGACC-ACCGGCGTAAAGCGTGTTTAAG

TATAA-----TAC-TCTAATAAAGTTAAAACCTAACTAAACTGTAAAACG

TTATAGTTAATA-CCAAAATATGCAACTAAAGTGACTTTAATC--TACAC

TGAACACACGATAGCTAAGACACAAACTGGGATTAGATACCCCACTATGC

TTAGCCCTAAACCCAAATAGTT-AAAA-AACAAAACTATTCGCCAGGGAA

CTACTAGCTAATGCTTAAAACTCAAAGGACTTGGCGGTGCCCTATACCCA

CCTAGAGGAGCCTGTTCTATAATCGATAAACCCCGATAAACCCCACCTCT

TCTTGCCAATA-CAGCCTATATACCGCCATCGTCAGCTCACCCCAAT-AG

GGACCCAAAGTAGGCAAAACTAT-ATAATCATAAAAACGTTA-GGTCAAG

GTGTAGCATATGAAGAGGG-AAGCAATGGGCTACATTTTCTAAACTAGAA

CA--------------TAACGAATGGCCTTATGAAATCTAA--GACCTGA

AGGAGGATTTAGTAGTAAATT-AGGAGTAGAGAGCCTAATTGAA-ATAGG

CAATAGGGCGCGCACACACCGCCCGTCACCCTCCTCGATTATACAAACTA

AATAAA--TAATAAAGTTAATACATAATAA--------------AGAGGA

GAAAAGTCGTAACATGGTAAGTGTACTGGAAAGTGCACTTGGAATA-CCA

AAATGTAGCTTA----ATTTAAAGCATTTAGCTTACACCTAAAAGATTTC

AGTTAATTCTGACCATTTTGAGCCATGCGCTT-AGCCTT--TACAA----

ACCTCAAATAACTATTTTAT-TATATTTAC-AAAACATTTAAC-TCA---

--TCCAAGTATAGGTGATAGAA---CAGATA--CAATGCGCTATAACGAT

AGTACCGCAAGGGAAA-ACTGAAAGA-CAAATT-AAAGCATAAAAAAGCA

AAGACTAAACCTTTTACCTTTTGCATAA-TGATTTAGCTAGTTAAA-CCG

GACAAAAAGAA--TTACGCCCGACCCCCCGAAATTAAGTGAGCTACTATA

AAACAGTAT-A-TAAGAACCAACTCGTCTATGTAGCAAAATAGTGAGAAG

ATTTTATAGTAGAGGTGAAAAGCCAATCGAACTTAAAGATAGCTGGTTGT

CCAAAATACGAATTTTAGTTCAACTTTAAATTATATATTTAGTGAAAATA

AAACCACACCTTAAATTTAAAAGCTAATCAAAAGAGGGACAACTCTTTTG

A-TCATGTAAACAAACTTTATTAGAGGGTAAT-----GCTATCCTTACAT

ACATTGTGGGCCTAAAAGCAGCCACCAATTAAGAAAGCGTTAAAGCTCAA

ACA------TTATTACTCCTTAATCCCACTAATT----AAACAAAACC--

CCTAATATGAATATTGGATGATCCTATATTTTTAT-AGAAGACATAATGC

TAAAATTAGTAACAAGAATCCC---TTCTCCTTGCACAAGCTTATATTAG

TAACGGAAAACCCACTAACAATTAACAAACCAATAATT--ACAATCAAGA

-ACTAGCAAA--TTATATCACTTA-TTGTTAACCCAACACAGGTGTGCAT

CTAA--AGGAAAGATAAAAAAGAATAAAAGGAACTCGGCAAACTAAAACC

CCGCCTGTTTACCAAAAACATCACCTCTAGCATAACAAATATTAGAGGCA

TCGCCTGCCCAGTGAGTTAAA----CTTTAACGGCCGCGGTATCCTGACC

GTGCAAAGGTAGCATAATCACTTGTCTCCTAATTAGGGACTCGTATGAAT

GGCATGACGAGGGTTTAACTGTCTCTTATTCTCTATCAGTGAAATTGACC

TCCCCGTGCAGAGGCGGGGATATAAATACAAGACGAGAAGACCCTGTGGA

GCTTAAGATTCATGATATATCTCTATC-CATTTTCACCC-------GAAA

GGACTAAGATC---ACGAGCTCTATATCATAATCTTTGGTTGGGGTGACC

TCGGAGCATAAAACAACCTCCGAATGAC---ATAACCTAGATTCTACCAA

TCTAAGT-GTAGCAATACCAGTAATTGACCCAA-----ATATTGATCAAC

GGAACAAGTTACCCCAGGGATAACAGCGCAATCCTATTTAAGAGCCCATA

TCGACAATTAGGGTTTACGACCTCGATGTTGGATCAGGACATCCCAATGG

TGTAACCGCTATTAATG-GTTCGTTTGTTCAACGATTAA-AGTCCTACGT

GATCTGAGTTCAGACCGGAGAAATCCAGGTCGGTTTCTATCTGTATTT-T

TATTTCTCCCAGTACGAAAGGACAAGAGAAATAAGGCCAACATTA-CTAA

TGAGCCTTAGAAGTAATATATGAAA------TTATCTTAATATCTTAATT

CGACATCTTTACTCCATAGAAA-ATGG---TTATTAAGGTGGCAGAG-TT

GGTAA-TTGCGTAAAACTTAAACCTTTACACCCAGAGGTTCAAATCCTCT

CCTTAATA----T-------------------------------------

------------------------------------------GTGTTCAT

TATTAACCTACTCTTATATATTGTCCCTATCCTACTAGCAGTAGCCTTCC

TTACACTAATTGAACGAAAAGTCCTAGGCTACATACAATTCCGCAAAGGC

CCAAATATTGTAGGACCTTTTGGCCTACTTCAACCATTCGCTGACGCAGT

AAAACTATTCACAAAAGAACCCCTACGACCACTCACATCCTCAATACTAA

TATTCATTATTGCCCCAATTTTAGCCCTAACCCTAGCATTAACCATCTGA

ACACCCCTCCCAATACCTAACACCTTAGTAGATATAAATCTAGGCCTACT

ATTCATCCTAGCACTGTCAGGACTCTCCGTATACTCAATCCTATGATCAG

GCTGAGCATCCAACTCAAAATATGCCCTAATTGGAGCCCTTCGAGCTGTT

GCTCAAACCATCTCCTATGAAGTAACTTTAGCCATCATTCTCCTATCAAT

TATACTCATTAATGGCTCCTTCACTTTAAAAACACTTATTATTACCCAAG

AAAACATATGAATAGTTATCGTCTCATGACCACTAGCTATAATATGATAC

ATTTCCACCCTAGCCGAAACTAATCGAGCCCCTTTCGACTTAACTGAAGG

TGAATCAGAACTAGTATCAGGCTTTAACGTAGAATATGCCGCAGGCCCTT

TTGCCATATTCTTTCTAGCAGAATATGCTAATATTATCGCTATAAATGCC

ATAACCGCTATTCTATTTATAGGGACCTCAATTAACCTATTTTCCCCTCA

TGCTAGTTCATTAACTTTTATAATCAAAACAACAGCCTTAACACTCATAT

TTTTATGAATTCGAGCATCCTACCCCCGATTCCGATATGATCAACTCATA

CATTTATTATGAAAAAACTTCTTACCCATTACATTAGCCCTATGCCTCTG

ATTCATTTCCATCCCAATCGCACTAGCATGCATTCCTCCCCAAATCTAA-

GAAATATGTCTGACAAAAGAGTTATCTTGATAGGATAAATTATAGGGGTT

TC-AACCCCCTTATTTCTAGAACAATAGGACTCGAACCTATATCCAAGAA

CTCAAAATCCTCTGTGTTTCC---TTTACACCACATTCTA----------

GTAAGGTCAGCTAAAT-AAGCTATCGGGCCCATACCCCGAAAATGTTGGT

TTGCACCCTTCCCATACTAATGTCCCCTTATGTATTAATAATTATATCAC

TGAGTCTTCTTATCGGTACATCCCTTACCTTATTTGGAAACAACTTAATA

ACAGCCTGAATAGGCCTAGAAATTAATACCCTAGCAATTATCCCCATAAT

AACATACCCCAACCATATGCGAGCCTCAGAATCTGCTATCAAATATTTTC

TCACACAAGCAACCGCCTCAATAATAATTATATTTGCCATTATCTACAAC

GCTTGAATAACAAATCAATGAAACCTATTTCAAATTTCTAACCAATGAGC

CTCCATTATTATAACCTTAGCCCTAGCTATAAAGCTAGGGCTTGTACCCT

TCCACTTCTGAGTACCCGAAGTCACACAAGGAATCCCTCTATTATCCGGA

ATAGTTCTATTAACTTGACAAAAAATTGCCCCCGCATCAATCATATACCA

AATTTCCCCATCGCTCGACATAAAAATTTTAACCTTACTAGCCATTATAT

CAACCATCCTAGGTGGATGAGGAGGATTAAATCAAACTCACATCCGAAAA

ATCTTAGCCTATTCATCCATTGCACATATAGGATGAATAACAATCATTAT

TCTAATCAACCCAGCAATAACTCTACTTAACCTAATTATCTACATCTTTA

CTACATTAACAATATTCCTTATCCTTAACCACGCCTCAGTCACCAAAATC

AAATCACTCGCAAACTTATGAAACAAATCAGCCCCTATGACTATTATTAT

TCTTCTCACCCTACTCTCCTTAGGAGGACTCCCACCCCTAACAGGCTTTA

TTCCAAAATGATTCATTCTCCATGAACTAGTTACCAACAACAACATCACA

ATAGCAACCTCAATAGCCTTATCAGCCCTACTCAATCTCTTCTTTTATAT

ACGTATCATCTATGCAGCCGCCCTTACCATATTCCCCACAACCAATAACT

CAAAACTTCGTTGACTCCACACATCCACTAACATAACCTCAATAATTCCC

ACACTTACAATTATTTCATCTCTTCTTCTTCCTCTATCCCCCCTACTAAT

TAACCTAATATACT----AAGAACT------ACAAGCCTTTATCTTGCAT

CCTTCGAACGCAAATCGAACACTTTAACTTAAGCTAAATTCTCCCTATCC

TTCAAGCCCTGGCAGCAAT-CAGCTACTCCTCTGAATTTGCAATTCAACG

TAA--CAAATACTTCAAGGCCTCA-TAAAGACT---------------TA

GGCTAACC-TAGACCAAAGGCCTTC-AAAGCCTTAAGTAGGTGTTAAACT

CACCTAGTCTTTGCCCC--ATTCACTTCCCCTCCGCCTTGTAAGAAAAAA

A---AGGCGGAGG--GGCCCCGGGCACCCAAACAAGTCTTTACCTCTAAA

TTGGAGGGTTTTTATCCCACTAAATCTTAGTTAACAGCTAAGCACCTAAA

CATTTGGCTTCAATTTATTTCATTGGTAAAAAGAGATA----TTTAGTCT

CTGTCTTTGAATTTACAGTTCAATGCTTGC-CTCAGCCATTTTACC----

-------TATGTTCATTAACCGTTGACTATTCTCAACAAACCATAAAGAT

ATTGGCACCCTTTACTTACTATTTGGTGCTTGAGCAGGAATAGTTGGTAC

AGCTCTAAGCCTATTAATCCGAGCTGAGCTTGGCCAACCTGGAACCCTGA

TTGGGGATGATCAGATTTATAATGTAATTGTTACCGCTCACGCATTTGTC

ATAATTTTCTTTATAGTAATACCCATTATAATTGGAGGGTTTGGCAACTG

ACTAGTTCCTCTAATAATTGGAGCACCTGACATAGCTTTCCCTCGCATAA

ACAATATAAGCTTCTGATTATTACCCCCTTCATTCCTCCTATTGCTTGCA

TCATCTACAGTTGAAGCCGGGGCTGGAACCGGATGAACAGTCTATCCTCC

TCTGGCTGGCAATTTAGCCCACGCAGGAGCCTCCGTGGACCTTGCCATTT

TTTCACTTCACCTTGCAGGAATTTCATCTATTCTAGGAGCTATTAATTTC

ATTACAACCATTATTAATATGAAACCACCAGCTATATCTCAATATCAAAC

TCCACTATTTGTCTGATCAGTAATAATCACGGCAGTTCTTCTCCTTCTAT

CCCTCCCAGTTCTAGCTGCAGGAATTACTATATTACTTACAGATCGCAAC

CTAAATACGACATTCTTTGATCCAGCAGGTGGAGGAGACCCAATCTTGTA

TCAACATCTCTTCTGATTCTTTGGCCACCCTGAAGTATACATTCTTATTC

TTCCAGGTTTCGGTATTATTTCACACATTGTAACTTACTACTCTGGCAAA

AAAGAACCATTCGGTTATATAGGAATAGTATGAGCAATAATGTCCATTGG

ATTCCTAGGCTTCATTGTATGAGCCCACCACATGTTTACAGTAGGACTTG

ACGTAGATACTCGAGCTTATTTCACCTCAGCTACGATAATTATTGCAATT

CCTACTGGGGTGAAAGTATTTAGCTGATTAGCCACTCTCCACGGAGGTAA

CATTAAATGATCCCCAGCAATACTATGAGCCCTTGGGTTTATTTTCCTAT

TTACAATTGGAGGATTAACAGGAATCGTTCTAGCTAATTCATCTTTAGAT

ATCGTTCTTCATGATACCTACTATGTGGTAGCTCACTTCCACTATGTCCT

TTCAATAGGCGCCGTATTCGCAATTATAGGAGGATTTGTACACTGATTCC

CTTTATTTACAGGCTATATGCTTAGCGATATGTGAGCAAAAATCCATTTT

TTCATTATGTTTGTAGGAGTGAATATAACATTCTTCCCTCAACACTTCTT

AGGACTATCAGGCATACCACGACGTTACTCAGATTACCCAGATGCCTACA

CTACATGAAATATTATATCATCAATTGGCTCCTTCATCTCCCTAACTGCC

GTAATTCTTATATTATTTATCATCTGAGAGGCTTTTGCATCTAAACGAGA

AGTCTCTTCCGTTGAATTAACCACAACCAATATCGAATGACTATACGGCT

GTCCCCCTCCTTATCACACATTTGAACAGCCGGTCTTCGTTAAATCTTAA

GA---------CTGGGTACAAGAAAGGGAGGAATTGAACCCCCTAAAATT

GATTTCAAGTCAACTCCATAACCTCTATGACTTTCTC---------ATAA

GATATTAGTAACAATCATTACAAAACTTTGCCATAGTTAAATCATAGGTT

------TAAACCCTATATATCTTATATGCCTTACCCAATAGAACTAGGCT

TTCAAGATGCCACATCCCCTATTATAGAGGAATTAACGTATTTCCATGAT

CATACCTTAATAATCGTTTTCCTAATCAGCTCATTAGTTCTATATATCCT

TCTCTTAATACTTACAACAAAACTAACTCATACCAGTACAATGGATGCCC

AAGAAGTAGAAACAATCTGAACCATTCTACCAGCTGTTATCTTAATCCTA

ATTGCTCTCCCATCCCTTCGCATCCTATACATAATAGACGAAATTTATAA

CCCTTATATAACAGTAAAAGCTATAGGTCATCAATGGTATTGAAGTTATG

AATATACCGATTATGAAGACTTAATATTTGACTCTTATATGATTCCTACC

CGTGACTTAGACCCAGGTCAATTTCGACTACTTGAAGTTGACAATCGAAT

GGTTCTACCAATAGAACTTTCAATCCGTATATTAATTTCATCAGAAGACG

TACTCCATGCATGAGCCGTACCATCCCTGGGGTTGAAAGCTGATGCTATC

CCAGGACGACTTAATCAAGTAACCCTTACATCTACCCGACCGGGTATTTA

CTATGGTCAATGCTCAGAAATTTGTGGCTCCAACCATAGTTTTATACCAA

TTGTTCTAGAGATATCTACGCTAAAATATTTCGAAAAATGGTCTTCTATG

ATGCAATCA-----------------------------------TTTTTG

AGTAGAACCCAAATACCGCTAGTATTTTAAG-------------GAATTA

AAAA-TCCCTCAAAAC-CCATGCCACAATTAGACACTTCAACATGATTTC

TAACTATTACCCTTA-TGATTATTTCCCTTTTCTGCATTTATCAACTAAA

AATAATGAATCAAAATATAATCTCTATTATAGCACAGAATGAAAAAATCA

AACCTACAAAAACAACTTTACCCTGAGAGAAAAAATGAACGAAAATCTAT

TTGCCCCATTCATCACCCCTACTATCCTAGGTATTTCAACACTACCAATC

ATTATATTATTCCCATGCCTAATTTTAACAACCCCAAAACGTTGATTACC

CAACCGAATCCAAATTTTACAAATTTGATTAATTCGCCTAATTACC-AAA

CAAATAATATCAATACACAATCAACGAGGCCGATCATGAACCCTAATACT

TATATCACTTATATTATTTATTGCATCAACAAATCTACTGGGTCTTCTTC

CATATTCTTTCACCCCAACAACACAGCTATCAATAAATATCGGTATAGCT

ATCCCACTCTGAATAGGAACAGTAGTTATAGGCTTCCGAAACAAAC-CCA

AAGTCTCTTTAGCCCACTTCCTCCCTCAAGGAACTCCTACCCCACTGATC

CCTATACTAATTATTATCGAAACAATCAGCCTATTTATTCAACCGCTAGC

CTTAGCAGTCCGACTAACAGCCAATATTACTGCTGGCCACCTTCTTATTC

ACCTCATTGGCTCTGCAACATTAGCCCTGTCATCCATTAACATAACAGTA

TCTACAATTACATTCACTATCCTATTCCTCTTAACAATCTTAGAACTAGC

AGTAGCTATAATCCAAGCCTATGTTTTTACTCTCCTAGTAAGCCTATATT

TGCATGATAATTCATAATGACCCACCAAACCCATGCCTACCACATAGTTA

ACCCCAGTCCATGACCTCTAACAGGGGCCCTCTCAGCCCTCCTAATTACA

TCAGGACTTATTATATGATTCCATTATAACTCTTTATCCCTATTAATCAT

TGGCCTCACTACCATACTATTAACAATATATCAATGATGACGAGATATCG

TTCGAGAAGGGACGTACCAAGGTCATCATACCCCTGTTGTCCAAAAAGGT

TTACGATATGGCATAATTCTTTTCATCCTATCAGAAGTTTTCTTCTTCTT

GGGGTTCTTCTGAGCTTTTTATCATTCTAGCCTAGCTCCAACCCACGAGC

TTGGGGGTTGCTGACCCCCAACGGGTATCCACCCCCTAAACCCACTAGAA

GTCCCACTACTTAATACATCTATCCTCCTTGCTTCCGGAGTATCAATTAC

ATGAGCCCATCATAGCCTTATAGAAGGAAACCGCAAACAAATGATTCAAG

CACTAGGCATTACTATTATTCTAGGAATGTATTTTACAGCCCTACAAGCC

ATAGAATATTATGAAGCCTCCTTTACTATTTCAGATGGAGTATATGGCTC

AACCTTCTTTGTAGCTACAGGATTTCATGGACTACATGTCATTATTGGCT

CTACTTTCCTAATTGTCTGTTTAATCCGACAATTTCATTATCACTTCACA

TCTACTCATCATTTTGGCTTTGAGGCAGCAGCCTGATACTGACACTTTGT

AGACGTAGTATGATTATTCCTTTATGTTTCTATCTACTGATGAGGTTCAT

ATTTT-TCTAGTAT--AATTAGTACTACTGATTTCCAATCATTAAGTTCT

GGGTA-AA-CCAGAGAAAAATAATTAATCTTATTATTATCTTGCTAGTAA

ACACTTTATTAGCCTCTATCGTTGTTTTAATTGCCTTTTGACTGCCTCAA

ATATATTTATACCTAGAGAAATCAAGCCCATACGAGTGCGGATTTGACCC

CCTGGGATCCGCTCGCCTGCCATTTTCAATAAAATTTTTTCTGGTCGCCA

TTACATTTCTCTTATTTGATCTAGAAATCGCCTTACTCTTGCCCTTGCCC

TGAGCAATCCAATTACCTAATCCAAAAGTCACACTAGTCTTCTCATATGG

TTTAATTTTACTACTAACAGCAGGTCTGGCTTATGAATGATTCCAAAAAG

GCCTAGAATGAACCGAATA-------GGTTTTTAATCTAA--TTAAGATA

ATTGATTTCGACTCAATAAATCATGGTTTAAACCCATGACTACC---TTA

TAATATCAATCAATTTAAACCTAATTATAGCCTTCTCATTAGCCCTACTA

GGGGTCCTCATTTACCGATCACACCTTATATCAACACTTCTATGTTTAGA

AGGAATGATGCTATCCCTATTTGTCCTTATAGCACTTCTAATTTCTCATT

TCCACATAATTTCTTTATCAATACTACCCCTAATCCTTTTAGTCTTTTCA

GCATGCGAGGCAGGCATTGGATTAGCCCTACTAGTCAAAACTTCTACCAA

CTATGGCAACGATTACATTCAAAACCTAAATCTACTACAATGCTAAAAAT

CTTAATCCCAACCTCAATGCTCATCCCCCTAACCTGATACTCCAAAAAAC

AGTGAGTATGAATTAATCCCACCATATATAGCCTTATTATTAGTTCTGCT

AGCTTACCTCTCTTATACCACAACTCAGACCTAGGTTACAATTACAACTC

CTCCTTCTCTATAGATTCCCTATCAAGCCCCTTACTAGTTCTATCTTGCT

GATTACTACCATTAATAATAATTGCCAGCCAAAGCCATTTAACCAATGAA

CCATTACTCCGAAAAAAAGTATACCTAACTATATTAATTATCCTTCAACT

ATCCCTAATTGCAGCCTTTACATCGTCAGAACTTATCATATTTTACATTT

TATTCGAAACAACACTCATCCCCACCCTTATCGTAATCACACGATGAGGA

AACCAAAATGAACGACTAAACGCTGGCTTATATTTTCTCTTTTATACCCT

AGCAGGATCACTACCATTACTAGTCGCTCTATTACTCATATACTATAACC

TTGGATCCCTACATATCTTAACTATATCCCTATTCTCCCCAGCTTTAAAC

TTTTCC----------CCCTCAGGCTCTATTCTATGATACGCATGCATAA

TTGCATTTATAGTCAAAATACCTCTATATGGCCTCCACCTATGACTTCCC

AAAGCCCACGTTGAAGCCCCAATCGCAGGCTCTATAGTCTTAGCCGCTAT

TCTCCTGAAACTTGGAGGTTATGGCATCATACGAATTACAATTTTTACCC

ACCCAGTCACTACTAACCTAGCTTATCCATTCATCATTCTATCTATATGA

GGCATAATCATAACAAGCTCTATCTGCTTACGCCAGACAGACTTAAAATC

ACTCATCGCTTATTCCTCTGTCAGCCACATAGGTCTAGTTATTATTGCTG

CTCTCATACAATCCACACTAAGCTTTATAGGAGCTACAACCCTGATAATT

GCTCATGGCCTCACCTCCTCTATATTATTCTGCCTAGCTAACACTAATTA

TGAACGTATCCACAGCCGAACAATAATTCTCACTCGAGGCCTTCAAATAA

TTCTTCCCCTTATATGTGCCTGATGACTATTAGCTAGTTTAGCTAATTTA

GCCCTACCCCCAACCATTAACCTGCTAGGAGAACTTATAGTCATCATTTC

ATCCTTCTCATGATCTAACTTCTCCATAATCTTACTAGGAATCAACACAG

TAATTACTGCACTCTACTCTTTACATATATTAATTACCTCACAACGAGGT

AAATTTACACACCACATAAATCCTATCAAACCCTCCATAACCCGCGAACA

CATCCTTATAACCCTTCACCTTATTCCCCAACTCATCATCTCCTTAAGCC

CCAAATTCATTTTGGGTCTTACATACTGCAAATATAGTTTAATAAAAACA

TTAGATTGTGAATCTAAACATAGAAGTT--TGAACCTTCTTATATGCCGA

GAAAGTCC----AAAGAACTGCTAACTCTTTAACCCGCATTTAACACATG

CGGCTT--------------------------------------------

-------------------TCTTA--CTTTTAAAGGATAATAGTA-ATCC

ATTGGTCTTAGGAACCAAAAACT-TTGGTGCAACTCCAAATAAAAGTAAT

TAATATATTAATTAATACACTCATTTTACTATCCATTAGCATTTTAATCT

TTCCACTAATCATTAATATTGTTACTCCCCATAAAACCCAACACTTCCCC

TTATACTGTAAAAATGCAGTAAAACTAGCCTTCTTTACTAGCCTACCTCC

AACCCTCATATTCATTTACTCGGGCCAAGAATCAACAATTACTAATTGAC

AATGATTCTCAATTAGCTCATTTAACATTTCTATAAGCTTTAAACTAGAC

TATTTCGCTCTTATCTTCATTCCAATCGCACTATATGTAACCTGATCTAT

CCTAGAATTTTCCCTATGATACATACATTCAGACCCTAACATCCACCGAT

TCTTTAAATACCTAATCACATTTCTATTTACCATAATCATCCTCGTATCA

GCCAATAACCTCTTCCAACTATTTATTGGCTGAGAAGGCGTAGGCATTAT

ATCATTTATACTGATCGGATGATGATACGGCCGAACCGATGCCAACACAG

CAGCTCTTCAAGCTGTCCTTTATAACCGCATTGGAGATATTGGATTTATG

CTAACAATAGCATGATTAATAATTCACAGTAACTCATGAGACCTTCAACA

AATTTTCATAACGGATATGAATACA------CTAGCCCTCCTAGGACTAA

TCATTGCCGCAACAGGCAAATCAGCCCAATTCGGTCTTCACCCATGACTA

CCTTCAGCCATAGAAGGTCCAACTCCAGTCTCAGCCCTTCTTCACTCAAG

CACTATAGTAGTTGCAGGGATTTTCCTACTCATTCGCTTCCATCCTATAT

TA-CAAAATAATAATATAACCCTCACCACAGCACTATGCCTGGGAGCAAT

TACTACTCTATTTACTGCAATCTGCGCAATCACCCAAAACGATATTAAAA

AAATCGTAGCATTCTCTACCTCTAGTCAACTAGGTCTCATAATAGTAACT

ATTGGCTTAAACCAGCCTCACTTGGCATTCCTCCATATTTGTACCCACGC

ATTCTTCAAAGCAATACTCTTTCTATGCTCCGGCTCTATTATCCACAGCC

TCAATGACGAGCAAGACATTCGCAAAATAGGGGGTCTCTTAACCACACTT

CCTATTACTTCATCTGCCTTAATAACAGGAAGCCTAGCACTTATTGGCAC

CCCATTCTTAGCAGGATTCTACTCCAAAGATTCTATTATTGAAGCAATAA

ACTCATCCTATGTTAACCTATGAGCACTTCTTATTACAATAATTGCTACA

GCTCTAACAGCCGTATACAGCCTGCGAATCGTATACTTTGCCTTACTCAA

CCAACCCCGATTCCCTCCACTCTCCCCACTAAATGAAAATAACCCCAGCC

TAATTAATCCTATTATTCGACTAGCCCTAGGCAGTATTTTCGCTGGCTTC

CTCCTAACTATAAATATTCCACCTACATCCATAATTCCACTTACTATGCC

TACTATAACTAAACTATCAGCATTAATCGTCACTATCGCAGGCTTATTCA

TTGCCATGGAACTAAACAAATTAACTAACTCTTCACCTATACTAAATATA

ATCCATAC-TCATAACTTCTCAAACATGTTAGGATACTTTACACATCTAT

TTCACCGTATTTACCCCCTAACAAACTTAAAATTAGGTCAACATATCGCT

ACCATACTAATTGATCTTAACTGATATGAAAAAACAGGCCCAAAAGGCCA

AGCTGATCTCCATACCATCGCATCTTCATCTATAACTTCTGCCCATAAAG

GACTAATCAAAATATACTTCATATCGTTCATCATCTCTATCTTAA---TT

ATCACAACAGTTATCTAAT-T----------------------ACAACCA

CGAACGACTTCTAAAACAATATAAATCGTAATAAAAAGAATCCAGCCTAA

CAGAACTAATGCTCATCCTCCACACCCGTACAATAAAGAAACTCCACTAT

AATCTTGCCCTACGCAGTAGTTACCAATAGTATCAAATAAC---------

---------TCAACTGCAGTAACTAATTCAA------------CTTCACC

AGACATAAAGTATCACAATA------ACTCTATCACCAGCGCAAATAATA

ATATACTCAACGCCACCGAATTACCAATTCAACTCTCAGGGTACTCCTCA

GTAGCCATTGCAGCCGTATAACCAAATACTACAAGCATGCCACCAAGATA

AACTAAAAAAACTACTAAACCTAAAAAGACATCTTCTAAACTAACCACAA

TAGCACATCCCAAACCTCCACTCACTACTAAACTTAAACCACCATATACC

GGTGAAGGTTTAGAAGCAAAAGCTACAAACCCAAAGATAAATAGGAGAGA

AAGCAAAAAAATGAGTATTATTTTCATTAATTTTAGTATGGACTCTA-AC

CATAACCTATGGCATGAAAAACCACCGTTGTTC-TTCAACTACAAAAAC-

------TAATGACCAACCTACGTAAAACCCACCCACTAATAAAAATCGTC

AACGACTCCTTCATTGACTTACCAGCACCCTCCAATATCTCTGCCTGATG

AAATTTCGGCTCATTACTAGGCACCTGCCTTGTAATCCAAATCCTAACAG

GCCTATTCTTAGCTATACACTACACCTCAGACACTTTAACCGCCTTCTCA

TCAGTAGCCCACATCTGTCGAGATGTAAATTACGGTTGACTAATTCGAAA

TCTCCACGCCAACGGAGCCTCAATATTCTTCATATGCTTATTCCTCCACG

TAGGACGAGGTATCTACTACGGCTCATTCTTATTTAAAGAAACATGAAAT

ATCGGTGTTATCCTTCTACTAACCGTTATAGCTACCGCCTTCGTAGGATA

TGTTCTCCCATGAGGACAAATATCCTTCTGAGGAGCCACAGTTATTACTA

ATCTCCTCTCCGCTATCCCATATATCGGAACTACCCTCGTAGAATGAATT

TGAGGGGGGTTTTCAGTAGATAAAGCAACCCTCACACGATTCTTTGCTTT

CCACTTCATCCTACCATTCATTGTTATAGCCCTGACTATTGTTCACCTCC

TATTCCTCCACGAAACTGGCTCAAACAACCCCTCAGGAGTAAACCCAGAC

TCAGATAAAATTCCATTCCACCCCTACTACACAATTAAAGACGCACTAGG

ATTCATCTTAATGCTCCTCGTACTTATACTTTTAGCCCTATTTTCTCCAG

ACCTACTGGGAGACCCAGATAACTTCTCTCCTGCCAATCCCCTCAACACA

CCCCCTCATATCAAACCAGAATGATATTTCCTCTTCGCTTACGCAATCCT

GCGATCAATTCCCAACAAACTAGGAGGTGTCCTAGCCCTACTTGCATCCA

TCCTAGTACTTCTCATTATTCCTCTTCTCCATACATCCAATCAACGTAGC

CTGATATTCCGACCAATCTCTCAAGCTCTTTTTTGACTCTTAACCGCTAA

CCTATTCACCCTTACCTGAATTGGGGGCCAACCAGTTGAACAACCTTACA

TTATCATTGGTCAACTAGCATCCATTCTATACTTCCTACTCATTCTTATC

CTAATACCCCTAGCAGGTTTATTCGAAAATTATTTACTAAAACCTAAATG

AAGACGTCCAAGTAATTTAACAAAAATATTGGCCTTGTAAGCCAACAACG

AAG-----------------------------------------------

--------------------------------------------------

--------------------------------------------------

--------------------------------------------------

--------------------------------------------------

--------------------------------------------------

--------------------------------------------------

--------------------------------------------------

--------------------------------------------------

--------------------------------------------------

--------------------------------------------------

--------------------------------------------------

--------------------------------------------------

--------------------------------------------------

--------------------------------------------------

--------------------------------------------------

--------------------------------------------------

--------------------------------------------------

--------------------------------------------------

--------------------------------------------------

--------------------------------------------------

--------------------------------------------------

--------------------------------------------------

--------------------------------------------------

--------------------------------------------------

------

>Homo_sapiens_

GTTTATGTAGCTTA--CCT----CCTCAAAGCAATACACTGAAAATGTTT

AGACGGGC-TCACATCACCCCATAAACAAATA--GGTTTGGTCCTAGCCT

TTCTATTAGCTCTTAGTAAGATTACACATGCAAGCATCCCCGTTCCAGTG

AGTTCACCCT-CTAAATCACCA------CGATCAAAAGGGACAAGCATCA

AGCACGCAGCAAT---GCAGCTCAAAACGCTTAGC-CTAGCCACACCCCC

ACGGGAAACAGCAGTGATTAACCTTTAGCA-ATAAACGAAAGTTTAACTA

AGCTATACTA--ACCCCAGGGTTGGTCAATTTCGTGCCAGCCACCGCGGT

CACACGATTAACCCAAGTCAATAGAA--GCCGGCGTAAAGAGTGTTTTAG

AT---CACCCCCTCCCCAATAAAGCTAAAACTCACCTGAGTTGTAAAAAA

CTCCAGTTGACA--CAAAATAGACTACGAAAGTGGCTTTA---ACATATC

TGAACACACAATAGCTAAGACCCAAACTGGGATTAGATACCCCACTATGC

TTAGCCCTAAACCTCAACAGTTAAATC-AACAAAACTGCTCGCCAGAACA

CTACGAGCCACAGCTTAAAACTCAAAGGACCTGGCGGTGCTTCATATCCC

TCTAGAGGAGCCTGTTCTGTAATCGATAAACCCCGATCAACCTCACCACC

TCTTGC-----TCAGCCTATATACCGCCATCTTCAGCAAACCCTGATGAA

GGCTACAAAGTAAGCGCAAGTACCCA---CGTAAAGACGTTA-GGTCAAG

GTGTAGCCCATGAGGTGGC-AAGAAATGGGCTACATTTTCTACCCC----

--AGA------AAACT--ACGATAGCCCTTATGAAACTTAA--GGGTCGA

AGGTGGATTTAGCAGTAAACT-GAGAATAGAGTGCTTAGTTGAACAG-GG

CCCTGAAGCGCGTACACACCGCCCGTCACCCTCCTCAAGTATACTTCAAA

GGACATTTAACTAAAA--------CCCCTACGCAT--TTATA-TAGAGGA

GACAAGTCGTAACATGGTAAGTGTACTGGAAAGTGCACTTGGACGAACCA

GAGTGTAGCTTA----ACACAAAGCACCCAACTTACACTTAGGAGATTTC

AACTTAACTTGACCGCTCTGA-GCT-AAACCT-AGCCCCAAACCC-----

ACTCCACCTTACTACCAGAC-AACCTTAGCCAAACCATTTACC-CAAA--

---TAAAGTATAGGCGATAGAAATTGAA-ACCT---GGCGCAATAGATAT

AGTACCGCAAGGGAAAGA-TGAAAAATTATAACCA-AGCATAATATAGCA

AGGACTAACCCCTATACCTTCTGCATAA-TGAATTAACTAGAAATAACTT

TGCAAGGAGAGCCA-AAGCTAAGACCCCCGAAACCAGACGAGCTACCTAA

GAACAGCTA-A--AAGAGCACACCCGTCTATGTAGCAAAATAGTGGGAAG

ATTTATAGGTAGAGGCGACAAACCTACCGAGCCTGGTGATAGCTGGTTGT

CCAAGAT-AGAATCTTAGTTCAACTTTAAATTTGCCCACAGAACC-CTCT

AAATCCCCTTGTAAATTTAACTGTTAGTCCAAAGAGGAACAGCTCTTTGG

ACACTAGGAAAAAACCTTGTAGAGAGAGTAA-----AAAATTTAACA--C

CCATAGTAGGCCTAAAAGCAGCCACCAATTAAGAAAGCGTTCAAGCTCAA

C------ACCCACTACCTAAAAAATCCCAAACAT---ATAACTGAACT--

CCTCACACC-CAATTGGACCAATCTATCATCCTAT-AGAAGAACTAATGT

TAGTATAAGTAACATGAA-AACAT-TCTCCTCCGCATAAGCCTGCGTCAG

ATTAAAACACTGAACTGACAATTAACAGCCCAATA------TCTACAATC

AACCAACAAGTCATTATTACCCTCACTGTCAACCCAACACAGGCATGC--

-TCATAAGGAAAGGTTAAAAAAAGTAAAAGGAACTCGGCAAATCTTA-CC

CCGCCTGTTTACCAAAAACATCACCTCTAGCATCACCAGTATTAGAGGCA

CCGCCTGCCCAGTGACA-CATG----TTTAACGGCCGCGGTACCCTAACC

GTGCAAAGGTAGCATAATCACTTGTTCCTTAAATAGGGACCTGTATGAAT

GGCTCCACGAGGGTTCAGCTGTCTCTTACTTTTAACCAGTGAAATTGACC

TGCCCGTGAAGAGGCGGGCATGACACAGCAAGACGAGAAGACCCTATGGA

GCTTTAATTTATTAATGCAAACAGTACCTA-ACAAACCC---------AC

AGGTCCT-AAACTACCAA-ACCTGCATTAAAAATTTCGGTTGGGGCGACC

TCGGAGCAGAACCCAACCTCCGAGCAGT--ACATGCTAAGACTTCACCAG

TCAAAGCGA-ACTACTATACTCAATTGATCCAA----TAACTTGACCAAC

GGAACAAGTTACCCTAGGGATAACAGCGCAATCCTATTCTAGAGTCCATA

TCAACAAT-AGGGTTTACGACCTCGATGTTGGATCAGGACATCCCGATGG

TGCAGCCGCTATTAAAG-GTTCGTTTGTTCAACGATTAA-AGTCCTACGT

GATCTGAGTTCAGACCGGAGTAATCCAGGTCGGTTTCTATCTANCTTCA-

AATTCCTCCCTGTACGAAAGGACAAGAGAAATAAGGCCTACT--TCACAA

AGCGCCTTC-CCC-CGTAAATGA-TATCATCTCAACTTAGTATTATACC-

CACAC-----CCACCCAAGAACAGGG--TTT-GTTAAGATGGCAGAGCCC

GGTAA-TCGCATAAAACTTAAAACTTTACAGTCAGAGGTTCAATTCCTCT

TCTTAACAAC----------------------------------------

------------------------------------------ATACCCAT

GGCCAACCTCCTACTCCTCATTGTACCCATTCTAATCGCAATGGCATTCC

TAATGCTTACCGAACGAAAAATTCTAGGCTATATACAACTACGCAAAGGC

CCCAACGTTGTAGGCCCCTACGGGCTACTACAACCCTTCGCTGACGCCAT

AAAACTCTTCACCAAAGAGCCCCTAAAACCCGCCACATCTACCATCACCC

TCTACATCACCGCCCCGACCTTAGCTCTCACCATCGCTCTTCTACTATGA

ACCCCCCTCCCCATACCCAACCCCCTGGTCAACCTCAACCTAGGCCTCCT

ATTTATTCTAGCCACCTCTAGCCTAGCCGTTTACTCAATCCTCTGATCAG

GGTGAGCATCAAACTCAAACTACGCCCTGATCGGCGCACTGCGAGCAGTA

GCCCAAACAATCTCATATGAAGTCACCCTAGCCATCATTCTACTATCAAC

ATTACTAATAAGTGGCTCCTTTAACCTCTCCACCCTTATCACAACACAAG

AATACCTCTGATTACTCCTGCCATCATGACCCTTGGCCATAATATGATTT

ATCTCCACACTAGCAGAGACCAACCGAACCCCCTTCGACCTTGCCGAAGG

GGAGTCCGAACTAGTCTCAGGCTTCAACATCGAATACGCCGCAGGCCCCT

TCGCCCTATTCTTCATAGCCGAATACACAAACATTATTATAATAAACACC

CTCACCACTACAATCTTCCTAGGAACAACATATGACGCACTCTCCCCTGA

ACTCTACACAACATATTTTGTCACCAAGACCCTACTTCTAACCTCCCTGT

TCTTATGAATTCGAACAGCATACCCCCGATTCCGCTACGACCAACTCATA

CACCTCCTATGAAAAAACTTCCTACCACTCACCCTAGCATTACTTATATG

ATATGTCTCCATACCCATTACAATCTCCAGCATTCCCCCTCAAACCTAA-

GAAATATGTCTGATAAAAGAGTTACTTTGATAGAGTAAATAATAGGAGCT

TA-AACCCCCTTATTTCTAGGACTATGAGAATCGAACCCATCCCTGAGAA

TCCAAAATTCTCCGTGCCACC--TATCACACCCCATCCTAA--------A

GTAAGGTCAGCTAAAT-AAGCTATCGGGCCCATACCCCGAAAATGTTGGT

T-ATACCCTTCCCGTACTAATTAATCCCCTGGCCCAACCCGTCATCTACT

CTACCATCTTTGCAGGCACACTCATCACAGCGCTAAGCTCGCACTGATTT

TTTACCTGAGTAGGCCTAGAAATAAACATGCTAGCTTTTATTCCAGTTCT

AACCAAAAAAATAAACCCTCGTTCCACAGAAGCTGCCATCAAGTATTTCC

TCACGCAAGCAACCGCATCCATAATCCTTCTAATAGCTATCCTCTTCAAC

AATATACTCTCCGGACAATGAACCATAACCAATACTACCAATCAATACTC

ATCATTAATAATCATAATGGCTATAGCAATAAAACTAGGAATAGCCCCCT

TTCACTTCTGAGTCCCAGAGGTTACCCAAGGCACCCCTCTGACATCCGGC

CTGCTTCTTCTCACATGACAAAAACTAGCCCCCATCTCAATCATATACCA

AATCTCTCCCTCACTAAACGTAAGCCTTCTCCTCACTCTCTCAATCTTAT

CCATCATAGCAGGCAGTTGAGGTGGATTAAACCAAACCCAGCTACGCAAA

ATCTTAGCATACTCCTCAATTACCCACATAGGATGAATAATAGCAGTTCT

ACCGTACAACCCTAACATAACCATTCTTAATTTAACTATTTATATTATCC

TAACTACTACCGCATTCCTACTACTCAACTTAAACTCCAGCACCACGACC

CTACTACTATCTCGCACCTGAAACAAGCTAACATGACTAACACCCTTAAT

TCCATCCACCCTCCTCTCCCTAGGAGGCCTGCCCCCGCTAACCGGCTTTT

TGCCCAAATGGGCCATTATCGAAGAATTCACAAAAAACAATAGCCTCATC

ATCCCCACCATCATAGCCACCATCACCCTCCTTAACCTCTACTTCTACCT

ACGCCTAATCTACTCCACCTCAATCACACTACTCCCCATATCTAACAACG

TAAAAATAAAATGACAGTTTGAACATACAAAACCCACCCCATTCCTCCCC

ACACTCATCGCCCTTACCACGCTACTCCTACCTATCTCCCCTTTTATACT

AATAATCTT---AT----AGAAATTTAGGTTAAA--TACAGACCAAGAGC

CTTCAAAGCCCTCAGTAAGT--TGCAAT---ACTTAATTTCTGT------

---AACAGCTAAGGACTGCAAAACCCCACTCTGCAT----CAACTGAACG

CAAATCAGCCACTTTAA--TT-AAGCTAAGCCCTTA-----------CTA

GACCAATG--GGACTTAAA-CCCAC-AAACACTTAGTTAACAGCTAAGCA

CCCTAATCAACTGGCTTCAATCTA-CT-TCTCCCGCCGCCGGGA-AAAAA

-----GGCGGGAGAAGCCCCGGCAGG---TTTGAAGCTGCTTCTTC--GA

-----------------------------ATTTGCAATTCAATA--TGAA

-A-ATCACCTCGGAGCT-------GGTAAAAAGAGGC----CTA-A-CCC

CTGTCTTTAGATTTACAGTCCAATGCTT-CACTCAGCCATTTTACCTCAC

CCCCACTGATGTTCGCCGACCGTTGACTATTCTCTACAAACCACAAAGAC

ATTGGAACACTATACCTATTATTCGGCGCATGAGCTGGAGTCCTAGGCAC

AGCTCTAAGCCTCCTTATTCGAGCCGAGCTGGGCCAGCCAGGCAACCTTC

TAGGTAACGACCACATCTACAACGTTATCGTCACAGCCCATGCATTTGTA

ATAATCTTCTTCATAGTAATACCCATCATAATCGGAGGCTTTGGCAACTG

ACTAGTTCCCCTAATAATCGGTGCCCCCGATATGGCGTTTCCCCGCATAA

ACAACATAAGCTTCTGACTCTTACCTCCCTCTCTCCTACTCCTGCTCGCA

TCCGCTATAGTGGAGGCCGGAGCAGGAACAGGTTGAACAGTCTACCCTCC

CTTAGCAGGGAACTACTCCCACCCTGGAGCCTCCGTAGACCTAACCATCT

TCTCCTTACACCTAGCAGGTGTCTCCTCTATCTTAGGGGCCATCAATTTC

ATCACAACAATTATCAATATAAAACCCCCTGCCATAACCCAATACCAAAC

GCCCCTCTTCGTCTGATCCGTCCTAATCACAGCAGTCCTACTTCTCCTAT

CTCTCCCAGTCCTAGCTGCTGGCATCACTATACTACTAACAGACCGCAAC

CTCAACACCACCTTCTTCGACCCCGCCGGAGGAGGAGACCCCATTCTATA

CCAACACCTATTCTGATTTTTCGGTCACCCTGAAGTTTATATTCTTATCC

TACCAGGCTTCGGAATAATCTCCCATATTGTAACTTACTACTCCGGAAAA

AAAGAACCATTTGGATACATAGGTATGGTCTGAGCTATGATATCAATTGG

CTTCCTAGGGTTTATCGTGTGAGCACACCATATATTTACAGTAGGAATAG

ACGTAGACACACGAGCATATTTCACCTCCGCTACCATAATCATCGCTATC

CCCACCGGCGTCAAAGTATTTAGCTGACTCGCCACACTCCACGGAAGCAA

TATGAAATGATCTGCTGCAGTGCTCTGAGCCCTAGGATTCATCTTTCTTT

TCACCGTAGGTGGCCTGACTGGCATTGTATTAGCAAACTCATCACTAGAC

ATCGTACTACACGACACGTACTACGTTGTAGCTCACTTCCACTATGTCCT

ATCAATAGGAGCTGTATTTGCCATCATAGGAGGCTTCATTCACTGATTTC

CCCTATTCTCAGGCTACACCCTAGACCAAACCTACGCCAAAATCCATTTC

ACTATCATATTCATCGGCGTAAATCTAACTTTCTTCCCACAACACTTTCT

CGGCCTATCCGGAATGCCCCGACGTTACTCGGACTACCCCGATGCATACA

CCACATGAAACATCCTATCATCTGTAGGCTCATTCATTTCTCTAACAGCA

GTAATATTAATAATCTTCATGATTTGAGAAGCCTTCGCTTCGAAGCGAAA

AGTCCTAATAGTAGAAGAACCCTCCATAAACCTGGAGTGACTATATGGAT

GCCCCCCACCCTACCACACATTCGAAGAACCCGTATACATAAAATC----

-TAGA-------------CAAAAAAGGAAGGAATCGAACCCCCCAAAGCT

GGTTTCAAGCCAACCCCATGGCCTCCATGACTTTTTC--------AAAAA

GGTATTAGAAAAACCA-TTTCATAACTTTGTCAAAGTTAAATTATAGGCT

-------AAATCCTATATATCTTA-ATGGCACATGCAGCGCAAGTAGGTC

TACAAGACGCTACTTCCCCTATCATAGAAGAGCTTATCACCTTTCATGAT

CACGCCCTCATAATCATTTTCCTTATCTGCTTCCTAGTCCTGTATGCCCT

CTTCCTAACACTCACAACAAAACTAACTAATACTAACATCTCAGACGCTC

AGGAAATAGAAACCGTCTGAACTATCCTGCCCGCCATCATCCTAGTCCTC

ATCGCCCTCCCATCCCTACGCATCCTTTACATAACAGACGAGGTCAACGA

TCCCTCCCTCACCATCAAATCAATTGGCCACCAATGGTACTGAACCTACG

AGTACACCGACTACGGCGGACTAATCTTCAACTCCTACATACTTCCCCCA

TTATTCCTAGAACCAGGCGACCTGCGACTCCTTGACGTTGACAATCGAGT

AGTACTCCCGATTGAAGCCCCCATTCGTATAATAATTACATCACAAGACG

TCTTGCACTCATGAGCTGTCCCCACATTAGGCTTAAAAACAGATGCAATT

CCCGGACGTCTAAACCAAACCACTTTCACCGCTACACGACCGGGGGTATA

CTACGGTCAATGCTCTGAAATCTGTGGAGCAAACCACAGTTTCATGCCCA

TCGTCCTAGAATTAATTCCCCTAAAAATCTTTGAAATAGGGCCCGTATTT

ACCCTATAG-----------CACCCCCTCTACCCCCTCTAGAGCCCACTG

TAAAGCTA--ACTTAGCATTAACCTTTTAAGTTAAAGATTAAGAGAACCA

ACAC-CTCTTTACAGTGAAATGCCCCAACTAAATACTACCGTATGGCCCA

CCATAATTACCCCCA-TACTCCTTACACTATTCCTCATCACCCAACTAAA

AATATTAAA-CA--CAAACTACCACCTACCTCCCTCACCAAAGCCCATAA

AAATAAAAAATTATAACAAACCCTGAGAACCAAAATGAACGAAAATCTGT

TCGCTTCATTCATTACCCCCACAATCCTAGGCCTACCCGCCGCAGTACTG

ATCATTCTATTTCCCCCTCTATTGATCCCCACCTCCAAATATCTCATCAA

CAACCGACTAATCACCACCCAACAATGACTAATCAAACTAAC-CTCAAAA

CAAATGATAACCATACACAACACTAAAGGACGAACCTGATCTCTTATACT

AGTATCCTTAATCATTTTTATTGCCACAACTAACCTCCTCGGACTCCTGC

CTCACTCATTTACACCAACCACCCAACTATCTATAAACCTAGCCATGGCC

ATCCCCTTATGAGCGGGCGCAGTGATTATAGGCTTTCGCTCTAAGA-TTA

AAAATGCCCTAGCCCACTTCTTACCACAAGGCACACCTACACCCCTTATC

CCCATACTAGTTATTATCGAAACCATCAGCCTACTCATTCAACCAATAGC

CCTGGCCGTACGCCTAACCGCTAACATTACTGCAGGCCACCTACTCATGC

ACCTAATTGGGAGCGCCACCCTAGCAATATCAACCATTAACCTTCCCTCT

ACACTTATCATCTTCACAACTCTAATTCTACTGACTATCCTAGAAATCGC

TGTCGCCTTAATCCAAGCCTACGTTTTCACACTTCTAGTAAGCCTCTACC

TGCACGACAACACATAATGACCCACCAATCACATGCCTATCATATAGTAA

AACCCAGCCCATGACCCCTAACAGGGGCCCTCTCAGCCCTCCTAATGACC

TCCGGCCTAGCCATGTGATTTCACTTCCACTCCATAACGCTCCTCATACT

AGGCCTACTAACCAACACACTAACCATATACCAATGATGGCGCGATGTAA

CACGAGAAAGCACATACCAAGGCCACCACACACCACCTGTCCAAAAAGGC

CTTCGATACGGGATAATCCTATTTATTACCTCAGAAGTTTTTTTCTTCGC

AGGATTTTTCTGAGCCTTTTACCACTCCAGCCTAGCCCCTACCCCCCAAC

TAGGAGGGCACTGGCCCCCAACAGGCATCACCCCGCTAAATCCCCTAGAA

GTCCCACTCCTAAACACATCCGTATTACTCGCATCAGGAGTATCAATCAC

CTGAGCTCACCATAGTCTAATAGAAAACAACCGAAACCAAATAATTCAAG

CACTGCTTATTACAATTTTACTGGGTCTCTATTTTACCCTCCTACAAGCC

TCAGAGTACTTCGAGTCTCCCTTCACCATTTCCGACGGCATCTACGGCTC

AACATTTTTTGTAGCCACAGGCTTCCACGGACTTCACGTCATTATTGGCT

CAACTTTCCTCACTATCTGCTTCATCCGCCAACTAATATTTCACTTTACA

TCCAAACATCACTTTGGCTTCGAAGCCGCCGCCTGATACTGGCATTTTGT

AGATGTGGTTTGACTATTTCTGTATGTCTCCATCTATTGATGAGGGTCTT

ACTCT-TTTAGTAT-AA-ATAGTACCGTTAACTTCCAATTAACTAGTTTT

GACAAC-ATTCAAAAAAGAGTAATAAACTTCGCCTTAATTTTAATAATCA

ACACCCTCCTAGCCTTACTACTAATAATTATTACATTTTGACTACCACAA

CTCAACGGCTACATAGAAAAATCCACCCCTTACGAGTGCGGCTTCGACCC

TATATCCCCCGCCCGCGTCCCTTTCTCCATAAAATTCTTCTTAGTAGCTA

TTACCTTCTTATTATTTGATCTAGAAATTGCCCTCCTTTTACCCCTACCA

TGAGCCCTACAAACAACTAACCTGCCACTAATAGTTATGTCATCCCTCTT

ATTAATCATCATCCTAGCCCTAAGTCTGGCCTATGAGTGACTACAAAAAG

GATTAGACTGAACCGAAT-------TGGTATATAGTTTAAAC--AAAACG

AATGATTTCGACTCATTAAATTATGATAA---T-CATATTTACCAA---A

TGCCCCTCATTTACATAAATATTATACTAGCATTTACCATCTCACTTCTA

GGAATACTAGTATATCGCTCACACCTCATATCCTCCCTACTATGCCTAGA

AGGAATAATACTATCGCTGTTCATTATAGCTACTCTCATAACCCTCAACA

CCCACTCCCTCTTAGCCAATATTGTGCCTATTGCCATACTAGTCTTTGCC

GCCTGCGAAGCAGCGGTGGGCCTAGCCCTACTAGTCTCAATCTCCAACAC

ATATGGCCTAGACTACGTACATAACCTAAACCTACTCCAATGCTAAAACT

AATCGTCCCAACAATTATATTACTACCACTGACATGACTTTCCAAAAAAC

ACATAATTTGAATCAACACAACCACCCACAGCCTAATTATTAGCATCATC

CCTCTACTATTTTTTAACCAAATCAACAACAACCTATTTAGCTGTTCCCC

AACCTTTTCCTCCGACCCCCTAACAACCCCCCTCCTAATACTAACTACCT

GACTCCTACCCCTCACAATCATGGCAAGCCAACGCCACTTATCCAGTGAA

CCACTATCACGAAAAAAACTCTACCTCTCTATACTAATCTCCCTACAAAT

CTCCTTAATTATAACATTCACAGCCACAGAACTAATCATATTTTATATCT

TCTTCGAAACCACACTTATCCCCACCTTGGCTATCATCACCCGATGAGGC

AACCAGCCAGAACGCCTGAACGCAGGCACATACTTCCTATTCTACACCCT

AGTAGGCTCCCTTCCCCTACTCATCGCACTAATTTACACTCACAACACCC

TAGGCTCACTAAACATTCTACTACTCACTCTCACTGCCCAAGAACTATCA

AACTCC----------TGAGCCAACAACTTAATATGACTAGCTTACACAA

TAGCTTTTATAGTAAAGATGCCTCTTTACGGACTCCACTTATGACTCCCT

AAAGCCCATGTCGAAGCCCCCATCGCTGGGTCAATAGTACTTGCCGCAGT

ACTCTTAAAACTAGGCGGCTATGGTATAATACGCCTCACACTCATTCTCA

ACCCCCTGACAAAACACATAGCCTACCCCTTCCTTGTACTATCCCTATGA

GGCATAATTATAACAAGCTCCATCTGCCTACGACAAACAGACCTAAAATC

GCTCATTGCATACTCTTCAATCAGCCACATAGCCCTCGTAGTAACAGCCA

TTCTCATCCAAACCCCCTGAAGCTTCACCGGCGCAGTCATTCTCATAATC

GCCCACGGACTTACATCCTCATTACTATTCTGCCTAGCAAACTCAAACTA

CGAACGCACTCACAGTCGCATCATAATCCTCTCTCAAGGACTTCAAACTC

TACTCCCACTAATAGCTTTTTGATGACTTCTAGCAAGCCTCGCTAACCTC

GCCTTACCCCCCACTATTAACCTACTGGGAGAACTCTCTGTGCTAGTAAC

CACGTTCTCCTGATCAAATATCACTCTCCTACTTACAGGACTCAACATAC

TAGTCACAGCCCTATACTCCCTCTACATATTTACCACAACACAATGGGGC

TCACTCACCCACCACATTAACAACATAAAACCCTCATTCACACGAGAAAA

CACCCTCATGTTCATACACCTATCCCCCATTCTCCTCCTATCCCTCAACC

CCGACATCATTACCGGGTTTTCCTCTTGTAAATATAGTTTAACCAAAACA

TCAGATTGTGAATCTGACAACAGAGGCT--TACGACCCCTTATTTACCGA

GAAAGC-TC--ACAAGAACTGCTAACTCATGCCC-CCATGTTTAACAACA

TGGCTT--------------------------------------------

-------------------TCTCA-ACTTTTAAAGGATAACAGCT-ATCC

ATTGGTCTTAGGCCCCAAAAATT-TTGGTGCAACTCCAAATAAAAGTAAT

AACCA---TGCACACTACTATAACCACCCTAACCCTGACTTCCCTAATCC

CCCCCATCCTTACCACCCTCGTTAACCCTAACAAAAAAAACTCATACCCC

CATTATGTAAAATCCATTGTCGCATCCACCTTTATTATCAGTCTCTTCCC

CACAACAATATTCATGTGCCTAGACCAAGAAGTTATTATCTCGAACTGAC

ACTGAGCCACAACCCAAACAACCCAGCTCTCCCTAAGCTTCAAACTAGAC

TACTTCTCCATAATATTCATCCCTGTAGCATTGTTCGTTACATGGTCCAT

CATAGAATTCTCACTGTGATATATAAACTCAGACCCAAACATTAATCAGT

TCTTCAAATATCTACTCATCTTCCTAATTACCATACTAATCTTAGTTACC

GCTAACAACCTATTCCAACTGTTCATCGGCTGAGAGGGCGTAGGAATTAT

ATCCTTCTTGCTCATCAGTTGATGATACGCCCGAGCAGATGCCAACACAG

CAGCCATTCAAGCAATCCTATACAACCGTATCGGCGATATCGGTTTCATC

CTCGCCTTAGCATGATTTATCCTACACTCCAACTCATGAGACCCACAACA

AATAACCCTTCTAAACGCTAATCCAAGCCTCACCCCACTACTAGGCCTCC

TCCTAGCAGCAGCAGGCAAATCAGCCCAATTAGGTCTCCACCCCTGACTC

CCCTCAGCCATAGAAGGCCCCACCCCAGTCTCAGCCCTACTCCACTCAAG

CACTATAGTTGTAGCAGGAGTCTTCTTACTCATCCGCTTCCACCCCCTAG

CA-GAAAATAGCCCACTAATCCAAACTCTAACACTATGCTTAGGCGCTAT

CACCACTCTGTTCGCAGCAGTCTGCGCCCTTACACAAAATGACATCAAAA

AAATCGTAGCCTTCTCCACTTCAAGTCAACTAGGACTCATAATAGTTACA

ATCGGCATCAACCAACCACACCTAGCATTCCTGCACATCTGTACCCACGC

CTTCTTCAAAGCCATACTATTTATGTGCTCCGGGTCCATCATCCACAACC

TTAACAATGAACAAGATATTCGAAAAATAGGAGGACTACTCAAAACCATA

CCTCTCACTTCAACCTCCCTCACCATTGGCAGCCTAGCATTAGCAGGAAT

ACCTTTCCTCACAGGTTTCTACTCCAAAGACCACATCATCGAAACCGCAA

ACATATCATACACAAACGCCTGAGCCCTATCTATTACTCTCATCGCTACC

TCCCTGACAAGCGCCTATAGCACTCGAATAATTCTTCTCACCCTAACAGG

TCAACCTCGCTTCCCCACCCTTACTAACATTAACGAAAATAACCCCACCC

TACTAAACCCCATTAAACGCCTGGCAGCCGGAAGCCTATTCGCAGGATTT

CTCATTACTAACAACATTTCCCCCGCATCCCCCTTCCAAACAACAATCCC

CCTCTACCTAAAACTCACAGCCCTCGCTGTCACTTTCCTAGGACTCCTAA

CAGCCCTAGACCTCAACTACCTAACCAACAAACTTAAAATAAAATCCCCA

CTATGCACATTT-TATTTCTCCAACATACTCGGATTCTACCCTAGCATCA

CACACCGCACAATCCCCTATCTAGGCCTTCTTACGAGCCAAAACCTGCCC

CTACTCCTCCTAGACCTAACCTGACTAGAAAAGCTATTACCTAAAACAAT

TTCACAGCACCAA---ATCTCCACCTCCATCATTACCTCAACCCAAAAAG

GCATAATTAAACTTTACTTCCTCTCTTTCTTCTTCCCACTCATCCTAACC

CTACTCCTAATCACATAACCT----------------------ATTCCCC

CGAGCAATCTCAATTACAATATATACACCAACAAACAATGTTCAACCAGT

AACTACTACTAATCAACGCCCATAATCATACAAAGCCCCCGCACCAATAG

GATCCTCCCGAATCAACCCTGACCCCTCTCCTTCATAAATTATTCAGCTT

CCTACACTATTAAAGTTTACCACAACC---ACCA------CCCCATCATA

CTCTTTCACCCACAGCACCAATCCTACCTCCATCGCTAACCCCACTAAAA

CACTCACCAAGACCTCAACCCCTGACCCCCATGCCTCAGGATACTCCTCA

ATAGCCATCGCTGTAGTATATCCAAAGACAACCATCATTCCCCCTAAATA

AATTAAAAAAACTATTAAACCCATATAACCTCCCCCAAAATTCAGAATAA

TAACACACCCGACCACACCGCTAACAATCAATACTAAACCCCCATAAATA

GGAGAAGGCTTAGAAGAAAACCCCACAAACCCCATTACTAAACCCACACT

CAACAGAAACAAAGCATACATCATTAT---TCTCG-CACGGACTACA-AC

CACGACCAATGATATGAAAAACCATCGTTGTAT-TTCAACTACAAGAACA

CC-----AATGACCCCAATACGCAAAACTAACCCCCTAATAAAATTAATT

AACCACTCATTCATCGACCTCCCCACCCCATCCAACATCTCCGCATGATG

AAACTTCGGCTCACTCCTTGGCGCCTGCCTGATCCTCCAAATCACCACAG

GACTATTCCTAGCCATGCACTACTCACCAGACGCCTCAACCGCCTTTTCA

TCAATCGCCCACATCACTCGAGACGTAAATTATGGCTGAATCATCCGCTA

CCTTCACGCCAATGGCGCCTCAATATTCTTTATCTGCCTCTTCCTACACA

TCGGGCGAGGCCTATATTACGGATCATTTCTCTACTCAGAAACCTGAAAC

ATCGGCATTATCCTCCTGCTTGCAACTATAGCAACAGCCTTCATAGGCTA

TGTCCTCCCGTGAGGCCAAATATCATTCTGAGGGGCCACAGTAATTACAA

ACTTACTATCCGCCATCCCATACATTGGGACAGACCTAGTTCAATGAATC

TGAGGAGGCTACTCAGTAGACAGTCCCACCCTCACACGATTCTTTACCTT

TCACTTCATCTTGCCCTTCATTATTGCAGCCCTAGCAGCACTCCACCTCC

TATTCTTGCACGAAACGGGATCAAACAACCCCCTAGGAATCACCTCCCAT

TCCGATAAAATCACCTTCCACCCTTACTACACAATCAAAGACGCCCTCGG

CTTACTTCTCTTCCTTCTCTCCTTAATGACATTAACACTATTCTCACCAG

ACCTCCTAGGCGACCCAGACAATTATACCCTAGCCAACCCCTTAAACACC

CCTCCCCACATCAAGCCCGAATGATATTTCCTATTCGCCTACACAATTCT

CCGATCCGTCCCTAACAAACTAGGAGGCGTCCTTGCCCTATTACTATCCA

TCCTCATCCTAGCAATAATCCCCATCCTCCATATATCCAAACAACAAAGC

ATAATATTTCGCCCACTAAGCCAATCACTTTATTGACTCCTAGCCGCAGA

CCTCCTCATTCTAACCTGAATCGGAGGACAACCAGTAAGCTACCCTTTTA

CCATCATTGGACAAGTAGCATCCGTACTATACTTCACAACAATCCTAATC

CTAATACCAACTATCTCCCTAATTGAAAACAAAATACTCAAATGGG---C

CTGTCCTTGTAGTATAAA--CTAATACACCAGTCTTGTAAACCGGAGATG

AAA-----ACCTTTTTCCAAGGACAA---ATCAGAGAAAAAGTCTTTAAC

TCCACCATTAGCACCCAAAGCTAAGATT-CTAATTTAAACTATTCTCTG-

--------------------------------------------------

--------------------------------------------------

--------------------------------------------------

--------------------------------------------------

--------------------------------------------------

--------------------------------------------------

--------------------------------------------------

--------------------------------------------------

--------------------------------------------------

--------------------------------------------------

--------------------------------------------------

--------------------------------------------------

--------------------------------------------------

--------------------------------------------------

--------------------------------------------------

--------------------------------------------------

--------------------------------------------------

--------------------------------------------------

--------------------------------------------------

--------------------------------------------------

--------------------------------------------------

--------------------------------------------------

--------------------------------------------------

------

>Homo_sapiens_FIN Homo sapiens haplogroup N1c mitochondrion, complete genome.

GTTTATGTAGCTTACCTCCT------CAAAGCAATACACTGAAAATGTTT

AGACGGGC-TCACATCACCCCATAAACAAATA--GGTTTGGTCCTAGCCT

TTCTATTAGCTCTTAGTAAGATTACACATGCAAGCATCCCCGTTCCAGTG

AGTTCACCCT-CTAAATCACCA------CGATCAAAAGGGACAAGCATCA

AGCACGC---AGCAATGCAGCTCAAAACGCTTAGC-CTAGCCACACCCCC

ACGGGAAACAGCAGTGATTAACCTTTAGCA-ATAAACGAAAGTTTAACTA

AGCTATACTA--ACCCCAGGGTTGGTCAATTTCGTGCCAGCCACCGCGGT

CACACGATTAACCCAAGTCAATAGAA--GCCGGCGTAAAGAGTGTTTTAG

AT---CACCCCCTCCCCAATAAAGCTAAAACTCACCTGAGTTGTAAAAAA

CTCCAGTTGACA--CAAAATAGACTACGAAAGTGGCTTTA---ACATATC

TGAACACACAATAGCTAAGACCCAAACTGGGATTAGATACCCCACTATGC

TTAGCCCTAAACCTCAACAGTTAAATC-AACAAAACTGCTCGCCAGAACA

CTACGAGCCACAGCTTAAAACTCAAAGGACCTGGCGGTGCTTCATATCCC

TCTAGAGGAGCCTGTTCTGTAATCGATAAACCCCGATCAACCTCACCACC

TCTTGC-----TCAGCCTATATACCGCCATCTTCAGCAAACCCTGATGAA

GGCTACAAAGTAAGCGCAAGTACCC---ACGTAAAGACGTTA-GGTCAAG

GTGTAGCCCATGAGGTGGC-AAGAAATGGGCTACATTTTCTACCCCAGAA

AA--------------CTACGATAGCCCTTATGAAACTTAA--GGGTCGA

AGGTGGATTTAGCAGTAAACT-GAGAGTAGAGTGCTTAGTTGAACAG-GG

CCCTGAAGCGCGTACACACCGCCCGTCACCCTCCTCAAGTATACTTCAAA

GGACATTTAACTAAAACCCCTACGCATTTATAT-----------AGAGGA

GACAAGTCGTAACATGGTAAGTGTACTGGAAAGTGCACTTGGACGAACCA

GAGTGTAGCTTA----ACACAAAGCACCCAACTTACACTTAGGAGATTTC

AACTTAACTTGACCGCTCTGA--------GCTAAACCTAGCCCCAAACCC

ACTCCACCTTACTACCAGACAACC-TTAACCAAACCATTTACC------C

AAATAAAGTATAGGCGATAGAAATTG----AAACCTGGCGCAATAGATAT

AGTACCGCAAGGGAAAGA-TGAAAAATTATAACCA-AGCATAATATAGCA

AGGACTAACCCCTATACCTTCTGCATAA-TGAATTAACTAGAAATAACTT

TGCAAGGAG-AGCCAAAGCTAAGACCCCCGAAACCAGACGAGCTACCTAA

GAACAGCT---AAAAGAGCACACCCGTCTATGTAGCAAAATAGTGGGAAG

ATTTATAGGTAGAGGCGACAAACCTACCGAGCCTGGTGATAGCTGGTTGT

CCAAGA-TAGAATCTTAGTTCAACTTTAAATTTGCCCACAGAACCCTCT-

AAATCCCCTTGTAAATTTAACTGTTAGTCCAAAGAGGAACAGCTCTTTGG

ACACTAGGAAAAAACCTTGTAGAGAGAGTAA-------AAAATTTAACAC

CCATAGTAGGCCTAAAAGCAGCCACCAATTAAGAAAGCGTTCAAGCTCAA

C---------ACCCACTACCTAAAAAATCCCAAACATATAACTGAACTCC

TCACACC---CAATTGGACCAATCTATCACCCTAT-AGAAGAACTAATGT

TAGTATAAGTAACATGAAAACATTCTCCTCC--GCATAAGCCTGCGTCAG

ATTAAAACACTGAACTGACAATTAACAGCCCAATA------TCTACAATC

AACCAACAAGTCATTATTACCCTCACTGTCAACCCAACACAGGCATGC--

-TCATAAGGAAAGGTTAAAAAAAGTAAAAGGAACTCGGCAAAT-CTTACC

CCGCCTGTTTACCAAAAACATCACCTCTAGCATCACCAGTATTAGAGGCA

CCGCCTGCCCAGTGACA-----CATGTTTAACGGCCGCGGTACCCTAACC

GTGCAAAGGTAGCATAATCACTTGTTCCTTAAATAGGGACCTGTATGAAT

GGCTCCACGAGGGTTCAGCTGTCTCTTACTTTTAACCAGTGAAATTGACC

TGCCCGTGAAGAGGCGGGCATGACACAGCAAGACGAGAAGACCCTATGGA

GCTTTAATTTATTAATGCAAACAGTACCTAACAAACCC------------

ACAGGTCCTAAACTACCAAACCTGCATTAAAAATTTCGGTTGGGGCGACC

TCGGAGCAGAACCCAACCTCCGAGCAGT--ACATGCTAAGACTTCACCAG

TCAAAGCGA-ACTACTATACTCAATTGATCCAATA----ACTTGACCAAC

GGAACAAGTTACCCTAGGGATAACAGCGCAATCCTATTCTAGAGTCCATA

TCAACAAT-AGGGTTTACGACCTCGATGTTGGATCAGGACATCCCGATGG

TGCAGCCGCTATTAAAG-GTTCGTTTGTTCAACGATTAA-AGTCCTACGT

GATCTGAGTTCAGACCGGAGTAATCCAGGTCGGTTTCTATCTACTTCAA-

-ATTCCTCCCTGTACGAAAGGACAAGAGAAATAAGGCCTACTTCACA--A

AGCGCCTTC--CCCCGTAAATGATATCA-TCTCAACTTAGTATTATACCC

ACA------CCCACCCAAGAACAGGG---TTTGTTAAGATGGCAGAGCCC

GGTAA-TCGCATAAAACTTAAAACTTTACAGTCAGAGGTTCAATTCCTCT

TCTTAACAAC----------------------------------------

------------------------------------------ATACCCAT

GGCCAACCTCCTACTCCTCATTGTACCCATTCTAATCGCAATGGCATTCC

TAATGCTTACCGAACGAAAAATTCTAGGCTATATACAACTACGCAAAGGC

CCCAACGTTGTAGGCCCCTACGGGCTACTACAACCCTTCGCTGACGCCAT

AAAACTCTTCACCAAAGAGCCCCTAAAACCCGCCACATCTACCATCACCC

TCTACATCACCGCCCCGACCTTAGCTCTCACCATCGCTCTTCTACTATGA

ACCCCCCTCCCCATACCCAACCCCCTGGTCAACCTCAACCTAGGCCTCCT

ATTTATTCTAGCCACCTCTAGCCTAGCCGTTTACTCAATCCTCTGATCAG

GGTGAGCATCAAACTCAAACTACGCCCTGATCGGCGCACTGCGAGCAGTA

GCCCAAACAATCTCATATGAAGTCACCCTAGCCATCATTCTACTATCAAC

ATTACTAATAAGTGGCTCCTTTAACCTCTCCACCCTTATCACAACACAAG

AACACCTCTGATTACTCCTGCCATCATGACCCTTGGCCATAATATGATTT

ATCTCCACACTAGCAGAGACCAACCGAACCCCCTTCGACCTTGCCGAAGG

GGAGTCCGAACTAGTCTCAGGCTTCAACATCGAATACGCCGCAGGCCCCT

TCGCCCTATTCTTCATAGCCGAATACACAAACATTATTATAATAAACACC

CTCACCACTACAATCTTCCTAGGAACAACATATGACGCACTCTCCCCTGA

ACTCTACACAACATATTTTGTCACCAAGACCCTACTTCTAACCTCCCTGT

TCTTATGAATTCGAACAGCATACCCCCGATTCCGCTACGACCAACTCATA

CACCTCCTATGAAAAAACTTCCTACCACTCACCCTAGCATTACTTATATG

ATATGTCTCCATACCCATTACAATCTCCAGCATTCCCCCTCAAACCTAA-

GAAATATGTCTGATAAAAGAGTTACTTTGATAGAGTAAATAATAGGAGCT

TA-AACCCCCTTATTTCTAGGACTATGAGAATCGAACCCATCCCTGAGAA

TCCAAAATTCTCCGTGCCACC--TATCACACCCCATCCTAA--------A

GTAAGGTCAGCTAAAT-AAGCTATCGGGCCCATACCCCGAAAATGTTGGT

T-ATACCCTTCCCGTACTAATTAATCCCCTGGCCCAACCCGTCATCTACT

CTACCATCTTTGCAGGCACACTCATCACAGCGCTAAGCTCGCACTGATTT

TTTACCTGAGTAGGCCTAGAAATAAACATGCTAGCTTTTATTCCAGTTCT

AACCAAAAAAATAAACCCTCGTTCCACAGAAGCTGCCATCAAGTATTTCC

TCACGCAAGCAACCGCATCCATAATCCTTCTAATAGCTATCCTCTTCAAC

AATATACTCTCCGGACAATGAACCATAACCAATACTACCAATCAATACTC

ATCATTAATAATCATAATGGCTATAGCAATAAAACTAGGAATAGCCCCCT

TTCACTTCTGAGTCCCAGAGGTTACCCAAGGCACCCCTCTGACATCCGGC

CTGCTTCTTCTCACATGACAAAAACTAGCCCCCATCTCAATCATATACCA

AATCTCTCCCTCACTAAACGTAAGCCTTCTCCTCACTCTCTCAATCTTAT

CCATCATAGCAGGCAGTTGAGGTGGATTAAACCAAACCCAGCTACGCAAA

ATCTTAGCATACTCCTCAATTACCCACATAGGATGAATAATAGCAGTTCT

ACCGTACAACCCTAACATAACCATTCTTAATTTAACTATTTATATTATCC

TAACTACTACCGCATTCCTACTACTCAACTTAAACTCCAGCACCACGACC

CTACTACTATCTCGCACCTGAAACAAGCTAACATGACTAACACCCTTAAT

TCCATCCACCCTCCTCTCCCTAGGAGGCCTGCCCCCGCTAACCGGCTTTT

TGCCCAAATGGGCCATTATCGAAGAATTCACAAAAAACAATAGCCTCATC

ATCCCCACCATCATAGCCACCATCACCCTCCTTAACCTCTACTTCTACCT

ACGCCTAATCTACTCCACCTCAATCACACTACTCCCCATATCTAACAACG

TAAAAATAAAATGACAGTTTGAACATACAAAACCCACCCCATTCCTCCCC

ACACTCATCGCCCTTACCACGCTACTCCTACCTATCTCCCCTTTTATACT

AATAATCTTA---T----AGAAATTTAGGTTAAA--TACAGACCAAGAGC

CTTCAAAGCCCTCAGTAAGT-----TGCAATACTTAATTTCTGT------

---AACAACTAAGGACTGCAAAACCCCACTCTGCAT----CAACTGAACG

CAAATCAGCCACTTTAA---TTAAGCTAAGCCCTTA-----------CTA

GACCAATG--GGACTTAAACCCA--CAAACACTTAGTTAACAGCTAAGCA

CCCTAATCAACTGGCTTCAATCTACT--TCTCCCGCCGCCGGGAAAAAA-

-----GGCGGGAGAAGCCCCGGCAGGTTT---GAAGCTGCTTCTTC----

---------------------------GAATTTGCAATTCAATATGAAAA

----TCACCTCGGAGCT-------GGTAAAAAGAGG------CCTAACCC

CTGTCTTTAGATTTACAGTCCAATGCTT-CACTCAGCCATTTTACCTCAC

CCCCACTGATGTTCGCCGACCGTTGACTATTCTCTACAAACCACAAAGAC

ATTGGAACACTATACCTATTATTCGGCGCATGAGCTGGAGTCCTAGGCAC

AGCTCTAAGCCTCCTTATTCGAGCCGAGCTGGGCCAGCCAGGCAACCTTC

TAGGTAACGACCACATCTACAACGTTATCGTCACAGCCCATGCATTTGTA

ATAATCTTCTTCATAGTAATACCCATCATAATCGGAGGCTTTGGCAACTG

ACTAGTTCCCCTAATAATCGGTGCCCCCGATATGGCGTTTCCCCGCATAA

ACAACATAAGCTTCTGACTCTTACCTCCCTCTCTCCTACTCCTGCTCGCA

TCTGCTATAGTGGAGGCCGGAGCAGGAACAGGTTGAACAGTCTACCCTCC

CTTAGCAGGGAACTACTCCCACCCTGGAGCCTCCGTAGACCTAACCATCT

TCTCCTTACACCTAGCAGGTGTCTCCTCTATCTTAGGGGCCATCAATTTC

ATCACAACAATTATCAATATAAAACCCCCTGCCATAACCCAATACCAAAC

GCCCCTCTTCGTCTGATCCGTCCTAATCACAGCAGTCCTACTTCTCCTAT

CTCTCCCAGTCCTAGCTGCTGGCATCACTATACTACTAACAGACCGCAAC

CTCAACACCACCTTCTTCGACCCCGCCGGAGGAGGAGACCCCATTCTATA

CCAACACCTATTCTGATTTTTCGGTCACCCTGAAGTTTATATTCTTATCC

TACCAGGCTTCGGAATAATCTCCCATATTGTAACTTACTACTCCGGAAAA

AAAGAACCATTTGGATACATAGGTATGGTCTGAGCTATGATATCAATTGG

CTTCCTAGGGTTTATCGTGTGAGCACACCATATATTTACAGTAGGAATAG

ACGTAGACACACGAGCATATTTCACCTCCGCTACCATAATCATCGCTATC

CCCACCGGCGTCAAAGTATTTAGCTGACTCGCCACACTCCACGGAAGCAA

TATGAAATGATCTGCTGCAGTGCTCTGAGCCCTAGGATTCATCTTTCTTT

TCACCGTAGGTGGCCTGACTGGCATTGTATTAGCAAACTCATCACTAGAC

ATCGTACTACACGACACGTACTACGTTGTAGCTCACTTCCACTATGTCCT

ATCAATAGGAGCTGTATTTGCCATCATAGGAGGCTTCATTCACTGATTTC

CCCTATTCTCAGGCTACACCCTAGACCAAACCTACGCCAAAATCCATTTC

ACTATCATATTCATCGGCGTAAATCTAACTTTCTTCCCACAACACTTTCT

CGGCCTATCCGGAATGCCCCGACGTTACTCGGACTACCCCGATGCATACA

CCACATGAAACATCCTATCATCTGTAGGCTCATTCATTTCTCTAACAGCA

GTAATATTAATAATTTTCATGATTTGAGAAGCCTTCGCTTCGAAGCGAAA

AGTCCTAATAGTAGAAGAACCCTCCATAAACCTGGAGTGACTATATGGAT

GCCCCCCACCCTACCACACATTCGAAGAACCCGTATACATAAAATCTAGA

------------------CAAAAAAGGAAGGAATCGAACCCCCCAAAGCT

GGTTTCAAGCCAACCCCATGGCCTCCATGACTTTTTCAAA--------AA

GGTATTAGAAAAACCA-TTTCATAACTTTGTCAAAGTTAAATTATAGGCT

-------AAATCCTATATATCTTA-ATGGCACATGCAGCGCAAGTAGGTC

TACAAGACGCTACTTCCCCTATCATAGAAGAGCTTATCACCTTTCATGAT

CACGCCCTCATAATCATTTTCCTTATCTGCTTCCTAGTCCTGTATGCCCT

TTTCCTAGCACTCACAACAAAACTAACTAATACTAACATCTCAGACGCTC

AGGAAATAGAAACCGTCTGAACTATCCTGCCCGCCATCATCCTAGTCCTC

ATCGCCCTCCCATCCCTACGCATCCTTTACATAACAGACGAGGTCAACGA

TCCCTCCCTTACCATCAAATCAATTGGCCACCAATGGTACTGAACCTACG

AGTACACCGACTACGGCGGACTAATCTTCAACTCCTACATACTTCCCCCA

TTATTCCTAGAACCAGGCGACCTGCGACTCCTTGACGTTGACAATCGAGT

AGTACTCCCGATTGAAGCCCCCATTCGTATAATAATTACATCACAAGACG

TCTTGCACTCATGAGCTGTCCCCACATTAGGCTTAAAAACAGATGCAATT

CCCGGACGTCTAAACCAAACCACTTTCACCGCTACACGACCGGGGGTATA

CTACGGTCAATGCTCTGAAATCTGTGGAGCAAACCACAGTTTCATGCCCA

TCGTCCTAGAACTAATTCCCCTAAAAATCTTTGAAATAGGGCCCGTATTT

ACCCTATAGCACCCCCTCTACCCCCTCTAGAG-----------CCCACTG

TAAAGCTA--ACTTAGCATTAACCTTTTAAGTTAAAGATTAAGAGAACCA

ACAC-CTCTTTACAGTGAAATGCCCCAACTAAATACTACCGTATGGCCCA

CCATAATTACCCCCA-TACTCCTTACACTATTCCTCATCACCCAACTAAA

AATATTAA---ACACAAACTACCACCTACCTCCCTCACCAAAGCCCATAA

AAATAAAAAATTATAACAAACCCTGAGAACCAAAATGAACGAAAATCTGT

TCGCTTCATTCATTGCCCCCACAATCCTAGGCCTACCCGCCGCAGTACTG

ATCATTCTATTTCCCCCTCTATTGATCCCCACCTCCAAATATCTCATCAA

CAACCGACTAATCACCACCCAACAATGACTAATCAAACTAAC-CTCAAAA

CAAATGATAACCATACACAACACTAAAGGACGAACCTGATCTCTTATACT

AGTATCCTTAATCATTTTTATTGCCACAACTAACCTCCTCGGACTCCTGC

CTCACTCATTTACACCAACCACCCAACTATCTATAAACCTAGCCATGGCC

ATCCCCTTATGAGCGGGCGCAGTGATTATAGGCTTTCGCTCTAAGA-TTA

AAAATGCCCTAGCCCACTTCTTACCACAAGGCACACCTACACCCCTTATC

CCCATACTAGTTATTATCGAAACCATCAGCCTACTCATTCAACCAATAGC

CCTGGCCGTACGCCTAACCGCTAACATTACTGCAGGCCACCTACTCATGC

ACCTAATTGGAAGCGCCACCCTAGCAATATCAACCATTAACCTTCCCTCT

ACACTTATCATCTTCACAATTCTAATTCTACTGACTATCCTAGAAATCGC

TGTCGCCCTAATCCAAGCCTACGTTTTCACACTTCTAGTAAGCCTCTACC

TGCACGACAACACATAATGACCCACCAATCACATGCCTATCATATAGTAA

AACCCAGCCCATGACCCCTAACAGGGGCCCTCTCAGCCCTCCTAATGACC

TCCGGCCTAGCCATGTGATTTCACTTCCACTCCATAACGCTCCTCATACT

AGGCCTACTAACCAACACACTAACCATATACCAATGATGGCGCGATGTAA

CACGAGAAAGCACATACCAAGGCCACCACACACCACCTGTCCAAAAAGGC

CTTCGATACGGGATAATCCTATTTATTACCTCAGAAGTTTTTTTCTTCGC

AGGATTTTTCTGAGCCTTTTACCACTCCAGCCTAGCCCCTACCCCCCAAT

TAGGAGGGCACTGGCCCCCAACAGGCATCACCCCGCTAAATCCCCTAGAA

GTCCCACTCCTAAACACATCCGTATTACTCGCATCAGGAGTATCAATCAC

CTGAGCTCACCATAGTCTAATAGAAAACAACCGAAACCAAATAATTCAAG

CACTGCTTATTACAATTTTACTGGGTCTCTATTTTACCCTCCTACAAGCC

TCAGAGTACTTCGAGTCTCCCTTCACCATTTCCGACGGCATCTACGGCTC

AACATTTTTTGTAGCCACAGGCTTCCACGGACTTCACGTCATTATTGGCT

CAACTTTCCTCACTATCTGCTTCATCCGCCAACTAATATTTCACTTTACA

TCCAAACATCACTTTGGCTTCGAAGCCGCCGCCTGATACTGGCATTTTGT

AGATGTGGTTTGACTATTTCTGTATGTCTCCATCTATTGATGAGGGTCTT

ACTCT-TTTAGTATAAA--TAGTACCGTTAACTTCCAATTAACTAGTTTT

GACAACA-TTCAAAAAAGAGTAATAAACTTCGCCTTAATTTTAATAATCA

ACACCCTCCTAGCCTTACTACTAATAATTATTACATTTTGACTACCACAA

CTCAACGGCTACATAGAAAAATCCACCCCTTACGAGTGCGGCTTCGACCC

TATATCCCCCGCCCGCGTCCCTTTCTCCATAAAATTCTTCTTAGTAGCTA

TCACCTTCTTATTATTTGATCTAGAAATTGCCCTCCTTTTACCCCTACCA

TGAGCCCTACAAACAACTAACCTGCCACTAATAGTTATGTCATCCCTCTT

ATTAATCATCATCCTAGCCCTAAGTCTGGCCTATGAGTGACTACAAAAAG

GATTAGACTGAACCGAAT-------TGGTATATAGTTTAAAC--AAAACG

AATGATTTCGACTCATTAAATTATGATAAT----CATATTTACCAA---A

TGCCCCTCATTTACATAAATATTATACTAGCATTTACCATCTCACTTCTA

GGAATACTAGTATATCGCTCACACCTCATATCCTCCCTACTATGCCTAGA

AGGAATAATACTATCGCTGTTCATTATAGCTACTCTCATAACCCTCAACA

CCCACTCCCTCTTAGCCAATATTGTGCCTATTGCCATACTAGTCTTTGCC

GCCTGCGAAGCAGCGGTGGGCCTAGCCCTACTAGTCTCAATCTCCAACAC

ATATGGCCTAGACTACGTACATAACCTAAACCTACTCCAATGCTAAAACT

AATCGTCCCAACAATTATATTACTACCACTGACATGACTTTCCAAAAAAC

ACATAATTTGAATCAACACAACCACCCACAGCCTAATTATTAGCATCATC

CCTCTACTATTTTTTAACCAAATCAACAACAACCTATTTAGCTGTTCCCC

AACCTTTTCCTCCGACCCCCTAACAACCCCCCTCCTAATACTAACTACCT

GACTCCTACCCCTCACAATCATGGCAAGCCAACGCCACTTATCCAGTGAA

CCACCATCACGAAAAAAACTCTACCTCTCTATACTAATCTCCCTACAAAT

CTCCTTAATTATAACATTCACAGCCACAGAACTAATCATATTTTATATCT

TCTTCGAAACCACACTTATCCCCACCTTGGCTATCATCACCCGATGAGGC

AACCAGCCAGAACGCCTGAACGCAGGCACATACTTCCTATTCTACACCCT

AGTAGGCTCCCTTCCCCTACTCATCGCACTAATTTACACTCACAACACCC

TAGGCTCACTAAACATTCTACTACTCACTCTCACTGCCCAAGAACTATCA

AACTCC----------TGAGCCAACAACTTAATATGACTAGCTTACACAA

TAGCTTTTATAGTAAAGATACCTCTTTACGGACTCCACTTATGACTCCCT

AAAGCCCATGTCGAAGCCCCCATCGCCGGGTCAATAGTACTTGCCGCAGT

ACTCTTAAAACTAGGCGGCTATGGTATAATACGCCTCACACTCATTCTCA

ACCCCCTGACAAAACACATAGCCTACCCCTTCCTTGTACTATCCCTATGA

GGCATAATTATAACAAGCTCCATCTGCCTACGACAAACAGACCTAAAATC

GCTCATTGCATACTCTTCAATCAGCCACATAGCCCTCGTAGTAACAGCCA

TTCTCATCCAAACCCCCTGAAGCTTCACCGGCGCAGTCATTCTCATAATC

GCCCACGGACTTACATCCTCATTACTATTCTGCCTAGCAAACTCAAACTA

CGAACGCACTCACAGTCGCATCATAATCCTCTCTCAAGGACTTCAAACTC

TACTCCCACTAATAGCTTTTTGATGACTTCTAGCAAGCCTCGCTAACCTC

GCCTTACCCCCCACTATTAACCTACTGGGAGAACTCTCTGTGCTAGTAAC

CACATTCTCCTGATCAAATATCACTCTCCTACTTACAGGACTCAACATAC

TAGTCACAGCCCTATACTCCCTCTACATATTTACCACAACACAATGGGGC

TCACTCACCCACCACATTAACAACATAAAACCCTCATTCACACGAGAAAA

CACCCTCATGTTCATACACCTATCCCCCATTCTCCTCCTATCCCTCAACC

CCGACATCATTACCGGGTTTTCCTCTTGTAAATATAGTTTAACCAAAACA

TCAGATTGTGAATCTGACAACAGAGGCTTACGA--CCCCTTATTTACCGA

GAAAGC-TC--ACAAGAACTGCTAACTCATGC-CCCCATGTCTAACAACA

TGGCTT--------------------------------------------

--------------------TCTCAACTTTTAAAGGATAACAGCT-ATCC

ATTGGTCTTAGGCCCCAAAAA-TTTTGGTGCAACTCCAAATAAAAGTAAT

AACCA---TGCACACTACTATAACCACCCTAACCCTGACTTCCCTAATTC

CCCCCATCCTTACCACCCTCGTTAACCCTAACAAAAAAAACTCATACCCC

CATTATGTAAAATCCATTGTCGCATCCACCTTTATTATCAGTCTCTTCCC

CACAACAATATTCATATGCCTAGACCAAGAAGTTATTATCTCGAACTGAC

ACTGAGCCACAACCCAAACAACCCAGCTCTCCCTAAGCTTCAAACTAGAC

TACTTCTCCATAATATTCATCCCTGTAGCATTGTTCGTTACATGGTCCAT

CATAGAATTCTCACTGTGATATATAAACTCAGACCCAAACATTAATCAGT

TCTTCAAATATCTACTCATTTTCCTAATTACCATACTAATCTTAGTTACC

GCTAACAACCTATTCCAACTGTTCATCGGCTGAGAGGGCGTAGGAATTAT

ATCCTTCTTGCTCATCAGTTGATGATACGCCCGAGCAGATGCCAACACAG

CAGCCATTCAAGCAATCCTATACAACCGTATCGGCGATATCGGTTTCATC

CTCGCCTTAGCATGATTTATCCTACACTCCAACTCATGAGACCCACAACA

AATAGCCCTTCTAAACGCTAATCCAAGCCTCACCCCACTACTAGGCCTCC

TCCTAGCAGCAGCAGGCAAATCAGCCCAATTAGGTCTCCACCCCTGACTC

CCCTCAGCCATAGAAGGCCCCACCCCAGTCTCAGCCCTACTCCACTCAAG

CACTATAGTTGTAGCAGGAATCTTCTTACTCATCCGCTTCCACCCCCTAG

CA-GAAAATAGCCCACTAATCCAAACTCTAACACTATGCTTAGGCGCTAT

CACCACTCTGTTCGCAGCAGTCTGCGCCCTTACACAAAATGACATCAAAA

AAATCGTAGCCTTCTCCACTTCAAGTCAACTAGGACTCATAATAGTTACA

ATCGGCATCAACCAACCACACCTAGCATTCCTGCACATCTGTACCCACGC

CTTCTTCAAAGCCATACTATTTATGTGCTCCGGGTCCATCATCCACAACC

TTAACAATGAACAAGATATTCGAAAAATAGGAGGACTACTCAAAACCATA

CCTCTCACTTCAACCTCCCTCACCATTGGCAGCCTAGCATTAGCAGGAAT

ACCTTTCCTCACAGGTTTCTACTCCAAAGACCACATCATCGAAACCGCAA

ACATATCATACACAAACGCCTGAGCCCTATCTATTACTCTCATCGCTACC

TCCCTGACAAGCGCCTATAGCACTCGAATAATTCTTCTCACCCTAACAGG

TCGACCTCGCTTCCCCACCCTTACTAACATTAACGAAAATAACCCCACCC

TACTAAACCCCATTAAACGCCTGGCAGCCGGAAGCCTATTCGCAGGATTT

CTCATTACTAACAACATTTCCCCCGCATCCCCCTTCCAAACAACAGTCCC

CCTCTACCTAAAACTCACAGCCCTCGCTGTCACTTTCCTAGGACTTCTAA

CAGCCCTAGACCTCAACTACCTAACCAACAAACTTAAAATAAAATCCCCA

CTATGCACATTTTA-TTTCTCCAACATACTCGGATTCTACCCTAGCATCA

CACACCGCACAATCCCCTATCTAGGCCTTCTTACGAGCCAAAACCTGCCC

CTACTCCTCCTAGACCTAACCTGACTAGAAAAGCTATTACCTAAAACAAT

TTCACAGCACCAAATCTCCA---CCTCCATCATCACCTCAACCCAAAAAG

GCATAATTAAACTTTACTTCCTCTCTTTCTTCTTCCCACTCATCCTAACC

CTACTCCTAATCACATAACCT----------------------ATTCCCC

CGAGCAATCTCAATTACAATATATACACCAACAAACAATGTTCAACCAGT

AACTACTACTAATCAACGCCCATAATCATACAAAGCCCCCGCACCAATAG

GATCCTCCCGAATCAACCCTGACCCCTCTCCTTCATAAATTATTCAGCTT

CCTACACTATTAAAGTTTACCACAACCACCA---------CCCCATCATA

CTCTTTCACCCACAGCACCAATCCTACCTCCATCGCTAACCCCACTAAAA

CACTCACCAAGACCTCAACCCCTGACCCCCATGCCTCAGGATACTCCTCA

ATAGCCATCGCTGTAGTATATCCAAAGACAACCATCATTCCCCCTAAATA

AATTAAAAAAACTATTAAACCCATATAACCTCCCCCAAAATTCAGAATAA

TAACACACCCGACCACACCGCTAACAATCAATACTAAACCCCCATAAATA

GGAGAAGGCTTAGAAGAAAACCCCACAAACCCCATTACTAAACCCACACT

CAACAGAAACAAAGCATACATCATTAT----TCTCGCACGGACTACA-AC

CACGACCAATGATATGAAAAACCATCGTTGTAT-TTCAACTACAAGAACA

CC-----AATGACCCCAATACGCAAAATTAACCCCCTAATAAAATTAATT

AACCACTCATTCATCGACCTCCCCACCCCATCCAACATCTCCGCATGATG

AAACTTCGGCTCACTCCTTGGCGCCTGCCTGATCCTCCAAATCACCACAG

GACTATTCCTAGCCATGCACTACTCACCAGACGCCTCAACCGCCTTTTCA

TCAATCGCCCACATCACTCGAGACGTAAATTATGGCTGAATCATCCGCTA

CCTTCACGCCAATGGCGCCTCAATATTCTTTATCTGCCTCTTCCTACACA

TCGGGCGAGGCCTATATTACGGATCATTTCTCTACTCAGAAACCTGAAAC

ATCGGCATTATCCTCCTGCTTGCAACTATAGCAACAGCCTTCATAGGCTA

TGTCCTCCCGTGAGGCCAAATATCATTCTGAGGGGCCACAGTAATTACAA

ACTTACTATCCGCCATCCCATACATTGGGACAGACCTAGTTCAATGAATC

TGAGGAGGCTACTCAGTAGACAGTCCCACCCTCACACGATTCTTTACCTT

TCACTTCATCTTGCCCTTCATTATTGCAGCCCTAGCAGCACTCCACCTCC

TATTCTTGCACGAAACGGGATCAAACAACCCCCTAGGAATCACCTCCCAT

TCCGATAAAATCACCTTCCACCCTTACTACACAATCAAAGACGCCCTCGG

CTTACTTCTCTTCCTTCTCTCCTTAATGACATTAACACTATTCTCACCAG

ACCTCCTAGGCGACCCAGACAATTATACCCTAGCCAACCCCTTAAACACC

CCTCCCCACATCAAGCCCGAATGATATTTCCTATTCGCCTACACAATTCT

CCGATCCGTCCCTAACAAACTAGGAGGCGTCCTTGCCCTATTACTATCCA

TCCTCATCCTAGCAATAATCCCCATCCTCCATATATCCAAACAACAAAGC

ATAATATTTCGCCCACTAAGCCAATCACTTTATTGACTCCTAGCCGCAGA

CCTCCTCATTCTAACCTGAATCGGAGGACAACCAGTAAGCTACCCTTTTA

CCATCATTGGACAAGTAGCATCCGTACTATACTTCACAACAATCCTAATC

CTAATACCAACTATCTCCCTAATTGAAAACAAAATACTCAAATGGG---C

CTGTCCTTGTAGTATA--AACTAATACACCAGTCTTGTAAACCGGAGATG

AA--------AACCTTTTTCCAAGGACAAATCAGAGAAAAAGTCTTTAAC

TCCACCATTAGCACCCAAAGCTAAGATT-CTAATTTAAACTATTCTCTG-

--------------------------------------------------

--------------------------------------------------

--------------------------------------------------

--------------------------------------------------

--------------------------------------------------

--------------------------------------------------

--------------------------------------------------

--------------------------------------------------

--------------------------------------------------

--------------------------------------------------

--------------------------------------------------

--------------------------------------------------

--------------------------------------------------

--------------------------------------------------

--------------------------------------------------

--------------------------------------------------

--------------------------------------------------

--------------------------------------------------

--------------------------------------------------

--------------------------------------------------

--------------------------------------------------

--------------------------------------------------

--------------------------------------------------

------

>Bos_primigenius_taurus_Yakutia Bos taurus isolate ERR2734956 mitochondrion, complete genome.

ATTGATGTAGCTTAACC---------CAAAGCAAGGCACTGAAAATGCCT

AGATGAGTCTCCC--AACTCCATAAACACATA--GGTTTGGTCCCAGCCT

TCCTGTTAACTCTTAATAAACTTACACATGCAAGCATCTACACCCCAGTG

AGAATGCCCT-CTAGGTTATTAAA------ACTAAGAGGAGCTGGCATCA

AGCACAC----ACCCTGTAGCTCACGACGCCTTGC-TTAACCACACCCCC

ACGGGAAACAGCAGTGACAAAAATTAAGCC-ATAAACGAAAGTTTGACTA

AGTTATATTA----ATTAGGGTTGGTAAATCTCGTGCCAGCCACCGCGGT

CATACGATTAACCCAAGCTAACAGGAGTAC-GGCGTAAAACGTGTTAAAG

CA-------CCATACCAAATAGGGTTAAATTCTAACTAAGCTGTAAAAAG

CCATGATTAA-AATAAAAATAAATGACGAAAGTGACCCTA----CAATAG

CCGACGCACTATAGCTAAGACCCAAACTGGGATTAGATACCCCACTATGC

TTAGCCCTAAACACAGATAATTACAT-AAACAAAATTATTCGCCAGAGTA

CTACTAGCAACAGCTTAAAACTCAAAGGACTTGGCGGTGCTTTATATCCT

TCTAGAGGAGCCTGTTCTATAATCGATAAACCCCGATAAACCTCACCAAT

TCTTGCTAATA-CAGTCTATATACCGCCATCTTCAGCAAACCCTAA-AAA

GGAAAAAAAGTAAGCGTAATTATGAT--ACATAAAAACGTTA-GGTCAAG

GTGTAACCTATGAAATGGG-AAGAAATGGGCTACATTCTCTACACCAAGA

GAAT---------CAAGCACGAAAGTTATTATGAAACCAA---TAACCAA

AGGAGGATTTAGCAGTAAACT-AAGAATAGAGTGCTTAGTTGAATTA-GG

CCATGAAGCACGCACACACCGCCCGTCACCCTCCTCAAATAGATTCAGTG

CATCTAACCCTATTTAAACGCACTAGCT-----------ACATGAGAGGA

GACAAGTCGTAACAAGGTAAGCATACTGGAAAGTGTGCTTGGATAAATCA

AGATATAGCTTA-----AACAAAGCATCCAGTTTACACCTAGAAGACTTC

A-TTCATTATGAATATCTTGAACT---AGACCTAGCCCAAAGATACCCT-

-CTCGACTAAACAACCAAGATAGAATAAAACAAAACATTTAATCCCAA--

-TTTAAAGTATAGGAGATAGAAATCTAAGTAC----GGCGCTATAGAGAA

AGTACCGCAAGGGAACGATGAAAGAAAAAAACTAAAAGTATAAAAAAGCA

AAGATTACCCCTTGTACCTTTTGCATAA-TGAATTAACTAGTATAAGACT

TAACAAAATGAATTTTAGCTAAGCAGCCCGAAACCAGACGAGCTACTCAC

AAACAGTTT-ACCAAGAACTAACTCATCTATGTGGCAAAATAGTGAGAAG

ATTTGTAAGTAGAGGTGACATGCCTAACGAGCCTGGTGATAGCTGGTTGT

CCAGAAAATGAATCTAAGTTCAGCTTTAAAGATACC--AAAAATTCAAAT

AAACCCCACTGTAGCTTTAAAAGTTAGTCTAAAAAGGTACAGCCTTTTAG

AA--ACGGATACAACCTTGACTAGAGAGTAA-------AATTTAACACTA

CCATAGTAGGCCTAAAAGCAGCCATCAATTAAGAAAGCGTTAAAGCTCAA

C------AACAAAAATTAAATAGATTCCAACAACAAAT--GATTAACT--

CCTAGCCCCAATACTGGACTAATCTATTATAGAAT-AGAAGCAATAATGT

TAATATGAGTAACAAGAAAAAT--TTTCTCCTTGCATAAGTCTAAGTCAG

TGCCTGATAATACTCTGACCACTAACAGTCAATAAA------AATAATCC

AACAATAAACAATTTATTGATTATACTGTTAACCCAACACAGGAGTGCA-

--TCTAAGGAAAGATTAAAAGAAGTAAAAGGAACTCGGCAAACACAAACC

CCGCCTGTTTACCAAAAACATCACCTCCAGCATTCCCAGTATTGGAGGCA

TTGCCTGCCCAGTGACAAC-----TGTTTAACGGCCGCGGTATCCTGACC

GTGCAAAGGTAGCATAATCATTTGTTCTCTAAATAAGGACTTGTATGAAT

GGCCGCACGAGGGTTTTACTGTCTCTTACTTCCAATCAGTGAAATTGACC

TTCCCGTGAAGAGGCGGGAATGCACAAATAAGACGAGAAGACCCTATGGA

GCTTTAACTAACCAACCCAAAGAGAATAGATTTAACCATT---------A

AGGAATAACAACAATCTCCA--TGAGTTGGTAGTTTCGGTTGGGGTGACC

TCGGAGAATAAAAAATCCTCCGAGCGATTTTAAAGACTAGACCC-ACAAG

TCAAA----TCACTCTATCGCTCATTGATCCAAAA----ACTTGATCAAC

GGAACAAGTTACCCTAGGGATAACAGCGCAATCCTATTCAAGAGTCCATA

TCGACAAT-AGGGTTTACGACCTCGATGTTGGATCAGGACATCCTGATGG

TGCAACCGCTATCAAAG-GTTCGTTTGTTCAACGATTAA-AGTCCTACGT

GATCTGAGTTCAGACCGGAGTAATCCAGGTCGGTTTCTATCTATTAC--G

TATTTCTCCCAGTACGAAAGGACAAGAGAAATAAGGCCAACTTTAAATCA

AGCGCCTTAAGA-CAACCAATGATAACA-TCTCAA-CTGACAACACAAAA

C--------CCTGCCCTAGAACAGGG--CTTAGTTAAGGTGGCAGAGCCC

GGTAA-TTGCATAAAACTTAAACTTTTATATCCAGAGATTCAAATCCTCT

CCTTAACAA-----------------------------------------

-----------------------------------------AATGTTCAT

AATTAACATCTTAATACTAATTATTCCCATCCTATTGGCCGTAGCATTCC

TTACGTTAGTGGAACGAAAAGTTCTAGGCTATATACAACTCCGAAAAGGT

CCAAATGTCGTAGGTCCATATGGCCTACTTCAACCCATCGCCGATGCAAT

CAAACTTTTCATTAAAGAACCACTACGACCCGCTACATCTTCAGCCTCAA

TATTTATCCTAGCACCTATCATAGCTTTAGGCCTAGCCTTAACCATGTGA

ATTCCCCTACCAATACCCTATCCTCTTATCAACATAAACCTAGGAGTCCT

ATTTATACTAGCCATATCAAGCCTAGCCGTATACTCCATTCTCTGATCAG

GCTGAGCTTCCAACTCAAAATACGCACTAATCGGAGCCCTACGAGCAGTA

GCACAAACAATCTCATACGAAGTAACGCTAGCAATTATCCTGTTATCAGT

ACTCCTAATAAGTGGGTCCTTTACCCTCTCCACATTAATTACTACACAAG

AACAAATATGGTTAATCCTCCCAGCATGGCCTCTAGCAATAATATGATTT

ATCTCAACACTAGCAGAAACAAACCGAGCTCCATTTGATTTAACTGAAGG

AGAATCAGAGCTAGTCTCGGGCTTCAACGTAGAATATGCAGCAGGACCAT

TTGCCCTCTTCTTCATAGCAGAGTACGCAAATATTATCATAATAAATATC

TTTACAGCAATTTTATTCCTAGGAACATCCCACAATCCACACATACCAGA

ACTCTACACAATCAATTTTACCATTAAATCCCTACTGCTCACAATATCCT

TCCTATGAATCCGAGCATCCTACCCTCGATTTCGCTATGACCAACTAATA

CACTTACTATGAAAAAATTTTCTACCTCTGACACTAGCCCTGTGCATGTG

ACACGTATCCCTACCCATCCTTACATCAGGCATCCCACCACAAACATAA-

GAAATATGTCTGACAAAAGAGTTACTTTGATAGAGTAAATAATAGAGGTT

CA-AACCCTCTTATTTCTAGAACTATAGGAATCGAACCTACTCCTAAGAA

TCCAAAACTCTTCGTGCTCCCAAT--TACACCAAATTCTA-------TTA

GTAAGGTCAGCTAA-TTAAGCTATCGGGCCCATACCCCGAAAATGTTGGT

TTATATCCTTCCCGTACTAATAAACCCAATTATCTTTATTATTATTCTAC

TAACCATTATACTAGGAACTATTATTGTCATAATCAGTTCTCACTGACTA

CTTGTCTGAATCGGGTTTGAAATAAATATACTCGCCATCATCCCCATCAT

AATAAAAAATCACAACCCACGAGCTACAGAAGCATCAACTAAATATTTTT

TGACTCAATCAACAGCCTCAATACTACTAATAATAGCCGTCATCATTAAC

CTAATATTCTCAGGCCAATGAACCGTAATAAAACTATTTAACCCAATAGC

CTCAATACTTATAACGATAGCCCTAGCTATAAAACTAGGAATAGCCCCAT

TTCACTTCTGAGTCCCAGAAGTAACACAGGGCATCCCCCTATCCTCAGGC

CTTATCCTACTGACATGACAAAAACTAGCACCTATATCTGTACTTTACCA

AATCTTCCCATCAATTAACCTAAACTTAATTCTAACCCTATCAGTTTTAT

CAATCCTAATTGGAGGCTGAGGGGGACTAAACCAAACACAACTCCGAAAA

ATCATAGCCTACTCATCAATCGCTCATATAGGCTGAATAACAGCAGTACT

ACCATATAACCCCACCATAACATTGCTAAACTTAATTATCTATATCATTA

TAACTTCCACCATATTTACCATATTTATAGCCAATTCCACCACCACTACC

CTGTCATTATCACACACATGAAATAAAACACCCATTATAACCGTCCTAAT

TCTTGCCACTCTCCTATCCATAGGAGGACTCCCTCCCCTATCTGGGTTTA

TACCAAAATGAATAATCATCCAAGAGATAACAAAAAATAACAGCATCATT

CTACCCACTTTCATAGCAATCACAGCTCTACTAAACTTATATTTTTATAT

ACGACTCACGTATTCTACCACACTAACAATATTTCCCTCCACAAACAACA

TAAAAATAAAATGACAATTTCCCCTTATGAAAAAAATAACTTTTCTACCA

ACAATAGTCGTATTATCTACCATAATACTACCACTCACGCCAATACTATC

AGTGTTAGAA-------TAGGAATTTAGGTTAAA----CAGACCAAGAGC

CTTCAAAGCCCTAAGCAAGTAC----AATTTACTTAATTCCTGA------

---------TAAGGATTGCAAGACTACACCTTACAT----CAATTGAATG

CAAATCAACCACTTTAA---TTAAGCTAAATCCTCA-----------CTA

GACTGGT---GGGCTCCACCCCCAC-GAAACTTTAGTTAACAGCTAAACA

CCCTAGCTAACTGGCTTCAATCTACT--TCTCCCGCCGCAAGAAAAAAAA

-----GGCGGGAGAAGCCCCGGCAGAATT---GAAGCTGCTTCTCT----

---------------------------GAATTTGCAATTCAACGTGTAAA

T---TCACCACAGGGCTT------GGTAAAAAGAGGAG----TCAAACCT

CTATCTTTAGATTTACAGTCTAATGCTT-TGCTCAGCCATTTTACC----

-------CATGTTCATCAACCGCTGACTATTCTCAACCAACCATAAAGAT

ATTGGTACCCTTTATCTACTATTTGGTGCTTGGGCCGGTATAGTAGGAAC

AGCTCTAAGCCTTCTAATTCGCGCTGAATTAGGCCAACCCGGAACTCTGC

TCGGAGACGACCAAATCTACAACGTAGTTGTAACCGCACACGCATTTGTA

ATAATCTTCTTCATAGTAATACCAATCATAATTGGAGGATTCGGTAACTG

ACTTGTTCCCCTAATAATTGGTGCTCCCGATATAGCATTTCCCCGAATAA

ATAATATAAGCTTCTGACTCCTCCCTCCCTCATTCCTACTACTCCTCGCA

TCCTCTATAGTTGAAGCTGGGGCAGGAACAGGCTGAACCGTGTACCCTCC

CTTAGCAGGCAACCTAGCCCATGCAGGAGCTTCAGTAGATCTAACCATTT

TCTCTTTACACTTAGCAGGAGTTTCCTCAATTTTAGGAGCCATCAACTTC

ATTACAACAATTATCAACATAAAGCCCCCCGCAATGTCACAATACCAAAC

CCCTCTGTTCGTATGATCCGTAATAATTACCGCCGTACTACTACTACTCT

CGCTCCCTGTATTAGCAGCCGGCATCACAATGCTATTAACAGACCGGAAC

CTAAATACAACCTTCTTCGACCCGGCAGGAGGAGGAGACCCTATTCTATA

TCAACACTTATTCTGATTCTTTGGACACCCCGAAGTCTATATTTTAATCT

TACCTGGGTTTGGAATAATCTCTCATATCGTGACCTACTACTCAGGAAAA

AAAGAACCATTCGGATATATGGGAATAGTTTGGGCTATAATGTCAATCGG

ATTTCTAGGTTTCATCGTATGAGCCCACCATATATTCACTGTCGGAATAG

ACGTCGACACACGAGCCTACTTCACATCAGCCACTATAATTATTGCTATT

CCAACCGGGGTAAAAGTCTTCAGCTGATTGGCAACACTTCATGGAGGTAA

TATCAAATGGTCTCCTGCTATAATGTGAGCCCTAGGCTTTATTTTCTTAT

TTACAGTAGGGGGTTTAACTGGAATTGTCTTAGCCAACTCTTCCCTCGAT

ATTGTTCTTCACGACACATACTACGTTGTCGCACATTTCCACTATGTTTT

ATCAATAGGAGCTGTATTTGCTATTATAGGGGGATTTGTTCATTGATTCC

CACTATTCTCAGGTTATACTCTCAACGATACATGAGCCAAAATCCACTTC

GCAATTATATTTGTAGGCGTCAATATAACCTTCTTCCCACAACACTTTCT

AGGACTATCTGGCATGCCTCGACGATACTCCGACTACCCAGATGCATACA

CAATATGAAATACTATCTCATCAATAGGCTCATTCATTTCCCTAACAGCA

GTTATACTAATAGTTTTCATCATCTGAGAAGCATTTGCATCTAAACGAGA

AGTCTTGACTGTAGACTTAACCACGACAAATCTAGAATGATTAAACGGAT

GCCCTCCACCATATCACACATTTGAAGAACCCACCTATGTTAACCTAAAA

------------------TAAGAAAGGAAGGAATCGAACCCCCTACTATT

GGTTTCAAGCCAACATCATAACCTCTATGTCTCTCTC----AATAAACGA

GGTGTTAGTAAAAC--ATTATATAATTTTGTCAAAGTTAAGTTACAAGTG

A------AAGTCCTGTACACCTCATATGGCATATCCCATACAACTAGGAT

TCCAAGATGCAACATCACCAATCATAGAAGAACTACTTCACTTTCATGAC

CACACGCTAATAATTGTCTTCTTAATTAGCTCATTAGTACTTTACATTAT

TTCACTAATACTAACGACAAAGCTGACCCATACAAGCACGATAGATGCAC

AAGAAGTAGAGACAATCTGAACCATTCTGCCCGCCATCATCTTAATTCTA

ATTGCTCTTCCTTCTTTACGAATTCTATACATAATAGATGAAATCAATAA

CCCATCTCTTACAGTAAAAACCATAGGACATCAGTGATACTGAAGCTATG

AGTATACAGATTATGAGGACTTAAGCTTCGACTCCTACATAATTCCAACA

TCAGAATTAAAGCCAGGGGAGCTACGACTATTAGAAGTCGATAATCGAGT

TGTACTACCAATAGAAATAACAATCCGAATGTTAGTCTCCTCTGAAGACG

TATTACACTCATGAGCTGTGCCCTCTCTAGGACTAAAAACAGACGCAATC

CCAGGCCGTCTAAACCAAACAACCCTTATATCGTCCCGTCCAGGCTTATA

TTACGGTCAATGCTCAGAAATTTGCGGGTCAAACCACAGTTTCATACCCA

TTGTCCTTGAGTTAGTCCCACTAAAGTACTTTGAAAAATGATCTGCGTCA

ATATTATAAAA---------------------------------TCACTA

AGAAGCTAT---ATAGCACTAACCTTTTAAGTTAGAGATTGAGAGCC---

ATATACTCTCCTTGGTGACATGCCGCAGCTAGACACGTCAACATGACTGA

CAATGATCTTATCAA-TATTCTTGACCCTTTTTATCATCTTTCAACTAAA

AGTTTCAAAACA---CAACTTTTATCACAATCCAGAACTGACACCAACAA

AAATATTAAAACAAAACACCCCTTGAGAAACAAAATGAACGAAAATTTAT

TTACCTCTTTTATCACCCCTGTAATTTTAGGTCTCCCTCTCGTAACCCTT

ATCGTACTATTCCCAAGCCTACTATTCCCAACATCAAACCGACTAGTAAG

CAATCGCTTTGTAACCCTCCAACAATGAATACTTCAACTTGTA-TCAAAA

CAAATAATGAGTATCCACAATTCTAAAGGACAAACATGAACATTAATATT

AATATCTCTGATCCTATTTATTGGATCAACAAACCTACTAGGCCTATTAC

CCCATTCATTCACACCAACAACACAACTATCAATAAACCTAGGCATAGCC

ATCCCCCTGTGAGCAGGAGCCGTAATTACAGGATTCCGCAATAAAA-CTA

AAGCATCACTTGCCCATTTCTTACCACAAGGAACACCCACTCCACTAATC

CCAATACTAGTAATTATTGAAACTATCAGCCTTTTTATTCAACCTATAGC

CCTCGCCGTGCGGTTAACAGCTAACATCACTGCAGGACACCTATTAATTC

ACCTAATCGGAGGAGCTACACTTGCACTAATAAGCATTAGCACTACAACA

GCTCTAATTACATTCACCATTCTAATCCTACTAACAATTCTAGAGTTTGC

AGTAGCTATAATCCAAGCCTATGTATTCACTCTCCTAGTCAGCCTATATC

TGCATGACAACACATAATGACACACCAAACTCATGCTTATCATATAGTAA

ACCCAAGCCCTTGACCTCTTACAGGAGCTTTGTCTGCCCTCTTAATAACA

TCCGGCCTAACCATGTGATTTCACTTTAACTCAATGACCCTGCTAATAAT

TGGCCTAACAACAAATATACTAACAATATACCAATGATGACGAGATGTTA

TCCGAGAAAGCACCTTCCAAGGGCACCATACCCCAGCTGTCCAAAAAGGC

CTCCGTTATGGAATAATTCTTTTTATTATCTCCGAAGTACTATTCTTTAC

CGGATTTTTCTGAGCTTTCTACCACTCAAGCCTCGCCCCCACCCCTGAAC

TAGGCGGCTGCTGACCCCCAACAGGCATTCACCCACTAAACCCCCTAGAA

GTCCCACTGCTCAACACCTCTGTCCTATTGGCTTCCGGAGTTTCTATTAC

CTGAGCCCATCATAGTTTAATAGAAGGGGACCGAAAGCATATATTACAAG

CCCTATTTATCACCATCACATTAGGAGTCTACTTCACACTACTACAAGCC

TCAGAATACTATGAAGCACCTTTTACTATCTCCGACGGAGTTTACGGCTC

AACTTTTTTTGTAGCCACAGGCTTCCACGGCCTCCACGTCATCATTGGGT

CCACCTTCTTAATTGTCTGCTTCTTCCGCCAATTAAAATTTCATTTTACT

TCTAACCACCACTTCGGCTTTGAAGCCGCTGCCTGATACTGACATTTCGT

AGACGTAGTCTGACTTTTCCTCTATGTTTCTATCTATTGATGAGGCTCCT

ATTCT-TTTAGTATTAA-CTAGTACAGCTGACTTCCAATCAGCTAGTTTC

GGT-CTAGTCCGAAAAAGAATAATAAATTTAATACTAGCCCTCCTGACCA

ATTTTACACTAGCCACCCTACTCGTCATCATCGCATTCTGACTTCCCCAA

CTAAATGTATACTCTGAGAAAACAAGCCCATACGAATGTGGATTTGACCC

CATAGGATCAGCCCGCCTTCCCTTCTCTATAAAATTCTTTCTGGTAGCCA

TCACATTCCTCTTATTTGACCTAGAAATTGCACTCCTCCTACCACTGCCA

TGAGCCTCACAAACAGCAAATCTAAACACAATGCTTACCATAGCCCTCTT

CCTAATTATCCTCCTAGCTGTAAGCCTAGCCTATGAGTGAACTCAAAAAG

GACTAGAATGAACCGAATA------TGGTACTTAGTTTAAAATAAAA-TA

AATGATTTCGACTCATTAGATTATGATTTAATT-CATAATTACCAA---A

TGTCTATAGTATACATAAACATTATAATAGCATTCACAGTATCTCTTGTA

GGACTACTAATATACCGATCCCACCTAATATCCTCCCTTCTATGCTTAGA

AGGAATAATGCTATCCCTATTCGTTATAGCAGCCCTAACAATCCTCAACT

CACATTTTACATTAGCTAGCATAATACCTATTATCCTACTAGTCTTCGCA

GCCTGTGAAGCAGCCCTAGGTCTATCTCTACTAGTAATAGTATCAAATAC

ATATGGTACTGATTATGTACAAAACCTCAACTTACTCCAATGCTAAAATA

CATTATTCCAACAATTATACTTATACCCCTAACCTGGTTATCAAAAAATA

ATATAATTTGGGTTAACTCCACAGCACACAGCCTTCTAATTAGCTTTACA

AGCCTCCTCCTCATAAACCAGTTTGGCGACAACAGCCTTAATTTTTCACT

ACTATTTTTCTCCGACTCCCTATCCACTCCACTACTAATTTTAACCATAT

GGCTCCTCCCTCTAATACTAATAGCTAGCCAACATCATCTATCAAAAGAA

AACCTAACCCGAAAAAAACTATTTATTACTATGCTGATCTCACTACAACT

ATTCCTAATTATAACCTTTACCGCCATGGAACTAATCTTATTTTATATTC

TATTTGAAGCAACACTAGTCCCAACACTCATTATTATTACCCGATGAGGA

AACCAAACAGAACGCCTAAACGCCGGACTCTATTTCCTATTCTATACACT

AGCTGGCTCCCTACCCCTATTAGTCGCACTAATTTATATCCAAAACACAG

TAGGATCCCTAAATTTCCTAATATTACAGTACTGAGTACAACCTGTTCAT

AACTCT----------TGATCTAATGTCTTCATATGACTAGCATGTATAA

TAGCTTTCATAGTAAAAATACCACTATATGGCCTCCACCTTTGACTACCT

AAAGCTCACGTAGAAGCCCCCATCGCAGGCTCCATAGTCCTTGCAGCAGT

TCTACTAAAACTAGGGGGGTACGGTATGCTACGAATCACACTAATTCTAA

ACCCTATGACCGACTTTATAGCATACCCATTCATTATACTCTCCCTATGA

GGCATAATTATAACCAGCTCAATCTGCCTCCGTCAAACGGACCTAAAATC

ACTCATCGCATACTCCTCTGTAAGCCACATAGCACTCGTTATCGTAGCCA

TCCTTATCCAGACACCTTGAAGCTACATAGGAGCAACCGCCCTTATGATT

GCCCACGGCCTCACATCCTCCATACTTTTCTGTCTAGCAAACTCAAACTA

CGAACGAATCCACAGCCGAACCATAATTCTAGCTCGAGGCCTACAAACGC

TCCTTCCACTAATAGCCACCTGATGACTACTAGCAAGTCTAACCAACTTA

GCTCTACCCCCAACAATCAACTTAATTGGAGAACTATTTGTAGTAATGTC

AACCTTTTCATGATCTAACATTACAATTATTCTAATAGGAGTAAATATAG

TAATCACCGCCCTATATTCTCTATACATGCTAATTATAACCCAACGAGGA

AAATATACCTACCACATTAATAATATCTCGCCTTCCTTTACACGGGAAAA

TGCACTCATATCATTACACATCCTACCCCTACTACTCCTAACCCTAAACC

CAAAAATTATTCTAGGACCTCTATACTGTAAATATAGTTTAACAAAAACA

TTAGATTGTGAATCTAACAATAGAAACTCATTA-CCTTCTTATTTACCGA

AAAAGTAT---GCAAGAACTGCTAATTCTATGCTCCCATATCTAATAGTA

TGGCTT--------------------------------------------

-------------------TTTCGAACTTTTAAAGGATAGTAGTTTATCC

GTTGGTCTTAGGAACCAAAAA--ATTGGTGCAACTCCAAATAAAAGTAAT

AAACA---TATTCTCCTCACTCTCACTAGTTACTTTACTCTTACTAACTA

TACCCATTATAATAATAAGCTTTAACACCTACAAACCTTCCAACTACCCA

CTCTACGTAAAAACAGCTATCTCATACGCCTTCATTACCAGCATAATTCC

CACAATAATATTTATCCACTCAGGCCAAGAACTAATTATTTCAAACTGAC

ACTGACTAACCATCCAAACTCTTAAATTATCCCTCAGCTTTAAAATAGAC

TATTTCTCAATAATATTTATCCCAGTAGCACTATTCGTCACATGATCTAT

TATAGAATTCTCAATATGATATATATACTCAGACCCCAATATTAACAAAT

TCTTCAAATATCTACTCCTATTCCTCATTACTATGCTCATCCTTGTAACC

GCAAACAACCTCTTCCAGCTATTCATTGGCTGAGAAGGCGTCGGAATCAT

ATCATTTCTACTCATCGGATGATGATACGGACGAGCAGATGCAAACACAG

CAGCCCTACAAGCAATCTTATATAACCGCATCGGCGACATTGGTTTCATT

TTAGCAATAGCATGGTTCCTAACAAATCTCAATACCTGAGACCTCCAGCA

GATCTTCATACTAAACCCAAGCGACTCAAACATACCCTTGATTGGACTAG

CATTAGCTGCAACCGGAAAATCCGCCCAATTTGGCCTCCACCCGTGACTT

CCCTCTGCAATAGAAGGCCCAACTCCCGTCTCAGCACTACTCCATTCAAG

CACAATAGTGGTAGCAGGTATCTTCCTACTAATCCGTTTCTATCCCCTCA

CA-GAAAACAATAAATACATCCAATCTATTACATTATGCTTAGGAGCCAT

TACCACACTATTTACAGCAATATGCGCCCTCACCCAAAATGACATTAAAA

AAATCATCGCCTTCTCCACATCCAGTCAACTGGGCCTTATAATAGTAACT

ATTGGCATTAACCAACCTTACCTAGCTTTCCTCCACATCTGTACCCACGC

CTTTTTCAAAGCTATACTATTCATATGCTCCGGTTCCATTATTCACAGCC

TAAACGACGAACAAGATATTCGAAAAATAGGAGGCCTATTTAAAGCCATG

CCATTCACCACAACAGCCCTCATTGTTGGCAGTCTCGCACTAACAGGAAT

ACCCTTCCTCACAGGATTCTACTCCAAAGACCTAATCATCGAAGCCGCCA

ACACGTCTTATACCAACGCCTGAGCCCTTCTAATAACATTAATTGCCACC

TCTTTCACAGCTATTTACAGCACCCGTATTATTTTTTTCGCACTTCTAGG

ACAACCCCGATTCCCTACCCTAGTTAATATTAACGAAAACAACCCCCTTC

TGATCAACTCTATCAAACGCTTACTAATTGGAAGCCTCTTCGCAGGATAC

ATCATTTCCAACAATATTCCTCCAACAACAATTCCCCAAATAACTATGCC

CTACTACCTAAAAACAACAGCCCTAATTGTTACAATCCTAGGCTTCATCT

TAGCCCTAGAAATCAGTAATATAACTAAAAATCTAAAATATCACTACCCC

TCAAACGC-CTTCAAGTTCTCAACCTTGCTAGGGTATTTCCCCACAATTA

TACATCGCCTAGCTCCATACATAAATTTATCAATAAGCCAAAAATCAGCA

TCCTCCCTTCTAGACCTAATCTGACTAGAAGCCATCCTACCAAAAACCAT

CTCACTCGCCCAAA---TAAAAGCATCTACCCTGGTCACAAACCAAAAAG

GCCTGATCAAACTATATTTCCTCTCCTTCTTAATCACAATCCTTATCAGC

ATAATCTTATTTA------------------------------ATTTCCA

CGAGTAATTTCTATAATAACCACAACACCAATTAATAAAGACCACCCAGT

TACAATAACTAATCAGGTACCATAACTGTATAAAGCCGCAATCCCTATGG

CCTCTTCACTAAAGAACCCAGAATCCCCTGTATCATAAATCACCCAATCC

CCTAAACCATTAAACTCAAACACAACCTCAA---------CTTCTTTATC

CTTTAATACATAATAGACCATAAAGAACTCCATCAACAAGCCAGTAACAA

ATGCCCCTAAAACAGCCTTATTAGAAAGCCAAATTTCAGGATACTGTTCT

GTAGCCATAGCCGTTGTATAACCAAAAACTACCATCATACCTCCCAAATA

AATTAAAAAGACCATCAACCCCAAAAAGGATCCACCAAAATTCAATACAA

TTCCACAGCCAACCCCACCACTCACAATTAACCCTAACCCCCCATAAATA

GGTGAAGGTTTCGAAGAAAACCCCACAAAACCTATCACGAAAATAACGCT

TAGAATAAATACAATGTATAGTATCATTATTCTTA-CATGGAATCTA-AC

CATGACTAATGATATGAAAAACCATCGTTGT-CATTCAACTACAAGAACA

C-----TAATGACTAACATTCGAAAGTCCCACCCACTAATAAAAATTGTA

AACAATGCATTCATCGACCTTCCAGCCCCATCAAACATTTCATCATGATG

AAATTTCGGTTCCCTCCTGGGAATCTGCCTAATCCTACAAATCCTCACAG

GCCTATTCCTAGCAATACACTACACATCCGACACAACAACAGCATTCTCC

TCTGTTACCCATATCTGCCGAGACGTGAACTACGGCTGAATCATCCGATA

CATACACGCAAACGGAGCTTCAATGTTTTTTATCTGCTTATATATGCACG

TAGGACGAGGCTTATATTACGGGTCTTACACTTTTCTAGAAACATGAAAT

ATTGGAGTAATCCTTCTGCTCACAGTAATAGCCACAGCATTTATAGGATA

CGTCCTACCATGAGGACAAATATCATTCTGAGGAGCAACAGTCATCACCA

ACCTCTTATCAGCAATCCCATACATCGGCACAAATTTAGTCGAATGAATC

TGAGGCGGATTCTCAGTAGACAAAGCAACCCTTACCCGATTCTTCGCTTT

CCATTTTATCCTTCCATTTATCATCATAGCAATTGCCATAGTCCACCTAC

TATTCCTCCACGAAACAGGCTCCAACAACCCAACAGGAATTTCCTCAGAC

GTAGACAAAATCCCATTCCACCCCTACTATACCATTAAGGACATCTTAGG

GGCCCTCTTACTAATTCTAGCTCTAATACTACTAGTACTATTCGCACCCG

ACCTCCTCGGAGACCCAGATAACTACACCCCAGCCAATCCACTCAACACA

CCCCCTCACATCAAACCCGAGTGATACTTCTTATTTGCATACGCAATCTT

ACGATCAATCCCCAACAAACTAGGAGGAGTACTAGCCCTAGCCTTCTCTA

TCCTAATTCTTGCTCTAATCCCCCTACTACACACCTCCAAACAACGAAGC

ATAATATTCCGACCACTCAGCCAATGCCTATTCTGAGCCCTAGTAGCAGA

CCTACTGACACTCACATGAATTGGAGGACAACCAGTCGAACACCCATATA

TCACCATCGGACAACTAGCATCTGTCCTATACTTTCTCCTCATCCTAGTG

CTAATACCAACGGCCGGCACAATCGAAAACAAATTACTAAAATGAAGACA

G-GTCTTTGTAGTACAT---CTAATATACTGGTCTTGTAAACCAGAGAAG

GAGAACAACTAACCTCCCTAAGAC------TCAAGGAAGAAACTGCA-GT

CTCACCATCAACCCCCAAAGCTGAAGTTCTA--TTTAAACTATTCCCTG-

--------------------------------------------------

--------------------------------------------------

--------------------------------------------------

--------------------------------------------------

--------------------------------------------------

--------------------------------------------------

--------------------------------------------------

--------------------------------------------------

--------------------------------------------------

--------------------------------------------------

--------------------------------------------------

--------------------------------------------------

--------------------------------------------------

--------------------------------------------------

--------------------------------------------------

--------------------------------------------------

--------------------------------------------------

--------------------------------------------------

--------------------------------------------------

--------------------------------------------------

--------------------------------------------------

--------------------------------------------------

--------------------------------------------------

------

>Bos_primigenius_indicus Bos indicus mitochondrion, complete genome.

ATTGATGTAGCTTAACC---------CAAAGCAAGGCACTGAAAATGCCT

AGATGAGTCTCCC--AACTCCATAAACACATA--GGTTTGGTCCCAGCCT

TCCTGTTAACTCTTAATAAACTTACACATGCAAGCATCTACACCCCAGTG

AGAATGCCCT-CTAGGTTGTTAAA------ACTAAGAGGAGCTGGCATCA

AGCACAC----ACCCTGTAGCTCACGACGCCTTGC-TTAACCACACCCCC

ACGGGAAACAGCAGTGACAAAAATTAAGCC-ATAAACGAAAGTTTGACTA

AGTTATATTA----ATTAGGGTTGGTAAATCTCGTGCCAGCCACCGCGGT

CATACGATTAACCCAAGCTAACAGGAGTAC-GGCGTAAAATGTGTTAAAG

CA-------CCACACCAAATAGGGTTAAATTCTAACCAAGCTGTAAAAAG

CCATGATTAA-AATAAAAATAAATGACGAAAGTGACCCTA----CAGTAG

CCGACGCACTATAGCTAAGACCCAAACTGGGATTAGATACCCCACTATGC

TTAGCCCTAAACACAGATAATTACAT-AAACAAAATTATTCGCCAGAGTA

CTACTAGCAACAGCTTAAAACTCAAAGGACTTGGCGGTGCTTTATATCCT

TCTAGAGGAGCCTGTTCTATAATCGATAAACCCCGATAAACCTCACCAAT

TCTTGCTAATA-CAGTCTATATACCGCCATCTTCAGCAAACCCTAA-AAA

GGAAAAAAAGTAAGCGTAATTATGAT--ACATAAAAACGTTA-GGTCAAG

GTGTAACCTATGAAATGGG-AAGAAATGGGCTACATTCTCTACACTAAGA

GAAT---------CAAGCACGAAAGTTATTATGAAACCAA---TAACCAA

AGGAGGATTTAGCAGTAAACT-AAGAATAGAGTGCTTAGTTGAATTA-GG

CCATGAAGCACGCACACACCGCCCGTCACCCTCCTCAAATAGATTCAGTG

CATCTAACCCTATTTAAACGCACTAGCT-----------ACATGAGAGGA

GACAAGTCGTAACAAGGTAAGCATACTGGAAAGTGTGCTTGGATAAATCA

AGATATAGCTTA-----AACAAAGCATCCAGTTTACACCTAGAAGACTTC

A-TTCATTATGAATATCTTGAACT---AGACCTAGCCCAAAGATATCCT-

-CTCGACTAAACAACTAAGATAGAATAAAACAAAACATTTAATCCCAA--

-TTTAAAGTATAGGAGATAGAAATCTAAGTAC----GGCGCTATAGAGAA

AGTACCGCAAGGGAACGATGAAAGAAAAAA-CTAAAAGTATAAAAAAGCA

AAGATTACCCCTTGTACCTTTTGCATAA-TGAATTAACTAGTATAAGACT

TAACAAAACGAATTTTAGCTAAGCAGCCCGAAACCAGACGAGCTACTCAC

AAACAGTTT-ACCAAGAACTAACTCATCTATGTGGCAAAATAGTGAGAAG

ATTTGTAAGTAGAGGTGACATGCCTAACGAGCCTGGTGATAGCTGGTTGT

CCAGAAGATGAATCTAAGTTCAGCTTTAAAGATACC--AAAAATACAAAT

AAATCCCACTGTAGCTTTAAAAGTTAGTCTAAAAAGGTACAGCCTTTTAG

AA--ACGGATACAACCTTGACTAGAGAGTAA-------AATTTAACACTA

CCATAGTAGGCCTAAAAGCAGCCATCAATTAAGAAAGCGTTAAAGCTCAA

C------AACAAAAACTAAATAGATTCCAACAACAAAT--GATTAACT--

CCTAGCCCCAATACTGGACTAATCTATTATAGAAT-AGAAGCAATAATGT

TAACATGAGTAACAAGAAAAAT--TTTCTCCTTGCATAAGTCTAAGTCAG

TGCCTGATAATACTCTGACCACTAACAGTCAATAAA------AATAATCC

AACAATAAACAATTTATTGATTATACTGTTAACCCAACACAGGAGTGCA-

--TCTAAGGAAAGATTAAAAGAAGTAAAAGGAACTCGGCAAACACAAACC

CCGCCTGTTTACCAAAAACATCACCTCCAGCATTCCCAGTATTGGAGGCA

TTGCCTGCCCAGTGACAAC-----TGTTTAACGGCCGCGGTATCCTGACC

GTGCAAAGGTAGCATAATCATTTGTTCTCTAAATAAGGACTTGTATGAAT

GGCCGCACGAGGGTTTTACTGTCTCTTACTTCCAATCAGTGAAATTGACC

TTCCCGTGAAGAGGCGGGAATGCACAAATAAGACGAGAAGACCCTATGGA

GCTTTAACTAACCAACCCAAAGAGAATAAATTTAACCATT---------A

AGGAGTAACAACAATCTCCA--TGAGTTGGTAGTTTCGGTTGGGGTGACC

TCGGAGAATAAAAAACCCTCCGAGCGATTTTAAAGACTAGACCC-ACAAG

TCAAA----TCACTCTATCGCTCATTGATCCAAAA----ACTTGATCAAC

GGAACAAGTTACCCTAGGGATAACAGCGCAATCCTATTCAAGAGTCCATA

TCGACAAT-AGGGTTTACGACCTCGATGTTGGATCAGGACATCCTGATGG

TGCAACCGCTATCAAAG-GTTCGTTTGTTCAACGATTAA-AGTCCTACGT

GATCTGAGTTCAGACCGGAGTAATCCAGGTCGGTTTCTATCTATTAC--G

TATTTCTCCCAGTACGAAAGGACAAGAGAAATAAGGCCAACTTTAAATTA

AGCGCCTTAAGA-CAACCAATGACAGCA-TCTCAATC-AACAACACAAAA

C--------CCTGCCCTAGAACAGGG--CTTAGTTAAGGTGGCAGAGCCC

GGTAA-TTGCGTAAAACTTAAACTTTTATACCCAGAGATTCAAATCCTCT

CCTTAACAA-----------------------------------------

-----------------------------------------AATGTTCAT

AATTAACATCTTAATACTAATTATTCCTATCCTATTAGCCGTAGCATTCC

TTACGTTAGTGGAACGAAAAGTTCTAGGCTATATACAACTCCGAAAAGGT

CCAAATGTCGTAGGTCCATATGGCCTACTTCAGCCCATCGCCGATGCAAT

CAAACTTTTCATTAAAGAACCACTACGACCCGCTACATCTTCAGCCTCAA

TATTTATCCTAGCACCCATCATAGCTCTAGGCCTAGCCTTAACCATATGA

ATTCCCCTACCAATACCCTACCCTCTTATCAACATAAACCTAGGAGTCCT

ATTTATACTAGCCATATCAAGCCTAGCCGTGTACTCCATTCTCTGATCAG

GCTGAGCTTCCAACTCAAAATACGCACTAATCGGAGCCCTACGAGCAGTA

GCACAAACAATCTCATACGAAGTAACACTAGCAATTATCCTATTATCAGT

ACTCCTAATAAGTGGGTCCTTTACCCTCTCCACATTAATTATTACACAAG

AACAAATATGGTTAATCCTCCCAGCATGGCCTCTAGCAATAATATGATTT

ATCTCAACACTAGCAGAAACAAACCGAGCTCCATTTGATTTAACTGAAGG

AGAATCAGAGCTAGTCTCGGGCTTCAACGTAGAATATGCAGCAGGACCAT

TTGCCCTCTTCTTCATAGCAGAGTACGCAAATATCATCATAATAAATATC

TTTACAGCAATTTTATTCCTGGGAACATCCCACAATCCACACATACCAGA

ACTCTACACAATCAACTTTACCATTAAATCCCTACTGCTCACAATATCCT

TCCTATGAATCCGAGCATCCTATCCTCGATTTCGCTATGACCAACTAATA

CACTTACTATGAAAAAACTTTCTACCCCTGACACTAGCCCTATGCATGTG

ACACGTATCCCTACCCATCCTTACATCAGGCATCCCACCACAAACATAA-

GAAATATGTCTGACAAAAGAGTTACTTTGATAGAGTAAATAATAGAGGTT

CA-AACCCTCTTATTTCTAGAACTATAGGAATCGAACCTACTCCTAAGAA

TCCAAAACTCTTCGTGCTCCCAAT--TACACCAAATTCTA-------TTA

GTAAGGTCAGCTAA-TTAAGCTATCGGGCCCATACCCCGAAAATGTTGGT

TTATATCCTTCCCGTACTAATAAACCCAATTATCTTTATTATTATTCTAC

TAACCATTATACTAGGAACTATTATTGTCATGATCAGTTCTCACTGACTA

CTTGTCTGAATCGGGTTTGAAATAAATATACTCGCCATCATCCCCATCAT

AATAAAAAATCACAACCCACGAGCTACAGAAGCATCAACTAAATACTTTT

TGACTCAATCAACAGCCTCAATACTACTAATAATAGCCGTCATCATTAAC

CTAATATTCTCAGGCCAATGAACCGTAATAAAACTATTTAACCCAATAGC

CTCAATACTTATAACAATAGCCCTAGCTATAAAACTAGGAATAGCCCCAT

TTCACTTCTGAGTCCCAGAAGTAACACAGGGCATCCCCCTATCCTCAGGC

CTTATCCTACTGACATGACAAAAACTAGCACCTATATCTGTACTTTACCA

AATCTTCCCATCAATTAACCTAAACTTAATTCTGACTCTATCAGTTTTAT

CAATCCTAATTGGAGGCTGAGGAGGACTAAACCAAACACAACTCCGAAAA

ATCATAGCCTACTCATCAATCGCTCATATAGGCTGAATAACAGCAGTACT

ACCATATAACCCCACCATAACATTACTAAACTTAATTATCTATATCATTA

TAACTTCCACCATATTTACCATATTTATAGCCAATTCCACTACCACTACC

CTGTCATTATCACACACATGAAATAAAACACCCATTATAACAGTCCTAAT

TCTTGCCACTCTCCTATCCATAGGAGGACTCCCTCCCCTATCTGGGTTTA

TACCAAAATGAATAATCATCCAAGAGATAACAAAAAATAACAGCATCATT

CTACCCACTTTCATAGCAATCACAGCTCTACTAAACTTATATTTTTATAT

ACGACTCACGTATTCTACCACACTAACAATATTTCCCTCCACAAACAACA

TAAAAATAAAATGACAATTTCCCCTTATGAAAAAAATAACTTTTCTACCA

ACAATAGTCGTATTATCTACCATAATACTACCACTCACACCAATACTATC

AGTGTTAGAA-------TAGGAATTTAGGTTAAA----CAGACCAAGAGC

CTTCAAAGCCCTAAGCAAGTAC----AATTTACTTAATTCCTGA------

---------TAAGGATTGCAAGACTACACCTTACAT----CAATTGAATG

CAAATCAACCACTTTAA---TTAAGCTAAATCCTCA-----------CTA

GACTGGT---GGGCTCCACCCCCAC-GAAACTTTAGTTAACAGCTAAACA

CCCTAGTTAACTGGCTTCAATCTACT--TCTCCCGCCGTAAGAAAAAAAA

-----GGCGGGAGAAGCCCCGGCAGAATT---GAAGCTGCTTCTCT----

---------------------------GAATTTGCAATTCAACGTGTAAA

T---TCACCACAGAGCTT------GGTAAAAAGAGGAG----TCAAACCT

CTATCTTTAGATTTACAGTCTAATGCTT-TGCTCAGCCATTTTACC----

-------CATGTTCATTAACCGCTGACTATTCTCAACCAACCATAAAGAT

ATTGGTACCCTTTACCTACTATTTGGTGCTTGGGCCGGTATAGTAGGAAC

AGCTTTAAGCCTTCTAATTCGCGCTGAATTAGGCCAACCCGGAACTCTGC

TCGGAGACGACCAAATCTACAACGTAGTTGTAACCGCACACGCATTTGTA

ATAATCTTCTTTATAGTAATACCAATCATAATTGGAGGGTTCGGTAACTG

ACTTGTTCCCCTAATAATTGGTGCTCCCGATATAGCATTTCCCCGAATAA

ATAATATAAGCTTCTGACTTCTCCCTCCCTCATTCCTACTACTCCTCGCA

TCCTCTATAGTTGAAGCTGGGGCAGGAACAGGCTGAACCGTGTACCCTCC

CTTAGCAGGCAACCTAGCCCATGCAGGAGCTTCAGTTGATCTAACCATTT

TCTCTTTACACTTAGCAGGAGTTTCCTCAATTTTAGGAGCCATCAACTTC

ATTACAACAATTATCAACATAAAGCCCCCCGCAATGTCACAATACCAAAC

CCCTCTATTCGTATGATCCGTAATAATTACCGCCGTACTACTACTACTCT

CGCTCCCTGTATTAGCAGCCGGCATCACAATGCTATTAACAGACCGGAAC

CTAAATACAACTTTCTTCGACCCGGCAGGAGGAGGAGATCCTATTCTATA

CCAACACTTATTCTGATTCTTTGGACACCCCGAAGTCTATATTTTAATCT

TACCTGGATTTGGAATAATCTCTCATATCGTAACCTACTACTCAGGAAAA

AAAGAACCATTCGGATATATGGGAATAGTTTGGGCTATAATGTCAATCGG

ATTTCTAGGTTTCATCGTATGAGCCCACCATATATTCACTGTCGGAATAG

ACGTCGACACACGAGCCTACTTCACATCAGCCACTATAATTATTGCTATT

CCAACCGGGGTAAAAGTCTTCAGCTGATTGGCAACACTTCATGGAGGTAA

TATCAAATGGTCTCCTGCTATAATGTGAGCCCTAGGCTTTATTTTCTTGT

TTACAGTAGGGGGTTTAACTGGAATTGTCTTAGCCAACTCTTCTCTCGAT

ATTGTTCTTCACGACACATACTACGTTGTCGCACATTTCCACTATGTTTT

ATCAATAGGAGCTGTATTTGCTATTATAGGGGGATTTGTTCATTGATTCC

CATTATTCTCAGGTTATACTCTCAACGATACATGAGCCAAAATTCACTTC

GCAATTATATTTGTAGGCGTCAATATAACCTTCTTCCCACAACACTTTCT

AGGACTATCTGGCATGCCTCGACGATACTCCGACTACCCAGATGCATACA

CAATATGAAATACTATCTCATCAATAGGCTCATTCATTTCCCTAACAGCA

GTTATACTAATAGTTTTCATCATCTGAGAAGCATTTGCATCTAAACGAGA

AGTCTTGACTGTAGACTTAACCACGACAAATCTAGAATGATTAAACGGAT

GCCCTCCACCATATCACACATTTGAAGAACCCACCTATGTTAACCTAAAA

------------------TAAGAAAGGAAGGAATCGAACCCCCTACTATT

GGTTTCAAGCCAACATCATAACCTCTATGTCTCTCTC----AATAAATGA

GGTGTTAGTAAAAC--ATTATATAACTTTGTCAAAGTTAAGTTACAAGTG

A------GAATCTTGTACACCTCATATGGCATATCCCATACAACTAGGAT

TCCAAGATGCAACATCACCAATCATAGAAGAACTACTTCACTTTCATGAC

CACACGCTAATAATTGTCTTCTTAATTAGCTCATTAGTACTTTACATTAT

TTCACTAATACTAACAACAAAGCTGACCCATACAAGCACGATAGATGCAC

AAGAAGTAGAGACAATCTGAACCATTCTGCCCGCCATCATCTTAATTCTA

ATTGCTCTTCCTTCTTTACGAATTCTATACATAATAGATGAAATCAATAA

CCCATCTCTTACAGTAAAAACCATAGGACATCAGTGATACTGAAGCTATG

AGTATACAGATTATGAGGACTTAAGCTTCGACTCCTACATAATTCCAACA

TCAGAATTAAAGCCAGGGGAGCTACGACTATTAGAAGTCGATAATCGAGT

TGTACTACCAATAGAAATAACAATCCGAATGCTAGTCTCCTCTGAAGACG

TACTACACTCATGAGCTGTGCCCTCTCTAGGACTAAAAACAGATGCAATC

CCAGGCCGTCTAAACCAAACAACCCTTATATCGTCCCGTCCAGGCTTATA

TTACGGTCAATGCTCAGAAATTTGCGGGTCAAACCACAGTTTCATACCCA

TTGTCCTTGAGTTAGTCCCACTAAAGTACTTTGAAAAATGATCTGCATCA

ATATTATAAAA---------------------------------TCACTA

AGAAGCTAT---ATAGCACTAACCTTTTAAGTTAGAGATTGAGAGCC---

ATATACTCTCCTTGGTGACATGCCGCAACTAGACACGTCAACATGACTGA

CAATGATCCTATCAA-TATTCTTGACCCTCTTTATTATCTTTCAACTAAA

AATTTCAAAACA---CAACTTTTATCACAATCCAGAACTGACACCAACAA

AAATATTAAAACAAAACACCCCTTGAGAAGCAAAATGAACGAAAATTTAT

TTGCCTCTTTTATTACCCCTGTAATTTTAGGTCTCCCTCTCGTAACCCTT

ATCGTACTATTCCCCAGCCTACTATTCCCAACATCAAACCGACTAGTAAG

CAATCGCTTTGTAACCCTCCAACAATGAATACTTCAACTTGTA-TCAAAA

CAAATAATGAGCATCCACAATTCTAAAGGACAAACATGAGCATTAATACT

AATATCTCTAATCCTATTTATTGGATCAACAAACCTACTAGGCCTATTAC

CCCATTCATTCACACCGACAACACAACTATCAATAAACCTAGGCATAGCC

ATCCCCCTGTGAGCAGGAGCCGTAATTACAGGATTCCGCAATAAAA-CTA

AAGCATCACTTGCCCATTTCTTACCACAAGGAACACCCACTCCACTAATC

CCAATACTAGTAATTATTGAAACTATCAGCCTTTTTATTCAACCTGTAGC

CCTCGCCGTGCGGTTAACAGCTAACATCACTGCAGGACACCTATTAATTC

ACCTAATCGGAGGAGCTACACTTGCACTAATAAGCATTAGCACTACAACA

GCTCTAATTACATTCACCATTCTAATCCTACTAACAATTCTAGAGTTTGC

AGTAGCTATAATCCAAGCCTATGTATTCACTCTCCTAGTCAGCCTATATC

TGCATGACAACACATAATGACACACCAAACCCATGCTTATCATATAGTAA

ATCCAAGCCCTTGACCTCTTACAGGAGCTTTGTCCGCCCTCTTAATAACA

TCCGGCCTAACCATATGATTTCACTTTAACTCAATGACCCTGCTAATAAT

TGGCCTAACAACAAATATACTAACAATATACCAATGATGACGAGATGTTA

TCCGAGAAAGCACCTTCCAAGGGCACCATACCCCAGCTGTCCAAAAAGGC

CTCCGTTATGGAATAATTCTTTTTATTATCTCCGAAGTACTGTTCTTTAC

CGGATTTTTCTGAGCTTTCTACCACTCAAGCCTCGCCCCCACCCCTGAGC

TAGGCGGCTGCTGACCCCCAACAGGCATTCACCCACTAAACCCCCTAGAA

GTCCCACTGCTCAACACCTCTGTCCTATTGGCTTCCGGAGTTTCTATTAC

CTGAGCCCATCATAGTTTAATAGAAGGGGACCGAAAGCATATATTACAAG

CCCTATTTATCACCATCACATTAGGAATCTACTTCACACTACTACAAGCC

TCAGAATACTATGAAGCACCTTTTACTATCTCCGACGGAGTTTACGGCTC

AACTTTTTTTGTAGCCACAGGCTTCCATGGCCTCCACGTCATCATTGGAT

CCACCTTCTTAATTGTCTGCTTCTTCCGCCAATTAAAATTTCATTTTACT

TCTAACCACCACTTCGGCTTTGAAGCCGCTGCCTGATACTGACATTTCGT

AGACGTAGTCTGACTTTTCCTCTATGTTTCTATCTATTGATGAGGCTCCT

ATTCT-TTTAGTATCAA-CTAGTACAGCTGACTTCCAATCAGCTAGTTTC

GGT-CTAGTCCGAAAAAGAATAATAAATTTAATACTAGCCCTCCTGACCA

ATTTTACACTAGCCACCCTACTCGTCATCATCGCATTCTGGCTTCCCCAA

CTAAATGTATACTCTGAGAAAACAAGCCCCTACGAATGTGGATTTGACCC

CATAGGATCAGCCCGCCTTCCCTTCTCCATAAAATTCTTTCTGGTAGCCA

TCACATTCCTCTTATTTGACCTAGAAATTGCACTCCTCTTACCACTGCCA

TGAGCCTCACAAACAACAAACCTAAACACAATGCTTACCATAGCCCTCTT

CCTAATTATCCTCCTAGCTGTAAGCCTAGCCTATGAATGAACTCAAAAAG

GATTAGAATGAACCGAATA------TGGTACTTAGTTTAAAATAAAA-TA

AATGATTTCGACTCATTAGATTATGATTTAATT-CATAATTACCAA---A

TGTCTATAGTATACATAAACATTATAATGGCATTCACAGTATCTCTTGTA

GGACTACTAATATACCGATCCCACCTAATATCTTCCCTTCTGTGCTTAGA

AGGAATAATGCTATCCCTATTCGTTATAGCAGCCCTAACAATCCTCAACT

CACATTTTACATTAGCTAGCATAATACCTATTATCCTACTAGTCTTCGCA

GCCTGCGAAGCAGCCCTAGGTCTATCTCTACTAGTAATAGTATCAAATAC

ATATGGTACTGATTATGTACAAAACCTCAACTTACTCCAATGCTAAAATA

CATTATTCCAACAATTATACTTATACCCCTAACCTGGTTATCAAAAAATA

GTATAATTTGGGTTAACTCCACAGCACACAGTCTTCTAATTAGCTTTACA

AGCCTCCTCCTCATAAACCAGTTTGGCGACAACAGCCTTAATTTTTCACT

AGTATTTTTCTCCGACTCCCTATCCACTCCACTACTAATTTTAACCATAT

GGCTCCTCCCTCTAATACTAATAGCTAGCCAACATCATCTATCAAAAGAA

AACCTAACCCGAAAAAAACTATTTATTACTATGCTGATCTCACTACAACT

ATTCCTAATCATAACCTTTACCGCCATGGAACTAATCTTATTTTATATTC

TATTTGAAGCAACACTAGTCCCAACACTCATTATTATTACCCGATGAGGA

AACCAAACAGAACGCCTAAACGCCGGACTCTATTTCCTATTCTATACACT

AGCTGGCTCCTTACCCCTATTAGTCGCACTAATTTATATCCAAAATACAG

TAGGATCCCTAAATTTCCTAATATTACAATACTGAGTACAACCTGTTCAT

AACTCT----------TGATCTAATGTCTTCATATGACTAGCATGTATAA

TAGCCTTCATAGTAAAAATACCACTATATGGCCTCCACCTTTGACTACCT

AAAGCTCACGTAGAAGCCCCTATCGCAGGCTCCATAGTCCTTGCAGCAGT

TCTACTAAAACTAGGGGGGTACGGTATGCTACGAATTACACTAATTCTAA

ACCCTATGACCGACTTTATAGCATACCCATTCATTATACTCTCCCTATGG

GGCATAATTATAACCAGCTCAATCTGCCTCCGTCAAACGGACCTAAAATC

ACTCATCGCATACTCCTCTGTAAGCCATATAGCACTCGTCATCGTAGCCA

TCCTTATCCAGACACCTTGAAGCTACATAGGAGCAACCGCCCTTATGATT

GCCCACGGCCTCACATCCTCCATACTTTTCTGTCTAGCAAACTCAAACTA

CGAACGAATCCACAGCCGAACCATAATTCTAGCTCGAGGCCTACAAACGC

TCCTTCCACTAATAGCCACCTGATGACTACTAGCAAGTCTAACCAACTTA

GCTCTACCCCCAACAATCAACTTAATTGGAGAACTATTTGTAGTAATGTC

AACCTTTTCATGATCTAACATTACAATTATTCTAATAGGAGTAAATATAG

TAATCACCGCCCTATATTCTCTATACATGCTAATTATAACCCAACGAGGA

AAATATACCTACCACATTAATAACATCTCGCCTTCCTTTACACGGGAAAA

TGCACTCATATCGTTACACATCCTACCCCTACTACTCCTAACCCTAAACC

CAAAAATTATTCTAGGACCTCTATACTGTAAATATAGTTTAACAAAAACA

TTAGATTGTGAATCTAACAATAGAAACTCATTA-CCTTCTTATTTACCGA

AAAAGTAT---GCAAGAACTGCTAATTCTATGCTCCCATATCTAATAGTA

TGGCTT--------------------------------------------

-------------------TTTCGAACTTTTAAAGGATAGTAGTTTATCC

GTTGGTCTTAGGAACCAAAAA--ATTGGTGCAACTCCAAATAAAAGTAAT

AAACA---TATTCTCCTCACTCTCACTGGTTACTTTACTCTTACTAACTA

TACCCATTATAATAATAAGCCTTAACACCTACAAACCTTCCAACTACCCA

CTCTACGTAAAAACAGCTATCTCATATGCCTTCATTACCAGCATAATTCC

CACAATAATATTTATCCACTCAGGCCAAGAACTAATTATTTCAAACTGAC

ACTGACTAACCATCCAAACTCTTAAATTATCCCTCAGCTTTAAAATAGAC

TATTTCTCAATAATATTTACCCCAGTAGCACTATTCGTCACATGATCTAT

TATAGAATTCTCAATATGATATATACACTCAGACCCCAATATTAACAAAT

TCTTCAAATACTTACTCCTATTCCTCATTACTATGCTCATCCTTGTAACC

GCAAATAACCTCTTCCAGCTATTCATTGGCTGAGAAGGCGTCGGAATCAT

ATCATTTCTACTCATCGGATGATGATACGGACGAGCAGATGCAAACACAG

CAGCCCTACAAGCAGTCTTATATAACCGCATCGGCGACATTGGTTTCATT

TTAGCAATAGCATGATTTCTAACAAACCTCAATACCTGAGACCTCCAACA

GATCTTCATACTAAACCCAAGCGACTCAAACATACCCTTGATCGGACTAG

CATTAGCTGCAACCGGAAAATCCGCCCAATTTGGCCTCCACCCATGACTT

CCCTCTGCAATAGAAGGCCCAACTCCCGTCTCAGCACTACTCCATTCAAG

CACAATAGTGGTAGCAGGTATCTTCCTACTAATCCGTTTCTACCCCCTCA

CA-GAAAACAATAAATTTATCCAATCTATTACATTATGCTTAGGAGCCAT

TACCACACTATTTACAGCAATATGCGCCCTCACCCAAAATGACATTAAGA

AAATCATCGCCTTCTCCACATCCAGTCAACTGGGCCTTATAATAGTAACA

ATTGGCATTAACCAACCTTACCTAGCTTTCCTCCACATCTGCACACATGC

CTTTTTCAAAGCTATACTATTCATATGCTCCGGTTCCATTATTCACAGCC

TAAACGACGAACAAGATATTCGAAAAATAGGAGGCCTATTTAAAGCCATG

CCATTCACCACAACAGCCCTCATTGTTGGCAGTCTCGCACTAACAGGAAT

ACCCTTCCTCACAGGATTTTACTCCAAAGACCTAATCATCGAAGCCGCCA

ACACGTCTTATACCAACGCCTGAGCCCTTCTAATAACATTAATTGCCACC

TCTTTCACAGCTATCTACAGCACTCGTATTATTTTTTTCGCACTTCTAGG

ACAACCCCGATTCCCTACCCTAGTTAGTATCAACGAAAACAACCCCCTTC

TGATCAACTCTATCAAACGCTTACTAATTGGAAGCCTCTTCGCAGGATAC

ATCATTTCCAACAATATTCCTCCAACAACAATTCCCCAAATAACTATACC

CTACTACTTAAAAACAACAGCCCTAATCGTTACAATCCTAGGCTTCATCT

TAGCCCTAGAAATCAGTAATACAACTAAAAATCTAAAATATCACTACCCC

TCAAACGC-CTTCAAGTTCTCGACCTTGCTAGGATACTTCCCCACAATTA

TACATCGCCTAGCTCCATACATAAATTTATCAATAAGCCAAAAATCAGCA

TCCTCCCTTCTAGACCTAATCTGACTAGAAGCCATCCTACCAAAAACCAT

CTCACTCGCCCAAA---TAAAAGCATCTACCCTGGTCACAAACCAAAAAG

GCCTGATCAAACTATATTTCCTCTCCTTCCTAATCACAATCCTTATCAGC

ATAATACTATTTA------------------------------ATTTCCA

CGAGTAATTTCTATAATAACCACAACACCAATTAATAAAGACCACCCAGT

TACAATAACTAATCAGGTACCATAACTGTATAAAGCCGCAATCCCTATGG

CCTCTTCACTAAAAAACCCAGAATCCCCTGTATCATAAATCACTCAATCC

CCTAAACCATTAAACTCAAACACAACCTCAA---------CTTCTTTATC

CTTTAACACATAATAAACCATAAAAAACTCCATCAACAAGCCAGTAACAA

ATGCCCCTAAAACAGCCTTATTAGAAAGCCAAATTTCAGGATACTGTTCT

GTAGCCATAGCCGTTGTATAACCAAAAACTACCATCATACCCCCCAAATA

AATTAAAAAGACCATCAACCCCAAAAAGGATCCACCAAAATTCAATACAA

TCCCACAGCCAACCCCACCACTCACAATTAACCCTAACCCCCCATAAATA

GGTGAAGGTTTCGAAGAAAACCCCACAAAACCTATCACGAAAATAACACT

TAAAATAAATACAATGTATAGTATCATTATTCTTA-CATGGAATCTA-AC

CATGACTAATGATATGAAAAACCATCGTTGT-CATTCAACTATAAGAACA

C-----TAATGACTAACATTCGAAAGTCCCACCCACTAATAAAAATTGTA

AACAATGCATTCATCGACCTTCCAGCCCCATCAAACATTTCATCATGATG

GAATTTCGGTTCCCTCCTGGGAATCTGCCTAATCCTACAAATCCTCACAG

GCCTATTCCTAGCAATACACTACACATCCGACACAACAACAGCATTCTCC

TCTGTTACCCATATCTGCCGAGACGTGAACTACGGCTGAATCATCCGATA

CATACACGCAAACGGAGCTTCAATGTTTTTTATCTGCTTATATATGCACG

TAGGACGAGGCTTATATTATGGGTCTTACACTTTTCTAGAAACATGAAAT

ATCGGAGTAATCCTTCTGCTCACAGTAATAGCCACAGCATTCATAGGATA

CGTCCTACCATGAGGACAAATATCATTCTGAGGAGCAACAGTCATCACCA

ACCTCTTATCAGCAATCCCATACATCGGCACAAATTTAGTCGAATGAATC

TGAGGCGGATTCTCAGTAGACAAAGCAACCCTTACCCGATTCTTCGCTTT

CCATTTTATCCTTCCATTTATCATCATAGCAATTGCCATAGTCCACCTAT

TATTCCTCCACGAAACAGGCTCCAACAATCCAACAGGAATCTCCTCAGAC

GTAGACAAAATCCCATTCCACCCCTACTATACCATTAAGGACATCTTAGG

GGCCCTCTTACTAATTCTAGCTCTAATACTACTAGTACTATTCGCACCCG

ACCTCCTCGGAGACCCAGATAACTACACCCCGGCCAATCCACTCAACACA

CCTCCTCACATCAAACCCGAATGATACTTCTTATTTGCATACGCAATCTT

ACGATCAATCCCCAACAAACTAGGAGGAGTACTAGCCCTAGCCTTCTCTA

TCCTAATTCTTGCTCTAATCCCCCTACTACACACCTCCAAACAACGAAGC

ATAATATTCCGACCACTCAGCCAATGCCTATTCTGAGCCCTAGTAGCAGA

CCTACTGACACTCACATGAATTGGAGGACAACCAGTCGAACACCCATATA

TCACCATTGGACAACTAGCATCTATCCTATATTTTCTTCTCATCCTAGTA

CTAATACCAACAGCCGGCACAGTTGAAAACAAATTACTAAAATGAAGACA

G-GTCTTTGTAGTACAT---CTAATATACTGGTCTTGTAAACCAGAGAAG

GAGAACAACTAACCTCCCTAAGAC------TCAAGGAAGAAACTGTA-GT

CTCACCGTCAACCCCCAAAGCTGAAGTTCTA--TTTAAACTATTCCCTG-

--------------------------------------------------

--------------------------------------------------

--------------------------------------------------

--------------------------------------------------

--------------------------------------------------

--------------------------------------------------

--------------------------------------------------

--------------------------------------------------

--------------------------------------------------

--------------------------------------------------

--------------------------------------------------

--------------------------------------------------

--------------------------------------------------

--------------------------------------------------

--------------------------------------------------

--------------------------------------------------

--------------------------------------------------

--------------------------------------------------

--------------------------------------------------

--------------------------------------------------

--------------------------------------------------

--------------------------------------------------

--------------------------------------------------

------

>Marmota_himalayana Marmota himalayana mitochondrion, complete genome.

GTTAATGTAGCTTAA--TC----TG-TAAAGCAAAGCACTGAAAATGCTT

AGACGGGTACTTA-T-ACCCCATAAACACATA--GGTTTGGTCCTGGCCT

TTTCATTAGCTTCTAGCTAACTTATACATGCAAGCATCCCCGTCCCAGTG

AGAATGCCCT-CTATATCTATTAAC---TGATCAAAAGGTGCAGGCATCA

AGTTCACT-AACCCTAGTAGCTCACAACGCCTTGC-TCCACCACACCCCC

ACGGGAAACAGCAGTAATTAAGATTAAGCTTATAAACGAAAGTTTGACTA

AGTTAAGCCACAAATAAAGAGTCGGTAAATTTCGTGCCAGCCACCGCGGT

CATACGATTAACTCTAGTTAATGAAAC-AC-GGCGTAAAGCGTGATTAAG

AGA------TCAAT-CAGATAAGATTAAAATAATATTAAACTGTAAAAAG

TC-TTGATAATTATGAAAATCAAATACGAAAGTAATCTTA---AATTTTC

TGAATTCACGATAGCTAAGATCCAAACTGGGATTAGATACCCCATTATGC

TTAGCCCTAAACATAAACA-TTCAAC-AAACAAGAATATTCGCCAGAGTA

CTACTAGCAATAGCCTGAAACTCAAAGGACTTGGCGGTGCTTTACACCCC

TCTAGAGGAGCCTGTTCTATAATCGATAAACCCCGATACACCTCACCACC

TTTAGCGAATATCAGCCTATATACCGCCATCTACAGCAAACCCTAA-AAA

GGCCCTACAGTAAGCAAGAAAATTTT--ACATTAGTACGTTA-GGTCAAG

GTGTAGCCTATAGGGTGGG-AAGAAATGGGCTACATTTTCTATTTTCCTA

GAATA------A-C-CCCACGATAGCTTTTATGAAACTCAG---AGCATA

AGGCGGATTTAGTAGTAAGTT-AAGAATAGAGAGCTTAACTGAATGG-GG

CAATAAAGCACGCACACACCGCCCGTCACCCTCTTCAAATATACTTC--A

AAGCAATTCTATTAATAATTTATTTCACCAAA--T--ATATA--AGAAGA

GATAAGTCGTAACAAGGTAAACATACTGGAAAGTGTGTTTGGAACAACCA

AAATGTAGCTTA--T-AATCAAAGCACCCGGCTTACACCCGAGAGATTTC

ACCT-ACTATGAACATTTTGA-ACT-AATACT-AGCCCAACTCTTC--TT

ATTCCTTTCAAATACAATACACCTAATAAATAAAACATTCATC-TGAA--

--TAAA-GTATAGGAGATAGAAATTTA--TATCT--GGAGCTATAGAGAA

AGTACCGCAAGGGAAAGA-TGAAAGAATAAACTAATAGTACTAAGAAGCA

AAGATTAACTCTTTTACCTTTTGCATAA-TGGTTTAACCAGAAAACATTT

GACAAAAAG-AATTTAAGCCAAACACCCCGAAACCAGACGAGCTACTTAT

AGGCAGCC--AATAAGAGCCTATCCGTCTATGTCGCAAAATAGTGGAACG

ACTTATAAGTAGAGGTGAAAAGCCTATCGAGCCTGGTGATAGCTGGTTGT

CCA-GACTAGAATTTAAGTTCTACTTTAAATTTACCTAAAGCACA-AAC-

AA-GCTGAATGTAAATTTAAATGTTATTCTAAAGAGGGACAGCTCTTTGG

AGTCAAGGAATTAGCCTTAACTAGAGAGTAA-----AACTATTAATT--T

CCATAGTTGGCCTAAAAGCAGCCATCAATTAAAAAAGCGTTAAAGCTTAA

TC-AACTAAT---TAAAACTTAATACCCAGCTCT---TTC-ATAATCT--

CCTAATTCAATA-CTGGACTAATCTATTTTACAAT-AGAAGAAACTATGT

TAAAATCAGTAACAAGAA-AATAT-T-CTCCCCGCATGAGCTTATATCAG

ATCGAA-TAATTCACTGATAGTTAACAACTCCATA-----ATATTAAATA

AACATAAAATCA-TTATTATTAAAATTGTTAATCCAACACTGGCATGCGC

--TTAAGGGAAAGATTAAAAAAAGTAAAAGGAACTCGGCAAACACTAACC

TCGCCTGTTTACCAAAAACATCACCTCTAGCATAAATAGTATTAGAGGCA

CTGCCTGCCCAGTGACA-CACG----TTCAACGGCCGCGGTATCCTGACC

GTGCAAAGGTAGCATAATCACTTGTTCTTTAAATAAGGACTAGCATGAAT

GGCTTAACGAGGGTTTAACTGTCTCTTACTTTTAATCAGTGAAATTGACC

TTCCCGTGAAGAGGCGGGAATTTCCTAATAAGACGAGAAGACCCTATGGA

GCTTTAATTTAAT-AGTCTCACAGCCCTAATAAAATCCT----------A

GGAATCAA-AATTATTGTTTA-CAGACTAGAAATTTTGGTTGGGGTGACC

TCGGAGTATAAATCAACCTCCGAATGATA-ATAATCT-AGACAC-ACATG

TCCAAATTATAACTC----ATTAATTGACCCAA-A---TCATTGATCAAC

GGAACAAGTTACCCTAGGGATAACAGCGCAATCCTACTCAAGAGTCCATA

TCGACAGTTAGGGTTTACGACCTCGATGTTGGATCAGGACATCCAAATGG

TGTAACCGCTATTAATG-GTTCGTTTGTTCAACGATTAA-AGTCCTACGT

GATCTGAGTTCAGACCGGAGAAATCCAGGTCGGTTTCTATCTATTATTA-

AATTTCTCCCAGTACGAAAGGACAAGAGAAATAAGGCCAATTAAACTC-T

AATGCCTTAGAATTAATGGATGAA-ACAATCTTAATCCAATAATATATTT

TAAAT--AACCTGCCCTAGAGCAGGG--CTT-GTTAAGATGGCAGAGCCT

GGTAA-TTGCGTAAGACTTAAAACTTTATA-TCAGAGGTTCAACTCCTCT

TCTTAACA-TTA--------------------------------------

------------------------------------------ATGTTTAT

AATTAACCTTCTACTTCTAATCATTCCGATTCTAGTAGCTATAGCTTTCC

TCACCTTAATCGAACGAAAAATACTAGGATATATACAACTTCGCAAAGGC

CCTAATGTTGTCGGACCTTACGGCTTACTTCAACCATTCGCTGATGCAAT

AAAATTATTTATTAAAGAACCCATAAAACCTCTAACATCATCAATTATAT

TATTTATTATCGCTCCAACCCTTGCCCTAACACTAGCATTCACCATATGA

ATTCCCCTACCTATACCCCAACCCCTCATTAATATAAATATGGGAGTCCT

ATTTATTTTAGCCACATCAAGTTTAGCTGTCTATGCAATCTTATGATCTG

GCTGAGCCTCTAACTCCAAATATGCCTTAATTGGAGCTCTGCGAGCCGTA

GCACAAACTATCTCCTATGAAGTAACACTAGCAATTATTCTCCTCTCAGT

CCTTCTAATGAATGGATCCTTCTCCCTATCTACTCTCATTATTACCCAAC

AATTCACATGACTCCTACTTCCAACATGACCTCTAGCAATAATATGATTT

ATTTCAACATTAGCAGAAACCAACCGAGCTCCATTTGACTTAACAGAAGG

GGAATCAGAACTTGTATCAGGATTTAATGTTGAATATGCAGCTGGTCCCT

TCGCCTTATTTTTTATAGCTGAATATACCAATATCATCATAATAAACGCG

TTAACAGTAACCCTCTTTATAGGAGCATTACTAAATCCTACTTCCCCTGA

AACTTTTACATTAAGCTTTACTTTAAAAACACTTATATTAACCTCTATCT

TTCTATGAATCCGAGCATCCTATCCCCGATTCCGTTATGACCAACTTATA

CATCTTCTATGAAAAAATTTTTTACCTCTAACATTAGCTTTATGCATATG

ACACATCTCTCTTCCAATCACGACTGCATGTGTACCACCTCAAACCTAA-

GAAATATGTCTGATAAAAGAGTTACTTTGATAGAGTAAATTATAGAGGTT

TA-AATCCTCTTATTTCTAGAACTATAGGGATCGAACCTAATCCTAAGAA

TCCAAAATTCTCCGTGCTACC-CTT-TACACCATGTTCTAAAC--A----

GTAAGGTCAGCTAAAT-AAGCTATCGGGCCCATACCCCGAAAATGTTGGT

TTATACCCTTCCCGTACTAATTAATCCCCTAACCTCCACCGCAATCTACC

TCACCCTCTTCTCCGGAACTATGATTACACTTTTCAGCTCACATTGACTT

CTAGCTTGAGTAGGACTAGAAATAAACATGCTAACTATTACCCCTATTCT

AATCCACAAAGGAAATCCTCGATCTACAGAAGCCACGTGTAAATATTTTC

TTATCCAAGCAACCGCATCAATAATCCTAATAATAGGCACAATAATTAAT

TTTATAGAATCAGGCCAGTGAACCCTATCCAACTCATATAATCAAATTTC

ATCATTTATACTTACCATCGCCCTCTCAATAAAAATAGGACTCGCCCCAT

TTCACCTATGAGTTCCAGAAATTACCCAAGGAATCCCACTTAAATCAGGC

TTAATCGTATTAACATGACAAAAAATTGCCCCAATCTCCATTGCATACCA

AATCGCACCCTCCATAAATTCCACACTCATACTATTTATAGGAGCCCTAT

CAATCATACTAGGAGGCTGAGGAGGACTTAACCAAACCCAACTACGGAAG

ATCCTGGCATACTCATCAGTCGCCCATATAGGATGAATAATAGCAATCAT

TACATATAATCCAACCTTAACAATATTTAACCTAATCATTTATATTATAC

TTACTATTAACATATTTATGCTTCTCCTTTATTATAAAAAAACTACTACC

CTTTCCCTATCAAATTTATGAAATAAATTTCCCCTCCTAACCCCTACAGT

TTTAATTGTATTAATATCACTAGGAGGCTTACCTCCCCTATCAGGATTTA

TACCAAAATGAGTTATTCTTAAAGAACTTATTTTAAATAACAACATCATT

TTCTCTACACTTATAGCAATACTAGCACTTCTAAACCTATATTTTTACAC

TCGACTAATCTATTCAACATCTCTAACCCTATTTCCATCATTTAACAACA

CCAAAATAAAATGACAATTCGAAAATGTAAAACTTACACCTCTTTTACCC

ATCTTTATCATTATTTCTACCCTCTCCCTCCCGTTGATACCCATA-CTCT

CACTCCT--AAACT----AGGAATTTAGGTTAAT--T-TAGACCAAGGAC

CTTCAAAGTCCTAAGCAAGTA-CCCAAC---ACTTAATTCCTGC------

---A----CTAAGGACTGCAAGATCTTATCTCACAT----CAACTGAATG

CAAATCAATCACTTTAA--TT-AAGCTAAGCCCTTC-ATT------TCTA

GACTGATG--GGATTTAAA-CCCAC-AAAATCTTAGTTAACAGCTAAACG

CCTTACTCAACTGGCTTCAATCTA-CT-TCTCCCGCCGT--AAGCAAAAA

-----GGCGGGAGAAGCCCCGGCAGAG-T-TGA-AGCTGCTCCTTT--GA

-----------------------------ATTTGCAATTCAATA--TGAC

TA-TTCACCTCGGGACT-T-----GGTAAAAAGAGGG----CT-CAACCT

CTGTCTTTAGATTTACAGTCTAATGCTTG--CTCAGCCATTTTACC----

ACCTACTTATGTTCATCAACCGTTGATTCTTCTCAACTAATCATAAAGAT

ATTGGTACACTTTACCTTTTATTTGGCGCTTGAGCCGGTATAGTAGGAAC

TGCACTCAGTCTATTAATCCGAGCTGAATTAGGCCAACCTGGGACTCTAC

TAGGCGATGATCAAATTTACAATGTTATTGTTACCGCCCACGCATTTATT

ATAATTTTCTTTATAGTTATGCCGATCATAATTGGTGGATTTGGGAATTG

ACTAGTACCCCTAATAATTGGAGCCCCTGATATGGCATTCCCACGTATAA

ATAACATAAGCTTCTGACTTCTCCCCCCTTCCTTCCTTCTCTTGCTCGCC

TCTTCTATAGTTGAAGCAGGTGCAGGAACAGGTTGAACCGTATATCCTCC

GCTGGCCGGAAATCTAGCCCATGCAGGGGCTTCAGTAGATCTAACTATCT

TTTCCCTTCACTTAGCAGGGGTATCATCAATTCTAGGTGCAATTAATTTT

ATTACAACAATTATTAATATAAAACCACCTGCCATATCTCAGTATCAAAC

CCCTCTATTCGTATGATCCGTACTAGTTACAGCAGTACTATTACTCTTAT

CACTTCCAGTTCTTGCAGCAGGAATTACTATACTCCTTACAGACCGTAAT

CTTAATACTACATTTTTCGATCCTGCTGGAGGTGGAGATCCCATTCTATA

CCAACACCTATTCTGATTTTTTGGACATCCTGAAGTTTACATCCTTATTC

TTCCAGGATTTGGTATAATTTCTCATATCGTAACATACTACTCAGGTAAA

AAGGAACCATTCGGTTATATAGGAATAGTCTGAGCCATAATATCCATTGG

CTTTCTTGGATTTATTGTATGAGCTCATCATATGTTTACTGTCGGAATAG

ATGTAGATACTCGAGCTTATTTTACATCTGCAACCATAATTATTGCCATC

CCTACAGGAGTAAAAGTCTTTAGCTGATTAGCAACTCTGCATGGAGGTAA

TATTAAATGATCACCAGCAATACTATGAGCACTCGGTTTCATTTTTTTAT

TCACCGTAGGAGGCTTAACAGGAATTGTCTTAGCTAACTCTTCATTAGAC

ATTGTTCTACATGATACATATTATGTTGTAGCTCACTTCCACTATGTATT

ATCAATGGGGGCTGTATTTGCCATCATAGGAGGATTCGTTCACTGATTTC

CCCTTTTTTCTGGCTATTCGCTAAATGACATATGAGCTAAAATTCATTTT

ACTGTAATGTTTGTTGGGGTAAATTTAACTTTCTTCCCTCAACACTTCCT

AGGATTATCAGGTATACCACGTCGATACTCTGACTACCCAGATGCATATA

CAGCATGGAATACTGTATCCTCAATAGGCTCATTCATCTCTCTTACAGCT

GTTATAATTATAATTTTCATAATCTGAGAAGCATTTGCATCAAAACGAGA

AGTACTTACTGTAGAGTTAATATCAACTAATTTAGAGTGACTACATGGGT

GCCCTCCACCCTATCACACATTTGAAGAACCTACTTACATTAAAATTTAG

AT--C--------------AAGAAAGGAAAGAATCGAACTTCCCAAGACT

AGTTTCAAGCTAGCCCCATAGCCACTATGA-CTTTCT---TCAT----GA

GATATTAGTAAAAT-AATTACATAACTTTGTCAAAGTTAATTTATAGGTT

A------AATTCCTATATATCTC-TATGGCATACCCACTCGAATTAGGAT

TTCAAGACGCCACATCTCCTATTATAGAAGAGCTTTTACACTTTCATGAC

CATACCCTTATAATTGTTTTCTTAATTAGCTCTCTAGTTCTTTATATTAT

TTCATTAATACTAACTACAAAATTAACTCACACAAGCACTATAGACGCCC

AAGAAGTAGAAACTATTTGAACTATTCTCCCTGCTATTATTCTTATCCTA

ATTGCTCTTCCCTCCCTACGTATTCTATATATAATAGACGAAATTAATGA

CCCATCCTTAACAGTAAAAACAATAGGCCATCAATGATATTGAAGCTACG

AGTATACAGACTATGAAGATCTTAACTTTGATTCTTACATGATTCCAACC

TCAGACTTAGCTCCAGGAGGCCTACGACTTCTAGAGGTTGATAATCGAGT

TGTACTTCCAATAGAACTACCCGTACGAATATTAATTTCATCTGAAGACG

TACTTCACTCTTGAGCAGTTCCATCCCTTGGGTTAAAAACAGATGCAATT

CCAGGCCGACTCAATCAAGCAACACTAACATCAACACGACCAGGACTATA

TTACGGGCAATGCTCTGAAATTTGTGGATCAAACCATAGCTTTATACCAA

TCGTTCTTGAACTAGTTCCGCTAAAACACTTTGAAAACTGATCCTCATCA

ATACTATAA--------------------------------ATT-CATTA

TGAAGCTA--TAGTAGCATCAACCTTTTAAGTTGAAGATTAGGAAT-T-A

A-AT-TTCCTCATAATGAAATGCCCCAACTAGATACATCTACATGATTTA

TTACAATTCTATCAA-TAATTCTAGCCCTCTTCTTTATATTTCAACTTAA

AATCTCAAATCA--CTATTATATATCTAGTCCTTCCCCTAAAGACACTAA

-ACTAATCGAGCACAAAACTCCTTGAGAAGAAAAATGAACGAAAATCTAT

TTGCCTCTTTCATTACCCCTACGTTAGTAGGTTTTCCTATTGTCCTTTTT

ATTATTATATTCCCCAACTTACTCTTCCCCTCCCCTACTCGACTAGTAAA

CAACCGTCTAGTATCATTTCAACAATGGCTAATTCAACTTGTACT-AAAA

CAAATAATGATTATACATAACCCAAAAGGACGTACCTGATCCCTAATACT

AGTTTCACTAATTATATTTATTGGCTCAACTAATCTCCTAGGTCTATTAC

CCCACTCTTTTACTCCAACTACCCAGCTATCAATAAATTTAGGAATAGCT

GTGCCTCTATGAGCAGGAGCAGTAATCACTGGATTTCGTCACAAAA-CTA

AAGCATCATTAGCCCACTTTCTCCCACAAGGAACCCCAATCCTACTTATT

CCTATGCTCATTATTATCGAAACAATTAGTCTTTTTATTCAACCTATAGC

ATTAGCCGTGCGACTAACAGCTAATATTACAGCCGGCCATCTTCTCATAC

ATTTAATCGGAGGAGCAACCCTTATACTAACATCCATTAGCCCTCCCACA

GCCATTTTAACCTTTATTATTCTTGTATTACTAACAATACTTGAATTTGC

AGTCGCATTAATTCAAGCCTACGTCTTCACTCTCCTAGTAAGCTTATATT

TACATGATAATACTTAATGACCCACCAAACCCATGCTTATCATATAGTCA

ACCCCAGCCCCTGACCCTTAACAGGAGCCCTCTCCGCCTTACTTCTAACA

TCTGGCTTGGTAATATGATTCCACTTTAACTCCTCTTTTATTCTCCTATT

AGGCCTAACAGCTAATATCCTGACAATATACCAATGATGACGAGATATTG

TACGGGAAGGCACATTTCAAGGTCACCATACATCAATTGTCCAAAAAGGC

TTACGATATGGTATAGTACTGTTTATTATCTCAGAAGTATTCTTCTTTGC

CGGGTTCTTCTGAGCGTTCTACCATTCTAGTCTAGCCCCAACTCCCGAAC

TAGGCAGCTGCTGACCTCCAGTAGGGATCAATCCACTTAATCCTCTAGAA

GTACCATTATTAAATACCTCTGTCCTTTTAGCTTCAGGAGTCTCAATTAC

TTGAGCTCACCATAGCCTAATAGAAGGAGACCGAAAGCATATAACTCAAG

CATTATCAATCACAATTGCTTTAGGACTTTACTTTACTCTTCTTCAAGCT

TCCGAGTATCTAGAAACATCCTTTACAATTTCAGATAGCGTATATGGCTC

AACATTTTTTATAGCCACAGGCTTCCATGGTCTCCATGTCATAATTGGAT

CTACCTTCCTTCTAGTATGTCTCATCCGTCAACTAAACTTTCATTTCACA

TCAAATCATCATTTTGGGTTTGAAGCAGCCGCATGATACTGACATTTCGT

AGATGTCGTATGACTCTTCCTCTATGTATCTATTTATTGATGAGGCTCAT

ATTCT-CTTAGTATCAA-TCAGTACAATTGACTTCCAATCATTTAGTTCT

GGAATTAACCCAGAAGAGAATAATAAACCTTATAGTAACTCTCCTTGTAA

ACTCTTTCATCGCCCTACTATTAATCTCTGTGGCATTCTGATTACCTCAA

CTAAACGTATATGCTGAAAAAGCAAGCCCTTATGAATGTGGTTTTGACCC

CATAGGATCTGCTCGCTTACCGTTTTCAATAAAATTTTTCCTTGTTGCAA

TCACATTCCTTCTATTTGACCTAGAGATTGCTCTTCTCCTCCCTCTTCCC

TGAGCTTCCCAAACAAATAATCTTAACCTTATATTAACTATGGCTTTACT

TTTGATCTTAATTCTTACTCTTGGACTAGCCTACGAGTGAATCCAAAAAG

GTTTAGAATGAATTGAATA------TGATAATTAGTTTAAAATAAAA-CA

AGTGATTTCGACTCACTAAATTATGGGTTA-C--CATAATTATCAA--AA

TGCCTATTATCACTCTTAATATCATATTAGCTTACTTTACATCTCTGCTA

GGAATATTCATTTACCGATCCCATCTAATATCATCACTTCTATGCTTAGA

AGGTATAATATTATCAATATTTGTCCTGTGCTCACTTCTAACTACAAGTT

TCCATTTCTCCCTATCATTTATAATTCCCATTACCTTATTAGTGTTTGCC

GCATGTGAAGCAGCTGTAGGTTTGGCCCTTCTTGTAATAGTATCCAATAC

ATATGGCCTAGACTACGTCCAAAACCTAAATATTCTTCAATGCTAAAAAT

TATTATTCCCACAATTCTACTCGCTCCCCTTATATGATTCTCAAAACCCT

CTATAATTTGAATTAATCCCTCAATTCATAGTTTAATTATTAGCCTAATT

GTTCTCCTCACATTAAACCACTCCATAAACACAGATTTAATTTTTTCATT

AGTCTTCTTTACAGACCCTCTATCTTCTCCCCTACTAATCTTAACAGCAT

GACTTCTACCCCTCATAATTATAGCAAGCCAAAGTCACTTAACTCAAGAG

CCATTAATCCGAAAAAAGCTATATATTCTTATATTAATCTTATTACAATT

CTTTCTAATTATAACTTTTTCTGCCACTGAACTAATCATATTCTATATCC

TATTTGAAGCTACCCTGATCCCTACACTTATCATTATCACTCGATGGGGA

AACCAAACTGAACGACTAAATGCAGGACTATATTTTCTATTTTATACCTT

AGTAGGTTCTCTACCCTTACTAGTAGCATTAATTTATATTCAGAAATCTA

CTGGATCTCTAAACTTTATTGTTTCAACATATCAGTCATCTGTCCTCCCC

ATATCT----------TGAACAAATCATATCCTATGACTAGCATGTACCA

TGGCTTTTATAGTTAAAATACCTCTATATGGTCTCCATCTTTGACTGCCA

AAAGCCCACGTCGAAGCTCCCATTGCCGGTTCCATGGTTTTAGCCGCCAT

TCTACTAAAACTTGGCGGATACGGAATGATCCGAATTTCAACTTTTCTAT

ATCCTATCACAAGCAATATAGCCTATCCTTTTATTTTATTATCCCTATGA

GGTATAATTATAACAAGTTCAGTCTGCTTACGACAAACAGATCTAAAATC

CCTAATCGCTTACTCGTCAGTAAGCCATATAGCGTTAGTAATTGTAGCAA

TCATAATTCAAACCCCCTGAAGCTTTATAGGAGCCACAGCATTAATAATC

GCTCACGGACTAACATCCTCCATACTATTTTGTCTAGCAAACACTAACTA

TGAACGAATTCATAGTCGAACTATAACATTAGCTCGAGGTTTACAGTCTA

TTCTTCCCCTCATAGCAACATGATGAATTCTAGCCACTCTAACCAATCTA

GCCCTTCCACCCTCCATTAACTTAATTGGTGAATTATTTATTATTATGGC

ATCGTTTACTTGATCAAATACCTCAATTATTTTAACTGGACTAAATATAC

TAATTACAGCTCTCTACTCGCTGTATATATTAATTACAACACAACAAGGA

AAACTTACATACCATACACTAAATATTAACCCTTCCTTCACACGAGAAAA

TACACTTATATTTCTTCATCTTTTTCCACTTATCATTCTATCAACGAATC

CTACCATCATTCTAGGCCAATCATATTGTAAATATAGTTTAAGCAAAACT

TTAGATTGTGAATCTAACAATAGAGAATCATAA--CCTCTTATTTACCAA

GAAAGCAC---GCAAGAACTGCTAACTCATGC-TACCGTGATTAAACTCA

CGGCTT--------------------------------------------

-------------------TCTTA-ACTCCTATAGGATAGAAGTA-ATCC

GTTGGTCTTAGGAACCAAAAA-A-TTGGTGCAACTCCAAATAGAAGTAAT

TAATA---TATTCTCCTCACTTATTCTTACATCACTTATAACCCTATCAT

TTCCTATTTTCCTAACTATAACTAACTACCATAAGCATATCAACTTCCCT

AACCACGTAAAAATCTCCATCGTTTGCGCATTATCATTCTGCATCGTACC

AACACTAATATTTATTAACTCAAATTATGAACTCATTATCTCTAACTGAC

ACTGAATGACCATTCAAACATTTACTCTTTCCATAAGCTTCAAATTAGAT

TACTTTTCCATATTATTTATACCTGTAGCACTATTCGTTACATGATCAAT

TATAGAATTCTCAATATGATATATGCACTCCGACCCTTTTATTAATCGTT

TCTTTAAATATCTCCTTTTATTCCTTATTACTATAATAATCCTAGTTACA

GCTAATAACCTATTTCAACTATTCATTGGCTGAGAAGGAGTAGGTATTAT

ATCCTTTTTACTAATCGGCTGATGATACGGCCGAACAGACGCTAATACAG

CAGCCCTCCAAGCTATTTTATATAACCGAATTGGAGATATCGGGTTTATC

CTAGCCATAGCATGATTCTTACTTAACTCAAACTCATGAGAACTACAACA

ACTTTTCACTATAGATGTCCCCCTA------TTCCCCCTACTAGGACTAC

TCTTAGCCGCCACAGGAAAATCCGCCCAATTCGGTCTTCATCCTTGACTA

CCTTCCGCCATAGAAGGTCCAACCCCCGTTTCAGCTTTACTCCACTCCAG

CACAATAGTAGTAGCAGGAGTCTTCCTCCTTATTCGCTTCTATCCATTAA

TA-GAACATAACAAGACTACCCAAACACTTACTCTTTGTTTAGGAGCTAT

TACCACCCTATTCACCGCTATCTGTGCTCTAACCCAAAATGATATTAAAA

AAATTATCGCATTCTCCACTTCAAGTCAACTAGGATTAATAATAGTAACT

ATTGGAATTAATCAACCCCATTTAGCATTTCTTCACATTTGCACACACGC

ATTCTTCAAAGCTATATTATTCATATGCTCAGGATCAATTATTCACAACC

TAGACAATGAACAAGACATTCGAAAAATAGGAGGCTTGCTCAAAGCCCTT

CCATTCACTTCATCTTCCCTTATTATTGGCAGCCTAGCATTAACAGGAAC

TCCCTTTCTAACAGGATTCTACTCTAAAGATTCGATTATTGAATCTGCTA

ACACGTCGAATACCAACGCCTGAGCCCTAATCATTACTCTTCTTGCCACT

TCCCTAACCGCTGTTTACAGTACACGAATTATCTTCTATGTTCTAATAGG

ACAACCTCGATTTTCCACACTAACTTCAATTAATGAAAATAACCCTCAAC

TGCTTAATTCAATTAAACGCCTTCTTATTGGCAGTATCATTGCAGGATTC

ATCCTCTCATATAATATCCCACCCATAAATATTCCAGTACTAACTATACC

TATTCACCTAAAACTTACGGCATTACTAGTAACCATCTTAGGGTTTGTTA

TCGCTATAGAACTAAACTCAATGACTCTTTATCTCCAAACCAAGATATAC

TCAAACATATCA-AAATTTTCAACCTTACTAGGCTATTTTCCTACCATTA

TTCACCGACTTAGCCCCCATCTCAATCTTGTTATAAGCCAAAAACTATCA

TCAATCCTCCTAGACCTAATTTGACTAGAAAAAACTATCCCTAAATTTAC

CGCTAATCTTCAT---TCAATAGCCTCTACCATATCCTCTAACCAAAAAG

GCCTCATCAAACTATATTTCTTATCATTTTTAATTTCAACACTCTTGGCA

ATTATCTCCATATTCTA---------------------------TTTCCA

CGTGTAATCTCAATCACAATAAAAATACTAACAAACAATGATCAACCAGC

TACAACCATTAATCAACTTCCATAACTATATATAGCCGCCACCCCTATTG

AATCTTCACGAATCAATCCCAACTCATCCCCCTCAAATACTACCCAATCC

TCTAAATCCTTAAACTCAACCACAATCTCTA---------CCTCACCATA

TAAAATTACAAGTATAACAATTAAGAATTCCGCTAAAAATCCCAGCAACA

AAACTCCTCAAATAACTACATTTGACCCCCACGTTTCTGGGTACTCCTCC

GTTGCTATGGCCGTAGTGTAACCAAACACCACTAACATTCCTCCTAGATA

AATTAAAAATATCATTAAACCTAAAAAAGACCCCCCAAAATATAACACAA

TCCCACACCCAATTCCCCCACTAATAATCAATCCTAAGCCTCCGTAAATA

GGAGAAGGCTTTGAAGAAACCCCCACAAAGCCTAAAACAAAAAGCATACT

AAATAAATACGTAATATATATCATTAT---TTTTA-CATGGACTCTA-AC

CATGACTAATGACATGAAAAATCATCGTTGT-TATTCAACTATAAAAACA

-CT----AATGACAAACACCCGCAAAACCCACCCTCTAATTAAAATAATT

AATCACTCCTTCATCGACTTACCTACACCCTCTAATATTTCAACATGATG

AAACTTTGGGTCTCTACTAGGACTTTGCCTAGCTATTCAAATCTTTACCG

GATTATTTCTAGCAATACACTATACATCTGATACTATAACAGCCTTCTCA

TCAGTTACCCACATCTGTCGAGACGTAAATTATGGCTGACTTATCCGTTA

TATTCATGCTAACGGTGCATCAATATTTTTTATCTGCCTCTTCCTTCACG

TAGGCCGAGGAGTGTACTACGGCTCATATACCTACTTTGAGACATGAAAT

ATTGGAGTTATTCTCCTACTTACAGTTATAACCACAGCTTTTATAGGCTA

TGTTCTCCCTTGAGGCCAAATGTCATTCTGAGGAGCAACCGTAATTACTA

ATCTCTTATCTGCTATCCCATATATTGGCACAACCTTAGTTGAATGAATC

TGAGGTGGATTCTCAGTAGACAAAGCCACTCTAACACGATTCTTTGCGTT

CCACTTCATTCTTCCCTTTATCATCGCAGCCCTAGCCATAGTCCATCTCC

TCTTTCTTCATGAAACTGGGTCGAATAATCCCTCAGGTCTTATCTCTGAC

TCAGACAAAATCCCCTTTCACCCATACTATACCATCAAGGATATCCTTGG

AGTTATCCTTCTTATTTCAATTCTAATAATCCTAGTCTTACTTTCACCTG

ATCTTCTAGGAGATCCTGATAATTATATACCCGCAAATCCCTTAAGTACA

CCACCTCATATTAAACCAGAATGATATTTCTTATTTGCTTATGCTATCCT

ACGATCCATTCCTAACAAGCTAGGAGGAGTTTTAGCCCTAGTCTTCTCTA

TCCTTATTCTAATACTCTTCCCACTACTCCATTTATCTAAGCAACGTAGC

ATAATATTCCGACCATTAAGTCAATGTGTATTCTGAATTCTAGTAGCAGA

CCTAATTACACTAACCTGAATCGGAGGACAACCTGTTGAATATCCATATA

CTATTATCGGCCAATTAGCATCAATTCTATACTTCACTATTATTCTCTTA

ATCTTACCAATCATTAGCCTAATCGAAAACAAACTCCTTAAATGAAGA--

--GCCCTAATAGTATAT-AA--ATTACCTTGGTCTTGTAAACCAAAAATG

AAGTT-ACAAAC--TTCTTAGAGCAATATATCAGGGAAGAAAATATA-TT

TCCACCTTCAACTCCCAAAGCTGACATTTCTTACTTAAACTATTCCCTG-

--------------------------------------------------

--------------------------------------------------

--------------------------------------------------

--------------------------------------------------

--------------------------------------------------

--------------------------------------------------

--------------------------------------------------

--------------------------------------------------

--------------------------------------------------

--------------------------------------------------

--------------------------------------------------

--------------------------------------------------

--------------------------------------------------

--------------------------------------------------

--------------------------------------------------

--------------------------------------------------

--------------------------------------------------

--------------------------------------------------

--------------------------------------------------

--------------------------------------------------

--------------------------------------------------

--------------------------------------------------

--------------------------------------------------

------

>Marmota_monax Marmota monax isolate WC2-LM ecotype Northwest Wildlifemitochondrion, complete sequence, whole genome shotgun sequence.

GTTAATGTAGCTTAA--TT----TA-TAAAGCAAAGCACTGAAAATGCTT

AGATGGGTATTTA-C-ACCCCATAAACACATA--GGTTTGGTCCTGGCCT

TTTCATTAGCTTATAGCTAACTTATACATGCAAGCATCCCCGTCCCAGTG

AGAATGCCCT-CTATATCTGTCAAC---TGATCAAAAGGTGCAGGCATCA

AGTTCACT-AATCCTAGTAGCTCACAACGCCTTGC-TTCACCACACCCCC

ACGGGAGACAGCAGTAATTAAAATTAAGCCTATAAACGAAAGTTTGACTA

AGTTAAGCCA--AATAAAGAGTTGGTAAATTTCGTGCCAGCCACCGCGGT

CATACGATTAACTCTAGTTAATGAAGC-AC-GGCGTAAAGCGTGATTAAG

AGA------CTAAC-CAGATAAGATTAAAATAATATTAAACTGTAAAAAG

TC-TTGATAATTATGAAAATCAAACACGAAAGTAATCTTA---AATTTTC

TGAATTCACGATAGCTAAGATCCAAACTGGGATTAGATACCCCACTATGC

TTAGCCCTAAACATAAACA-TTCAAC-AAACAAGAATGTTCGCCAGAGTA

CTACTAGCAATAGCCTGAAACTCAAAGGACTTGGCGGTGCTTTACATCCC

TCTAGAGGAGCCTGTTCTATAATCGATAAACCCCGATACACCTCACCACC

TTTAGCATATATCAGCCTATATACCGCCATCTGCAGCAAACCCTAA-AAA

GGCCCTATAGTAAGCAAGAAAATTCT--ACATTAGTACGTTA-GGTCAAG

GTGTAGCCTATAAGGTGGG-AAGAAATGGGCTACATTTTCTACCTTTCTA

GAATA------AAC-CCCACGATAGCTTTTATGAAACTCAG---AGCATA

AGGCGGATTTAGTAGTAAGTT-AAGAATAGAGAGCTTAACTGAATGG-GG

CAATAAAGCACGCACACACCGCCCGTCACCCTCTTCAAATATACTT---A

ATATAATTCTATTAATAATTTATTTTACTAAA--T--ATATA--AGAAGA

GATAAGTCGTAACAAGGTAAACATACTGGAAAGTGTGTTTGGAATAACCA

AAATGTAGCTTA--T-AATTAAAGCACCCGGCTTACACCCGAGAGATTTC

ACTT-ACTATGAACATTTTGA-ACT-AATGCT-AGCCCAACTCTT-----

ATTCCTTTCAAATACAATCCACTCAATAAGTAAAACATTTATC-TGAAT-

--TAAA-GTATAGGAGATAGAAATTTA--TATAT--GGCGCTATAGAGAA

AGTACCGCAAGGGAAAGA-TGAGAGAACAAATTAATAGTATTAAAAAGCA

AAGATTAACCCTTTTACCTTTTGCATAA-TGATTTAACCAGAAAACACTT

GACAAAAAG-AATTTAAGCCAAACACCCCGAAACCAGACGAGCTACTTAT

GAGCAGCC--AATAAGAGCCTATCCGTCTATGTTGCAAAATAGTGGAGCG

ACTTATAAGTAGAGGTGAAAAGCCTACCGAGCCTGGTGATAGCTGGTTGT

CCA-GACTAGAATTTAAGTTCTGCTTTAAATTTACCTAAAGCATA-AAT-

AA-GCCAAATGTAAGTTTAAATGTTATTCTAAAGAGGGACAGCTCTTTAG

AGCCAAGGAATTAACCTTAACTAGAGAGTAA-----AACTACTAACT--T

TCATAGTTGGCCTAAAAGCAGCCATCAATTAAAAAAGCGTTAAAGCTTAA

TCTAACTAAT---TAAAACTTAATACCCAACTCT---TCC-ATAATCT--

CCTAAATCAATA-CTGGACTAATCTATTTTACAAT-AGAAGAAACTATGT

TAAAATTAGTAACAAGAA-AATAT-T-CTCCCTGCATAAGCTTATATCAG

ATCGAA-TAATTCACTGATAGTTAACAACTGCATA-----ATATTAAATA

AATACAAAATCAATTATTATTAGTATTGTTAATCCAACACTGGTATGCAT

-AT-AAAGGAAAGATTAAAAAAAGTAAAAGGAACTCGGCAAACACTAACC

TCGCCTGTTTACCAAAAACATCACCTCTAGCATAAATAGTATTAGAGGCA

CTGCCTGCCCAGTGACA-TACG----TTCAACGGCCGCGGTATCCTGACC

GTGCAAAGGTAGCATAATCACTTGTTCTTTAAATAAGGACTAGTATGAAT

GGCTTGACGAGGGTTTAACTGTCTCTTACTTTTGATCAGTGAAATTGACC

TTCCCGTGAAGAGGCGGGAATTTCCTAATAAGACGAGAAGACCCTATGGA

GCTTTAATTTAAT-AGTCTCACAACCCTAATAAAATCTT----------A

GGAGTCAA-AATTATTGTTTA-CAGACTAGAAATTTTGGTTGGGGTGACC

TCGGAGTACAAATCAACCTCCGAATGATA-ATAATCT-AGACAC-ACATG

TCCAAATTATAATTC----ATTAATTGACCCAA-A---TCATTGATCAAC

GGAACAAGTTACCCTAGGGATAACAGCGCAATCCTACTCAAGAGTTCATA

TCGACAGTTAGGGTTTACGACCTCGATGTTGGATCAGGACATCCAAATGG

TGTAACCGCTATTAATG-GTTCGTTTGTTCAACGATTAA-AGTCCTACGT

GATCTGAGTTCAGACCGGAGAAATCCAGGTCGGTTTCTATCTATTATTA-

AATTTCTCCCAGTACGAAAGGACAAGAGAAATAAGGCCAATTGAACCC-T

AATGCCTTA-AATTAATGGATGAA-ATAATCTTAATCCAATAATATATTT

TAAAT--AACCTGCCCTAGAGCAGGG--CTT-GTTAAGATGGCAGAGCCT

GGTAA-TTGCGTAAGACTTAAAACTTTATT-TCAGAGGTTCAACTCCTCT

TCTTAACA-TTA--------------------------------------

------------------------------------------ATGTTTAT

AATTAACCTCCTACTTTTAATTATTCCAATCTTAGTAGCTATAGCCTTCC

TTACCTTAATTGAACGAAAAATATTAGGATACATACAACTTCGCAAAGGT

CCTAATATTGTTGGGCCTTACGGCTTACTCCAACCAATTGCTGACGCAGT

AAAATTATTTATTAAGGAACCCATAAAACCTTTGACATCATCAATTGTAT

TATTTATTATTGCCCCAACCCTTGCCCTAACACTAGCATTCACCATATGA

ATTCCTCTACCTATGCCCCAACCTCTCATTAACATAAATTTAGGAGTCCT

ATTCATTTTAGCCACCTCAAGTCTAGCTGTCTATGCAATCTTATGATCTG

GCTGAGCCTCTAACTCCAAATATGCTTTAATTGGAGCCCTACGAGCCGTA

GCACAAACTATCTCATACGAAGTAACACTAGCAATTATCCTTCTCTCAGT

CCTTCTAATAAATGGATCCTTTACCCTATCTACACTTATCATTACCCAAC

AATTCACATGACTCCTATTTCCAACATGACCTCTTGCAATAATGTGATTT

ATTTCAACATTAGCAGAAACCAACCGAGCTCCATTTGATTTAACAGAAGG

AGAGTCAGAACTTGTATCAGGATTTAATGTTGAGTACGCAGCTGGTCCCT

TCGCCTTATTTTTCATAGCTGAATATACAAATATTATCATAATAAACGCA

TTAACAGCAACCCTCTTTATAGGAGCACTACTAAACCCAACTTCCCCTGA

AATTTTTACATTAAGCTTTACTCTAAAAACACTCATACTAACCTCCATCT

TTCTATGAATCCGAGCATCCTACCCTCGATTCCGTTACGACCAACTTATA

CATCTTCTATGAAAAAATTTTCTACCTCTAACATTAGCCTTATGCATATG

ACACATCTCTCTTCCAATTACAGCTGCATGTGTACCACCTCAAATCTAA-

GAAATATGTCTGATAAAAGAGTTACTTTGATAGAGTAAATTATAGAGGTT

TA-AACCCTCTTATTTCTAGAACTATAGGGATCGAACCTAATCCTAAGAA

TTCAAAATTCTTCGTGCTACC-CTT-TACACCATGTCCTAAAC--A----

GTAAGGTCAGCTAAAT-AAGCTATCGGGCCCATACCCCGAAAATGTTGGT

TTATATCCTTCCCGTACTAATTAATCCCTTAACTTCCACCGCAATCTACT

TCACCCTCTTTTCCGGAACTATAATTACACTTTTCAGCTCACATTGACTT

CTAGCTTGGGTGGGCTTAGAAATAAGCATGCTAGCTATTACCCCTATTCT

AATCCACAAAGGAAACCCTCGATCCACAGAAGCTGCATGTAAATATTTTC

TTATTCAAGCAACCGCATCAATAATCTTAATAATAGGTACAATAATTAAT

TTTATAGACTCAGGTCAGTGAATCCTATCCAACTCATACAATCAAATTTC

ATCATTTATACTTACTATTGCCCTTTCAATAAAAATAGGACTCGCCCCAT

TTCACCTATGAGTTCCAGAAATTACCCAAGGGGTCCCGCTTAAATCAGGC

CTAATCATATTAACATGACAAAAAATTGCCCCTATCTCTATCGCATACCA

AATTGCATCTTCCATAAACCCCACACTCATACTATTTATAGGAACATTAT

CAATTATACTAGGAGGCTGGGGAGGACTTAACCAAACTCAACTACGAAAA

ATCCTAGCATATTCATCAATTGCTCACATAGGATGAATAATAGCAATTAT

TACATATAATCCAACTCTGACAATATTTAACCTAATCATCTATATCATAC

TTACTATTAACATATTTATACTCCTTCTTTATTATAAAAAAACTACTACC

CTCTCCCTATCAAACCTATGAAATAAATTTCCCCTTCTAACACCTGCAAT

TTTAATTTTATTAATATCATTAGGAGGTTTACCTCCCCTAACAGGATTTA

TACCAAAATGAATTATTCTTAAAGAACTTATTTCAAATAACAACATTATT

TTCTCTACACTTATGGCAGTTCTAGCACTCCTAAACCTATATTTCTATAC

CCGACTAATCTATTCAACATCCCTAACCCTATTCCCATCATTTAACAACA

CCAAAATAAAATGACAATTCGAAAATGTAAAACTTATACCTCTGCTACCG

ATCTTTATTATTATTTCCATCCTATTTCTCCCATTAACGCCCATA-CTCT

CACTCCT--GAACT----AGGAATTTAGGTTAAT--T-CAGACCAAGGAC

CTTCAAAGTCCTAAGCAAGTA-CCCAAC---ACTTAATTCCTGC------

---A----CTAAGGACTGCAAGACTTTATCTCACAT----CAACTGAATG

CAAATCAATCACTTTAA--TTTAAGCTAAGCCCTTC-ATC------TCTA

GATTGATG--GGATTTAAA-CCCAT-AAAATCTTAGTTAACAGCTAAACG

CCTTACTCAACTGGCTTCAATCTA-CT-TCTCCCGCCGT--AAGTAAAAA

-----GGCGGGAGAAGCCCCGGCAGAG-T-TGA-AGCTGCTCCTTT--GA

-----------------------------ATTTGCAATTCAATA--TGAC

TA-TTCACCTCAGGACT-T-----GGTAAAAAGAGGA----TT-CAACCT

CTGTCTTTAGATTTACAGTCTAATGCTTA--CTCAGCCATCTTACC----

ACCTACCTATGTTCATCAACCGTTGATTCTTCTCAACTAATCATAAAGAT

ATTGGTACACTTTACCTTCTATTTGGCGCTTGAGCCGGTATAGTAGGAAC

TGCGCTCAGCCTACTAATCCGAGCTGAATTAGGCCAACCTGGGACTCTAC

TAGGCGACGATCAAATCTACAATGTTATTGTTACCGCCCATGCCTTTGTT

ATAATTTTCTTTATAGTTATACCAATCATGATTGGTGGATTTGGAAACTG

ACTAGTACCCCTAATAATTGGAGCTCCTGATATAGCATTTCCACGTATAA

ATAACATAAGCTTCTGACTTCTTCCCCCTTCCTTCCTTCTCTTGCTCGCC

TCTTCTATAGTTGAAGCAGGTGCAGGAACAGGATGAACCGTATATCCTCC

ACTGGCCGGAAATCTAGCCCATGCAGGGGCTTCAGTAGATCTAACTATCT

TCTCCCTTCACTTAGCAGGGGTGTCATCAATTCTAGGTGCAATTAATTTT

ATTACAACAATTATCAATATAAAACCGCCTGCCATATCTCAATACCAAAC

CCCTCTGTTTGTATGATCCGTATTGGTTACAGCAGTACTATTACTCTTAT

CTCTTCCAGTTCTTGCAGCAGGGATTACTATGCTCCTTACAGACCGTAAT

CTTAATACTACATTTTTTGATCCTGCTGGAGGCGGAGATCCTATTCTATA

TCAACACTTATTCTGATTTTTTGGACACCCTGAAGTTTATATCCTTATTC

TTCCAGGATTTGGCATAATTTCTCATATCGTAACATACTACTCAGGTAAA

AAGGAACCATTCGGTTATATAGGAATAGTCTGAGCCATGATATCTATTGG

TTTTCTTGGATTTATTGTATGAGCCCATCATATATTTACTGTTGGAATAG

ATGTAGACACTCGAGCTTATTTTACATCCGCAACCATAATTATTGCCATT

CCTACAGGAGTAAAAGTTTTTAGCTGATTAGCAACCCTGCACGGAGGTAA

TATTAAGTGATCACCAGCAATACTATGAGCACTTGGCTTTATTTTTTTAT

TTACCGTAGGAGGCTTAACAGGAATTGTCTTGGCTAACTCTTCATTAGAT

ATTGTTCTACACGATACATACTATGTTGTAGCTCATTTCCACTATGTATT

ATCAATGGGGGCCGTATTTGCTATTATAGGAGGATTTGTTCACTGATTTC

CCCTTTTTTCTGGTTATTCACTAAATGATATATGAGCTAAAATTCATTTT

ACTGTAATGTTTGTTGGAGTAAATTTAACCTTCTTCCCTCAACATTTCCT

GGGACTATCAGGTATACCACGACGATACTCTGACTACCCAGATGCATATA

CAGCATGAAATACTGTATCCTCAATAGGCTCATTCATCTCTCTTACAGCT

GTTATAATTATAATTTTTATAATCTGAGAAGCATTTGCATCAAAACGAGA

AGTACTTACCGTAGAGTTAACACCAACTAACTTAGAGTGACTACATGGAT

GTCCTCCACCCTACCACACATTTGAAGAGCCCGCTTATATTAAAGTTTAG

AC--C--------------AAGAAAGGAAAGAATCGAACTTCCTAAAACT

AGTTTCAAGCTAGCCCCATAGCCACTATGA-CTTTCT---TCAT----GA

GATGTTAGTAAAAT-AATTACATAACTTTGTCAAAGTTAATTTATAGGTT

A------AACTCCTATATGTCTC-TATGGCATACCCACTCGAATTAGGAT

TTCAAGACGCCACATCCCCAATTATGGAAGAGCTTTTACATTTTCACGAC

CATACCCTTATAATTGTTTTCTTAATTAGCTCTCTAGTTCTTTACATCAT

TTCATTAATACTAACTACAAAATTAACTCACACAAGCACTATAGATGCCC

AAGAAGTAGAAACTATTTGAACCATTCTTCCTGCTATCATCCTTATCCTA

ATCGCTCTTCCCTCCCTACGTATTCTATATATAATAGACGAAATTAATGA

CCCATCCTTAACAGTAAAAACAATAGGCCACCAATGATATTGAAGCTACG

AATATACAGACTATGAAGATCTTAATTTTGACTCCTACATAATTCCGACC

TCAGACTTAACTCCAGGAGGCCTACGACTTCTAGAAGTTGATAATCGAGT

TGTTCTTCCAATAGAACTACCTGTGCGAATATTAATTTCATCTGAAGACG

TACTTCACTCTTGAGCAGTCCCATCCCTTGGGTTAAAAACAGATGCAATT

CCAGGCCGACTCAATCAAGCGACACTAACATCAACACGACCAGGACTATA

TTACGGACAATGCTCTGAAATTTGTGGGTCAAATCATAGCTTTATACCAA

TCGTTCTTGAACTAGTCCCGTTAAAACACTTTGAAAACTGATCCTCATCA

ATATTATAA--------------------------------ATT-CATTA

TGAAGCTA--TAGCAGCATCAACCTTTTAAGTTGAAGATTAGGAAC-C-A

A-AT-CTCCTCATAATGAAATGCCCCAACTAGATACATCTGCATGATTTA

TCACAATTTTATCTA-TAATTTTAGCCCTTTTCTTTATACTTCAACTTAA

AATCTCAAATCA--CTATTATCCGTCTAGTATTTCCTCCAAAGACATCAA

-ACTAGTCGAGCACAAAACTCCTTGAGAAGAAAAATGAACGAAAATCTAT

TTGCCTCTTTCATTACCCCTACATTAATAGGCTTCCCTATTGTCCTTTTT

ATTATTATATTTCCCAATTTACTCTTCCCCTCCCCTACTCGACTAGTAAA

TAACCGTTTAGTATCATTTCAACAATGACTAATCCAACTTGTATT-AAAA

CAAATAATGATTATGCATAACCCAAAAGGACGAACCTGAGCCCTAATACT

AATCTCATTAATTATATTTATTGGCTCAACTAATCTTCTAGGTTTACTAC

CCCATTCCTTTACACCCACTACCCAGCTATCAATAAATCTAGGAATAGCT

GTTCCTCTATGAGCAGGAGCAGTAATTACTGGGTTTCGTCACAAAA-TCA

AAGCATCATTAGCCCATTTTCTCCCACAAGGAACCCCAATTATACTTATT

CCCATACTTATTATTATCGAAACAATCAGCCTTTTTATTCAACCTATAGC

ACTAGCCGTTCGACTAACAGCTAATATCACGGCTGGCCATCTTCTTATAC

ATCTAATTGGAGGAGCAACCCTTGTACTGACATCTATTAGCCCCCCTACA

GCTATTTTAACCTTTATTATTCTTGTATTACTAACAATGCTTGAATTTGC

AGTTGCATTAATTCAAGCCTACGTCTTCACTCTCCTAGTAAGCCTATATT

TACATGATAATACTTAATGACCCACCAAACCCACGCTTATCATATAGTTA

ACCCCAGCCCCTGACCCCTAACAGGAGCTCTCTCCGCCTTACTTCTAACA

TCTGGCTTGGTAATATGATTCCACTTTAATTCCTCTCTTATTCTCATATT

AGGCCTAACAACTAATACCCTTACAATATACCAGTGGTGACGAGATATTG

TACGAGAAGGCACATTTCAAGGCCACCACACATCAATTGTCCAAAAAGGC

TTACGATATGGTATAGTACTATTTATTATCTCAGAAGTATTCTTCTTTGC

CGGGTTCTTCTGAGCATTTTACCATTCCAGTCTGGCCCCAACTCCCGAAT

TAGGCAGCTGCTGACCTCCAGTAGGAATTAATCCACTTAATCCTCTAGAA

GTACCATTATTAAATACCTCTGTCCTTTTAGCTTCAGGAGTCTCAATTAC

TTGAGCTCACCATAGCCTAATAGAAGGGGACCGAAAACACATAATTCAAG

CATTATCAATTACAATTGCTTTAGGACTTTACTTTACTCTTCTTCAAGCC

TCTGAATACCTAGAAACATCCTTTACAATTTCAGATAGCGTATATGGCTC

AACATTTTTTATAGCCACAGGCTTCCATGGTCTTCATGTCATAATTGGAT

CAACCTTCCTTCTAGTATGTCTTATTCGTCAACTAAACTTTCACTTTACA

TCAAATCACCATTTTGGATTTGAAGCAGCCGCATGATACTGACATTTTGT

AGATGTCGTATGACTCTTCCTCTATGTATCTATTTATTGATGAGGCTCAT

ATTCT-CTTAGTATTAA-TCAGTACAATTGACTTCCAATCACTTAGTTCT

GGAGCTA-CCCAGAAGAGAATAATAAACCTTATAGTAACTCTCTTCGTGA

ACTCTTTCATTGCCCTACTATTAATCTCCGTAGCATTTTGGTTACCTCAA

CTAAACGTATATGCTGAAAAAGCAAGCCCCTACGAATGTGGCTTCGATCC

CATAGGATCTGCTCGCCTACCATTTTCAATAAAATTTTTCCTTGTTGCAA

TTACATTTCTTCTATTTGATCTAGAAATTGCTCTTCTTCTCCCTCTTCCC

TGAGCTTCCCAAACAAACAATCTTAACCTTATGCTAACTATAGCTTTGCT

CTTAATCCTAATTCTCATCCTCGGACTAGCCTATGAATGAGTCCAAAAAG

GCTTAGAATGAATTGAATA------TGGTAATTAGTTTAAATTAAAA-CA

AGTGATTTCGACTCACTAAATTATGGGCTG-C--CATAATTATCAA--AA

TGCCTATTGTTACTCTCAACATTATATTAGCCTACTCTACATCCCTTCTA

GGGGTATTCATTTATCGATCCCATTTAATATCATCACTTTTATGTTTAGA

GGGTATAATGCTATCAATATTTATTCTATGCTCTCTCCTAATTATAAACT

TACACTTTTCACTATCATTTATGATTCCCATTATCTTATTAGTGTTTGCC

GCATGTGAAGCAGCTGTAGGCTTGGCCCTTCTGGTAATAGTATCTAATAC

ATACGGCCTAGACTACGTCCAAAACTTAAATATCCTTCAATGCTAAAAAT

TATTATTCCTACAATCCTACTCGCTCCCCTTATATGATTCTCAAAACCCT

CTATAATTTGAATTAATCCTTCTATTCATAGTTTAATTATTAGCCTAATT

GTTCTCCTTACATTAAATCGCCCCACAAATACAGATTTAATTTTTTCATT

AGTCTTCTTCACAGACCCTTTATCTTCTCCTTTATTAGTCCTGACAGCAT

GACTTCTACCCCTCATAATTATGGCTAGCCAAAGTCACTTAACTCAAGAA

CCTCTAATCCGAAAGAAACTCTATATTCTCATATTAATCTCACTACAATC

TTTTCTAATTATAACTTTCTCCGTTACTGAACTAATCATATTCTATATCC

TTTTTGAGGCCACCCTAATCCCTACACTTATTATTATCACTCGATGGGGA

AACCAAACTGAACGATTAAATGCAGGATTATATTTCCTATTTTACACCCT

AGTGGGCTCTCTACCCTTATTAGTAGCATTAATTTACATTCAAAAATCTA

CTGGATCTTTAAACTTTATTATTTCAACATACCAATCATCTGCCCTTTCC

ATATCT----------TGAACAAATCATATTCTATGACTAGCATGCATTA

TAGCCTTTATAGTTAAAATACCTCTATACGGTCTCCATCTTTGACTACCA

AAAGCCCATGTCGAAGCTCCCATCGCTGGTTCTATAGTTTTAGCCGCTAT

TCTACTAAAGCTTGGTGGATATGGAATGATCCGAATTTCAGCTCTTCTTC

ATCCTATCACAAGTAACATAGCCTATCCTTTTATTATATTATCTTTATGA

GGTATAATTATAACAAGTTCAATCTGCTTACGACAAACAGACCTAAAATC

CCTCATCGCCTACTCATCAGTAAGTCATATAGCACTAGTAATTGTAGCAA

TTATAATTCAAACCCCCTGAAGCTTTATAGGAGCCACAGCATTAATAATC

GCTCACGGATTGACATCCTCTATACTTTTTTGTCTAGCAAACACTAACTA

TGAACGAATTCATAGTCGAACTATAACATTAGCTCGAGGCTTACAATCTA

TTCTCCCCCTTATAGCAACATGATGAGTTCTAGCAACCCTAACCAATCTA

GCCCTCCCACCCTCCATTAACTTAATTGGTGAACTATTTATTATTATAGC

ATCATTTACTTGATCAAACATTTCAATTATCTTAACTGGGCTAAATATAT

TAATCACAGCTCTCTATTCGCTATATATACTAATTACAACACAACAAGGA

AAATTTACATATCACACACTAAATATTAGCCCCTCCTTCACACGAGAAAA

TACACTTATATTTCTTCACCTTTTTCCACTTATCATCCTATCAACGAACC

CTACCATCATTCTGGGACATTTATACTGTAAATATAGTTTAAGCAAAACT

TTAGATTGTGAATCTAACAATAGAGAATCGTAA--TCTCTTATTTACCAA

GAAAGCAT---GCAAGAACTGCTAACTCATGC-TACCGTGACTAAACCCA

CGGCTT--------------------------------------------

-------------------TCTTA-ACTTTTATAGGATAGAAGCA-ATCC

GTTGGTCTTAGGAACCAAAAA-A-TTGGTGCAACTCCAAATAGAAGTAAT

TAATA---TATTCTCTTCACTTATTCTCACATCACTTACAACCCTATCAC

TTCCCATTTTTCTAACAATAACTAATCACCACAAGCACATTAACTTTCCA

AACTACGTTAAAATCTCCATTATTTGTGCATTATCACTCTGCATCGTACC

AACACTAATATTTATTAACTCAAATTATGAACTCATTATCTCAAACTGAC

ACTGAATAACTATTCAAACATTCACTTTTTCCATAAGCTTTAAATTAGAC

TACTTTTCCATACTATTTATGCCTGTAGCACTATTCGTCACATGATCAAT

TATAGAATTCTCAATATGATATATACACTCCGATCCCTTCATCAACCGTT

TCTTTAAATATCTCCTTTTATTCCTTATCACCATAATAATCCTAGTAACA

GCTAATAACCTATTTCAACTATTTATCGGCTGAGAAGGAGTAGGTATTAT

ATCTTTCTTACTAATTGGCTGGTGATACAGCCGAACAGATGCTAATACAG

CAGCCCTCCAAGCCATTTTATATAACCGAGTTGGAGATATTGGATTCGTT

CTAGCTATAGCATGGTTCCTACTTAACTCAAACTCATGGGAACTACAACA

ACTTTTTATTATAGATGCCTCTCTA------TTTCCTCTTCTAGGACTAC

TCCTGGCCGCCACAGGAAAATCTGCCCAATTTGGTCTTCACCCTTGATTA

CCTTCCGCCATAGAAGGTCCAACCCCTGTTTCAGCTTTACTTCACTCCAG

CACAATAGTAGTGGCAGGAATCTTTCTCCTTATTCGCTTTTACCCATTAA

TA-GAACATAATAAAACTATCCAAACACTTACTCTTTGCTTAGGAGCTAT

TACCACCCTATTCACCGCTATCTGTGCTTTAACCCAAAATGACATTAAAA

AAATTATCGCATTCTCTACTTCAAGTCAATTAGGATTAATAATAGTAACT

ATTGGAATTAATCAACCTCACCTAGCATTTCTTCATATTTGCACACACGC

ATTCTTCAAAGCTATACTATTCATATGCTCAGGATCAATTATTCACAACC

TAAATGACGAACAAGACATTCGAAAAATAGGAGGCTTATTTAAAGCCCTC

CCATTCACTTCATCTTCACTTATTATTGGTAGCTTAGCATTAACAGGAAC

TCCCTTTCTAACAGGATTTTATTCTAAAGACCTAATCATTGAATCTGCTA

ATACGTCGAATACCAACGCCTGAGCCCTAATTATTACTCTCCTTGCCACT

TCCCTAACCGCTGTCTACAGTACACGAATTATCTTCTATGTTCTAATAGG

ACAACCTCGATTTTCTACACTAATTCCAATCAATGAAAATAACCCTCAAC

TGCTTAATTCAATTAAACGCCTTCTTATTGGCAGTATTATCGCAGGATTT

ATCCTTTCATATAATATCCCACCCATAAATACCCCAGTACTAACTATACC

TATTTATCTAAAACTTATAGCATTATTAGTAACCATCCTAGGGTTTGTTA

TCGCGATAGAACTAAACTCAATGACCCTCTATCTCCAAACCAAAATGTAC

TCAAATATATCA-AAATTTTCAACTTTACTAGGCTATTTTCCTACTATTA

TTCACCGACTCAGCCCCCGTCTCAATCTTATTATAAGCCAAAAACTATCA

TCAACCCTTCTAGACCTAGTCTGATTAGAAAAAACTATCCCCAAATTCAC

TGCTAACCTTCAT---TCAGTAGCCTCTACCATATCCTCTAACCAAAAAG

GCCTCATCAAACTATACTTCTTATCATTTCTAATTTCAACACTCTTAGCA

ACTATCTCTATATTCTA---------------------------TTTCCA

CGTGTAATCTCAATCACAATAAAAATACTAACGAACAATGATCAACCAGC

TACAACCATTAATCAACTTCCACAGCTGTACATAGCCGCCACCCCTATTG

AATCCTCACGAACTAATCCCAACTCATCCCCTTCAAATACTATCCAATTC

TCCAAATCCTTAAACTCAACTACAATCTCTA---------CCTCATCATA

TATAATCATAAGTATGATAATTAAAAACTCCACTAAAAATCCCAACAACA

AAACTCCTCAAATAACCACACTTGACCCTCACGTTTCTGGATATTCCTCC

GTTGCTATAGCCGTAGTATAACCAAATACCACCAACATTCCCCCCAAATA

AATCAAAAATACTATTAAACCTAAAAAAGACCCTCCAAAATATAACACAA

CCCCACATCCAATTCCCCCACTAATAATCAGCCCCAAACCTCCATAAATA

GGAGAAGGCTTTGAAGAAAATCCTACAAAGCCTAAAACAAAAAGTATACT

TAACAAGTACGTAATATATGTCATTAT---TTTTA-CATGGAATCTA-AC

CATGACCAATGACATGAAAAATCATCGTTGT-TATTCAACTATAAAAACA

-CT----AATGACAAACACCCGCAAAACCCATCCTCTATTTAAAATTATT

AACCACTCCTTTATTGACCTACCTGCACCCTCTAATATTTCAGCATGATG

AAATTTTGGGTCACTACTAGGGCTCTGCTTAGCTATCCAAATCCTCACCG

GGTTATTTCTAGCAATACACTATACTTCCGATACCATAACAGCCTTTTCG

TCAGTTACTCATACATGCCGAGACGTAAATTACGGCTGACTTATCCGCTA

CATACATGCTAACGGCGCATCAATATTTTTTATCTGCCTCTTTCTTCATG

TAGGCCGAGGAATATACTATGGCTCATACACCTACTTTGAAACATGAAAC

ATTGGAGTTATTCTCCTATTTACAGTAATAGCTACAGCTTTCATAGGCTA

TGTACTCCCCTGAGGCCAAATATCATTCTGAGGAGCAACCGTAATTACCA

ATCTTTTATCTGCTATTCCATACATTGGTACAACTTTAGTTGAATGAATC

TGAGGCGGATTCTCAGTAGATAAAGCCACTCTAACACGGTTCTTTGCATT

CCACTTCATCCTTCCCTTTATCATCGCAGCTCTGGTCATAGTCCATCTCC

TTTTTCTTCATGAAACCGGATCAAACAACCCCTCAGGCCTTATTTCTGAC

TCAGACAAAATCCCCTTTCACCCATACTATACCATCAAGGACATCCTTGG

AGTTCTTCTTCTTATTTTAATTCTAATAATTCTAGTCTTATTTTCACCTG

ACCTTCTAGGAGATCCTGATAATTATACACCTGCAAACCCCTTAAGTACA

CCACCCCATATTAAACCAGAGTGATACTTCTTATTTGCCTACGCTATCCT

ACGATCTATCCCCAACAAACTAGGAGGAGTTTTAGCCCTAGTCTTCTCAA

TTCTTATTCTAATACTCTTCCCGCTACTCCACTTATCTAAGCAACGTAGC

ATAATATTCCGACCATTAAGTCAATGCATATTTTGGGTCCTAGTAGCAGA

CCTAATTACACTAACCTGAATCGGAGGACAACCCGTTGAATACCCATATA

CTATCATTGGCCAACTAGCGTCAATTCTATACTTTGCTATTATTCTCTTA

ATCCTACCAACCATTAGTCTAATCGAAAACAAACTTCTTAAATGAAGA--

--GCCCTAATAGTATAT-GC--ATTACCTTGGTCTTGTAAACCAAAAATG

AAGTT-ATAAAC--TTCTTAGAGCAATATATCAGGGAAGAAAATACACTT

TCCACCTTCAACTCCCAAAGCTGACATTTCTTACTTAAACTATTCCCTG-

--------------------------------------------------

--------------------------------------------------

--------------------------------------------------

--------------------------------------------------

--------------------------------------------------

--------------------------------------------------

--------------------------------------------------

--------------------------------------------------

--------------------------------------------------

--------------------------------------------------

--------------------------------------------------

--------------------------------------------------

--------------------------------------------------

--------------------------------------------------

--------------------------------------------------

--------------------------------------------------

--------------------------------------------------

--------------------------------------------------

--------------------------------------------------

--------------------------------------------------

--------------------------------------------------

--------------------------------------------------

--------------------------------------------------

------

>Marmota_marmota Marmota marmota marmota mitochondrion, complete genome.

GTTAATGTAGCTTAA--TT----TA-TAAAGCAAAGCACTGAAAATGCTT

AGATGGGTATTTA-T-ACCCCATAAACACATA--GGTTTGGTCCTGGCCT

TTTCATTAGCTTCCAGCTAACTTATACATGCAAGTATCCCCGTCCCAGTG

AGAATGCCCT-CTATATCTATTAAC---TGATCAAAAGGTGCAGGCATCA

AGTTCACC-AATCCTGGTAGCTCACAACGCCTTGC-TCCACCACACCCCC

ACGGGAGACAGCAGTAATTAAAATTAAGCCTATAAACGAAAGTTTGACTA

AGTTAAGCCA--AACAAAGAGTTGGTAAATTTCGTGCCAGCCACCGCGGT

CATACGATTAACTCTAGTTAATGAAAT-AC-GGCGTAAAGCGTGATTAAG

AAA------CCAAT-CAAATAAGATCAAAATAGTATTAAACTGTAAAAAG

TC-TTGATAGTTATGAAGATCCAATACGAAAGAAATCTTA---AATTCTC

TGAATTCACGATAGCTAAGATCCAAACTGGGATTAGATACCCCACTATGC

TTAGCCCTAAACATAAACA-TTCAAC-AAACAAGAATGTTCGCCAGAGTA

CTACTAGCAACAGCCTGAAACTCAAAGGACTTGGCGGTGCTTTACATCCC

TCTAGAGGAGCCTGTTCTATAATCGATAAACCCCGATATACCTCACCACC

TTTAGCAAATATCAGCCTATATACCGCCATCTGCAGCAAACCCTAA-AAA

GGCCTTACAGTAAGCAAGAAAACTCT--ACATTAACACGTTA-GGTCAAG

GTGTAGCCTATAAGGTGGG-AAGAAATGGGCTACATTTTCTACCTTCCTA

GAATA------GAC-CCCACGATAGCTTTTATGAAACTCAG---AGCATA

AGGCGGATTTAGTAGTAAGTT-AAGAATAGAGAGCTTAACTGAATAG-GG

CAATAAAGCACGCACACACCGCCCGTCACCCTCTTCAAATATACTT--TA

ATACAACTCTATTAATAATTTACTCTATTAAA--T--ATATA--AGAAGA

GACAAGTCGTAACAAGGTAAACATACTGGAAAGTGTGTTTGGAATAACCA

AAATGTAGCTTA--T-AATTAAAGCACCCGGTTTACACCCGAGAGATTTC

ACTT-ACTATGAACATTTTGA-ACT-AATGCT-AGCCCAACTCTT-----

ATTCCTTTCAAATACAATAAA-TTAATAAATAAAACATTTATC-CAAA--

--TAAA-GTATAGGAGATAGAAATTTA--TATCT--GGAGCTATAGAAAA

AGTACCGCAAGGGAAAGA-TGAAAGAATAAATTAATAGTACTAAAAAGCA

AAGATTAACTCTTTTACCTTTTGCATAA-TGATTTAACCAGAAAACACTT

GACAAAAAG-AATTTAAGCCAAGCACCCCGAAACCAGACGAGCTACTTAT

GAGCAGCC--AATAAGAGCCTATCCATCTATGTCGCAAAATAGTGGAACG

ACTTATAAGTAGAGGTGAAAAGCCTATCGAGCCTGGTGATAGCTGGTTGT

CCA-GACTAGAATTTAAGTTCTACTTTAAATTTACCTAAAGCACA-AAT-

AA-GCCAAATGTAATTTTAAATGTTATTCTAAAGAGGGACAGCTCTTTAG

AGTCAAGGAATTAACCTTAACTAGAGAGTAA-----AACTACCAACT--T

CCATAGTTGGCCTAAAAGCAGCCATCAATTAAAAAAGCGTTAAAGCTTAA

TC-AACTAAC---TAAAACTTAATACCCAACTCT---TTC-ATAACCT--

CCTAAATCAATA-CTGGACTAATCTATTCTACAAT-AGAAGAAACTATGT

TAAAATCAGTAACAAGAA-AATAT-T-CTCCCCGCATAAGCTTATATCAG

ATCGAA-TAATTCACTGATAGTTAACAACTCCATA-----ATATTAAATA

GACACAAAATCA-TTATTATTAATATTGTTAATCCAACACTGGTATGCGT

-ATTAAGGGAAAGATCAAAAAAAGCAAAAGGAACTCGGCAAACACTAACC

TCGCCTGTTTACCAAAAACATCACCTCTAGCATAAATAGTATTAGAGGCA

CTGCCTGCCCAGTGACA-TACG----TTTAACGGCCGCGGTATCCTGACC

GTGCAAAGGTAGCATAATCACTTGTTCTTTAAATAAGGACTAGCATGAAT

GGCCTAACGAGGGTTTAACTGTCTCTTGCTTTTGATCAGTGAAATTGACC

TTCCCGTGAAGAGGCGGGAATTCCCTAATAAGACGAGAAGACCCTATGGA

GCTTTAATTTAAC-AGTCTCACAGCCCTAATAAAATCTT----------A

GGACTCAA-AATCATTGTTTA-CAGACTAGAAATTTTGGTTGGGGTGACC

TCGGAGTACAAACCAACCTCCGAATGATA-ATAATCT-AGACGT-ACATG

TCCAAATTATAATTC----ATCAATTGACCCAA-A---T-ATTGATCAAC

GGAACAAGTTACCCTAGGGATAACAGCGCAATCCTACTCAAGAGTTCATA

TCGACAGTCAGGGTTTACGACCTCGATGTTGGATCAGGACATCCAAATGG

TGTAACCGCTATTAATG-GTTCGTTTGTTCAACGATTAA-AGTCCTACGT

GATCTGAGTTCAGACCGGAGAAATCCAGGTCGGTTTCTATCTATTACTA-

AATTTCTCCCAGTACGAAAGGACAAGAGAAATAAGGCCAATTAAACCT-T

AATGCCTTA-AACCAATGGATGAA-ATAATCTTAATTCAGTAATATATTT

TAAAT--AACCTGCCCTAGAGCAGGG--CTT-GTTAAGATGGCAGAGCCT

GGTAA-CTGCGTAAGACTTAAAACTTTATT-TCAGAGGTTCAACTCCTCT

TCTTAACA-TTA--------------------------------------

------------------------------------------ATGTTTAT

AATTAATCTCCTACTTCTAATCATCCCAATCCTAGTAGCTATAGCCTTCC

TTACCTTAATCGAACGAAAAATACTAGGATATATACAACTTCGCAAAGGC

CCTAATGTCATTGGGCCTTACGGCTTACTTCAACCGTTCGCTGACGCAAT

AAAATTATTTATTAAAGAACCTATAAAACCCTTAACATCATCAATTACAT

TATTTATTACTGCTCCAACCCTTGCCCTAACACTAGCATTCACCATATGA

ATTCCTCTACCCATACCCCAACCCCTTATTAACATAAACATAGGAGTCCT

ATTCATCTTAGCTACCTCAAGTCTAGCTGTCTATGCAATCTTATGATCTG

GCTGGGCCTCTAACTCCAAATATGCCTTAATCGGAGCTCTACGAGCCGTA

GCACAAACTATCTCATATGAAGTAACACTAGCAATTATTCTCCTCTCAGT

CCTTCTAATAAATGGATCCTTCACTCTATCTACACTTATTATTACCCAAC

AATTTACGTGACTTCTAATTCCAACATGACCTCTAGCAATAATATGATTT

ATTTCAACATTAGCAGAAACCAACCGAGCCCCATTTGATTTAACAGAAGG

AGAATCAGAACTTGTATCAGGATTTAATGTTGAATATGCAGCTGGTCCCT

TCGCCTTATTTTTCATAGCTGAATACACCAATATCATTATAATAAACGCA

TTAACGGTAACTCTCTTTATAGGAACATTACTAAATCCTTCTTCCCCTGA

AACTTTTACATTAAGCTTTACTCTAAAAACACTCATACTAACCTCCATTT

TTCTATGAATCCGAGCATCCTATCCTCGATTCCGTTACGACCAACTTATG

CACCTCCTATGAAAAAACTTTCTACCCCTAACATTAGCTTTATGCATATG

ACATATCTCTCTTCCAATTACAACTGCATGTGTACCACCCCAAATCTAA-

GAAATATGTCTGATAAAAGAATTACTTTGATAGAGTAAATTATAGAGGTT

TA-AATCCTCTTATTTCTAGAACTATAGGGATTGAACCTAATCCTAAGAA

TTCAAAATTCTTCGTGCTACC-CTT-TACACCATGTCCTAAAC--A----

GTAAGGTCAGCTAAAT-AAGCTATCGGGCCCATACCCCGAAAATGTTGGT

TTATACCCTTCCCGTACTAATTAATCCCTTAACTTCCACCACAATCTACT

TCACCCTCTTCTCCGGAACTATAATCACACTTTTTAGCTCACATTGACTT

CTAGCTTGAGTAGGCTTAGAAATAAGCATGTTAGCTATTACCCCTATTCT

AATCCACAAAGGAAATCCTCGATCCACAGAAGCTACATGCAAATATTTTC

TCATCCAAGCAACCGCATCAATAATCTTAATAATAGGCACAATAATTAAT

TTTATAGACTCAGGCCAGTGAACCCTATCCAACTCATATAATCAAATTTC

ATCATTTATACTTACTATCGCCCTCTCAATAAAAATAGGACTCGCCCCAT

TTCACTTATGAGTTCCAGAAATTATCCAAGGGATCCCACTTAAATCAGGC

CTAATCGTATTAACATGACAAAAAATTGCTCCAATCTCTATTGCATATCA

AATTGCATCCTCCATAAACTCCACACTCATACTATTTATAGGGACCCTAT

CAATTATACTAGGAGGCTGAGGAGGACTTAACCAAACCCAACTACGAAAA

ATTCTAGCATACTCATCAATCGCCCATATAGGATGAATAATAGCAATTAT

TACATATAATCCAACCCTAACAATATTTAACCTAATCATTTATATCATAC

TTACTATCAACATATTTATGCTTCTCCTTTATTATAAAAAAACAACTACC

CTTTCCCTATCAAACCTATGAAATAAATTTCCTTTCCTAACACCTATAGT

TTTAATTGTATTAATATCATTAGGAGGTTTACCTCCCCTAACAGGATTTA

CACCAAAATGAATCATTCTTAAAGAACTTATCTCAAATAACAACATTATT

TTCCCTACACTTATAGCAATACTAGCCCTTCTAAACCTGTATTTCTATAC

CCGACTAATCTATTCAACATCCTTAACCCTATTTCCATCATTTAACAACA

CCAAAATAAAATGACAGTTCGAAAGTGTAAAACTCACACCTCTCTTACCA

ATCTTCATCATTATTTCTATTCTCTCTCTCCCATTAATACCTATA-CTCT

CACTCCT--GAACT----AGGAATTTAGGTTAAT--T-TAGACCAAGGAC

CTTCAAAGTCCTAAGCAAGTA-CCCAAC---ACTTAATTCCTGC------

---A----CTAAGGACTGCAAGACTTTATCTTACAT----CAACTGAATG

CAAACCAATCACTTTAA--TT-AAGCTAAGTCCTTT-ATT------TCTA

GACTGATG--GGATTTAAA-CCCAT-AAAATCTTAGTTAACAGCTAAACG

CCTCACTCAACTGGCTTCAATCTA-CT-TCTCCCGCCGT--AAGTAAAAA

-----GGCGGGAGAAGCCCCGGCAGAG-T-TGA-AGCTGCTCCTTT--GA

-----------------------------ACTTGCAATTCAATA--TGAC

TA-TTCACCTCGGGACT-T-----GATAAAAAGAGGA----CT-CAACCT

CTGTCTTTAGATTTACAGTCTAATGCTTA--CTCAGCCATTTTACC----

ACCTACCTATGTTCATCAACCGTTGATTCTTCTCAACTAATCATAAAGAT

ATTGGTACACTTTACCTTTTATTTGGCGCTTGAGCTGGTATAGTAGGAAC

TGCACTCAGTCTACTAATTCGAGCTGAATTAGGCCAACCTGGGACTCTAT

TAGGCGACGATCAAATTTACAATGTCATTGTTACCGCCCACGCATTTGTT

ATAATTTTCTTTATAGTTATGCCGATCATAATTGGTGGATTTGGAAACTG

ACTGGTACCTCTAATAATCGGAGCCCCTGACATAGCATTTCCACGTATAA

ATAACATAAGCTTCTGACTTCTCCCTCCTTCCTTTCTTCTCTTGCTCGCC

TCTTCTATAGTTGAAGCAGGTGCGGGAACAGGTTGGACCGTATACCCTCC

ACTGGCCGGAAATCTAGCCCATGCAGGAGCTTCAGTAGATCTAACTATCT

TTTCCCTTCACTTGGCAGGGGTATCATCGATTCTAGGTGCAATTAATTTT

ATTACAACAATTATTAACATAAAACCACCTGCCATATCTCAATATCAAAC

CCCTCTATTTGTGTGATCCGTACTAGTCACAGCGGTACTATTACTCCTAT

CTCTTCCTGTTCTTGCGGCAGGAATTACTATACTCCTTACAGACCGTAAT

CTTAATACTACATTTTTTGACCCTGCTGGAGGTGGAGATCCTATTTTATA

TCAACACTTATTCTGATTTTTTGGGCACCCTGAAGTTTATATCCTTATTC

TTCCAGGATTTGGCATAATTTCTCATATCGTAACATACTACTCAGGAAAA

AAGGAACCATTCGGTTATATAGGAATAGTCTGAGCCATAATATCTATCGG

CTTTCTTGGATTTATTGTATGAGCCCATCATATGTTTACTGTTGGAATAG

ATGTAGACACTCGAGCTTACTTTACATCTGCGACCATAATTATTGCTATT

CCTACAGGAGTTAAAGTTTTTAGCTGATTAGCAACCCTGCACGGAGGTAA

TATTAAATGATCTCCAGCAATACTATGAGCACTTGGTTTCATTTTCTTAT

TTACCGTAGGAGGCTTAACAGGAATTGTCTTAGCTAATTCTTCATTAGAT

ATTGTTCTACACGATACATATTATGTTGTAGCTCACTTCCACTATGTATT

ATCAATAGGGGCCGTATTTGCTATTATAGGAGGATTCGTTCACTGATTTC

CCCTTTTTTCTGGCTATTCACTAAATGACATATGAGCTAAAATTCATTTT

ACTGTGATGTTTGTCGGAGTAAATTTAACCTTCTTCCCTCAACATTTCTT

AGGATTATCAGGTATACCACGTCGATACTCTGACTACCCAGATGCATATA

CAGCATGAAATACCGTATCCTCAATAGGTTCATTCATCTCTCTTACAGCC

GTCATAATTATAATTTTCATAATTTGAGAAGCATTTGCATCAAAACGAGA

AGTACTTACTGTAGAGTTAACACCAACTAATTTAGAGTGACTACATGGAT

GTCCTCCACCCTATCACACATTTGAAGAGCCTACCTATATTAAAGTTTAA

GT--C--------------AAGAAAGGAAAGAATCGAACTTCCTAAAACT

AGTTTCAAGCTAGCCCCATAGCCACTATGA-CTTTCT---TCAT----GA

GATATTAGTAAAAT-AATTACATAACTTTGTCAAAGTTAATTTATAGGTT

A------AACTCCTATGTATCTC-TATGGCATACCCACTCGAATTAGGAT

TTCAAGACGCCACATCTCCCATTATAGAAGAGCTTTTACACTTTCATGAC

CACACCCTTATAATTGTTTTCTTAATTAGCTCTCTAGTTCTCTATATTAT

TTCATTAATACTAACTACAAAATTAACTCATACAAGCACTATAGATGCCC

AAGAAGTTGAAACTATTTGAACTATTCTCCCTGCTATTATTCTTATCCTA

ATCGCTCTTCCCTCCCTACGTATTCTATATATAATAGATGAAATTAATGA

CCCATCCTTAACAGTAAAAACAATAGGCCACCAATGATATTGAAGCTATG

AGTATACAGACTATGAAGATCTTAGTTTCGATTCTTACATGATTCCAACC

TCAGACTTAGCCCCAGGAGGCCTACGGCTTCTAGAAGTCGATAATCGAGT

TGTACTTCCAGTAGAACTACCCGTACGAATTTTAATTTCATCTGAAGACG

TTCTTCACTCTTGAGCAGTTCCATCCCTTGGATTAAAAACAGATGCAATT

CCAGGCCGACTTAATCAAGCAACACTAACATCAACACGACCAGGACTATA

TTACGGACAATGCTCTGAAATCTGTGGATCAAATCATAGTTTTATACCAA

TTGTTCTTGAACTAGTTCCATTAAAACACTTCGAAAACTGATCCTCATCA

ATATTATAA--------------------------------ATT-CATTA

TGAAGCTA--TAGTAGCATCAACCTTTTAAGTTGAAGATCAGGAAC-C-A

A-AT-CTCCTCATAATGAAATGCCCCAACTAGACACATCCACATGATTTA

TTACAATTCTATCAA-TAATTCTAGCTCTTTTCTTTATATTTCAACTTAA

AATATCAAATCA--CTATTACCCATCCAGTCCTTCCCCCAAAGACACTAA

-ACTAGCTGAGCATAAAACTCCTTGAGAAGAAAAATGAACGAAAATCTAT

TTGCCTCTTTCATTACCCCTACATTAATAGGTTTTCCTATTGTCCTTTTT

ATTATTATATTTCCTAGTCTACTCTTCCCCTCCTCTACTCGACTAGTAAA

CAGCCGTCTAGTATCATTTCAACAATGACTAGTCCAATTTGTACT-AAAA

CAAATAATAATTATGCATAGCCCAAAAGGACGTACTTGATCCCTAATACT

AATTTCATTAATTATATTTATTGGCTCGACTAACCTTCTAGGTTTACTAC

CCCACTCTTTTACACCAACTACCCAGCTATCAATAAATTTAGGGATAGCC

GTGCCTCTATGAGCAGGAACAGTAATTACTGGATTTCGTCACAAAA-CTA

AAGCATCATTAGCCCACTTTCTCCCACAAGGAACCCCAACTCTACTTATT

CCCATACTCATTATTATTGAAACAATTAGCCTTTTTATTCAACCTATAGC

ATTGGCCGTACGACTAACAGCTAATATTACAGCCGGTCATCTCCTCATAC

ATTTAATTGGAGAAGCAACCCTCGTACTAATATCTATTAGCCCCCCTGCA

GCTATTTTAACCTTTATCATTCTTGTATTACTAACAATGCTTGAATTCGC

AGTTGCATTAATTCAAGCTTACGTCTTCACTCTCCTAGTAAGCTTATATT

TACATGATAATACTTAATGGCCCACCAAACCCACGCTTATCATATAGTCA

ACCCCAGCCCCTGACCCCTAACAGGAGCCCTCTCCGCCTTACTCCTAACA

TCTGGCTTAGTAATATGATTCCACTTTAATTCCTCTTTAATTCTCATATT

AGGCCTGATAGCTAATACCCTAACAATATATCAATGGTGACGAGATATTG

TACGAGAAGGCACATTTCAAGGTCACCATACATCAATTGTCCAAAAAGGC

CTACGATATGGTATAGTGCTATTTATTATCTCAGAAGTATTCTTCTTTGC

CGGGTTCTTCTGAGCATTCTACCATTCTAGTCTGGCCCCGACTCCCGAAC

TAGGCAGCTGCTGACCTCCAGTAGGAATTAATCCACTTAATCCTCTAGAA

GTACCATTATTAAATACCTCCGTTCTTTTAGCTTCAGGAGTCTCAATCAC

TTGAGCTCACCATAGCCTAATAGAAGGAGACCGAAAGCATATAATTCAAG

CATTATCAACCACAATTGCTTTAGGACTTTACTTTACCCTCCTTCAAGCC

TCTGAATATCTAGAAACATCCTTTACAATTTCAGATGGCGTATATGGCTC

AACATTTTTTATAGCTACAGGCTTCCATGGTCTACATGTTATAATTGGAT

CCACTTTTCTCCTAGTATGTCTCATTCGTCAACTAAACTTTCATTTTACA

TCAAATCACCATTTTGGATTTGAAGCAGCCGCGTGATATTGACATTTCGT

AGATGTCGTATGACTTTTCCTCTACGTATCTATTTATTGATGAGGCTCAT

ATTCT-CTTAGTATTAA-TCAGTACAATTGACTTCCAATCATTTAGCTCT

GGAACTAGCCCAGAAGAGAATAATAAACCTTATAGTAACTCTCTTTGTAA

ACTCTTTCATTGCTCTACTATTAATCTCCGTAGCATTTTGACTACCTCAA

CTAAACGTATATGCTGAAAAAGCAAGCCCCTACGAATGTGGTTTTGACCC

TATAGGATCTGCTCGCTTGCCATTTTCAATAAAATTTTTCCTTGTTGCAA

TTACATTTCTTCTATTTGACCTAGAAATTGCTCTTCTTCTCCCCCTTCCC

TGAGCTTCCCAGACAAATAATCTTAACCTTATACTAACTATAGCCTTACT

CTTAATCTTAACTCTTACCCTCGGACTAGCCTACGAATGAATTCAAAAAG

GTCTAGAATGAATCGAATA------TGATAATTAGTTTAAAATAAAA-CA

AGTGATTTCGACTCACTAAATTATGAGCTG-C--CATAATTATCAA--AA

TGCCTATTATTACTCTCAATATTATATTAGCTTACTTTACATCTCTACTA

GGAATATTCATTTACCGATCCCATTTAATATCGTCACTTTTATGCTTAGA

GGGTATAATATTATCAATATTTGTCCTATGCTCTCTCCTAATTATAAATT

TCCACTTTTCCCTATCATTTATAATTCCCATTACCTTACTAGTGTTTGCT

GCATGTGAAGCAGCTGTAGGTTTGGCCCTTCTCGTAATAGTATCCAACAC

ATATGGTCTAGACTACGTACAAAACCTAAACATTCTTCAATGCTAAAAAT

TATTATTCCTACAATTCTACTTGCTCCCCTTATATGATTCTCAAAACCCT

CTATAATCTGAATTAACCCCTCAATTCATAGTCTTATCATTAGCCTAATC

GTTCTTCTTACATTAAATCACCCCACAAACACAGATTTAACTTTCTCATT

AACCTTCTTTACAGACCCTCTATCCTCTCCCTTATTAATTCTAACAGCAT

GACTTCTACCCCTCATAATCATAGCAAGCCAAAGTCATTTGACTCAAGAG

CCATTAATCCGAAAGAAACTTTATATCCTTATATTAATCTCACTACAATC

CTTTCTAATTATAACTTTTTCCGCCACTGAACTAATCATATTCTATATCC

TATTTGAAGCTACCCTAATCCCTACACTTATCATTATCACTCGATGAGGA

AACCAAACTGAACGATTAAATGCAGGGCTATATTTCCTATTTTATACCTT

AGTAGGTTCTCTACCCTTACTAGTAGCATTAATTTATATTCAAAAATCTA

CTGGATCTTTAAACTTTATTATTTCAGCGTATCAGTCATCTGTCCTCCCC

ATATCT----------TGAACAAATCATATTCTATGACTAGCATGTATTA

TAGCTTTTATAGTTAAAATGCCCTTATACGGCCTTCACCTTTGACTACCG

AAAGCCCATGTCGAAGCTCCTATTGCTGGTTCTATAGTTTTAGCCGCCAT

TCTACTAAAACTTGGTGGGTACGGGATGGTCCGAATTTCAACTTTTCTAT

GTCCTATCACAAGTAATATAGCCTACCCTTTTATCATATTATCCTTATGA

GGCATAATTATAACGAGTTCAATCTGCTTACGACAAACAGACCTGAAATC

TCTCATCGCATACTCGTCAGTAAGTCATATAGCATTAGTAATTGTAGCAA

TTATAATTCAAACCCCCTGAAGCTTTATAGGAGCCACAGCATTAATAATT

GCTCATGGACTAACATCTTCCATACTATTTTGTCTAGCAAATACTAACTA

TGAACGAACTCATAGTCGAACTATGACATTAGCTCGAGGTTTACAGTCTA

TTCTTCCCCTCATAGCAACATGATGAATTCTAGCAACTCTAGCTAATTTG

GCCCTCCCACCCTCCATCAACTTAGTTGGCGAATTATTTATCATTATAGC

ATCATTTACTTGATCAAATATTTCAATTATCTTAACTGGACTAAATATAT

TAATTACAGCTCTCTACTCGCTATATATATTAATTACAACACAACAAGGA

AAACTTACATATCACACACTAAATATTAGCCCATCCTTTACGCGAGAAAA

TACACTCATATTCCTTCATCTTTTCCCGCTTATTATCCTATCAACGAACC

CTGCCATTATTCTAGGCCATTTATACTGTAAATATAGTTTAAGCAAAACT

TTAGATTGTGAATCTAACAATAGAGAATTGTAG--TCTCTTATTTACCAA

GAAAGCAT---GCAAGAACTGCTAACTCATGC-TACCGTGACTGAACTCA

CGGCTT--------------------------------------------

-------------------TCTTA-ACTTCTATAGGATAGAAGCA-ATCC

GTTGGTCTTAGGAACCAAAAA-A-TTGGTGCAACTCCAAATAGAAGTAAT

TAACA---TATTCTCTTCACTTATTATCATATCACTTATAACCCTATCAT

TTCCTATTCTCCTAACCATAACTAACCACCATAAATATATTAACTTCCCA

AACTACGTAAAAACCTCCATTATTTGTGCATTATCGTTCTGCATTATGCC

AACACTAATATTTATTAATTCAAATTATGAACTCATTATCTCAAACTGAC

ATTGAATAACTATCCAAACACTCACCCTTTCCATAAGCTTTAAACTGGAT

TACTTTTCCATATTATTTATACCTGTAGCATTATTCGTCACATGATCAAT

TATAGAATTCTCAATGTGATATATGCACTCTGACCCCTTTATTAACCGTT

TCTTTAAATATCTCCTTTTATTCCTTATCACTATAATAATTTTAGTTACA

GCTAACAACTTGTTTCAACTATTTATCGGCTGAGAAGGAGTAGGCATTAT

ATCTTTCTTATTAATTGGCTGATGATACGGCCGAACAGACGCTAATACAG

CAGCCCTTCAAGCTATCCTATATAACCGAATTGGAGATATTGGGTTTATT

CTAGCCATGGCATGATTTTTACTTAACTCAAACTCATGAGAACTACAACA

ACTCTTCATCACAGATGTCTCTCTA------TTCCCCCTACTAGGACTAC

TCTTAGCCGCTACAGGAAAATCTGCCCAATTTGGTCTTCATCCTTGACTA

CCCTCCGCCATAGAAGGCCCAACCCCTGTTTCAGCCTTACTCCACTCCAG

CACAATAGTAGTGGCAGGAATTTTCCTCCTCATCCGCTTCTACCCATTAA

TA-GAACATAACAAGACTATCCAAACACTTACTCTCTGTCTAGGAGCTAT

TACCACCCTATTCACCGCTATCTGTGCTCTAACTCAAAACGACATTAAAA

AAATTATCGCATTCTCTACTTCAAGCCAACTAGGATTAATAATAGTAACT

ATTGGAATTAATCAACCCCATTTAGCATTTCTTCACATCTGCACACATGC

ATTCTTCAAAGCTATACTATTCATATGCTCAGGATCAATTATTCACAACC

TAAACAATGAACAGGACATTCGAAAAATAGGAGGCCTATTTAAAGCCCTT

CCATTCACTTCATCCTCACTTATTATTGGCAGTCTAGCATTAACAGGAAC

TCCCTTTCTAACGGGATTTTATTCTAAAGATCTAATTATTGAGTCTGCTA

ACACGTCGAACACCAACGCCTGAGCCTTAATTATCACTCTCCTCGCCACT

TCCCTAACCGCTGTCTACAGTACACGAATTATCTTTTATGTTCTAATAGA

ACAACCCCGATTTTCCACACTAACTTCAATTAATGAAAATAACCCTCAAC

TGCTTAATTCAATTAAACGCCTTCTTATTGGCAGTATCATTGCAGGATTC

ATCCTTTCATATAATATCCCACCTATAAATATTCCAGTATTAACCATACC

CACTTACCTAAAACTTACAGCATTATTAGTTACCATCTTAGGGTTTGCCA

TCGCTATAGAACTAAACTCAATAACCCTCTACCTCCAAACCAAAATATAC

TCAAATATAACA-AAATTTTCAACCTTACTAGGCTATTTTCCTACTATCA

TTCACCGACTTAACCCCCATCTTAATCTTATTATAAGCCAAAAAATATCA

TCAACCCTTCTAGACCTAATCTGACTAGAAAATACTATCCCTAAACTCAC

CGCTAATCTTCAT---TCAATAGCCTCTACCATATCCTCTAACCAAAAAG

GCCTCATCAAATTATATTTCTTGTCATTTTTAATTTCAACACTCTTAGCA

ACCATCTCCATATTCTA---------------------------CTTCCA

CGTGTAATCTCAATTACAATAAAAATACTAACAAACAACGATCAACCAGC

CACAACTATTAATCAACTTCCACAACTATATATGGCCGCCACCCCTATTG

AATCTTCACGAATCAATCCCAACTCACCCCCCTCAAATACTATTCAGCTC

CCTGAATCCTTAAACTTAACTACAATCTCTA---------CCTCATCATA

TAAAATTATAAGTATAATAATCAAGAACTCCACTAGAAACCCCAGCAATA

AAACTCCTCAAATAACCACATTTGACCCTCACGTTTCTGGATATTCCTCC

GTTGCTATAGCCGTAGTATAACCAAACACCACCAACATTCCTCCCAGATA

AATTAAAAATACTATTAAACCTAAAAAAGATCCCCCAAAATATAACACGA

CCCCACATCCAATTCCCCCACTAATAATCAATCCTAAGCCTCCATAAATA

GGAGAAGGCTTTGAAGAAAATCCCACGAAACCTAAAACAAAAAGTATACT

TAATAAGTACATAGTATATGTCATTAT---TTTTA-CATGGAGCTTA-AC

CATGACTAATGACATGAAAAATCATCGTTGT-TATTCAACTATAAAAACA

-CT----AATGACAAACACCCGCAAAACCCACCCTCTAATAAAAATCATT

AATCGCTCCCTTATTGATTTACCCGCACCCTCTAATATTTCAACATGATG

AAACTTTGGATCTCTACTAGGACTTTGCTTAGCTATCCAAATCTTTACCG

GATTATTTCTAGCAATACACTACACATCGGATACCATAACAGCCTTCTCA

TCCGTCACCCACATCTGTCGAGACGTGAATTACGGTTGACTCATCCGCTA

TATACATGCTAACGGTGCATCAATATTTTTTATCTGCCTCTTTCTTCATG

TAGGTCGAGGAATATACTATGGCTCATACACCTACTTCGAAACATGAAAC

ATTGGAGTTATTCTTCTACTTGCAGTAATAGCCACAGCTTTCATAGGCTA

CGTTCTCCCCTGAGGCCAAATATCATTCTGAGGGGCAACTGTAATTACTA

ATCTCTTATCTGCTATCCCATACATCGGTACAACCTTAGTTGAATGAATC

TGAGGTGGATTCTCAGTAGACAAAGCCACTCTAACACGATTCTTTGCATT

TCACTTCGTTCTCCCTTTTATCATCGCAGCCCTGGTCATAGTCCATCTCC

TTTTTCTTCACGAAACTGGATCAAACAACCCCTCAGGCCTTATTTCTAAC

TCAGATAAAATCCCCTTTCACCCGTACTTTACCATCAAGGATGTCCTTGG

AATCCTTCTCCTTATTTTAATTCTAATAATCCTAGTCTTATTTTCACCCG

ACCTCCTAGGAGATCCTGACAATTATACACCTGCAAATCCCTTAAGTACA

CCACCCCATATTAAACCAGAATGATACTTTCTATTTGCCTATGCTATTCT

ACGATCCATCCCCAACAAACTAGGAGGAGTTTTAGCCCTAGTCTCCTCAA

TTCTTATTCTAATACTCTTCCCACTGCTTCACCTATCTAAGCAACGCAGC

ATAATATTTCGACCATTAAGTCAATGTACATTTTGAATCTTAGTAACAGA

TCTAATTACACTAACCTGAATCGGAGGACAACCTGTTGAATATCCATATA

CCATTATCGGCCAATTAGCATCAATTCTATACTTCGCTATTATCCTCTTA

ATCTTACCAGCCATTAGCCTAATTGAAAACAAACTTCTTAAATGAAGA--

--GCCCTAATAGTATAT-AA--ATTACTTTGGTCTTGTAAACCAAAAATG

AAGTT-ATAAGC--TTCTTAGAGCAATATCTCAGGGAAGAAAATACACTT

TTCACCTTCAACTCCCAAAGCTGATATTTCTTACTTAAACTATTCCCTG-

--------------------------------------------------

--------------------------------------------------

--------------------------------------------------

--------------------------------------------------

--------------------------------------------------

--------------------------------------------------

--------------------------------------------------

--------------------------------------------------

--------------------------------------------------

--------------------------------------------------

--------------------------------------------------

--------------------------------------------------

--------------------------------------------------

--------------------------------------------------

--------------------------------------------------

--------------------------------------------------

--------------------------------------------------

--------------------------------------------------

--------------------------------------------------

--------------------------------------------------

--------------------------------------------------

--------------------------------------------------

--------------------------------------------------

------

>Urocitellus_parryii Urocitellus parryii mitochondrion, complete genome.

GTTAATGTAGCTTAA--TC----AAATAAAGCAAAGCACTGAAAATGCTT

AGATGGGTATTTTA--ACCCCATGAACATATA--GGCTTGGTCCTGGCCT

TTTTATTAGCTGTTAGCTGACTTATACATGCAAGCATCCCCGCCCCAGTG

AGAATGCCCT-CTATATCTATTAAT---CGATCAAAAGGTGCAGGCATCA

AGTTCACT-TATCCTAGTAGCTCACAACGCCTTGC-TCCACCACACCCCC

ACGGGATACAGCAGTAATTAGAATTAAGCCCATAAACGAAAGTTTGACTA

AGTTAAGCTA--AATT-AGGGTTGGTAAATTTCGTGCCAGCCACCGCGGT

CATACGATTAACCCTAGTTAACAAAGT-AC-GGCGTAAAGCGTGATTAAG

AGA------CTAGT-TAGATAAGATTAAAGTAGTACTAAACCGTAAAAAG

TC-TTGGTACTAATAAAAATCAAATACGAAAGTAATCTTA---AACTTTC

TGAATTCACGATAGCTAAGACCCAAACTGGGATTAGATACCCCACTATGC

TTAGCCCTAAACATAAATA-TTCAAC-AAACAAGAATATTCGCCAGAGAA

CTACTAGCAATAGCCTAAAACTCAAAGGACTTGGCGGTGCTTTACACCCC

TCTAGAGGAGCCTGTTCTATAATCGATAAACCCCGATACACCTCACCACC

TTTAGCAAATATCAGCCTATATACCGCCATCTGCAGCAAACCCTAA-AAA

GGCCTCACAGTAAGCAAGAAAATTCT--ACATTAGTACGTTA-GGTCAAG

GTGTAGCCTATAAGGTGGG-AAGAAATGGGCTACATTTTCTACTTCTCTA

GAATA------AATATCCACGATAGCTTTCATGAAACTTAA---AGCATA

AGGCGGATTTAGTAGTAAGTC-AAGAATAGAGAGCTCGACTGAATAG-GG

CAATAAAGCACGCACACACCGCCCGTCACCCTCTTCAAATATACCT----

GTACAAAACTATAAATAATCTATTTTACCAAA--T--ATATA--AGAAGA

GATAAGTCGTAACAAGGTAAACATACTGGAAAGTGTGTTTGGAAGAATCA

AAATGTAGCTTA--TTAACTAAAGCACCCGGCTTACACCCGAGAGATTTC

ATTTTATTATGAACATTTTGA-ACC-AATGCT-AGCCCAACTTTAC----

-CCCCATCCAAATACTACTCACCCAACAAATAAAACATTCACC-TA-AA-

--TAAA-GTATAGGAGATAGAAATTTA--TACCA--GGAGCTATAGAGAA

AGTACCGCAAGGGAAAGA-TGAAAGAATAAATTTATAGTATTAAAAAGCA

AAGACTAACTCTTTTACCTTTTGCATAA-TGATTTAACCAGAAAATGCTT

GACAAAAAG-AATTTAAGCCAAACACCCCGAAACCAGACGAGCTACTCAT

AAGCAGCT--AATAAGAGCTAATCCGTCTATGTTGCAAAATAGTGGAGCG

ACTTATAAGTAGAGGTGAAAAGCCTACCGAGCCTGGTGATAGCTGGTTAT

CCA-GACTAGAATTTTAGTTCTACTTTAAATTTACCTAAAGCATA-AAT-

AG-GCCGAATGTAAATTTAGATGTTATTCTAAAGAGGGACAGCTCTTTAG

AGTTAAGGAATAAACCTTATTTAGAGAGTAA-----ATCAATCAATT--T

CCATAGTTGACTTAAAAGCAGCCATCAATTAAAAAAGCGTTAAAGCTTAA

CCTAATCA-T---AAAAACTTAATACCTAACTCT---TTCAGTAATCT--

CCTAAATCAACA-CTGGATTAATCTATTT-ATAAT-AGAAGAAATTATGT

TAAAATAAGTAACAAGAA-AATAT-T-CTCCCTGCATAAGCTTATATCAG

ATCGAA-TAATTCACTGATAGTTAACAACCTCATA-----ATATTAAACA

AAACCATAAATCATTATTATTTACATTGTTAACCCAACACTGGCATGCA-

--TTAAGGGAAAGATTAAACAAAGTAAAAGGAACTCGGCAAACATTAACC

TCGCCTGTTTACCAAAAACATCACCTCTAGCATAATTAGTATTAGAGGCA

CTGCCTGCCCAGTGACAATACG----TTCAACGGCCGCGGTATCCTGACC

GTGCAAAGGTAGCATAATCACTTGTTCTTTAAATAAGGACTAGCATGAAT

GGCTTGACGAAGGTTTAACTGTCTCTTACTTTTAATCAGTGAAATTGACC

TTCCCGTGAGGAGGCGGGAATTTTATAATAAGACGAGAAGACCCTATGGA

GCTTTAATTTAAC-AGTCTCACAACCTTAATAGTATCTT----------A

AGAATTAA-AATTACTGTTTA-TAGACTAGAAATTTTGGTTGGGGTGACC

TCGGAGTATAAATCAACCTCCGAATGATA-ATAATCT-AGACTCTACATG

TCCAAATTACAACTC----ATAAATTGACCCAG-A---ATACTGATCAAC

GGAACAAGTTACCCTAGGGATAACAGCGCAATCCTACTCAAGAGTCCATA

TCGACAGTTAGGGTTTACGACCTCGATGTTGGATCAGGACATCCAAATGG

TGTAACCGCTATTAATG-GTTCGTTTGTTCAACGATTAA-AGTCCTACGT

GATCTGAGTTCAGACCGGAGAAATCCAGGTCGGTTTCTATCTATTTCTA-

TATTTCTCCCAGTACGAAAGGACAAGAGAAATAAGGCCAATTAAACAC-T

TATGCCTTAAATTTAATGGATGAG-ACAATCTTAATCCAGTAAAATATTT

TAAAC--AACCTGCCCTAGAAAAGGG--CTT-GTTAAGATGGCAGAGCCT

GGTAA-TTGCGTAAGACTTAAAACTTTATT-TCAGAGGTTCAACTCCTCT

TCTTAACA-TTA--------------------------------------

------------------------------------------ATGTTTAT

AATTAATCTTCTACTTTTAATTATCCCAATCCTAGTAGCCATAGCTTTTC

TTACCCTAATCGAACGAAAAATATTAGGATATATACAACTCCGCAAAGGC

CCCAATGTTGTTGGACCTTACGGCCTACTTCAACCATTTGCTGACGCAAT

AAAGCTATTCATTAAAGAGCCTATAAAACCCCTAACATCGTCAATTATAT

TATTTATTATTGCTCCAACCCTAGCCCTGACACTAGCTTTTACCATATGA

ATTCCCCTACCCATACCAATACCCCTCATTAACATAAACATAGGAGTGCT

ATTTATCTTAGCCACATCAAGTCTAGCCGTATATGCAATTCTATGATCTG

GGTGAGCATCCAACTCTAAATATGCTTTGATTGGAGCCCTACGAGCCGTA

GCACAGACCATCTCGTATGAAGTAACATTAGCAATCATCCTTCTCTCAGT

ACTTCTAATAAATGGATCATTTACCCTATCCACACTTATCACCACCCAAC

AATTTACATGACTGCTACTTCCAACATGACCTCTAGCAATAATGTGATTT

ATTTCAACATTAGCAGAAACTAATCGAGCTCCATTTGATCTAACAGAAGG

AGAATCAGAACTTGTATCAGGATTTAATGTTGAGTACGCAGCCGGTCCAT

TCGCCTTATTCTTTATAGCCGAATATACTAACATTATCATAATAAACGCA

TTAACAGTAACTCTTTTTATAGGAGCACTACTAAATCCCATCTTTCCCGA

AACTTTTACATTAAACTTCACCTTAAAAACACTCATCTTAACTTCCACTT

TCCTATGAATCCGGGCATCCTATCCTCGATTCCGTTACGACCAACTCATA

CATCTTTTATGAAAAAACTTCTTACCCCTAACCCTAGCCTTATGCATATG

ACACATCTCTCTTCCAATTATAACTGCATGCGTACCACCCCAAATCTAA-

GAAATATGTCTGATAAAAGAGTTACTTTGATAGAGTAAATTATAGAGGTT

TA-AATCCTCTTATTTCTAGAACTATAGGAATTGAACCTAATCCTAAGAA

TTCAAAATTCTTCGTGCTACC-TTT-TACACCACGTCCTAAAT--A----

GTAAGGTCAGCTAATT-AAGCTATCGGGCCCATACCCCGAAAATGTTGGT

TTATATCCTTCCCGTACTAATCAATCCCTTAACTTCCTCCGCAGTCTACT

TTACCCTCTTTTCTGGAACTATAATTACACTTTTTAGCTCACATTGACTC

CTAACTTGAGTAGGTCTAGAAATAAGCATATTAGCTATCACCCCTATTCT

AATCAATAAAGGAAACCCCCGATCTACAGAAGCTGCATGCAAATACTTTC

TCATTCAAGCCACCGCATCAATAATCTTAATAATAGGCACAATAATTAAC

TTTATAGACTCAGGCCAATGAACCCTATCTAACTCATATAATCAAATTTC

ATCATTCATATTTACAATTGCACTCTCAATAAAAATAGGACTAGCTCCCT

TTCACCTATGAGTCCCAGAAGTCACCCAAGGAATTCCACTTATATCAGGC

CTAATCATGTTAACATGACAAAAAATTGCCCCAATCTCTATCGTTTATCA

AATCGCACCTTCCATAAACCCTACCCTTATGCTACTTTTAGGAATTCTAT

CAATCATACTAGGAGGCTGAGGAGGACTTAACCAAACCCAACTACGAAAA

ATCCTAGCGTATTCATCAATTGCCCACATAGGATGAATAATAGCAATCGT

TACATATAATCCAACCTTAACAATATTTAACCTAATTATTTATATCATTC

TTACTATCAACATATTTATACTTCTTCTTTTCCATAAAAAAACTACTACC

CTCTCCCTATCTAACTTATGAAATAAATTCCCTCTTTTAACACCCACAAT

TCTAATTGTACTAATATCGCTAGGAGGATTACCTCCTCTAACAGGATTTA

CACCAAAATGAATTATCCTTAAAGAACTTATCTCAAATAACAACATTATT

TTCTCTACACTAATAGCGATATTCGCACTCCTAAACTTATATTTCTATAC

ACGACTTATCTACTCAGCATCTCTAACTTTATTCCCATCATTCAACAATA

CCAAAATAAAATGACAATTCGAGAACACAAAGCTTATACCCATATTATCC

ACTCTAATCATTACTTCCACCCTCTCCCTCCCACTAATACCCCTC-TTCT

CACTCCT--GAACT----AGGAATTTAGGTTAAT--T-CAGACCAAGGAC

CTTCAAAGTCCTAAGCAAGTA-CCTAAT---ACTTAATTCCTGC------

---A----TTAAGGACTGCAAGACTTTATCTTACAT----CAATTGAATG

CAAACCAATCACTTTAA--TT-AAGCTAAGCCCTTC-ATT------CCTA

GACTGATG--GGATTTAAA-CCCAT-AAGATCTTAGTTAACAGCTAAACG

CCTTACTCAACTGGCTTCAATCTA-CT-TCTCCCGCCGTTAAGGGAAAAA

-----GGCGGGAGAAGCCCCGGCAGAG-T-TGA-AGCTGCTCCTTT--GA

-----------------------------ATTTGCAATTCAATA--TGAA

TA-TTCACCTCGGGACT-T-----GGTAAAAAGAGGG----TT-CAACCT

CTGTCTTTAGATTTACAGTCTAATGCTTA--CTCAGCCATTTTACC----

ACCTACTTATGTTCATCAACCGTTGATTCTTCTCAACAAATCACAAAGAT

ATCGGTACACTCTACCTTCTATTTGGTGCTTGAGCTGGAATAGTAGGAAC

TGCGCTTAGTCTACTAATCCGAGCTGAACTAGGTCAACCCGGAGCCCTAT

TAGGTGATGATCAAATTTACAATGTTATTGTCACCGCTCATGCATTTGTT

ATAATTTTCTTTATAGTTATACCAATTATGATTGGTGGATTTGGAAACTG

ACTAGTCCCCTTAATAATTGGAGCTCCTGACATAGCATTCCCACGTATAA

ATAATATAAGCTTCTGACTTCTACCCCCTTCTTTCCTTCTCTTACTCGCT

TCTTCTATAGTTGAAGCAGGTGCAGGAACTGGTTGAACTGTTTATCCTCC

ATTAGCCGGAAACCTTGCCCATGCAGGGGCTTCAGTGGACCTAACCATCT

TCTCTCTTCACTTAGCAGGAGTTTCATCAATTCTAGGTGCAATTAACTTT

ATTACAACTATTATCAACATAAAACCACCTGCTATATCTCAATATCAAAC

TCCTTTATTTGTATGGTCCGTATTAATTACAGCAGTACTGTTACTCCTGT

CCCTCCCAGTTCTTGCAGCAGGAATTACTATGCTGCTAACAGACCGTAAT

CTTAACACCACATTCTTTGATCCTGCTGGAGGTGGAGATCCAATCCTCTA

TCAACACCTATTCTGATTTTTTGGACACCCTGAAGTTTACATTCTTATCC

TACCAGGATTTGGTATAATTTCTCATATCGTAACATATTACTCAGGAAAA

AAGGAACCATTCGGTTATATAGGTATGGTATGAGCTATAATATCTATTGG

CTTCCTTGGATTTATCGTATGAGCCCATCATATATTTACCGTTGGAATAG

ATGTTGACACTCGAGCCTACTTTACATCTGCAACCATAATTATTGCTATT

CCTACAGGAGTAAAAGTTTTTAGTTGACTAGCAACTCTACACGGAGGAAA

TATCAAATGATCGCCCGCAATACTATGAGCACTAGGCTTTATTTTCCTAT

TCACTGTAGGAGGCCTTACAGGAATTGTCTTAGCCAATTCTTCACTAGAC

ATTGTTTTACACGATACATACTATGTCGTAGCCCACTTCCACTATGTGTT

ATCGATAGGAGCTGTATTTGCTATTATAGGAGGATTCGTTCACTGATTCC

CCCTTTTCTCCGGTTACACACTAAATGACCTTTGAGCTAAAATTCATTTT

ACTGTAATATTTGTCGGAGTGAATTTAACTTTCTTCCCTCAACATTTCTT

AGGACTATCAGGTATGCCACGCCGATACTCTGATTACCCAGATGCATACA

CAGCATGAAATACTGTTTCCTCAATAGGTTCATTCATTTCTCTTACAGCT

GTTATAATTATAATCTTTATAATTTGGGAAGCATTTGCATCAAAACGAGA

AGTTCTCACCGTAGAACTAACACCAACTAATTTAGAGTGACTACACGGGT

GTCCTCCACCCTATCACACATTTGAAGAACCCACTTACATTAAGGCCTAG

AT--C--------------AAGAAAGGAAAGAATCGAACTTTCTAAAACT

AGTTTCAAGCCAGCCTCATAACCATTATGA-CTTTCT---TTAT----GA

GATATTAGTAAAAT-AATTACATAACTTTGTCAAAGTTAATTTATAGGTT

A------GACTCCTATATATCTC-TATGGCATACCCCTTCGAATTAGGAT

TTCAAGACGCTACATCTCCTATTATAGAAGAACTTCTACACTTTCATGAC

CACACTCTTATAATTGTTTTCCTAATTAGCTCCCTAGTCCTTTACATCAT

TTCATTAATATTAACTACAAAATTAACTCATACAAGCACCATAGACGCTC

AAGAAGTAGAAACCATTTGAACCATTCTCCCCGCTATTATCCTTATCCTA

ATTGCTCTTCCCTCCCTACGTATTCTATATATAATAGATGAAATTAATGA

CCCAACCCTAACAGTAAAAACAATAGGTCACCAATGATATTGAAGTTATG

AATACACGGATTATGAGGACCTAAATTTTGATTCCTATATGGTTCCAACT

TCAGATCTAGCCCCAGGAGACCTACGACTTCTTGAAGTCGACAATCGAGT

TGTACTTCCAATGGAAATACCCGTACGAATGTTAATTTCATCTGAAGATG

TTCTTCACTCCTGAGCAGTCCCATCTCTTGGATTAAAAACAGATGCCATC

CCAGGCCGACTTAATCAAGCCACACTTACATCAACACGACCAGGACTTTA

TTATGGTCAATGCTCTGAAATTTGTGGGTCAAATCATAGCTTCATACCAA

TCGTTCTTGAATTAGTTCCGCTAAAGCATTTTGAAAACTGATCCTCATCA

ATACTATAA--------------------------------ATT-CATTA

TGAAGCTA--AAATAGCATCAACCTTTTAAGTTGAAGACTAGGAGT-T-A

A-AT-CTCCTCATAATGAAATGCCCCAACTAGATACATCCACATGATTTA

TTACAATTCTATCAA-TAATTCTAGCTCTTTTCTTTATGTTTCAACTTAA

AATCTCAAACCA--CTCTTACCCATCTAATCCCTCCCCTAAAGATACTAA

-ATTAATTGAGCATAAAACCCCTTGAGAAGAAAAATGAACGAAAATCTAT

TTGCCTCTTTCATTACCCCCACATTAGTAGGTCTTCCTATTGTCCTTTTT

ATCATTATATTCCCCAACTTACTTTTTCCTTCGCCTACCCGATTAGTAAA

CAACCGCTTAGTGTCATTCCAACAATGACTAATTCAACTTGTACT-AAAA

CAAATAATGGCAATGCACAACCCAAAAGGACGTACCTGATCCCTAATATT

AATCTCATTAATTATATTCATTGGCTCAACTAATCTTCTAGGATTAATAC

CTCACTCTTTTACACCAACAACCCAACTATCAATAAATTTAGGAATAGCT

ATCCCCTTATGAGCAGGAGCAGTAATTACTGGATTTCGTCATAAGA-CTA

AAGCATCATTAGCCCACTTTCTTCCACAAGGAACCCCAATTCCTCTTATC

CCCATACTAATTATTATCGAGACAATTAGCCTCTTTATCCAGCCTATAGC

GCTAGCTGTACGATTAACAGCCAACATCACAGCTGGCCATCTTCTCATAC

ATTTAATCGGAGGAGCAACTCTTGTATTAATATCTATTAGCCCTCCTACA

GCCATCATTACTTTCATTATTCTTGTACTATTAACAATGCTCGAATTCGC

AGTTGCACTAATTCAAGCTTACGTTTTCACCCTCCTAGTAAGCCTGTATC

TACATGATAATACTTAATGACCCACCAAACCCACGCCTACCATATAGTTA

ATCCTAGCCCTTGACCCTTAACAGGGGCCCTCTCCGCCTTACTCCTAACC

TCCGGCCTAGTAATATGATTCCATTTCAATACCTCCTTCCTACTCACACT

AGGCCTATTAGCCAATACTCTAACAATGTATCAATGATGACGAGATATTG

TACGAGAAGGTACATTTCAAGGTCACCATACATCAATTGTCCAAAAAGGC

CTACGATATGGTATAGTACTATTTATTATTTCAGAAGTATTCTTCTTTGC

CGGATTTTTCTGAGCATTCTATCACTCTAGTTTAGCTCCAACTCCCGAAC

TTGGCAGCTGCTGACCTCCAGTAGGAATCAACCCACTCAACCCCTTAGAA

GTACCACTATTAAATACCTCTGTTCTTTTAGCTTCAGGGGTTTCAATTAC

TTGAGCCCATCATAGCCTAATAGAAGGAGACCGAAAACATATAGTTCAAG

CACTATCAATTACAATTGCTCTAGGACTTTATTTTACTCTTCTCCAAGCT

TCTGAGTATCTTGAGACATCTTTTACAATTTCAGATGGTGTATATGGTTC

AACATTCTTTATGGCCACGGGCTTCCATGGTCTCCACGTTATAATTGGAT

CAACCTTCCTTCTAGTATGCCTCATTCGTCAACTAAATTTCCACTTTACA

TCAAAACACCACTTTGGATTCGAAGCAGCCGCATGATACTGACATTTTGT

AGATGTAGTATGACTCTTCCTTTACGTGTCTATTTATTGATGAGGCTCAT

ATTCT-CTTAGTATCAA-TTAGTACAATTGACTTCCAATCAATTAGTTCT

GGAACTAATCCAGAAGAGAATAATTAACCTTATATTATCCCTCATTGTAA

ATTCTTCCGTTGCTCTTCTATTAATCTCCGTAGCATTTTGATTACCCCAA

CTAAACGTATACGCTGAAAAGGCAAGTCCTTACGAATGCGGATTTGACCC

CATAGGATCTGCTCGCCTACCATTCTCAATAAAATTTTTTCTTGTTGCAA

TTACATTTCTCTTATTTGACTTAGAAATTGCTCTTCTTCTCCCCCTTCCC

TGAGCCTCTCAAACAAACAACCTCAGCCTTATATTAACTATAGCCTTGCT

CTTAATCTTAATTCTTACTCTCGGATTGGCCTACGAGTGAATTCAAAAAG

GCTTAGAATGAATTGAATA------TGATAATTAGTTTAAAGTAAAA-CA

AGTGATTTCGACTCACTAAATTATGGGCTA-C--CATAATTATCAA--AA

TGCCTATTATTATCCTCAACACTATTTTAGCCTACTCTACATCTCTATTA

GGAATATTTATTTACCGATCTCACCTAATATCATCACTTCTATGCTTAGA

AGGGATAATATTATCAATATTTGTCTTATGCTCCCTTTTAATTATAAATT

TTCACTTCTCCTTATCATTTATAATCCCCATCACTTTACTAGTATTTGCT

GCATGCGAAGCAGCTGTAGGCCTAGCCCTTCTCGTAATAGTATCTAATAC

ATATGGTCTAGACTATGTCCAAAACCTAAACATTCTTCAATGTTAAAAAT

TATTATCCCCACAATTCTACTTGCTCCCCTTATATGGCTCTCAAAGCCTT

CCATAATCTGAATTAACCCTTCAATTCACAGTCTGATAATTAGCCTAATT

GTTCTTTCTACATTAAATCGTCCTATAAATATAGATCTGATTCTCTCATT

AACCTTCTTCACTGATCCTTTATCCTCTCCTCTACTAATTTTAACAGCAT

GACTCCTACCTCTCATAATTATAGCAAGCCAAAGCCATTTAACCCATGAA

CCATTAATCCGAAAAAAACTATATATTCTTATATTAATCTCTCTACAATC

CTTTTTGATTATAACTTTCTCTGCCACTGAACTAATTATATTCTATATCC

TATTTGAAGCTACCCTAATCCCTACACTAATTATTATCACTCGATGAGGA

AACCAAACTGAACGATTAAATGCAGGATTATATTTTCTATTTTATACCCT

GGTCGGTTCGTTACCCTTACTAGTAGCATTAATTTATATCCAAAAATCTA

CAGGATCCTTAAATTTTATTATATCAATATATCAATCATCTAATCTTCCT

ATATCT----------TGAACAAATGACATTCTGTGACTAGCATGTATTA

TGGCCTTTATAGTTAAAATACCTCTATACGGCCTCCACCTCTGATTACCA

AAAGCCCATGTTGAAGCTCCTATTGCTGGTTCTATAGTTTTAGCCGCTAT

CCTGCTAAAACTCGGTGGATACGGAATAATTCGAATTTCAACTTTACTTC

ATCCCATTACATGTAACATAGCCTACCCCTTTATTATATTATCGCTATGA

GGAATAATCATAACAAGCTCAATCTGCCTACGACAAACAGACCTAAAATC

TCTCATCGCTTACTCCTCAGTAAGTCATATAGCACTAGTAATTGTAGCAA

TCATGATTCAAACTCCCTGAAGCTTTATAGGAGCCACAGCACTAATAATT

GCCCACGGACTTACATCTTCTATGCTATTCTGTCTAGCAAACACCAATTA

TGAACGAATTCACAGCCGAACTATAACACTAGCCCGAGGCTTGCAATCTA

TCCTTCCCCTTATAGCAACATGATGAGTTCTAGCCACCCTAACTAATTTA

GCCCTCCCACCTTCTATTAATCTAATCGGTGAATTATTCATTGTTATAGC

ATCATTCACTTGATCGAATATAACAATTATCTTAACTGGACTAAACATAT

TAATCACAGCCCTCTACTCATTATATATACTAATTATAACACAACGAGGA

AAATTTACGTACCATACATTAAATATTAATCCTTCTTTCACACGAGAAAA

TACACTTATATTTCTTCACCTCTTTCCACTTGCCATTCTATCAACAAACC

CTGCCATTATTCTGGGTCAATCATACTGTAAATATAGTTTAAGCAAAACT

TTAGATTGTGAATCTAACAATAGAGAATCATAA--CCTCTTATTTACCAA

GAAAGCAT---GCAAGAACTGCTAATTCATGC-CCCCGTGATTACACCCA

CGGCTT--------------------------------------------

-------------------TCTTA-ACTTTTTTAGGATAGTAGTA-ATCC

GTTGGTCTTAGGAACCAAAAA-A-TTGGTGCAACTCCAAACAAAAGTAAT

AAATA---TATTCTCTTCACTTATCCTTACATCACTCGTAACCCTCTTAT

TTCCTATTTTTCTCACCATATCAGACTACCATAAACACGTTAATTACCCA

AACTACGTAAAAATCTCTATTATCTGTGCACTATCATTTTGCATAGTACC

AACACTAATGTTTATTAACTCAAATTATGAACTTATCATCTCAAACTGAC

ACTGAATGACTATCCAAACATTCACCCTTTCCATAAGCTTTAAACTAGAT

TATTTCTCTATATTATTCATTCCCGTAGCATTATTCGTTACATGATCAAT

TATAGAATTCTCAATATGATACATGCACTCTGACCCTTTTATTAACCGTT

TCTTCATATATCTCCTCTTATTCCTTATCACTATAATGATTCTAGTTACA

TCTAACAACCTATTTCAACTATTTATTGGCTGAGAAGGGGTAGGTATTAT

ATCTTTCTTATTAATCGGCTGATGGTATGGTCGAACAGACGCTAATACAG

CAGCCCTTCAAGCTATTTTATATAACCGAATTGGAGACATTGGGTTTGTT

CTAGCTATAGCATGATTCTTACTTAACTCAAATTCATGAGAACTTCAACA

GCTATTCATAATAGACGTGTCCTTA------TTTCCTCTACTAGGACTTC

TCTTAGCCGCCACAGGAAAATCTGCCCAATTCGGCCTTCACCCTTGATTG

CCCTCTGCTATAGAAGGCCCAACCCCCGTATCAGCTTTACTTCATTCCAG

TACAATAGTAGTAGCAGGAGTCTTTCTCCTCATCCGCTTCTACCCGCTAA

TA-GAACATAATAAAGTCATCCAAACACTCACTCTCTGCTTAGGAGCTAT

TACCACTCTATTCACCGCCATTTGTGCCCTTACTCAAAATGACATTAAAA

AGATTATCGCATTCTCCACTTCAAGCCAACTAGGATTGATAATAGTGACT

ATTGGAATTAACCAGCCCCACCTAGCCTTTCTCCATATTTGCACACACGC

ATTCTTTAAAGCCATACTATTTATATGCTCAGGATCAATTATCCATAACT

TAAACGATGAACAAGACATTCGAAAAATAGGAGGACTATTTAAAGCTCTC

CCATTTACTTCATCCTCACTTATCATCGGCAGCTTAGCACTAACAGGAAC

TCCATTCCTAACCGGATTTTACTCTAAAGACCTAATTATCGAATCTGCAA

ACACGTCATATACCAACGCCTGAGCCCTAGCTGTTACTCTCTTTGCCACC

TCTCTAACCGCTGTCTATAGCACACGAATTATTTTCTACGCCCTTATAGG

ACGACCTCGATTCTCTTCACTAACCTCAATCAACGAAAATAACCCCCAAC

TACTTAACTCAATTAAACGCCTTCTTATTGGAAGTATTATTGCAGGATTT

ATCCTTTCATATAATATCCCACCTATAAATATCCCAGTCTTAACCATACC

CATCTATCTTAAACTCACAGCACTACTAGTAACCATCTTAGGATTTGTCA

TCGCTATAGAACTTAACTCAATAACTCTTTATCTAAAAACTAAAATGTAC

TCAAACACATCA-AAATTTTCAACCTTACTAGGCTACTTCCCTACTGTTA

TTCACCGACTTAATCCCCATCTTAATCTCATCATAAGCCAAAAACTTTCA

TCAACCCTATTAGACCTGGTCTGACTAGAAAAAACTATCCCCAAATCTAC

CACCAACTTTCAC---TCAACAACCTCCACTATAACCTCTAACCAAAAGG

GCCTCATCAAATTATATTTCCTATCGTTCCTGACTTCAACACTTCTAGCA

ACCACAGCCGTATTCTA---------------------------TTTCCA

CGTGTAATTTCAATCACAATAAAAATACTAACAAACAATGATCAACCAGC

TACAACCATCAATCAACTTCCACAGTTATATATAGCTGCCACCCCTATTG

AATCTTCACGAACCAATCCTAACTCACCCCCCTCAAACACTATTCAATTC

CCTGAATCCTTAAACTCAATTACAACTTCCA---------TCTCATCATA

TAGAACCATAAACATAATAATTAAAAACTCCACTAAAAACCCCAATAACA

AAACCCCCCAAATAACTACATTTGATCCTCACGTTTCTGGATATTCCTCC

GTTGCTATAGCCGTAGTATAACCAAATACCACTAATATTCCCCCCAAATA

AATTAAAAATACTATTAAACCTAAAAAAGACCCTCCAAAATATAATACAA

CCCCACACCCAATTCCCCCACTAACAATTAACCCTAAACCTCCATAAATA

GGAGAAGGTTTTGAAGAAAATCCTACAAAACCTAAAACAAAAAGTATACT

TAATAAATATGTTACATATGTCATTAT---TTTTA-CATGGAATTTA-AC

CATGACTAATGACATGAAAAATCATCGTTGT-TATTCAACTATAAAAACG

-CT----AATGACAAACATCCGCAAAACTCACCCTTTAATTAAAATCGTC

AACCACTCCTTTATCGACTTACCTGCACCCTCCAACATCTCTGCATGATG

AAATTTTGGGTCTCTTCTAGGCCTCTGCCTAGCAATCCAAATTCTCACTG

GATTATTTCTAGCAATACATTATACATCTGATACTATAACAGCCTTTTCA

TCAGTTACTCACATTTGCCGAGATGTAAATTATGGTTGACTAATCCGCTA

TATACATGCTAACGGCGCATCTATATTTTTTATCTGCCTCTTTCTTCATG

TAGGCCGAGGACTGTACTATGGCTCATACACTTACTTTGAAACATGAAAC

ATTGGAGTCATTCTCCTGTTCGTAGTAATAGCCACAGCTTTCATGGGCTA

TGTTCTTCCCTGAGGTCAAATGTCATTCTGAGGAGCAACTGTAATTACTA

ACCTTTTATCCGCTATTCCATACATTGGAACAACCCTAGTAGAATGAATT

TGAGGCGGCTTCTCAGTAGATAAAGCTACTCTAACACGATTTTTCGCATT

CCACTTTATTCTTCCATTTATTATCGCAGCTCTAGTCATAGTTCACCTTC

TTTTCCTTCACGAAACTGGATCAAACAACCCTTCAGGCCTTATTTCTGAT

TCAGATAAAATCCCCTTTCACCCGTACTACACCATCAAAGATATCCTTGG

AGTCCTTCTTCTTATTTTAGCCCTAATAACTCTAGTCCTGTTTTCACCTG

ATCTTCTAGGAGACCCTGATAATTATACACCCGCAAACCCCCTAAGCACC

CCACCTCACATCAAACCAGAATGATATTTCCTATTTGCCTACGCTATCCT

CCGATCTATCCCCAACAAACTAGGAGGTGTATTAGCCTTAGTACTCTCAA

TTCTCATCCTAATACTTTTCCCACTACTCCATTTATCTAAACAACGAGGC

ATAATATTTCGACCACTAAGTCAATGCATATTCTGAATTCTAGTAGCAGA

CCTATTTACATTAACCTGAATTGGAGGACAACCCGTTGAATACCCATTTA

TCATTATTGGCCAACTAGCATCAATCCTATATTTCACCATTATTCTCCTA

ATCTTACCAACCGTCAGCCTAATTGAAAATAAACTTCTTAAATGAAGA--

--GCCCTAATAGTATAA-AC--ATTACTTTGGTCTTGTAAACCAAAAATG

AAGTT-ACAAAC--TTCTTAGAGCAATAAATCAGGGAAGAAAATAAACTT

TCCACCTTCAACTCCCAAAGCTGATATTTCTTACTTAAACTATTCCCTG-

--------------------------------------------------

--------------------------------------------------

--------------------------------------------------

--------------------------------------------------

--------------------------------------------------

--------------------------------------------------

--------------------------------------------------

--------------------------------------------------

--------------------------------------------------

--------------------------------------------------

--------------------------------------------------

--------------------------------------------------

--------------------------------------------------

--------------------------------------------------

--------------------------------------------------

--------------------------------------------------

--------------------------------------------------

--------------------------------------------------

--------------------------------------------------

--------------------------------------------------

--------------------------------------------------

--------------------------------------------------

--------------------------------------------------

------

>Cheirogaleus_medius Cheirogaleus medius mitochondrion, complete genome.

GTTAATGTAGCTTAA---------ATTAAAGCAAGGCACTGAAAATGCCT

AGACGGGT--TATATAGCCCCATAAACATATA--GGTTTGGTCCCAGCCT

TATTATTAGTTATCAGTAAGATTACACATGCAAGTAACCGCACGCCAGTG

AGAATGCCCT-CTAGATC-----TTAATCTATCAACAGGAGCAGGTATCA

AGCACGCT---AAACAGCAGCTCACCACACCTTGC-TAAACCACGCCCCC

ACGGGATACAGCAGTGATAAAACTTAAGCA-ATAAACGAAAGTTTGACTA

AGCTATACTGACAAATTAGGGTTGGTCAATTTCGTGCCAGCCACCGCGGT

CATACGATTAACCCAAACTAATAAAAT-AC-GGCGTAAAGTGTGTTTAAG

ATATCTG--------ATAATAAAGTTAAATTTTAACTAAGTTGTAGAACA

CTTCAGCTAA-AACAAAAATAATCCACGAAAGTGACTTTA----ATATCC

TGAAAACACGACAGCCAAGACCCAAACTGGGATTAGATACCCCACTATGC

CTGGCCATAAACATAAGTAACTTATG--AACAACCTTACTCGCCAGAGAA

CTACAAGCAATAGCTTAAAACTCAAAGGACTTGGCGGTGCTTTATATCCC

TCTAGAGGAGCCTGTTCTATAATCGATAAACCCCGATAAACCTCACCATC

CCTTGCTAAT-TCAACCTATATACCGCCATCCTCAGCGAACCCT-ACTAA

GGAAACAAAGTAAGCACAAACATTC---GACTAAAAACGTTAAGGTCAAG

GTGTAGTCTATGAGATGGG-AAGAAATGGGCTACATTTTCTAACACCAGA

ATA---------ATCTTCACGGCAACCTTCATGAAATAAAA---GGCCAA

AGGAGGATTTAGTAGTAAGTT-AAGAATAGAGAGCTTAACTGAATAG-GG

CCATAAAGCACGCACACACCGCCCGTCACCCTCCTCAACTCTCAAT----

-TACCAATACTTAAATAACACAATGCTCTACA--------TATAAGAGGA

GATAAGTCGTAACAAGGTAAGCATACTGGAAAGTGTGCTTGGAACACTCA

AAGTGTAGCTTA----ACATAAAGCATTCGGCCTACGCCCGAAAGATTTC

ATAACAGCATGACCACTTTGAAACT-AACCCT-AGCCTAGTATCT-----

ACAACTAACAACAAATCCAAAAATCCTAATCAAACCATTTACCACAAGCC

AATAGCAGTATAGGAGATAGAAACTT-----TATTTAGCGCTATAGAGAA

AGTACCGTAAGGGAAAAA-TGAAAGAAGAAACCTAAAGTACAAAAAAGCA

AAGCTCACTCCTTGTACCTTTTGCATAA-TGACTTAACTAGAATA-ATTT

GACAAAAAG-AATTTCAGCCAAGGACCCCGAAACCAGACGAGCTACTTAT

AAACAGCTTTAATCAGAGCACACTCATCTATGTAGCAAAATAGTGAGAGG

ATTTATGAGTAGAGGTGAAAAGCCTACCGAGCCTGGTGATAGCTGGTTAT

CCA-GAAAAGAATTTTAGTTCAACTTTAAATTAACCTAAAGTAATACAAT

AAA-CCACATGTTAATTTAAATGTTATTCTAAAGAGGAACAGCTCTCTAG

ATC--AGGCTACAACCTTTAGTATAGAGTAA-------ATAATCAAATAT

TCATAGTTGGCCTAAAAGCAGCCACCAATTAAGAAAGCGTTCAAGCTCAA

C------ACACAACATCTCTTAATTTCACAATTC----CCTAACACCT--

CCTA-CTAACTTACTGGATTAATCTATTGTCTAAT-AGAAGAAATACTGT

TAATATAAGTAACAAGAATTCTAT-TTCTCCCAGCACCAGCCTATGCCAG

ACCGGAT--GTCCACTGGTAATTAACAACAAGATAAAATTATATATTACA

AATACACACT---TTATCCTAAAAACTGTTAACCCAACACAGGCGTGCG-

--CTTAAGGAAAGATTTAAAAAAGTAAAAGGAACTCGGCAAACACCTACC

CCGCCTGTTTACCAAAAACATCACCTCCAGCCTAATCAGTATTGGAGGCA

CTGCCTGCCCAGTGACA-----CATGTTTAACGGCCGCGGTATCCTGACC

GTGCAAAGGTAGCATAATCACTTGTTCTCTAATTAAGGACTTGAATGAAT

GGCAACACGAGGGTTCAGCTGTCTCTTACTTTCAATCAGTGAAATTGACC

TCCCCGTGAAGAGGCGAGGATACTACAATAAGACGAGAAGACCCTATGGA

GCTTAAATCAAATAATCCAAATATAATGCCCCTCAAACCTAAAAGCTGGT

ATAACCAAATAATATTAT----TGGATTAAAAATTTCGGTTGGGGTGACC

TCGGAGCACAACAAAACCTCCGAACAAC--TTTAACCTAGACCTAACTAG

TCAAGGT---ATACACATCATCAATTGACCCAA-AACTAATTTGATCAAC

GGAACAAGTTACCCTAGGGATAACAGCGCAATCCTATTAAAGAGTCCATA

TCGACAAT-AGGGTTTACGACCTCGATGTTGGATCAAGACACCCCAATGG

TGCAACCGCTATTAATG-GTCCGTTTGTTCAACGGTTAA-AGTCTTACGT

GATCTGAGTTCAGACCGGAGTAATCCAGGTCGGTTTCTATCTATTT--AA

TATTTCTCCCAGTACGAAAGGACAAGAGAAAAGGAACCAACTCAAAAT--

AGCGTTCCCAGCATAACAAATGCAATTAA-CTTAATTTAGTAATCTACTA

TCTAT----CTTACCCAAGACAAGGG---TTTGTTAAGATGGCAGAGCCC

GGTAA-TTGCATAAAACTTAAACCTTTATAATCAGAGGTTCAAATCCTCT

TCTTAACA-TACT-------------------------------------

------------------------------------------ATGTTTAT

AGTTAATCTCCTCCTCCTAATTATCCCCGTCCTCCTAGCCATAGCCTTCT

TCACCCTAATTGAACGAAAAATTTTAGGTTATATACAACTCCGCAAGGGC

CCCAACGTTGTTGGCCCACATGGCCTCCTACAACCCTTCGCCGACGCAAT

AAAACTATTTATTAAAGAACCTCTCCGACCCCTAACTTCATCATCACTAC

TTTATACAGTAGCGCCAACTCTAGCCCTTTCAATTGCACTAATTATATGA

ATTCCCATACCACTACCATACCCATTAATTAATATAAATATAGGACTTTT

ATTTATCCTTGCTACATCAAGTCTGGCAGTATATTCAATTTTATGATCAG

GCTGAGCCTCCAACTCAAAATACGCCTTAATCGGAGCCCTACGAGCAGTA

GCCCAAACAATCTCCTATGAAGTTACTCTAGCCATTATTTTATTATCTGT

CCTCCTAATAAATGGGTCATTTACCCTTTCCACCCTTATCACAACTCAAG

AATATCTATGACTAATTATCCCGTCATGACCACTAACCATAATATGATTT

ATCTCAACTCTAGCAGAAACAAACCGAGCCCCCTTCGACCTTACAGAAGG

AGAATCTGAACTAGTCTCAGGCTTTAACGTAGAGTATGCTGCGGGACCAT

TTGCCCTATTCTTTATGGCAGAATATACAAATATCATCATAATAAATGCC

CTAACTACAATTATCTTTTTAGGAGCATTATATGACCCTCATATTCCAGA

AACGTATACGACAAACTTTGCCATTAAAACTCTATTGCTAGCCACACTAT

TTCTATGAGTACGAGCATCCTACCCACGATTTCGATATGATCAACTTATA

CATTTATTATGAAAAAGTTTCCTACCCCTGACACTAGCCCTATGTATATG

ATACATCTCCCTCCCCATCTTAATATCCTGCATCCCACCACAAATATA--

GAAATATGTCTGATAAAAGAATTACTTTGATAGAGTAAATAATAGAGGTT

CA-AATCCTCTTATTTCTAGGATTATAGGTATCGAACCTATCCCTAAGGA

TTCAAAATCCATTGTGCTACCAA-TATACACCACATCCTATAC------A

GTAAGGTCAGCTAAAT-AAGCTATCGGGCCCATACCCCGAAAATGTTGGT

TTATATCCTTCCCGTACTAATCAAACCATCCATCCTCATACTTATCCTAA

TAACAATCTTTTCAGGAACAATACTAACAATAATTAGTTCACACTGACTC

CTAATTTGAATTGGATTAGAAATTAATATATTATCCCTTATTCCGATTCT

AACAAAAAAGATAAGTCCACGATCCACAGAAGCAGCTACTAAATATTTCC

TTACACAAGCCACAGCCTCCATACTATTAATATTTACTATTATCATAAAT

GCCATAAACTCCGGCCAATGAACCACCATTAACACTTACAATAATTTAAC

TTCCTTCACGATCATTATCGCCCTGACAATAAAATTAGGAATAACCCCAT

TTCACTTTTGAGTGCCAGAAGTCACACAAGGAGTTACACTAACAGCAGGA

ATACTGCTCCTAACATGACAAAAACTAGCCCCCATTTCCATTATAATTCA

AATTTATCCCTCAATAAACCCAAACATCCTCTTATTAGTATCCCTACTAT

CAATTCTAGTGGGAGGGTGAGGAGGTCTAAACCAAACACAACTACGAAAA

ATCCTAGCTTACTCATCAATTGCACATATAGGATGAATAGTCTCCATTCT

CATATTCTACCCTTCCCTAACAATTCTAAATCTATTAATCTACTTAATAC

TAACCATTACCGTGTTCACTATATTAAATATTAATACAAACACAACCACT

CTAGCCCTATCAAACTTATGAAACAAAACACCAACAATTACCATAACCAT

TCTAATTTCCTTATTATCCCTAGGAGGTCTACCCCCACTCATAGGCTTCC

TACCAAAATGAGCCATCATCCAAGAACTAACAAAGAACAGCAACATTATT

ATAGCTACAACAATAGCTATCATGGCACTACTCAACTTATACTTCTATAT

ACGATTGATTTACTCCACATCACTAACCATATTCCCATCTTCCAACAACA

TAAAAGTAAAGTGACAACTTCAACCCACAAAACAGACTCTCCTTTTATCA

CCACTAGTAATTTTATCTACCCTAACCCTACCTTTATCACCAGCTTTCTT

AATCAT---AAATT----AGAAATTTAGGTTAAA----TAGACCAAGAGC

CTTCAAAGCCCTAAGCAAGTAGTCATAT---ACTTAATTTCTGCAAA---

---------TAAGGACTGCAAGAATTTATCCTACAT----CAACTGAATG

CAAATCAATTACTTTTA---TTAAGCTAAATCCTCA-----------CTA

GATTGGT---GGGCTCAGACCCCACGAAAAATTTAGTTAACAGCTAAATA

CCCTAATCAACTGGCTTCAATCTACT--TCTCCCGCCGCTCAGAAAAAAA

-----GGCGGGAGAAGCCCCGGCAGAATT---GAAGCTGCTTCTTT----

---------------------------GAATTTGCAATTCAACATGATA-

---TTCACCTCAGAGCTT------GATAGAAAGAGGG-----CTTAACCT

CTGTCTTTAGATTTACAGTCTAATGCTTA--CTCAGCCATTCTACCA---

--CTACCTATGTTCATCAATCGTTGATTCTACTCAACAAACCATAAAGAT

ATTGGAACCCTTTACCTCCTATTCGGAGCTTGAGCAGGCATAGTAGGAAC

AGCCCTTAGTCTCCTCATCCGTGCAGAACTCGGCCAGCCCGGAGCATTAC

TAGGAGATGATCAAATTTATAACGTAATCGTAACAGCCCACGCATTCGTT

ATAATTTTCTTCATAGTAATACCCATCATAATTGGAGGCTTCGGAAACTG

ATTGGTACCTCTTATAATTGGAGCCCCTGATATAGCATTTCCACGAATAA

ATAATATAAGCTTCTGACTTTTACCCCCATCTTTCCTACTTCTCCTCGCT

TCCTCTATAGTAGAAGCAGGTGCAGGAACCGGGTGAACAGTATACCCCCC

TTTAGCTGGAAATTTAGCACACGCAGGAGCATCCGTAGATTTAACTATTT

TCTCTTTACACTTAGCAGGAGTATCCTCAATTTTAGGTGCCATTAACTTT

ATCACAACAGTCATTAATATAAAACCCCCAGCCATATCACAATATCAAAC

TCCTCTGTTCGTATGATCAGTTGTAATTACTGCTGTACTCTTACTACTAT

CCTTACCAGTATTAGCAGCAGGAATCACTATACTCCTAACTGATCGTAAC

CTCAACACAACCTTTTTTGACCCTGCAGGAGGAGGTGATCCTATTTTATA

TCAACATCTATTCTGATTCTTCGGTCATCCTGAAGTATATATTCTAATTC

TTCCAGGCTTTGGCATAATCTCCCATATCGTTACCTATTACTCAGGTAAA

AAAGAACCCTTCGGTTATATAGGAATGGTCTGAGCTATAATATCTATCGG

CTTCTTAGGCTTCATTGTATGAGCACATCACATATTCACTGTCGGAATAG

ACGTAGATACCCGTGCGTACTTTACGTCTGCTACTATAATTATTGCTATT

CCTACCGGAGTAAAAGTTTTCAGCTGATTAGCCACATTACATGGTGGCAG

CATTAAATGGTCACCCGCTATACTATGAGCTCTAGGTTTCATCTTCCTAT

TTACAGTGGGGGGTCTAACAGGGATTGTGCTTGCCAATTCATCACTAGAT

ATTGTGCTCCACGACACATATTACGTAGTAGCCCATTTTCACTATGTCTT

ATCTATAGGAGCAGTTTTTGCTATTATAGGAGGCTTCGTACACTGATTTC

CCCTGTTCTCGGGTTATTCTCTAGATAACACCTGAGCTAAAATTCACTTC

GCAATCATATTCGTAGGCGTAAATATAACTTTTTTCCCGCAACACTTTCT

AGGCCTAGCAGGAATACCTCGACGTTATTCTGACTACCCCGATGCCTACA

CCATGTGAAACACAGTCTCGTCCATCGGCTCTTTTATCTCTCTTACAGCA

GTAATACTAATAATTTTCATAATCTGAGAAGCATTTGCCTCAAAACGAGA

AATTACGATAGTAGAATTAACCCCAACTAACCTAGAATGACTTCATGGCT

GCCCGCCACCCTACCACACATTTGAAGAGCCTACATACGTAAAAGCTTTA

AT----------------CGAAAAAGGAAGGAGTCGAACCCCCTATAATT

GGTTTCAAGCCAACCACATAACCGCTATGACTTTCTCCATTTA-----AA

GATATTAGTAAAAT-AATTGCATAACTTTGTCAAAGTTAACTTATAGGTG

G------AACTCCTATATATCTTA-ATGGCCTGCCCAGTCCAATTAGGAT

TTCAAGACGCTGCTTCTCCTATTATAGAGGAACTTACATATTTCCATGAC

CACACTTTAATAATCGTCTTCCTAATCAGCTCCTTAGTCCTATACATTAT

TTCCCTTATACTCACCACAGAACTTACCCATACAAGTACTATAGATGCTC

AAGAAGTAGAGACAGTATGAACCATTTTACCCGCTGTCATCTTAATTCTA

ATTGCCCTTCCATCACTACGTATTCTGTACATAATAGATGAAATCACCAC

ACCCTCCCTTACCCTAAAAACTATAGGCCATCAATGATACTGAAGCTACG

AATATACAGATTACGAAAATCTATGTTTTGACTCATACATAACGCCTTCA

TCAGACCTAAAGCCTGGAGAACTCCGCCTACTAGAAGTCGACAATCGAGT

CGTTTTACCTACAGAGATATCCATCCGTATACTTATTTCTTCAGAAGATG

TTTTACATTCATGAACCGTACCCTCCTTAGGCGTAAAAACAGACGCCATC

CCAGGACGCCTAAATCAAGCAACCCTAATAACTTCTCGTCCAGGTATCTA

TTATGGTCAATGCTCAGAAATCTGTGGTGCAAACCACAGCTTTATACCAA

TTGTACTTGAGTTAGTCCCACTAAAACACTTTGAAGAGTGATTACTAGCC

ATGCTTTAATAA---------------------------------CACTG

CGAAGCTAT---ATAGCATTAACCTTTTAAGTTAAAGACTGAAAGCCTTG

A--C-CTTTCCACAGTGAGATGCCACAATTAGAAACATCAACGTGATTTA

TCACCATCTTATCAA-TAGTTCTAACCCTATTTATTGTATTCCAACTAAA

AATCTCAAAAC--TTAACTTCCCTTTAAAACCTGAGTTAAAAATTACCAG

TAAATATCAA-CATACTAATCCTTGAGATACAAAATGAACGAAAATTTAT

TCGCCTCTTTCACTACCCCAACCATCATAGGCATTCCTATTGTAATTCTT

ATTATTTTAACTCCCAGTATTTTCTTCCCCTCTTCACCCCACCTTCTCGG

CAACCGACTAATATCTCTTCAACAATGACTTGTCCAACTCGTATT-AAAA

CAATTAATAGCAATCCACAATATCAAAGGGCGAACCTGATCCCTCATACT

AATCTCACTGATTCTATTTATTGGATCTACCAATTTACTAGGCCTGTTAC

CCCACTCATTTACTCCTACCACACAATTATCAATAAATCTAGGAATAGCC

ATTCCCCTATGAGCAGCTACGGTTATTATAGGCTTTCGACACAAAA-CAA

AAAAATCCTTAGCCCATTTCCTCCCACAAGGAACACCCATTCCCCTCATT

CCTATACTAGTAATTATCGAAACCATTAGTCTTTTTATTCAACCTATAGC

ATTAGCCGTACGATTGACAGCTAATATTACTGCAGGCCACCTTCTTATAC

ACTTAATCGGAGGAGCTACCATAGCATTAACCTCCATTAGCCCTACCATT

TCCTCAATCACATTTACTATTTTAATACTCCTTACAGTCCTTGAGTTTGC

CGTTGCCCTCATTCAAGCCTATGTGTTTACCCTCTTAGTAAGCCTTTATT

TACATGACAACGCCTAATGACCCACCAAATCCATGCCTATCATATAGTTA

ATCCCAGCCCCTGACCTCTTACAGGAGCTCTCTCTGCACTTTTAATAACA

TCTGGCCTTGCCCTATGATTTCATTTTAATTCAAGCACACTCCTATTCTT

GGGCCTATTAACTAATCTACTAACAATATATCAATGATGACGAGATATTG

TACGAGAAGGCACATTCCAAGGCCACCATACCCCCGCTGTCCAAAAAGGG

CTCCGATACGGTATAATTCTTTTTATCATCTCAGAAATTTTCTTCTTTAC

AGGTTTCTTCTGAGCCTTCTATCATTCAAGCCTGGCACCTACTCCTGAAC

TAGGCGGCTACTGACCCCCAGCAGGCATTAAACCCCTCAATCCACTAGAA

GTTCCTTTACTCAATACAACTGTACTCCTAGCCTCAGGTGTATCCATCAC

CTGAGCTCACCATAGTCTAATAGAAGGTGACCGCACAAGCATGCTACAAT

CCTTACTCATCACCATTACCCTAGGAGTCTATTTTACCCTACTCCAGGCA

TCAGAATACCTCGAAACATCCTTTACAATCTCAGATGGCGTATATGGCTC

AACATTTTTCATAGCAACAGGTTTCCATGGCTTACATGTCATCATCGGAT

CTACCTTCCTAACCATCTGCCTTCTCCGCCAACTAAAATTTCACTTCACT

TCCAATCACCATTTCGGTTTTGAAGCCGCAGCCTGATACTGACATTTCGT

TGACGTAGTCTGACTTTTCCTTTATGTATCAATCTACTGATGAGGATCAT

ATTCT-TTTAGTATC-ACTCAGTACAACTGACTTCCAATCAGTTAGCTTC

GGCACTAACCC-GAAAAGAATAATTAATCTCCCATTGACTCTTTTAACCG

ATATTCTACTAGTATTAGTACTCGTATTTATCGCATTTTGATTACCTCAA

CTAAACATTTACACAGAAAAATATAACCCATATGAATGCGGCTTCGACCC

TATAGGATCTGCTCGCCTGCCATTTTCCATAAAATTCTTTCTAGTAGCAA

TTACATTTTTATTATTTGATCTAGAAATTGCTCTCTTACTCCCACTACCA

TGAGCATCCCAATCAAACAATCTAAAACTCACGACAATTGTAGCCCTTAC

ATTAATTACAATTCTAGCTCTAAGCCTCGCCTATGAATGACTTCAAAAAG

GATTAGAGTGAGAAGAGTAATC---TGGTAATTAGTTTCAACTAAAA-CA

AATGATTTCGACTCATTAGATTATGATTAATT--CATAATTACCAAC--A

TGCCCTCAATCTCCACAAACATGACTTTAGCCTTTATCACTGCCCTATTA

GGGATATTAATATTCCGATCTCACCTAATATCCTCCCTCCTATGCTTAGA

AGGAATAATATTATCTATATTTATTTTAAGTACCCTTCTAATTCTAAATA

TACAATTTACAATCACCTTTATTATACCCATCCTTCTTTTAGTCTTTGCA

GCCTGCGAAGCTGCCATCGGCCTAGCCCTCCTAGTAACAGTGTCTAATAC

CTATGGCCTAGACCATATTCAAAATCTTAACCTTCTCCAATGTTAAAAAT

TATTACCCCCACAATTATACTATTCCCAATAATTTGATACTCCAATAATA

ACAAAATCTGAATTAACACAACCCTATACAGCCTGATAATTAGTATCATA

ATATTATTACTACTTAATCAAACTGACAACAACAGTAATAATTTCTCACT

AACTTTTTTCTCCGACTCACTATCCTCTCCTCTCCTAATACTAACAGTAT

GGTTACTCCCCTTAATAATTATAGCTAGCCAACATCATCTTATAAAAGAG

ACCTGAATACGAAAAAAACTATATCTATCTATGTTAATCTTTCTACAAGT

GTTTCTAATTATAACCTTTACAGCCACCGAACTTATTCTATTTTATATCC

TCTTTGAAGCTACACTAATCCCAACCCTTATTATTATTACCCGGTGAGGT

AACCAAACAGAACGACTAAACGCGGGCCTATACTTTCTATTTTATACCCT

TATCGGGTCTCTACCATTACTCGTAGCACTAATTTATACCCAAAATTTCC

TAGGTTCCCTAAACATATTAACAATTACTCTTTATAATCAAGAATTATCC

AATTC----------ATGATCTAATAGTTTTTTATGAATAGCATGTATTA

TAGCATTTATAGTCAAAATACCACTATATGGGCTTCACTTATGATTACCC

AAAGCCCATGTAGAAGCACCCATCGCCGGCTCCATAGTACTTGCAGCCGT

ACTTTTAAAATTAGGTGGATATGGAATGATACGTATTACCATAATCCTAG

ACCCCATAACAAAATTCTTGGCATACCCTCTCCTTATACTATGCCTATGA

GGAATAATTATGACCAGTTCAATCTGCCTACGACAAACAGACCTAAAATC

ACTTATCGCTTATTCATCAGTAAGTCATATGGCACTAGTTACTATAGCAA

TCCTAATTCAAACACCATGAAGCTTTATAGGAGCAACAGCCCTTATAATC

GCACACGGCCTTACATCATCCATATTATTTTGCCTTGCAAACTCGAATTA

CGAACGTATCCACAGCCGTACAATACTCCTAGCACGAGGACTTCAATCCT

TTCTCCCCTTAATAGCTACCTGATGACTTCTAGCCAGCTTAACCAATCTA

GCTCTACCTCCCTTCATTAATCTAATTGGTGAACTCCTCGTAATTATAGC

TTCCTTCTCATGATCGAATATCACAATTATCCTAACGGGCCTAAATATAT

TTATTACAGCCCTTTATTCCCTTTATATATTAACCGTTACACAACGAGGC

AAATCCACCTACCACATACATAACCTTAACCCCTCATTCACACGAGAAAA

TACCCTAATATCTATACATATATTTCCTATTGCCCTACTAACCCTCAATC

CAAAACTTATTTTAGGTCCTACGTACTGTAAATATAGTTTAAACAAAACT

CTAGATTGTGAATCTAGCAATAGAGGCTTA-AAATCCTCTTGTCTACCGA

GAGAGTGAC--GTA-GAATTGCTAACTC-TGCTCCCCGTATATAAAAGTA

TGGCTCCCTCAACTTTTAAACTTTTAAACTTTTAAACTTTTAAACTTTTA

AACTTTTAAACTTTTAAACTTTTAAACTTTTAAAGGATGGTAGTT-ATCC

ATTGGCCTTAGGAGCCAAAAATAATTGGTGCAACTCCAAATAAAAGTAAT

AATAA---TGCACACCTCCATTTTTATATTAGCCCTAACCCCCTTAATCT

TTCCGATTATTATTACCCTTATTAGCCCCAATAAAAATAATATATACCCC

AACTATGTAAAAACAACTATGATATTTACCTTTACTATTAGTCTCATCCC

CACAACCATATATACTTTCCTAGGTCAAGATACAATTATATCAACCTGAC

ATTGAATAACCATCCAATCACTAGAAATTACACTAAGTTTTAAATTGGAC

TATTACTCCGTAATATTTACCCCAATTGCACTATTTATTACTTGGTGCAT

TATAGAATTCTCACTATGATACATAGACTCAGACCCAAACATTAACCAAT

TCTTCAAATATCTTCTCATCTTCCTTATTACCATGCTAATTTTAGTTACC

GCTAACAACCTCTTCCAGCTCTTTATTGGGTGAGAAGGTGTAGGAATTAT

ATCATTTCTACTAATCGGCTGATGATACGCTCGAACAGACGCTAACACAG

CAGCCATTCAAGCAATTCTGTATAACCGCATTGGTGATATTGGTTTCATT

CTAGCCATAATATGGTTTCTCCTCCATTATAACTCATGAGACTTACAACA

AATATTTATCCTAGATCCCAACCCCGATCTACTTCCATTAGTGGGTCTAC

TATTAGCAGCAACAGGAAAATCAGCCCAACTCGGCCTCCATCCCTGATTA

CCCTCGGCTATAGAAGGCCCAACTCCAGTATCAGCCCTACTTCACTCCAG

TACTATAGTAGTAGCTGGGGTTTTCTTACTTATTCGCTTCCACCCACCAA

TA-GAAAATAATACAACAATTCAAAGTCTTACACTATGCCTAGGAGCTAT

TACTACCATATTCATAGCAATCTGCGCCCTAACACAAAATGACATTAAAA

AAATTGTAGCCTTCTCTACCTCAAGTCAACTGGGACTTATAATAGTTACT

ATTGGTATTAATCAACCGCACCTAGCATTTCTACATATCTGTACTCATGC

CTTTTTCAAGGCTATACTATTTATCTGCTCTGGGTCTATAATTCATAACC

TAAATAATGAACAAGACATCCGAAAAATAGGAGGACTATTTAAAACAATA

CCCCTCACCTCAACTTCCCTGATAATCGGTAGCCTAGCACTCACAGGCAT

ACCTTTTCTTACAGGTTATTACTCCAAAGACCTCATCATCGAAACCGCAA

ACACATCATACACCAACGCCTGGGCCCTGTGTATTACTCTTATCGCTACC

TCTATAACAAGCGCCTACAGCACCCGAACTATTATCCTCACACTAACAGG

ATCACCTCGTTTTTCAACTTCCGTATATATTAATGAGAACAACCCAACCC

TACTAAACCCAATAAAACGCCTAGCAGCAGGTAGTCTACTCGCAGGATTT

TTCATCGTCAACAACATCTCTCCGACTACAGTTCCTCAATTAACAATACC

TTATCACCTGAAACTCCTAGCCTTATGCGTAACCACCCTAGGCTTCTTAA

CAGCCCTAGATCTGACTCTCATAACTAACAGTCTCAAAATAAATACCCCA

TCGCACATATTC-AAATTCTCCAATATACTAGGATATTTTACCATTACAA

TTCACCGAACAGTTCCCTACCAAAACCTAACCATAAGCCAAAACCTAGCC

TTCCTATTACTAGACTTACTCTGACTAGAGAAATCAATACCTAAAACAAT

TTCACACACCCATATTATTACGGCCATCACCTCAA---CCACCCAAAAAG

GCATAATCAAGCTATATTTCCTCTCTTTTCTTATTTCCCTCACACTAATC

CCACTTTTAATTATATAATCTCCCACTTTTAATTATATAATCTATTACCC

CGAGTAATTTCAATAACAATATAAACACCAACAAATAATGTTCAACCAAC

AACTACGACCAACCAACGCCCATAATCATACAAAGCACCCGCACCAATAG

AATCCTCACGAATCAACCCCGACCCCTCCCCCTCAAAAATCACCCAACTC

CCTATATTATCCAAATTAATTATCACTACCAA---------CTCATTATA

ATCTATAACCCACAGAACTAAATATACCTCCATTGCCAATCCAACCAAAA

AACTCCCCAAAACCTCAAATCCTGAAACCCATGCTTCAGGATATTCTTCA

ATAGCCATCGCAGTAGTATAACCAAAGACAACCATTATACCCCCCAGATA

AATCAAAAACATTATTAAACCTATATAAGTACCCCCATAACTTAAAATAA

TAGCACAACCAATCACACCACTAACAACCAATGCTAAACCCCCATAAATA

GGAGAAGGCTTAGAAGAAAAGCCCACAAAACCCATAACTAATAATACGCT

TAATAAAAATAAAATATACGACATTGT----TTCCACATGGACTCTA-AC

CATGATTAATGATATGAAAAACCATCGTTGTAT-TTCAACTATAAAAACA

CT-----AATGATCCCCATACGCAAATCTAATCCAATTATAAAATTAATT

AATCACTCCCTCATTGATCTACCAACCCCATCAAACATCTCAGCATGATG

AAACTTTGGTTCCCTTTTAGCAATCTGCTTAATTTTACAAATCATCACAG

GTCTATTCCTAGCAATACATTACTCACCTAGCACCTCCTCAGCCTTCTCC

TCAATCGCCCATATCACTCGAGACGTAAACTACGGCTGAATTATCCGCTA

CCTTCATGCCAATGGTGCCTCCATATTCTTTATCTGCCTATTCCTACATG

TGGGTCGAGGATTATATTATGGCTCATTCCTTCTTCTTGAAACTTGAAAC

ATTGGCATTGCACTATTACTTATAGTTATAGCAACGGCCTTTATAGGCTA

TGTACTCCCATGGGGGCAAATATCATTTTGAGGTGCTACAGTAATTACAA

ATTTATTATCCGCAATCCCATACATCGGAACAAACCTCGTTCAATGAGTA

TGAGGTGGGTATTCTATCGATAACCCAACCCTTACCCGATTCTTCACTCT

CCACTTTACCCTGCCTTTCATTATCACAGCCTTTACAGTTCTACATCTAC

TTTTCCTACACGAAACAGGATCTAATAATCCATGCGGAATTCCCTCAAAC

TCCGACAAAATCCCCTTCCACCCCTACTACACAATCAAAGATATGCTAGG

CCTAGTCCTCCTTATTCTTCTCCTAATAACTCTAGTATTATTTTCACCTG

ACCTTTTAGGCGACCCAGACAACTATACACCAGCTAATCCACTAAACACC

CCACCACACATCAAACCAGAATGGTACTTCTTATTCGCATATGCAATCCT

ACGATCTGTACCTAACAAATTGGGAGGCGTACTAGCACTTCTTATATCCA

TTCTTATCCTAATAATTATCCCCATACTTCACAAATCCAAACAACAGAGC

ATAATATTCCGCCCATTCAGCCAATTTACACTATGATTACTAATCACAGT

TCTATTAACCCTAACCTGAATTGGAAGTCAACCAGTAAACCAACCCTTTA

TTATAATCGGACAGGTAGCATCTATAATATATTTCACCACAATTCTGATC

TTAATACCACTAGCCTCTATAATCGAAAACAATCTCCTCAAATGAA---C

CTGCCCTTGTAGTATA--AACCAATACTCTGGTCTTGTAAACCAGAAATG

GAGATC----ACTCTCCCTAGGACAA--CCTCAAGGAAGAAG-CATTAGC

CCCACCTTCAACACCCAAAGCTGAAATTCT--ACTTAAACTACTCCTTG-

--------------------------------------------------

--------------------------------------------------

--------------------------------------------------

--------------------------------------------------

--------------------------------------------------

--------------------------------------------------

--------------------------------------------------

--------------------------------------------------

--------------------------------------------------

--------------------------------------------------

--------------------------------------------------

--------------------------------------------------

--------------------------------------------------

--------------------------------------------------

--------------------------------------------------

--------------------------------------------------

--------------------------------------------------

--------------------------------------------------

--------------------------------------------------

--------------------------------------------------

--------------------------------------------------

--------------------------------------------------

--------------------------------------------------

------

>Urocitellus_richardsonii Urocitellus richardsonii mitochondrion, complete genome.

GTTAATGTAGCTTAA--TC----AAATAAAGCAAAGCACTGAAAATGCTT

AGATGGGTATTTTA--ACCCCATGAACATATA--GGTTTGGTCCTGGCCT

TTTTATTAGCTGTTAGCTAACTTATACATGCAAGCATCCCCGCCCCAGTG

AGAATGCCCT-CTATATCTATAAAT---CGATCAAAAGGTGCAGGCATCA

AGTTCACT-TACTCTAGTAGCTCACAACGCCTTGC-TCCACCACACCCCC

ACGGGATACAGCAGTAATTAGAATTAAGCCTATAAACGAAAGTTTGACTA

AGTTAAGCTA--AATT-AGGGTTGGTAAATTTCGTGCCAGCCACCGCGGT

CATACGATTAACCCTAGTTAATGAAGT-AC-GGCGTAAAGCGTGATTAAG

AGA------CTAGT-TAGATAAGATTAAAGTAATACTAAACCGTAAAAAG

TC-TTGGTACTAATGAAAATCAAATACGAAAGTAATCTTA---AACTTTC

TGAATTCACGATAGCTAAGACCCAAACTGGGATTAGATACCCCACTATGC

TTAGCCCTAAACATAAATA-TTCAAC-AAACAAGAATATTCGCCAGAGAA

CTACTAGCAATAGCCTAAAACTCAAAGGACTTGGCGGTGCTTTACACCCC

TCTAGAGGAGCCTGTTCTATAATCGATAAACCCCGATACACCTCACCACC

TTTAGCAAATATCAGCCTATATACCGCCATCTGCAGCAAACCCTAA-AAA

GGCCTCATAGTAAGCAAGAAAATTCT--ACATTAGTACGTTA-GGTCAAG

GTGTAGCCTATAAGGTGGG-AAGAAATGGGCTACATTTTCTACTTTTCTA

GAATA------AATATCCACGATAGCTTTCATGAAACTTAA---AGCATA

AGGCGGATTTAGTAGTAAGTC-AAGAATAGAGAGCTTGACTGAATAG-GG

CAATAAAGCACGCACACACCGCCCGTCACCCTCTTCAAATATACCT----

ATACAAAACTATAAATAATTTACCTAATCAAG--T--ATATA--AGAAGA

GATAAGTCGTAACAAGGTAAACATACTGGAAAGTGTGTTTGGAAGAATCA

AAATGTAGCTTA--TTAACTAAAGCACCCGGCTTACACCCGAGAGATTTC

ATTTTATTATGGACATTTTGA-ACC-AATGCT-AGCCCAACTTTTC----

-TCCCATCCAAATACTATTCACCCAACAAATAAAACATTCACC-TAGAA-

--TAAA-GTATAGGAGATAGAAATTTA--TACCA--GGAGCTATAGAAAA

AGTACCGTAAGGGAAAGA-TGAAAGAATAAATTTATAGTATTAAAAAGCA

AAGACTAACTCTTTTACCTTTTGCATAA-TGATTTAACCAGAAAATGCTT

GACAAAAAG-AATTTAAGCCAAACACCCCGAAACCAGACGAGCTACTCAT

AAGCAGCT--AATAAGAGCTAATCCGTCTATGTTGCAAAATAGTGGAGCG

ACTTATAAGTAGAGGTGAAAAGCCTACCGAGCCTGGTGATAGCTGGTTGT

CCA-GACTAGAATTTTAGTTCTACTTTAAATTTACCTAAAGCATA-AAT-

AG-GCTGAATGTAAATTTAAATGTTATTCTAAAGAGGGACAGCTCTTTAG

AGTTAAGGAATAAACCTTATTTAGAGAGTAA-----ATCAAACAATC--T

CCATAGTTGACTTAAAAGCAGCCATCAATTAAAAAAGCGTTAAAGCTTAA

CCTAATCA-T---AAAAACTTAATACCTAACTCT---TTCAATAATCT--

CCTAAATCAACA-CTGGACTAATCTATTT-ATAAT-AGAAGAAATTATGT

TAAAATAAGTAACAAGAA-AATAT-T-CTCCCTGCATAAGCTTATATCAG

ATCGGA-TAATTCACTGATAGTTAACAACCTCATA-----ATATTAAACA

AAACCATAAATCATTATTATTTACATTGTTAACCCAACACTGGCATGCA-

--TTAAGGGAAAGATTAAACAAAGTAAAAGGAACTCGGCAAACATTAACC

TCGCCTGTTTACCAAAAACATCACCTCTAGCATAAATAGTATTAGAGGCA

CTGCCTGCCCAGTGACAATACG----TTCAACGGCCGCGGTATCCTGACC

GTGCAAAGGTAGCATAATCACTTGTTCTTTAAATAAGGACTAGCATGAAT

GGCTTAACGAAGGTTTGACTGTCTCTTACTTTTAATCAGTGAAATTGACC

TTCCCGTGAAGAGGCGGGAATTTTATAATAAGACGAGAAGACCCTATGGA

GCTTTAATTTAAC-AGTCTCACAACCTTAATAATATCTT----------A

GGAATTAA-AATTATTGTTCA-TAGACTAGAAATTTTGGTTGGGGTGACC

TCGGAGTACAAATCAACCTCCGAATGATA-ATAATCT-AGACTCTACATG

TCCAAATTACAATTC----ATAAATTGACCCAG-G---ATACTGATCAAC

GGAACAAGTTACCCTAGGGATAACAGCGCAATCCTACTCAAGAGTCCATA

TCGACAGTTAGGGTTTACGACCTCGATGTTGGATCAGGACATCCAAATGG

TGTAACCGCTATTAATG-GTTCGTTTGTTCAACGATTAA-AGTCCTACGT

GATCTGAGTTCAGACCGGAGAAATCCAGGTCGGTTTCTATCTATTTCTA-

TATTTCTCCCAGTACGAAAGGACAAGAGAAATAAGGCCAATTAAACAC-T

TATGCCTTAAGTTTAATGGATGAG-ACAATCTTAATCCAGTAGAATATTT

TAAAC--AACCTGCCCTAGAAAAGGG--CTT-GTTAAGATGGCAGAGCCT

GGTAA-TTGCGTAAGACTTAAAACTTTATT-TCAGAGGTTCAACTCCTCT

TCTTAACA-CTA--------------------------------------

------------------------------------------ATGTTTAT

AATTAATCTTCTACTTTTAATTATCCCAATCCTAGTAGCCATAGCTTTTC

TTACCCTAATCGAACGAAAAATATTAGGATACATACAACTCCGCAAAGGC

CCCAATGTTGTTGGACCTTACGGCCTACTTCAACCATTTGCTGACGCAAT

AAAGCTATTCATTAAAGAGCCCATAAAACCCCTAACATCGTCAATTATAT

TATTTATTATTGCTCCAACCCTAGCCCTAACACTAGCTTTTACCATATGA

ATCCCCCTACCCATACCAATACCCCTCATCAACATAAACATAGGAGTGCT

ATTTATCTTAGCCACATCAAGTCTAGCCGTATATGCAATTCTATGATCTG

GATGAGCATCCAACTCTAAATATGCTTTGATTGGAGCCCTACGAGCCGTA

GCACAGACCATCTCATATGAAGTAACATTAGCAATCATCCTTCTCTCAGT

ACTTCTAATAAATGGATCATTTACCCTATCCACACTTATCACCACCCAAC

AATTTACATGGCTGCTACTTCCAACATGACCTCTAGCAATAATATGATTT

ATTTCAACATTAGCAGAAACTAATCGAGCTCCATTTGATCTAACAGAAGG

GGAATCAGAACTTGTATCAGGATTTAATGTTGAGTACGCAGCCGGTCCAT

TCGCCTTATTCTTTATAGCCGAATATACTAACATTATCATAATAAACGCA

TTAACAGTAACTCTTTTTATAGGAGCACTACTAAATCCCATCTTTCCCGA

AACTTTTACATTAAACTTCACCTTAAAAACACTCATCTTAACTTCCACTT

TCCTATGAATCCGGGCATCCTATCCTCGATTCCGTTACGACCAACTCATA

CATCTTTTATGAAAAAACTTCTTACCCCTAACCCTAGCCTTATGTATATG

ACATATCTCTCTTCCAATTATAACTGCATGCGTACCACCCCAAATCTAA-

GAAATATGTCTGATAAAAGAGTTACTTTGATAGAGTAAATTATAGAGGTT

TA-AATCCTCTTATTTCTAGAACTATAGGAATTGAACCTAATCCTAAGAA

TTCAAAATTCTTCGTGCTACC-TTT-TACACCACGTCCTAAAT--A----

GTAAGGTCAGCTAATT-AAGCTATCGGGCCCATACCCCGAAAATGTTGGT

TTATATCCTTCCCGTACTAATTAATCCCTTAACTTCCTCCGCAGTCTACT

TTACCCTCTTTTCTGGAACTATAATTACACTTTTTAGCTCACATTGACTC

CTAACTTGAGTAGGTCTAGAAATAAGCATATTAGCTATTACCCCTATTCT

AATCAATAAAGGAAACCCCCGATCTACAGAAGCTGCATGCAAATACTTTC

TCATTCAAGCCACCGCATCAATAATCTTAATAATAGGCACAATAATTAAC

TTTATAGACTCAGGCCAATGAACCCTATCTAACTCATATAATCAAATTTC

ATCATTTATATTTACAATCGCACTCTCAATAAAAATAGGACTAGCTCCCT

TTCACCTATGAGTCCCAGAAGTCACCCAAGGAATTCCACTTATATCAGGT

CTAATCATGTTAACATGACAAAAAATTGCCCCAATCTCTATCGTTTATCA

AATCGCACCTTCTATAAACCCTACCCTTATGTTACTTTTAGGAGTCCTAT

CAATCATACTAGGAGGTTGAGGAGGACTTAACCAAACCCAACTACGAAAA

ATCCTAGCGTATTCATCAATTGCCCACATAGGATGAATAATAGCAATCGT

TACATATAATCCAACCTTAACAATATTTAACCTAATTATCTACATCATTC

TTACCATCAACATATTTATACTTCTTCTTTTCCATAAAAAAACTACTACC

CTCTCCCTATCTAATTTATGAAATAAGTTCCCTCTTCTAACACCCACAAT

TCTAATTGTACTAATATCGCTAGGAGGATTACCCCCTCTAACAGGGTTCA

CACCAAAATGAATTATCCTTAAAGAACTTATCTCAAATAACAACATTATT

TTCTCTACACTAATAGCGATACTCGCACTCCTAAACTTATATTTCTATAC

ACGACTTATCTACTCAACATCTCTAACTTTATTTCCATCATTCAACAATA

CCAAAATAAAATGACAATTCGAGAACACAAAGCTTATACCCTTACTACCC

ACCCTAATCATTACTTCTACCCTCTCCCTCCCACTAATACCCCTC-TTCT

CACTCCT--GAACT----AGGAATTTAGGTTAAT--T-CAGACCAAGGAC

CTTCAAAGTCCTAAGCAAGTA-CCTAAT---ACTTAATTCCTGC------

---A----TTAAGGACTGCAAGACTTTATCTTACAT----CAATTGAATG

CAAACCAATCACTTTAA--TT-AAGCTAAGCCCTTC-ACT------CCTA

GACTGATG--GGATTTAAA-CCCAT-AAGATCTTAGTTAACAGCTAAACG

CCTTACTCAACTGGCTTCAATCTA-CT-TCTCCCGCCGTTAAGGGAAAAA

-----GGCGGGAGAAGCCCCGGCAGAG-T-TGA-AGCTGCTCCTTT--GA

-----------------------------ATTTGCAATTCAATA--TGAA

TA-TTCACCTCGGGACT-T-----GGTAAAAAGAGGG----TT-CAACCT

CTGTCTTTAGATTTACAGTCTAATGCTTA--CTCAGCCATTTTACC----

ACCTACTTATGTTCATCAACCGTTGATTCTTCTCAACAAATCATAAAGAT

ATCGGTACACTCTACCTTCTATTCGGTGCTTGAGCTGGAATAGTAGGAAC

TGCGCTTAGTCTACTAATCCGAGCTGAACTAGGTCAACCCGGAGCCCTAT

TAGGTGATGATCAAATTTACAATGTTATTGTCACCGCTCATGCATTTGTT

ATAATTTTCTTTATAGTTATACCAATTATGATTGGTGGATTTGGAAACTG

ACTAGTCCCCTTAATAATTGGAGCTCCTGACATAGCATTCCCACGTATAA

ATAATATAAGCTTCTGACTTCTACCCCCTTCTTTCCTTCTCTTACTCGCT

TCTTCTATAGTTGAAGCAGGTGCAGGAACTGGTTGAACTGTTTATCCTCC

ATTAGCCGGAAACCTTGCCCATGCAGGGGCTTCAGTAGACTTAACCATTT

TCTCTCTTCACTTAGCAGGAGTTTCATCAATTCTAGGTGCAATTAACTTT

ATTACAACTATTATCAACATAAAACCACCTGCTATATCTCAATATCAAAC

TCCTTTATTTGTATGATCCGTATTAATTACAGCAGTACTATTACTCCTCT

CCCTCCCAGTTCTTGCAGCAGGAATTACTATGCTGCTAACAGACCGTAAT

CTTAATACCACATTTTTTGATCCTGCCGGAGGTGGAGATCCAATTCTCTA

TCAACACTTATTCTGATTTTTTGGACACCCTGAAGTTTACATTCTTATCC

TACCGGGATTTGGTATAATTTCTCATATCGTAACATATTACTCAGGAAAA

AAGGAACCATTCGGTTATATAGGTATAGTATGAGCTATAATATCTATTGG

TTTCCTTGGATTTATCGTATGGGCCCATCATATATTTACCGTTGGAATAG

ATGTTGACACTCGAGCCTACTTTACATCTGCAACCATAATCATTGCTATT

CCTACAGGAGTAAAAGTTTTTAGTTGATTAGCAACTCTACACGGAGGAAA

TATCAAATGATCGCCAGCAATACTGTGAGCACTAGGCTTTATTTTCCTAT

TCACTGTAGGAGGCCTTACAGGAATTGTCTTAGCCAATTCTTCACTAGAC

ATTGTTTTACACGATACATACTATGTCGTAGCCCACTTCCACTATGTGTT

ATCGATAGGAGCTGTGTTTGCTATTATAGGAGGGTTCATTCACTGATTCC

CCCTTTTCTCCGGTTACACACTAAATGACCTTTGAGCTAAAATTCATTTT

ACTGTAATATTTGTCGGAGTAAATTTAACTTTCTTCCCTCAACATTTCTT

AGGATTATCAGGTATGCCACGCCGATACTCTGATTACCCAGATGCATACA

CAGCATGAAATACTGTTTCCTCAATAGGTTCATTCATTTCTCTTACAGCT

GTTATAATTATAATCTTTATAATTTGGGAAGCATTTGCATCAAAACGAGA

AGTTCTCACCGTAGAACTAACACCAACTAATTTAGAGTGACTACACGGGT

GTCCTCCACCCTATCACACATTTGAAGAACCCACTTACATCAAGGCCTAG

AT--C--------------AAGAAAGGAAAGAATCGAACTTTCTAAAACT

AGTTTCAAGCCAGCCTCATAACCATTATGA-CTTTCT---TTAT----GA

GATATTAGTAAAAT-AATTACATAACTTTGTCAAAGTTAATTTATAGGTT

A------AACTCCTATATATCTC-TATGGCATACCCCTTCGAATTAGGAT

TTCAAGACGCCACATCTCCTATTATAGAAGAACTTCTACACTTTCATGAC

CATACTCTTATAATTGTTTTCCTAATTAGCTCCCTAGTCCTTTACATCAT

TTCATTAATATTAACCACAAAATTAACTCATACAAGCACCATAGACGCTC

AAGAAGTAGAAACCATTTGAACCATTCTCCCCGCTATTATCCTTATTCTA

ATTGCTCTTCCCTCCCTACGTATTCTATACATAATAGATGAAATTAATGA

CCCAACCCTAACAGTAAAAACGATAGGTCACCAATGATATTGAAGTTATG

AATACACGGATTATGAGGACCTAAATTTTGATTCCTATATGATTCCAACT

TCAGATCTAGCCCCAGGAGACCTACGACTTCTTGAAGTCGACAATCGAGT

TGTTCTTCCAATGGAAATACCTGTACGAATGCTAATCTCATCTGAAGATG

TTCTTCACTCCTGAGCGGTCCCATCTCTTGGACTAAAAACAGATGCCATC

CCAGGCCGACTTAATCAAGCTACACTTACATCAACACGACCAGGACTTTA

TTATGGTCAATGCTCTGAAATTTGTGGGTCAAACCATAGCTTCATACCAA

TCGTTCTTGAATTAGTTCCGCTAAAGCATTTTGAAAACTGATCCTCATCA

ATACTATAA--------------------------------ATT-CATTA

TGAAGCTA--AAATAGCATCAACCTTTTAAGTTGAAGACTAGGAGT-T-A

A-AT-CTCCTCATAATGAAATGCCCCAACTAGATACATCCACATGATTTA

TTACAATTCTATCAA-TAATCCTAGCTCTTTTCTTTATGTTTCAACTTAA

AATCTCAAATCA--CTCTTACCCATCTAATCCCTCCCCTAAAGATACTAA

-ATTAATTGAGCATAAAACCCCTTGAGAAGAAAAATGAACGAAAATCTAT

TTGCCTCTTTCATTACCCCTACATTAATAGGTCTTCCTATTGTCCTTTTT

ATCATTGTATTCCCCAATTTACTTTTTCCTTCACCTACCCGATTAGTAAA

CAACCGCTTAGTGTCATTCCAACAATGACTAATTCAACTTGTACT-AAAA

CAAATAATGGCAATGCACAACCCAAAAGGACGTACCTGATCCCTAATATT

AATCTCACTAATCATATTCATTGGCTCAACTAATCTTCTAGGACTATTAC

CTCACTCTTTTACACCAACAACCCAACTATCAATAAATTTAGGAATAGCT

ATCCCTTTATGAGCAGGAGCAGTAATTACTGGATTTCGTCATAAGA-CTA

AAGCATCATTAGCCCACTTTCTTCCACAAGGAACCCCAATTCCTCTTATC

CCTATACTAATTATTATCGAGACAATTAGCCTCTTTATCCAGCCTATAGC

GCTAGCTGTACGATTAACAGCCAACATTACAGCCGGCCATCTTCTCATAC

ATTTAATCGGAGGAGCAACTCTTGTATTAATATCTATTAGCCCTCCTACA

GCCATCATTACTTTCATTATTCTTGTACTACTGACAATGCTCGAATTCGC

AGTTGCACTAATTCAAGCTTACGTTTTCACTCTCCTAGTAAGCCTATATC

TACATGATAATACTTAATGACCCACCAAACCCACGCCTACCATATAGTTA

ATCCTAGCCCTTGACCCTTAACAGGGGCCCTCTCCGCCTTACTCCTAACC

TCCGGCCTAGTAATATGATTCCATTTCAATTCCTCCTTCCTACTCACACT

AGGCCTAATAGCCAATACTCTAACAATATATCAATGATGACGAGACATTG

TACGAGAAGGTACATTTCAAGGCCACCATACATCAATTGTCCAAAAAGGC

CTGCGATATGGTATAGTACTATTTATTATTTCAGAAGTATTCTTCTTTGC

CGGATTTTTCTGAGCATTCTATCACTCTAGTTTAGCTCCAACTCCCGAAC

TTGGCAGCTGCTGACCTCCAGTAGGAATTAATCCACTCAACCCCTTAGAA

GTACCACTATTAAATACCTCTGTTCTTTTAGCTTCAGGGGTTTCAATTAC

TTGAGCTCACCATAGCCTAATAGAAGGGGACCGAAAACATATAGTCCAAG

CACTATCTATTACAATTGCTCTGGGACTTTATTTTACTCTCCTTCAAGCT

TCTGAATACCTAGAGACTTCTTTTACAATCTCAGATGGTGTATATGGTTC

AACATTCTTTATGGCCACAGGCTTCCATGGTCTCCACGTTATAATTGGAT

CAACCTTCCTTCTAGTATGCCTCATTCGTCAACTAAATTTCCACTTTACA

TCAAAACACCACTTTGGATTCGAAGCAGCCGCATGATACTGACATTTTGT

AGATGTAGTATGACTCTTCCTTTACGTGTCTATTTATTGATGAGGCTCAT

ATTCT-CTTAGTATCAA-TTAGTACAATTGACTTCCAATCAATTAGTTCT

GGAACTAATCCAGAAGAGAATAATTAATCTTATATTATCCCTCATTGTAA

ATTCTTCCATTGCTCTTCTACTAATCTCCGTAGCATTTTGATTACCCCAA

CTAAACGTATACGCTGAAAAGGCAAGCCCTTACGAATGCGGATTTGACCC

TATAGGATCTGCTCGCCTACCATTCTCAATAAAATTTTTTCTTGTTGCAA

TTACATTTCTCTTATTTGACTTAGAAATTGCTCTTCTTCTCCCCCTTCCC

TGAGCCTCTCAAACAAACAACCTCAGCCTTATATTAACTATAGCCTTGCT

CTTAATCTTAATTCTTACTCTCGGATTGGCCTACGAATGAATTCAAAAAG

GCTTAGAATGAATTGAATA------TGATAATTAGTTTAAAATAAAA-CA

AGTGATTTCGACTCACTAAATTATGGGCTA-C--CATAATTATCAA--AA

TGCCTATTATTATTCTCAACACTATTTTAGCCTACTCTACATCTCTATTA

GGGATATTTATTTACCGATCTCACCTAATATCATCACTTCTGTGCTTAGA

AGGGATAATATTATCAATATTTGTCTTATGCTCCCTTTTAATTATAAATT

TTCACTTCTCCTTATCATTTATAATCCCCATCACTTTACTAGTATTTGCT

GCATGTGAAGCAGCTGTAGGCCTAGCCCTTCTCGTAATAGTATCTAATAC

ATATGGCCTAGACTATGTCCAAAACCTAAATATTCTTCAATGTTAAAAAT

TATTATCCCCACAATTCTACTTGCTCCCCTTGTATGGCTCTCAAAGCCTT

CCATAATCTGAATTAATCCTTCAATTCACAGTCTGATAATTAGCCTAATT

GTTCTTTCTACATTAAATCGTCCTATAAATATAGATCTGATTTTCTCACT

AGCTTTCTTCACTGATCCTTTATCCTCTCCTCTACTAATTTTAACAGCGT

GACTCCTACCTCTCATAATTATAGCAAGCCAAAGCCATTTAACCCACGAA

CCATTAATCCGAAAAAAACTATACATTCTTATATTAATCTCACTGCAATC

CTTTTTGATTATAACTTTCTCTGCCACTGAACTAATTATATTCTATATCC

TATTTGAAGCTACCCTAATCCCTACGCTAATTATTATCACTCGATGAGGA

AACCAAACTGAACGATTAAATGCAGGATTATATTTTCTATTTTATACCCT

GGTTGGTTCGTTACCCTTACTAGTAGCATTAATTTATATCCAAAAATCTT

CAGGATCCTTAAATTTTATTATATCAATATATCAATCATCCAATCTTCCT

ATATCC----------TGAACAAATGACATTCTGTGACTAGCATGTATTA

TGGCTTTTATAGTTAAAATGCCTCTATACGGCCTCCACCTCTGATTACCA

AAAGCCCATGTTGAGGCCCCTATTGCTGGTTCTATAGTTTTAGCCGCTAT

CCTGCTAAAACTCGGTGGATACGGAATAATTCGAATTTCAACTTTACTTC

ATCCCATTACATGTAACATAGCCTACCCTTTTATTATGCTATCGCTATGA

GGAATAATCATAACAAGCTCAATTTGCCTACGACAAACAGACTTAAAATC

TCTCATCGCTTACTCCTCAGTAAGTCATATAGCACTAGTAATTGTAGCAA

TCATAATTCAAACTCCCTGAAGTTTTATGGGAGCCACAGCACTAATAATC

GCCCACGGACTTACATCTTCTACGCTATTCTGTTTAGCAAACACCAATTA

TGAACGAATCCACAGTCGAACTATAACACTAGCCCGAGGCTTGCAATCTA

TCCTTCCCCTTATAGCAACATGATGAGTTCTAGCCACCCTAACTAATTTA

GCCCTCCCACCTTCTATTAACCTAATCGGTGAATTATTTATTGTTATAGC

ATCATTCACTTGATCAAATATAACAATTATCTTAACTGGTCTAAACATAT

TAATCACAGCCCTCTACTCATTATATATACTAATTATAACACAACGAGGA

AAATTTACGTACCATACATTAAATATTAATCCTTCTTTCACACGAGAAAA

TACACTTATATTTCTTCATCTCTTTCCACTTGCCATTCTATCAACAAACC

CTGCCATTATTCTGGGTCAATTATACTGTAAATATAGTTTAAGCAAAACT

TTAGATTGTGAATCTAACAATAGAGAATCATAA--CCTCTTATTTACCAA

GAAAGCAT---GCAAGAACTGCTAATTCATGC-CCCCGTGATTACACCCA

CGGCTT--------------------------------------------

-------------------TCTTA-ACTTTTTTAGGATAGTAGTA-ATCC

GTTGGTCTTAGGAACCAAAAA-A-TTGGTGCAACTCCAAACAAAAGTAAT

AAATA---TATTCTCTTCACTTATCCTTACATCACTCGTTACCCTCTCAT

TTCCTATTTTTCTCACCATATCAGACTACCATAAACACATTAATTACCCA

AACTACGTAAAAATCTCTATCATCTGTGCACTATCATTTTGCATAGTACC

AACACTAATGTTTATTAACTCAAATTATGAACTTGTCATCTCAAACTGAC

ACTGAATGACTATTCAAACATTCACCCTTTCCATAAGCTTTAAACTAGAT

TATTTTTCTATATTATTCATTCCCGTAGCATTATTCGTTACATGGTCAAT

TATAGAATTCTCAATATGATACATGCACTCCGACCCTTTTATTAACCGTT

TCTTCATATATCTCCTCTTATTCCTTATCACTATAATGATTCTAGTTACA

TCTAACAACCTATTTCAACTATTCATTGGCTGAGAGGGAGTAGGTATCAT

ATCTTTCTTATTAATCGGTTGATGGTATGGTCGAACAGACGCTAATACAG

CAGCCCTTCAAGCTATCCTATACAACCGAATTGGAGACATTGGGTTTGTT

CTAGCTATAGCATGATTCTTACTTAACTCAAATTCATGAGAACTTCAACA

ACTATTCATAATAGACGTATCCTTA------TTTCCTCTACTAGGACTTC

TCTTAGCCGCCACAGGAAAATCTGCCCAATTCGGCCTTCACCCTTGACTG

CCCTCTGCTATAGAAGGCCCAACCCCCGTATCAGCTTTACTTCATTCCAG

TACAATAGTAGTAGCAGGAGTCTTTCTCCTCATCCGCTTCTACCCGCTAA

TA-GAACATAACAAAGTCATCCAAACACTCACTCTCTGCTTAGGAGCTAT

TACCACTCTATTCACCGCCATTTGTGCTCTTACTCAAAATGACATTAAAA

AGATTATCGCATTCTCCACTTCAAGCCAACTAGGATTGATAATAGTGACT

ATTGGAATCAACCAGCCCCACCTAGCCTTTCTCCATATTTGCACACACGC

ATTCTTTAAAGCCATATTATTTATATGCTCAGGATCAATTATCCATAACT

TAAACGATGAACAAGACATTCGAAAAATAGGAGGACTATTTAAAGCTCTC

CCATTTACTTCATCCTCACTTATTATCGGCAGCTTAGCACTAACAGGAAC

TCCATTCCTAACCGGATTTTACTCTAAAGACCTAATCATCGAGTCTGCAA

ACACGTCATATACCAACGCCTGAGCCCTAACTATTACTCTCTTTGCCACC

TCTCTAACCGCTGTCTATAGTACACGAATTATTTTTTATGCCCTTATAGG

ACAGCCTCGATTCTCTTCACTAACCTCAATCAACGAAAATAATCCCCAAC

TACTTAATTCAATTAAACGCCTTCTTATTGGAAGTATTATTGCAGGATTT

ATTCTTTCTTATAACATCCCACCTATAAATATCCCAGTCCTAACCATACC

CATCCATCTTAAACTCACAGCACTACTAGTAACCATCTTAGGATTTGTCA

TTGCTATAGAACTTAACTCAATAACTCTTTATCTAAAAACTAAAATGTAC

TCAAACACATCA-AAATTTTCAACCTTACTAGGCTATTTCCCTACTGTTA

TTCACCGATTTAATCCCCATCTTAATCTCATCATAAGCCAAAAACTTTCA

TCAACCCTATTAGACCTGGTCTGACTAGAAAAAACTATCCCCAAATCTAC

CACTAACTTTCAC---TCAACAGCCTCCACTATAACCTCTAACCAAAAGG

GCCTCATCAAATTATATTTCCTATCGTTCCTGACTTCAACACTTCTAGCA

ATCATAGCCGTATTCTA---------------------------TTTCCA

CGTGTAATTTCAATCACAATAAAAATACTAACAAACAATGACCAACCAGC

TACAACCATCAATCAACTTCCACAGTTGTATATAGCTGCCACCCCTATTG

AATCCTCACGAACCAGCCCTAACTCACCCCCCTCAAACACTATTCAATTC

CCTGAATCCTTAAACTCAATTACAACTTCCA---------TCTCATCATA

TAAAACCATAAACATAATAATTAAAAACTCCACTAAAAATCCCAATAACA

AAACCCCCCAAATAACTACATTGGATCCTCACGTTTCTGGGTATTCCTCC

GTTGCTATAGCCGTAGTATAACCAAATACCACTAATATTCCCCCCAAATA

AATTAAAAATACTATTAAACCTAAAAAAGACCCTCCAAAATACAATACAA

CCCCACACCCAATTCCCCCACTAACAATTAACCCTAGACCCCCATAAATA

GGAGAAGGTTTTGAGGAAAATCCTACAAAACCTAAAACAAAAAGTATACT

CAATAAATACGTTACATACGTCATTAT---TTTTA-CATGGAATTTA-AC

CATGACTAATGACATGAAAAATCATCGTTGT-TATTCAACTATAAAAACG

-CT----AATGACAAACATCCGCAAAACTCACCCTTTAATTAAAATCGTC

AACCACTCCTTCATCGACTTACCCGCACCCTCCAACATCTCTGCATGATG

AAACTTTGGGTCTCTTCTAGGCCTCTGCCTAGCAATCCAAATCCTCACTG

GATTATTTCTAGCAATACATTATACATCTGATACTATAACAGCCTTTTCA

TCAGTTACTCACATTTGCCGAGATGTAAATTATGGTTGACTAATCCGCTA

TATACATGCTAACGGCGCATCTATATTTTTTATCTGCCTCTTCCTTCATG

TAGGCCGAGGACTATACTATGGCTCATATACTTATTTTGAAACATGAAAC

ATTGGAGTCATTCTCTTATTCGTAGTAATAGCCACAGCTTTCATGGGCTA

TGTTCTTCCCTGAGGTCAAATATCATTCTGAGGAGCAACTGTAATTACTA

ACCTTTTATCCGCTATTCCGTACATTGGAACAACCCTAGTAGAATGAATT

TGAGGCGGCTTCTCAGTAGATAAAGCTACTCTAACACGATTTTTCGCATT

CCACTTTATTCTTCCATTTATTATCGCAGCTCTAGTTATAGTTCACCTTC

TTTTCCTTCATGAAACTGGATCAAACAACCCTTCAGGCCTTATTTCTGAT

TCAGATAAAATCCCCTTTCACCCATATTACACCATCAAAGATATCCTTGG

AGTCCTTCTTCTTATTTTAGCCCTAATAACTCTGGTCCTGTTTTCACCTG

ATCTTCTAGGAGACCCTGATAATTATACACCCGCAAACCCCCTAAGCACC

CCACCTCACATCAAACCAGAATGATATTTCCTATTTGCCTACGCTATCCT

CCGATCTATCCCTAACAAACTAGGAGGTGTATTAGCCTTAGTATTCTCAA

TTCTTATCCTAATACTTTTCCCACTTCTCCATCTATCTAAACAACGAAGC

ATAATATTTCGACCACTAAGTCAATGCATATTCTGAATTCTAGTAGCAGA

CCTATTTACATTAACCTGAATTGGAGGACAACCCGTTGAATATCCATTTA

TTATTATTGGCCAACTAGCATCAATCCTTTACTTCACTATTATTCTCCTA

ATCTTACCAACCGTCAGCCTAATCGAAAATAAACTTCTTAAATGAAGA--

--GCCCTAATAGTATAA-AC--ATTACTTTGGTCTTGTAAACCAAAAATG

AAGTT-ACAAAC--TTCTTAGAGCAATAAATCAGGGAAGAAAATAAACTT

TCCACCTTCAACTCCCAAAGCTGATATTTCTTACTTAAACTATTCCCTG-

--------------------------------------------------

--------------------------------------------------

--------------------------------------------------

--------------------------------------------------

--------------------------------------------------

--------------------------------------------------

--------------------------------------------------

--------------------------------------------------

--------------------------------------------------

--------------------------------------------------

--------------------------------------------------

--------------------------------------------------

--------------------------------------------------

--------------------------------------------------

--------------------------------------------------

--------------------------------------------------

--------------------------------------------------

--------------------------------------------------

--------------------------------------------------

--------------------------------------------------

--------------------------------------------------

--------------------------------------------------

--------------------------------------------------

------

>Ictidomys_tridecemlineatus Ictidomys tridecemlineatus mitochondrion, complete genome.

GTTAATGTAGCTTAA--CT----CAATAAAGCAAAGCACTGAAAATGCTT

AGATGGGTATTTTGT-ACCCCATAAACA-ATA--GGTTTGGTCCTGGCCT

TTTCATTAGCTTTTAGTTAACTTATACATGCAAGCATCCCCGCCCCAGTG

AGAATGCCCT-CTATATCTAT-AAC---TGATCAAAAGGTGCAGGCATCA

AGTTCACT-ACTCCTAGTAGCTCATAACGCCTTGC-TCCACCACACCCCC

ACGGGACACAGCAGTAATTAACATTAAGCCTATAAACGAAAGTTTGACTA

AGTTAAGCTA--AATTTAGGGTTGGTAAATTTCGTGCCAGCCACCGCGGT

CATACGATTAACCCTAGTTAATGAAAT-AC-GGCGTAAAGCGTGATTAAG

AGA------CTAAT-TAGATAAGATTAAAATTATACTAAACTGTAAAAAG

TC-TTGGTGTTAATGAAAATCAAATACGAAAGTAATCTTA---AATTTTC

TGAATTCACGATAGCTAAGACTCAAACTGGGATTAGATACCCCACTATGC

TTAGCCCTAAACATAAATA-TTCAAC-AAACAAGAATATTCGCCAGAGTA

CTACTAGCAATGGCCTAAAACTCAAAGGACTTGGCGGTGCTTTATACCCC

TCTAGAGGAGCCTGTTCTATAATCGATAAACCCCGATACACCTCACCACC

TTTAGCAAATATCAGCCTATATACCGCCATCTGCAGCAAACCCTAA-AAA

GGTCCTATAGTAAGCAAGAAAATTCT--ACATTAGTACGTTA-GGTCAAG

GTGTAGCCTATAAGGTGGG-AAGAAATGGGCTACATTTTCTATTTTCCTA

GAACA------AAT---TACAATAGCTTTTATGAAATTCAG---AGCATA

AGGCGGATTTAGTAGTAAGTT-AAGAATAGAGAGCTTAACTGAATAG-GG

CAATAAAGCACGCACACACCGCCCGTCACCCTCTTCAAATATATCT----

TCATAGTTCTATAAGTAATATATTAAACCAAA--T--ATATA--AGAAGA

GATAAGTCGTAACAAGGTAAACATACTGGAAAGTGTGTTTGGAAGAATCA

AAATGTAGCTTA--TTAACTAAAGCACCCGGCTTACACCCGAGAGATTTC

ACTCAAT-ATGAACATTTTGA-ACT-AATACT-AGCCCAACTTTTA----

-TCCCCTCCAAATACCATTCACCTAATAAATAAAACATTCATC-TA-AA-

--TAAA-GTATAGGAGATAGAAATTTA--TATTA--GGAGCTATAGAAAA

AGTACCGTAAGGGAAAGA-TGAAAGAATAAATTTATAGTACAAAAAAGCA

AAGATTAACCCTTTTACCTTTTGCATAA-TGATTTAACCAGAAAATGTTT

GACAAAAAG-AATTTAAGCCAAATACCCCGAAACCAGACGAGCTACTTAT

GAGCAGCC--GATAAGAGCCCATCCATCTATGTTGCAAAATAGTGGAATG

ACTCATAAGTAGAGGTGAAAAGCCTACCGAGCCTGGTGATAGCTGGTTGT

CCA-GACTAGAATTTTAGTTCTACTTTAAATTTACCTCAAGCACT-TAT-

AA-GCCAAATGTAAATTTAAATGCTATTCTAAAGAGGGACAGCTCTTTAG

AGTCAAGAAATAAACCTTATTTAGAGAGTAA-----ATTTATTAATT--T

CCATAGTTGGCCTAAAAGCAGCCATCAATTAAAAAAGCGTTAAAGCTTAA

CCCAATCA-C---CAAAACTTAATACCTGACTAC---TTCCATGACCT--

CCCAAATCAACA-CTGGACTAATCTATTTCATAAT-AGAAGAAATTATGT

TAAAATAAGTAACAAGAA-AGTAT-T-CTCCCTGCATAAGCTTATATCAG

ATCGAA-TGATTCACTGATAGTTAACAGCTTCATA-----ATATTAAACA

AACAGAACAACCATTATTACTTATACTGTTAACCCAACACTGGTATGCA-

--TAAAGGGAAAGATTAAATAAAGTAAAAGGAACTCGGCAAACACTAACC

CCGCCTGTTTACCAAAAACATCACCTCTAGCATTAATAGTATTAGAGGCA

CTGCCTGCCCAGTGACA-CACG----TTTAACGGCCGCGGTATCCTGACC

GTGCAAAGGTAGCATAATCACTTGTTCTTTAAATAAGGACTAGTATGAAT

GGCTTGACGAGGGTTTAACTGTCTCTTACTTTTAATCAGTGAAATTGACC

TTCCCGTGAAGAGGCGGGAATTTTATAATAAGACGAGAAGACCCTATGGA

GCTTTAATTTAAC-AGTCTCACAGCCTTAATTATATCTT----------A

GGAGTTAA-AACTATTGTTCA-TAGACTAGAAATTTTGGTTGGGGTGACC

TCGGAGTACAAACTAACCTCCGAATGATA-ATAATCT-AGACATTACATG

TCCAAATTACAACTC----ATTAATTGACCCAA-A---TTATTGATCAAC

GGAACAAGTTACCCTAGGGATAACAGCGCAATCCTACTCAAGAGTCCATA

TCGACAGTTAGGGTTTACGACCTCGATGTTGGATCAGGACATCCAAATGG

TGTAACCGCTATTAATG-GTTCGTTTGTTCAACGATTAA-AGTCCTACGT

GATCTGAGTTCAGACCGGAGAAATCCAGGTCGGTTTCTATCTATTACTA-

CATTTCTCCCAGTACGAAAGGACAAGAGAAATAAGGCCAATTATACAC-T

CATGCCTTAAGTT-AATGGATGAT-ATAATCTTAATCCAGTAAAATATTT

TAAAC--AATCTGCCCTAGAATAGGG--CTT-GTTAAGATGGCAGAGCCT

GGTAA-TTGCGTAAGACTTAAAACTTTATT-TCAGAGGTTCAACTCCTCT

TCTTAACA-TTA--------------------------------------

------------------------------------------ATGTTTTT

AGTTAACCTTCTACTTTTAATTATCCCAATCTTAGTAGCCATAGCCTTTC

TTACCCTAATTGAACGAAAAATATTAGGATATATACAACTTCGCAAAGGC

CCTAATGTTGTTGGACCTTATGGCCTACTCCAACCATTTGCTGACGCAAT

AAAGCTATTCATTAAAGAACCCCTAAAACCCTTAACATCATCTATTATAC

TATTTATTATTGCTCCAACCCTCGCCCTAACACTGGCATTTACCATATGA

ATTCCCTTACCCATACCCATACCCCTTATTAACATAAATATAGGAGTCCT

ATTTATCTTAGCCACTTCAAGCTTAGCCGTATATGCAATTTTATGATCTG

GATGAGCTTCCAATTCTAAGTATGCTTTAATTGGAGCTCTACGAGCCGTA

GCACAAACTATCTCATACGAAGTAACATTAGCAATTATCCTTCTCTCAGT

ACTTCTAATAAATGGATCCTTTACTTTGTCCACACTCATTACTACTCAAC

AATTTTCATGACTCTTATTTCCAACATGACCTCTAGCAATAATATGATTT

ATTTCAACATTAGCAGAAACTAATCGAGCTCCATTTGATTTAACAGAAGG

AGAGTCAGAACTCGTATCAGGATTTAATGTTGAATACGCAGCCGGTCCCT

TTGCCTTATTTTTCATAGCTGAATATACTAACATTATCATAATAAACGCA

TTAACAGTAACCTTATTTATAGGAGCACTACTAAACCCTATCTTACCCGA

AACTTTTACATTAAACTTTGTCCTAAAAACACTCATTTTAACTTCCACTT

TTCTATGAATCCGAGCATCCTATCCTCGATTCCGTTACGACCAACTCATG

CACCTCCTATGAAAAAACTTTTTACCCCTAACTCTAGCCTTATGCATATG

ACATATCTCTCTCCCAATTATAACCGCATGCGTACCACCCCAAATATAA-

GAAATATGTCTGATAAAAGAGTTACTTTGATAGAGTAAATTATAGAGGTT

TA-AATCCTCTTATTTCTAGAACTATAGGGATTGAACCTAATCCTAAGAA

TTCAAAATTCTTCGTGCTACC-TCT-TACACCATGTCCTAAAT--A----

GTAAGGTCAGCTAAAT-AAGCTATCGGGCCCATACCCCGAAAATGTTGGT

TTATATCCTTCCCGTACTAATTAACCCCTTGACTTCCTCCGCAATCTACT

TTACTCTATTTTCTGGGACTATAATTACACTTTTCAGCTCACATTGACTT

CTAACTTGAGTAGGCCTTGAAATAAGCATACTAGCTATTATCCCCATTCT

AATTAACAAAGGAAACCCTCGATCCACAGAAGCTGCATGCAAATATTTTC

TCATTCAAGCCACCGCATCAATAATCCTAATAATAGGCACAATAATTAAC

TTCATAGACTCAGGTCAATGAACTCTGTCCAATCCATATAATCAAATCTC

ATCATTCATATTTACAATTGCACTTTCAATAAAAATAGGACTCGCTCCAT

TTCACCTATGAGTTCCAGAAGTAACCCAAGGAATTCCACTCAAATCAGGA

CTAATCATGCTAACATGACAAAAAATTGCCCCAATCTCTATCGTATATCA

AATCGCACCCTCTATAAACCCAACCCTCATACTACTTATAGGGACCCTAT

CAGTCATACTAGGAGGATGAGGAGGACTTAACCAAACCCAACTACGAAAG

ATCATAGCATACTCATCAATCGCCCATATAGGATGAATAATAGCAATCGT

CACATATAATCCAACCTTAACAATATTTAACCTAGTCATTTATATTATAC

TTACCATCAATATATTTATACTTCTCCTATTTTATAAAAAAACTTCCACC

CTTTCCCTGTCTAACATATGAAATAAGTTCCCTCTTCTAACACCCACAAT

TTTAATTGTACTAATATCACTAGGAGGATTACCTCCTCTAACAGGATTTA

CACCAAAATGAATTATCCTTAAAGAACTTATTTCAAATAACAACATCATC

TTCTCTTCACTAATAGCAACTCTAGCACTACTAAACCTATACTTCTATAC

ACGACTTATCTATTCAACATCTCTAACCTTATTCCCATCATTTAATAACA

CTAAGATAAAATGACAATTCGAAAATATGAAACTCACACCTTTTCTACCC

ACACTAATTATTACCTCTGCTCTTTCTCTCCCACTAATACCCCTC-TTCT

CACTCCT--AAACT----AGGAATTTAGGTTAAT--A-TAGACCAAGGAC

CTTCAAAGTCCTAAGTAAGTA-TCCAAT---ACTTAATTCCTGT------

---G----CTAAGGACTGCAAGACTTTATCCTACAT----CAATTGAATG

CAAACCAATCACTTTAA--TT-AAGCTAAGCCCTTA-ATC------TCTA

GACTGATG--GGATTTAAA-CCCAT-AAGACCTTAGTTAACAGCTAAACG

CCCTACTCAACTGGCTTCAATCTA-CT-TCTCCCGCCGTAAGGGAAAAAA

-----GGCGGGAGAAGCCCCGGCAGAG-T-TGA-AGCTGCTCCTTT--GA

-----------------------------ATTTGCAATTCAATA--TGAA

TA-TTCACCTCGGGACT-T-----GGCAAAAAGAGGA----CT-CAACCT

CTGTCTTTAGATTTACAGTCTAATGCTTA--CTCAGCCATTTTACC----

ACCTACCTATGTTCATCAACCGTTGATTCTTCTCAACTAATCACAAAGAT

ATTGGAACACTTTACCTTCTATTTGGCGCTTGAGCTGGAATAGTAGGAAC

TGCACTTAGTCTACTAATCCGTGCTGAGCTAGGTCAACCTGGAGCTCTAT

TGGGTGACGACCAAATTTACAATGTTATTGTTACTGCTCATGCATTCGTT

ATAATTTTCTTTATAGTTATACCAATTATAATTGGTGGATTTGGAAACTG

ATTAGTACCCCTAATAATTGGAGCTCCTGATATAGCATTCCCACGAATAA

ATAATATAAGCTTCTGACTTCTTCCCCCTTCTTTTCTCCTCTTACTCGCT

TCTTCTATGGTTGAGGCAGGAGCAGGAACTGGCTGAACCGTTTATCCTCC

ATTAGCTGGAAATCTTGCCCATGCAGGAGCTTCAGTAGACCTAACCATCT

TCTCCCTTCACTTAGCAGGAGTTTCATCCATCCTAGGAGCAATTAACTTT

ATTACAACAATTATTAACATAAAACCACCTGCCATATCTCAATATCAAAC

TCCTTTATTTGTATGATCCGTGTTAATTACAGCAGTACTTCTACTCTTGT

CCCTTCCGGTTCTTGCGGCAGGAATCACTATATTACTTACAGATCGTAAT

CTTAATACCACATTTTTTGACCCTGCTGGAGGTGGAGATCCTATCCTCTA

TCAACATCTATTTTGATTCTTTGGACACCCTGAAGTCTATATTCTTATCC

TTCCAGGATTTGGTATAATTTCTCATATCGTAACATATTACTCAGGAAAA

AAAGAACCATTCGGTTATATAGGAATAGTATGAGCTATAATATCTATTGG

TTTCCTTGGATTTATTGTATGAGCCCATCACATATTTACCGTTGGAATAG

ATGTAGATACTCGAGCTTATTTTACATCCGCAACTATAATTATTGCTATT

CCTACAGGAGTAAAAGTTTTTAGCTGATTAGCGACCCTGCACGGAGGAAA

TATTAAATGATCACCTGCAATACTATGAGCACTCGGCTTTATTTTCTTAT

TCACTGTAGGAGGCCTAACAGGTATTGTCTTAGCCAACTCCTCATTAGAT

ATTGTCCTACACGATACATATTATGTAGTAGCCCACTTTCACTATGTATT

ATCAATGGGAGCTGTATTTGCCATTATGGGAGGATTCGTTCACTGATTCC

CTCTTTTTTCTGGTTACACACTAAATGATATATGAGCTAAAATTCATTTT

ACTGTAATATTTGTTGGAGTAAATTTAACTTTCTTTCCTCAACATTTCCT

AGGGTTATCAGGTATACCACGTCGATATTCTGATTACCCAGATGCTTACA

CAGCATGAAATACTGTATCCTCAATAGGTTCCTTTATTTCTCTCACAGCT

GTTATAATCATAATTTTTATAATTTGAGAAGCATTTGCATCAAAACGAGA

AGTCCTCACCGTAGAACTAACGCCAACTAACCTAGAATGACTGCATGGGT

GCCCTCCACCCTATCACACATTTGAAGAACCTACTTACATTAAAGCTTAA

AT--C--------------AAGAAAGGAAAGAATCGAACTTCCTAAGACT

AGTTTCAAGCCAGCCTCATAACCATTATGA-CTTTCT---TCAT----GA

GATATTAGTAAAAT-AATTACATAACTTTGTCAAAGTTAACTTATAGGTT

A------AATTCCTATATATCTC-TATGGCTTACCCGCTCGAATTAGGAT

TTCAAGACGCTACATCTCCCATTATAGAAGAGCTTTTACACTTTCATGAC

CATACCCTTATAATTGTTTTCTTAATTAGCTCCTTAGTCCTCTATATCAT

CTCATTAATATTAACCACAAAATTAACTCATACAAGCACTATAGATGCTC

AAGAAGTAGAAACTATCTGAACCATCCTTCCTGCTATTATCCTTATTCTG

ATTGCTCTCCCCTCTCTACGTATTCTATACATAATAGACGAAATTAACGA

CCCATCCTTAACAGTAAAGACAATAGGCCACCAATGATATTGAAGCTATG

AATACACTGACTACGAAGACCTTAACTTTGACTCTTATATAATTCCAACT

TCAGACTTAGCCCCCGGAGATCTACGACTTCTGGAAGTCGACAATCGAGT

TGTTCTTCCAATAGAGTTACCTGTACGAATATTAATCTCATCTGAAGATG

TACTTCACTCTTGAGCAGTCCCATCCCTTGGCTTAAAAACAGATGCCATC

CCAGGCCGACTTAATCAAGCTACGCTAACATCAACACGACCAGGACTTTA

TTATGGGCAATGCTCTGAGATTTGCGGATCAAATCATAGCTTCATACCTA

TTGTTCTTGAATTAGTTCCACTAAAACACTTTGAAAACTGATCCTCATCA

ATACTATAA--------------------------------ATT-CATTA

TGAAGCTG--AA-TAGCGTCAACCTTTTAAGTTGAAGATTAGGAGT-T-G

A-AC-CTCCTCATAATGAAATGCCCCAACTAGACACATCCACATGATTTA

TCACAATTCTATCAA-TGATTCTAGCCCTTTTCTTTATATTTCAACTTAA

AATCTCAAATCA--CTCTTATCCGTCTAATCCCTCTCTTAAATATACTAA

-ACTAATTGAACATAAAACTCCTTGAGAAGAAAAATGAACGAAAATCTAT

TTGCCTCTTTCATTACCCCTACACTTATAGGTCTCCCTATTGTCCTTCTT

ATTATTATATTTCCCAATTTACTCTTTCCTTCACCTACTCGACTAATAAA

CAACCGTTTAGTATCATTCCAACAATGACTAATTCAACTTGTACT-AAAA

CAAATAATGGCAATGCACAACCCTAAAGGACGTACCTGATCCTTAATACT

AATTTCATTAATTATATTTATTGGCTCAACTAACCTTCTAGGCCTTCTAC

CTCATTCTTTCACACCTACAACCCAATTATCAATAAACTTAGGAATGGCC

ATCCCTCTATGAGCAGGAGCCGTAATCACCGGATTCCGTCATAAGA-CTA

AAGCATCATTAGCCCACTTTCTCCCACAAGGAACCCCAATTCCTCTAATC

CCTATACTTATTATTATTGAGACAATTAGCCTTTTTATTCAACCTATAGC

ATTAGCTGTACGATTAACAGCTAATATTACAGCTGGTCATCTTCTTATAC

ATTTAATTGGAGGAGCAACTCTTGTATTAACATCTATTAGCCCTCCCACA

GCTATTCTTACTTTTATTATTCTCGTCCTATTAACAATACTCGAATTTGC

AGTTGCATTAATTCAAGCCTATGTTTTTACCCTCTTAGTAAGTCTTTATT

TACACGATAATACTTAATGACCCACCAAACCCACGCCTATCATATAGTTA

ATCCTAGTCCCTGACCATTAACAGGGGCCCTCTCCGCCTTACTCCTAACG

TCTGGCCTAGTAATATGATTTCACTTCAACTCCTCCTTCCTTCTTACACT

AGGCCTAACAGCTAATATCCTAACAATATACCAATGATGACGAGACGTCG

TGCGAGAAGGTACATTTCAAGGTCACCACACATCAATTGTTCAAAAAGGT

TTACGATATGGCATAGTACTATTTATTATTTCAGAAGTATTCTTCTTTGC

CGGATTTTTCTGAGCATTCTACCATTCTAGCTTAGCCCCAACCCCCGAAT

TAGGCAGTTGCTGACCTCCAGTAGGAATCAACCCACTTAATCCCTTAGAA

GTTCCACTATTAAATACCTCTGTTCTTTTAGCTTCGGGAGTTTCAATTAC

TTGAGCTCACCACAGCCTAATAGAAGGAGACCGAAAACATATAGTCCAAG

CACTATCAATTACAATTGCTCTAGGACTTTATTTTACTCTTCTCCAAGCC

TCTGAATATTTAGAGACTTCTTTTACAATCTCAGATGGAGTATATGGCTC

AACATTTTTCATAGCCACAGGCTTCCACGGCCTTCACGTCATTATTGGGT

CAACCTTTCTCCTAGTATGTCTCATTCGTCAACTAAATTTTCACTTTACA

TCAAGCCATCATTTCGGATTTGAAGCAGCCGCATGATACTGACACTTTGT

AGACGTAGTATGACTCTTCCTATACGTATCTATCTATTGATGAGGTTCAT

ATTCT-CTTAGTATTAA-TTAGTACAATTGACTTCCAATCAATTAGTTCT

GGAACTAACCCAGAAGAGAATAATAAACCTTATAGTATCCCTCTTTATAA

ATTCTTTCATTGCTCTACTATTAATTTCCGTAGCATTTTGACTACCCCAG

CTAAACGTGTATGCTGAAAAAGCAAGTCCTTACGAATGCGGCTTTGATCC

TATAGGATCTGCTCGCCTACCATTCTCAATAAAATTTTTTCTTGTTGCAA

TTACATTTCTTCTATTTGATCTAGAGATTGCTCTTCTTCTCCCCCTCCCC

TGAGCCTCCCAAACAAACAATCTTAATCTTATATTAACTATAGCCTTACT

TTTAGTCTTAATTCTCACTCTCGGACTAGCCTATGAATGAGTTCAAAAGG

GCCTAGAATGAATTGAATA------TGATAATTAGTTTAAAATAAAA-CA

AGTGATTTCGACTCACTAAATTATGGGTTA-C--CATAATTATCAA--AA

TGCCTATTATTGTTCTTAATACTATCATAGCTTATTCTACGTCCCTATTA

GGAATATTCATCTACCGATCCCACCTAATATCGTCACTCCTATGCCTAGA

AGGAATAATATTATCAATATTTGTCTTATGCTCACTCTTAATTATTAATT

TCCATTTTTCTTTATCATTTATGATTCCCATTATTTTACTAGTATTTGCT

GCATGTGAAGCAGCTGTAGGTCTAGCCCTTCTCGTAATAGTATCAAATAC

ATACGGTCTAGACTATGTCCAAAACCTAAATATTCTTCAATGCTAAAAAT

TGTTATTCCCACAATACTACTTGCCCCCCTTATATGATTCTCAAAACCCT

CCATAATCTGAATCAACCCCTCAGTTCACAGTCTGTTAATTAGCCTAATT

GTTCTTTTCACATTAAATCGTCCCACAAATACAGATCTAATTTTTTCGCT

AGCTTTCTTTACCGATCCTCTATCCTCCCCCCTATTAATTCTTACAGCAT

GACTCTTACCCCTTATAATTATAGCAAGTCAGAGCCATCTAACCCATGAA

CCATTAATTCGAAAAAAACTGTACATTCTTATATTAATCTCGCTACAATC

CTTTTTGATTATAACCTTCTCTGCTACTGAATTAATTATATTTTATATTC

TATTTGAGGCTACCTTAATCCCCACACTAATTATTATCACTCGATGAGGG

AACCAAGCTGAGCGATTAAATGCAGGATTATATTTCCTATTTTATACCCT

AGTAGGTTCATTACCCTTACTAGTAGCATTAATCTATATTCAAAAATCTA

CAGGATCCTTAAACTTTATCATCTCATTATACCAGTCACCTAACCTCTCC

ATATCC----------TGAACAAATGACATTTTATGATTAGCATGTATAA

TAGCTTTTATAGTTAAAATACCCTTATATGGTCTTCACCTTTGATTGCCA

AAAGCTCACGTTGAAGCCCCCATTGCTGGTTCCATAGTCCTAGCTGCTAT

CCTACTAAAACTTGGCGGATATGGTATAATCCGAATTTCAACTTTTCTAC

ATCCTATTACATGTAATATAGCGTACCCCTTTATTATGTTATCGTTATGA

GGTATAATTATAACAAGCTCAATTTGCTTACGACAAACAGACCTAAAATC

CCTCATTGCTTATTCATCAGTAAGCCACATAGCACTAGTAATTGTAGCAA

TCATAATTCAAACTCCTTGAAGCTTTATAGGAGCCACAGCACTAATAATT

GCCCACGGACTAACATCTTCCATATTATTCTGCCTAGCAAACACTAATTA

TGAACGAATCCACAGCCGAACTATAACTTTAGCTCGAGGCTTACAATCTA

TTCTCCCCTTAATAGCAACATGATGAGTTTTAGCTACCTTAACCAATTTA

GCTCTCCCACCTTCTATTAATCTAATTGGCGAGCTATTCATTATTATAGC

ATCATTTACTTGATCAAATATTACAATTATCTTAACCGGACTAAATATAC

TAATTACAGCCCTCTACTCACTATATATGCTAATCATAACACAACGAGGA

AAATTTACATACCATACATTAAATATTAGTCCCTCCTTTACGCGAGAAAA

CACGCTCATATTTCTTCACATTTTCCCACTTATTATTTTATCAACAAACC

CCACTATTATTCTGGGTCAATTATACTGTAAATATAGTTTAAGCAAAACT

TTAGATTGTGAATCTAACAATAGAGAATCGTAA--TCTCTTATTTACCAA

GAAAGCAT---GCAAGAACTGCTAATTCATGCTCCCCGTGTTTACATCCA

CGGCTT--------------------------------------------

-------------------TCTTA-ACTTTTATAGGATAGTAGTA-ATCC

ATTGGTCTTAGGAACCAAAAA-A-TTGGTGCAACTCCAAATAAAAGTAAT

AAATA---TATTCTCTTCACTTATACTCACATCACTCGTAACCCTCTCAT

TTCCTATTTTTCTCACCATGACTAATTATTATAAACATATCAACTACCCA

AACTACGTAAAAATCTCTATTATTTGTGCATTATCACTCTGCATAGTACC

AACACTAATATTTATTAACTCAAACTATGAACTTATTATCTCAAACTGAC

ACTGAATAACTATCCAAACATTCACTCTCTCCATAAGCTTCAAGTTAGAC

TATTTTTCTATACTTTTTATACCTGTAGCATTATTTGTGACATGGTCAAT

TATAGAATTTTCAATATGATATATACACTCAGACCCTTTCATTAACCGCT

TCTTCAAATACCTACTCCTATTCCTTATCACTATAATAATTCTAGTTACA

TCCAACAATCTATTCCAACTATTCATTGGCTGAGAAGGAGTAGGCATCAT

ATCCTTTTTATTAATTGGCTGATGATACGGTCGAACAGATGCTAATACAG

CAGACCTTCAAGCTATCCTATATAACCGAATTGGAGATATTGGATTTGTA

CTAGCTATAGCATGATTCTTACTCAACTCAAACTCATGAGAATTTCAACA

ACTTTTCATGATAGATGTATCTTTA------TTCCCCCTACTAGGCCTAC

TCTTGGCCGCCACAGGGAAATCTGCCCAATTCGGTCTTCATCCCTGATTA

CCCTCTGCTATAGAAGGTCCAACCCCTGTATCAGCTCTACTTCACTCCAG

TACTATAGTAGTAGCAGGAGTTTTTCTCCTCATCCGCTTCTACCCGCTAA

TA-GAACATAATAAAATCATCCAAACACTTACCCTCTGCTTAGGAGCTAT

TACTACTCTATTTACTGCCATCTGTGCTCTTACCCAAAATGACATTAAAA

AGATTATTGCATTTTCCACTTCAAGCCAACTAGGATTAATAATAGTAACC

ATTGGAATTAATCAGCCTCACTTAGCCTTTCTCCACATTTGTACACATGC

ATTCTTTAAAGCCATATTATTTATATGCTCAGGATCAATTATTCATAACC

TAAACGATGAACAAGACATTCGAAAAATAGGAGGACTATTCAAAGCCCTT

CCATTTACCTCATCTTCACTCATTATTGGCAGTCTAGCACTTACAGGAAC

CCCTTTCCTAACTGGATTCTACTCTAAAGACCTAATCATTGAATCCGCAA

ACACGTCGTATACCAACGCCTGAGCCCTAACTATTACTCTCCTCGCCACC

TCCCTAACTGCTGTCTACAGCACACGAATTATTTTCTATGTTCTAATAGG

ACGACCTCGATTTTCTACACTAACTTCAATTAATGAAAATAACCCCCAAC

TACTTAACTCAATTAAACGTCTTCTCATTGGAAGTATTATTGCAGGATTT

ATCCTTTCGTATAATATTCCACCTATAAATATCCCAGTCCTAACTATACC

CACCTACCTGAAACTTACTGCACTATTAGTAACCATTCTAGGATTCGTCA

TTGCTATAGAACTAAACTCAATAACTCTTTATCTAAAAACCAAAATATAC

TCAAACACATCA-AAATTTTCAACCTTACTAGGCTATTTTCCTACCATCG

TTCATCGATTCAACCCTCATCTTAACCTTGTAATAAGCCAAAAATTCTCA

TCAACCCTATTAGACTTAGTATGACTAGAAAAATCTATTCCCAAACTTAC

AACTAACCTTCAC---TCAACAGCCTCCACCATAACCTCTAATCAAAAAG

GCCTCATCAAATTATACTTTTTATCATTCTTAACTTCAACACTCCTAGCA

ACCATAATCGTATTCTA---------------------------TTTCCA

CGCGTAATCTCAATTACAATAAAAATACTAACAAACAACGATCAGCCAGC

TACAACTATTAATCAACTTCCACAATTATATATAGCTGCCACCCCTATTG

AATCCTCACGAATTAATCCCAACTCATCCCCCTCAAACACTATCCAATTT

CCCGAATCCTTAAACTCAATTACAACTTCTA---------CTTCATCATA

TAAAACTATAAACATAACAATTAAAAATTCCACTAAAAACCCTAATAACA

AAGCCCCCCAAATAACTACACTTGATCCCCATGTCTCCGGATATTCCTCT

GTTGCTATAGCCGTAGTGTAGCCAAACACTACCAATATCCCCCCTAAATA

AATTAAAAACACCATTAAACCTAAGAAAGACCCTCCAAAATATAATACTA

CTCCGCATCCAATCCCCCCACTAACAATTAATCCTAAACCTCCGTAAATA

GGAGAAGGTTTTGAAGAAAATCCTACAAAACCTAAAACAAAAAGTATGCT

TAATAAATATGTTATATAAGTCATTAT---TTTTA-CATGGAATCTA-AC

CATGACTAATGACATGAAAAATCATCGTTGT-TATTCAACTATAAAAACA

-CT----AATGACAAACATTCGTAAAACTCACCCTTTAATCAAAATTGTT

AATCACTCCTTTATTGACTTACCTGCACCTTCCAACATCTCCGCATGATG

AAACTTTGGATCCCTACTAGGTCTCTGCCTAGCCATCCAAATCCTCACTG

GGTTATTCCTAGCAATACATTATACGTCTGATACTATAACAGCCTTTTCA

TCAGTCACCCACATCTGTCGAGATGTTAATTACGGCTGACTTATCCGCTA

TATACATGCTAATGGTGCATCCATATTTTTTATCTGCCTTTTCCTTCATG

TAGGCCGAGGATTATACTATGGCTCATACACTTACTTTGAAACATGAAAC

ATTGGAGTTATCCTCCTATTTGCAGTAATAGCCACAGCTTTTATAGGCTA

TGTCCTTCCCTGAGGCCAAATATCATTCTGAGGGGCAACCGTAATTACCA

ATCTTTTATCTGCCATCCCATACATCGGTACAACCCTAGTAGAGTGAATT

TGAGGTGGTTTCTCAGTAGACAAAGCTACCCTAACTCGATTCTTCGCATT

CCATTTTGTTCTTCCATTTATCATCGCAGCTCTAGTCATAGTCCACCTTC

TTTTCCTTCATGAAACTGGGTCAAACAACCCCTCAGGACTTATTTCTGAC

TCAGACAAAATCCCCTTTCACCCATATTACACTATTAAAGACATCCTCGG

AGTCCTCCTTCTTATTCTAGCTCTGATAACCCTAGTCCTATTTTCACCTG

ACCTTCTAGGAGATCCTGATAATTACACACCCGCAAATCCTTTAAGTACC

CCACCTCATATTAAACCAGAATGGTACTTCTTATTTGCCTACGCCATTCT

ACGATCTATTCCCAACAAACTAGGAGGCGTCCTAGCCTTAGTCTTCTCAA

TCCTTATTCTAATACTCTTTCCACTGCTTCACTTATCTAAACAACGTAGC

ATGATATTCCGACCATTAAGTCAATGTATATTCTGAATCCTAGTAGCAGA

CCTATTTACATTAACCTGAATCGGAGGACAACCCGTTGAATATCCATTTA

TCATTATTGGCCAACTAGCATCGGTCCTATACTTCACCATCATTCTCTTA

ATTCTACCAACCATCAGCCTAATTGAAAATAAACTTCTTAAATGAAGA--

--GCCCTAATAGTATAA-AC--ATTACTTTGGTCTTGTAAACCAAAAATG

AAGTT-ATAAAC--TTCTTAGAGCATTCCATCAGGGAAGAAAATACACTT

TCCACCTTCAACTCCCAAAGCTGATATTTCTTACTTAAACTATTCCCTG-

--------------------------------------------------

--------------------------------------------------

--------------------------------------------------

--------------------------------------------------

--------------------------------------------------

--------------------------------------------------

--------------------------------------------------

--------------------------------------------------

--------------------------------------------------

--------------------------------------------------

--------------------------------------------------

--------------------------------------------------

--------------------------------------------------

--------------------------------------------------

--------------------------------------------------

--------------------------------------------------

--------------------------------------------------

--------------------------------------------------

--------------------------------------------------

--------------------------------------------------

--------------------------------------------------

--------------------------------------------------

--------------------------------------------------

------

>Spermophilus_citellus Spermophilus citellus mitochondrion, complete genome.

GTTAATGTAGCTTAA--CT----CAACAAAGCAAAGCACTGAAAATGCTT

AGATGGGTGTTCC-C-ACCCCATAAACATATA--GGTTTGGTCCTAGCCT

TTCCATTAGCTTTTAGCTAACTTATACATGCAAGAATCCCCGCTCCAGTG

AGAATGCCCT-CTATATCCATTAAT---TGATCAAAAGGTGCAGGCATCA

AGTTCACT-AGTTTCAGTAGCTCACAACGCCTTGC-TCCACCACACCCCC

ACGGGATACAGCAGTAATTAAAATTAAGCCTATAAACGAAAGTTTGACTA

AGTTAAGCTA--TAATTAGGGTTGGTAAATTTCGTGCCAGCCACCGCGGT

CATACGATTAACCCTAGTTAATGAAAT-AC-GGCGTAAAGCGTGATTAAG

AGA------CTAAT-TTAATAAGATTAAAACACTATTAAACCGTAAAAAG

TC-TTGATAGTAATGAAAATCAAATACGAAAGTAATCTTA---AATTTTC

TGAATTCACGATAGCTAAGACCCAAACTGGGATTAGATACCCCACTATAC

TTAGCCCTAAACATAAACA-TTTAAC-AAACAAAAGTGTTCGCCAGAGTA

CTACTAGCAATAGCCTAAAACTCAAAGGACTTGGCGGTGCTTTAAACCCC

ACTAGAGGAGCCTGTTCTATAATCGATAAACCCCGATACACCTCACCACC

TTTAGCATATATCAGCCTATATACCGCCATCTGCAGCAAACCCTAA-AAA

GGCCTTACAGTAAGCAAGAAAATTTT--ACATTAAAACGTTA-GGTCAAG

GTGTAGCCTATAAGGTGGG-AAGAAATGGGCTACATTTTCTACCCCCCTA

GAACA------AATTCCTACGATAGCTTTTATGAAACTCAA---AGCATA

AGGCGGATTTAGTAGTAAGTT-AAGAATAGAGAGCTTAACTGAATTG-GG

CAATAAAGCACGCACACACCGCCCGTCACCCTCTTCAAATATACCA----

ACACAAAACTATAAATAATTTACTTAACCACACAT--ATATA--AGAAGA

GACAAGTCGTAACAAGGTAAACATACTGGAAAGTGTGTTTGGAAGAATCA

AAATGTAGCTTA--TTAATAAAAGCACCCGGCTTACACCCGAGAGATTTC

ATTC-ACCATGGACATTTTGA-ACT-AATGCT-AGCCCAACTTTTT----

-TCCTTCCCAAATACAACTTACTCAATAAATAAAACATTTACC-TA-AA-

--TAAA-GTATAGGAGATAGAAATTTA--TATAA--GGAGCTATAGAGAA

AGTACCGTAAGGGAAAGA-TGAAAGAATAAATTTATAGTACTAAAAAGCA

AAGATTAACTCTTCTACCTTTTGCATAA-TGATTTAACCAGAAAATACTT

GACAAAAAG-AATTTAAGCCAAACACCCCGAAACCAGACGAGCTACTTAT

GAGCAGCC--AATAAGAGCCTATCCGTCTATGTTGCAAAATAGTGGAAAG

ACTTATAAGTAGAGGTGAAAAGCCTATCGAGCCTGGTAATAGCTGGTTAT

CCA-GACTAGAATTTTAGTTCTACTTTAAATTTACCTAAAGCATA-AAT-

AA-GCCAAATGTAAATTTAAATGTTATTCTAAAGAGGGACAGCTCTTTAG

AGTCAAGGAAAAAACCTTCATTAGAGAGTAA-----CCCTATTAACT--T

CCATAGTTGGCTTAAAAGCAGCCATCAATTAAAAAAGCGTTAAAGCTTAA

CCTAATTA-C---CAAAACTTAATACCTAACTCT---TCCTATAAACT--

CCTAAATCAACA-CTGGATTAATCTATTTAGTAAT-AGAAGAAACTATGT

TAAAATAAGTAACAAGAA-AATAT-T-CTCCCCGCATAAGCTTATATCAG

ATCGAA-TAATTCGCTGATAGTTAACAACCTCATA-----ATATTAAATA

ACTTATAAATCCATTATTATTTACATTGTTAACCCAACACTGGCATGCA-

--TAAAGGGAAAGATTAAACAAAGTAAAAGGAACTCGGCAAACACTAACC

TCGCCTGTTTACCAAAAACATCACCTCTAGCATAAATAGTATTAGAGGCA

CTGCCTGCCCAGTGACA-TACG----TTCAACGGCCGCGGTATCCTGACC

GTGCAAAGGTAGCATAATCACTTGTTCTTTAAATAAGGACTAGTATGAAT

GGCTTGACGAGGGTTTAACTGTCTCTTACTTTTAATCAGTGAAATTGACC

TTCCCGTGAAGAGGCAGGAATTTTTCAATAAGACGAGAAGACCCTATGGA

GCTTTAATTTTAT-AGTCTCACAAACTTAATAGAATCCA----------A

GGAAATAA-AATTATTGTCTA-TAGACTAGAAATTTTGGTTGGGGTGACC

TCGGAGTACAAATAAGCCTCCGAATGATA-ATAATCT-AGACATAACATG

TCAAAATCATAATTC----ATTAATTGACCCAA-A---TTATTGATCAAC

GGAATAAGTTACCCTAGGGATAACAGCGCAATCCTACTCAAGAGTCCATA

TCGACAGTTAGGGTTTACGACCTCGATGTTGGATCAGGACATCCAAATGG

TGTAACCGCTATTAATG-GTTCGTTTGTTCAACGATTAA-AGTCCTACGT

GATCTGAGTTCAGACCGGAGAAATCCAGGTCGGTTTCTATCTATTATTA-

CATTTCTCCCAGTACGAAAGGACAAGAGAAATAAGGCCAATTAAACAC-T

TATGCCTTAAATT-AATGGATGAA-ATTATCTTAATCCAATAAAATGTTT

TAATT--AACCCGCCCTAGAAAAGGG--CTT-GTTAAGATGGCAGAGCCT

GGTAA-TTGCGTAAGACTTAAAACTTTATT-CCAGAGGTTCAACTCCTCT

TCTTAACA-CTT--------------------------------------

------------------------------------------ATGTTTAT

AATCAATCTCCTACTCCTAATTATTCCAATCCTAGTAGCCATAGCCTTTC

TTACCCTAATTGAACGAAAGATACTAGGATATATACAACTCCGTAAAGGC

CCTAATGTTGTTGGACCCTACGGCCTACTTCAACCATTCGCTGATGCAAT

AAAACTATTTATTAAAGAACCCACAAAACCCCTTACATCATCAATCATAC

TATTTATTATTGCCCCAACCCTAGCCCTAACACTAGCATTTACCATATGG

ATTCCTCTACCCATACCCATACCCCTCATTAATATAAATATAGGAGTCCT

ATTCATCCTAGCCACCTCAAGTTTAGCCGTATATGCAATTCTATGATCTG

GGTGAGCTTCCAACTCTAAGTATGCTTTAATCGGAGCTCTACGAGCTGTA

GCACAAACCATTTCATATGAAGTAACTCTAGCAATTATTCTTCTCTCAGT

ACTTCTAATAAATGGATCCTTTACCTTATCTACACTAATTACCACTCAAC

AATTCACGTGACTCCTACTCCCAACATGACCTCTAGCAATAATGTGATTT

ATCTCAACATTAGCAGAAACCAACCGAGCTCCATTTGACCTAACAGAAGG

AGAATCAGAACTTGTATCAGGATTTAATGTTGAATATGCAGCTGGTCCCT

TTGCCTTATTCTTTATAGCTGAATACACCAACATTATCATAATAAACGCA

TTAACAGTAACCCTTTTTATAGGAGCATTACTCAACCCAATCTCTCCTGA

AACCTTCACATTAAACTTTACCTTAAAAACACTCATCTTAACCTCTACCT

TTCTATGAATTCGAGCATCTTACCCTCGATTTCGTTATGACCAACTCATA

CATCTTTTATGAAAAAACTTTCTACCTCTAACCCTAGCTCTATGCATGTG

ACATATCTCTCTTCCAATCATAACTGCATGCGTACCACCCCAAATCTAA-

GAAATATGTCTGATAAAAGAGTTACTTTGATAGAGTAAATTATAGAGGTT

CA-AACCCTCTTATTTCTAGAACTATAGGGATTGAACCTAATCCTAAGAA

TCCAAAATTCCTCGTGCTACC-TCT-TACACCATGTCCTAAAT--AAATA

GTAAGGTCAGCTAAAT-AAGCTATCGGGCCCATACCCCGAAAATGTTGGT

TTATATCCTTCCCGTACTAATTAACCCCTTAACTTCCTCCACAATCTACC

TCACTCTATTTTCTGGAACTATAATTACACTTTTCAGCTCACATTGACTT

CTAATCTGAATAGGCCTAGAAATAAGTATACTAGCCATTATCCCCATTTT

AATTAATAAAGGAAATCCCCGATCTACAGAAGCTGCATGTAAATATTTCC

TCATTCAAGCCACCGCATCAATAATTCTAATAATAAGTACAATAATTAAT

TTCATAGACTCAGGCCAATGAATCTTATCTAATTCATACAATCAAATCTC

ATCATTTATATTTACAATTGCACTTTCCATAAAAATAGGACTTGCTCCAT

TTCACCTATGAGTTCCAGAAGTTACCCAAGGAATCTCCCTTAAATCAGGC

CTAATTATGCTAACATGACAAAAAATTGCCCCAATTTCTATCGTATATCA

AATTGCACCCTCTATAAACTCTACCCTTATATTATTTATAGGAATTCTAT

CAATCATATTAGGAGGCTGAGGAGGACTTAACCAAACCCAACTACGAAAA

ATTCTAGCATATTCATCAATCGCCCATATAGGATGAATAATAGCAATTAT

TACATATAACCCAACCTTAGCAATATTTAACCTAATCATTTATATTATAC

TTACCACTAGCATATTCATACTTCTCCTTTTATACAAAAAAACTACTACC

CTTTCCCTATCTAATCTATGAAATAAATCCCCTCTTCTAACACCTATAAT

TTTAATTGTATTAATATCACTAGGAGGACTACCTCCCCTAACAGGATTTA

CACCAAAATGAATTATCCTTAAAGAACTTATCTCAAATAACAGCATTATT

TCCTCTACATTAATAGCAATATTAGCACTTCTAAATTTATACTTCTATAC

ACGACTTATTTATTCAACATCTCTAACTTTATTCCCATCATTCAATAATA

CTAAAATAAAATGACAATTCGAAAGCATAAAACTTACACCTTTTCTATCT

ATCCTTGTTATCACCTCTACCCTCTCCCTCCCATTATTTCCCCTT-CTCT

CACTCCT--GAACT----AGGAATTTAGGTTAAC--T-CAGACCAAGGAC

CTTCAAAGTCCTAAGCAAGTA-CCCAAT---ACTTAATTCCTGT------

---A----CTAAGGACTGCAAGACTCTATCTTACAT----CAATTGAATG

CAAACCAATCACTTTAA--TT-AAGCTAAGCCCTTCTATT------CCTA

GACTGATG--GGATTTAAA-CCCAT-AAGACCTTAGTTAACAGCTAAACG

CCTTATTCAACTGGCTTCAATCTA-CT-TCTCCCGCCGT-AAGTAAAAAA

-----GGCGGGAGAAGCCCCGGCAGAG-T-TGA-AGCTGCTCCTTT--GA

-----------------------------ATTTGCAATTCAATA--TGAC

TA-TTCACCTCAGGACT-T-----GGTAGAAAGAGGG----TT-CAACCT

CTGTCTTTAGATTTACAGTCTAATGCTTA--CTCAGCCATTCTACC----

ACCTACTTATGTTCATCAACCGTTGATTCTTCTCAACTAACCACAAAGAT

ATTGGTACACTCTACCTTCTATTTGGAGCCTGAGCCGGAATAGTAGGAAC

TGCACTTAGCCTACTAATTCGAGCCGAATTAGGACAACCTGGAGCTCTAT

TAGGCGATGACCAAATTTATAATGTTATCGTCACAGCCCACGCATTTGTC

ATAATTTTCTTTATAGTAATACCAATTATGATTGGTGGGTTCGGAAACTG

ATTAGTACCCCTAATAATTGGAGCCCCTGATATAGCATTTCCACGTATAA

ATAATATAAGCTTCTGACTTCTTCCCCCTTCTTTTCTTCTCTTACTTGCC

TCTTCTATAGTTGAAGCAGGTGCAGGAACCGGTTGAACCGTATATCCCCC

TCTAGCTGGAAATCTTGCCCATGCAGGAGCTTCAGTTGACCTAACCATCT

TTTCCCTTCATTTAGCAGGAGTATCATCAATTCTAGGTGCGATTAACTTT

ATTACAACAATTATTAACATAAAACCACCTGCCATGTCTCAATATCAAAC

CCCTCTGTTTGTATGATCGGTATTAATTACAGCAGTCCTATTACTCCTGT

CTCTTCCGGTTCTTGCAGCAGGAATTACTATACTACTCACAGATCGAAAC

CTTAATACTACATTTTTCGATCCTGCTGGGGGTGGAGATCCTATCCTCTA

TCAACACTTATTCTGATTTTTTGGACACCCTGAAGTTTATATTCTAATTC

TCCCAGGATTTGGCATAATTTCTCATATCGTAACATATTACTCAGGAAAA

AAAGAACCATTCGGTTATATAGGAATAGTATGAGCTATAATATCTATCGG

TTTCCTTGGGTTCATTGTATGAGCTCATCACATGTTTACCGTTGGAATAG

ACGTAGACACTCGAGCTTACTTTACATCCGCAACTATAATTATCGCTATC

CCCACAGGAGTTAAAGTCTTTAGCTGACTGGCAACCTTGCATGGAGGAAA

TATTAAATGGTCACCAGCAATACTATGAGCACTAGGCTTTATTTTTCTGT

TTACTGTAGGAGGTCTAACAGGAATTGTCTTAGCTAATTCTTCATTAGAC

ATTGTCTTACACGATACATATTATGTAGTAGCTCATTTTCACTATGTACT

ATCAATAGGAGCTGTATTTGCAATTATAGGAGGATTCGTTCATTGATTCC

CCCTTTTTTCTGGTTATACATTAAATGATCTATGAGCTAAAATCCATTTT

ACTGTAATGTTTGTTGGAGTAAACTTAACTTTCTTCCCCCAACACTTCTT

AGGGTTATCAGGTATACCACGTCGATACTCTGACTATCCAGATGCATATA

CAGCATGAAATACTGTTTCCTCAATGGGTTCATTTATCTCCCTTACAGCT

GTTATAATTATAATCTTTATAATTTGAGAAGCATTTGCATCAAAACGAGA

AGTCTCTACCGTAGAACTAACACCAACTAACTTAGAATGACTACATGGAT

GTCCTCCACCTTACCACACATTTGAAGAACCTACTTATATTAAAGCCTAG

AT--C--------------AAGAAAGGGAGGAATCGAACTTCCTAAAACT

AGTTTCAAGCTAGCCCCATAACCATTATGA-CTTTCT---TCAT----GA

GATATTAGTAAAAT-AATTACATAACTTTGTCAAAGTTAATTTATAGGTT

A------AATTCCTATATATCTC-TATGGCATATCCACTCGAATTAGGAT

TTCAAGACGCCACATCTCCCATTATAGAAGAACTTTTACATTTTCACGAT

CATACTCTTATAATCGTTTTCTTAATTAGCTCTCTAGTTCTTTATATTAT

CTCATTAATATTAACTACAAAATTAACTCATACAAGCACTATAGATGCTC

AAGAAGTAGAAACTATTTGAACTATTCTTCCCGCTATTATCCTTATTCTA

ATTGCCCTTCCCTCTCTACGCATTCTATATATAATAGACGAAATTAATGA

TCCATCCTTAACAGTTAAAACAATAGGCCATCAATGATACTGAAGTTACG

AATATACAGACTATGAAGACCTCAATTTTGATTCTTATATGATCCCAACT

TCAGATTTAGCCCCAGGAGACCTACGACTTCTAGAGGTCGATAATCGAGT

TGTTCTTCCAATAGAATTGCCTGTACGAATATTAATCTCATCTGAAGACG

TACTTCACTCTTGAGCAGTCCCATCCCTTGGACTAAAAACGGATGCAATC

CCAGGTCGACTTAATCAAGCTACACTTACATCAACACGACCCGGACTTTA

TTATGGACAATGCTCCGAAATTTGTGGATCAAATCATAGCTTTATACCAA

TTGTCCTCGAGCTAGTCCCATTAAAACACTTTGAAAACTGATCTTCATCA

ATGCTATAA--------------------------------ATT-CATTA

TGAAGCTA--TAGTAGCATCAACCTTTTAAGTTGAAGATTAGGAGC-C-A

A-AT-CTCCTCATGATGAAATGCCCCAACTAGATACATCTACATGATTTA

TTACAATTCTATCAA-TAATTCTAGCTCTCTTCTTTATATTTCAACTTAA

AATTTCAAATCA--CTCTTACCCATCTAATCCTACCCCTAAAGACACTAA

-ACTAATTGAGCAAAAAACTCCTTGAGAAGAAAAATGAACGAAAATCTAT

TTGCCTCTTTCATTACCCCTACATTAATAGGTCTTCCCATTGTTCTCTTA

ATTATTATATTTCCTAATCTACTTTTCCCTTCACCCTCTCGACTAGTAAA

TAACCGTCTAGTATCTTTCCAACAATGACTTATTCAACTTGTACT-AAAA

CAAATAATGGCCATACACAACCCAAAAGGACGTACCTGATCCCTAATACT

AATCTCATTAATTATATTTATCGGCTCAACCAATCTTCTAGGTTTATTAC

CCCACTCTTTTACACCAACAACTCAGCTATCAATAAATTTAGGAATAGCT

ATTCCTCTTTGAGCAGGAGCAGTAATTACTGGATTCCGTCACAAAA-CTA

AAGCATCATTAGCCCACTTCCTTCCACAAGGAACCCCAATTCCACTTATT

CCAATACTTATTATTATCGAAACAATTAGCCTCTTTATTCAACCCATAGC

ATTAGCTGTACGACTTACAGCTAATATTACAGCTGGTCATCTTCTTATAC

ACTTAATCGGAGGGGCAACCCTTGTATTAATATCTATTAGTCCTCCTACA

GCTATTTTAACTTTTATTATTCTCGTACTACTAACAATACTCGAATTTGC

AGTTGCATTAATTCAAGCTTACGTCTTCACCCTTTTAGTAAGTTTATATT

TACATGATAATACCTAATGACCCACCAAACCCATGCCTATCATATAGTTA

ATCCCAGTCCTTGACCCTTAACAGGAGCCCTCTCCGCTTTACTCCTAACT

TCTGGCTTAGTAATATGATTCCACTTCAACTCCTCCCTTCTCCTTATACT

AGGTCTAACAGCTAACACCTTAACAATATACCAATGATGACGAGACGTTG

TACGAGAAGGCACATTTCAAGGTCACCATACATCAATTGTCCAAAAAGGT

TTACGATATGGTATAGTATTATTCATTATTTCAGAAGTATTCTTCTTCGC

TGGATTTTTCTGAGCATTCTATCATTCTAGTCTAGCTCCAACTCCTGAAC

TTGGCAGTTGCTGACCTCCAGTAGGAATTAATCCACTTAATCCCCTAGAA

GTACCACTATTAAATACTTCTGTTCTTCTAGCTTCAGGAGTATCAATTAC

CTGAGCTCATCACAGCTTAATAGAAGGAGACCGAAAACACATAGTCCAAG

CATTATCAATTACAATTGCTCTAGGACTTTATTTTACCCTTCTTCAAGCT

TCTGAGTATCTAGAGACATCTTTTACAATTTCAGATGGTGTATATGGCTC

AACATTCTTTATAGCCACAGGTTTCCATGGCCTTCATGTCATTATTGGAT

CAACCTTCCTTTTAGTATGTCTCATTCGTCAACTAAACTTTCACTTTACA

TCAAACCACCATTTTGGATTTGAAGCAGCCGCATGATATTGACATTTCGT

AGATGTCGTATGACTCTTCCTTTACGTATCTATTTATTGATGAGGTTCAT

ATTCT-CTTAGTATTAA-TTAGTACAATTGACTTCCAATCAATTAGTTCT

GGAATTAACCCAGAAGAGAATAATAAACCTTATAATATCTTTCCTCACAA

ACTCCTTTATCGCTTTACTACTAATCTCTGTAGCATTTTGATTACCTCAA

CTAAACGTAAATGCCGAAAAAGCAAGCCCCTACGAATGTGGCTTTGATCC

CATAGGATCTGCTCGCTTACCATTCTCTATGAAATTTTTTCTCGTTGCAA

TTACATTCCTTTTATTTGATTTAGAAATTGCTCTTCTTCTCCCCCTTCCC

TGAGCCTCCCAAACAAATAATCTTAACCTTATATTAACTATAGCTTTACT

CTTAGTCTCAATTCTTACCCTCGGATTAGCCTATGAATGAATTCAAAAGG

GCTTAGAATGAATTGAATA------TGATAATTAGTTTAAAATAAAA-CA

AGTGATTTCGACTCACTAAATTATGAACTC-T--CATAATTATCAA--AA

TGCCTATTATTATTCTCAATACTATTTTAGCATACTCTGTATCCTTGCTA

GGAATGTACATCTACCGATCCCACCTAATATCATCACTTCTATGCTTAGA

GGGTATGATACTATCAATATTTGTTTTATGCTCGCTTCTAGTTATAAATT

TCCACTTCTCCTTATCATTTATAATTCCTATTACTCTATTAGTATTTGCT

GCGTGTGAAGCAGCTGTAGGCCTAGCCCTTCTAGTAATAGTATCTAACAC

ATACGGCCTGGATTACGTTCAAAATTTAAATATTCTCCAATGCTAAAAAT

TATTATCCCCACAATCTTACTTGCTCCCCTTGTATGATTCTCAAAACCAT

CCATAATCTGAATCAACCCCTCAATTCATAGCTTAATAATTAGCCTAATT

GTCCTCCTTGCACTAAGTCACTCTACAAACATAAATTCAACTTTTTCATT

ATCTTTCTTTACCGACCCCTTATCTTCTCCTCTATTAATTTTAACAGCAT

GACTTCTACCTCTTATAATTATAGCAAGTCAAAATCACCTAGCCCAAGAA

CCGTTGATCCGAAAAAAACTATACATTCTTATACTAATCTCACTACAATC

CTTTTTAATTATAACTTTCTCCGCTACTGAATTAATTATATTTTACATTT

TATTCGAGGCTACCTTAATCCCTACACTAATTATTATTACCCGATGAGGA

AACCAAGCTGAACGATTAAATGCAGGACTATATTTTCTATTTTATACTCT

TGTAGGCTCTCTACCTCTATTAGTAGCATTAATTTTTATTCAAAAATCCA

CAGGATCTTTAAATTTTATCATTTCAATATATCAGTCATTTAATCTTCCC

ATATCT----------TGAACAAATGATATTATATGATTGGCATGCATTA

TAGCCTTTATAGTTAAAATACCCCTATATGGCCTTCATCTCTGACTACCA

AAAGCTCATGTAGAAGCTCCCATTGCTGGTTCTATAGTCTTAGCTGCTAT

CTTACTAAAACTAGGTGGGTATGGAATAATTCGAATTTCAACTTTTCTAC

ATCCCATTACATGCAACATAGCATATCCCTTTATTATATTATCATTATGA

GGTATAATCATAACAAGCTCAATTTGCTTACGACAAACAGACCTAAAATC

CCTCATCGCTTACTCATCAGTAAGTCACATAGCACTAGTAATTGTAGCAA

TTATGATTCAAACTCCCTGAAGCTTTATAGGAGCTACAGCACTAATAATT

GCCCACGGATTAACATCTTCCATATTATTCTGTCTAGCAAACACTAACTA

TGAACGAATTCACAGCCGAACTATAACACTGGCCCGAGGCTTACAATCTA

TTCTTCCCCTTATAGCAACATGATGAATTCTAGCTACTTTAACCAACCTA

GCTCTTCCACCCTCTATCAATCTAATTGGTGAATTATTCATTATTATAGC

ATCATTCACTTGATCAAATGCTACAATTATCCTAACCGGACTAAATATGT

TAATTACAGCTCTTTATTCATTATATATACTAATTATAACACAACGAGGA

AAATTTACGTACCACACATTAAATATTAACCCTTCCTTCACACGAGAAAA

CACACTCATATTTCTTCACTTTTTTCCACTTGCCATCCTATCAACAAACC

CAACTATTATCCTGGGTCATTTATACTGTAAATATAGTTTAATAAAAACT

TTAGATTGTGAATCTAAAAATAGGGAATTATAA--TCTCTTATTTACCAA

GAAAGCAT---GCAAGAACTGCTAATTCATGC-TCCCGTGTTTACATACA

CGGCTT--------------------------------------------

-------------------TCTTA-ACTTTTATAGGATAGAAGTA-ATCC

ATTGGTCTTAGGAACCAAAAA-A-TTGGTGCAACTCCAAATAAAAGTAAT

TAATA---TATTCTCTTCACTCATTATTACATCACTTACAATCCTCTCAT

TCCCCATTCTCCTTACAATAACTAACTATCATAAGCATATTAACTATCCA

AACTATGTAAAAACTTCTATTATTTGTGCATTATCATTCTGCATTGTACC

AACACTAATGTTCATTAACTCTAACTACGAAACCGTTATCTCAAACTGAC

ACTGAATGACTATTCAAACATTTACCTTTTCTATAAGCTTTAAACTAGAT

TATTTTTCTATGCTATTTATTCCCGTAGCATTACTTGTTACATGATCAAT

CACAGAATTCTCAATATGATACATGCACTCTGATCCATTCATTAATCGTT

TTTTCAAGTATCTCCTCCTATTTCTTATCACTATATTAATTTTAGTTACA

TCCAACAATCTATTTCAACTATTCATCGGCTGAGAAGGAGTAGGTATTAT

ATCTTTCTTATTAATTGGCTGATGATATGGCCGAACAGACGCTAATACAG

CAGCCCTTCAAGCCGTCCTATATAATCGAATTGGAGATATTGGATTTGTT

TTAGCTATAGCATGATTCTTACTTAACTCAAACTCATGAGAACTACAACA

ACTTTTCATAATAAATGTATCACTA------CTTCCTCTATTAGGCCTAC

TCTTAGCCGCCGCAGGAAAATCCGCCCAATTCGGCCTCCATCCTTGACTA

CCTTCCGCTATAGAAGGTCCAACCCCTGTATCAGCTTTACTCCACTCAAG

TACAATAGTAGTAGCAGGAGTTTTTCTCCTTATTCGTTTTCATCCACTAA

TA-GAACATAATAAGACTATTCAAACACTTACTCTCTGCTTAGGAGCTAT

AACCACCTTATTTACCGCTATCTGCGCCCTCACCCAAAATGACATTAAAA

AAATTATTGCATTCTCCACTTCCAGTCAATTAGGATTAATAATAGTAACC

ATCGGAATTAATCAACCCTACCTAGCTTTCCTTCATATTTGCACACATGC

ATTCTTTAAAGCTATATTATTTATATGCTCAGGATCAATTATTCATAATC

TAAATAATGAACAAGATATTCGAAAAATAGGAGGTCTATTTAAAGCTCTC

CCATTCACCTCATCTTCACTTATCATTGGCAGCCTAGCATTAACAGGAAT

TCCCTTCTTAACCGGATTTTATTCCAAAGACCTAATTATTGAAGCTATAA

ACACGTCATATACCAACGCCTGAGCCCTAACTATTACTCTTATTGCCACT

TCCTTAACCGCTGTCTACAGTACACGAATTATTTTTTATGTCCTAATAGG

ACAACCTCGATTCTCTACACTAACCTCAATTAATGAAAATAACCCCTATT

TACTTAACTCAATTAAGCGTCTTCTTATTGGAAGCATTATTGCAGGATTT

ATCCTTTCATACAACATCCCACCTATAAACGTCCCAACTTTAACTATACC

TCTGTATCTAAAAATTACAGCGCTGTTAGTAACTATTTTAGGATTTGTTA

TTGCCATAGAACTTAACTTAATAACTCTATATCTCAAAACCAAAATATAC

TCAAACACAACA-AAATTCTCAACTCTATTAGGCTATTTTCCTATTATTA

TCCACCGACTTAACCCCTATCTTAACCTTACTATAAGCCAAAAACTCTCA

TCAACCCTATTAGATTTAGTCTGACTAGAAAAAACCATTCCCAAATTTAC

CACCAACTTCCAC---TCAACAGCCTCTACTATAACCTCCAATCAAAAAG

GCCTAATCAAATTATATTTCTTATCATTTTTAACCTCAACACTCCTAGCA

ACTATAACCGTATTCTA---------------------------TTTCCA

CGCGTAATCTCAATCACAATAAAAATACTAACAAACAAGGATCAACCAGC

CAAAACCATCAATCAACTTCCATAATTATACATAGCTGCCACCCCCATTG

AATCTTCACGAACCAACTCCAATTCACTTTCCTCAAATACTATTCAATTC

CCCGAATCCTTAGATTCAATTACAATATCTA---------TTTCATCATA

TAAAACTATAAATATAATAATTAAAAACTCTACCAAAAATCCTAATAACA

AAACCCCTCAAATAACCACATTTGATCCCCATGTTTCTGGGTATTCCTCC

GTTGCTATAGCCGTAGTATAACCAAACACTACTAATATTCCCCCTAAATA

AATTAAAAACATTATTAAACCTAAAAAAGACCCTCCAAAATATAGTACAA

TTCCACACCCAATTCCCCCACTAATAATTAACCCCAAACCCCCATAAATA

GGAGAAGGCTTTGAAGAAAACCCCACAAAACCTAAAACAAAAATCATACT

TAATAAATATATAATATATGTCATTAT---TTTTA-CATGGAATCTA-AC

CATGACCAATGACATGAAAAATCATCGTTGT-TATTCAACTATAAAAACT

-TT----AATGACAAACACTCGTAAAACCCACCCTTTAATTAAAATTATT

AACCACTCTTTCATTGATTTACCAGCACCCTCCAACATCTCCGCATGATG

AAACTTTGGATCCCTACTAGGCCTATGTCTAATTATTCAAATCCTTACTG

GGTTATTTTTAGCAATACACTATACATCAGATACTATAACAGCCTTTTCA

TCAGTTACTCACATCTGCCGAGATGTAAACTACGGCTGACTTATTCGATA

CATACATGCTAATGGTGCATCTATATTCTTTATCTGCCTCTTTCTTCATG

TAGGCCGAGGGTTATATTACGGCTCATACACCTACTTTGAAACATGAAAT

ATTGGAATTATTCTCCTATTTGCAGTAATAGCTACAGCTTTCATAGGCTA

TGTTCTTCCCTGAGGCCAAATATCATTCTGAGGGGCAACCGTAATTACCA

ATCTTTTATCTGCCATCCCATACATTGGTACAACCTTAGTAGAATGAATC

TGAGGTGGCTTTTCAGTAGATAAAGCTACTCTAACACGATTTTTCGCATT

CCATTTTATCCTTCCATTTATTATCACAGCTCTAGTCATAATTCACCTCC

TTTTTCTCCATGAAACTGGGTCAAATAACCCTTCAGGCCTCATTTCTGAT

TCAGACAAAATCCCATTTCACCCATATTATACAATTAAAGATATCCTTGG

AATTCTCCTTCTTATCCTAACCCTAATAATGCTAGTCCTATTTTCACCCG

ACCTTCTAGGAGACCCCGATAATTATATACCCGCAAACCCCCTAAGCACT

CCACCCCATATTAAACCAGAATGATATTTCTTATTTGCCTACGCTATTCT

CCGATCTATCCCTAACAAACTAGGAGGTGTTTTAGCCCTAATTTTCTCAA

TTCTTATCCTAATACTCTTTCCACTACTCCACCTGTCTAAACAACGTAGC

ATGATATTTCGACCAATAAGTCAATGTGTATTCTGAATTCTAGTGGCAGA

CTTGCTTACACTAACATGAATTGGAGGACAACCTGTTGAACACCCATTTG

TTATCATTGGCCAACTGGCATCAATCCTATATTTTGCTATCATTCTCTTA

ATTTTACCAACTGTTAGCATAATCGAAAACAAACTTCTCAAATGAAGA--

--GCCCTAATAGTATAA-AT--ATTACTTTGGTCTTGTAAACCAAAAATG

AAGT--ATAAAC--TTCTTAGAGCAAT--ATCAGGGAAGAAA-TAAACTT

TCCACCTTCAACTCCCAAAGCTGATATT-CTTACTTAAACTATTCCCTG-

--------------------------------------------------

--------------------------------------------------

--------------------------------------------------

--------------------------------------------------

--------------------------------------------------

--------------------------------------------------

--------------------------------------------------

--------------------------------------------------

--------------------------------------------------

--------------------------------------------------

--------------------------------------------------

--------------------------------------------------

--------------------------------------------------

--------------------------------------------------

--------------------------------------------------

--------------------------------------------------

--------------------------------------------------

--------------------------------------------------

--------------------------------------------------

--------------------------------------------------

--------------------------------------------------

--------------------------------------------------

--------------------------------------------------

------

>Muscardinus_avellanarius_DEN Muscardinus avellanarius isolate DM333 mitochondrion, completegenome.

GTTAATGTAGCTTAA--C------TA-AAAGCAAAGCACTGAAAATGCTT

AGATGGGTACTCT-T-ACCCCATAAACATAAA--GGTTTGGTCCCAGCCT

TCTTATTAATTTATAACAAGATTACACATGCAAGCATCTACGACCCTGTG

AGAATGCCCT-CTATGTTAACAAATATTTAACCTAAAGGAGCAGGTATCA

AGCACAC----TATTAGTAGCTCAAAACACCTTGC-TTAACCACACCCCC

ACGGGAAACAGCAGTGATTAAAATTAAG-ACATAAACGAAAGTTTGACTA

AACTATGTTA-TAAA-TAGGGTTGGTAAATTTCGTGCCAGCCACCGCGGT

CATACGATTAACCCAAATTAATAAGTC-AC-GGCGTAAAGAGTGTTTTAG

A----TTATTAC--CTTAATAAAGTTAAACTTTAACTAAGCTGTAAAAAG

CC-CTAGTCAAAAGTAAAGTAATGAACGAAAGTCACTTTAGTAT--TTTC

TGAATACACGATAGCTAAGACACAAACTGGGATTAGATACCCCACTATGC

TTAGCCCTAAACATAAACT-CTTATT--AACCTAATTGTTCGCCAGAGAA

CTACAAGCCAAAGCTAAAAACTCAAAGGACTTGGCGGTGCTTTATATCCC

TCTAGAGGAGCCTGTTCTATAATCGATAAACCCCGATATACCTCACCATT

TCTTGT-CACCACAGCCTATATACCGCCATCTTCAGCAAACCTTAA-CAA

GGAGTAAAAGTAAGCTCAAGCACTG---ACATAAAAACGTTA-GGTCAAG

GTGTAGCTTATGAAATGGA-AAGAAATGGGCTACATTT----ATTTCACC

AAATA------CAT--TTACGTTAACTCTTATGAAACCTGA--GAGTGGA

AGGAGGATTTAGTAGTAAGCT-AGGAGTAGAGAGCCTAACTGAATAG-GG

CCATTAAGCACGCACACACCGCCCGTCACCCTCCTCAAGTATCCAACATT

ATATAGATATATACATATACAAGTTTATTTAA-----ATGCA--AGAGGA

GATAAGTCGTAACATGGTAAGCGTACTGGAAAGTGTGCTTGGATTAATCA

AAACGTAGCTTA----AAATAAAGCATCTGGCTTACACCCAAAAGATTTT

ATAT-ATGATAAACGTTTTGA-ACA-AAATCT-AGCCCACTTT---A---

ATTTAATACAATTAACAAAAATCAATAAAATAAACCATTCAT---A-AC-

--TTAAAGTATAGGAGATAGAAATTTTA-TATTT--GGCGCTATAGAGAC

AGTACCGTAAGGGAAAGA-TGAAAGATA--ACTTTAAGTAAATGAAAGCA

AAGATTAAACCTTGTACCTTTTGCATAA-TGAATTAACTAGAATATGCCT

AGCATAAAGCAATTAAAGTTAGCTACCCCGAAACCAGACGAGCTACATAT

GAGCAGCT--AACTTGAGCCAACCCGTCTGTGTAGCAAAACAGTGGGAAG

ACTTATATGTAGAGGTGAAAAGCCTATCGAGCCTGGAGATAGCTGGTTAC

CCAAG-TAAGAATTTCAGTTCAACTTTAATCTTACCTAAAGAATA-AA-G

AA-TCCACTTGTAAGATTAAATTATATTCTAAAGAGGGACAGCTCTTTA-

GAT-AAGGAAATAACCTGATTTAGAGAGTAA-----ATTATTACTTCATA

CCACAGTTGGCTTAAGAGCAGCCATCAGTTAAGAAAGCGTTCAAGCTCAA

C---AT--ATCCATTCTTCTTAAT-TTTAATAGA---CTAGTTCAACT--

CCTATATCCTCAATTGGGTTAATCTATTACATAAT-AGAAGCAATAATGT

TAATATGAGTAACAAGAA-AAATT-TTCTCCTAGCATAAACTTATATCAA

TTCTAA-TA--TCATTGATAGTTAACATTATAATA-----TTATTATACA

AACTAATAATC--ATATTTATTATTATGTTAATCCAACACAGGAATGCAC

CACTCAAGGAAAGATTAAAAGAAGTAAAAGGAACTCGGCAAACATTAACC

CCGCCTGTTTACCAAAAACATCACCTCTAGCATCTCAAGTATTAGAGGCA

CTGCCTGCCCAGTGACA-TATG----TTCAACGGCCGCGGTATCCTGACC

GTGCAAAGGTAGCATAATCATTTGTTCCTTAATTAGGGACTTGTATGAAT

GGCTTAACGAGGGTTTAACTGTCTCTTACTTCTAATCAGTGAAATTGACT

TCCCAGTGAAGAGGCTGGGATAATCCAATAAGACGAGAAGACCCTATGGA

GCTTAAATT-AACTAGCTTAATTACCCTATCAACACTTCTGT------A-

GATACATAATGCAAAGTATTAATAAGCTACCAATTTCGGTTGGGGTGACC

TCGGAGAACAAATTAACCTCCGAAAGATA-AAAACTAGACAAACCAGTCA

AATCTATCAAATTTA----TCATATTGACCCAA-AA--TACTTGATCAAC

GGAACAAGTTACCCTAGGGATAACAGCGCAATCCTATTTTAGAGTTCATA

TCGACAAT-AGGGTTTACGACCTCGATGTTGGATCAGGACATCCTAATGG

TGCAACCGCTATTAAAG-GTTCGTTTGTTCAACGATTAATAGTCCTACGT

GATCTGAGTTCAGACCGGAGCAATCCAGGTCGGTTTCTATCTATTAACA-

GTTTCTCCC-AGTACGAAAGGACAAGAGAAACAAGGCCAATTATCAATAT

AATGCCTTAATTTTTATAGATGAA-CTATTCTCAATCTAGCATAATTAAC

TCTA------CTACCCTAAATAAGGG---TTCGTTAAGGTGGCAGAGCCC

GGTAAATTGCATAAAACTTAAAACTTTATATTCAGAGATTCAAATTCTCT

CCTTAACATC----------------------------------------

------------------------------------------ATGTTTTT

AATTAATCTTCTTCTCTTAATTATTCCGATTCTTTTAGCCATAGCATTTT

TAACTTTAATCGAACGAAAAATCCTAGGCTATATACAACTCCGCAAAGGT

CCAAACATTATTGGACCCTACGGTCTTCTACAACCTATCGCAGATGCTAT

AAAACTTTTTATTAAAGAACCACTTCGACCTCTATCATCATCAATAAGTC

TATTCATCATCGCACCAACATTAGCCCTCACACTAGCATTCACTATATGA

ATTCCATTACCTATGCCACATCCCCTAATCAACATAAACTTAGGTGTTAT

CTTTATATTAGCCACATCCAGTCTAGCCGTATATTCAATCTTATGATCAG

GATGAGCATCAAACTCAAAATACTCACTAATTGGAGCCCTACGAGCCGTA

GCACAAACCATTTCATATGAAGTTACACTAGCCATCATTCTACTCTCAGT

ACTAATATATAATGGATCATTTACCCTAATAATATTAACACAAACTCAAG

AACATATATGACTAATTTTTCCTACATGACCACTAGCAATGATATGATTT

ATTTCCACACTAGCTGAAACAAATCGCGCACCCTTCGACTTAACCGAAGG

GGAATCAGAATTAGTTTCAGGCTTTAATGTTGAGTATGCAGCAGGCCCAT

TCGCCCTATTCTTCATAGCAGAATATACTAATATTATTATAATAAATGCC

CTAACCTCTACACTATTTCTAGGAGCCCTAAACAACATCTTCTACCCAGA

ACTATTCACGTTAAGCTTTATAACAAAAACCCTCATTCTTACATCTACTT

TTTTATGAATTCGTGCATCATATCCACGATTTCGATACGATCACCTTATA

CATCTCTTATGAAAAAATTTTCTACCACTGACACTGGCCCTCTGCATATG

ACATATCTCATTACCAGTCATTATATCAAATATTCCACCCCAAACCTAA-

GAAATATGTCTGAAAAAAGAGTTACTTTGATAGAGTAAATTATAGAGGTT

TA-AGCCCTCTTATTTCTAGAATAAAAGGAATTGAACCTTAACTTAAGAA

TTCAAAATTCTTCGTGCTACCTAGT-TACACCATATCCTAATT--A---A

GTAAGGTCAGCTAATT-AAGCTATCGGGCCCATACCCCGAATATGTTGGT

TTAAACCCTTCCCGTACTAATTAATCCTATAACTGCTACAGCTATCTACT

TCACCTTATTCTCAGGGACAATAGTTGTTATATTTAGTCTCCATTGACTA

CTTACCTGAATCGGACTGGAAATAAGTATACTAGCTATTATCCCAATTAT

TATCAATAAAGCTAATCCCCGATCAACAGAAGCTGCAACTAAATATTTTC

TAATTCAAGCCACAGCATCCATAATTCTAATAATAGCAATTATCTCAAAT

ATACTTCTTACTGGGCAATGAACAATATATAATTTTTCTAACCCTCTACC

GTCTACATTAGCTACAATTGCCCTCACCATAAAATTAGGCATAAGCCCAT

TTCACTTCTGAGTACCAGAAGTAATCCAAGGAACTACTATTATATCAGGG

CTAGTAATTTTAACATGACAAAAACTCGCCCCAATCTTAATCCTGTACCA

AATCTCTCCTTTTATTAATAAATCATTAATTATAACTATAGCAATTTTAT

CAATTGCCTTAGGTGGTTGAGGAGGTCTTAACCAAACCCAACTACGAAAA

ATTATAGCCTACTCATCCATTGCACATATAGGATGAATAATAGCCATCAT

CACCCTAAATCCATCAATCGCCTCCCTCAACTTAATTATTTATATCATAT

TAACAATTTCTATATTTATAACAATACTTAATAATAATAGCACAACTACA

CTTACTCTATCTACTTTATGAAATTGCATTCCACTAATAACCCTAATTAC

CTTAACAATTCTCATATCACTAGGAGGTTTACCTCCACTAACAGGATTTC

TACCCAAATGAATTATCATTCAAGAATTAATTAAAAATAATATTAATATA

TTATCTTTAATCATGGCTATAATAGCTCTACTAAACCTATACTTTTATAC

ACGACTAATTTACTCTACTTCCCTAACTCTATTCCCATCATCCAACAACA

TAAAAATAAAATGAAAATTTGAATCTATAAAGCTTATTACTCTAATCCCC

TCACTTACCATAATATCAACACTTCTAATACCATTAACACCAATAACT-T

CAATCTT--AAGCT----AGGAATTTAGGTTAAT----TAGACCAATAGC

CTTCAAAGCTTTAAGTAAGTGACCTAA----ACTTAATTCCTGA------

-----TA-ATAAGGATTGCAGATTTAT-TTCTACAT----CATTTGAACG

CAAATCAAAAGCTTTAA--TT-AAGCTAAATCCTTTATCA------CCTA

GGCTGATG--GGAATTCAA-CCCAC-GAAACTTTAGTTAACAGCTAAAAA

CCCTAAACAACTGGCTTCAACCTA-CT-TCTCCCGCCGGGGAGGAAAAAA

A----GGCGGGAGAAGCCCCGGCAG-C-TTCGA-AGCTGCTTCTTT--GA

-----------------------------ATTTGCAATTCAATG--TGAT

TA-ATCACCACAAGACT-T-----GGCAGAAAGAGGT----TA-C-CCCT

CTGTCTTTAGATTTACAGTCTAATACTTAT-CTCAGCCATTCTACC----

ATTTACTTATGTTCATCAACCGTTGACTCTTTTCAACAAATCATAAAGAT

ATTGGCACACTGTACCTTATTTTTGGTGCCTGAGCCGGGATAGTGGGTAC

TGCCCTAAGCCTATTAATTCGAGCTGAACTAGGCCAGCCAGGAGCACTCT

TAGGTGACGACCAAATTTATAACGTTATTGTTACTGCTCACGCCTTCATT

ATAATTTTCTTTATAGTAATACCCATAATAATTGGTGGCTTCGGAAACTG

ACTCGTCCCTTTAATAATTGGAGCCCCTGATATAGCATTTCCTCGTATAA

ATAATATAAGCTTCTGACTATTACCCCCATCATTCCTGTTACTTCTAGCC

TCTTCTATAGTAGAAGCTGGAGCAGGAACCGGCTGAACAGTTTACCCCCC

ATTAGCAGGCAATCTAGCACATGCAGGAGCCTCAGTTGATCTTACTATTT

TCTCACTTCACTTAGCTGGAGTATCATCAATTTTAGGGGCTATTAACTTT

ATCACAACTATCATTAATATAAAACCTCCAGCTATATCACAATATCAAAC

TCCATTATTCGTTTGATCAGTTCTTATTACCGCTGTCTTACTTCTACTTT

CACTTCCAGTTTTAGCAGCCGGCATTACTATACTCCTCACTGACCGTAAT

TTAAATACTACCTTTTTTGATCCTGCTGGAGGAGGTGACCCAATTCTGTA

TCAACACCTGTTCTGGTTCTTTGGGCACCCTGAAGTTTATATTCTCATTT

TACCCGGATTTGGCATTATTTCTCACATCGTTACCTATTATTCAGGAAAA

AAAGAGCCTTTTGGTTATATAGGAATAGTATGAGCCATAATATCAATTGG

CTTCCTAGGCTTCATCGTATGGGCCCATCACATATTTACAGTTGGCTTAG

ATGTAGATACGCGAGCATACTTCACATCAGCAACAATAATTATCGCTATT

CCCACAGGAGTAAAAGTATTTAGTTGATTAGCAACTCTTCACGGAGGGAA

TATTATTTGATCTCCCGCTATATTATGAGCCCTAGGCTTTATTTTCTTGT

TTACCGTTGGAGGGCTAACCGGTATTGTTCTAGCCAACTCATCTCTAGAT

ATTGTGCTCCATGATACTTATTATGTTGTAGCACATTTCCACTACGTATT

ATCAATGGGAGCCGTATTCGCAATTATGGGAGGTTTTGTTCACTGATTCC

CATTATTCACAGGTTATACACTTGATTCTTCCTGAGCCAAAATCCACTTT

ACAGTAATATTCGTAGGAGTAAACATGACATTTTTCCCACAACACTTTTT

AGGCCTTTCTGGAATACCACGACGATACTCGGATTATCCTGATGCATATA

CTACATGAAATGCAGTATCATCAATAGGTTCATTTATCTCTCTAACAGCT

GTAATAATTATAATCTTTATGATCTGAGAGGCATTCGCTTCTAAACGAGA

AGTAATAACAGTTGAATTACCAACAACAAATTTAGAGTGACTTCATGGAT

GCCCACCCCCATATCACACATTTGAAGAACCTACATTCGTAAAA--ACAC

AA--T-------------CAAGAAAGGAAGGAATTGAACCCCCTAAAGCT

AGTTTCAAGCCAGCCCTATAACCATTATAATCTTTCTTCATAACCA---A

GATATTAGTAAAAT-AATTACATAACTTTGTCAAAGTTAAATTACAGATC

------TAACATCTGTATATCTTACATGGCATATCCTTTCGAAATAGGCT

TTCAAGACGCCACATCACCTATCATAGAGGAACTATTAAATTTTCATGAT

CATGCTTTAATAATCGTTTTCTTGATCAGTTCATTAGTTCTTTACATTAT

CTCTCTTATATTAACAACAAAATTAACACATACTAGTACAATAGATGCCC

AAGAAGTTGAAACTATTTGAACTATTCTTCCAGCTATTATCCTTATTATG

ATCGCCCTACCATCCCTTCGCATCTTATATATAATAGATGAAATTAATAA

CCCAGTCCTTACAGTCAAAACAATAGGTCATCAATGATATTGAAGCTATG

AATACACAGATTATGAAGACTTAAATTTTGACTCTTATATAATTCCCACA

ACAGAACTAAAACCAGGAGAACTACGTCTACTAGAAGTTGATAACCGAGT

TGTATTACCAATAGAACTTCCAATCCGAATATTAATTTCATCTGAGGATG

TCCTCCACTCATGAGCAGTTCCATCTTTAGGCTTGAAAACTGATGCTATT

CCAGGACGCTTAAATCAAGCAACCCTAACATCTACACGACCGGGACTTTA

TTACGGACAATGTTCAGAAATTTGCGGATCCAATCACAGCTTTATACCTA

TTGTCCTTGAAATAGTACCACTAAAGTATTTCGAAAACTGATCCCTATCT

ATAATTTAA--------------------------------ATTACATTA

TGAAGCTA--AATTAGCATTAACCTTTTAAGTTAAAGATTGAGGATAAA-

A-CT--TCTCCATAATGAGATGCCCCAACTAGACACATCAACATGATTTA

TCATTATTCTAGCGTCTA-CCACGACATTATTCCTAATGATTCAACTAAA

ACTACATACACA--TGTTTATTCACCTAACCCTACACCTATAGATTTAAA

-AACACTTAAACACAACTGCCCTTGAGATAAAAAATGAACGAAAATTTAT

TTGCCTCATTCGTAATACCATCCTTTATTGGCCTTCCTATTGTTATTATT

ATCATTATATTTCCCATTATGCTATTCCCTTCACCAAGTCGTTTAATTAA

TAATCGTCTAATTACATTCCAATTATGATTAACACGATTAGTTCT-AAAA

CAAATAATAGCCATACATAATAACAAAGGACGAACATGATCCCTTATATT

AGTCTCACTAATTATATTTATTGGATCTACCAACTTACTAGGCTTATTAC

CTCATACATTTACCCCAACAACTCAATTATCAATAAATCTAGGTATAGCA

ATTCCCTTATGAGCAGGGGCTGTTATCATAGGATTTCGCCATAAAA-CTA

AATCATCATTAGCACATTTCCTGCCTCAAGGAACGCCTATTCCATTAATT

CCAATACTTATTATTATCGAAACAATTAGCCTATTTATTCAACCCATAGC

TTTAGCCGTTCGACTAACGGCTAACATCACAGCAGGTCATCTCCTCATTC

ATCTTATTGGTGGAGCAACATTAGCACTCATCTCTATTAGTACTCCTACT

GCTATAGTTACATTTATTATTCTTATTATATTAACAATCCTAGAATTTGC

CGTTGCCCTAATTCAGGCTTATGTATTTACACTCTTAGTAAGCCTGTATC

TACATGATAACACTTAATGACCCACCAAACCCATGCTTATCATATAGTTA

ATCCTAGCCCCTGACCAATTACAGGAGCCTTCTCAGCCCTTTTATTAACA

TCCGGCCTAGTAATATGATTTCACTTTAATTCTACTACCCTCCTCACCTT

AGGTCTAATTACTAACACTTTAACTATATATCAATGATGACGTGATATTA

TCCGTGAAGGAACTTTCCAAGGACACCACACTTCAATTGTCCAAAAAGGC

CTACGATATGGCATAATTTTATTTATTGTATCTGAAGTTTTCTTTTTCGC

AGGTTTCTTCTGAGCCTTTTACCACTCAAGTTTAGCTCCAACACCTGAAT

TAGGAGGATGTTGACCACCTACAGGAATCTTCCCACTTAACCCCTTAGAA

GTCCCTCTCCTTAACACAACAGTTCTCCTAGCTTCAGGAGTATCTATTAC

TTGGGCCCATCACAGCCTAATAGAAGGGAATCGAAATCACATGACTCAAG

CCCTATCTATTACTATTTTACTAGGCTTATATTTTACTATTCTTCAAGCA

TCAGAATATTTAGAAACATCATTTACTATCTCAGATGGAGTTTATGGGTC

AACATTTTTTATAGCTACAGGATTCCATGGACTTCACGTAATCATTGGAT

CAACTTTCCTAACTGTCTGCCTATTACGCCAACTAAACTATCACTTTACA

TCTAAACATCATTTCGGTTTTGAAGCCGCTGCCTGATATTGACATTTCGT

TGACGTAGTATGATTATTCTTATATGTATCGATCTATTGATGAGGTTCAT

ACTCT-TTTAGTAT-AA-CTAGTACTACTGACTTCCAATCAGTAAGCTTC

AGAAATTAACTGAAAAAGAGTAATAAATATAATATTAACTATTTTTATTA

ACATCATGTTAGCAACCATTTTAATTTCCATCGCTTTCTGATTACCTCAA

ATAAATGTTTACACAGAAAAATCTAGTCCATACGAATGTGGGTTTGATCC

ACTAGGCTCTGCACGCCTCCCATTCTCAATAAAATTCTTCTTAGTTGCTA

TTACTTTCCTATTATTTGACCTAGAAATTGCCCTTCTCCTTCCACTACCT

TGAGCCTCACAAATTAATAACCTCTCCCACATACTAATAATCGCACTCAT

ATTAATTTCCATCCTGGCACTAGGTTTAGCATACGAATGGTTACAAAAAG

GACTAGAATGAGTAGAATAAAACAATGGTAATTAGTTTAACATAAAA-CA

AATGATTTCGACTCATTAAATTATAAGAATATT-TATAATTACCAA--AA

TGCCTTTATTTTTACTTAATATATTCTCTGCATTCTTATTATCATTTTTA

GGGGTCTTATTATATCGATCACACCTTATATCATCACTTTTATGTTTAGA

AGGCATAATACTTTCTATATTTATTATTAACTCAGTAATTATTATTAATC

TCCACTTTTCATTATCATTCATCATACCAATTATCTTACTTGTATTCGCT

GCCTGCGAAGCTGCTATTGGTCTGGCCCTATTAGTTATAGTATCAAACTC

ATATGGCCTAGATTACGTAAAAAATTTAAATATATTACAATGTTAAAAAT

TATTATCCCCACAATCTTACTTATTCCAATCACTTGATATTCAAAAATTA

CCTCTCTATGAACAAACGTAACACTTCACAGTCTCTTAATTAGTATATTT

AGCCTTTTTCTACTTAATCAAATTGATTCTACTAACTTAAATTTTTCACT

CACATTTTCTTCAGACTCATTATCAAGCCCCCTACTAATTTTAACAGCAT

GACTATTCCCTTTAATAATTATTGCAAGCCAGAATCACTTGACTAAAGAG

GCTGAATTACGAAAAAAACTATTTATTACTACCTTAATTTCACTCCAAAT

CTTTTTAATTATAACATTTTCTGCCACCGAACTAATTTTTTTCTATATTC

TATTTGAAGCTACATTGATTCCTACTCTCATTATTATTACACGATGAGGA

AATCAAACAGAACGACTCAACGCAGGACTATATTTTCTATTTTATACCTT

AATTGGATCACTCCCACTTTTAATTGTTCTTATCTTTATCCAAACTAAAT

TAGGATCTCTAAATTTTCTAATT---ATATCTTATTCGAATAATTTAACT

GAAACTAAC-------TGATCAACTAGTCTACTCTGACTAGCATGTATAA

TAGCCTTTATAGTAAAAATACCACTATATGGTTTACATCTATGACTACCA

AAAGCCCATGTAGAAGCACCAATTGCAGGGTCAATAGTACTTGCTGCTAT

TCTACTAAAACTAGGAGGATATGGAATAATACGAGTAACTATTTTATTAG

ACCCTATTACAAATATTATATCTTATCCATTTATAATATTATCACTATGA

GGCATAATTATAACTAGCTCGATTTGCCTACGGCAAACCGACCTTAAATC

ATTAATTGCGTACTCTTCAGTTAGTCATATAGCATTAGTAATCATCGCTA

TCTTAATCCAAACCCCATGAAGCTTTATAGGAGCTACCATCCTAATAATT

GCCCATGGCCTAACCTCCTCCCTATTATTCTGCTTAGCCAATACTAATTA

TGAACGAACCCACAGTCGAACCCTTATATTAGCCCGAGGATTACAAATAG

TTCTTCCACTAATATCTACTTGATGAATTATAGCAAACTTAACCAACCTA

GCATTACCACCTTCAATTAATCTTATTGGAGAATTACTTATTATCTTAGC

CTCATTCTCATGATCCAACTTTACAATAATCTTTATAGGTCTTAATATGA

TTATTACAGCCCTCTATTCAATATATATGCTAATTATAACTCAACGAGGC

AAACTATCACAACATATCAAAATTATTACTCCATCATTTACACGAGAAAA

TATTTTAATAGTCCTACATCTATTTCCTATTATCCTACTTACTATTAGCC

CTAAAATTATTTTAGGCAACTTATACTGTAAATATAGTTTAACAAAAACA

TTAGATTGTGAATCTATTAATAGAAGACTTTAA-TCTTCTTATTTACCAA

GAAAGTAAA--GCAAGAACTGCTAACTCATTGCCACCATATATAAAAATA

TGGCTT--------------------------------------------

-------------------TCTTA-ACTTTTAAAGGATAGAAGTA-ATCC

GTTGGTCTTAGGAACCAAAAA-A-TTGGTGCAACTCCAAATAAAAGTAAT

AAAAA---TATTCGCTTCCTTTACAATATTAACCATTATAATTCTTATTT

ATCCTATTATTATAACTTTGTCAAACAACTATAATATTCTTAATTTTCCC

AAACACGTAAAATCTACTATTAAATCTGCTTTCATATTTAGCCTTATCCC

TACATTCATGTTCATCAACGCAGGATACGAAGCAGTTATCACAACTTGAC

ACTGAACTATCTTTCAAACTTTGGACATTTCCATTAGCCTAAAGATAGAT

TTCTTCTCAATAATATTTGTACCCGTAGCCCTATTTGTTACATGGTCCAT

TATAGAATTCTCAATATGATATATACATAATGATCCTAACATAGACCGAT

TCTTCAAATATCTCCTTCTTTTCCTTATTACAATAATTATCCTAGTAACA

GCAAACAACCTATTCCAACTATTTATTGGTTGAGAGGGTGTAGGGATTAT

ATCTTTCCTATTAATTGGCTGATGATATGGGCGACCCGATGCAAACACAG

CAGCACTCCAAGCCATCTTATATAATCGAATTGGTGATATTGGATTCGTT

CTATCTATAGCATGATTTTTAATCTATTCTAACTCATGAGAATTTCAACA

AC--TTTTTGTTATTAATT-GCAATAACCTGATTCCTTTACTAGGTTTGA

CTCTAGCAGCAACTGGTAAATCAGCACAATTTGGCCTTCACCCATGACTT

CCATCAGCTATAGAAGGTCCAACCCCTGTATCAGCCTTACTTCACTCTAG

CACTATAGTAGTTGCAGGCATTTTCCTACTAATCCGATTTTATCCTATAT

TA-GAAAATAATCAAACAATTTTAACAATCATCATATGTCTAGGAGCTAT

TACAACCTTATTTACAGCAATCTGTGCTCTAACCCAAAATGATATTAAAA

AAATTATTGCCTTCTCAACTTCAAGCCAATTAGGTCTTATAATAGTAACT

ATTGGTATTAACCAACCTCACCTAGCTTTTCTCCACATCTGCACTCATGC

CTTCTTTAAAGCCATACTATTCATATGTTCCGGATCCATTATCCATAATC

TAAATGATGAACAAGATATCCGAAAAATAGGAGGCCTATTTAAAACTCTT

CCTTTCACTTCATCAGCATTAACTGTTGGTAGCCTAGCATTAACAGGCAC

ACCTTTCCTAACCGGATTCTACTCCAAAGACCTCATTATCGAATCCGCTA

ACACGTCGTATACCAACGCCTGAGCCCTATTAATTACTCTAATTGCCACA

TCCATAACAGCCACTTACAGTACTCGTATTATTTTCTTCGCCCTTATAGG

CCAACCACGATTCTCACCACTTAGTCCTATTAATGAAAATAATCCCTTAT

TAAATAATGCTATTAAACGCCTACTAATCGGTAGCATTTTTGCTGGGTTT

ATTATTACCAACAACATAGACCCAATAAATATCCAACAAATAACCATACC

TTGATACTTAAAACTAGCAGCCTTAATAGTTACTATCTCCGGCTTCATAA

TCGCAATAGAACTTAATAACCTAACCTACTTTCTAAAAATCAAAATAACC

TCACA-ACACATAAAATTCTCTAATCTCTTAGGATACTTCCCATCAACCA

TTCACCGACTATTTACTAACACAAACCTAACAGCAAGCCATAAATCAGCA

TCAAAATTAATAGACTTAATTTGATTAGAAAAATCTACCCCTAAACTCCT

AACCTCACTTCAA---CTAATTATATCAACAATAACTTCAAACCAATCAG

GACTTATCAAAATTTACTTTATATCATTTCTATTATCTGTTGTGATTTCC

TTAACTCTTCTTATCTAA--------------------------TTCCCA

CGTGTAATCTCAATCACAATAAAAATGCTTACAAATAAAGATCAACCAGC

TACTACTATTAATCAACTCCCGTAACTATATAAGGCAGCAACACCTATTG

AATCTTCACGAATTAAACCAACATCATCAGTCTCGAACAACACTCAATCC

TCTAGTCCATTGAAATTAATAATTACCTCCA---------CCTCATCATA

AATTTTTATTAACAAAATTAAAACAATCTCCACCACAAACCCCGATAAAA

GTATACTTCATACCATAGTATTAGAGCCCCATGTTTCAGGGTATTCTTCA

GTAGCCATAGCAGTTGTATAACCAAATACAACCAACATCCCACCTAAATA

AATCAAAAACATTATTAAACCTAAAAATGAACCCCCAAAATATAATACTA

TGCCACACCCAACCCCTCCACTTAAAATCAACCCTAAACCTCCATAAATA

GGTGAAGGCTTCAAAGAAATCCCTAAAAAGCCAAACACAAATGCTAAACT

TAACAGAAATAAAATATATGTCATAGT---TTTTA-CATGGAAT-TAAAC

CATGACTAATGACATGAAAAATCATCGTTGTAAATTCAACTATAAAAACC

--C---TAATGACAAACATCCGCAAATCTCATCCACTCATTAAAATTATT

AATGACTCTTTCATCGATCTTCCAACTCCATCCAACATTTCAGCATGATG

AAATTTCGGTTCCCTCCTAGGCGCTTGCTTAGGCATTCAAATTCTTACAG

GTCTATTTCTTGCTATACATTATACATCAGATACAATAACAGCATTCTCC

TCAGTTACACACATCTGCCGAGATGTAAATTACGGCTGATTAATTCGTTA

TATACACGCAAACGGAGCATCCATATTCTTTATTTGTCTTTTCCTTCACA

TCGGCCGAGGTGTCTACTATGGCTCCTATATATTTACAGAAACATGAAAT

ATTGGAATCATTCTCCTATTCGCAGTTATAGCAACTGCATTCATGGGATA

TGTCCTTCCATGAGGACAAATATCTTTCTGAGGAGCAACAGTAATTACCA

ATCTATTATCTGCCATCCCTTATATTGGTACAACCCTAGTAGAATGAATC

TGAGGGGGTTTCTCAGTTGATAAAGCTACCCTAACACGATTCTTCGCATT

TCACTTTATCTTACCTTTCATCGTCGCAGCCCTAGTAATAGTTCACCTTC

TTTTCCTACACGAAACAGGATCCAACAATCCATCAGGCCTAAACTCTGAC

ACAGACAAAATTCCTTTCCACCCCTACTTTACAATTAAAGATATTCTAGG

CCTTCTTCTCCTCATTTCTCTTCTAATATCACTAGTATTATTTTCCCCAG

ACCTTCTAGGTGACCCTGATAATTATACACCCGCTAATCCCCTAAGCACT

CCTCCCCACATTAAACCAGAATGATATTTCCTATTTGCATATGCCATCCT

TCGATCTATTCCCAACAAACTAGGAGGAGTATTAGCCCTCGTATTCTCAA

TCTTAATCCTAGCAATCTTCCCTATCATCCAAATCTCTAAACAACGAAGC

ATAATATTTCGTCCCTTAAGCCAACTCCTATTCTGAATTTTAACAGCAGA

CCTATTTACATTGACATGAATTGGAGGTCAACCAGTTGAACACCCATTTA

TTATCATTGGCCAATTAGCCTCAATCCTATATTTTTCTATTATTCTTGTT

ATTCTTCCCATAATTAGCCTTATCGAAAATAAGCTTCTAAAATGAT-A--

--GTCCTGATAGTATAAC-CCA-TTACACTGGTCTTGTAAACCAGAAATG

AGATTCATTAACTTCTCTTAGGATAA----TCAAGGAAAAGG-CCCTAGC

CCCACTACCGACCCCCAAAGCCGGAGTTCTA--ATTAAACTATTCCTTG-

--------------------------------------------------

--------------------------------------------------

--------------------------------------------------

--------------------------------------------------

--------------------------------------------------

--------------------------------------------------

--------------------------------------------------

--------------------------------------------------

--------------------------------------------------

--------------------------------------------------

--------------------------------------------------

--------------------------------------------------

--------------------------------------------------

--------------------------------------------------

--------------------------------------------------

--------------------------------------------------

--------------------------------------------------

--------------------------------------------------

--------------------------------------------------

--------------------------------------------------

--------------------------------------------------

--------------------------------------------------

--------------------------------------------------

------

>Eliomys_quercinus Eliomys quercinus mitochondrion, complete genome.

GTTAATGTAGCTTAA--T------AACAAAGCAAAGCACTGAAAATGCTT

AGATGGGTGTTAA-C-ACCCCATAAACATAAA--GGTTTGGTCCTGGCCT

TCTTATTGATTCTTAACAAGATTACACATGCAAGCATCCACGCCCCTGTG

AGAATGCCCT-ATAAATTTTACTATGTA-AACCTAAAGGAGCAGGTATAA

AGCACAC---GCAATAGTAGCTCAAAACACCTCGC-TTAGCCACACCCCC

ACGGGACACAGCAGTGATTAAAATTAAG-CAATAAACGAAAGTTTGACTA

AGCTATGTTA---ATACAGGGTTGGTCAATCTCGTGCCAGCCACCGCGGT

CATACGATTAACCCAAGTCAATAAGTT-TC-GGCGTAAAGAGTGTTTTAG

A----TTAATCAAGTATAATAAAGTTAAGTCTCATCTAAACCGTAAAAAG

TC-CTAGATGAAGATAAAATAAGGAACGAAAGTCACTTTATAAACTAATC

TGAATACACGATAGCTAAGACACAAACTGGGATTAGATACCCCACTATGC

TTAGCCCTAAACACAAAAA-ATTCAT--AACAAAATTATTCGCCAGAGAA

CTACGAGCCACAGCTCAAAACTCAAAGGACTTGGCGGTGCTTTATATCCC

TCTAGAGGAGCCTGTTCTATAATCGATAAACCCCGATATACCTTACCTCT

TCTTGC-TATTTCAGCCTGTATACCGCCATCTTCAGCAAACCTTAA-CAA

AGGACAAAAGTAAGCACAAGTATGA---ACATAAAAACGTTA-GGTCAAG

GTGTAGCCAATGAATAGGG-AAGAAATGGGCTACATTT----ACTTTAT-

AAGTA------AAT----ACGCAAACTCTCGTGAAATCCGA--GAGCAGA

AGGCGGATTTAGTAGTAAGTT-AAGAATAGAGAGCTTAACTGAATCG-GG

CCATGAAGCACGCACACACCGCCCGTCACCCTCCTCAAGTATTTAATAAC

AAAACAGCATACCCAAATGCACGTAAACATAA-----TT-CA--AGAGGA

GACAAGTCGTAACATGGTAAGCGTACTGGAAAGTGTGCTTGGAATAATCA

AAATGTAGCTTA----AAA-AAAGCATCTGGCTTACACCCAGAAGACTTT

ACCA-ATTGTAAACATTTTGA-ACT-AACCCT-AGCCCACCTATTAA---

ATTAAATTCAACTA-TATAAAAAATTAAAATAAATCATTTAGA-TT-AA-

--TAAAAGTATAGGAGATAGAAATTATA--ATAT--GGCGCTATAGAGAT

AGTACCGCAAGGGAAAGA-TGAAAGATT--ATTAAAAGTATAAAAAAGCA

AAGATAACACCTTGTACCTTTTGCATAA-TGAGATAACAAGAAATGATTT

AGCATAAAG-AATTGAAGCTAAACACCCCGAAACCAAACGAGCTACTCAT

GAGCAGCC--A-TTAGAGCAAACCCGTCTATGTGGCAAAATAGTGGGAAG

ACTTCTGAGTAGAGGTGAAAAGCCAATCGAGTTTGGTGATAGCTGGTCAC

CCAGGATAAGAATTTAAGTTCAACTTTAAACTTACCTAAAGAAAT-TA-A

AA-TCTTAATGTAAGTTTTAAGTATACTCTATAGAGGTACAGCTCTATA-

GAT-AAGGAAAAAACCTGAAATAGTGAGTAA-----AATTTATAATAA--

CCACAGTTGGCCTAAAAGCAGCCATCAGTTAAGAAAGCGTTCAAGCTCAA

C---ACTAATATATTAAATTAATC-TAAGCATTC---TTTACTCAACT--

CCTATTTTTTC-ACTGGGTTAATCTATTACTCAAT-AGAAGAGATAATGT

TATTATAAGTAACAAGAA-TTATA-TTCTCCTAGCACAAGTCTATATCAG

ATCGAA-TACATCACTGATAGTTAACAATAAGGTA-----ACAATAATAA

AACAAAACACT--TACCTACCCTAATTGTTAACCCAACACAGGAGTGC--

--TATAAGGAAAGATAAAAAGAAGTAAAAGGAACTCGGCAAATACAAACC

CCGCCTGTTTACCAAAAACATCACCTCTAGCATAAAAAGTATTAGAGGCA

CCGCCTGCCCAGTGACA-TACG----TTCAACGGCCGCGGTATTCTGACC

GTGCAAAGGTAGCATAATCATTTGTTCCTTAACTAGGGACTTGTATGAAT

GGCATGACGAGGGTTTAACTGTCTCTTACTTCCTATCAGTGAAATTGACC

TTCCAGTGAAGAGGCTGGAATAAACCAATAAGACGAGAAGACCCTATGGA

GCTTAAATT-AACCAATCTAACCATATTTAAAGCTACTCTAC------AC

GAAATCTAACATTATATGAACCTAGATTGGCAATTTTGGTTGGGGTGACC

TCGGAGCATAAAAAAACCTCCGAAAGATA-AA-TTTAGACTAACTAGTCG

AAATGATATTATCCA----TCTAAATGATCCAA-ACACTATTTGATCAAC

GGAACAAGTTACCCTAGGGATAACAGCGCAATCTTGCTTAAGAGTTCATA

TCGACAGC-AAGGTTTACGACCTCGATGTTGGATCAGGACATCCTAATGG

TGCAGCAGCTATTAAGG-GTTCGTTTGTTCAACGATTAATAGTCCTACGT

GATCTGAGTTCAGACCGGAGCAATCCAGGTCGGTTTCTATCTATTTACA-

AGTCTCTCCCAGTACGAAAGGACAAGAGAGGCAAGGCCTATTAACCATAT

AAAGCCTTAACCCACACAGATGAA-ATA-TCTCAATCTGATAAAGTATCA

TAAAA-----TCACCCTAGATAAGGG---TTCGTTAAGGTGGCAGAGCCC

GGTAA-TTGCATAAAACTTAAAACTTTATATTCAGAGATTCAAATTCTCT

CCTTAACAAA----------------------------------------

------------------------------------------ATGTTCTT

AATTAATCTATTAATATTAATTATCCCAATCTTATTAGCCATAGCTTTCC

TAACACTAGTAGAACGAAAAATTCTAGGATACATGCAACTCCGTAAAGGT

CCTAACATCATCGGACCTTACGGCACCCTCCAACCATTTGCTGATGCAAT

AAAACTATTTATTAAAGAACCACTACGACCATCATATTCATCCATAAGCC

TATTCACCATTGCACCCACACTAGCATTAACTCTAGCATTCACCATATGA

ATCCCCCTCCCCATACCCCATCCACTAATCAATATAAACATAGGACTAAT

TTTTATACTTGCAACCTCCAGCTTAGCCGTATATTCCATCCTATGGTCAG

GCTGAGCATCAAACTCCAAATACTCGTTAATTGGAGCCCTACGAGCCGTA

GCCCAAACGATCTCGTATGAAGTAACACTCGCTATTATTCTCCTATCAGT

ATTAATATATAATGGCTCTTTTACGCTACGTACATTAATACAAACCCAAG

AGCACATATGGCTAATTTTACCAACATGACCATTAGCCATAATATGATTT

ATTTCTACCCTTGCCGAAACAAACCGAGCACCTTTTGACCTAACAGAAGG

CGAATCAGAGCTTGTATCCGGATTCAACGTAGAATACGCAGCAGGACCCT

TCGCCCTATTCTTTATAGCTGAGTATACCAATATCATTATAATAAATGCC

CTAACCTCTATTATCTTCCTAGGGGCATTAGACGACACTTCCAACCCCGA

AATATCTACATTAAGCTTTATAATTAAAACCTTATTCCTTACCTGCACTT

TCTTATGAATCCGAGCTTCATACCCACGATTCCGATATGACCATCTTATA

CATCTACTATGAAAAAATTTCCTACCGTTAACTCTAGCTATGTGCATGTG

ACATGTAACTATACCCATCGCACTATCCAACATCCCACCATTAACTTAA-

GAAACATGTCTGATAAAAGAGTTACTTTGATAGAGTAAATAATAGAGGTT

TA-AACCCTCTTGTTTCTAGAGTTATAGGGATTGAACCTAAACCTAAGAA

TTCAAAATTCTTCGTGCTACCCCAAATACACCAAACTCTAACC--T---A

GTAAGGTCAGCTAATT-AAGCTATCGGGCCCATACCCCGAAAATGTTGGT

TTATATCCTTCCCGTACTAATCAATCCATTCACCGCCTCAGCTATTCTCA

TCACATTATTTTCTGGGACTATAATTGTATTATTTAGCATACACTGATTA

TCAACTTGGATCGGCCTAGAAATAAGTATGCTAGCCATCATCCCAATTAT

TATTAACAAAGCAAGCCCGCGGTCCACGGAAGCCGCAACCAAATATTTCC

TAGTCCAAGCTACAGCCTCCATGATCTTAATAATAGCCATCATCTTAAAC

ATATTACTCACCGGGCAATGAACAATAATTAATTTTTCCAACTTTTATCC

AGCTACCTTGGCTACAATTGCCTTAGCAATAAAACTAGGACTAAGCCCAT

TTCACTTTTGAGTCCCAGAAGTCATTCAAGGCACATCTCTTTTATCAGGC

ATAACAATCCTAACATGACAAAAAATTGCCCCTATATTCATTATATACCA

AATTCACCCATTCATCAACTCCAACCTCCTATTATCTATAGCACTTCTAT

CAATCGCACTAGGGGGTTGAGGAGGGCTAAATCAAACTCAACTACGAAAA

ATTATAGCCTACTCATCAATTGCCCACATAGGATGAATGATAGCTATTAT

ACCATTCAACCCCACCGCTATATTACTTAACCTAATTATTTATATTATAC

TCACCATTACTATATTTATTATTCTCTACCATAATAATAACACCACAACA

CTTAACCTGTCAATAACATGAAGCACCACCCCCTTAATAATCTCCGCAGC

ACTAATCACATTAATATCCCTAGGAGGTCTGCCCCCACTTACAGGTTTTA

CACCCAAATGGGTTATTATTCAAGAACTAATTAAAAACAAAGTAACAATC

CTGGCCACAATTATAGCCATATTAGCCCTACTAAATTTATACTTCTACAC

CCGCCTTATTTACTCAACTTCCCTGACATTATTCCCATCAACAAACAATA

TAAAAATAAAATGAAAATTTGAAAGTACAAAACTAACCACACTAATATCC

CCTATACTACTCCTATCTACATTAACTTTGCCAGTTATACCTATTA-TAT

CTATCCT--AAACT----AGGAATTTAGGTTAAA----TAGACCAATAGC

CTTCAAAGCTTTAAGCAAGTAGTATA-T---ACTTAATTCCTG-------

-----TA-ATAAGGATTGCAGGATA---TCCTACAT----CATCTGAATG

CAAGTCAAACGCTTTCA--TT-AAGCTAAATCCTTACCCCACA--TTCTA

GGTTGACG--AGACTTAAA-CTCGC-GAAACTTTAGTTAACAGCTAAAAA

CCCTAATCAACTGGCTTCAACCTA-CT-TCTCCCGCCGCGAAAAAAAAAA

A-AGAGGCGGGAGAAGTCCCGGCAG-C-TCTGA-AGCTGCTCCTTT--GA

-----------------------------ATTTGCAATTCAATG--TGAT

TA-ATCACCTCAAGACT-T-----GATAGGAAGAGGT----C--CACCCT

CTGTCTTTAGATTTACAGTCTAATGCTTA--CTCAGCCATCCTACC---C

ATCTACTTATGTTCATTAACCGTTGACTTTATTCAACTAATCACAAGGAT

ATTGGTACTCTTTACTTATTATTTGGTGCCTGAGCTGGGATAGTAGGCAC

TGCCTTAAGCCTGCTAATCCGAGCAGAGCTCGGCCAACCAGGGGCATTAA

TGGGTGATGACCAAATCTATAATGTCGTAGTCACAGCCCACGCTTTTATT

ATAATTTTCTTCATAGTCATGCCAATCATAATTGGAGGGTTCGGAAATTG

ACTTGTCCCATTAATAATTGGAGCGCCTGATATAGCTTTCCCTCGAATAA

ATAATATAAGCTTCTGACTTCTTCCGCCCTCATTTCTTCTTCTTCTGGCA

TCCTCTATAGTAGAAGCTGGAGCAGGAACAGGGTGAACTGTATACCCCCC

ATTAGCAGGGAATTTAGCACATGCAGGAGCCTCAGTTGACCTAACTATTT

TCTCACTTCACCTAGCCGGAGTATCATCAATCCTAGGCGCTATCAATTTT

ATCACAACTATCATCAATATAAAACCACCAGCCATATCGCAATATCAAAC

ACCTCTATTCGTATGATCAGTCCTTATTACAGCTGTTTTACTATTATTAT

CCCTACCCGTGTTAGCCGCAGGCATTACAATACTTCTAACGGACCGAAAT

CTTAACACTACTTTCTTCGACCCTGCTGGCGGAGGAGACCCTATCCTATA

CCAACACTTATTCTGATTCTTCGGACACCCAGAAGTATATATCTTAATCC

TTCCAGGCTTCGGCATTATTTCTCATATCGTAACTTATTATTCAGGAAAA

AAAGAGCCATTTGGCTACATGGGAATAGTATGAGCTATAATATCAATCGG

ATTTCTAGGCTTCATTGTATGAGCCCATCATATATTTACCGTGGGGTTAG

ATGTAGACACCCGAGCGTACTTCACATCCGCTACCATAATTATTGCTATC

CCTACTGGAGTAAAAGTATTCAGCTGATTAGCCACCTTACATGGAGGTAA

TATTAACTGATCTCCAGCCATACTATGAGCACTAGGATTTATTTTCCTAT

TCACTGTGGGGGGACTTACAGGGATTGTTCTAGCCAATTCATCATTAGAC

ATTGTCCTTCACGACACCTACTACGTAGTAGCCCACTTCCACTATGTACT

ATCTATAGGGGCCGTTTTTGCAATTATGGGTGGGTTCGTACACTGATTCC

CATTATTCTCAGGCTACACAATCGACACGTCATTAGCCAAAATGCACTTT

ACTATTATATTTATAGGAGTCAACCTCACATTCTTCCCACAACACTTTCT

AGGCCTATCAGGTATACCACGACGATATTCTGACTACCCAGACGCTTATA

CTACATGAAACGTGGTATCCTCATTAGGATCATTTATCTCACTAACAGCC

GTAATCTTTATAATTTTTATGATCTGAGAAGCATTTGCTTCCAAGCGAGA

AGTAGTAATAGTTGAATACTCATCAACAAATTTAGAATGATTACACGGAT

GTCCTCCCCCATATCACACATTCGAAGAACCAACTTACGTAAAAACTCTT

AA--C-------------CAAGAAAGGAAGGAATTGAACCCCCTAAAGCT

AGTTTCAAGCCAGCCCTATAACCTTTATAATCTTTCTTCATAA-------

GGTATTAGTAAAAC-AATTACATAACTTTGTCAAAGTTAAATTACAGACC

CCCCCCTCAATTCTGTATATCTTAAATGGCTTATGCACTCGAACTAGGAT

TTCAAGACGCAACATCCCCAATTATAGAAGAACTACTCAACTTCCACGAT

CACGCCCTAATAATTGTATTTCTTATTAGCTCCTTAGTATTATATATTAT

CTCCTTAATACTGACAACGGAGCTAACCCATACAAGCACAATAGATGCAC

AAGAAGTAGAAACTATTTGAACCATTCTACCAGCTATTATCCTCATTATA

ATTGCCCTCCCATCTTTGCGAATCTTATACATAATAGATGAAATCAACAA

CCCAATCCTAACAGTCAAAACTATAGGCCACCAATGATACTGAAGTTATG

AGTACACGGACTACGAAGATTTGAACTTCGACTCTTACATGGTCCCTATA

GATGACCTCAAACCTGGAGAGATACGATTACTAGAGGTAGATAATCGTGT

TGTCATACCAATAGAACTCCCTGTACGAATACTTATTTCATCAGAAGACG

TATTACACTCATGAGCCGTCCCCTCATTAGGATTAAAAACTGACGCAATC

CCAGGACGCCTAAATCAAGCTACATTAACATCCACACGACCGGGGATTTA

TTATGGTCAATGCTCAGAAATTTGTGGATCAAATCATAGCTTCATACCTA

TTGTATTAGAAATCGTTCCACTAAAAAATTTCGAAAATTGATCCTTGTCT

ATAATTTAA---------------------------------CAGCATTA

TGAAGCTA--AAATAGCATTAACCTTTTAAGTTAAAGACTGAGAATTCTA

A-TT--TCTCCATAATGAAATGCCACAATTAGACACATCAACGTGATTTA

TTACTATTATATCTACTA-TAATAGCACTATACTTGATTATTCAACTTAA

AATCTCAACACA--CCTTCACCAACCAAACCCTGCACCCAAAGATTTTAA

-ATCCTTAAAACATAAAACCCCTTGAGATACAAAATGAACGAAAATTTAT

TTGCCTCATTCATAACCCCAACAATCCTAGGCCTACCTGTAGTAATCCTG

ATTATTCTATTCCCAAACATCTTATTCCCCAAAATGGACCGACTTATTAA

CAACCGCCTAATTTCCTTTCAACTTTGATTAACACGCCTAATCCT-AAAA

CAAATAATAACAATACATAGCAATAAAGGACGAACATGATCACTGATAAT

AGCATCACTAATACTATTTATTACGTCCACAAATCTTCTTGGCCTTATAC

CATATACATTCACACCTACAACTCAACTATCTATAAATCTAGGCATAGCT

ATCCCTTTATGAGCTGGTGCCGTTATCACCGGATTCCGTTATAAAA-TCA

AATCATCTCTAGCCCACTTCTTACCCCAAGGCACACCTCTTCCACTTATC

CCTATACTAATTATTATCGAAACTATTAGTCTATTTATTCAACCCATAGC

ACTAGCTGTGCGACTAACCGCCAACATTACAGCAGGACACCTACTAATAC

ATCTTATCGGAGGAGCCGCACTAACCCTAATATCTCTTAGTGTCCCAACC

GCCATGGTAACATTTATTATTCTAGTGTTACTCACAATTTTAGAATTCGC

AGTGGCTCTCATTCAAGCCTATGTATTCACCCTCCTTGTAAGCCTTTATC

TACATGACAATACCTAATGACCCACCAAACACACGCTTACCATATAGTTA

ACCCAAGCCCATGACCACTAACAGGAGCTCTATCAGCCCTTCTACTCACA

TCAGGCTTAGTCATATGATTCCACTATAATTCAAACACTATTTTAAACCT

AGGATTTATTACAAATCTATTAACTATATACCAATGATGACGAGACATTG

TACGCGAAGGCACATTCCAAGGCCACCACACACCAATCGTACAAAAAGGC

CTACGATACGGAATAATTTTATTTATTGTCTCAGAAGTGTTTTTCTTTGC

TGGCTTCTTTTGGGCATTCTATCACTCCAGCCTAGCACCAACACCCGAAT

TAGGAGGATGTTGACCACCAACAGGAATCTTACCGCTCAACCCACTTGAA

GTTCCACTACTAAATACTACAGTCCTACTGGCATCAGGAGTATCAATTAC

TTGAGCTCACCACAGCTTAATAGAAGGGGATCGAAACCATATAATTCAAG

GCCTATCTATTACAATTATTTTAGGCCTATATTTTACCATTCTTCAAGCC

TCTGAATACCTGGAAACACCTTTCACAATCTCAGACAGTGTATACGGATC

AACATTCTTCATAGCCACAGGATTCCATGGACTTCACGTAATCATTGGTT

CTACCTTTCTCGGCGTATGTCTCCTTCGACAATTAAACTTTCACTTTACA

TCCAACCACCATTTCGGATTTGAAGCAGCCGCCTGATACTGACATTTCGT

AGATGTAGTCTGACTTTTCCTTTACGTATCTATTTATTGATGAGGCTCAT

ACTTT-CTTAGTATCAA-CTAGTACAACTGACTTCCAATCAGTTAGACTC

AGCCTTAATCTGAGAGAAAGTAATTAATATAATAATAACTATTTTTATCA

ACTCAACACTAGCCCTCCTATTAATCTCTATTGCCTTCTGACTACCACAA

ATAAATACCTATAATGAAAAATCTAGCCCCTATGAATGCGGATTTGACCC

ACTTGGATCAGCACGTCTACCATTCTCAATAAAATTTTTTCTAGTGGCTA

TTACCTTCTTACTATTCGACCTAGAGATCGCTCTCCTACTTCCACTCCCA

TGAGCTTCCCAAATCAACAATCTATCCACCATATTAACTATAGCACTCAT

ACTAATCTCCATCCTAGCCCTTGGTTTAGTATACGAATGAACTCAAAAGG

GTTTAGAATGAACTGAATAAC----TGGTAATTAGTTTAAACCAAAA-CA

AATGATTTCGACTCATTAAATTATAAGACTATT--ATAATTACCAA--TA

TGCCCTTAATTATATTTAACATAATCTCAGCATACGCATTATCCCTATTA

GGAGTAATAATCTACCGGTCACACCTAATATCATCCCTATTATGCCTAGA

GGGAATAATACTCTCTATATTTATTATCAACTCATTGATTTCCTTAAATT

TGCATTTCACTTTATCATATATAATACCTATTATACTACTAGTATTCGCA

GCGTGTGAGGCAGCAGTAGGATTAGCCCTGCTGGTAATAATCTCCAACTC

CCATGGACTTGATTACGTAAAAAACTTAAATCTTTTACAATGCTAAAAAT

CATTATCCCAACCACCCTACTGATTCCAATCACATGATACTCTAAACCCT

CATTAATATGAATAAATATTACACTCCACAGCCTACTTATTAGCCTTATT

AGTCTGTTCCTTTTCTACCAATTAAATGAAAACAGCATAAACTTCTCAAC

CCTATTCTTTTCTGACCCCTTATCAAGCCCATTATTAATCCTAACAACAT

GACTCTTACCACTAATAATTCTAGCCAGCCAAAATCACCTAGCCAAAGAA

AGCCATGCACGAAAAAAACTATTCGCAACAACCCTAATCTCCCTTCAAAT

CTTTCTAATCATAACATTTTCAGCCTCAGAACTTATCATATTCTACATTC

TATTTGAAGCCACATTAATTCCCACACTAATTATTATTACACGTTGAGGT

AACCAAACAGAACGGCTCAACGCCGGATTATATTTTCTATTCTATACACT

AATTGGATCCCTTCCCCTTCTAGTAATCCTCATCCACATTCAAGTTTCAT

CAGGATCTCTCAACTTACCCATG---ATAATAC-------TAACTAGTTC

AAATATAAACAACAACTGATCAAGTAACCTAACATGATTAGCATGTATAA

TAGCTTTCATAGTGAAAATACCCCTATACGGAGTACATCTTTGACTACCA

AAAGCCCACGTTGAAGCTCCAATTGCAGGATCTATAGTCCTCGCGGCAAT

CTTACTAAAACTAGGAGGCTATGGGATAATACGCATTACCATCATCTTAA

ACCCTATTACAGAAACTATAGCATACCCCTTCATTATATTATCCTTATGG

GGAATAATCATAACCAGCTCTATTTGTCTGCGACAAACAGACTTAAAATC

ACTAATTGCTTACTCATCAGTAAGCCACATAGCACTCGTAATCGTAGCAA

TTATAATTCAAACACCATGAAGCTTTGCAGGAGCCACCATCCTAATAATT

GCTCACGGCCTAACTTCCTCCCTTCTATTCTGCCTAGCCAATTCTAACTA

CGAACGAGTACACAGCCGAACCTTAACACTAGCCCGAGGCATACAAGTTA

TTCTACCCTTAATAGGAACTTGGTGAATCATGGCTAACCTTACAAATCTA

GCACTCCCTCCGACCATTAATTTAATAGGAGAACTGGCAATCATCATGTC

GTCATTCACATGATCAAACTTTACAATTATCTTCATAGGAATCAACATAC

TAATCACTGCCCTATACTCTATATATATATTAATCATGACACAACGAGGA

AAAACACCCCACCACATCAACAACCTAACACCATCATTTACACGAGAAAA

TATATTAATAACAATACACCTAATCCCTATCCTATTATTAATAATCAATC

CTAATCTTATTATAGGCAATCTCCCATGTAAACATAGTTTAAGAAAAACA

ATAGATTGTGAATCTATTAATAGAAGAACT-AAGCCTTCTTGCTTACCAA

GAAAGTACA--ACAAGAACTGCTAACTCATTGTAACCGTACTTAAAAATA

CGGCTT--------------------------------------------

-------------------TCTTA-ACTTTTAAAGGATAGGAGTT-ATCC

ATTGGTCTTAGGAGCCAAAAA-A-TTGGTGCAACTCCAAATAAAAGTAAT

AAATA---TATTTTCTTCCTTCACACTACTTACACTCACTATTTTAACTT

ACCCAATTTTACTCTCACTAACTAATCCAAGTGACAACCCTACATTCCCC

CAATACGTAAAAATATCAATTATTCTAGCCTTTACATTCAGCCTTATCCC

CACCCTCATATTCATTCACTCTGGCTACGAAACTGTTATTTCCAACTGAC

ACTGAACATCAATTAACGGGTTTAAAATCTCAATAAGCCTGAAAACGGAT

TTTTTCTCAATTATATTTATCCCAGTAGCCCTATTTGTAACATGATCCAT

TATGGAGTTCTCCCTATGGTATATACACTCAGACCCCAATATTAACCGAT

TCTTTAAATATTTATTACTCTTCCTGATTACTATAATCATCCTGGTCACA

GCAAACAACCTGCTACAATTATTCATCGGCTGGGAAGGAGTTGGAATTAT

ATCTTTCTTATTAATCGGGTGGTGACAAGCACGGTCAGAGGCAAACACAG

CGGCAATACAAGCAATACTATATAATCGAATTGGGGATATCGGTTTCGTA

TTATCTATAGCATGATTCTTAAAACACTCAAACTCATGGGAACTACAACA

ACTCTTCATACTTAACCAAACTACCAGCTACCTCCCTCTGCTAGGATTAA

TTCTAGCTGCAGCTGGGAAATCAGCACAATTTGGCCTCCACCCATGACTA

CCATCAGCCATAGAAGGCCCTACACCAGTATCAGCATTACTCCACTCCAG

CACAATAGTTGTAGCTGGTGTGTTTCTTCTAGTACGATTCCACCCATTAC

TG-GAAAATCACCCATCAATGCAAACCACCATCCTATGCCTAGGAGCCCT

AACCACCCTGTTTACAGCAATCTGCGCTTTAACACAAAACGACATTAAAA

AAATTATCGCTTTCTCTACCTCTAGCCAACTAGGCCTTATAATAGTAACA

ATTGGCATTAACCAACCACACCTAGCCTTCTTTCATATCTGCACGCATGC

TTTCTTTAAAGCAATATTATTCTTATGCTCCGGATCTATTATCCACAATC

TAAACGATGAGCAAGACATTCGAAAAATAGGAGGCCTCTTCAAAACCCTA

CCACTCACATCCTCCGCACTAACAATCGGAACATTAGCACTAACTGGAAC

TCCATTCCTAACAGGTTTTTATTCCAAAGACCTTATTATTGAGTCCGCTA

ACACGTCGTACACCAACGCCTGAGCCCTATTAACTACTCTCATTGCCACT

TCCCTAACAGCTGTCTACAGTACTCGCATCATCTTCTTCGCCCTTATAGG

CCAACCACGTTTCCTCCCTATATCGCCAATCAATGAAAATATCCCTACCC

TGAACAATTCAATTAAACGACTACTAATCGGTAGCATTTTTGCCGGATTC

TTAATTACTAACAACATAAACCCCACCAGCATCCAACAAATAACCATACC

ATGATTTATTAAACTAGCAGCATTAATCGTTACTATTTCAGGATTTATAA

TGGCCATAGAACTTAACAATATATCACTGTACTTTAAATTTAAAACTACC

TCAAA-ATACATAAAATTCTCTAATATGCTAGGATACTTTCCCCAAGTAA

CCCACCGACTTATTCCGAATATAAACCTTATTATAAGCCAAAAATTAGCC

TCCTCACTGTTAGACAACACCTGACTAGAAAAATTAACACCCAAATTTAT

CTCTATAATTCAA---CTAACCATATCAACTTTAACCTCAAGTCAAAAAG

GATTAATTAAACTATACTTTTTATCTTTCCTTCTATCAACCCTAGTACTA

CTAATAATTATATTTTAA--------------------------TTGCCA

CGAGTAATCTCAATAGCAATAAAAATACTAACAAACAATGACCAACCAGC

TACAACTACTAGTCAACTCCCATAACTATACAAAGCAGCCACACCTATAG

AATCTTCACGAACCAGGCCAACATCATCAGTCTCAAATAAGACTCAATCC

TCGAGCCCATTAAAATTAATAACAACTTCAA---------CACCATCATA

GACTTCCATTAATAAGATTAAAATAATCTCTAAGATAATTCCAGATAATA

AAATACTTCAAGATAAAATATCTGAATTCCACGTCTCAGGATACTCCTCC

GTGGCCATAGCTGTAGTATAACCAAAAACAACCAACATCCCACCCAAATA

TACCAGAAATACCATTAAACCCAAAAATGAACCACCAAAATACAATACAA

TCCCACAACCCACTCCACCACTCAAAATCAACCCTAAACCTCCATAGATT

GGAGAAGGCTTTAGAGCAACCCCTAAAAAGCCAAAAACAAAAGCTAAACT

TAGTAAAAGTAAAGTAAATGTCATAAT---TTTTA-CATGGAGTCTAGAC

CATGACCAATGACATGAAAAATCATCGTTGTAA-TTCAACTATAAAAACA

TCC---TAATGAAAAATATCCGAAAAACACACCCGCTAATTAAAATTATT

AACCATTCATTCATTGACTTACCAACACCCTCTAATATTTCAGCATGATG

AAACTTCGGCTCGCTACTAGGAGTATGCCTAGGAATTCAAATCCTAACAG

GCCTATTTCTCGCGATACACTACACCTCTGATACTTTAACAGCATTCTCT

TCAGTAACCCACATTTGCCGCGATGTTAATTATGGCTGATTGATCCGCTA

TATACACGCTAACGGAGCATCAATGTTTTTTATTTGCCTATTCCTTCACG

TAGGACGCGGAATTTACTATGGGTCATACCTATACACCGAAACATGAAAT

ATTGGAATTATTCTCCTGTTCGCAGTTATGGCAACTGCTTTCATAGGGTA

TGTTCTTCCATGAGGACAAATATCTTTTTGAGGGGCTACAGTAATTACAA

ATCTCCTGTCCGCCATCCCCTACATCGGAACAACATTAGTAGAGTGAATC

TGAGGAGGCTTCTCCGTAGACAAAGCAACCCTAACACGATTCTTCGCATT

CCACTTCATTCTCCCATTCATCATTACTGCCTTAGTAATAGTGCACCTCT

TATTTCTACACGAAACAGGATCTAACAACCCCTCAGGGATTAACTCTGAC

TCAGATAAAATTCCGTTTCATCCTTACTTTACTATTAAAGATATTTTAGG

CTTTCTTCTCCTAATCTCATTAATAATGTCTCTAGTATTATTCTCCCCAG

ATCTCTTAGGAGACCCAGACAACTACACACCAGCAAACCCTCTAAACACA

CCCCCACATATTAAACCAGAATGATACTTCTTATTCGCATATGCCATCCT

ACGATCCATTCCTAACAAACTAGGAGGAGTATTAGCCCTAGTATTCTCTA

TCCTAATTCTAGCTTTATTCCCACTTCTACAACTCTCCAAACAACGAAGC

ATAATATTCCGACCAGTCAGCCAATGTGTATTCTGAATTCTGACAGCTGA

CCTCCTAACTCTAACCTGAATCGGTGGACAACCAGTCGAACACCCATTTA

TTATCATCGGTCAGGTAGCATCTATTCTCTACTTCTTAAATATCCTAGTT

ATACTCCCTATAGCTAGTCTACTAGAGAATAAACTTTTAAAATGAAGA--

--GCTCTGATAGTATAATACCAATTACTCTGGTCTTGTAAACCAGCAATG

AGATAGACA---TTCTCTCAGAACAA---ATCAAAGAAGAAG-CACTAGC

CCCACCATCGGCACCCAAAGCCGAGATTCTACTTTTAAACTATTCTTTG-

--------------------------------------------------

--------------------------------------------------

--------------------------------------------------

--------------------------------------------------

--------------------------------------------------

--------------------------------------------------

--------------------------------------------------

--------------------------------------------------

--------------------------------------------------

--------------------------------------------------

--------------------------------------------------

--------------------------------------------------

--------------------------------------------------

--------------------------------------------------

--------------------------------------------------

--------------------------------------------------

--------------------------------------------------

--------------------------------------------------

--------------------------------------------------

--------------------------------------------------

--------------------------------------------------

--------------------------------------------------

--------------------------------------------------

------

>Cricetus_cricetus_AUT Cricetus cricetus mitochondrion, complete genome.

GCCTGTGTAGCTTAA--C------A-CAAAGCAAAGCACTGAAGATGCTT

AGATAAATTTCT---AATTTCACGGGCACA-AA-GGTTTGGTCCTGGCCT

TATAATTAATTGAAGGTAGAATTACACATGCAAATCTCCTTATACCAGTG

TCAAATCCCT---AGGAC-TTTACTATTAAGCTCTAAGGAGAGGGCATCA

AGCACATACA-----A-TAGCTAAAGACGCCTTGC-CTAGCCACACCCCC

ACGGGACTCAGCAGTGATAAAAATTAAGCA-ATAAACGAAAGTTTGACTT

AGTCATACCCT----TCAGGGTTGGTAAATTTCGTGCCAGCCACCGCGGT

CATACGATTAACCCAAACTAATTATAT-TC-GGCGTAAAACGTGTTATAA

ATAAG-------A-TAAAATAGGGTTAAAACCTAACCAATATGTGAAATT

TCATCGTTAG-AATTAAACTCAGTGACGAAAGTAACCCTAATTATAT-C-

--TATACACGATAGCTAAGACCCAAACTGGGATTAGATACCCCACTATGC

TTAGCCATAAACCTGAGAAGTTCAAC--AACAAAACTACTTGCCTGAGAA

CTACTAGCCACAGCTTAAAACTCAAAGGACTTGGCGGTACTTTATATCCA

CCTAGAGGAGCCTGTTCTATAATCGATAAACCCCGTTATACCTTACCACC

CCTTGCTAAT-TCAGCCTATATACCGCCATCTTCAGCAAACCTTCA-AAA

AGGATAAAAGTAAGCAAGAGCAT-AC--CCGTAAAAACGTTA-GGTCAAG

GTGTAGCCTATGAGGTGGG-AAGCAATGGGCTACATTT-------TCTTA

ATAAG------AACATTCACGCTATCCTTTATGAAACCTAAAGGA--CAA

AGGAGGATTTAGTAGTAAATT-AAGAGTAGAGAGCTTAATTGAATAG-AG

CAATGAAGTACGTACACACCGCCCGTCACCCTCCTCAAACTAGGCTGCCG

ATCTA-TATACCTAATAA-----CAATCAATAAGC--CTATG--AGAGGA

GATAAGTCGTAACAAGGTAAGCATACTGGAAAGTGTGCTTGGATTAACCA

TAACGTAGCTTAAACCCAATAAAGCATCTGGTCTACGCCCAGAAGATTCC

CTCCC-CACCGGACATTATGA-ACT-AACTCT-AGCCCTTACCTC-----

GATCAACTA-AACTATTTAACTCTGTTAACTAAAACATTTA---CTTA--

--TTTAAGTATTGGTGAAAGAAACT--T-TATTTGAGGAGCTATAGAAAC

AGTACCGCAAGGGAAAGA-TGAAAGA-TAAATTAAAAGTACTAAAAAGCA

AAGCTAAACCCTTGTACCTTTTGCATAA-TGAACTAACTAGAAAACCCCC

GGCTAAGAG-ACCTGCAGTCGGGAACCCCGAAACCAAACGAGCTACCTAA

GAGCAACTT---TATGAGTGCACCCGTCTATATGGCAAAATAGTGGGAAG

ACTTTTAGGTAGAGGTGAAAAGCCTAACGAGCTTGGTGATTGCTGGTTAC

CCAATAAA-GAATTTTAGTTCAACTTTAATCTTGCCCCAAGAA-A-CTAG

AA-TCCTAATGCAAGGTTAAAATATAGCCTAAAGAGGGACAGCCCTTTAG

GA--ATGGATACAACCTTTAGCAGTGAGTAA--GGTAAAATATTTGATAA

CCATAGTTGGCTTAAAAGCAGCCATCAATAAAGAAAGCGTTCAAGCTCAA

C------ACTTAAATCAGTCTAATTCCCAACA-T---CTCACCTACCT--

CCTGCAATTACAATTGGGTCAATCTATTAATAAAT-AGATGAGATACTGT

TAGTATGAGTAACAAGAATAATA--TTCTCCTTGCACAAGCATATAACAA

CCCGGA-TAA-CCATTTATAGTAAACACTCATATAAGTGCATACTCCCAC

TATTAAT--T-CACTTATTACC-CCATGTTAATCCAACACAGGTGTGC--

--TTAAAGGAAAGATTAAAAGAAGTAAGAGGAACTCGGCAAACACGAATC

CCGCCTGTTTACCAAAAACATAACCTCTAGCATTAAAAGTATTAGAGGCA

ATGCCTGCCCGGTGACTA-A-G----TTTAACGGCCGCGGTATCCTGACC

GTGCAAAGGTAGCATAATCACTTGTTCCGTAATTGGGGACTAGCATGAAC

GGCTAAACGAGGATTCAACTGTCTTTTACTTCTGATCAGTGAAATTGACC

TTCCCGTGAAGAGGCGGGAATATCATAATAAGACGAGAAGACCCTATGGA

GCTTTAGTCCAAC-TTCTTAGCCTCTACAATCAACAA-CCTA------CT

GGCTTAAATATA-AAGGCA---TAAGATAAGGACTTCGGTTGGGGTGACC

TCGGAGCATAAAAAAGCCTCCGAATGATT-ACAGCTAAGG--CCCACAAG

CCAAGGCACC---TCTA-ATCTTATTGATCCAA-ATATAATT-GATCAAC

GGACCAAGTTACCCTAGGGATAACAGCGCAATCCTATTCGAGAGTCCATA

TCGACAATTAGGGTTTACGACCTCGATGTTGGATCAGGACATCCCAATGG

TGTAGCAGCTATTAAAG-GTTCGTTTGTTCAACGATTAA-AGTCCTACGT

GATCTGAGTTCAGACCGGAGTAATCCAGGTCGGTTTCTATCTATTTACG-

-ATTTCTCCCAGTACGAAAGGACAAGAGAAATGGAGCCTCCTTATCAA-A

AGCGCCCCCAA-ACAATTCATGAGTACA-TCTCAATGAAGTAAATCCGTA

CAACC----CTC-CCCAAGACAAGGG--TTT-ATTAGGGTGGCAGAGCCC

GG-AAATTGCATAAGACTTAAACCCTTACTCCCAGAGGTTCAAATCCTCT

CCCTAATA------------------------------------------

------------------------------------------GTGCATTA

CATTAATATCTTGATATTACTACTCCCCATCTTAATCGCCATAGCCTTCT

TGACGCTGGTGGAACGAAAAATCTTAGGTTACATACAACTACGCAAAGGA

CCTAATATTGTAGGCCCCTTTGGTATCCTACAACCATTCGCGGATGCAAT

AAAACTCTTTATCAAGGAACCCCTTCGCCCCCTAGCCACCTCAACATCCC

TATTTATTATTGCACCCACACTATCGCTTACCCTAGCATTCAGTTTATGA

GTTCCCCTTCCCCTACCTCAACCCCTGATCAACATGAACATAGGAATACT

ATTTATCCTAGCCACCTCTAGCCTCTCCGTATATTCCATCTTATGGTCAG

GATGAGCCTCTAACTCAAAATACTCCATATTTGGGGCACTTCGAGCTGTA

GCCCAAACAATTTCATATGAAGTAACAATAGCCATTATCCTACTCTCAGT

ACTCCTCTTAAGCGGCTCTTTCACCCTCCAAACCCTCATTATCACACAAG

AACCCCTATGATTGCTCGCAACGTCCTGACCCCTAGCCATAATATGATAC

ATCTCCACTCTTGCCGAAACAAACCGAGCCCCATTTGACCTGACAGAAGG

AGAATCTGAGCTAGTATCAGGCTTCAATGTAGAATACGCCGCCGGCCCCT

TCGCACTATTCTTTATAGCAGAATACACTAACATTATCCTAATAAACGCA

CTGTCGACCATTGTCTTTCTAGGTCCCTTACATGACCCCCTTCATCCAGA

AATATATACTACAAACTTCATAGTAAAGACACTAGCTCTAACCTCCTTAT

TCCTATGAATTCGAGCTTCCTACCCACGATTCCGCTATGACCAACTAATA

CACCTCCTATGAAAAAACTTCCTACCCCTAACTCTAGCACTATGTACATG

ACATATTTCCATACCAATTTTCCTGGCAAGCATCCCCCCCTACACCTAAA

GAAACATGTCTGACAAAAGAGTTACTTTGATAGAGTAAATTATAGAGGTT

TA-AACCCTCTTGTTTCTAGAATAATAGGAATTGAACCTACACCTTAGAA

TTCAAAATTCTACGTACTACC---TATGTACTATACCCTATAT--C---A

GTAAGGTCAGCTAACT-AAGCTATCGGGCCCATACCCCGAAAATGTTGGT

TTAAACCCTTCCCGTACTAATTAACCCCTTCACCCTTCTCATCATTTATT

TCACTATTTTTTCAGGCCCAATAGTCACTATCTTCAGCACCAGCTTCTTT

CTTATATGAGTTGGCCTAGAAATAAACTTGCTAGCCATTATCCCCCTCAT

ACTTAACAAATCCCACCCACGATCTACAGAAGCGGCAACTAAATACTTTA

TCACCCAAGCCACAGCATCAATAATTTTTCTACTGTCCGTTATTCTAAAC

TACAAACAACTAGGAGTCTGATTTCCCCAACCTCAAACCAACAACTTAGC

AACCACCCTAACATTTATCTCACTAGCAATTAAACTAGGCCTCTCTCCTT

TTCACGCCTGACTACCAGAAGTAACCCAAGGAATCCCATTCAACACCGGC

TTACTCCTTCTCACCTGACAAAAACTAGCCCCCATATGTATCCTCTACCA

AATCTATCAATTCATTGATCCCGTAACCCTAATTATTTCAGCCCTTACCT

CAGTACTAATCGGCGCATGAGGGGGCCTCAACCAAACACAAACCCGAAAA

ATCATGGCCTACTCCTCTATTGCCCACATAGGATGAATAATCTCTATTCT

TCCTTATAACCCCTCCCTCACCATACTCAACCTTATCATTTATATTATAT

TGACACTAGCTGTATTCTTAATCTTACAGCCCCACTCCTTCTCGTCCATT

AAAACTGCCTCACTCCTATGAAATAAAACACCAACCATACTCCCCCTAAT

AGCCCTCCTTCTACTATCTATAGGAGGCCTACCGCCCCTCACAGGCTTCC

TACCTAAATGACTCATTATTACTGAAATATTAAAAAACTATAATCATATT

ATAGCTACAACAATAGCAATCACAGCCCTAATTAATCTATTCTTTTACAC

ACGCCTAATCTACTCCACCTCACTAACCCTATTTCCAACAAACAATAACT

CCAAAATATTTACCCACCAACCAAGCACAAAAAACTTCATCACCCTACCA

ACAGTTGCTATTCTAGCCACACTAACACTTCCCCTCTCCTCCC---TAAC

AAC---------AT----AGAAGTTTAAGATAAA--T-AAGTCCGAAGGC

CTTCAAAGCCTTCAGTAAACACTCAAGT-----TTAACTTCTGA------

---A-----TAAGGATTGTAAGCCTTCATCTTACAT----CTAATGAGTG

CAAATCAATTACTTTAT--TTTAAGCTAAATCCTTCA----------CTA

GATTGGCA--GGACTCAAA-CCTAC-GAAACTTTAGTTAACAGCTAAACA

CCCAAA---ACTGGCCTCAATCTA-GC-TCTCCCGCCTATCAGAAAGGG-

-----GGCGGGAGAAGCCTCAGTAGAG-TATGTTATCTACACCTTC--GA

-----------------------------ATTTGCAATTCGATG--TGAA

TA-TCACCTTAAGGC-T-------GGCAAAAAGAGGG----CTATAACCT

CTGTCTTTAGATTTACAGTCTAATGCTTAT-CTCAGCCATTTTACC----

-------TATGTTCATCAACCGCTGACTCTTTTCGACCAACCACAAAGAC

ATCGGAACCTTGTACTTAATATTTGGTGCTTGGGCAGGGATAGTAGGTAC

AGCCCTTAGCATTCTTATTCGAGCAGAGCTCGGCCAACCCGGAGCCCTGC

TAGGAGATGATCAAATCTACAATGTGGTTGTCACGGCCCACGCCTTTGTT

ATAATCTTCTTCATGGTAATACCAATTATGATTGGTGGGTTTGGGAACTG

ATTAGTCCCCCTAATAATTGGAGCTCCGGACATGGCATTTCCCCGCATAA

ATAACATAAGCTTTTGACTTTTACCCCCCTCCTTCCTTCTACTCTTAGCC

TCATCTATAGTCGAAGCCGGGGCCGGAACAGGATGAACAGTGTACCCCCC

ACTAGCTGGAAATTTAGCCCACGCAGGGGCCTCCGTGGATCTCACTATTT

TCTCACTTCATCTAGCTGGTGTCTCTTCCATCCTCGGGGCCATTAACTTC

ATTACCACAATCATTAACATAAAACCTCCTGCTATAACACAATATCAGAC

ACCACTATTTGTATGATCAGTCCTAATTACGGCTGTCCTACTACTTCTCT

CCCTGCCAGTCCTAGCAGCAGGTATTACCATATTACTTACAGACCGCAAC

CTAAACACAACATTCTTTGATCCTGCGGGAGGAGGAGACCCGATTCTATA

CCAACATCTATTCTGATTCTTCGGACACCCTGAAGTCTACATCCTCATCC

TCCCAGGCTTTGGTATTATCTCGCATATTGTTACGTACTACTCTGGAAAA

AAAGAACCCTTCGGTTATATAGGAATAGTATGAGCTATAATATCTATTGG

ATTCTTAGGCTTCATCGTCTGAGCCCACCACATATTTACAGTAGGATTAG

ATGTCGACACCCGAGCCTACTTCACATCAGCCACTATGATCATTGCCATT

CCCACTGGAGTTAAGGTCTTCAGCTGACTAGCCACACTCCACGGAGGAAA

TATCAAATGATCTCCTGCAATATTATGAGCCCTAGGTTTTATCTTCCTAT

TCACTGTAGGAGGGCTAACAGGAATCGTACTATCTAACTCCTCCCTAGAC

ATTGTTCTTCATGATACATATTATGTAGTAGCTCACTTCCACTACGTATT

ATCTATGGGAGCTGTATTTGCCATTATAGCAGGATTTGTACACTGATTCC

CACTATTTTCTGGTTACACCCTTGATGATGCGTGAGCTAAAGCCCATTTC

GCTATCATATTTGTAGGAGTAAACTTAACTTTCTTCCCTCAACACTTCCT

TGGTCTCTCAGGTATACCTCGACGCTACTCTGATTACCCAGACGCATACA

CTACATGAAATACCGTATCCTCAATAGGATCGTTCATCTCACTCACAGCA

GTGCTTGTAATAATTTTTATAATCTGAGAAGCCTTCGCTTCTAAACGAGA

AGTATTAATAGTAAGCTACCCATCTACTAACCTAGAATGACTTCACGGCT

GCCCCCCTCCTTACCACACATTTGAAGAGCCAACTTTCGTAAAAAT----

-TAAA-------------TAAGAAAGGAAGGATTCGAACCCCCTAAAATT

GGTTTCAAGCCAACTCCATAACCTCTATG-TCTTTCTCAAT-------GA

GATATTAGTAAATTCA-TTACATAACTTTGTCAAAGTTAAGTTATAGATT

A------A-AATCTATATATCTTAA-TGGCTTATCCCTCTCAACTTGGCC

TGCAAGACGCCACATCACCAATCATGGAGGAACTCATAAACTTTCACGAC

CATACCTTAATAATCGTTTTCCTTATTAGTTCTCTCGTCTTGTACATTAT

TACACTTATATTAACCACAAAGCTCACTCATACGAGCACGATAGATGCCC

AAGAAGTAGAAACCATTTGAACTATTTTACCAGCAGTAATTCTTATCCTC

ATTGCCCTCCCATCCCTTCGAATCCTATATATAATAGATGAAATCAACAA

CCCAGTGCTAACAGTAAAGACTATAGGCCATCAATGATACTGAAGCTACG

AATATACAGATTACGAAGACCTATGCTTTGACTCCTACATAATCCCAACT

ACTGACCTAAAACCAGGAGAAATACGATTACTCGAAGTTGACAATCGAGT

GGTTCTTCCCATGGAACTACCTATCCGTATGTTAATTTCCTCTGAAGATG

TTCTTCACTCGTGAACTGTACCCTCGCTAGGCCTTAAGACTGACGCAATC

CCAGGACGTCTGAATCAAGCGACCTTTTCATCAAATCGACCAGGATTATA

CTATGGGCAGTGCTCAGAGATTTGCGGGTCTAATCACAGCTTCATACCTA

TTGTACTTGAAATAGTACCTCTTAAGTGTTTCGAAAACTGGTCTATCTCA

ATGATCTAG--------------------------------TCC-CATTG

TGAAGCTC--AG--AGCGTTAACCTTTTAAGTTAAAATTAGAGACCTCGC

A-AT-CTC--CACAATGAAATGCCACAACTAGACACATCCACATGGTTTA

CAACAGTACTAGCCTCCA-CTGTCACACTTTTTACTCTCATCCAATTAAA

GCTTTCCCTACA--TAACTTTCCCTCCAACCCCTCCAGTAAA-CAAGTAA

CATACCCAAAACTAATTAACCCTTGAGAAACAAAATGAACGAAAATCTAT

TTGCCTCTTTCATTACCCCTTCCTTAGTAGGCCTACCTATTGTCATCGTC

ATTATTATGTTCCCTCTAACCCTAATAACAACCTCCGATCGTCTAATAAG

CAACCGCCTACACACCTTCCAACAATGACTCATTAAGCTAATTGCT-AAA

CAAATAATAATGATTCACTCCCCAAAAGGACGAACCTGATCCCTAATACT

GATCTCCCTAATTATATTTATTGGAAGCACTAACCTCCTGGGGCTGCTAC

CACACACATTTACACCCACAACTCAACTATCTATAAACTTAGGTATAGCC

GTACCACTCTGAGCTGGAACAGTCATCCTAGGCTTCCGACACAAGA-CAA

AACAGTCACTAGCACACTTTCTACCCCAAGGTACTCCTATCCCCTTAATC

CCAATACTCGTTATCATTGAGACAATTAGCCTATTTATTCAACCCATAGC

CCTCGCAGTTCGTCTAACAGCCAACATCACTGCAGGACACCTACTAATAC

ACCTAATTGGAGGAGCCACATTAGTCCTTACTTCCATTAGCCCTCCAACC

GCCATTATCACATTTATTATCCTCCTGCTATTAACCATTCTAGAGTTTGC

CGTAGCCCTAATTCAAGCTTACGTATTCACCCTGCTAGTAAGCCTATATC

TACACGATAACACCTAATGACCCACCAAACACACGCATATCATATAGTTA

ATCCAAGCCCATGACCTCTCACTGGAGCTCTTTCAGCCCTCCTATTGACC

TCAGGGTTAGTAATATGGTTCCACTATAACTCGTCCACCCTTTTATATGT

TGCACTGTTAACAAACTTATTAACTATGTATCAATGATGACGAGACATTG

TACGAGAAGGAACCTACCAAGGCCATCACACCCCTATCGTCCAAAAAGGC

CTCCGATATGGAATAATCCTATTTATCATTTCAGAAGTTTTCTTCTTCGC

TGGATTCTTCTGAGCCTTTTACCACTCAAGCTTAGTCCCTACTCACGACT

TAGGAGGTTGCTGACCTCCCACAGGTATTATTCCACTTAACCCCCTTGAA

GTACCCCTACTAAATACATCAGTCCTTCTAGCATCCGGAGTGTCAATTAC

ATGGGCACATCACAGCCTTATAGAGGGTAATCGAAAAAACATAAACCAGG

CCTTGCTTATTACAATTATCCTAGGAGCTTATTTCACTGCACTACAAGCA

TCAGAATACTTAGAAACCCCCTTCTCTATCTCAGACGGAGTTTACGGTTC

AACATTTTTTATAGCCACAGGCTTCCACGGACTTCACGTAATCATTGGGT

CAACCTTCCTCACAGTTTGTCTTCTACGACAACTAAAATTCCATTTTACA

TCTAAACACCACTTCGGGTTTGAAGCAGCAGCCTGATATTGACATTTCGT

AGACGTTGTTTGACTATTCCTTTATGTATCTATCTATTGATGAGGCTCAT

ACTTC-CTTAGTAT-AA-TCAGTACAACTGACTTCCAATCAGTTAGATCT

AGCCCCGACCTAGAAGGAAGTAATAAACCTCATCTTAGCTATCTCCATCA

ACATCCTACTGTCTGCTCTGCTTATCTCAATTGCCTTCTGATTACCACAA

CTTAATGTTTATGCAGAAAAAGCAGGGCCTTATGAATGCGGATTTGATCC

TATAAGCTCCGCCCGTCTGCCCTTCTCTATAAAATTTTTCCTAGTAGCTA

TTACCTTTCTCCTATTTGACTTAGAAATTGCCCTTCTCCTCCCTCTGCCC

TGAGCAATACAACTTACCACACTTCACACCACACTAATTATTTCCTTCGC

ATTTCTAACCATTTTGGGCCTAGGTTTAGCCTATGAATGAAGTCAAAAGG

GCCTAGAATGAACGGAGTAACTC--TGGTAATTAGTTTAA--TAAAAATA

AATGATTTCGACTCATTAGATTATGATACTAAC-CATAATTACCAG--CA

TGATAACCACAGTCTTTAATATTATCTTGGCCTTTATCTTCTCTCTCACT

GGAACCCTCATATTTCGATCCCACCTAATATCTACCCTCCTATGCTTGGA

GGGTATGATGCTGTCCCTATTTATTATAACGGCTATTACCTCGCTTAACA

CCCACTCCATAATTACATTCTCAATCCCCATTGTAATTCTAGTGTTCGCA

GCCTGTGAAGCAGCAGTGGGCTTAGCCCTACTGGTTAAAATTTCAACCAC

ATATGGTACAGACTATGTACAAAACCTGAACCTTCTACAATGTTAAAAAT

TATAATTCCCTCTTTAATATTACTCCCCCTGACTTGACTGTCACCTATAA

AAAATATATGGACCAACGTCACTATCTACAGCTTTATAATTAATATCTTC

GCCATACCAACACTGTGACAAATAAACGAAGCAGGTACAGGACTTTCCCC

AACATTTTACACCGACCCAATTTCCTCACCATTAGTCATACTAACAATCT

GACTCCTTCCATTAACACTCCTAGCCAGCCAAAATCACCTAAAAAGCGAA

ACTGATTTTAACAAAAAACTTTATGTCTCCTTATTAGTTACCCTACAACT

CCTCCTAGTTGCTACATTTACAGCAAGCGAACTGATCTTATTTTATATCT

TATTTGAAGCTACACTCATCCCCACCCTCATTATTATCACTCGATGGGGT

AATCAAACAGAACGTTTAAACGCAGGACTTTACTTCCTGTTCTACACCCT

AATTGGATCAATCCCACTACTAATCGCCCTCATCAACATCTATTCCTCAC

TAGGATCTTTAAACATTCTTCTCTTATCCCTCTCCTCTCACTCCCTTAGC

CCATC----------ATGATCAAACCATATCTTATGACTTGCATGTATGA

TAGCCTTTATAGTTAAAATACCCCTATATGGGGTCCACCTATGACTCCCC

AAAGCCCACGTAGAAGCCCCCATCGCAGGGTCTATAATCTTAGCAGCCAT

CTTACTAAAACTAGGCGGCTATGGGATAATTCGAATCTCAATTATCCTTG

ACCCCCTAACAAAATTTATGGCCTACCCCTTCATCCTACTCTCTTTATGA

GGTATGATCATAACTAGCTCCATCTGCCTGCGACAGACAGACCTAAAATC

TCTGATTGCCTACTCTTCAGTCAGCCACATGGCATTAGTGATCGCAGCCA

TTATAATCCAAACCCCGTGAAGCTTTATGGGTGCCTCAGCACTAATAATT

GCACACGGTCTCACATCTTCATTACTATTCTGCCTAGCTAATACTAATTA

CGAACGAACTCACACCCGAACCATAATTCTTGCACGAGGTCTTCAAATGG

CCTTCCCACTAATAGCTCTATTTTGACTCCTTGGAAACCTAGCAAACCTA

GCCTTACCCCCCTCAATCAATCTAGTCGGAGAACTTCTAATCTCAATCTC

CCTATTCTCCTGGTCTAATTTAACCATTATCCTAGTAGGAGTTAACATTC

TTATCACAGCCCTATATTCCCTATATATACTAATCACTACTCAACGGGGA

AAACTCTCAGCCCATGTCAAAAATCTCTACCCATCTCACACCCGAGAACT

TACTCTCATAATACTCCACATTGCCCCTCTGACCCTTCTAACAATTAACC

CTAAGCTAATCCTAGGGATCACCCTGTGTAAATATAGTTTATTAAAA-CA

TCAGACTGTGAATCTGAAGATATGAGT---TAAAACTCTTTTTTTACCAA

GAAAGAAT---GCAAGAGCTGCTCTCTCATGC-CCC-ATATATAACAGTA

TGGCTT--------------------------------------------

-------------------TCTTA--CTTTTATAGGATAGAAGTA-ATCC

GTTGGTCTTAGGAACCAAAAAC--TTGGTGCAACTCCAAATGAAAGTAAT

TAACT---CTATTGCCTCAACCATTATCCTGATCCTCCTACTCCTGGCCT

CCCCTATTGCAATAACCATAACTAACTACTATAAAACAACAAACTTCCCC

TCACACGTCACATCACTAATTAAACTCACCTTTTGACTAAGCCTCGTCCC

CATATGCATCCTATTCCACTCTAACACTGAACTCCTCGTCACCAATTGAC

ACTGACTAACTATTAATACCATCAAACTCTCTATTAACCTAAAGTTTGAC

TTCTTCTGTATCATCTTCCTACCTGTCGCCCTCTTTGTAACCTGATCAAT

CATAGAGTTCTCCTCCTGATATATACATTCAGACCCTCACCTAGACCGCT

TTATTAAGTATCTACTTATTTTCCTAATTACTATAATTATCCTCACCTCA

GCCAACAATATGTTCCAACTCTTTATTGGCTGGGAAGGCGTCGGGGTAAT

ATCCTTTCTCTTAATTGCTTGATGGTACGCTCGCCCAGATGCTAATACCG

CAGCCCTACAGGCAGTCTTATATAACCGTATTGGCGATATTGGCTTCATT

GCTACAATAGCATGACTATGCTTAAATAATAACTCATGGGAATTTCAACA

AATT---TTTATAATAGATAATAAAAGCCTGCTTCCTCTTCTAGGTCTCC

TTATTGCCGCAGCAGGAAAATCCGCCCAATTCGGACTTCACCCATGACTG

CCCTCAGCTATAGAAGGTCCCACCCCAGTATCAGCCTTACTTCACTCTAG

CACCATAGTAGTAGCAGGAATCTTCCTTCTAATCCGATTTCACCCCATAA

TA-TCTAGCAATAATACGGCCCTGACTATAATACTATGCATTGGATCGCT

AACCACCCTATTTACAGCAATTTGCGCTCTTACCCAAAATGACATTAAAA

AAATTGTAGCCTTCTCCACATCAAGCCAACTAGGTCTTATAATAGTTACC

CTCGGAATTAATCAACCCTACCTTGCCTTCCTACACATTTGCACCCACGC

ATTCTTTAAAGCCATACTATTTCTGTGTTCTGGATCAATCATCCACAGCC

TAAATGATGAACAAGATATCCGAAAAATAGGAGGCCTCCTAAAAGCTCTC

CCATTCACATCCTCATGCCTAACAATCGGCAGCTTAGCCCTAACAGGAGT

CCCCTTCCTCACAGGCTTTTACTCTAAAGACTTGATCATTGAAGCCGCAA

ATACCTGCTATACAAACGCCTGAGCCCTCCTAATTACACTCCTAGCCTCA

TCCCTAACAGCTGTCTATAGCATACGAATTATCTTCTTTGCCCTTATATC

AAAACCCCGATTCACTCCACTAATTACCCTAAATGAAAATAATCCCACAC

TTATTAACCCCATTAAACGCTTAGCCCTGGGCAGCATATTCGCCGGCTAC

ATTATCTTTTATAATATCCCAATCACCACTGTACAAGTCATAACAATGCC

CTGATATCTAAAAACTGCTGCTATAATCATTACAATCCTAGGATTTTCTA

TCGCCCTAGAACTCAACAATTTAACTCTCAACCTAAAAACAAACTTCCAA

ACAC-CACAAAACACATTCTCCACCTCTCTAGGCTTCTTCACACTTACAC

TACACCGCCTTCTCCCGGCAAAATACCTGACAATAAGCTTCAACACCACC

CTAGCCACCCTTGACCTCACCTGGTTAGAAAAAGCAATCCCCAAGACAAT

CTCACTTATTAAC---ACCCAAGCATCCCAAACAGTAGCAAATCAAAAAG

GCCTAATTAAACTGTATTTTTTATCTTTCTTAATTACTCTACTAACAGTT

CCTACCCTCATTAT-TAG--------------------------TTTCCC

CGAGTAATCTCAATAATGATAAACACCCCTATTAACAAGGTCCATCCAGA

AACCACCATTAATCAAGCCGAGCAACTATATAAAGCCGCAACACCAGCTC

CCCCTTCCCCTATTAACCCCAACTCGTCACTATCATAAACAATTCAACCT

CCCATGCTATCAAAACTCAACATCAATCCTA---------CGCCCTCATA

ATACTTCGTCACAAATATTATCCCTAATTCTATAAACATACTTATGACCA

TAATCCCAAATATTAGTCAACTAGATACTCATGTTTCCGGGTACTCCTCA

GCCGACATAGCAGTTGTATAACCAAAAACAACCATCATCCCCCCTAGATA

AATTAAAAACACCATTAACCCTAAAAAAGACCCCCCAAACCCTAATACCA

CCAAACAACCAGCACACCCACTAACAATTAAACACATCCCTCCATAAATA

GGTGAGGGCTTCAACGATGTGCCCAAGCAACCTAGAGCAATTAATGAGCT

TAAAATGAAAATAGATTCTGTCATAAT---TTCTA-CATAGATACTA-CC

TATGACCAATGACATGAAAAATCATCGTTGT-TATTCAACTATAGAAAC-

CCT----AATGACAATCATACGTAAGAGTCACCCACTAATAAAAATCATC

AACCATGCGTTCATTGATCTCCCTGCCCCCTCAAACATCTCATCCTGATG

AAACTTTGGATCCCTCCTCGGCCTTTGCCTTATTATTCAAATCTTAACCG

GTCTATTCCTAGCCATACATTACACATCAGACACCACAACTGCATTCTCA

TCAGTAACCCACATCTGCCGGGATGTTAACTACGGCTGACTAATTCGCTA

CCTCCACGCCAATGGAGCTTCAATATTCTTTATCTGCCTATTCCTCCACG

TAGGGCGGGGAATCTACTACGGATCATACGTTATAGTAGAGACATGAAAT

TTAGGTATTGTTCTCCTATTTACAGTAATAGCAACAGCATTTATAGGCTA

CGTACTCCCATGAGGCCAAATATCCTTTTGAGGCGCTACAGTCATTACTA

ACCTCCTATCAGCTATCCCTTACATCGGCACTACCCTAGTAGAATGAGTC

TGAGGTGGTTTCTCTGTAGACAAAGCCACCCTCACACGATTCTTCGCATT

CCACTTTATCCTCCCCTTCATCATCACAGCCCTAGTGGTAGTTCACCTTC

TATTTCTCCACGAAACCGGCTCCAACAACCCTACCGGCCTTAATTCAGAC

GCAGACAAAATTCCATTCCACCCCTACTACACAATCAAAGACCTTCTCGG

AGTATTCCTACTATTAATTGCTCTCATAATTTTAGTATTATTTTTCCCAG

ATATTCTCGGAGACCCAGATAATTACACTCCTGCAAATCCACTCAATACT

CCCGCACACATTAAACCAGAATGATACTTCCTATTCGCCTACGCAATCCT

ACGATCCATCCCCAATAAATTAGGAGGCGTACTTGCCCTTATTCTCTCTA

TCCTAATTCTCGCCACCCTGCCACTCCTTCACACATCAAAACTACGAACC

ATAATCTTCCGCCCTATCACACAAGCCCTTTACTGAACCCTAGTGGCAGA

CCTCCTCTTACTAACATGAATTGGGGGACAACCAGTAGAATACCCTTTCA

TCATTATTGGTCAAATCGCCTCCATTCTATACTTCGCTATTATTGTAATC

TTCATACCAATCGCAAGCATAATTGAAGACAGCGTCCTAAAATTTACTTA

ATGTCCTAATAGTATAAAT---ATTACTTTGGTCTTGTAAGCCAGAAATG

AAG---AATAAATCTTCTTAGGGCATG--CTCAAGAAAGAGGG-TTAACC

CTCACCGTCAGCACCCAAAGCTGAAATTCTTG--TTAAACTACTTCTTG-

--------------------------------------------------

--------------------------------------------------

--------------------------------------------------

--------------------------------------------------

--------------------------------------------------

--------------------------------------------------

--------------------------------------------------

--------------------------------------------------

--------------------------------------------------

--------------------------------------------------

--------------------------------------------------

--------------------------------------------------

--------------------------------------------------

--------------------------------------------------

--------------------------------------------------

--------------------------------------------------

--------------------------------------------------

--------------------------------------------------

--------------------------------------------------

--------------------------------------------------

--------------------------------------------------

--------------------------------------------------

--------------------------------------------------

------

>Cricetus_cricetus_RUS Cricetus cricetus mitochondrion, complete genome.

GCCTGTGTAGCTTAA--C------A-CAAAGCAAAGCACTGAAGATGCTT

AGATAAATTTCT---AATTTCACGGGCACA-AA-GGTTTGGTCCTGGCCT

TATAATTAATTGAAGGTAGAATTACACATGCAAATCTCCTTATACCAGTG

TCAAATCCCT---AGGAC-TTTACTATTAAGCTCTAAGGAGAGGGCATCA

AGCACATACA-----A-TAGCTAAAGACGCCTTGC-CTAGCCACACCCCC

ACGGGACTCAGCAGTGATAAAAATTAAGCA-ATAAACGAAAGTTTGACTT

AGTCATACCCT----TCAGGGTTGGTAAATTTCGTGCCAGCCACCGCGGT

CATACGATTAACCCAAACTAATTATAT-TC-GGCGTAAAACGTGTTATAA

ATAAG-------A-TGAAATAGGGTTAAAACCTAACCAATATGTGAAATT

TCATCGTTAG-AATTAAACTCAGTGACGAAAGTAACCCTAATTATAT-C-

--TATACACGATAGCTAAGACCCAAACTGGGATTAGATACCCCACTATGC

TTAGCCATAAACCTGAGAAGTTCAAC--AACAAAACTACTTGCCTGAGAA

CTACTAGCCACAGCTTAAAACTCAAAGGACTTGGCGGTACTTTATATCCA

CCTAGAGGAGCCTGTTCTATAATCGATAAACCCCGTTATACCTTACCACC

CCTTGCTAAT-TCAGCCTATATACCGCCATCTTCAGCAAACCTTCA-AAA

AGGATAAAAGTAAGCAAGAGCAT-AC--CCGTAAAAACGTTA-GGTCAAG

GTGTAGCCTATGAGGTGGG-AAGCAATGGGCTACATTT-------TCTTA

ATAAG------AACATTCACGCTATCCTTTATGAAACCTAAAGGA--CAA

AGGAGGATTTAGTAGTAAATT-AAGAGTAGAGAGCTTAGTTGAATAG-AG

CAATGAAGTACGTACACACCGCCCGTCACCCTCCTCAAACTAGGCTGCCG

ATCTA-TATACCTAATAA-----CAATCAATAAGC--CTATG--AGAGGA

GACAAGTCGTAACAAGGTAAGCATACTGGAAAGTGTGCTTGGATTAACCA

TAACGTAGCTTAAACCCAATAAAGCATCTGGCCTACGCCCAGAAGATTCC

CTCCC-CACCGGACATTATGA-ACT-AACTCT-AGCCCTTACCTC-----

GATCAACTA-AACTATTTAACTCTGTTAACTAAAACATTTA---CTTA--

--TTTAAGTATTGGTGAAAGAAACC--T-TATTTGAGGAGCTATAGAAAC

AGTACCGCAAGGGAAAGA-TGAAAGA-TAAATTAAAAGTACTAAAAAGCA

AAGCTAAACCCTTGTACCTTTTGCATAA-TGAACTAACTAGAAAACCCCC

GGCTAAGAG-ACCTGCAGTCGGGAACCCCGAAACCAAACGAGCTACCTAA

GAGCAACTT---TATGAGTGCACCCGTCTATATGGCAAAATAGTGGGAAG

ACTTTTAGGTAGAGGTGAAAAGCCTAACGAGCTTGGTGATTGCTGGTTAC

CCAATAAA-GAATTTTAGTTCAACTTTAATCTTGCCCTAAGAA-A-CTAG

AA-TCCTAATGCAAGGTTAAAATATAGCCTAAAGAGGGACAGCCCTTTAG

GA--ATGGATACAACCTTTAGCAGTGAGTAA--GGTAAAATATTTGATAA

CCATAGTTGGCTTAAAAGCAGCCATCAATAAAGAAAGCGTTCAAGCTCAA

C------ACTTAAATCAATCTAATTCCCAACA-T---CTCACCTACCT--

CCTGCAATTACAATTGGGTCAATCTATTAATAAAT-AGATGAGATACTGT

TAGTATGAGTAACAAGAATAATA--TTCTCCTTGCACAAGCATATAACAA

CCCGGA-TAA-CCATTGTTAGTTAACATCCATATAAGTGCATACTCTCAC

TATTAAT--T-CACTTATTACC-CCATGTTAATCCAACACAGGTGTGC--

--TTAAAGGAAAGATTAAAAGAAGTAAAAGGAACTCGGCAAACACGAATC

CCGCCTGTTTACCAAAAACATAACCTCTAGCATTAAAAGTATTAGAGGCA

ATGCCTGCCCAGTGACTA-A-G----TTTAACGGCCGCGGTATCCTGACC

GTGCAAAGGTAGCATAATCACTTGTTCCTTAATTGGGGACTAGCATGAAC

GGCTAAACGAGGATTCAACTGTCTTTTACTTCTGATCAGTGAAATTGACC

TTCCCGTGAAGAGGCGGGAATACCATAATAAGACGAGAAGACCCTATGGA

GCTTTAGTCCCAC-TTCTTAGCCTCTACAATCAACAA-CCTA------CT

GGCTTAAACATA-TAGGCA---TAAGATAAGGACTTCGGTTGGGGTGACC

TCGGAGCATAAAAAATCCTCCGAATGATT-ACAGCTAAGG--CCCACAAG

CCAAGGCACC---TCTA-ATCTTATTGATCCAA-ATATAATT-GATCAAC

GGACCAAGTTACCCTAGGGATAACAGCGCAATCCTATTCGAGAGTCCATA

TCGACAATTAGGGTTTACGACCTCGATGTTGGATCAGGACATCCCAATGG

TGTAGCAGCTATTAAAG-GTTCGTTTGTTCAACGATTAA-AGTCCTACGT

GATCTGAGTTCAGACCGGAGTAATCCAGGTCGGTTTCTATCTATTTACG-

-ATTTCTCCCAGTACGAAAGGACAAGAGAAATGGAGCCTCCTTATCAA-A

AGCGCCCCCAA-ACAATTCATGAGTACA-TCTCAATGAAGTAAATCCGTA

CAACC----CTC-CCCAAGACAAGGG--TTT-ATTAGGGTGGCAGAGCCC

GG-AAATTGCATAAGACTTAAACCCTTACTCCCAGAGGTTCAAATCCTCT

CCCTAATA------------------------------------------

------------------------------------------GTGCATTA

CATTAATATCTTGATACTACTACTCCCCATCTTAATCGCCATAGCCTTCT

TGACACTGGTGGAACGAAAAATCTTAGGTTACATACAACTACGCAAAGGA

CCTAATATTGTAGGCCCCTTTGGTATCCTACAACCATTCGCGGATGCAAT

AAAACTCTTTATCAAGGAACCCCTTCGCCCCCTAGCCACCTCAACATCTC

TATTTATTATCGCACCCACACTATCGCTTACCCTAGCATTCAGTTTATGA

GTCCCCCTTCCCCTACCCCAACCCCTGATCAACATGAATATAGGAATACT

ATTTATCCTAGCCACCTCTAGCCTCTCCGTATATTCTATCTTATGATCAG

GATGAGCCTCTAACTCAAAATACTCCATATTTGGGGCACTTCGAGCTGTA

GCCCAAACAATTTCATATGAAGTAACAATAGCCATTATCCTTCTCTCAGT

ATTCCTCTTAAGCGGCTCTTTCACCCTCCAAACCCTCATTATCACACAAG

AACCCCTATGATTGCTCGCAACGTCCTGACCCCTAGCCATAATATGATAT

ATTTCCACTCTTGCCGAAACAAACCGAGCCCCATTTGACCTGACAGAAGG

AGAATCTGAGCTAGTATCAGGCTTCAATGTAGAATACGCCGCCGGCCCCT

TCGCACTATTCTTTATAGCAGAATACACCAACATTATCCTAATAAACGCA

CTGTCGACCATCGTCTTTCTAGGTCCCTTACATGACCCCCTACATCCAGA

AATATTTACTACAAACTTCATAGTAAAAACACTAGCTCTAACCTCCTTAT

TCCTATGAATTCGAGCTTCCTACCCACGATTCCGCTATGACCAACTAATG

CATCTCCTATGAAAGAACTTCCTACCCCTAACTCTAGCACTATGTACATG

ACATATTTCCATACCAATTTTCCTGGCAAGCATCCCCCCTTACACCTAAA

GAAACATGTCTGACAAAAGAGTTACTTTGATAGAGTAAATCATAGAGGTT

CA-AACCCTCTTGTTTCTAGAATAATAGGAATTGAACCTACGCCTTAGAA

TTCAAAATTCTACGTACTACC---TATGTACTATATCCTATAT--T---A

GTAAGGTCAGCTAACT-AAGCTATCGGGCCCATACCCCGAAAATGTTGGT

TTAAACCCTTCCCGTACTAATTAACCCCTTTACCCTTCTCATCATCTACT

TCACTATTTTTTCAGGCCCAATAGTCACTATCTTCAGCACCAGTTTCTTT

CTTATATGAATTGGCCTAGAAATAAACTTGCTAGCCATTATCCCCCTCAT

ACTTAACAAATCCCACCCGCGATCTACAGAAGCGGCAACTAAATACTTTA

TTACCCAAGCCACAGCATCAATAATTTTCCTACTGTCCGTTATTCTAAAT

TACAAACAACTAGGAGTCTGATTTCCCCAACCTCAAACCAACAACTTAGC

AACCACCCTGACATTTATCTCACTAGCAATTAAACTAGGCCTCTCTCCTT

TTCACGCCTGACTACCAGAAGTAACCCAAGGAATCCCATTCAACACCGGC

TTACTCCTTCTCACCTGACAAAAACTAGCCCCCATATGTATCCTCTACCA

AATCTATCAATTCATTGATCCCGTGACCCTAATTATTTCAGCCCTTACCT

CAGTACTAATCGGCGCATGAGGGGGCCTCAACCAAACACAAACCCGAAAA

ATCATGGCCTACTCCTCTATTGCCCACATAGGATGAATAATCTCTATTCT

TCCTTATAACCCCTCCCTCACCATACTCAACCTTATCATTTATATTATAT

TGACACTAGCTGTATTCTTAATCTTACAGCCCCACTCCTTTTCGTCCATT

AAAACTGCCTCACTCCTATGAAATAAAACACCAACCATACTCCCCCTAAT

AGCCCTCCTTCTACTATCTATAGGAGGCCTACCGCCCCTCACAGGCTTCC

TACCTAAATGACTCATTATCACTGAAATATTAAAAAACTATAATCATATT

ATAGCCACAATAATAGCAATCACAGCCCTAATTAATCTATTCTTCTACAC

ACGCCTAATCTACTCCACTTCACTAACCCTATTTCCAACAAACAATAACT

CCAAAATATTTACCCACCAACCAAGCACAAAAAACTTCATCACCCTACCA

ACAGTTGCTATTCTAGCCACACTAACACTTCCCCTCTCCTCCC---TAAC

AAC---------AT----AGAAGTTTAAGATAAA--T-AAGTCCGAGGGC

CTTCAAAGCCTTCAGTAAACACTCAAGT-----TTAACTTCTGA------

---A-----TAAGGATTGTAAGCCTTCATCTTACAT----CTAATGAGTG

CAAATCAATTACTTTAT--TTTAAGCTAAATCCTTCA----------CTA

GATTGGCA--GGACTCAAA-CCTAC-GAAACTTTAGTTAACAGCTAAACA

CCCAAA---ACTGGCCTCAATCTA-GC-TCTCCCGCCTATCAGAAAGGG-

-----GGCGGGAGAAGCCTCAGTAGAG-TATGTTATCTACACCTTC--GA

-----------------------------ATTTGCAATTCGATG--TGAA

TA-TCACCTTAAGGC-T-------GGCAAAAAGAGGG----CTATAACCT

CTGTCTTTAGATTTACAGTCTAATGCTTTT-CTCAGCCATTTTACC----

-------TATGTTCATCAACCGCTGACTCTTTTCGACCAACCACAAAGAC

ATCGGAACCTTATACTTAATATTTGGTGCTTGGGCAGGGATAGTAGGTAC

AGCCCTTAGCATTCTTATTCGAGCAGAGCTCGGCCAACCCGGAGCCCTGC

TAGGAGATGATCAAATCTACAATGTGGTTGTCACGGCCCACGCCTTTGTT

ATAATCTTCTTCATGGTAATACCAATTATGATTGGCGGGTTTGGGAACTG

ATTAGTCCCCCTAATAATTGGAGCTCCGGACATGGCATTCCCCCGCATAA

ATAACATAAGCTTTTGACTTTTACCCCCCTCCTTCCTTCTACTCTTAGCC

TCATCTATAGTCGAAGCCGGGGCCGGAACAGGATGAACAGTGTACCCCCC

ACTAGCTGGAAATTTAGCCCACGCAGGGGCCTCCGTGGATCTCACTATTT

TCTCACTTCATCTAGCTGGTGTCTCTTCCATCCTCGGGGCCATTAACTTC

ATCACCACAATCATTAACATAAAACCTCCTGCTATAACACAATATCAGAC

ACCACTATTTGTATGATCAGTCCTAATTACGGCTGTCCTACTACTTCTCT

CCCTACCAGTCCTAGCAGCAGGTATTACCATATTACTTACAGACCGCAAC

CTAAACACAACATTCTTTGATCCTGCGGGAGGAGGAGACCCCATTCTATA

CCAACATCTATTCTGATTCTTCGGACACCCTGAAGTCTACATCCTCATCC

TCCCAGGCTTTGGCATCATCTCACATATTGTTACGTACTACTCTGGAAAA

AAAGAACCCTTCGGTTATATAGGAATAGTATGAGCAATAATATCTATTGG

ATTCTTAGGCTTCATCGTCTGAGCCCACCACATATTTACAGTAGGATTAG

ATGTCGACACCCGAGCCTACTTCACATCAGCCACTATAATCATTGCTATT

CCCACTGGAGTTAAAGTCTTCAGCTGACTAGCCACACTCCACGGAGGAAA

TATCAAATGATCTCCCGCAATATTATGAGCCCTAGGTTTTATCTTCCTAT

TCACTGTAGGGGGGCTTACAGGAATCGTACTATCTAACTCCTCCCTAGAC

ATTGTTCTTCATGATACATACTATGTAGTAGCTCACTTCCACTACGTATT

ATCTATGGGAGCTGTATTTGCCATTATAGCAGGATTTGTACACTGATTCC

CGCTATTTTCTGGTTACACCCTTGATGATGCATGAGCTAAAGCCCATTTC

GCTATCATGTTTGTAGGAGTAAACTTAACTTTCTTCCCTCAACACTTCCT

TGGTCTCTCAGGTATACCTCGACGCTACTCTGATTATCCAGACGCATACA

CTACATGAAATACCGTATCCTCAATAGGATCGTTCATCTCACTCACAGCA

GTGCTTGTAATAATTTTTATAATCTGAGAAGCCTTCGCTTCTAAACGAGA

AGTATTAATAGTAAGCTACCCATCTACTAACCTAGAGTGACTTCACGGCT

GCCCCCCTCCTTACCACACATTTGAAGAGCCAACTTTCGTAAAAAT----

-TAAA-------------TAAGAAAGGAAGGATTCGAACCCCCTAAAATT

GGTTTCAAGCCAACTCCATAACCTCTATG-TCTTTCTCAAT-------GA

GATATTAGTAAATTCA-TTACATAACTTTGTCAAAGTTAAGTTATAGATT

A------A-AATCTATATATCTTAA-TGGCTTACCCCTCTCAACTTGGCC

TGCAAGACGCCACATCACCAATCATGGAGGAACTCATAAACTTTCACGAC

CATACCTTAATAATCGTCTTCCTTATTAGTTCTCTCGTCTTGTATATTAT

TACACTTATACTGACCACAAAGCTCACTCATACAAGCACGATAGATGCCC

AAGAAGTAGAAACCATTTGAACTATTCTACCAGCAGTAATTCTTATCCTC

ATTGCCCTCCCATCCCTTCGAATCCTATATATAATAGATGAAATCAACAA

CCCAGTGCTAACAGTAAAGACTATAGGCCATCAATGATACTGAAGCTACG

AATATACAGATTACGAAGACCTATGCTTTGACTCCTACATAATCCCAACT

ACTGACCTAAAACCAGGAGAAATACGATTACTCGAAGTTGACAATCGAGT

GGTTCTTCCCATGGAACTACCTATCCGTATGTTAATTTCCTCTGAAGATG

TTCTTCACTCGTGAACTGTACCCTCGCTAGGCCTTAAAACTGACGCAATC

CCGGGACGTCTGAATCAAGCGACTCTTTCATCAAATCGACCAGGATTATA

CTATGGGCAGTGCTCAGAGATTTGCGGGTCTAATCACAGCTTCATACCTA

TTGTACTTGAAATAGTACCTCTTAAGTGTTTCGAAAACTGGTCTATCTCA

ATGATCTAG--------------------------------TCC-CATTG

TGAAGCTC--AG--AGCGTTAACCTTTTAAGTTAAAATTAGAGACCTCGC

A-AT-CTC--CACAATGAAATGCCACAACTAGACACATCCACATGGTTTA

CAACAGTACTAGCCTCCA-CTGTCACACTTTTTACTCTCATCCAATTAAA

GCTTTCCCTACA--TAACTTTCCCTCCAACCCCTCCAGTGAA-CAAGTAA

CATACCCAAAACTAATTAACCCTTGAGAAACAAAATGAACGAAAATCTAT

TCGCCTCTTTCATTACCCCTTCCTTAGTAGGCCTGCCTATTGTCATCGTC

ATTATTATGTTCCCTCTAACCCTAATAACAACCTCCAATCGTCTAATAAG

CAACCGCCTACACACCTTCCAACAATGACTCGTTAAGCTAATTGCT-AAA

CAAATAATAATGATTCACTCCCCAAAAGGACGAACCTGATCCCTAATACT

GATCTCCCTAATTATATTTATTGGAAGCACTAACCTCCTGGGGCTGCTAC

CACACACATTTACACCCACAACTCAACTATCTATAAACTTAGGTATAGCC

GTACCACTCTGAGCTGGAGCAGTCATCCTAGGCTTCCGACACAAGA-CAA

AACAGTCACTAGCACACTTTCTACCCCAAGGTACTCCTATCCCCTTAATC

CCAATACTCGTTATCATTGAGACAATTAGCCTATTTATTCAACCCATAGC

CCTCGCAGTTCGTCTAACAGCCAACATCACTGCAGGACACCTACTAATAC

ACCTAATTGGGGGAGCCACATTAGTCCTTACTTCCATTAGCCCTCCAACC

GCCATTATCACATTTATTATCCTCCTGCTATTAACCATTCTAGAGTTTGC

CGTAGCCCTAATTCAAGCTTACGTATTCACCCTGCTAGTAAGCCTATATC

TACATGATAACACCTAATGACCCACCAAACACATGCATATCATATAGTTA

ACCCAAGCCCATGACCTCTCACTGGAGCTCTCTCAGCCCTCCTATTGACC

TCAGGGTTAGTAATATGGTTCCACTATAACTCGTCCACCCTTTTATATGT

TGCACTGTTAACAAACTTATTAACTATGTATCAATGATGACGAGACATTG

TACGAGAAGGAACCTACCAAGGCCATCACACCCCTATCGTCCAAAAAGGC

CTCCGATATGGAATAATCCTATTTATCATTTCAGAAGTTTTCTTCTTCGC

CGGGTTCTTCTGAGCCTTTTACCACTCAAGCTTAGTCCCTACTCACGACT

TAGGAGGTTGCTGACCTCCCACAGGTATTATTCCACTCAACCCCCTTGAG

GTACCCCTACTAAACACATCAGTCCTTCTAGCATCCGGAGTGTCAATTAC

ATGGGCACATCACAGCCTTATAGAGGGTAATCGAAAAAACATAAACCAGG

CCTTGCTTATTACAATTATCCTAGGAGCTTATTTCACCGCACTACAAGCA

TCAGAATACTTAGAAACCTCCTTCTCTATCTCAGACGGAATTTACGGTTC

AACATTTTTTATAGCCACAGGCTTCCACGGACTTCACGTAATCATTGGGT

CAACTTTCCTCACAGTTTGTCTTCTACGACAACTAAAATTCCATTTTACA

TCCAAACACCACTTCGGGTTTGAAGCAGCAGCCTGATATTGACATTTCGT

AGACGTTGTTTGACTGTTCCTTTATGTATCTATCTATTGATGAGGCTCAT

ACCTC-CTTAGTAT-AA-TCAGTACAACTGACTTCCAATCAGTTAGATCT

AGCCCCGACCTAGAAGGAAGTAATAAACCTCATCTTAGCCATCTCCATCA

ACATCCTACTGTCTGCTCTGCTTATCTCAATTGCCTTCTGGTTACCACAA

CTTAATGTTTATACAGAAAAAGCAGGGCCTTATGAATGCGGATTTGATCC

TATAAGCTCCGCCCGTCTGCCCTTCTCTATAAAATTTTTCCTAGTAGCTA

TTACCTTTCTCCTATTTGATTTAGAAATCGCCCTTCTCCTCCCTCTGCCC

TGAGCAATACAACTTACCACACTTCACACCACACTAATTATCTCCTTCGC

ATTTCTAACCATTTTGGGCCTAGGTTTAGCCTATGAATGAAGTCAAAAGG

GCCTAGAATGAACGGAGTAACTC--TGGTAATTAGTTTAA--TAAAAATA

AATGATTTCGACTCATTAGATTATGATACTAGC-CATAATTACCAG--CA

TGATAACCACAGTCTTTAATATTATCTTGGCCTTTATCTTCTCTCTCACT

GGAACCCTCATATTTCGATCCCACCTAATATCTACCCTCCTATGCTTAGA

GGGCATGATGCTGTCCCTATTTATTATAACGGCCATTACCTCGCTTAACA

CCCACTCCATAATTATATTCTCAATCCCCATTGTAATCCTAGTGTTCGCA

GCCTGTGAAGCAGCAGTAGGCTTAGCCCTACTGGTTAAAATTTCAACCAC

ATACGGTACAGACTATGTACAAAACCTGAACCTTCTACAATGTTAAAAAT

TATAATCCCCTCTTTAATATTACTCCCCCTGACTTGACTGTCACCTATAA

AAAACATATGAACCAACGTCACTATCTACAGCTTTATAATTAATATCCTC

GCCATACCAACACTGTGACAAATAAACGAAGCAGGTACAGGACTTTCCCC

AACATTTTACACCGACCCAATTTCCTCACCATTAGTCATACTAACAATCT

GACTCCTTCCATTAACACTCCTAGCCAGCCAAAATCACCTAAAAAGCGAA

ACTGATTTTAACAAAAAACTTTATGTCTCCTTATTAGTCACCCTACAACT

CCTCCTAGTTGCTACATTTACAGCAAGCGAACTGATCTTATTTTATATCT

TATTTGAAGCTACACTCATCCCCACCCTCATTATTATCACTCGATGGGGC

AATCAAACAGAACGTTTAAACGCAGGACTTTACTTCCTGTTCTACACCCT

AATTGGATCAATCCCACTACTAATCGCCCTCATCAACATGCATTCCTCAC

TAGGATCTTTAAACATTCTTCTCTTATCCCTCTCCTCTCACTCCCTTAGC

CCATC----------ATGATCAAACCATATCTTATGACTTGCATGTATGA

TAGCCTTTATAGTTAAGATACCCCTATATGGGGTCCACCTATGACTCCCC

AAAGCCCACGTAGAAGCCCCCATCGCAGGGTCTATAATCTTAGCGGCCAT

CTTACTAAAACTAGGCGGCTATGGGATAATTCGAATCTCAATTATCCTTG

ACCCCCTAACAAAATTTATGGCCTACCCCTTCATCCTACTCTCTTTATGA

GGCATGATCATAACTAGCTCCATCTGCCTGCGACAGACAGACCTAAAATC

TCTGATTGCCTACTCTTCAGTCAGCCACATGGCATTAGTGATCGCAGCCA

TTATAATCCAAACCCCCTGAAGCTTTATGGGTGCCTCAGCACTAATAATT

GCGCACGGTCTCACATCTTCATTACTATTCTGCCTAGCTAATACTAATTA

CGAACGAACTCACACCCGAACCATAATTCTTGCACGAGGTCTTCAAATGG

CCTTCCCACTAATAGCTCTATTTTGACTCCTTGGAAACCTAGCAAACCTA

GCCTTACCCCCCTCAATCAACCTAGTCGGAGAACTTCTAATCTCAGTCTC

CCTATTCTCCTGGTCTAATTTAACCATTATCCTAGTAGGAGTTAATATTC

TTATCACAGCCCTATACTCCCTATATATACTAATCACTACTCAACGGGGA

AAACTCTCAGCCCATGTCAAAAATCTCTACCCATCTCACACCCGAGAACT

TACTCTCATAATACTCCACATTGCCCCTCTGACCCTTCTAACAATTAACC

CTAAACTAATCCTAGGGACCCCCCTGTGTAAATATAGTTTATTAAAA-CA

TCAGACTGTGAATCTGAAGATATGAGT---TAAAACTCTTTTTTTACCAA

GAAAGAAT---GCAAGAGCTGCTAACTCATGC-CCCCATATATAACAGTA

TGGCTT--------------------------------------------

-------------------TCTTA--CTTTTATAGGATAGAAGTA-ATCC

GTTGGTCTTAGGAACCAAAAAC--TTGGTGCAACTCCAAATGAAAGTAAT

TAACT---CTATTGCCTCTACCATTATCCTGATCCTCCTACTCCTGGCCT

CCCCTATTGCAATAACCATAACTAACTACTATAAAACAACAAACTTCCCC

TCACACGTCACATCACTAATTAAACTCACCTTTTGACTCAGCCTTATCCC

CATATGCATTCTATTCCACTCTAACACCGAACTCCTTGTCACCAATTGAC

ACTGACTAACTATTAATACTATCAAACTCTCTGTTAACCTAAAGTTTGAC

TTCTTCTGTATCATCTTCCTACCTGTCGCCCTCTTTGTAACCTGATCAAT

CATAGAGTTCTCCTCCTGATATATACATTCAGACCCCCACCTAGACCGCT

TTATTAAATACCTACTTATTTTCCTAATTACTATAATTATCCTCACCTCA

GCCAACAATATGTTCCAACTCTTTATTGGCTGGGAAGGCGTCGGGGTTAT

ATCCTTTCTCTTAATTGCTTGATGGTACGCTCGCCCAGATGCTAATACCG

CAGCCCTACAGGCAGTCTTATATAACCGTATTGGCGATATTGGCTTCATT

GCTACAATAGCATGACTATGCTTAAATAATAACTCATGGGAATTTCAACA

AATT---TTTATAATAGATAATAAAAGCCTGCTTCCTCTGCTAGGTCTCC

TTATTGCCGCAGCAGGAAAATCCGCCCAATTCGGACTTCACCCATGACTG

CCCTCAGCTATAGAAGGTCCCACCCCAGTATCAGCCCTACTTCACTCTAG

CACCATAGTAGTAGCAGGAATCTTCCTTCTAATCCGATTTCACCCCATAA

TC-TCTAGCAATAGTACGGCCCTGACTATAATATTATGCATTGGATCGCT

AACCACCCTATTTACAGCAATTTGCGCTCTCACCCAAAATGACATTAAAA

AAATTGTAGCCTTCTCCACATCAAGCCAACTAGGTCTTATAATAGTTACC

CTCGGAATTAATCAACCCTACCTTGCCTTCCTACACATTTGCACCCACGC

ATTCTTTAAAGCCATGCTATTTCTGTGTTCTGGATCAATTATCCACAGCC

TAAATGACGAACAAGATATCCGAAAAATAGGAGGCCTCCTAAAAGCTCTC

CCATTCACATCCTCATGCCTAACAATCGGCAGCTTAGCTCTAACAGGAGT

CCCCTTCCTCACAGGCTTTTACTCTAAAGACTTAATCATTGAAGCCGCAA

ATACCTGCTATACAAACGCCTGAGCCCTCCTAATTACACTCCTAGCCACA

TCCCTAACAGCTGTCTATAGCATACGAATTATCTTCTTTGCCCTCATATC

AAAACCCCGATTCACTCCACTAATTACCCTAAATGAAGATAACCCCACAC

TTATTAACCCCATTAAGCGCTTAGCCTTGGGCAGCATATTCGCCGGCTAC

ATTATCTTTTATAATATCCCAATCACCACTGTACAAGTCATAACAATGCC

CTGATATCTAAAAACTGCTGCTATAATCATTACAATCCTAGGATTTTCTA

TCGCCCTAGAACTCAATAATTTAACTCTCAACCTAAAAACAAGCTTCCAA

ACAC-CACAAAACGCATTCTCCACCTCTCTAGGCTTCTTCACACTTACAC

TACACCGCCTTCTCCCGGCAAAATACCTGACAATAAGCTTCAACACCACC

CTAGCCACCCTTGACCTCACCTGGTTAGAAAAAGCAATCCCTAAGACAAT

CTCACTTATTAAC---ACCCAAGCATCCCAAACAGTAGCAAATCAAAAAG

GCCTAATTAAACTGTATTTTTTATCTTTCTTAATTACTCTACTAACAATT

CCTATCCTCATTAT-TAG--------------------------TTTCCC

CGAGTAATCTCAATAATGATAAGCACCCCCATTAACAAGGTCCATCCAGA

AACCACCATTAATCAAGCCGAGCAACTATATAAAGCCGCAACACCAGCTC

CCCCTTCCCCTATTAACCCCAACTCGTCACTATCATAAACAATTCAACCT

CCCATGCTATCAAAACTCAACATCAACCCTA---------CGCCCTCGTA

ATACTTCGTCACAAATATTATCCCTAATTCTATGAGTATACTTATGACCA

TAATCCCAAATACCAGTCAACTAGATACTCATGTTTCCGGGTACTCCTCA

GCCGACATAGCAGTTGTATAACCAAAAACAACCATCATCCCCCCTAGATA

AATTAAAAACACCATTAACCCTAAAAAAGACCCCCCAAACCCTAATATCA

CCAAACAACCAGCACACCCACTAACAATTAAACACATCCCCCCATAGATA

GGTGAGGGCTTCAACGATGTGCCCAAACAACCTAGAGCAATTAATGAGCT

TAAAATGAAAATAGATTCTGTCATAAT---TTCTA-CATAGATACTA-CC

TATGACCAATGACATGAAAAATCATCGTTGT-TATTCAACTATAGAAAC-

CCT----AATGACAATCATACGTAAGAGTCACCCACTAATAAAAATCATC

AACCATGCGTTCATTGATCTCCCTGCCCCCTCAAACATCTCATCCTGATG

AAACTTTGGATCCCTCCTCGGCCTTTGCCTTATTATTCAAATCTTAACCG

GTCTATTCCTAGCCATACATTACACATCAGACACCACAACTGCATTCTCA

TCAGTAACTCACATCTGCCGGGATGTTAACTACGGCTGACTAATTCGCTA

CCTCCACGCCAATGGAGCTTCAATATTCTTTATCTGCCTATTCCTCCACG

TAGGGCGGGGAATCTACTACGGATCATACGTTATAGTAGAGACATGAAAT

TTAGGTATTGTTCTCCTATTTACAGTAATAGCAACAGCATTTATAGGCTA

CGTACTCCCATGAGGCCAAATATCCTTTTGAGGCGCTACAGTTATTACTA

ACCTCCTATCAGCTATCCCTTACATCGGCACTACCCTAGTAGAATGAATC

TGAGGTGGTTTCTCTGTAGACAAAGCCACCCTCACACGATTCTTCGCATT

CCACTTTATCCTCCCCTTCATCATCACAGCCCTAGTGGTAGTCCACCTTC

TATTCCTCCACGAAACCGGCTCCAACAACCCTACCGGCCTTAATTCAGAC

GCAGACAAAATTCCATTCCACCCCTACTACACAATCAAAGACCTTCTCGG

AGTATTCCTACTATTAATTGCTCTCATAATTTTAGTATTGTTTTTCCCAG

ATATTCTCGGAGACCCAGATAATTACACTCCTGCAAATCCACTTAATACT

CCCGCACACATTAAACCAGAATGATACTTCCTATTCGCCTACGCAATCCT

ACGATCCATCCCCAATAAATTAGGAGGCGTACTTGCCCTTATTCTCTCTA

TCCTAATTCTCGCCACCCTGCCACTCCTTCACACGTCAAAACTACGAACC

ATAATCTTCCGCCCTATCACACAAGCCCTTTACTGAACCCTAGTAGCAGA

CCTCCTCTTACTAACATGAATTGGGGGCCAACCAGTAGAATACCCTTTCA

TCATTATTGGGCAAATCGCCTCCATTCTATACTTCGCTATTATTGTAATC

TTCATACCAATCGCAAGCATAATTGAAGACAGCGTCCTAAAATTTACTTA

ATGTCCTAATAGTATAAAT---ATTACTTTGGTTTTGTAAGCCAGAAATG

AAG---AATAAATCTTCTTAGGGCACG--CTCAAGAAAGAGGG-TTAACC

CTCACCGTCAGCACCCAAAGCTGAAATTCTTG--TTAAACTACTTCTTG-

--------------------------------------------------

--------------------------------------------------

--------------------------------------------------

--------------------------------------------------

--------------------------------------------------

--------------------------------------------------

--------------------------------------------------

--------------------------------------------------

--------------------------------------------------

--------------------------------------------------

--------------------------------------------------

--------------------------------------------------

--------------------------------------------------

--------------------------------------------------

--------------------------------------------------

--------------------------------------------------

--------------------------------------------------

--------------------------------------------------

--------------------------------------------------

--------------------------------------------------

--------------------------------------------------

--------------------------------------------------

--------------------------------------------------

------

>Mesocricetus_auratus Mesocricetus auratus mitochondrion, complete genome.

GTTGATGTAGCTTAA--T------A-CAAAGCAAAGCACTGAAAATGCTT

AGATGGATTTTTTTCAATCCCATAAACATA-AAAGGTTTGGTCCTAGCCT

TATAGTTAGTTAGAGGTAGAGTTACACATGCAAATCTCTATAAACCAGTG

TCAAATCCCT---AGG-T-TTTACT-TTAAACCCTAAGGAGAGGGTATCA

AGCACATACACATATA-TAGCTAAAGACACCTTGC-CTAGCCACACCCCC

ACGGGACTCAGCAGTGATAAAAATTAAGCC-ATAAACGAAAGTTTGACTT

AGTCATACCTC---ATCAGGGTTGGTAAATTTCGTGCCAGCCACCGCGGT

CATACGATTAACCCAAACTAACTATTC-TCCGGCGTAAAATGTGTTTTTA

TTACG-------AACATAATAGAATTAAAACCCAACTAATATGTGAAAAT

TCATTGTTGG-ACTTAAAATCAATAACGAAAGTAATTCTAATTATAT-T-

--AATACACGATAGCTAAGATCCAAACTGGGATTAGATACCCCACTATGC

TTAGCCCTAAACCTAAGTGATTAAAT--AACAAAATCACTTGCCTGAGAA

CTACTGGCCACAGCTTAAAACTCAAAGGACTTGGCGGTACTTTATATCCA

TCTAGAGGAGCCTGTTCTATAATCGATAAACCCCGTTATACCTTACCACC

CCTTGCTAAT-TCAGCCTATATACCGCCATCTTCAGCAAACCTTAA-AAA

AGAACAAGAGTAAGCAAGAGAAT-AC--CCATAAAAACGTTA-GGTCAAG

GTGTAGCCTATGGGCTGGG-AAGTAATGGGCTACATTT-------TCTTC

TAAAG------AACAGTTACGCTATCCTCTATGAAACTTAGAGGA--CAA

AGGAGGATTTAGTAGTAAATT-AAGAATAGAGAGCTTAATTGAATAG-AG

CAATGAAGTACGTACACACCGCCCGTCACCCTCCTCAAATTAAGCTAACT

GTTAACTATACCTAATAT-----TGACTCATAAAC--TTATG--AGAGGA

GATAAGTCGTAACAAGGTAAGCATACTGGAAAGTGTGCTTGGACTAACCA

TAAGGTAGCTTAAACCCCATAAAGCATCTGGTCTACACCCAGAAGATTCC

ACATC-CAATGGACATTATGA-ACT-AACTCT-AGCCCTCATTTT-----

TTCAATCTA-TAAAATTTTAACTTACAAATTAAAACATTCA---CTAA--

--AAGAAGTATTGGAGAAAGAAACT--T-T--TTAAGGAGCCATAGAGAT

AGTACCGTAAGGGAAAGA-TGAAAGA-CTTGTTAAAAGTAAATAAAAGCA

AAGATTAAACCTTGTACCTTTTGCATAA-TGAACTAACTAGAAATCTTCT

AACTAAGAG-AACTTTAGCTAGAGACCCCGAAACCAAGCGAGCTACCCAA

GAGCAACTT---TAAGAGTTAACCCGTCTATGTAGCAAAATAGTGGGAAG

ACTTCTGGGTAGAGGTGAAAAGCCTAACGAGCCTGGTGATAGCTGGTTAC

CCAATAAA-GAATTTTAGTTCAACTTTAAGCCTACCATAAACT-T-TACT

AA-CACAAATGTAAGCTTAAAATATAGCCTAAAGAGGGACAGCTCTTTAG

GA--ACGGAAACAACCTCTAATAGTGAATAA--GACAATCAATCAACTAA

CCATAGTTGGCTTAAGAGCAGCCATCAATAAAGAAAGCGTTCAAGCTCAA

C------ACTTAATAGTACATAATACCCTAAA-T---AAAATCTATTT--

CCTATCTTCATAACTGGGTTAATCTATTAGCTAAT-AGAAGCAATACTGT

TAGCATGAGTAACAAGAATATTA--TTCTCCACGCACAAGCCTATAACAA

CCCGGA-TAA-CCATTGTTAGTTAACAAAT-TATAAGTACATCACCCACT

TATAAAA--AGTACTTATTATC-CAATGTTAGTCCAACACAGGTGTGC--

--TAAAAGGAAAGATTAAAAGAAATAAAAGGAACTCGGCAAACATGAATC

CCGCCTGTTTACCAAAAACATAACCTCTAGCATTATAAGTATTAGAGGCA

ATGCCTGCCCAGTGACTA-A-G----TTAAACGGCCGCGGTATCCTGACC

GTGCAAAGGTAGCATAATCACTTGTTCCTTAATTAGGGACTAGTATGAAA

GGCTAAACGAGGGTTCAACTGTCTCTTATTTCCAATCAGTGAAATTGACC

TTCCCGTGAAGAGGCGGGGATATAATAATAAGACGAGAAGACCCTATGGA

GCTTTAATCTCAC-AACTTAATTTTAATAACAAACAG-TCTA------CT

GACTTTAAAAAACTAAATA---TAAGTTGTAGATTTCGGTTGGGGTGACC

TCGGAGAATAAAAAAACCTCCGAATGATT-ATAACCTAGG--CTTACAAG

CCAAAGTACAAAATATA-ATCTTATTGACCCAA-ACTAATTT-GATCAAC

GGACCAAGTTACCCTAGGGATAACAGCGCAATCCTATTCAAGAGTCCATA

TCGACAATTAGGGTTTACGACCTCGATGTTGGATCAGGACATCCCAATGG

TGTAGCAGCTATTAAAG-GTTCGTTTGTTCAACGATTAA-AGTCCTACGT

GATCTGAGTTCAGACCGGAGTAATCCAGGTCGGTTTCTATCTATTTACG-

-ATTTCTCCCAGTACGAAAGGACAAGAGAAATGGGACCTCCTTAATAC-A

AGTGCCCCTGA-TTAATTAATGAAACCA-TCTCAATATAGTAAACACGTA

CTATA----CCCACCCTAGACAAGGG--TTT-ATTAGGGTGGCAGAGCCC

GG-AAATTGCGTAAGACTTAAAACCTTGTTTTCAGAGGTTCAAATCCTCT

CCCTAATA------------------------------------------

------------------------------------------GTGCATCT

AATTAACATTCTAATACTACTTATCCCAATTCTAATCGCAATAGCCTTTC

TTACCCTAGTAGAACGAAAAATCTTAGGATATATACAACTACGAAAAGGC

CCTAACATCGTTGGCCCATATGGCATCCTCCAACCATTTGCCGACGCTAT

AAAACTATTTATTAAAGAACCTTTACGTCCTTTAGCCACCTCAACATCCC

TATTTATTATTGCCCCCACACTATCACTTATACTTGCATTTAGCCTATGA

ATTCCTCTACCCATACCTCACCCTTTAATTAATATAAATATAGGCATATT

ATTTATCTTAGCTATCTCCAGCCTATCCGTATATTCTATCTTATGGTCAG

GCTGAGCCTCTAATTCAAAATATTCTATATTTGGAGCCTTACGTGCAATC

GCACAAACAATCTCCTACGAAGTAACTATAGCAATCATCTTACTATCAGT

CCTTCTAATAAATGGCTCATTTTCCTTACAATTCCTTATTACTACACAAG

AGCACACATGACTCTTACTTATATCCTGACCCTTAGCTATAATATGATTT

ATCTCAACCCTAGCAGAAACCAATCGAGCACCCTTTGATTTAACCGAAGG

TGAATCTGAACTAGTTTCAGGGTTTAATGTAGAGTATGCCGCAGGACCAT

TCGCCCTATTCTTTATAGCTGAGTACACTAATATTATTCTAATAAATGCT

CTATCAACAATTGTATTCTTAGGACCAATCCATAGCCTATATCTACCACA

TGTTTATACAACCGACTTTATAATCAAAACATTGATACTAACTTCCTTAT

TTTTATGAATTCGAGCATCTTACCCACGATTTCGCTATGATCAACTTATA

CACCTCCTCTGAAAAAACTTCTTACCCCTGACACTAGCTTTATGTACATG

ACACATTTCAATTCCTATCTTCATAGCAAGTATTCCACCCTACACTTA--

GAAATATGTCTGACAAAAGAGTTACTTTGATAGAGTAAATTATAGAGGTT

CA-AATCCTCTTATTTCTAGGACAGTAGGAATTGAACCCACCTCTTAGAA

TTCAAAATTCTATGTAATACC---AATATACTTCATCCTAAA-------A

GTAAGGTCAGCTAATC-AAGCTATCGGGCCCATACCCCGAAAATGTTGGT

TTAAACCCTTCCCGTACTAATTAACCCTATTACCCTCTTAATTATTTACT

TTACTATTCTCTCAGGCCCAATAGTCACAATACTCAGCAACAACTTCTTT

CTTATATGAATTGGTCTAGAAATAAACCTTCTAGCCATTATTCCAGTTAT

AACTAACAAGTCAAACCCACGAACTACAGAAGCAGCTACAAAATATTTCA

TCACCCAGGCTACAGCTTCCATAATTTTCCTTCTTTCTATTATCATAAAC

TATAAACAATTAGGAACATGAACAATTCAACCTCAAACTAGTAGCCTCAC

ATCTACACTAATCTTTATCTCCTTAGCAATCAAATTAGGTCTATCACCCT

TCCACGCTTGACTACCAGAAGTAACTCAAGGCATTCCTCTAAGCACAGGA

CTCCTGCTACTAACTTGACAAAAACTAGCTCCTCTATCTATCTTATTTCA

AGTCTACGAACTAATTAACCCACATGTACTAATCATGTCAGCTTGCGCCT

CAGTAATAATTGGAGCATGAGGAGGCCTCAATCAAACTCAAACTCGAAAA

ATCATAGCTTATTCCTCCATCGCCCACATAGGCTGAATAATCTCGATTCT

ACCCTATAATCCATCCCTTACCATTATTAACCTTATCATTTATATTCTAC

TAACACTAGCCATATTCTCAATTCTTCACCCCCACTCATTTTTCTCTATC

AAATCAACATCACTTCTATGAAATAAATCACCTATTATACTTCCCTTAAT

GTCCTCTATCCTCTTATCCATAGGAGGGTTGCCCCCTCTTACCGGCTTTC

TACCTAAATGACTTATCATTACAGAACTCCTAAAAAACAACAACCAACTA

CTAGCTTCAATCATAGCAATTCTAGCCCTAGTCAATCTATTCTTTTATAC

TCGCTTAATTTACTCAACTTCACTTACTACATTCCCTTCCACTAATAACT

CTAAAATATTTAACCACCAAAAAAACATAAAAAAATACCCAACCCTACCA

CTAACTGCAATCACAAGCACGCTAATACTCCCCCTTCTACCCC---TAAT

ATT---------AT----AGAAGTTTAGGATAAA--T-AAGTCCGATGGC

CTTCAAAGCCCTTAGTAAACCCTTA-GT-----TTAACTTCTGC------

---A-----TAGAGATTGTAAGACTATACCTTACAT----CTAATGAATG

CAAATCAATTGCTTT-T--ATTAAGCTAAATCCCCC------------TA

GATTGGCA--GGATTCAAA-CCTAC-AAAATTTTAGTTAACAGCTAAACA

CCCAACT--ACTGGCTTCAATCTA-CT-TCTCCCGCCTATCAGAAAGGG-

-----GGCGGGAGAAGCCTTAGTAGAA--ATATTATCTACACCTTC--GA

-----------------------------ACTTGCAATTCGATA--TGAT

TA-TCACCTTAAGGCCT-------GGTAAAAAGAGGG----TTATAACCT

CTGTGCTTAGATTTACAGTCTAATGCTATG-CTCAGCCATTCTACC----

-------TATGTTCATTAATCGCTGGTTATTTTCAACTAATCATAAAGAT

ATTGGAACACTGTATTTAATATTTGGGGCCTGAGCAGGTATAGTGGGCAC

TGCTCTTAGCATCTTAATTCGAGCAGAGCTTGGTCAACCTGGGGCTTTAC

TAGGTGATGATCAAATCTATAATGTAGTTGTAACAGCTCATGCATTTGTT

ATAATCTTCTTTATGGTTATACCAATAATAATTGGGGGATTTGGAAACTG

ACTTGTACCACTAATAATTGGAGCCCCTGATATGGCATTCCCTCGAATAA

ATAACATAAGTTTCTGACTTCTACCCCCCTCATTCCTTCTTTTATTAGCA

TCATCTATAGTCGAAGCGGGAGCTGGAACTGGTTGAACAGTCTACCCCCC

ACTAGCAGGAAACTTAGCACATGCTGGAGCATCCGTAGATCTTACTATCT

TTTCACTCCATTTGGCTGGAGTATCTTCAATTTTAGGGGCTATCAATTTT

ATTACTACAATTATTAACATAAAACCCCCAGCCATAACACAGTATCAAAC

CCCCCTATTTGTCTGATCAGTATTAATCACAGCTGTTTTACTATTATTAT

CTCTACCTGTCCTAGCTGCTGGAATTACAATGCTACTTACAGATCGTAAT

TTAAATACAACTTTCTTCGATCCTGCCGGAGGAGGAGATCCAATTCTATA

CCAACACCTATTTTGATTCTTTGGTCATCCAGAAGTATACATCTTAATCC

TACCGGGCTTCGGAATTATTTCACACATTGTTACTTATTACTCCGGAAAA

AAAGAACCTTTTGGCTATATAGGTATAGTATGAGCAATAATATCAATTGG

ATTCCTGGGCTTTATTGTTTGAGCTCATCATATATTTACAGTAGGACTTG

ACGTAGACACACGAGCCTATTTTACATCAGCCACTATAATTATTGCAATC

CCAACTGGAGTAAAAGTATTTAGCTGACTAGCAACGCTTCATGGAGGAAA

CATCAAGTGATCACCAGCTATACTATGAGCCCTAGGGTTTATCTTCTTAT

TTACAGTTGGAGGTCTAACAGGTATTGTCTTATCAAACTCATCCTTAGAT

ATTGTATTACATGATACTTATTACGTAGTTGCCCATTTCCACTACGTTCT

ATCAATAGGAGCTGTATTTGCTATTATAGCAGGCTTTGTTCACTGATTCC

CTCTATTTTCAGGCTATACTCTTGACGATACATGAGCAAAAGCCCATTTC

ATTATTATATTTGTTGGGGTAAACATAACATTCTTCCCTCAACACTTCTT

AGGGCTAGCAGGAATACCACGACGCTATTCTGACTACCCAGATGCTTATA

CTATATGAAACACTGTGTCATCAATAGGATCTTTTATCTCACTAACAGCC

GTGCTTGTAATAATCTTTATAATCTGAGAGGCTTTCGCTTCTAAGCGAGA

AGTTCTCACAGTAGACTATTCCTCAACGAACCTAGAATGACTTCACGGTT

GTCCTCCCCCCTATCATACATTTGAAGAACCTACTTTCGTTAAAGTA-AA

ATAAA-------------TGAGAAAGGAAGGATTCGAACCCCCTAAAATT

GGTTTCAAGCCAACTCCATAACCTCTATG-TCTCTCTCAAT-------GA

GATATTAGTAAAATCA-TTACATAACTTTGTCAGAGTTAAATTATAGACT

A------A-TCTCTATATATCTTAAATGGCTTATCCTTCTCAATTAGGCT

TACAAGACGCCACATCACCTATTATAGAAGAATTAATAAACTTTCATGAT

CATACATTAATAATCGTCTTCCTTATTAGTTCTCTGGTCCTATATGTTAT

TACACTAATATTAACTACTAAGTTAACTCACACAAGTACAATAGATGCAC

AAGAAGTAGAAACAATCTGAACTATTCTACCTGCAGTAATTCTTATCCTT

ATTGCCCTACCGTCTTTACGAATTTTGTATATAATAGATGAGATTAACAA

CCCAGTATTAACAGTGAAAACTATAGGACATCAGTGATACTGAAGTTATG

AGTATACAGACTATGAAGACCTATGCTTTGACTCATATATAATCCCTACG

ACAGATCTAAAACCAGGAGAACTTCGATTACTAGAAGTTGACAATCGAGT

TGTACTCCCCATAGAACTACCGATTCGCATGCTAATCTCATCCGAAGACG

TTCTTCACTCATGAGCTGTCCCATCTCTTGGATTAAAAACAGATGCAATT

CCCGGACGACTAAATCAAGCTACTATTTCATCAAATCGACCAGGGTTATT

CTACGGTCAGTGTTCAGAAATCTGTGGGTCTAATCATAGCTTTATACCTA

TTGTTTTAGAATTAGTCCCTCTTAAATACTTTGAAAACTGATCCGTATCA

ATAGTCTAA--------------------------------ATC--ACTA

TGAAGCTC--AG--AGCGTTAACCTTTTAAGTTAAAATTAGAGACTTC-T

A-GT-CTC--CATGGTGAAATGCCACAACTAGATACATCTACATGATTTA

CTACTGTTCTAACTTCTA-CAATCACACTATTCATCCTAATTCAGTTAAA

AATTTCTTTACA--GAACTTTCCCAATAAACCATCAAACAAA-TATCTTA

AAACCCTTAAACTAAGTAACCCTTGAGAACTAAAATGAACGAAAATCTTT

TCGCCTCTTTCATTACCCCTACAATAATAGGTCTACCTGTTGTAATTATT

ATTATTATACTCCCATCCATAATACTAACATCTTCTAACCGTCTCTTAAG

CAACCGCTTCCATACATTTCAACAATGAGTAGTTAAAATAATTACC-AAA

CAAATAATATCAATTCATTCGCCAAAAGGACAGACTTGATGCCTAATACT

AGTTTCCCTAATTATCTTTATTGGCTCAACAAACCTCTTAGGCTTATTAC

CTCATACCTTCACCCCAACAACCCAACTATCAATAAATCTAGGAATAGCA

ATTCCACTATGAGCAGGAACAGTTTTCCTCGGATTTCGCCACAAAA-TAA

AAAGCACATTAGCCCATTTCCTACCGCAAGGAACACCTATTCCTCTTATT

CCTATACTCATTATTATTGAAACAATCAGCCTATTTATTCAACCCATAGC

CCTTGCAGTTCGACTAACAGCCAATATTACTGCAGGACATTTACTCATAC

ATCTTATCGGAGGGGCAACCCTAGTTTTAACCTCTATCAGTCCCCCAACA

GCTATAATTACATTTATCATTCTTGTACTCCTAACTATTCTAGAATTTGC

TGTTGCCCTAATTCAAGCATATGTGTTTACTCTACTAGTAAGCTTATATC

TACATGATAATACTTAATGACCCACCAAACTCATGCCTATCACATAGTTA

ACCCAAGCCCATGGCCCCTCACAGGAGCACTCTCTGCCCTCCTTCTTACC

TCAGGATTAGCAATATGATTCCATTATAACACTCTTACACTTCTTTATCT

AGGCCTCTTAACTAATATACTAACAATATACCAATGATGACGAGATATCG

TTCGTGAAGGTACCTACCAAGGCCACCATACACCCATCGTCCAAAAAGGC

CTTCGATATGGCATAATTCTCTTTATTGTCTCAGAAGTCTTCTTTTTCGC

TGGTTTCTTCTGAGCCTTTTATCACTCTAGCTTAGTTCCTACCCACGACC

TAGGAGGCTGCTGACCACCTACAGGAATTATTCCACTTAATCCACTTGAA

GTACCCTTACTTAACACATCTGTACTTTTAGCATCTGGTGTCTCAATTAC

ATGAGCTCATCATAGCCTTATAGAAGGTAAACGAAATAATATAAATCAAG

CTTTACTTATTACTATTGGCTTAGGAGTATATTTTACAGCTCTTCAAGCG

TCAGAATACTTAGAGACTTCCTTTTCTATTTCAGATGGTATCTATGGATC

AACATTTTTTATAGCAACAGGCTTTCATGGCCTTCATGTAATTATCGGAT

CCACATTCCTTATGGTGTGCCTTTTACGTCAACTAAAATATCACTTTACA

TCAAAACACCATTTCGGCTTTGAAGCAGCAGCTTGATACTGACACTTTGT

AGATGTCGTATGACTATTCCTCTACGTTTCTATCTATTGATGAGGGTCGT

ACTTT-CTTAGTAT-AT-TTAGTACAGTTGACTTCCAATCAACTAGGTCC

AACTATAATTTGGAAGAAAGTAATTAACTTAATCCTAGCAGTTTCAATCA

ACATAATACTCTCATTAATTTTAATTCTAGTTGCCTTCTGGCTTCCCCAA

CTTAATGTATATGCAGAAAAAGCAAGTCCCTATGAATGTGGTTTTGACCC

CATAAGCTCAGCTCGACTCCCTTTCTCTATAAAATTTTTTCTTGTAGCTA

TTACATTTCTTTTATTTGACTTAGAAATTGCTCTCCTCCTCCCCCTACCT

TGAGCCATACAATCTACAAACCTTAAAACAACTCTAATTATCTCTACAAT

ATTTCTATCTATTCTAGCGCTAGGCTTAGCCTATGAATGAAAACAAAAAG

GCCTTGAATGGACTGAATAGTT----GGTGATTAGTTTAA--CTAAAATT

AATGATTTCGACTCATTAGATTATGACATATCT-CATAATCACCAA--CA

TGACAACTACTCTATTTAATATTACATTAGCATTCGTATTTTCATTAATT

GGAACTCTTATGTTTCGCTCACACCTCATATCTACACTTCTATGCCTAGA

GGGAATAATATTAACCTTATTTATTATACTCACTATCACCTCATTAAACA

TCCACTCAATAATCACATATCCTATCCCTATTGTTATCCTAGTATTTGCG

GCATGCGAAGCAGCAGTTGGACTCGCCCTATTAGTTAAAGTATCCTCTAC

ATATGGAATAGACTATGTACAAAATCTTAACTTACTACAATGCTAAAAAT

TATTGTACCTTCATTAATATTACTACCATTGACATGATTTTCTAGTAGTA

AAAAAGTGTGAATCAACGTAACAGCCTATAGTTTTTTAATTAATATCATT

GCTATAACAACATTATGACAAAACAGCGACGGAACTTTAAACTTCTCCTC

CATATTTTCTATAGACTCCCTATCCTCACCTCTTACAGCCCTTACTATTT

GACTTCTACCATTAATACTCCTAGCTAGTCAAAAACATATTAAAAAAGAA

ACCGTACTTAATAAAAAGCTTTATATCTCCATACTTGTAACCTTACAAAT

TCTTCTAATTATGACATTTTCTGCTAATGAATTAATCATATTTTATATCC

TTTTTGAAGCTACTTTAATCCCAACATTAATTATTATTACCCGATGAGGA

AATCAAACAGAACGATTAAACGCAGGATTGTACTTCTTATTTTACACATT

AATTGGTTCCATTCCCCTTCTCATCGCTCTTATTTCAATTCAAAAATCCC

TAGGAACATTAAATATTCTACTTTTATCACTAAACACTTCAACACTAGAT

AACAA----------CTGGTCTAATTACATCCTATGATTAGCATGTATAA

TAGCTTTTATAATTAAAATGCCTCTATATGGAGTTCACTTATGACTACCC

AAAGCTCATGTAGAAGCTCCTATTGCAGGATCAATAATTCTAGCTGCAAT

TTTACTGAAACTAGGTGGTTACGGAATAATACGAGTATCAATTATCCTTG

ATCCTATAACAGAGACTATAGCATATCCCTTCATTCTTTTATCACTATGA

GGTATACTTATAACAAGTTCTATCTGCCTACGACAAACAGATCTAAAGTC

ATTAATCGCTTACTCTTCAGTAAGTCATATAGCACTAGTTATTGCAGCTA

TTATAATTCAAACTCCCTGAAGTTTTATAGGAGCCTCAGCATTAATAATC

GCACATGGACTCACATCATCACTATTATTCTGCCTAGCAAATACTAACTA

TGAACGAATCCATAGTCGAACTATAATTATAGCCCGAGGCCTTCAAACAG

CATTCCCTCTAATAGCAACTCTATGATTTCTAGGAAGCCTGGCAAACCTA

GCCTTACCCCCTTCAATTAACCTAATTGGAGAACTACTTATTACAATTAC

ACTATTCTCCTGATCACACCTGTCAATCATTCTTGTAGGTACAAATATCC

TAATTACAGCTCTATACTCACTCTATATACTTATCATAACCCAACGAGGA

AAACTTACATTTCATATAAACAACCTCCAACCTTCCTACACACGAGAGTT

AACCCTAATAATACTACACATCACACCTCTAATCCTACTTATCATGAACC

CTAAACTAATTCTAGGTCCCACATTATGTAGGCATAGTTTACTAAAA-TA

TTAGATTGTGGATCTGAAGATAGGAGA---TAAAACTCCTTACCTACCGA

GAATGTAT---GCAAGAGCTGCTAACTCCTGC-TACCATGTATAATAACA

TGGCTT--------------------------------------------

-------------------TCTTA--CTTTTATAGGATAGTAGTA-ATCC

GTTGGTCTTAGGAATCAAAAAC--TTGGTGCAACTCCAAATGAAAGTAAT

TAACC---CTATCACATCTGTAATCATCCTAATCTTTGTAGCTCTAATAC

TTCCTATTATATTATCCTCAACTAACATCTATAAAACCTATAGCTTCTCT

AATCACGCTACATCAATAATTAAGTACTCATTTATATTAAGCCTTATTCC

ACTATGCACTCTATTTTATTCCAATACCGAACTTCTAATTACCAGTTGAC

ACTGAGTAACTATCAATACAATTAAATTATCCATTAACCTTAAATTTGAC

TTCTTCTGTATCATTTTCCTATCAGTAGCCCTATTCGTCACCTGATCCAT

TATAGAATTTTCCTCATGATATATACATTCCGACCCCAACTTAAATCGAT

TTATTAAATACCTACTACTATTTCTTATTACCATAATTATTCTCACTTCA

GCTAATAACATGTTCCAACTATTCGTAGGCTGAGAAGGCGTGGGGATTAT

ATCTTTTCTACTAATCGGCTGATGATACGGACGATCTGATGCTAACACAG

CAGCACTACAAGCTATCCTCTATAATCGCATTGGCGACATCGGCTTTATC

GCTACAATAGCTTGACTGAGCCTAAACATAAATTCATGGGAATTTCAACA

AATC---TTTATAACAAATAACAACAGCATTATCCCGCTGCTAGGTCTTT

TAATTGCAGCCATAGGTAAATCAGCACAATTTGGCCTTCATCCATGACTT

CCATCAGCTATAGAAGGCCCCACTCCAGTTTCAGCATTACTCCACTCAAG

CACAATAGTGGTGGCAGGAATTTTCCTTTTAATTCGTTTTCATCCACTGA

TA-TCTAATAACAACATGGCCCTAACCATGATACTCTGCCTAGGATCAAT

TACCACATTATTTACAGCAATCTGTGCTCTAACTCAAAATGACATTAAGA

AAATTGTAGCCTTCTCTACATCAAGCCAGTTAGGACTTATAATAGTTACA

CTAGGAATCAATCAACCATATTTAGCATTCCTTCACATTTGTACTCACGC

TTTCTTTAAAGCCATGCTATTTATATGCTCTGGATCAATTATCCACAACC

TAAATGATGAACAAGACATCCGAAAAATAGGGGGTCTCTTAAAACCCCTA

CCATTTACATCCTCTTGTCTTATTATTGGAAGTTTAGCTTTAACAGGAGT

TCCATTTCTTACAGGTTTTTATTCAAAAGATCTAATCATTGAAGCCATAA

ACACGTGTTATACCAACGCCTGAGCCCTTCTAGTCACACTTCTAGCTACC

TCTCTAACAGCTGCTTACAGCCTACGAATTATCTTCTTTGCTCTAATATC

AAAACCTCGCTACCTACCCCTAATTACACTAAACGAAAATAACCCCAAAC

TTATAAACCCCATTAAACGCTTAGCCATAGGAAGTATATTTGCAGGATTT

ATCATGACATACAACATCCCAACTACATCTATTCAAGTAATAACTATACC

CTGATACTTAAAAACAACAGCAATCTTAGTCACTCTAATAGGATTCACTA

TCGCTTTAGAACTAAACAACCTAACATATAATCTAAAATCAAATACATCA

ACAC-TAACAAACTTATTCTCCACATCACTAGGGTATTTCACCCTTATCT

TCCACCGCTTATTACCCAAAAAATTCCTAACTATAAGCTTTAACACACCC

CTAAACATACTAGACTTAACATGACTGGAAACTTCTATCCCCAAAGCCAT

CTCATATATAAAT---CTCACAGCCTCCCAAATCCTTGCCAACCAAAAAG

GTTTAATTAAACTTTACTTCATATCCTTCCTCATTACTCTCCTTTCGATT

CCTATTTTACTAAT-TAG--------------------------TTTCCA

CGAGTAATCTCAATAATAATAAAAATACCCATAAATAAAGTTCAACCAGA

TACTACTATTAATCATGCAGAACAACTGTATAAAGCTGCTACTCCAGCTC

CCCCATCTCCTATTAAACCTAACTCATCTCCATCATAAACTACCCAACCA

CCCATACTATTAAAATTCAAGACTACCTCTA---------CTCCCTCATA

GTAATTAGTTACAAATGTTATCCCTAACTCTATAAAGATACTTATTACTA

AAACTCCAAATATTAACCAACTAGATACTCACGTTTCAGGATATTCTTCA

GCTGACATAGCAGTTGTATACCCAAATACAACCATCATTCCACCTAAATA

AATTAAAAATACTATTAACCCTAAAAACGACCCCCCAAGACCTATCACCG

CTAAGCAACCAGCACACCCACTAATAATCAAACATATCCCCCCATAAATA

GGTGAAGGCTTCAAAGATGTGCCTAAACACCCCAATGCAATAAATGAACT

TAAAATGAGAATTGATTCTATCATAGT---TTCTA-CACAGATACTA-TC

TATGACTAATGACATGAAAAATCATCGTTGT-AATTCAACTATAGAAAC-

ATT----AATGACAAACATTCGAAAAAAACATCCTCTAATAAAAATTATT

AACCACTCATTCATTGATCTTCCAACTCCATCTAATATCTCATCATGATG

AAACTTTGGATCCTTAATTGGACTTTGCCTAATTATTCAAATCCTTACAG

GCCTATTCCTAGCCATACACTATACATCAGACACTACTACAGCCTTCTCA

TCAGTAGCACATATTTGTCGAGACGTAAACTATGGTTGACTAATTCGCTA

TCTTCATGCTAATGGAGCTTCAATATTCTTTATTTGCTTATTTCTTCATG

TCGGTCGGGGTATCTACTATGGCTCATATACTATAGTAGAAACTTGAAAC

GTTGGAATTGTTTTACTATTCGCAGTTATAGCTACAGCATTCGTAGGCTA

CGTACTACCATGAGGCCAAATATCATTTTGAGGAGCTACAGTCATCACTA

ATCTTCTATCAGCTATCCCTTACATCGGCACAACTCTAGTAGAATGAATT

TGAGGCGGGTTTTCAGTCGATAAAGCCACACTAACTCGATTCTTTGCATT

TCATTTCATTCTCCCATTTATTGTAACAGCACTTGTTCTAGTTCACCTTC

TATTCTTACATGAAACAGGATCTAATAACCCATCAGGCCTAAACTCAGAT

GCAGATAAAATCCCATTTCACCCTTATTATACAATTAAAGACCTCTTAGG

TGTATTCCTACTATTGATTGCCCTCATAACTTTAGTATTGTTTTTCCCAG

ATGTTCTCGGAGACCCAGATAATTATACTCCTGCAAATCCACTTAATACT

CCAGCACATATTAAACCAGAATGATATTTTCTATTCGCATACGCTATCTT

ACGATCTATCCCCAATAAACTAGGCGGAGTTCTAGCTCTAATTCTCTCCA

TCCTAGTCCTAGCTGCTCTACCCCTTCTTCATACATCAAAACATCGAGCT

ATAATCTTCCGACCTATCACACAAACTATATACTGAATTCTAGTCGCAGA

CTTACTTTTACTAACTTGAATCGGAGGACAACCTGTAGAATATCCATTTA

TCATTATTGGCCAACTAGCCTCAATCGCTTATTTCGCTATTATTATTATC

TTCATACCAATTGCAAGCATAATCGAAGATAGCATTCTAAAATTCATTT-

--GTCTTGATAGTATAATA---ATTACTCTGGTCTTGTAAGCCAGAAATG

AAG---ATTAAATCTTCTCAAGGCA-----TCAAGAAGGAAGGACTATCC

CCCACCTTCAGCACCCAAAGCTGAAATTCTAG--TTGAACTACTTCTTG-

--------------------------------------------------

--------------------------------------------------

--------------------------------------------------

--------------------------------------------------

--------------------------------------------------

--------------------------------------------------

--------------------------------------------------

--------------------------------------------------

--------------------------------------------------

--------------------------------------------------

--------------------------------------------------

--------------------------------------------------

--------------------------------------------------

--------------------------------------------------

--------------------------------------------------

--------------------------------------------------

--------------------------------------------------

--------------------------------------------------

--------------------------------------------------

--------------------------------------------------

--------------------------------------------------

--------------------------------------------------

--------------------------------------------------

------

>Muscardinus_avellanarius_BEL Muscardinus avellanarius mitochondrion, complete genome.

GTTAATGTAGCTTAAC----------TAAAGCAAAGCACTGAAAATGCTT

AGATGGGTACT--ATTACCCCATAAACATAAA--GGTTTGGTCCCAGCCT

TCTTATTAATTTATAGCAGGATTACACATGCAAGCATCTACGACCCTGTG

AGAATGCCCT-CTATGTTAATACATGTTTAACCTAAAGGAGCAGGTATCA

AGCACACT--ATAATAGTAGCTCAAAACACCTTGC-TTAACCACACCCCC

ACGGGATACAGCAGTGATTAAAATTAAGT-TATAAACGAAAGTTTGACTA

AGCCATGTTATA---TTAGGGTTGGTAAATTTCGTGCCAGCCACCGCGGT

CATACGATTAACCCAAATTAATAAGTC-AC-GGCGTAAAGAGTGTTTTAG

AT------TTTAACTATAATAAAGTTAAACTTTAACTAAGCCGTAAAAAG

CCCTAGTTAAAAGT-AAAATAACGAACGAAAGTCACTTTAAT--ATTCTC

TGAGTACACGATAGCTAAGACACAAACTGAGATTAGATACCCCACTATGC

TTAGCCCTAAACATAAACCCTTA---TTAACCTAACTGTTCGCCAGAGAA

CTACAAGCCAAAGCTAAAAACTCAAAGGACTTGGCGGTGCTTTATATCCC

TCTAGAGGAGCCTGTTCTATAATCGATAAACCCCGATATACCTCACCATT

TCTTGT-CACCACAGCCTATATACCGCCATCTTCAGCAAACCTTAATAAG

GAGAT-AAAGTAAGCTCAAGCACT---GACATAAAAACGTTA-GGTCAAG

GTGTAGCTTATGAAATGGG-AAGAAATGGGCTACATTTACTTCACCAAGT

ATAT------------CCACGTTAACTCTTATGAAATCTGA--GAGTAGA

AGGAGGATTTAGTAGTAAGCT-AGGAATAGAGAGCCTAACTGAATAG-GG

CCATTAAGCACGCACACACCGCCCGTCACCCTCCTCAAGCATT-------

---TACTATTAATATTAGTTTATATTACCTTACAT--ACATGCAAGAGGA

GATAAGTCGTAACATGGTAAGCATACTGGAAAGTGTGCTTGGATTAATCA

AAACGTAGCTTA----AATTAAAGCATTTGGCCTACACCCAAAAGATTTT

A-TACATAATAAACGTTTTGA-AC-AAAACCT-AGCCCACTTTAT-----

-TTTTATACAAATAACAAAACTTAGTAAAATAAATCATTCAC--------

AACTAAAGTATAGGAGATAGAAATTT---TATTTTTGGCGCTATAGAGAC

AGTACCGTAAGGGAAAGA-TGAAAGAC--AACTTTAAGTGAGTAAAAGCA

AAGATTAAATCTTGTACCTTTTGCATAA-TGAATTAACTAGAAAATTTCT

AGCACAAAGCAATTAAAGTTAGATACCCCGAAACCAGACGAGCTACATAT

GAGCAGCT---ATTTGAGCCAACCCGTCTGTGTAGCAAAACAGTGGGAAG

ACTTGTATGTAGAGGTGAAAAGCCTATCGAGTCTGGAGATAGCTGGTTAC

CCAAG-CAAGAATTTCAGTTCAACTTTAATCTTACCTAAAGAACAATA--

AATCCTATTTGTAAGATTAAATCATATTCTAAAGAGGGACAGCTCTTTAG

A--TAAGGAAATAACCTGACATAGAGAGTAA-----ACTACCATTCTATA

CCACAGTTGGCTTAAGAGCAGCCATCAGTTAAGAAAGCGTTCAAGCTCGA

CACAACAATTTTCCTTAATTTA---------TATAGACTAGTTCAACT--

CCTATAAATTCAATTGGGTTAATCTATTCCATAAT-AGAAGCAATAATGT

TAATATGAGTAACAAGAAAAAAAAATTCTCCTAGCATAAACTTATACCAA

CTTTA---ATACTATTGATAGTTAACATCATAATAT-----TATTAATTA

AACTAATAAC---ATATTTATTATTATGTTAATCCAACACAGGAATGCAC

CACTCAAGGAAAGATTAAAAAAAGTAAAAGGAACTCGGCAAACACTAACC

CCGCCTGTTTACCAAAAACATCACCTCTAGCATCCCAAGTATTAGAGGCA

CTGCCTGCCCAGTGACA-TATG----TTCAACGGCCGCGGTATCCTGACC

GTGCAAAGGTAGCATAATCATTTGTTCCTTAATTAGGGACTTGTATGAAT

GGCTTAACGAGGGTTTAACTGTCTCTTACTTTTAATCAGTGAAATTGACC

TCCCAGTGAAGAGGCTGGGATAACCTAATAAGACGAGAAGACCCTATGGA

GCTTAAATTAAAT-AGCTTAATTATCACATTATATATCT--------AAA

GATATCCAATAAATAGTAACAATAAGCTATCAATTTCGGTTGGGGTGACC

TCGGAGAATAAACTAGCCTCCGAACGATAAAAA---TTAGACAA-ACTAG

TCAAATCTATTTATATTTATCATATTGACCCAA-AA--TATTTGATCAAC

GAAACAAGTTACCCTAGGGATAACAGCGCAATCCTATTTTAGAGTTCATA

TCGACAAT-AGGGTTTACGACCTCGATGTTGGATCAGGACATCCTAATGG

TGCAACCGCTATTAAAG-GTTCGTTTGTTCAACGATTAACAGTCCTACGT

GATCTGAGTTCAGACCGGAGTAATCCAGGTCGGTTTCTATCTATTTTCA-

-GCTTCTCCCAGTACGAAAGGACAAGAGAAGCAAGGCCAATTATTAGTAT

TACGCCTTAATTTTTATAGATGAA-CTAATCTTAATCTAGAACAATTTAC

TCAAT------TACCCTAAAAAAGGG---TTCGTTAAGGTGGCAGAGCCC

GGTAAATTGCATAAAACTTAAAACTTTATATTCAGAGATTCAAATTCTCT

CCTTAACAAC----------------------------------------

------------------------------------------ATGTTCTT

GATCAACCTTTTTATCTTAATTATCCCAATCCTTTTAGCCATAGCATTTT

TAACTCTAATCGAACGAAAAATTCTAGGCTACATACAACTCCGTAAAGGC

CCAAACATCATTGGACCTTATGGGCTCCTCCAGCCCATTGCAGACGCCAT

AAAACTTTTTATTAAAGAACCCCTCCGACCTCTATCATCATCAATAGCTC

TATTCACTATTGCACCAACACTAGCTCTTACACTAGCATTCACCATATGA

ATTCCACTACCCATACCATATCCCCTAATCAACATAAACCTAGGAATTAT

CTTTATACTAGCCACATCTAGCCTAGCTGTATATTCAATCTTGTGATCAG

GGTGAGCATCAAACTCCAAATACTCACTAATTGGCGCTCTACGAGCCGTA

GCACAAACTATCTCATATGAAGTTACACTAGCCATCATTCTACTCTCAGT

ATTAATATACAACGGATCATTTACCCTAATAACACTAACACAAACTCAAG

AACATGTATGACTAATTTTTCCCACATGACCACTAGCAATAATATGATTT

ATTTCCACACTAGCTGAAACAAACCGCGCACCCTTCGACTTAACTGAAGG

AGAATCAGAACTTGTCTCAGGATTTAATGTTGAATATGCAGCAGGCCCAT

TCGCCCTATTCTTCATAGCAGAGTATACTAACATCATTATAATAAACGCC

TTAACTGCTACACTATTCCTGGGAGCCCTAAATAACATTTTTCACCCAGA

ACTATTTACACTAAGTTTTATAACAAAAGCCCTTGTACTTACATCTACTT

TCCTATGAATCCGAGCATCCTACCCACGGTTCCGATATGATCACCTCATA

CATCTTCTATGAAAAAATTTTCTACCACTGACATTAGCTCTTTGTATATG

ACATATCTCATTACCAGTTACTATCTCGAACATTCCACCCCAAACCTAA-

GAAATATGTCTGA-AAAAGAGTTACTTTGATAGAGTAAAGCATAGAGGTT

TA-AACCCTCTTATTTCTAGAATAAAAGGAATTGAACCTTAACCTAAGAA

TTCAAAATTCTTCGTGCTACCTGAC-TACACTATATCCTAATT--A---A

GTAAGGTCAGCTAATT-AAGCTATCGGGCCCATACCCCGAATATGTTGGT

TTAAACCCTTCCCGTACTAATTAATCCTGTCATTGCTACAGCCATTTATT

TAACTTTATTCTCAGGAACAGTAATTGTTATATTCAGCCTTCATTGATTA

CTGACTTGAGTCGGATTAGAGATAAGTATACTAGCTATTATTCCAATCAT

CATCAATAAAGCCAACCCACGATCAACAGAAGCTGCAACCAAATATTTTC

TAATTCAAGCTACAGCATCCATAATTCTAATAATAGCAATTATCTCAAAT

ATAATTCTTACAGGACAATGAACAATATATAATTTCACTAACCCCCTACC

ATCAACATTAGCCACAATTGCTCTCACCATAAAATTAGGTATAAGTCCAT

TCCACCTATGAGTACCTGAAGTAATCCAAGGAACTACTATCATAGCAGGA

CTAGTAACTCTAACATGACAAAAACTCGCCCCAATCTTAATCTTGTATCA

AATTTCTCCCTTTATTAACAAATCAATAATTATAGCTATAGCAATTTTAT

CAATTGCCCTAGGAGGTTGAGGAGGCCTCAACCAGACCCAACTACGAAAA

ATCATAGCCTATTCATCCATCGCACACATAGGATGAATAATAGCCATCAT

TACCCTAAATCCATCAATCACTTCTCTTAACTTAATTATCTATATTATGT

TAACAATTACTATATTTATAATAATACTTTACAATAATAGCACTACTACA

CTCACTCTATCAACCCTATGAAATTTTACTCCACTAATAACCCTAATTAT

CCTAACAACTCTCATGTCATTAGGAGGTTTACCACCACTAACAGGATTCT

TACCTAAATGAATTATTATCCAAGAACTAATTAAAAACAGCATTAATATG

CTATCTTTAACCATGGCTATAATAGCCTTACTAAATCTATATTTTTATAC

CCGACTAATTTATTCTACTTCCCTAACTCTATTCCCGTCATCCAACAACA

TAAAAATAAAATGAAAATATGAACCCATAAAACTTATTATCCTAATTCCA

ACATTTACTTTAATATCAACACTACTTATACCATTAACACCAATAATCTC

AATCTT---AAGCT----AGGAATTTAGGTTAAG----CAGACCAATAGC

CTTCAAAGCTTTAAGCAAGCCGTCTAA----GCTTAATTCCTGTC----A

--------TTAAGGATTGCA-GATTTATTTCTACAT----CATTTGAACG

CAAATCAAAAACTTTAA--TT-AAGCTAAATCCTTTA------TTACCTA

GGCTGATG--GGAATTCAA-CCCAC-GAAACTTTAGTTAACAGCTAAAAA

CCCTAATCAACTGGCTTCAACCTA-CT-TCTCCCGCCGGGGAGAAAAAAA

A----GGCGGGAGAAGCCCCGGCAG---CTTCGAAGCTGCTTCTTT--GA

-----------------------------ATTTGCAATTCAATG--TGAT

TAA-TCACCACAAGACT-T-----GGCAGAAAGAGGT----TACC--CCT

CTGTCTTTAGATTTACAGTCTAATACTTA--CTCAGCCATTCTACCA---

---TACTTATGTTCATTAACCGTTGACTATTTTCAACAAATCATAAAGAT

ATTGGCACACTATACCTCATTTTTGGTGCTTGAGCCGGGATAGTAGGAAC

TGCTTTAAGTCTATTAATTCGGGCTGAATTAGGCCAACCCGGTGCACTCC

TAGGTGACGACCAGATTTATAACGTTATCGTTACCGCCCATGCCTTCATT

ATAATCTTCTTTATAGTTATACCCATAATAATCGGTGGCTTCGGAAACTG

ACTCGTTCCTTTAATAATTGGAGCCCCTGATATAGCATTCCCTCGAATAA

ATAACATAAGCTTTTGATTATTACCACCGTCATTTCTACTACTTCTAGCC

TCTTCTATGGTAGAAGCCGGTGCCGGCACTGGCTGAACAGTCTACCCACC

GTTAGCAGGCAATTTAGCACATGCAGGAGCCTCAGTTGATCTTACTATCT

TTTCACTTCACTTAGCGGGAGTCTCATCAATTTTAGGAGCTATTAACTTT

ATTACAACTATTATTAACATAAAACCCCCTGCTATATCACAATATCAAAC

CCCTTTATTTGTTTGATCAGTCCTAATTACCGCTGTCTTACTGCTTCTTT

CACTTCCAGTATTAGCAGCCGGCATTACTATACTACTCACTGACCGTAAT

CTAAATACTACTTTCTTCGATCCTGCCGGAGGAGGTGACCCAATCTTATA

TCAACACTTATTCTGATTCTTCGGACACCCTGAAGTTTATATTCTCATTT

TACCAGGGTTTGGTATTATCTCTCATATTGTTACATATTATTCAGGGAAA

AAAGAACCTTTCGGCTACATGGGTATAGTCTGAGCTATGATATCAATCGG

CTTCCTAGGCTTCATCGTATGAGCCCACCACATATTTACAGTTGGTCTAG

ACGTAGATACCCGAGCATACTTTACATCAGCAACAATAATTATTGCTATC

CCTACGGGAGTAAAAGTATTTAGTTGATTAGCAACCCTTCACGGGGGAAA

TATTATTTGATCTCCCGCTATATTATGAGCTCTAGGTTTCATTTTCTTAT

TTACTGTTGGAGGACTAACCGGTATTGTTCTAGCCAATTCATCCCTAGAC

ATCGTCCTCCATGATACTTATTATGTCGTAGCACATTTCCACTACGTATT

ATCAATAGGAGCCGTATTCGCAATTATAGGTGGCTTTGTCCACTGATTCC

CATTATTTACAGGCTATACACTCGACTCTTCCTGAGCCAAAATTCACTTT

ACAGTCATATTCGTAGGTGTAAACATAACATTCTTCCCACAGCACTTTTT

AGGTCTTTCGGGGATACCACGACGATATTCTGATTATCCCGATGCCTATA

CTACATGAAATGCAGTATCTTCAATAGGCTCATTCATCTCTCTTACAGCT

GTAATAATTATAATCTTCATAATCTGAGAAGCATTCGCCTCTAAACGAGA

AGTAATAACAGTTGAATTACCAACAACAAACTTAGAATGACTTCACGGAT

GCCCACCCCCCTATCATACATTCGAAGAACCTACATTCGTAAAAAC----

ACAGT-------------CAAGAAAGGAAGGAATTGAACCCCCTAAAGCT

AGTTTCAAGCCAGCCCTATAACCACTATAATCTTTCTTTATAAT---CAA

GATATTAGTAAAAT-AATTACATAACTTTGTCAAAGTTAAATTACAGATC

------TAATATCTGTATATCTTATATGGCATATCCTTTCGAATTAGGCT

TCCAAGACGCCACATCACCAATTATAGAAGAACTACTAAATTTCCACGAC

CATGCCTTAATAATCGTTTTCTTAATTAGTTCACTAGTCCTTTATATCAT

CTCTCTAATACTGACAACAAAACTAACACATACTAGTACAATAGATGCCC

AAGAAGTTGAGACTATTTGAACTATTCTCCCAGCTATTATTCTTATTATA

ATTGCCCTACCATCCCTTCGCATTCTATATATAATAGATGAAATCAATAA

CCCAGTTCTAACAGTTAAAACAATAGGCCATCAATGGTATTGAAGCTATG

AATATACAGACTATGAAGACTTAAATTTTGACTCCTATATGATCCCTACA

ACAGATTTAAAACCGGGAGAACTACGTCTTCTCGAAGTCGATAACCGAGT

TGTATTACCTATAGAATTACCTATCCGCATATTAATTTCATCTGAAGATG

TTCTCCATTCATGAGCAATCCCATCTTTAGGCTTAAAAACGGACGCTATT

CCAGGACGCTTAAATCAAGCAACCCTTACATCTACACGACCAGGGCTTTA

CTATGGCCAATGCTCAGAAATTTGCGGATCCAATCACAGCTTTATACCTA

TTGTGCTTGAAATAGTACCACTAAAATATTTTGAAAACTGATCCCTGTCC

ATAATTTAA--------------------------------ATTACATTA

TGAAGCTA--AAATAGCATTAACCTTTTAAGTTAAAGAATGAGAATACAA

---T-TTCTCCATAATGAGATGCCCCAACTAGACACATCAACATGATTTA

TCATCATCCTGGCGTCTA-CCATGACATTATTTATAATGATTCAACTAAA

ACTCCATTCACA--TGTTTATTCGCCTAACCCCACACCCAAAGAT-TTAA

AAACACTTAAACATAACTGCCCTTGAGATAAAAAATGAACGAAAATCTAT

TTGCCTCATTCGTAATACCGTCCTTTATTGGCCTTCCCATTGTTATTATC

ATTGTTATATTTCCCATTATACTATTCCCTTCACCAAGCCGTTTAATTAA

CAATCGTCTAATTACATTTCAATTATGACTAACACGATTAGTTCT-AAAA

CAAATAATAGCCATACATAGTAACAAAGGACGTACATGGTCTCTTATATT

AGTCTCACTTATTATATTTATTGGCTCTACTAATTTATTAGGCTTATTAC

CCCATACATTCACCCCAACAACTCAATTATCAATGAACCTAGGTATAGCA

ATCCCCCTTTGAGCAGGGGCTGTCATTATAGGATTCCGCCATAAAA-CTA

AATCATCATTAGCACATTTTCTTCCCCAAGGAACACCTATTCCATTAATC

CCAATACTTATTATTATCGAAACCATTAGCCTGTTTATTCAACCCATAGC

ATTAGCCGTTCGACTAACAGCCAACATCACAGCAGGCCATCTCCTTATTC

ACCTTATTGGAGGGGCAGCTTTAGCACTTATCTCTATCAACATTCCTACC

GCTATAGTTACATTTATTATTCTTATTATATTAACAATTCTAGAATTCGC

CGTTGCCCTAATTCAAGCTTATGTATTTACACTTTTAGTAAGCCTGTATC

TTCATGATAATACTTAATGACCCACCAAACTCATGCCTATCATATAGTTA

ACCCCAGTCCCTGGCCAATTACAGGAGCTTTCTCAGCCCTATTACTAACA

TCTGGCCTAGTAATATGATTCCACTTTAACTCTACTACTCTCCTCACTTT

AGGCTTAGTTACTAACACTTTAACTATATACCAATGATGACGTGATATCA

TTCGTGAAGGAACTTTCCAAGGACATCATACTTCAATTGTACAAAAAGGT

CTACGATACGGTATAATTCTATTCATTGTGTCTGAAATTTTCTTTTTCGC

AGGCTTCTTCTGGGCTTTCTATCACTCAAGTTTAGCCCCAACACCTGAAT

TAGGAGGATGCTGACCCCCAACAGGAATCTTCCCACTTAATCCTTTAGAA

GTCCCCCTTCTTAACACAACGGTCCTTCTGGCCTCCGGAGTATCCATTAC

CTGAGCCCATCACAGTTTAATAGAAGGTAACCGAAATCACATAACTCAAG

CCCTATCTATCACCATTTTATTAGGCTTATATTTTACAATTCTTCAAGCA

TCAGAATATTTAGAAACATCATTTACTATCTCAGATGGGGTTTATGGATC

AACATTTTTTATAGCCACAGGATTCCATGGACTTCACGTAATTATCGGAT

CAACCTTCCTAACTGTCTGCTTACTACGCCAATTAAATTTTCACTTTACA

TCAAAACACCATTTCGGATTTGAAGCTGCTGCCTGATATTGACATTTCGT

TGACGTAGTGTGATTATTCCTGTATGTATCAATCTATTGATGAGGTTCAT

ACTCT-TTTAGTATTAA-CTAGTACAACTGACTTCCAATCAGTAAGTTTC

AGAAATTAACTGAAAAAGAGTAATCAACATAATATTAGCTATATTTATTA

ATACTACTCTAGCAACCATTCTAATCTCTATCGCTTTCTGATTACCCCAG

ATAAATATTTATACAGAAAAATCTAGCCCATACGAATGTGGGTTTGACCC

ACTAGGTTCTGCACGTCTCCCATTCTCAATAAAATTCTTCCTAGTAGCCA

TTACTTTTCTACTATTCGACCTAGAAATCGCTCTTCTACTCCCACTACCT

TGAGCCTCACAAATTAATAACCTCCCCCATATATTGATAATCGCACTCAT

ATTAATCTCTATCTTAGCCTTAGGTCTGGCATACGAATGGCTACAAAAAG

GCTTAGAATGAGTAGAATAAA-CGATGGTAATTAGTTTAATATAAAA-CA

AATGATTTCGACTCATTAAATTATAAGAGTATT-TATAATTACCAA--AA

TGCCTTTATTTTTTCTTAATATATTCTCTGCTTACTTATTATCATTTTTA

GGGGTTTTATTATATCGATCACACCTTATATCATCACTTTTATGCTTAGA

AGGTATGATACTCTCTATATTTATTATTATCTCAGTAGTTATCATTAATC

TCCACTTTACATTAACATTTATTATACCAATCATTTTACTTGTATTTGCT

GCCTGTGAAGCTGCTATTGGCCTAGCCCTACTAGTCATAGTATCAAACTC

ATACGGCCTAGACTATGTAAAAAACCTAAACATATTACAATGTTAAAAAT

TATTGTCCCCACAATCTTACTTATCCCCATCACTTGATGCTCAAAAATCT

CTTCTCTATGAACAAACGTAACACTTCACAGTTTCTTAATTAGTATTCTT

AGCCTTTTCCTACTTAATCAAATCGATATTACCAACTTAAATTTCTCACT

CATATTTTCCTCAGATTCACTATCAAGCCCTTTATTAATTTTAACAGCAT

GATTATTCCCTCTAATAGTCATTGCAAGCCAAAATCATTTGACTAAAGAA

ACTGAAATACGAAAAAAACTATTTATTACTACCTTAGTTTCTCTTCAAGT

CTTTCTAATTATAACATTTTCTGCTACTGAACTAATTTTTTTCTATATTT

TATTTGAAGCTACATTAATTCCTACTCTTATTATTATTACACGATGAGGA

AACCAAACTGAACGACTCAATGCAGGATTATACTTTCTATTCTATACATT

AATTGGATCACTCCCACTTTTAATTGTGCTTATTTTTATTCAAACCAAAT

CGGGTTCTCTAAATTTTCTAATC---ATATCCTACTCAAATTACCTAATT

GAAGTCAAC-------TGATCAACTAATTTACTCTGATTAGCATGCATAA

TAGCTTTTATAGTAAAAATACCATTATATGGATTACACTTATGACTACCA

AAAGCCCATGTAGAAGCACCAATTGCAGGATCAATAGTACTTGCCGCTAT

TCTACTGAAACTAGGTGGCTATGGAATAATACGAGTAACCATTTTACTAG

ATCCTATTACAAATACTATGTCTTACCCATTTATGATACTATCACTTTGG

GGAATAATTATAACTAGCTCGATCTGCCTACGGCAGACTGATCTAAAATC

TTTAATTGCATACTCCTCAGTTAGCCATATAGCATTAGTAATCATCGCTA

TCTTGGTTCAAACCCCATGAAGCTTTATAGGAGCTACTATTCTAATAATC

GCACACGGCTTAACCTCCTCCCTCTTATTCTGCTTGGCTAATACTAATTA

TGAACGTATCCACAGTCGAACCCTCATATTAGCCCGAGGCTTACAAATGA

TTCTTCCACTAATAGCTACTTGATGAATTATAGCAAATTTAACTAACCTA

GCATTGCCACCCTCAATTAATCTCATTGGAGAACTACTCATTATCTTAGC

CTCATTCTCATGATCCAATTTTACAATGATCTTTATAGGCCTTAATATAA

TTATTACAGCCCTATATTCTATGTATATATTAATCATTACTCAACGAGGT

AAACTATCACAACATATCAAAATGATAATCCCATCATTCACACGAGAAAA

TATTTTAATGGCTTTCCATTTACTTCCCATTATCTTACTCACTGTTAACC

CTAAAATTATTTTAGGAAACTTATACTGTAAATATAGTTTAACAAAAACA

TTAGATTGTGAATCTATTAATAGAAGACT-TAAATCTTCTTATTTACCAA

GAAAGTGAA--GCAAGAACTGCTAACTCATTGCCACCATATATAAAAATA

TGGCTT--------------------------------------------

-------------------TCTTA-ACTTTTAAAGGATAGTAGTA-ATCC

ATTGGTCTTAGGAACCAAAAA-A-TTGGTGCAACTCCAAATAAAAGTAAT

AAAAA---TATTTGCTTCCTTCACAATGCTTACCATTATAATCTTAATTT

ACCCTATTGTCATAACCCTATCAAGTAACTACAACATTTTCAACTTTCCT

AAGCACGTAAAAACTACTATTAAATCTGCTTTCTTATTTAGTCTTATCCC

CACCTTTATATTCATTGATAAAGGCTATGAAGCAATTATCACAACTTGAC

ACTGAACTATCTTTCAAACCCTGGACATTTCCATTAGCCTAAAAATAGAC

TTCTTCTCAATAATATTTGTTCCCGTAGCCTTATTTGTTACATGATCCAT

TATAGAATTTTCAATATGATATATGCATAGTGACCCTAATGTAGACCGAT

TCTTCAAATACCTTCTTCTTTTCCTCATTACAATAATTATCCTAGTAACA

GCAAATAACCTATTCCAACTTTTTATCGGTTGGGAAGGAGTAGGAATCAT

GTCCTTCCTACTAATTGGCTGATGATATGGACGACCAGATGCAAATACAG

CAGCACTCCAAGCTATTCTATATAATCGAATTGGCGACATCGGGTTCGTC

TTATCCATAGCATGATTTTTAACTAACTCCAACTCATGAGAACTCCAACA

ACTATTTATTACTGACCATAATAA---CCTTATTCCTTTACTAGGCTTAA

CCCTAGCAGCAACCGGTAAATCAGCCCAATTTGGTCTCCACCCATGACTT

CCATCAGCTATAGAAGGCCCAACCCCTGTATCAACCCTACTTCACTCTAG

CACTATAGTAGTTGCAGGTATTTTTCTCCTAATCCGATTTTACCCTATAT

TA-GAAAATAATCAAACTATCCTAACAACTATTATATGTCTAGGAGCTAT

TACAACCCTATTTACAGCAATCTGTGCTCTCACACAAAATGATATTAAAA

AAATTATTGCTTTCTCAACTTCAAGCCAACTAGGTCTTATAATAGTAACT

ATTGGCATTAACCAACCCCATCTAGCCTTTCTTCACATCTGCACTCACGC

TTTCTTCAAAGCTATATTATTTATATGCTCAGGGTCTATTATTCACAACC

TTAATGATGAGCAAGACATCCGAAAAATAGGAGGCCTATTTAAAACTCTC

CCTTTCACTTCATCAGCATTAACTATTGGTAGCCTAGCACTAACAGGCAC

CCCATTCCTAACCGGATTCTATTCCAAAGACCTAATTATCGAATCCGCTA

ATACGTCGTATACCAACGCCTGAGCCCTACTAATTACACTAATTGCCACA

TCCCTTACTGCTACTTACAGCACTCGTATTATCTTCTTCGCCCTTATAGG

CCAACCACGGTTTTCACCACTTAGTCCTATTAATGAAAATAACCCTTTAC

TTAACAATGCTATTAAACGCTTACTAATTGGTAGCATTTTTGCCGGGTTC

ATTATTACCAACAATATAGACCCAATGAACATCCAACAAATAACCATACC

CTGATACCTAAAACTAGCAGCCTTAATAGTTACTGTCTCCGGCTTCATAA

TCGCAATAGAACTTAATAACCTAACATATTTCTTAAAAATCAAATCAACC

TCACA-ACAAATAAAATTTTCTAATCTCTTGGGATACTTCCCATCAACCA

TTCACCGACTAATCCCCAACACAAACCTCATAACAAGCCACAAATCAGCA

TCAAAACTAATAGACCTAATTTGACTAGAAAAATCTACCCCCAAACTCTT

AGCCTTACTCCAA---TTGATAATATCAACATTAACTTCAAACCAATCAG

GACTTATCAAAGTTTACTTTATAACTTTTTTACTATCCATATTAATCTCT

TTACCTTTTCTTATTTA--------------------------ATTTCCA

CGTGTAATCTCAATTACAATAAAAATACTTACAAATAAAGACCACCCAGC

CACTACTATTAATCAACTTCCACAGCTATACAAAGCAGCAACACCCGTTG

AATCCTCACGAATTAAACCAACATCATCAACTTCAAACAACACTCAATCC

TCTAACCCATTAAAATTAATAACAATCTCCA---------CTTCGTCATA

AGTCTTCATTAACAAAATTAAAACAATCTCCACCACAAACCCTGATAAAA

GTATACTTCAAACTATACTATTAGAGCCCCATGTCTCAGGATATTCTTCA

GTAGCCATAGCAGTTGTATAACCAAACACAACTAATATCCCACCTAAATA

AATTAAAAATACCATTAAACCTAAAAATGAACCCCCAAAATATAATACTA

TACCACAACCAAACCCCCCACTTAAAATTAACCCTAAACCCCCATAAATA

GGCGAAGGCTTTAAAGAAATCCCTAAAAAGCCAAATACAAATGCTAAACT

TAACAGAAATAAAATGTATGTCATAAT---TTTTA-CATGGG-TTTAAAC

CATGACTAATGACATGAAAAATCATCGTTGTATCTTCAACTATAAAAACA

CT-----AATGACAAACGTCCGCAAATCTCATCCACTCATTAAAATTATT

AACAACTCATTTATTGACCTACCTACCCCCTCCAATATTTCAGCATGGTG

GAATTTCGGTTCTCTATTAGGCGCTTGCCTAGGCATTCAAATTCTTACTG

GTCTTTTTCTTGCCATACACTATACATCAGATACATTAACAGCATTCTCC

TCAGTTACACACATCTGCCGAGATGTAAACTACGGCTGATTAATTCGTTA

CATGCACGCAAACGGGGCATCCATATTCTTTATTTGCCTCTTTCTCCACA

TTGGCCGAGGCGTATACTACGGCTCCTACATATTTATAGAAACATGAAAT

ATTGGAATTATTCTCCTATTCGCAGTTATAGCAACTGCATTCATGGGATA

TGTACTCCCATGAGGACAAATATCCTTCTGAGGAGCAACAGTAATCACCA

ATTTATTATCTGCTATTCCTTATATTGGTACTACCCTAGTAGAATGAATC

TGAGGAGGATTCTCAGTTGACAAAGCTACCCTTACACGGTTTTTCGCATT

CCACTTTATTCTACCTTTCATCGTTGCAGCCCTAGTAATAGTTCACCTTT

TATTCCTCCACGAGACAGGATCCAACAACCCATCAGGCCTAAACTCTGAC

ACAGACAAAATTCCATTCCACCCTTACTTTACAATTAAAGACATCCTAGG

TCTTCTTCTCCTCATTTCTATTCTAATATCATTAGTATTATTTTCCCCAG

ACCTTCTGGGCGATCCTGATAATTATACACCAGCCAATCCCCTAAGTACT

CCACCACATATTAAACCAGAGTGATATTTCCTATTTGCATACGCCATCCT

TCGATCCATCCCAAACAAACTAGGCGGAGTACTAGCTCTAGTATTCTCGA

TCCTAATCCTAGCAATCTTCCCCATAATCCAAATTTCTAAACAACGAAGC

ATAATATTCCGCCCTCTAAGCCAACTCCTATTTTGAATTCTAACAGCAGA

CCTATTTACCTTAACATGAATTGGAGGCCAACCAGTTGAACACCCATTTA

TTATCATCGGACAATTAGCATCAGTCTTATACTTTTCTATTATTCTAGTA

TTTCTCCCCACAACCAGTCTAATCGAAAATAAACTCCTAAAATGATA---

--GCCCCGATAGTATAA--CCTATTACACTGGTCTTGTAAGCCAGAAATG

AGATTCACTAACCTCTCTCAGGGTAA----TCAAGGAAAAGGCTCTA-GC

CCCACTTCCGACCCCCAAAGCCGGAGTTCTAA--TTAAACTATTCCTTG-

--------------------------------------------------

--------------------------------------------------

--------------------------------------------------

--------------------------------------------------

--------------------------------------------------

--------------------------------------------------

--------------------------------------------------

--------------------------------------------------

--------------------------------------------------

--------------------------------------------------

--------------------------------------------------

--------------------------------------------------

--------------------------------------------------

--------------------------------------------------

--------------------------------------------------

--------------------------------------------------

--------------------------------------------------

--------------------------------------------------

--------------------------------------------------

--------------------------------------------------

--------------------------------------------------

--------------------------------------------------

--------------------------------------------------

------

>Myotis_bechsteinii Myotis bechsteinii mitochondrion, complete genome.

GTTAATGTAGCTTAATAC--------TAAAGCAAGGCACTGAAAATGCCT

AGATGAGTCTCTCC--ACTCCATAAACACATA--GGTTTGGTCCTGACCT

TTCTATTAGTTACTAGTAAACTTACACATGCAAGAATCCCCATTCCAGTG

AGAACGCCCT-CTATGCCACTTAA----TGACAA-AAGGAGCTGGTATCA

AGCACACT---ATAAAGTAGCTCATGACACCTCGC-TTAGCCACACCCCC

ACGGGACACAGCAGTGATAAAAATTAAGCA-ATAAATGAAAGTCTGACTA

AGTTCTACTA----TTTAGGTCTGGTAAATCTCGTGCCAGCCACCGCGGT

CATACGATTAGATCAAACTAATAGACA-CTCGGCGTAAAGCGTGTTTTAG

AGAACAA----ATAACAAATAAAATCGAGCCTTAACTAAGCTGTAAAAAG

CCTCAGCTATTG--TAAGATAAACAACGAAAGTGATTTTA---AAAAATC

TGACTACACGACAGCTAAGACCCAAACTGGGATTAGATACCCCACTATGC

TTAGCCCTAAACATGAAAAATTATA---AACAAAATTATTCGCCAGAGTA

CTACTAGCAATAGCCTAAAACTCAAAGGACTTGGCGGTGCCTTAAATCCC

TCTAGAGGAGCCTGTTCTATAATCGATAAACCCCGATCCACCTCACCAAT

CTTTGCTAA-ATCAGCCTATATACCGCCATCTCCAGCAAACCCTAA-AAG

GGAATTAAAGTAAGCACAAGTATTA---ACATAAAAACGTTA-GGTCAAG

GTGTAGCTTATAGATTGGA-AAGAAATGGGCTACATTCCCCGATCCGGAG

AACTAGAAT----TCTATACGAAAACTTATGTGAAACCAAA---AGTCAA

AGGTGGATTTAGCAGTAAATT-AAGAGTAGAGAGCTTAATTGAATGTTGG

CCATAAGGCACGCACACACCGCCCGTCACCCTCCTCAAATAAAACAATAT

TAAATATACCCTAAATGATATACA------AATATTAATCTATGAGAGGA

GACAAGTCGTAACAAGGTAAGTGTACTGGAAAGTGCACTTGGATAACACA

AAGTGTAGCTTAA---ACA-AA-GCACCTAGTTTACACCTAGAAGATTTC

A-CATAAACGGACCACTTTGA-GACAAAAAAT-AGCTCATAA--TACACC

AAATGATACTATCCTAA-A-CATTA--AAATAAAACATTCACA---AAA-

-ACAAAAGTATAGGTGATAGAAATTT-----TACTTGACGCTATAGAGAA

AGTACCGCAAGGGAAA-AATGAAAGAA-GACTTACAAGTAAAAAAAAGCA

AAGTTTAACTCTTGTACCTTTTGCATAA-TGACTCAACTAGAAAATA-TT

AGCAAAAAG-AATTTAAGTTAAACACCCAGAAACTAGACGAGCTACCCAT

TAGCAGCCT-AA--AGAGCAAACTCATCTATGTGGCAAAATAGTGAGAAG

ACTGACGGGTAGTGGCAATAAACCTACCGAGCCTAGTGATAGCTGGTTGT

CCA-GAATGGAATTTTAGTTCTAACTTAAGCCTACCATAAAAAAAA-T--

AAATTTAAATGTATGCTTAAATATTAATCTAAAAGGGTACAGCTTTTTAG

ATATG-GGATACAACCCCTATTAGTGAGTAAACAGAAG-CTAGAATA---

CTCTAGTTGGCCTAAAAGCAGCCATCAAACAAGATAGCGTTCAAGCTCAA

T------AATACACAAAAACTAATTTCAATTATCAATAATAATA--CT--

CCTAATATAA-AACTGGACTATTCTATTTTTTAAT-AGAAGCAATAATGT

TGATATTAGTAACAAGAAATAT---TTCTCCCTGCATGCGTGTATATCAG

ACCGAA-TAGATCACTGATAATTAACAA----GTACAAACATATTCACTC

AACTAAAAG----CC-CAATGT-AATTGTTAGCCCAACACAGGTATGCA-

--AACAGGGAAAGATTAAAAAAAGTAAAAGGAACTCGGCAAACATAAACC

CCGCCTGTTTACCAAAAACATCACCTCTAGCATAAAAAGTATTAGAGGCA

CTGCCTGCCCAGTGACTTTA-G----TTCAACGGCCGCGGTATCCTGACC

GTGCAAAGGTAGCATAATCACTTGTTCTCTAAATAAGGACTTGTATGAAC

GGCTACACGAGGGTTTTACTGTCTCTTACTTTCAATCAGTGAAATTGACT

TTCACGTGAAGAGGCGTGAATTAAAAAATAAGACGAGAAGACCCTATGGA

GCTTTAATTAATCAACTCAAAAAC-ACAATTATATTCTAC---------T

AGAAATTTAAACCTATTTCTATTGAGTTGACAATTTAGGTTGGGGTGACC

TCGGAATAAAAACTAACTCCCGAGAAAATTTCAATT--AAGATAAACAAG

TCAAAATTATACTATTA---CATATTGACCCGTTAAATTAAACGATCAAC

GGAACAAGTTACCCTAGGGATAACAGCGCAATCCTATTTAAGAGTCCATA

TCGACAATTAGGGTTTACGACCTCGATGTTGGATCAGGACATCCCAATGG

TGCAGCCGCTATTAATGTGTTCGTTTGTTCAACGATTAA-AGTCCTACGT

GATCTGAGTTCAGACCGGAGTAATCCAGGTCGGTTTCTATCTATTT-AAG

-GTCCCTCCTAGTACGAAAGGACAAGAGAGACAGGGCCTACTTAAAATAA

GCGCCCTATTTACT--TAGATGAA-CTAATCTTAATCTCATAATATATAT

AT--------CTACCCAAGAACAGGG--TCCAGTTAAAGTGGCAGAGACC

GGTAA-TTGCATAAAACTTAAACTTTTAAAGCCAGAGGTTCAACTCCTCT

CTTTAACAACTTC-------------------------------------

------------------------------------------ATGTATTT

CATTAACCTATTAACAATAATTATTCCTATCTTACTAGCCGTAGCATTCT

TGACCTTACTAGAACGAAAAATATTAGGCTACATACAACTTCGAAAAGGA

CCCAATATTGTAGGTCCCTACGGCCTACTACAACCAATCGCTGACGCAGT

TAAATTATTTACTAAAGAACCCATGCAACCGTCAACGTCATCCCTTATCC

TATTCATTATTGCACCAACCCTAGCCCTAACTCTGGCCCTAATAATATGA

GCCCCATTGCCCATGCCACACCCACTAATCAACATAAACTTAAGCATACT

ATTTATACTAGCCCTATCAAGTTTAGCCGTATACACCATCTTATGATCAG

GCTGAGCTTCAAACTCAAAATACGCATTAATTGGAGCTCTACGGGCAGTA

GCCCAAACAATCTCCTACGAAGTAACCCTTGCCATTATTATTTTATCTGT

CCTACTTATAAATGGCTCTTTTACATTAACTATACTAATCGCAACACAAG

AATGTATTTGATTAATTATACCCTCATGACCCCTAGCCATAATATGATTT

ATCTCAACTTTAGCAGAGACCAACCGAGCACCCTTTGACCTAACAGAAGG

TGAATCAGAACTAGTATCTGGTTTCAATGTAGAATATGCAGGAGGACCCT

TCGCTCTCTTTTTCCTAGCAGAATATGCAAATATTATTATAATAAATGCC

CTCACAACTATCCTATTTTTAGGTGCATACAATAACCCAATATTCTCACA

ACTCTATACTATAAACTTCACCACCAAAACTATTTTATTTACAATAATCT

TTCTATGAATCCGAGCATCATACCCTCGATTCCGATATGACCAATTAATA

CATTTATTATGAAAAAATTTCCTACCCCTTACTTTAGTTATATGTATATG

ACATGTAACCCTACCAATTATCCTAGCAAGCATCCCACCCATAACATAA-

GAAATATGTCTGATAATAGAGTTACTTTGATAGAGTAAATTATAGGGGTT

TA-AATCCCCTTATTTCTAGAGTTGCAGGAATTGAACCCGCTCTTAAGAA

TCCAAAAATCTTCGTGCTACCTATATTACACCATACTCTAA---------

GTAAGGTCAGCTAAACTAAGCTATCGGGCCCATACCCCGAAAATGTTGGT

TCATATCCTTCCCGTACTAATTAACCCCATAGTCCTATCACTAGTATTGA

CAACAATAATCTCAGGCACTCTAATCGTTATAACAAGCTCACACTGATTC

CTAACTTGAATAGGATTTGAAATAAACATGTTAGCTATAATTCCACTACT

AACAAAAGAACATACTCCACGATCCACAGAAGCAGCAACTAAATATTTTC

TTACCCAAGCTACAGCATCCATACTCCTCATAATAGCTGCAATTATTAAT

CTATTACATACAGGCCACTGATCTATTATAAAATTAATTAACCCAACAGC

ATCAATTTTAATAACAATAGCTCTCACAATAAAATTAGGCCTATCCCCAT

TCCATTTTTGAGTACCAGAAGTAACTCAAGGAATCCCACTGATATCAGGA

TTAATCCTACTAACATGACAAAAACTAGCACCCCTATCAGTCCTCTACAT

AATTACACCTCTCATTAATATAAATCTTCTTCTAACCATATCACTAATAT

CTATCGCAATCGGCGGCTGAGGCGGACTAAACCAAACCCAACTACGCAAA

ATTATAGCATATTCATCTGTCACCCACATAGGATGAATATTAGCCATCTT

AGCCTATAATCCAACTATAACACTACTAAATCTTTACCTATACATTCCAA

TAACAATTACAATTTTTATACTACTAATATTAAGCTCAACAACTACAACA

ACCTCATTATCCTACATGTGAAATAAGCTACCACTAATCACCATATTAAT

CTTAACTACCATACTATCTTTAGGAGGACTACCTCCCCTGACCGGGTTCT

TACCAAAATGAGCAATTATTCAAGAAATAACAAAAAACAGTAGCATCCTG

ATACCTACACTAATAACTCTTCTGGCCCTATTAAACTTGTACTTTTACAT

ACGAATTACATACACCACATCACTAACAATATTCCCAACAACAAATAATA

TAAAAATAAAATGACAATTCAAAATTCCAAAACAAATAATATATCTACCC

CTAATAATCACAATCTCTACCATAACTCTCCCACTAGCACCAATTATAAT

GATTCT---GGAAT----AGGAGCTTAGGTTAC---TTTAGACCAGAAGC

CTTCAAAGCTTCCAGCAAATA-TGATTT---ATTTAGCTCCTGTG----C

AA-------TAAGGACTGCAAGACTTTATCCTACAT----CAAATGAATG

CAAATCAATCACTTTAA--TT-AAGCTAAGCCCTTC-----------CTA

GATTGATG--GGATTTTAA-CCCAT-AAAAAATTAGTTAACAGCTAAAAA

CCCTAAACAACTGGCTTCAATCTA-CT-TCTCCCGCCGCAAATAAAAAAA

-----GGCGGGAGAAGCCCCGGCAGGATT---GAAGCTGCTCCTTT--GA

-----------------------------ATTTGCAATTCAATA--TG--

TATTACACTACAGGACT-T-----GGCAAAAAGAGGAA----TTCCACCT

CTGTCTTTAGATTTACAGTCTAATGCCTA--CTCAGCCATTTTACC----

-------TATGTTCATTAATCGATGATTATTTTCAACAAACCACAAAGAC

ATCGGTACCTTATACTTATTATTTGGTGCTTGAGCCGGTATAGCAGGAAC

TGCTTTAAGCCTACTAATTCGAGCAGAACTAGGTCAGCCAGGGGCCCTGC

TAGGAGATGATCAGATTTATAATGTAATTGTCACTGCTCATGCCTTTGTA

ATAATTTTCTTTATAGTTATACCTATCATGATTGGGGGATTTGGAAATTG

ACTAGTACCCCTGATAATCGGAGCTCCCGATATAGCCTTCCCTCGAATAA

ATAATATGAGCTTCTGACTACTTCCTCCCTCCTTCCTATTACTACTAACT

TCATCTATAGTTGAAGCAGGAGCTGGTACTGGTTGAACAGTTTATCCTCC

TTTAGCAGGAAACCTTGCCCATGCAGGAGCTTCTGTCGACCTTGCAATTT

TTTCCCTACACCTAGCAGGTGTATCCTCAATTCTAGGAGCAATCAATTTT

ATTACCACTATTATTAACATGAAACCTCCTGCACTTTCTCAATATCAAAC

ACCATTATTTGTCTGATCTGTCTTAATTACAGCTGTATTACTCCTTCTAT

CCCTCCCAGTTCTAGCTGCCGGAATCACAATACTGTTAACAGACCGAAAT

CTAAATACCACCTTTTTTGATCCTGCCGGCGGAGGAGATCCAATTCTGTA

TCAACATCTATTTTGATTCTTTGGACACCCTGAAGTATATATTTTAATTC

TACCTGGATTTGGAATCATTTCCCATATCGTTACATATTACTCAGGAAAA

AAAGAACCCTTCGGGTATATAGGAATAGTATGAGCCATGATATCTATTGG

ATTCCTGGGCTTCATTGTATGAGCTCATCACATGTTTACAGTGGGAATAG

ATGTAGATACCCGAGCTTATTTTACATCAGCTACTATAATTATTGCTATT

CCTACCGGAGTTAAAGTCTTTAGCTGACTAGCCACCCTTCATGGGGGTAA

TATTAAATGATCTCCCGCTATACTGTGAGCACTGGGATTTATTTTCTTAT

TTACAGTCGGAGGCCTAACAGGAATTGTACTTGCTAATTCTTCTTTGGAT

ATTGTTCTCCATGATACCTATTATGTAGTAGCTCATTTCCACTATGTCCT

ATCTATAGGAGCAGTTTTTGCTATTATAGCCGGTTTTATTCATTGATTCC

CCCTGTTTTCAGGCTATACAATTAGTACAACCTGAGCAAAAATCCACTTT

CTAATTATATTTGTAGGAGTTAATATAACTTTCTTTCCACAACATTTCCT

AGGACTATCAGGCATGCCCCGACGCTACTCAGATTATCCAGATGCTTACA

CCACTTGAAATACTGTATCTTCTATGGGCTCATTCATCTCACTAACTGCT

GTTATTTTAATAATCTTTATAGTATGAGAAGCATTTGCATCTAAACGAGA

GGTATCAACAGTAGAATTATCATCAACAAATCTTGAATGACTTCACGGAT

GCCCTCCCCCTTATCATACTTTTGAAGAACCTACTTATGT-AAATCCTAA

ATAACGTATT--TTACCACAAGAAAGGAAGGATTTGAACCCCCTAAAATT

GGTTTCAAGCCAACACCATATCCACTATGA-CTTTCTCAATAA---TTAA

GATATTAGTAAAAT---TTACATAACTTTGTCAAAGTTAAATTATAGGCG

A------AACTCCTATATATCTT-TATGGCATACCCCTTCCAACTAGGTT

TCCAAGATGCAACATCTCCCATTATAGAAGAGTTATTAAGCTTTCACGAC

CATGCCCTAATAATTGTTTTCCTAATTAGTTCCCTCGTACTATATATTAT

TTCATCAATACTTACAACTAAACTAACTCATACCAACACTATAGACGCAC

AAGAAATCGAAACAATTTGAACTATCTTACCAGCCATAATCTTAATCATA

ATTGCACTTCCCTCATTACGAATTCTTTACATGATAGATGAAATAAATAA

TCCTTCTCTAACCATCAAAACCCTAGGCCACCAATGATATTGAAGCTATG

AGTATACAGATTATGAAGACTTAATATTTGATTCCTACATAGTACCCACC

TCAGAATTAAATCCGGGCCAGCTCCGCTTATTGGAAGTTGACAATCGAGT

AATTTTACCTGCTGAACTAACAATCCGAATACTAATCTCATCAGAAGATG

TTTTACACTCTTGAGCTGTTCCTTCTCTAGGGTTAAAAACAGACGCTATC

CCTGGTCGCTTAAACCAAACAACTCTACTTGCCACCCGACCAGGCTTATA

TTTCGGACAGTGCTCCGAAATCTGCGGATCTAACCATAGCTTTATACCCA

TCGTACTTGAAATAATTCCTTTAAAACATTTCGAAAAATGATCAACATCC

ATATTATAAC---------------------------------CTCATTA

AGAAGCTA---AATAGCACTAACCTTTTAAGTTAGAGATTGGAAGTTTTA

AACT-CCCCTTA--ATGATATGCCACAATTAGATACATCCACATGATCTA

TTATAATCACTTCAA-TAATCATTACACTATTCATTATATTTCAATTAAA

AATTTCAAAGCA--CTATTATTATAATAGCCCTGAACCCCTAACAACCAA

-ATTACAAAAACACTCAACCCCTTGAGAAACTAAATGAACGAAAATCTAT

TTGCCTCTTTCATTACTCCAACAATAATAGGATTACCTATTGTTGTACTT

ATTATTATATTCCCAAGTATATTATTCCCATCAACAGCACGACTGATTAA

CAACCGTTTAATCTCAATCCAACAGTGACTAATTCGCATGACAAC-AAAA

CAAATAATAACTATTCACAATAAAAAGGGACAAACTTGAACACTTATATT

AATTTCACTTATTATATTTATTGGTTCAACAAACCTCTTAGGCCTTTTAC

CCTACTCTTTTACTCCAACCACCCAACTATCAATAAACCTGGGCATAGCT

ATCCCCCTTTGAGCAGGCACAGTCATCCTAGGCTTTCGCCACAAAA-CAA

AAGCATCTTTAGCACACTTCTTACCTCAGGGAACACCACTACCCCTAATC

CCTATACTAATTATTATTGAAACAATTAGCCTATTCATTCAACCAATAGC

ACTAGCAGTACGACTTACAGCAAACATTACTGCAGGACACTTACTTATTC

ACTTGATTGGAGGAGCTACTCTAGCACTAATAAACATTAGCATAACCACT

GCTTTTATCACATTTATCATTCTAATCCTATTAACAGTACTAGAATTTGC

TGTTGCCCTAATTCAAGCATATGTCTTTACCCTACTAGTAAGTCTATATT

TACATGATAATACCTAATGACCCACCAGACACATGCCTACCACATAGTTA

ATCCAAGTCCTTGACCATTAACAGGAGCTCTATCAGCCCTTCTCCTAACA

TCCGGATTAGTTATATGATTCCACTTCGATTCCCCGCTCTTATTATTAAT

AGGTTTAACCACTAATATATTAACAATATATCAATGATGACGAGATATTG

TTCGAGAAAGTACATTCCAAGGACACCATACACCAATTGTACAAAAAGGC

CTTCGCTATGGAATGGTCTTATTTATTTTATCAGAAGTATTCTTTTTCTC

TGGCTTCTTCTGAGCTTTCTACCACTCAAGCTTAGCTCCCACACCAGAAT

TAGGAGGCTATTGACCCCCAGCAGGCATTATTCCCCTTAACCCCATAGAA

GTACCACTCCTAAATACATCCGTTCTATTAGCTTCCGGAGTATCTATTAC

CTGAGCTCACCATAGTCTAATAGAAGGAAATCGCATGCACACAATGCAAG

CATTACTAATTACTATTCTCCTAGGCCTGTATTTTACACTCTTACAGGCC

TCTGAATATTATGAAACATCATTTACTATTTCCGATGGTGTCTATGGATC

TACCTTTTTTATAGCTACAGGATTTCATGGCCTCCATGTCATTATTGGCT

CAACATTTCTTATCGTATGCTTTCTACGACAAATAAATTTTCACTTTACA

TCCAATCATCACTTCGGGTTTGAAGCTGCAGCCTGATACTGACATTTTGT

AGACGTCGTATGATTATTCTTATATGTCTCTATTTACTGATGAGGATCCT

ATTCT-TTTAGTATTTA-CTAGTACAACTGACTTCCAATCAGTTAGCCTT

GGA-AAACCCCAAGAAAGAATAATAAATTTTATTTTAACCCTAATTATTA

ATACCTTACTAGCCTCACTACTTGTAACAATCGCATTTTGACTCCCCCAA

GTAAATGTATATGCTGAAAAATCAAGTCCATACGAATGTGGCTTTGACCC

TATAGGCTCAGCTCGTCTTCCTTTTTCAATAAAATTCTTTCTAGTGGCAA

TTACTTTTTTATTATTCGATCTTGAAATCGCATTACTACTACCACTCCCA

TGGGCCTCTCAAACTGACAAACTAACAACCATACTACTTACATCTTTATT

CCTAATCTCTTTATTAATTATTAGCCTAACATACGAATGAACCCAAAAAG

GACTAGAATGGTCAGAATA------TGATAATTAGTTTAATATAAAA-TA

AATGATTTCGACTCATTAGATTATGACTACCTT-CATAATTATCAAT--A

TGTCTCTAACCCATATAAATATTTTATTAGCATTTACTACATCTCTTCTA

GGCCTACTTATATACCGATCACATCTAATATCTTCACTACTATGCCTAGA

AGGAATAATACTTTCCCTATTCGTCCTCACCGCTGTAACAATTCTTATTA

CCCATACAACTCTAGCCAGCATAATACCTATTATTCTCCTAGTATTCGCA

GCCTGTGAAGCAGCACTTGGACTATCACTACTAGTAGTTGTATCTAACAC

ATACGGAATCGACTATGTACAAAATCTTAACCTCCTTCAATGCTAAAAAT

TATCATTCCTACAATTATACTAATTCCACTTACATGACTATCAAAAAACA

ACATAATATGAATTAACTCAACAATCTACAGTTTAATAATCAGCATAACA

TGCTTACCCCTAATAAATCAACCCTGCGATAATAGCCTAAATTTGTCTGT

ATTATTTTTCTCCGATTCACTCTCAACTCCCCTATTAATATTAACAACCT

GACTCCTACCACTCATAATTATTGCTAGCCAATACCACATAACCAAAGAG

CCCCCTATACGAAAAAAACTATATATTACCATAATAATTTTACTTCAAAT

CTTTCTAATCATAACCTTTTCTGCCACCGAACTAATTATATTCTATATTC

TATTTGAAGCCACACTAGTACCAACTCTAATTATTATCACCCGATGAGGA

GGCCAAACAGAACGACTAAACGCTGGAGTCTATTTTCTTTTTTATACCCT

GGCTGGGTCATTACCACTTCTAATTGCCTTAATCTATACTCAAATCACCC

TAGGCTCACTAAATATGTTACTAACCCAGTACATGACCGAGCCACTTACC

TGCATT----------TGAAGCAGTTCACTCTTATGATTAGCATTTATGA

TAGCATTTATAGTAAAAATACCCCTCTATGGTCTCCACCTATGATTACCA

AAAGCTCATGTTGAAGCCCCAATTGCAGGCTCAATAGTCCTAGCAGCTAT

TTTATTAAAACTAGGCGGATACGGAATATTACGTATTACTATGATACTTA

ACCCCACCATGAATTTCATAACATACCCTTTCATAATATTATCCATATGA

GGTATAGTCATAACTAGCTCTATTTGCCTACGTCAAACAGACCTAAAATC

CCTGATTGCTTACTCATCTGTTAGTCACATAGCACTTGTAATTGTAGCCA

TTTTAGTTCAAAGCCCCTGAAGCTTTATAGGAGCTACCGCACTAATAATT

GCACATGGCCTAACATCCTCACTACTATTCTGCCTAGCAAACTCTAACTA

CGAACGAACTCACAGTCGAACCATAATTTTAGCTCGAGGCTTACAAACAA

TTCTTCCCCTAATAGCAGCCTGATGACTTCTGGCAAGTTTAACAAATTTA

GCTCTACCCCCATCTATCAATCTAATCGGAGAACTATTAATTACAGTATC

CATATTCTCATGATCTGGCTTTTCCATTATTCTACTAGGTATTAATATTA

CCATTACTGCCTTATATACACTCTACATACTAATTATAACCCAACGGGGC

AAATATACTTATCATATTCACAACATAAAACCCTCCTACACCCGAGAAAA

CACCCTAATATTACTACACCTCATACCCCTATTACTACTAATAACTAACC

CCAAAATTATCCTAGGAAATATATACTGTAAATATAGTTTAACAAAAACA

TTAGATTGTGAGTCTAACAATAGAAAC--ATAAAATT-CTTATTTACCGA

AAAAGAAT---GCAAGAACTGCTAACTCATGCCTCCGCATTTAAA-AATG

CGGCTT--------------------------------------------

-------------------TTTTAAGCTTTTAAAGGATAGGAGTT-ATCC

GTTGGTCTTAGGAACCAAAAA-A-TTGGTGCAACTCCAAATAAAAGTTAT

AAATC---TATTTTCCACTCTAATACTACTATCACTAATAATTCTTATAT

TACCCCTTATACTCCCCCTAAACAAATTTTCTAACCACCACCCCTACCCC

GAATATGCTAAAACGATAATTTCTTATTCTTTCATGATTAGCATACCCCC

AACTATCATATTCACCCAATCTGGCCAAGAAATAATAATCTCAAATTGAC

ATTGAATATCAATCCAAACCATAAAACTATCTCTTAGCTTTAAATTTGAT

TTTTTCTCCATAATATTCGTACCCGTAGCCTTATTCATTACTTGATCCAT

TATAGAATTCTCAATATGATATATACACCTAGACCCTAACATCAACCGAT

TTTTCAAATATCTATTAGTATTTCTAATTACTATATTAATTCTAGTTACA

GCTAATAATATCTTCCAACTTTTCATCGGCTGAGAGGGAGTAGGCATTAT

ATCTTTTCTACTAATTGGCTGATGATATGGACGAGCAGACGCTAACACAG

CTGCACTACAAGCAATCCTATATAATCGCATCGGAGACATCGGATTTATT

TTATCTATGGCATGACTAATAGCAAACTCAAACTCATGAGAACTTCAACA

AATTTTTATATTAAACTTAAATAATACCACCCTTCCACTCGCAGGCTTAC

TACTAGCCGCCACAGGAAAATCAGCCCAATTTGGACTACACCCATGACTA

CCTTCTGCCATAGAAGGACCTACGCCAGTCTCAGCCCTATTACACTCCAG

CACAATAGTAGTAGCGGGCATTTTCCTACTAATTCGATTTTACCCCCTGC

TA-GAGAATAACAAAATGATCCAATCACTAGCCCTATGCCTGGGAGCCAT

TACTACCCTATTCACAGCTATCTGTGCACTAACCCAGAACGATATTAAAA

AAATCGTAGCTTTTTCTACTTCAAGTCAACTAGGCCTAATAATAGTGACA

ATTGGCATCAACCAACCACACCTAGCATTTCTTCATATCTGCACACACGC

ATTCTTCAAAGCCATACTATTCCTATGCTCCGGATCCATCATTCATAGCC

TAAATGACGAACAAGACATTCGAAAAATAGGCGGACTATTCAAACCTTTA

CCATTCACAACTACTGCCCTAATCATTGGTAGTCTCGCATTAACAGGAAT

ACCATTCCTGACAGGATTCTACTCAAAAGACCTAATTATTGAAGCAGCAA

ATACATCCTACACAAACGCCTGAGCCCTTTTAATTACCCTCATTGCCACT

TCACTAACAGCTGTCTACAGCACTCGAATTATCTTCTTCGCATTACTTGG

AAAACCACGATTTCTCCCTTTAACCACAATTAATGAAAATAACCCCTTAC

TAATAAATTCTATCACACGACTACTAATTGGCAGCATCTTTGCCGGATTC

ATTATTTCTAATAACATAAATCCCTCAACCGTACCTCAAATAACTATACC

AATTCACTTAAAAATAATAGCCCTATTCGTAACCCTAACAGGTTTTATTA

TCGCCCTAGAACTTAACTACTTAACCCAAAACCTGAAATACAAACACCCC

TCAACTACATTT-AAATTCTCAACTATATTAGGATACTTTCCACTCACCA

TGCATCGCCTAAGCCCCTTGACAAACTTAACTATTGGCCAAAAATCAGCT

ACTCTATTACTTGACTTAATCTGACTAGAAAATATTCTACCAAAACTAAT

TATTAAAACCCAA---CAAAACTTTTCAACTACAGTAACCAACCAAAAAG

GACTAGTCAAATTATATTTTCTCTCCTTCCTAGTCACTATTACCACAACC

CTTTTTATATTTA------------------------------ATTTCCA

CGCGTAATTTCCAAAATAATTACTACACCAATAAATAAAGACCACCCAGT

AATAACTACTAGTCAGCTCCCATAACTATATAATGCAGCAACCCCTATCG

ACTCTTTAGCCAACACTCCAAAATCACCCACATCATAAATTGCTCAATCC

CCAATTTCATTAAATTCAAAAACCACCTCCAA---------TTTCTCAAC

AACCAACACATACAAAATCAACATAAACTCTATCATCAAGCCAACAATAA

ATGACCCCAGTACCACCACATTAGAGACCCAAACTTCAGGATATTGCTCC

ATAGCTATAGCTGTTGTGTATCCAAAAACTACTAATATTCCCCCCAAGTA

GATTAAAAACACTATTAAACCTAAAAATGACCCACCAAAACTCACAACAA

TCCCACAACCAACCCCACCACTCACAATCAACCCAACACCCCCATAAATA

GGAGAAGGCTTAGAAGAAAACCCTACAAAACCAATTATAAAAATAATACT

TAAAACAGATACAATGTATACTATCATTATTCCTG-CATGGATTACAT-C

CACGACTAGTGACACGAAAAATCACCGTTGTAT-TTCAACTACAAGAACA

---GACAAATGACCAACATTCGAAAATCTCACCCCCTAATAAAAATTATT

AACAACTCATTCATTGATCTACCCACCCCATCAAATATCTCCTCCTGATG

AAACTTTGGCTCTCTTTTAGGAATCTGCCTAACACTACAAATCACAACAG

GACTATTTCTAGCTATACACTACACATCAGACACCGCAACAGCCTTCAAC

TCAGTCACCCATATCTGTCGAGACGTAAATTACGGCTGAATCCTGCGCTA

CCTCCATGCAAACGGAGCCTCCATATTTTTCATTTGTCTATACCTCCATG

TAGGACGAGGGCTTTATTATGGCTCCTATATATATACAGAAACTTGAAAC

ATTGGAGTCATTCTACTATTTACTGTAATAGCAACAGCTTTCATAGGATA

TGTACTCCCATGGGGCCAAATATCCTTCTGAGGAGCAACTGTAATTACTA

ATCTACTATCCGCAATCCCATATATTGGCACAGACCTTGTAGAATGGATC

TGAGGTGGCTTCTCTGTCGACAAAGCTACTCTAACTCGATTCTTTGCCTT

TCACTTTTTACTTCCATTCATCATTACAGCTATAGTTATAGTACATCTTT

TATTCCTCCATGAAACAGGGTCCAACAATCCAATAGGAATCCCCTCTAAC

GCAGACATAATCCCCTTTCACCCTTACTACACAATCAAAGACATCCTCGG

CCTATTAGCAATAATTACAACACTACTAACACTAGTATTATTCTCCCCAG

ACATACTAGGAGACCCCGACAATTACACACCAGCAAACCCATTAAGTACT

CCACCCCATATTAAACCAGAATGATACTTCCTATTCGCATACGCAATCCT

ACGATCAATTCCAAATAAATTAGGAGGAGTACTAGCTCTAATTCTATCAA

TTCTTATCCTAATTATTATCCCCCTACTTCATACATCCAAACAACGTAGC

ATAGCCTTTCGCCCCCTAAGCCAATGCTTGTACTGACTACTAGTAGCAGA

CCTCCTAACTTTAACATGAATTGGAGGACAACCTGTCGAATACCCATTCG

TCATCATTGGACAACTCGCATCAATCCTCTATTTCTCTATTATTATTATC

CTAATACCACTCACCAGCCTACTAGAAAATCACCTATTAAAATGAAGA--

--GTCTCTGTAGTATATTA---ATTATACTGGTCTTGTAAATCAGAGAAG

GGGAAAAC--ATTCCCCCAAAGACTC-------AAGAGGAAGGCTCATAC

CCTGCCATCAGCACCCAAAGCTGATATTCTAA--TTAAACTACCTCCTG-

--------------------------------------------------

--------------------------------------------------

--------------------------------------------------

--------------------------------------------------

--------------------------------------------------

--------------------------------------------------

--------------------------------------------------

--------------------------------------------------

--------------------------------------------------

--------------------------------------------------

--------------------------------------------------

--------------------------------------------------

--------------------------------------------------

--------------------------------------------------

--------------------------------------------------

--------------------------------------------------

--------------------------------------------------

--------------------------------------------------

--------------------------------------------------

--------------------------------------------------

--------------------------------------------------

--------------------------------------------------

--------------------------------------------------

------

>Myotis_myotis Myotis myotis mitochondrion, complete genome.

GTTAATGTAGCTTAATGC--------TAAAGCAAGGCACTGAAAATGCCT

AGATGAGTATATTT--ACTCCATAAACACATA--GGTTTGGTCCTGGCCT

TTCTATTAGTTATCAGTAAACTTACACATGCAAGAATCCTCGTCCCAGTG

AGAATGCCCT-CTACACCGATAAT----GGTAAA-AAGGAGCTGGTATCA

AGCACACT---ATAA-GTAGCTCATAACACCTCGC-TCAGCCACACCCCC

ACGGGATACAGCAGTGATAAAAATTAAGCA-ATAAATGAAAGTCTGACTA

AGTTATACTA----TTCAGGACTGGTAAATCTCGTGCCAGCCACCGCGGT

CATACGATTAGGCCAAACTAATAGACA-CTCGGCGTAAAGCGTGTTTTAG

AAAATAA----ATAATAAATAAAATCGAACTCTAACTAAGCTGTAAAAAG

CCTCAGATACTG--TAAGATATACAACGAAAGTGATTTTA---AAAAATC

TGACTACACGATAGCTAAGACCCAAACTGGGATTAGATACCCCACTATGC

TTAGCCCTAAACATAAAAAATTATA---AACAAAATTATTCGCCAGAGTA

CTACTAGCAATAGCTTAAAACTCAAAGGACTTGGCGGTGCCTTATATCCA

TCTAGAGGAGCCTGTTCTATAATCGATAAACCCCGATCTACCTCACCAGT

CTTTGCTAA-ATCAGCCTATATACCGCCATCTTCAGCAAACCCTAA-AAA

GGAATTAAAGTAAGCACAAGTATTA---ACATAAAAACGTTA-GGTCAAG

GTGTAGCTTATAGAATGGA-AAGAAATGGGCTACATTCCTCGAGTCGAGG

AATAAAACT----CTTATACGAAAACCTATGTGAAATCAAA---AGTTAA

AGGTGGATTTAGTAGTAAACT-AAGAATAGAGAGCTTAGTTGAATAT-GG

CCATAAGGCACGCACACACCGCCCGTCACCCTCCTCAAATAAAACAATAT

TAAATATATCTTAGATAATATATATACATAGATATTGACACATGAGAGGA

GACAAGTCGTAACAAGGTAAGTGTACTGGAAAGTGCACTTGGATAATACA

AAGTGTAGCTTAA---ATA-AAAGCACCTAGTTTACACCTAGAAGATTTC

A-TATAAACTGAGCGCTTTGACAACCGAATAT-AGCTCAAAA--TATATT

AAATAATATTACTCTAA-AACATTA--AAATAAAACATTCACA-TTAAA-

-ACAAAAGTATAGGCGATAGAAATTC-----TATTTGACGCTATAGAGAA

AGTACCGCAAGGGAAATAATGAAAGAA-AACTCAACAGTAAAAAAAAGCA

AAGTTTAGTCCTTGTACCTTTTGCATAA-TGACTCAACTAGAAAACA-TT

AGCAAAAAG-AATTTAAGTTAAATACCCCGAAACTAGACGAGCTACCCAT

TAGCAGCTT-AA--AGAGCAAACTCGTCTATGTGGCAAAATAGTGAGAAG

ACTAATGGGTAGGGGCAATAAACCTACCGAGCCTAGTGATAGCTGGTTGT

CCA-GAACAGAATTTTAGTTCTAATTTAAGCCTACTATAAAAAAAAAC--

AAATTTTAACGTATGCTTAAATAATAATCTAAAAGGGTTCAGCTTTTTAG

ATATG-GGATACAACCCCTATTAGTGAGTAAACAAAAAATTAAAACA---

CCCTAGTTGGCCTAAAAGCAGCCATCAAACAAGATAGCGTTCAAGCTCAA

C------AATATACTAAAATCAATTTCAACTATTAACAATGATA--CT--

CCTAATATAA-AACTGGACTATTCTATTTTTAAAT-AGAAGTAATAATGT

TGGCATGAGTAACAAGAAACAT---TTCTCCTCGCATGCGTGTATATCAG

AGCGAA-TAAATCACTGATAGTTAACAA----ACATAAATATAACTATCA

AACTAAAAA----TCATAATAT-TATTGTTAGCCCAACACAGGTATGCA-

--GCTAAGGAAAGATTAAAAAAAGTAAAAGGAACTCGGCAAATACAAACC

CCGCCTGTTTACCAAAAACATCACCTCTAGCATAAAAAGTATTAGAGGCA

TTGCCTGCCCAGTGACTTCA-G----TTCAACGGCCGCGGTATCCTGACC

GTGCAAAGGTAGCATAATCACTTGTTCTCTAAATAGGGACTTGTATGAAT

GGCTCAACGAGGGTTTTACTGTCTCTTACTTTTAATCAGTGAAATTGACC

TTCCCGTGAAGAGGCGTGAATAAAAAAATAAGACGAGAAGACCCTATGGA

GCTTTAATTCACCAATTCACAAATTATAACACCACTCTAC---------T

AGAAGC--AAACTTATTTTAATTGAATTGACAATTTAGGTTGGGGTGACC

TCGGAATAAAAACTAACTCCCGAGAAAATTTCAATT--AAGACAAACAAG

TCAAGATTACTCCATTA---TATATTGATCCGTTAAA---AACGATCAAC

GAAACAAGTTACCCTAGGGATAACAGCGCAATCCTATTTAAGAGTCCATA

TCGACAATTAGGGTTTACGACCTCGATGTTGGATCAGGACATCCCAATGG

TGCAGCCGCTATTAATGTGTTCGTTTGTTCAACGATTAA-AGTCCTACGT

GATCTGAGTTCAGACCGGAGAAATCCAGGTCGGTTTCTATCTATTTTAAA

-GTCTCTCCTAGTACGAAAGGACAAGAGAGACAGGGCCCACTTTAAATAA

GTGCCCTACTTGAT--TAGATGAA-TTAATCTTAATCTTATAATACATAT

AA--------ATACCCGAGAACAGGG--CTTAGTTAAAGTGGCAGAGACC

GGTAA-TTGCATAAAACTTAAGCTTTTAGAGTCAGAGGTTCAACTCCTCT

CTTTAACAATCTC-------------------------------------

------------------------------------------ATGTACTA

TATCAATTTATTAATAATAATTGTTCCTATTTTACTAGCGGTAGCATTCT

TAACTCTACTGGAACGAAAAGTGCTAGGCTATATACAACTTCGAAAAGGG

CCTAACATTGTAGGTCCCTATGGCCTATTACAACCAATCGCCGATGCAGT

CAAACTATTTACTAAAGAACCCATACAACCACTGACATCATCTCTCATTC

TATTTATCATTGCACCAACTCTAGCCCTAACCCTAGCCCTAATAATATGA

ATTCCACTACCTATACCACACCCATTAATTAATATAAACCTAAGCATTCT

ATTTATATTAGCCCTATCAAGTTTAGCTGTCTACGCCATTTTATGATCAG

GATGAGCTTCAAATTCAAAATACGCACTAATTGGGGCCCTACGAGCAGTA

GCTCAAACAATTTCTTATGAAGTAACCCTAGCTATCATTGTCTTATCTAT

CCTACTTATAAATGGCTCCTTTACATTAACAACATTAATCACAACACAAG

AATATATCTGATTAATTATCCCCTCATGGCCTCTAGCTATAATATGATTT

ATCTCAACTCTAGCAGAAACTAATCGAGCTCCCTTTGACTTAACGGAAGG

TGAATCAGAATTAGTATCTGGCTTTAATGTAGAATATGCAGGAGGACCAT

TCGCCCTTTTTTTCCTAGCAGAATATGCAAATATTATTATAATAAACACC

CTCACAATTATTCTATTTTTAGGCGCATATAATAATCCAATATTTCCAGA

ACTATACACCATTAATCTCACCACTAAAACCCTTTTATTTACAACAATTT

TTTTATGGGTCCGAGCATCCTATCCCCGATTTCGATATGATCAACTAATA

CACTTACTATGAAAAAACTTCCTACCCCTCACCCTAGTAATATGCATGTG

GCATGTAACTCTGCCAATTATTCTGGCAAGCATTCCACCTCTAACATAA-

GAAATATGTCTGATAATAGAGTTACTTTGATAGAGTAAATCATAGGGGTT

TA-AATCCCCTTATTTCTAGAATCACAGGAATTGAACCTGCTCTTAAGAA

TTCAAAAATCTTCGTGCTACCCACATTACACTATACTCTAA---------

GTAAGGTCAGCTAAATTAAGCTATCGGGCCCATACCCCGAAAATGTTGGT

TTATATCCTTCCCGTACTAATTAATCCCATAACCCTGTCACTAGTATTAG

CAACAATATTCTCAGGCACTCTAATTGTCATAACAAGCTCACACTGATTT

ATAATTTGAGTAGGGTTTGAAATAAACATACTAGCCATTATCCCTCTGTT
[truncated: 157,276 more chars]
