# Supplementary material for: Extension of Mitogenome Enrichment Based on Single Long-Range PCR: mtDNAs and Putative Mitochondrial-Derived Peptides of Five Rodent Hibernators
Source: Front Genet. 2021 Dec 13;12:685806. doi: 10.3389/fgene.2021.685806 (PMC8749263; doi:10.3389/fgene.2021.685806)
Supplement: Supplementary file 1 [file DataSheet1.zip › Table S1.docx]

**Supplementary Table S1.** Analysis of sequence variation for two mtDNAs of *Cricetus cricetus* (black-bellied or common hamster)

| **Mitotype origin** | **Austria** | | | | **Caucasus (Russia)** | | | |
| --- | --- | --- | --- | --- | --- | --- | --- | --- |
| **GenBank accession no.** | MF405145.2 (this study) | | | | NC_037888.1 | | | |
| **mtDNA length** (bp) | 16,264 | | | | 16,263 | | | |
| **Nucleotide frequency** (%) | T(U) | C | A | G | T(U) | C | A | G |
|  | 31 | 26 | 30 | 13 | 28.23 | 27.15 | 30.62 | 13.99 |
| **Genes** | **Start** | **Stop** | **Strand** | **Length** | **Start** | **Stop** | **Strand** | **Length** |
| *trnF(ttc)* | 1 | 66 | + | 66 | 1 | 66 | + | 66 |
| *rrnS* | 67 | 1,017 | + | 951 | 67 | 1,017 | + | 951 |
| *trnV(gta)* | 1,018 | 1,089 | + | 72 | 1,018 | 1,089 | + | 72 |
| *rrnL* | 1,088 | 2,649 | + | 1,562 | 1,088 | 2,649 | + | 1,562 |
| *trnL2(tta)* | 2,650 | 2,724 | + | 75 | 2,650 | 2,724 | + | 75 |
| *MT-ND1* | 2,722 | 3,672 | + | 951 | 2,722 | 3,672 | + | 951 |
| *trnI(atc)* | 3,682 | 3,750 | + | 69 | 3,682 | 3,750 | + | 69 |
| *trnQ(caa)* | 3,748 | 3,818 | - | 71 | 3,748 | 3,818 | - | 71 |
| *trnM(atg)* | 3,823 | 3,891 | + | 69 | 3,823 | 3,891 | + | 69 |
| *MT-ND2* | 3,892 | 4,911 | + | 1,020 | 3,892 | 4,911 | + | 1,020 |
| *trnW(tga)* | 4,925 | 4,991 | + | 67 | 4,925 | 4,991 | + | 67 |
| *trnA(gca)* | 4,994 | 5,063 | - | 70 | 4,994 | 5,063 | - | 70 |
| *trnN(aac)* | 5,066 | 5,135 | - | 70 | 5,066 | 5,135 | - | 70 |
| *trnC(tgc)* | 5,167 | 5,234 | - | 68 | 5,167 | 5,234 | - | 68 |
| *trnY(tac)* | 5,325 | 5,303 | - | 67 | 5,325 | 5,303 | - | 67 |
| *MT-CO1* | 5,303 | 6,841 | + | 1,539 | 5,303 | 6,841 | + | 1,539 |
| *trnS2(tca)* | 6,845 | 6,913 | - | 69 | 6,845 | 6,913 | - | 69 |
| *trnD(gac)* | 6,917 | 6,984 | + | 68 | 6,917 | 6,984 | + | 68 |
| *MT-CO2* | 6,985 | 7,665 | + | 681 | 6,985 | 7,665 | + | 681 |
| *trnK(aaa)* | 7,672 | 7,736 | + | 65 | 7,672 | 7,736 | + | 65 |
| *MT-ATP8* | 7,738 | 7,932 | + | 195 | 7,738 | 7,932 | + | 195 |
| *MT-ATP6* | 7,899 | 8,573 | + | 675 | 7,899 | 8,573 | + | 675 |
| *MT-CO3* | 8,579 | 9,361 | + | 783 | 8,579 | 9,361 | + | 783 |
| *trnG(gga)* | 9,363 | 9,431 | + | 69 | 9,363 | 9,431 | + | 69 |
| *MT-ND3* | 9,432 | 9,776 | + | 345 | 9,432 | 9,776 | + | 345 |
| *trnR(cga)* | 9,783 | 9,850 | + | 68 | 9,783 | 9,850 | + | 68 |
| *MT-ND4L* | 9,855 | 10,145 | + | 291 | 9,855 | 10,145 | + | 291 |
| *MT-ND4* | 10,142 | 11,509 | + | 1,368 | 10,142 | 11,509 | + | 1,368 |
| *trnH(cac)* | 11,520 | 11,586 | + | 67 | 11,520 | 11,586 | + | 67 |
| *trnS1(agc)* | 11,587 | 11,644 | + | 58 | 11,587 | 11,645 | + | 59 |
| *trnL1(cta)* | 11,644 | 11,713 | + | 70 | 11,645 | 11,714 | + | 70 |
| *MT-ND5* | 11,723 | 13,504 | + | 1,782 | 11,724 | 13,517 | + | 1,794 |
| *MT-ND6* | 13,524 | 14,042 | - | 519 | 13,525 | 14,043 | - | 519 |
| *trnE(gaa)* | 14,043 | 14,111 | - | 69 | 14,044 | 14,112 | - | 69 |
| *MT-CYB* | 14,116 | 15,249 | + | 1,134 | 14,117 | 15,250 | + | 1,134 |
| *trnT(aca)* | 15,260 | 15,326 | + | 67 | 15,261 | 15,327 | + | 67 |
| *trnP(cca)* | 15,330 | 15,395 | - | 66 | 15,331 | 15,396 | - | 66 |
| *MOTs-c* | 757 | 843 | + | 28 | 757 | 843 | + | 28 |
| *SHLP2* | 1,609 | 1,684 | + | 24 | 1,609 | 1,684 | + | 24 |
| *SHLP4* | 1,874 | 1,942 | - | 22 | 1,874 | 1,942 | - | 22 |
| *SHLP6* | 2,408 | 2,438 | - | 9 | 2,408 | 2,438 | - | 9 |

Orange box: change of sequence length
