# Supplementary material for: Extension of Mitogenome Enrichment Based on Single Long-Range PCR: mtDNAs and Putative Mitochondrial-Derived Peptides of Five Rodent Hibernators
Source: Front Genet. 2021 Dec 13;12:685806. doi: 10.3389/fgene.2021.685806 (PMC8749263; doi:10.3389/fgene.2021.685806)
Supplement: Supplementary file 1 [file DataSheet1.zip › Table S2.docx]

**Supplementary Table S2.** Comparison of mitogenomes of the cryptic *Muscardinus* *avellanarius* (hazel dormouse) species

| **Lineage (sample origin)** | **Western Europe (Belgium)** | | | | **Central-Eastern Europe and Anatolia (Denmark)** | | | |
| --- | --- | --- | --- | --- | --- | --- | --- | --- |
| **GenBank accession no.** | MN935778.1 (this study) | | | | NC_050264 | | | |
| **mtDNA length** | 16,727 bp | | | | 16,731 bp | | | |
| **Nucleotide frequency (%)** | T(U) | C | A | G | T(U) | C | A | G |
|  | 31.30 | 23.03 | 33.09 | 12.58 | 32.07 | 22.16 | 33.38 | 12.40 |
| **Genes** | **Start** | **Stop** | **Strand** | **Length** | **Start** | **Stop** | **Strand** | **Length** |
| *trnF(ttc)* | 1 | 66 | + | 66 | 1 | 67 | + | 67 |
| *rrnS* | 67 | 1,027 | + | 961 | 68 | 1,027 | + | 965 |
| *trnV(gta)* | 1,028 | 1,095 | + | 68 | 1,033 | 1,100 | + | 68 |
| *rrnL* | 1,094 | 2,661 | + | 1,568 | 1,099 | 2,666 | + | 1,568 |
| *trnL2(tta)* | 2,662 | 2,737 | + | 76 | 2,667 | 2,742 | + | 76 |
| *MT-ND1* | 2,740 | 3,690 | + | 951 | 2,745 | 3,695 | + | 951 |
| *trnI(atc)* | 3,696 | 3,763 | + | 68 | 3,701 | 3,769 | + | 69 |
| *trnQ(caa)* | 3,761 | 3,833 | - | 73 | 3,767 | 3,839 | - | 73 |
| *trnM(atg)* | 3,838 | 3,906 | + | 69 | 3,844 | 3,912 | + | 69 |
| *MT-ND2* | 3,907 | 4,944 | + | 1,038 | 3,913 | 4,938 | + | 1,026 |
| *trnW(tga)* | 4,949 | 5,015 | + | 67 | 4,955 | 5,021 | + | 67 |
| *trnA(gca)* | 5,020 | 5,087 | - | 68 | 5,026 | 5,093 | - | 68 |
| *trnN(aac)* | 5,094 | 5,166 | - | 73 | 5,100 | 5,172 | - | 73 |
| *trnC(tgc)* | 5,200 | 5,266 | - | 67 | 5,206 | 5,272 | - | 67 |
| *trnY(tac)* | 5,267 | 5,331 | - | 65 | 5,273 | 5,338 | - | 66 |
| *MT-CO1* | 5,338 | 6,876 | + | 1,539 | 5,347 | 6,885 | + | 1,539 |
| *trnS2(tca)* | 6,881 | 6,950 | - | 70 | 6,890 | 6,959 | - | 70 |
| *trnD(gac)* | 6,958 | 7,026 | + | 69 | 6,967 | 7,035 | + | 69 |
| *MT-CO2* | 7,028 | 7,708 | + | 681 | 7,037 | 7,717 | + | 681 |
| *trnK(aaa)* | 7,716 | 7,782 | + | 67 | 7,725 | 7,791 | + | 67 |
| *MT-ATP8* | 7,784 | 7,966 | + | 183 | 7,793 | 7,975 | + | 183 |
| *MT-ATP6* | 7,945 | 8,619 | + | 675 | 7,954 | 8,628 | + | 675 |
| *MT-CO3* | 8,625 | 9,407 | + | 783 | 8,634 | 9,416 | + | 783 |
| *trnG(gga)* | 9,409 | 9,478 | + | 70 | 9,418 | 9,486 | + | 69 |
| *MT-ND3* | 9,485 | 9,823 | + | 339 | 9,487 | 9,831 | + | 345 |
| *trnR(cga)* | 9,831 | 9,899 | + | 69 | 9,840 | 9,908 | + | 69 |
| *MT-ND4L* | 9,922 | 10,194 | + | 273 | 9,931 | 10,203 | + | 273 |
| *MT-ND4* | 10,191 | 11,558 | + | 1,368 | 10,200 | 11,567 | + | 1,368 |
| *trnH(cac)* | 11,569 | 11,638 | + | 70 | 11,578 | 11,647 | + | 70 |
| *trnS1(agc)* | 11,639 | 11,699 | + | 61 | 11,648 | 11,708 | + | 61 |
| *trnL1(cta)* | 11,700 | 11,769 | + | 70 | 11,709 | 11,778 | + | 70 |
| *MT-ND5* | 11,776 | 13,563 | + | 1,788 | 11,785 | 13,581 | + | 1,797 |
| *MT-ND6* | 13,581 | 14,099 | - | 519 | 13,590 | 14,108 | - | 519 |
| *trnE(gaa)* | 14,100 | 14,169 | - | 70 | 14,109 | 14,178 | - | 70 |
| *MT-CYB* | 14,174 | 15,307 | + | 1,134 | 14,183 | 15,316 | + | 1,134 |
| *trnT(aca)* | 15,313 | 15,383 | + | 71 | 15,322 | 15,392 | + | 71 |
| *trnP(cca)* | 15,385 | 15,450 | - | 66 | 15,394 | 15,459 | - | 66 |
| *MOTs-c* | 768 | 837 | + | 22 | 768 | 837 | + | 22 |
| *Humanin* | 2,058 | 2,067 | + | 2 | 2,063 | 2,072 | + | 2 |
| *SHLP4* | 1,870 | 1,909 | - | 12 | 1,875 | 1,914 | - | 12 |
| *SHLP6* | 2,418 | 2,433 | - | 9 | 2,423 | 2,438 | - | 9 |

Orange box: change of sequence length
