# Supplementary material for: Extension of Mitogenome Enrichment Based on Single Long-Range PCR: mtDNAs and Putative Mitochondrial-Derived Peptides of Five Rodent Hibernators
Source: Front Genet. 2021 Dec 13;12:685806. doi: 10.3389/fgene.2021.685806 (PMC8749263; doi:10.3389/fgene.2021.685806)
Supplement: Supplementary file 1 [file DataSheet1.zip › Table S4.docx]

**Supplementary Table S4.** Species used for phylogenetic reconstruction

| **Latin name** | **Common name at NCBI** | **GenBank accession number** | **Submission date** | **Reference** |
| --- | --- | --- | --- | --- |
| *Bos primigenius indicus* | Cattle | NC_005971.1 | 07-JUN-2021 | Xia et al., 2021 |
| *Bos primigenius taurus* (Yakutia) | Cattle | MT576760.1 | 25-MAY-2021 | Xia et al., 2021 |
| *Burramys parvus* | Mountain pygmy possum | KJ868103.1 | 29-NOV-2016 | Mitchell et al., 2014 |
| *Cheirogaleus medius* | Lesser dwarf lemur | NC_021945.1 | 09-JUN-2016 | Finstermeier et al., 2013 |
| *Cricetus cricetus* (AUT) | Black-bellied hamster | MF405145.2 | 21-JUL-2021 | This study |
| *Cricetus cricetus* (RUS) | Black-bellied hamster | NC_037888.1 | 14-JUL-2018 | Ding et al., 2020 |
| *Dromiciops gliroides* | Monito del monte | NC_005826.1 | 01-FEB-2010 | Nilsson et al., 2003 |
| *Eliomys quercinus* | Garden dormouse | MN935777.1 | 11-MAY-2021 | This study |
| *Glis glis* (*Myoxus glis*) | Fat dormouse | NC_001892.1 | 06-JAN-2011 | Reyes et al., 1998 |
| *Homo sapiens* | Human | NC_012920.1 | 02-SEP-2020 | Andrews et al., 1999 |
| *Homo sapiens* (FIN) | Human | HQ221886.1 | 25-JUL-2016 | Direct submission |
| *Ictidomys tridecemlineatus* | Thirteen-lined ground squirrel | NC_027278.1 | 24-JUN-2015 | Zhang et al., 2016 |
| *Lynx lynx* | Eurasian lynx | MH706704 | 18-OCT-2021 | Wu et al., 2018 |
| *Lynx lynx* | Eurasian lynx | MK229208.2 | 18-OCT-2021 | Direct submission |
| *Marmota himalayana* | Himalayan marmot | NC_018367.1 | 12-JUN-2014 | Chao et al., 2014 |
| *Marmota marmota* | European marmot | MN935776.1 | 22-MAR-2021 | This study |
| *Marmota monax* | Woodchuck | LR632920.1 | 06-SEP-2019 | Direct submission |
| *Mesocricetus auratus* | Golden hamster | NC_013276.1 | 07-JUN-2021 | Direct submission |
| *Muscardinus avellanarius* (BEL) | Hazel dormouse | MN935778.1 | 22-MAR-2021 | This study |
| *Muscardinus avellanarius* (DEN) | Hazel dormouse | NC_050264.1 (or MT410887) | 26-SEP-2020 | Margaryan et al., 2021 |
| *Mustela siberica* | Siberian weasel | AP017415.1 | 24-MAR-2017 | Shalabi et al., 2017 |
| *Mustela siberica* | Siberian weasel | AP017418.1 | 24-MAR-2017 | Shalabi et al., 2017 |
| *Myotis bechsteinii* | Bechstein's bat | NC_034227.1 | 13-APR-2017 | Jebb et al., 2017 |
| *Myotis myotis* | Mouse-eared bat | NC_029346.1 | 10-MAR-2016 | Jebb et al., 2017 |
| *Panthera tigris altaica* | Amur tiger | MN624080 | 27-JAN-2020 | Direct submission |
| *Panthera tigris sumatrae* | Sumatran tiger | JF357969.1 | 27-FEB-2012 | Kitpipit et al., 2012 |
| *Plecotus auritus* | Brown big-eared bat | HM164052.1 | 25-JUL-2016 | Direct submission |
| *Spermophilus citellus* | European suslik | MN935779.1 | 22-MAR-2021 | This study |
| *Tachyglossus aculeatus* | Australian echidna | NC_003321.1 | 01-FEB-2010 | Janke et al., 2002 |
| *Urocitellus parryii* | Arctic ground squirrel | MN935780.1 | 22-MAR-2021 | This study |
| *Urocitellus richardsonii* | Richardson's ground squirrel | NC_031209.1 | 12-OCT-2016 | Direct submission |

Black: hibernators, blue: cold-adapted individual; grey: not cold-adapted and not hibernating phenotype

Country abbreviations: AUT (Austria), BEL (Belgium), FIN (Finland), and DEN (Denmark)

Yakutia: province in north-eastern Siberia (Russian Federation)

References

Andrews *et al*. (1999) *Nat Genet* 23: 147

Chao *et al*. (2014) *Genet Mol Res* 13(2): 2739-51

Ding *et al.* (2020) *Genomics* 112(2): 1716-25

Finstermeier *et al.* (2013) *PloS One* 8(7): e69504

Janke *et al.* (2002) *J Mol Evol* 54(1): 71-80

Jebb *et al.* (2017) *Mitochondrial DNA B Resour* 2(1): 92-4

Margaryan *et al*. (2021) *Environ DNA* 3(2): 472-80

Mitchell *et al*. (2014) *Mol Biol Evol* 31(9): 2322-30

Nilsson *et al*. (2003) *J Mol Evol* 57(1): S3-S12

Reyes *et al*. (1998) *Mol Biol Evol* 15(1): 499-505

Shalabi *et al*. (2017) *Biol J Linn Soc* 120(2): 333-48

Xia *et al*. (2021) *Heredity* 126(6): 1000-8

Zhang *et al*. (2016) *Mitochondrial DNA A DNA Mapp Seq Anal* 27(4): 2608-9
