# Supplementary material for: Extension of Mitogenome Enrichment Based on Single Long-Range PCR: mtDNAs and Putative Mitochondrial-Derived Peptides of Five Rodent Hibernators
Source: Front Genet. 2021 Dec 13;12:685806. doi: 10.3389/fgene.2021.685806 (PMC8749263; doi:10.3389/fgene.2021.685806)
Supplement: Supplementary file 1 [file DataSheet1.zip › Table S5.docx]

**Supplementary Table S5.** Summary on mitochondrial-derived peptides (MDPs)

| **MDP** | **Strand** | **Experimental validation** | **No. of amino acids*** | |
| --- | --- | --- | --- | --- |
|  |  |  | Human | Target hibernators^†^ |
| gau | light | Immunohistochemistry (Faure et al., 2011) | 100 | no ORF |
| Humanin | heavy | ELISA (Muzumdar et al., 2009; Woodhead et al., 2020) | 24 | no ORF |
| MOTs-c | heavy | Western blot (Lee et al., 2015)  Mass spectrometry (Knoop et al., 2019)  ELISA (Reynolds et al., 2021) | 16 | 19, 21, 22, 25, 31^‡^ |
| SHLP1 | light | Northern blot (Cobb et al., 2016)  Western blot (Cobb et al., 2016)^§^ | 24 | no ORF |
| SHLP2 | light | Northern blot (Cobb et al., 2016)  Western blot (Cobb et al., 2016)^§^  ELISA (Xiao et al., 2017; Woodhead et al., 2020) | 26 | 8 or no ORF^‡^ |
| SHLP3 | light | Northern blot (Cobb et al., 2016)  Western blot (Cobb et al., 2016)^§^ | 38 | no ORF |
| SHLP4 | light | Northern blot (Cobb et al., 2016)  Western blot (Cobb et al., 2016)^§^ | 26 | 12, 35^‡^ |
| SHLP5 | light | Northern blot (Cobb et al., 2016) | 24 | no ORF |
| SHLP6 | heavy | Northern blot (Cobb et al., 2016)  Western blot (Cobb et al., 2016)^§^  ELISA (Woodhead et al., 2020) | 20 | 9, 20^‡^ |

*****Translated according to the nuclear genetic code; ^†^target hibernator models of this study; ^‡^for details see Supplementary Figure 2; ^§^Negative control: HeLa- ρ0 (Rho 0, mtDNA-less) cells

References

Cobb *et al.* (2016) *Aging* 8(4): 796-808

Faure *et al.* (2011) *Biol Direct* 6: 1-22

Knoop *et al.* (2019) *Rap Comm in Mass Spectr* 33(4): 371-80

Lee *et al.* (2015) *Cell Metab* 21(3): 443-54

Reynolds *et al.* (2021) *Nat Commun* 12(1): 1-12

Woodhead *et al.* (2020) *J Appl Physiol* 128(5): 1346-54

Xiao *et al.* (2017) *Oncotar* 8(55): 94900-9
